# New insights into the role of the CHI3L2 protein in invasive ductal breast carcinoma

**DOI:** 10.1038/s41598-024-77930-5

**Published:** 2024-11-18

**Authors:** Agnieszka Rusak, Ewa Kątnik, Tomasz Górnicki, Christina Schmuttermaier, Krzysztof Kujawa, Aleksandra Piotrowska, Katarzyna Ratajczak-Wielgomas, Alicja Kmiecik, Andrzej Wojnar, Piotr Dzięgiel, Julia Kzhyshkowska

**Affiliations:** 1 Division of Histology and Embryology, Department of Human Morphology and Embryology, Wroclaw Medical University, T. Chalubinskiego St. 6a, 50-368 Wroclaw, Poland; 2 Institute of Transfusion Medicine and Immunology, Medical Faculty Mannheim, Heidelberg University, Ludolf-Krehl Street 13-17, 68167 Mannheim, Germany; 3 German Red Cross Blood Service Baden-Württemberg–Hessen, Ludolf-Krehl Street 13-17, 68167 Mannheim, Germany; 4 Statistical Analysis Centre, Wroclaw Medical University, K. Marcinkowskiego 2-6 St, 50-368 Wroclaw, Poland; 5 Department of Preclinical Sciences, Pharmacology and Diagnostics, Faculty of Medicine, Wroclaw University of Science and Technology, Hoene-Wronskiego 13 C St, 58-376 Wroclaw, Poland; 6 Department of Physiotherapy, University School of Physical Education, I. Paderewskiego 35 Al, 51-612 Wroclaw, Poland

**Keywords:** Chitinase-like proteins, CHI3L2, CHI3L1, Invasive ductal breast carcinoma, ERK1/2, STAT-3, Cancer, Cell biology, Molecular biology

## Abstract

Chitinase-like proteins have multiple biological functions that promote tumor growth, angiogenesis and metastasis. Expression of CHI3L2, which is similar in structure to CHI3L1, is detected in glioma cells and tumor-associated macrophages (TAMs) in glioma and breast cancer. However, its exact role remains unclear. We analyzed the expression of CHI3L2 in 74 invasive ductal breast carcinoma (IDC) tumors, breast cancer and macrophages cell cultures using immunohistochemistry, immunofluorescence, Western blot and PCR methods. Clinicopathologic data were included in the analysis. The results obtained show that CHI3L2 expression decreases with increasing degree of tumor grade and negative status of estrogen (ER) and progesterone receptors (PR). Furthermore, CHI3L2 is significantly and positively correlated with phosphorylation of STAT-3 and ERK1/2 signaling pathways, but negatively correlated with macrophage infiltration. CHI3L2 is expressed both in the cytoplasm of cancer cells and in macrophages and may regulate STAT-3 and ERK1/2 phosphorylation in breast cancer cell lines. Analysis of the clinicopathologic data revealed that CHI3L2 levels had no effect on patient survival. CHI3L2 expression may be specific for cancer cells in IDC and involved in cross-talk with the tumor microenvironment. Our study has shown that IDC cancer cells express the CHI3L2 protein, possibly indicating a novel function of this protein.

**Supplementary Information:**

The online version contains supplementary material available at 10.1038/s41598-024-77930-5.

## Introduction

CHI3L2 (chitinase 3-like 2 protein; YKL-39) is a 39 kDa lectin that belongs to the chitinase-like protein family and shows high homology and structural similarity to CHI3L1. Of the chitinases found in human tissues, only mammalian acid chitinase and chitotriosidase exhibit chitinase activity. However, chitinase-like proteins, including CHI3L1, CHI3L2 and SI-CLP (stabilin-1 interacting chitinase-like protein), have biological functions that contribute to stimulation of cell proliferation, support of angiogenesis and tumor invasion, cell interactions and immunomodulation^1–7^. The best-known protein in this family is undoubtedly CHI3L1, also known as YKL-40. Its established role in promoting angiogenesis and involvement in development of various cancers, such as breast cancer, colorectal cancer and glioblastoma is well documented^8–11^. In addition, it is involved in bypassing the cancer immune system and in pathological inflammation in various diseases, including Alzheimer’s and Parkinson’s disease, multiple sclerosis and even asthma^12–18^.

Elevated levels of the CHI3L1 protein have been detected in the serum of cancer patients, including those with gliomas, colorectal, lung and breast cancer as well as leukemia. These elevated levels are usually associated with poor prognosis, tumor growth and metastasis^6^. In our previous study, we have shown that increased CHI3L1 expression in IDC tumors can promote angiogenesis and be associated with the growth of triple-negative tumors^19^. Furthermore, our results show a correlation between the expression of CHI3L1 and Nogo-A in IDC and their involvement in angiogenesis in this cancer subtype^20^. Recent studies also indicate increased levels of CHI3L1 in bronchoalveolar lavage fluid and serum of critical COVID-19 patients, suggesting the possible involvement of this protein in the pathogenesis of the disease and its importance as a biomarker^21–23^.

The significance of CHI3L2 in breast cancer is still largely unknown. There are only a limited number of articles pointing to its importance in the expression of TAMs. Recent studies emphasize the central role of this protein in promoting angiogenesis and supporting distant metastasis^3^,^24^. In fact, it is more likely that the source of CHI3L2 protein in breast tumor tissue is macrophages.

In this article, the expression of CHI3L2 in the cytoplasm of IDC and breast cancer cell lines is presented for the first time, and the influence of CHI3L2 protein on the phosphorylation of STAT-3 and ERK1/2 signaling pathways is discussed. Furthermore, the correlation between CHI3L2 expression and clinicopathological data of patients diagnosed with IDC was analyzed. Our results reveal a novel role for chitinase-like proteins. CHI3L2 may have a protective function against tumors that differs from CHI3L1 despite the structural similarity of the two proteins.

## Materials and methods

### Cell lines

Human cancer cell lines were cultured from various clinicopathological breast tumors, including MCF-7, SK-BR-3, T-47D, BT-474, B-T549, MDA-MB-231, MDA-MB-436, MDA-MB-468, and BO2 (all from ATCC, American Type Culture Collection ATCC, Old Town Manassas, VA, USA)^25^,^26^. The MCF10A cell line, which is derived from fibrocystic breast disease, and the HME1-hTERT (Me16C) (ATCC) cell line, which is normal mammary epithelial cells immortalized with hTERT, were also used. Cells were cultured in appropriate media: α-MEM (Sigma-Aldrich) for MCF-7, BO2 and BT-474, L-15 medium (ATCC) for MDA-MB-231 and MDA-MB-468, EMEM (Lonza) for SK-BR-3, L-15 (ATCC) supplemented with 10 µg/ml insulin and 16 µg/ml glutathione (both from Sigma-Aldrich) for MDA-MB-436, RPMI-1640 (Gibco, Thermo-Fisher Scientific) supplemented with 0.1% insulin (Sigma-Aldrich) for BT-549. The human acute monocytic leukemia cell line THP-1 (ATCC) was cultured in RPMI-1640 medium (Gibco, ThermoFisher Scientific, Wilmington, DE, USA). All media were supplemented with fetal bovine serum (FBS) up to 10% (Sigma-Aldrich) and penicillin–streptomycin solution up to 1% of the final volume (Sigma-Aldrich). For MCF10A and hTERT (Me16C), the MEGM ™ bullet kit (Lonza) was used for cell culture. Cell passaging was performed with TrypLE (Gibco, ThermoFisher Scientific) at a maximum confluence of 70%. The media were changed twice a week. Cell culture was performed under standard conditions in a humidified atmosphere with 5% CO2. The molecular characteristics of the individual breast cancer lines according to Kao et al.^27^ are listed in Table 1.

**Table 1:** Expression of CHI3L2 in breast cancer cell lines. The characteristics of the human breast cancer cell lines used in the study are described according to Kao et al. ^27^.

Cell line	ER	PR	HER2	Tumor type	Subtype	CHI3L2
HME1-hTERT (Me16C)	–	NA	–	Normal mammary gland	–	+
MCF-7	+	+	–	Metastatic adenocarcinoma	Luminal A	–
MCF10A	–	–	–	Fibrcoystic disease	Basal	+
SK-BR-3	–	–	+	Adenocarcinoma	Luminal B-like (HER2-subtype)	–
T-47D	+	+	-	Invasive ductal breast carcinoma	Luminal A	–
BT-474	+	+	+	Invasive ductal breast carcinoma	Luminal B	–
BT-549	–	–	–	Papillary ductal breast carcinoma	Basal	+
MDA-MB-231	–	–	–	Metastatic adenocarcinoma	Basal	+
MDA-MB-436	–	–	–	Adenocarcinoma	Basal	–
MDA-MB-468	–	–	–	Metastatic adenocarcinoma	Basal	–
BO2	–	–	–	Metastatic adenocarcinoma	Basal	+

ER: estrogen receptor, PR: progesterone receptor, HER2: human epidermal growth factor receptor 2; NA-not analyzed.

### Monocyte differentiation

THP-1 cells were cultured for 24 h in complete RPMI-1640 medium (Gibco), supplemented with 100 nM PMA (phorbol 12-myristate 13-acetate) (Sigma-Aldrich)^28^,^7^. Subsequently, adherent macrophages were identified at the bottom of the vessel.

### Co-cultures

Macrophages were cultured for one week in conditioned media of MCF-7, MDA-MB-468, MCF 10A and MDA-MB-231 cells. Macrophage pellets were then harvested and frozen at -80°C for protein expression analysis. 

### Transfections experiment

In turn, effect of CHI3L2 siRNA on ERK1/2 and STAT-3 patchways phosphorylations were investigated in two cell lines characterised by expresion of this protein: BT-549 and MDA-MB-231. Cells were seed on 6-well plate in amount 3 × 10^5^ per well. Next day, transfection were provided with 2 µg siRNA CHI3L2 (cat. AM16708, ID: 119,204, ThermoFischer Scientific) and X-tremeGENE siRNA Transfection Reagent (Roche, Mannheim, Germany) (proportion 1:5) in 2 ml of Opti-MEM medium (Gibco). Cells without siRNA were used as controls. After 24 and 48h, cells were harvested for molecular analysis and stored in −80 °C.

### CHI3L2 recombinant protein

Infuence of exogenous CHI3L2 were next analysed on breast cancer cell lines without expression of this protein. First, MD-MB-468 and BT-474 cells were seeded on 6-well plates in amount 3 × 10^5^ in dedicated media. Next day, media were removed and CHI3L2 human recombinant protein (cat. 5112-CH, R&D Systems, Minneapolis, MN, USA) was added to cell culture in amount 3 µg per well (1.5 µg/ml), in 2 ml of Opti-MEM medium (Gibco). After 24 and 48h, cells were harvested for molecular analysis and stored in -80 °C.

### Patients and tumors

The 74 samples of IDC used in this study were archival material from patients diagnosed and operated on at the Lower Silesian Oncology Center (Wroclaw, Poland) between 1999 and 2009. As a control, 12 non-malignant breast tissue lesions (NBTL) were included in the study. Breast cancer samples were graded according to the WHO system^29,30^ and tissue samples were either embedded in paraffin or stored at −80 °C.

### Immunohistochemical reactions

Immunohistochemistry was performed on 4 µm sections of paraffin-embedded tissue samples using the Dako Autostainer Link48 (Dako, Glostrup, Denmark). Paraffin removal and antigen retrieval were performed in PT-Link (Dako) with basic EnVision FLEX Target Retrieval Solution (97 °C, 20 min; Dako) for immunostaining of CHI3L2, CD68 and CD206, CHI3L1, VEGFA, VEGFD, CD31, CD34, estrogen receptor (ER), progesterone receptor (PR) and human epidermal growth factor receptor 2 (HER2). The VEGFC and Ki-67 antibodies were treated with Dako’s acidic EnVision FLEX Target Retrieval Solution. Dako’s EnVision FLEX Peroxidase Blocking Reagent was used to block endogenous peroxidase for 5 min. The EnVision FLEX + Mouse, High pH System (Dako) was used to visualize IHC reactions by mouse monoclonal antibodies: CHI3L2 (1:100 with linker, MAB5116, R&D, Minneapolis, Minnesota, USA), CD206 (1:100, MAB25341, R&D), CD31 (RTU, Dako; 20 min), CD34 (RTU, Dako; 20 min), VEGFA (1:50, Dako 18 h, 4°C), Ki-67 (RTU, Dako, 20 min), ER (RTU, Dako, 20 min) and PR (RTU, Dako, 20 min).

The LSAB + detection system (Dako) was used to visualize IHC reactions obtained with goat polyclonal antibodies against CHI3L1 (1:100, AF2599, 20 min; R&D Systems, Minneapolis, MN USA), VEGFC (1:100, 18 h, 4°C; ReliaTech GmbH, Braunschweig, Germany) and VEGFD (1:100, 18 h, 4°C; ReliaTech GmbH). HER2 expression was measured using the HercepTest kit (Dako). Counterstaining with hematoxylin (5 min) was performed (EnVision FLEX Hematoxylin). The visualization systems were used according to the manufacturer’s instructions. A secondary HRP-conjugated rat antibody was applied at a dilution of 1:400 (Jackson ImmunoResearch) and incubated for 1 h at room temperature (RT). EnVision FLEX/HRP was also applied and incubated for 1 h at RT (Dako). EnVisionFLEX Substrate Buffer was then added and incubated for 10 min at RT (Dako)^19^,^20^,^26^,^31^,^32^.

### Evaluation of the immunohistochemical reactions

The immunohistochemical reactions for CHI3L2, CHI3L1, VEGFA, VEGFC and VEGFD were evaluated using the 12-point immunoreactive score (IRS) developed by Remmele and Stegner^33^ (Table 2).

**Table 2:** Semi-quantitative immunoreactive scale (IRS) according to Remmele and Stegner^33^, in which the result of the multiplication of the percentage of positively stained cells (A) and the intensity of the immunohistochemical reaction (B), (AxB) gives a score.

Points (AxB)	Percentage of positive cells (A)	Intensity of immunohistochemical reaction (B)
0	0%	0: no color reaction
1	≤ 10%	1: weak reaction
2	11–50%	2: moderate reaction
3	51–80%	3: intense reaction
4	> 80%	

For the evaluation of the pan-macrophage marker CD68 and the anti-inflammatory macrophage CD206, the positively tested cells were counted in five hotspots with a × 200 magnification and the average value was given. The Chalkley’s ophthalmoscope (Pyser Inc., Edenbridge, UK) was used to score CD31- and CD34-positive blood vessels based on the number of grid points (n = 1–25) and to estimate the relative microvessel count (MVC)^34–36^. Methodologically, the basis for the relative microvessel density (MVD) was determined by taking the mean of the three hotspots with the highest vascularization (magnification × 200), according to the approach of Fox and Harris^36^. A semi-quantitative scale from 0 to 4 was used for Ki-67 expression: 0 for 0% of positive cells, 1 for 1–10% of positive cells, 2 for 11–25% of positive cells, 3 for 26–50% of positive cells and 4 for 51–100% of positive cells^37^. For this marker, high expression was defined as values > 25% of positive cells, while low expression was considered ≤ 25%. For estrogen (ER), progesterone (PR) and human epidermal growth factor receptor 2 (HER2), a semi-quantitative scale of 0–3 was used: 0 means 0% of positive cells, 1 means 1–10% of positive cells, 2 means 11–50% of positive cells and 3 means 51–100% of positive cells. For HER2, a positive response was detected when more than 10% of cancer cells showed an intense membrane response (score of 3)^38^. CHI3L2 protein expression and its correlation with the clinicopathological data of the patients are shown in Table 3. For the markers CHI3L1, CHI3L2, VEGFA, VEGFC, VEGFD, CD31, CD34, CD68 and CD206, the median expression values were used as cut-off points for the analysis of low and high expression (Table 4). The immunohistochemical reactions were analyzed using a BX41 light microscope (Olympus, Tokyo, Japan).

**Table 3:** The expression of the protein chitinase 3-like 2 (CHI3L2) was measured using the immunoreactive score (IRS)^33^ and its correlation with the clinicopathological data of patients diagnosed with IDC was analyzed. The cut-off point was determined using the median value.

		Total		IRS (0–6)		IRS (8–12)	
Clinicopathological data		N 74	% 100	N 39	% 52.7	N 35	% 47.3
Age	> 50	21	28.4	13	17.6	8	10.8
	≤ 50	53	71.6	27	36.5	26	35.1
Menopausal status	Pre-	22	29.7	12	16.2	10	43.3
	Post-	44	59.5	23	31.1	21	28.4
	Unknown	8	10.8	4	5.4	4	5.4
ER status	Positive	45	60.8	20	27.0	25	33.8
	Negative	21	28.4	16	21.6	5	6.8
	Unknown	8	10.8	3	4.0	5	6.8
PR status	Positive	40	54.1	17	23.0	23	31.1
	Negative	26	35.1	19	25.7	7	9.4
	Unknown	8	10.8	3	4.0	5	6.8
HER2 status	Positive	15	20.3	12	16.2	3	4.1
	Negative	51	68.9	24	32.4	27	35.6
	Unknown	8	10.8	3	4.0	5	6.8
Triple negative (TN)	Yes	10	13.5	7	9.4	3	4.1
	No	50	67.6	25	33.8	25	33.8
	Unknown	14	18.9	7	9.4	7	9.4
Tumor grade (G)	1	7	9.5	1	1.4	6	8.1
	2	38	51.3	21	28.4	17	22.9
	3	27	36.5	17	23.0	10	13.5
	Unknown	2	2.7	0	0	2	2.7
Stage	I	19	25.7	10	13.5	9	12.2
	II	37	50.0	19	25.7	18	24.3
	III	9	12.2	6	8.1	3	4.1
	IV	1	1.3	0	0	1	1.3
	Unknown	8	10.8	4	5.4	4	5.4
Tumor size (pT)	pT0	12	16.2	7	9.4	5	6.8
	pT1	29	39.2	14	19.0	15	20.2
	pT2	21	28.4	10	13.5	11	14.9
	pT3	6	8.1	5	6.8	1	1.3
	pT4	4	5.4	2	2.7	2	2.7
	Unknown	2	2.7	1	1.3	1	1.3
Lymph node (pN)	pN0	42	56.8	24	32.4	18	24.3
	pN1	16	21.6	7	9.4	9	12.2
	pN2	5	6.8	3	4.0	2	2.7
	pN3	3	4.0	1	1.3	2	2.7
	Unknown	8	10.8	4	5.4	4	5.4
Ki-67	Low ≤ 25%	29	39.2	15	20.3	14	18.9
	High > 25%	9	12.2	8	10.8	1	1.3
	Unknown	36	48.6	16	21.6	20	27.0

ER: estrogen receptor; PR: progesteron receptor; HER2: human epidermal growth factor receptor 2; TN: triple negative (no expression of ER, PR and HER2 receptor).

**Table 4:** Immunohistochemical analysis was performed using the IRS scale according to Remmele and Stegner^33^ to evaluate the expression of the markers. Microvessel density (MVC) was assessed using the Chalkley ocular^34^. The number of macrophages was assessed in the field of view under 200 × magnification. The median value was used as the cut-off value.

		Low expresion		High expression	
IHC marker	Cut-off value	N	%	N	%
CHI3L2	IRS 0–6 vs. 8–12	39	52.7	35	47.3
CHI3L1	IRS 0–3 vs. 4–12	49	66.2	25	33.8
VEGFA	IRS 0–4 vs. 6–12	47	63.5	27	36.5
VEGFC	IRS 0–4 vs. 6–12	48	64.9	26	35.1
VEGFD	IRS 0–4 vs. 6–12	42	56.8	32	43.2
CD31	MVC ≤ 8 vs > 8	43	50.0	43	50.0
CD34	MVC ≤ 9 vs > 9	44	59.5	30	40.5
CD68	≤ 86.4 >	38	51.4	36	48.6
CD206	≤ 43.75 >	38	51.4	36	48.6

CHI3L1: chitinase-3 like 1; CHI3L2: chitinase-3 like 2; VEGFA: vascular endothelial growth factor A; VEGFC: vascular endothelial growth factor C; VEGFD: vascular endothelial growth factor D.

### Immunofluorescence reactions

Immunofluorescence reactions were performed on 4 µm thick tissue sections. Deparaffinization was performed with a graded alcohol series at room temperature, and epitope retrieval was performed at 90 °C for 15 min at pH = 9.0. For blocking, a 1% BSA solution in PBS/0.1% Tween 20 was used for 30 min at RT. Slides were then incubated overnight at 4 °C with anti-CHI3L2 anti-rat antibody (1:5, #18H10)^24^ and mouse monoclonal antibody (1:500, MAB5116, R&D). Incubation with secondary anti-rat and anti-mouse antibodies conjugated to the fluorochromes Alexa568 (Jackson ImmunoResearch) and Alexa 488 (ab150113, Abcam, Cambridge, UK) was performed for 1 h at RT (dilution 1:2000 in 1% BSA in PBS/0.1% Tween20). Slides were coverslipped with FluoroShield mounting medium with DAPI (Abcam, Cambridge, UK, ab104139). A confocal microscope (Olympus Fluoview FV3000, Tokyo, Japan) and imaging software (Olympus Cell Sense) were used to analyze the immunofluorescence reactions^26,39^.

### Western blot

Tissue samples were digested in T-PER Tissue Protein Extraction Reagent using a TissueRuptor homogenizer (Qiagen, Hilden, Germany), while cell lysates were homogenized in RIPA buffer. For protein isolation, 5 mM PMSF (phenylmethanesulfonyl fluoride), EDTA and Heat™ Protease Inhibitor Coctail × 100 (all Thermo Scientific, Wilmington, DE, USA) were added. The bicinchoninic acid assay (Pierce BCA Protein Assay Kit) was used to determine total protein content, which was measured using the NanoDrop1000 (Thermo Fisher). Samples were denatured in a buffer consisting of 250 mM TRIS at pH 6.8, 40% glycerol, 20% (v/v) β-mercaptoethanol, 0.33 mg/ml bromophenol blue and 8% sodium dodecyl sulfate (SDS) for 10 min at 95 °C. To perform SDS-PAGE, a Mini Protean 3 instrument (Bio-Rad, Hercules, CA, USA) was used with a 10% polyacrylamide gel for total protein and 12% for VEGFA and VEGFC; 30 μg of protein^40^,^41^ was applied per lane. We then performed a wet transfer in Tris–glycine buffer containing 20% methanol and 0.05% SDS onto a PVDF 0.45 µm (polyvinylidene difluoride membrane) (Immobilon, Millipore, Bedford, MA, USA) for a 1h transfer at 140 V to separate the proteins. For VEGFA and VEGFC proteins, 0.20 µm nitrocellulose membranes (Bio-Rad) were used at transfer conditions of 0.5 h at 70 V. The blocker used was 5% skim milk in 0.05% TBST or 5% BSA in 0.05% TBST for CHI3L1.

Incubation with primary antibodies was performed overnight at 4 °C at the following concentrations: CHI3L2 (mouse monoclonal antibody, MAB5116, R&D, 1:1000 in buffer group 1 recommended by producer), STAT-3 (rabbit polyclonal antibody, 1:1000 in 0.1%, ab68153, Abcam, Cambridge, UK), p-STAT-3 (rabbit monoclonal antibody, 1:2000 in 5% milk in TBST 0.05%, ab76315, Abcam), ERK1/2 (rabbit monoclonal, 1:10 000, ab184699, Abcam), pERK1/2 (rabbit monoclonal, 1:1000, ab201015, Abcam), β-tubulin (rabbit polyclonal, 1:1000, ab6046, in 0.1% BSA in 0. 1% TBST, ab6046, Abcam,), VEGFA (mouse monoclonal, 1:1000, M7273, Dako, Glostrup, Denmark), VEGFC (mouse monoclonal, 101-M90, ReliaTech, Wolfenbüttel, Germany), VEGFD (mouse monoclonal, MAB286, R&D)^7^.

For the secondary HRP-conjugated antibodies, incubation was performed for 1 h at RT. Donkey anti-mouse (1:3000), donkey anti-rabbit (1:6000), both in 5% milk in 0.05% TBST (Jackson ImmunoResearch, Suffolk, UK) or donkey anti-goat (1:10,000 in TBST, Jackson ImmunoResearch) were used. Super-Signal West Femto chemiluminescence substrate from ThermoFisher was used for the chemiluminescence reaction. Visualization was performed using Bio-Rad’s ChemiDocTM MP system with Bio-Rad’s ImageLab software (exposure time from 1 s to 3 min). Densitometric analysis was based on independent triplicates using ImageLab software (Bio-Rad). Reference protein was β-tubulin, except for pSTAT-3 and pERK1/2, where STAT-3 and ERK1/2 levels, respectively, served as reference^42^.

### The Droplet Digital PCR™ (ddPCR)

For determination of the absolute number of genes mRNA copies in the analyzed cells, the Droplet digital PCR (ddPCR) method was applied. Isolation of total RNA was provided with RNeasy Mini kit (Qiagen, Hilden, Germany), and reverse transcription (RT-PCR) with iScript™ Reverse Transcription Supermix for RT-qPCR (Bio-rad, Hercules, 345 CA, USA). From each sample, 70 ng of RNA was reverse transcribed with the C1000 Touch Thermal Cycler (Bio-Rad). Reactions were provided in the conditions as follows: priming (5 min, 25°C), reverse transcription (20 min, 46°C), inactivation of reverse transcriptase (1 min, 95°C). The ddPCR reaction mixtures were consisted of 2.3 μl of RT product, 1 μl of TaqMan specific probe (Applied Biosystems, Foster City, CA, USA), 6.7 μl of molecular biology-grade water and 10 μl of 2X ddPCR™ MasterMix for Probes (Bio-Rad). In the experiment, the TaqMan-specific probes were applied to assessed mRNA expression of investigated gene Hs00970220_m1 (CHI3L2). Next, 20 μl of the reaction mixtures were transferred into a plastic cartridge (Bio-Rad) with addition of 50 μl of Droplet Generation Oil for Probes (Bio-Rad) in the QX100 Droplet Generator (Bio-Rad). Finally, droplets get from each sample were loaded on 96-well PCR plate (Eppendorf, Hamburg, Germany). PCR amplifications were provided in a C1000 Touch Thermal Cycler (Bio-Rad) with conditions: enzyme activation (10 min, 95°C), followed by 40 cycles of denaturation (30 s, 94°C) and annealing/extension (1 min, 60°C), enzyme deactivation (10 min, 98°C), ending (10 min, RT). At the end, the plate was transferred to Droplet Reader (Bio-Rad) and automatically reading were applied. The absolute quantification of each mRNA was obtained from the number of positive counts per panel with the Poisson distribution. The quantification of the target mRNA was visualised as the number of copies/μl (AQ) in the PCR reaction mixture^7,41^.

### mRNA expression profile

For the transcriptomic analysis of tumor material we used the database GSE3494 of the Gene Expression Omnibus Portal. This database contains clinical data and a complete microarray evaluation of gene expression for 251 breast cancer cases (G1: N = 67, G2: N = 128, G3: N = 54) and uses the GPL96 [HG-U133A] Affymetrix Human Genome U133A Array (Thermo Fischer)^43^. Patients underwent surgery in Sweden between 1987 and 1989. The raw gene expression data were normalized using the global mean approach, where the logarithmic signal values were transformed and scaled with log 500 as the target signal value. In our study, we examined CHI3L2 expression data from breast cancer tumors with different tumor grades (G1, G2, G3) and estrogen (ER) and progesterone receptor (PR) expression.

### Institutional Review Board Statement

The study was conducted in compliance with the Declaration of Helsinki and was approved by the Bioethics Committee of WROCLAW MEDICAL UNIVERSITY (No. KB-517/2017, approved on July 22, 2017; KB-217/2022 and No. KB-216/2022, both approved on March 15, 2022).

### Statistical analysis

The normal distribution was analyzed using the Shapiro–Wilk test. As the variables had a non-normal distribution, a non-parametric test was used for the statistical analysis. The expression of the markers was analyzed using Spearman’s and Kendall’s tau -b correlation, the Kruskal–Wallis test with post-hoc Dunn test and the Mann–Whitney U test. The Breslow, Kaplan–Meier and Cox hazard regression models were used to examine the survival rates. The proportionality assumption was met for all predictors except pT, which was converted to a categorical variable in the results. For analysis of results obtained with ddPCR, Kruskal–Wallis test with post-hoc Dunn test was applied. Statistically significant p-values were defined at a level ≤ 0.05. Statistical analyzes were performed using Statistica 13.3 (Tibco, Palo Alto, CA, USA).

## Results

### CHI3L2 expression in *cancer* cells is higher in lower tumors grade ER + and PR + and is not associated with angiogenesis and macrophage infiltration

The clinicopathologic data and the results of the immunohistochemical reactions are shown in Table 3.

Cytoplasmic expression of CHI3L2 in cancer cells in IDC was detected by immunohistochemistry. Low expression of this protein occurred in 52.7% of cases, while high expression occurred in 47.3% of cases (Table 4).

**Fig. 1:**
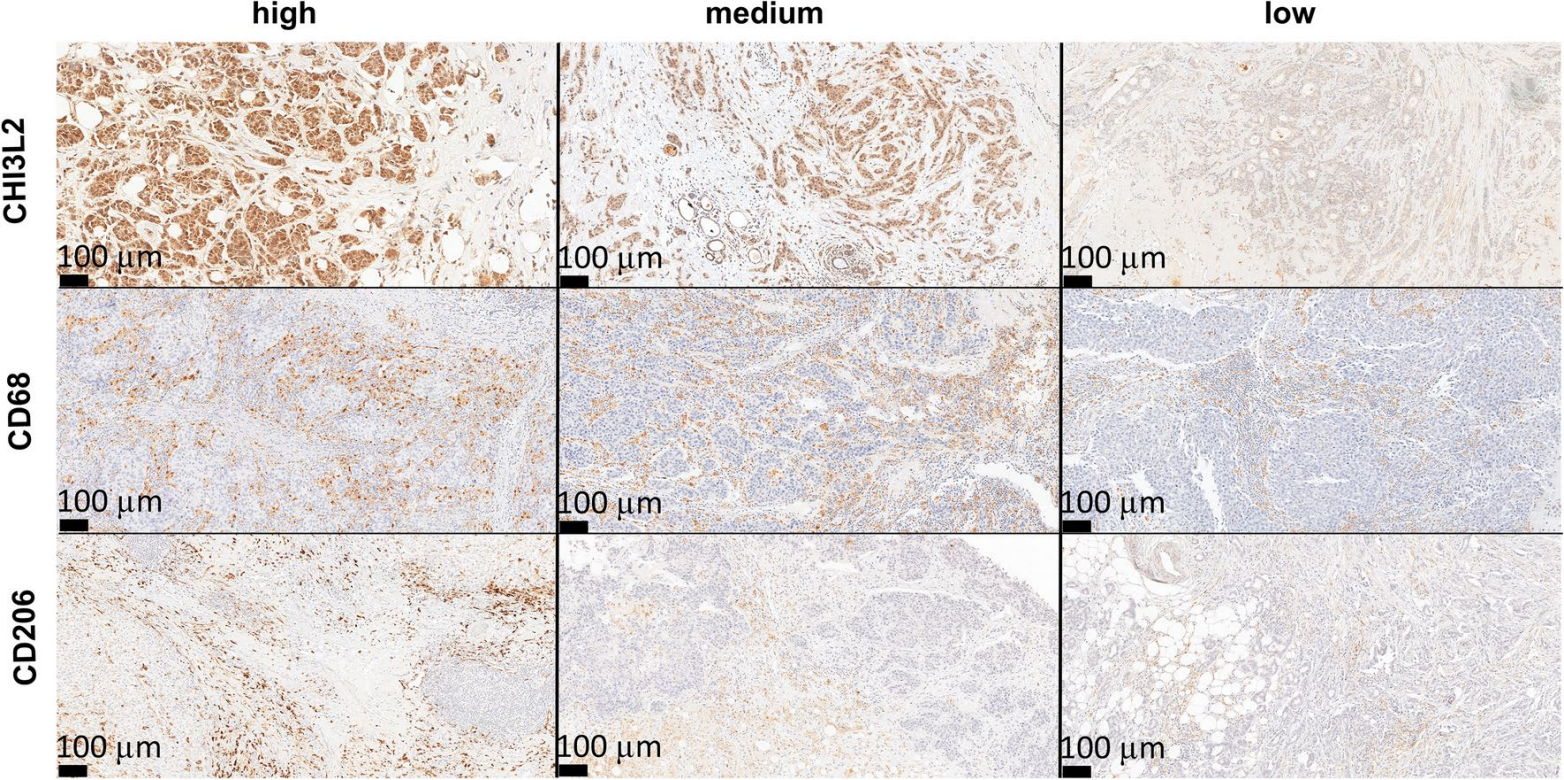
Immunohistochemical reactions show differential expression of CHI3L2 in the cancer cells, CD68 pan-macrophage markers and CD206-positive TAMs, magnification 100x.

The differentiation of the expression levels of CHI3L2 and other markers detected by immunohistochemical reactions is shown in Fig. 1.

**Fig. 2:**
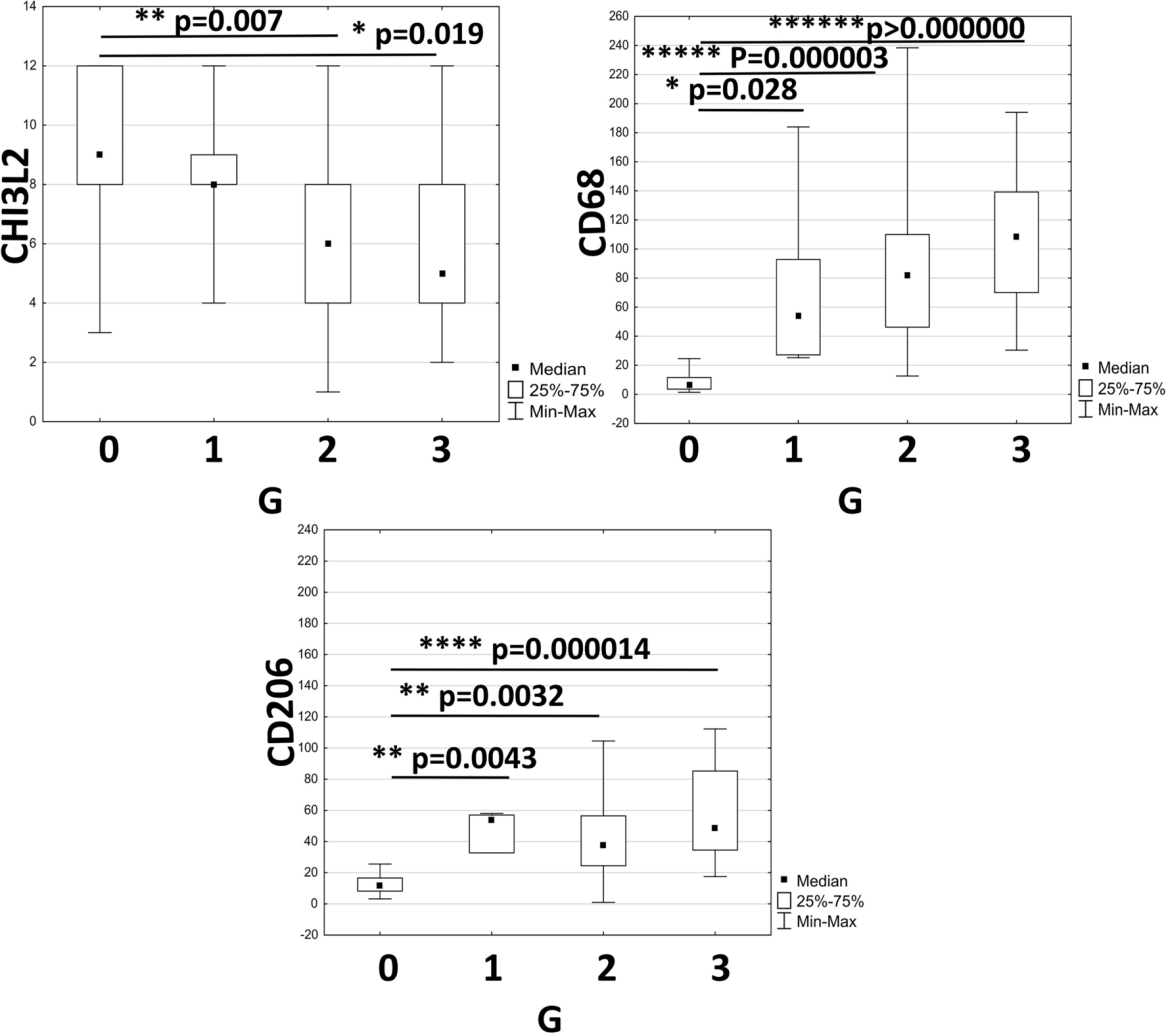
Analysis of immunohistochemical expression of CHI3L2, CD68 and CD206 in tumors of different malignancy grade (G1, G2, G3; G0: non-malignant breast tissue lesion (NBTLs); Kruskal–Wallis test followed by Dunn test; statistically significant p-values were < 0.05.

The expression level of CHI3L2 was highest in non-malignant breast tissue lesions (NBTLs) and decreased significantly with higher tumor grades. Conversely, the expression of CD68, indicative of macrophage infiltration, and CD206 (specific for an anti-inflammatory macrophage phenotype) increased with malignancy grade (Fig. 2).

**Fig. 3:**
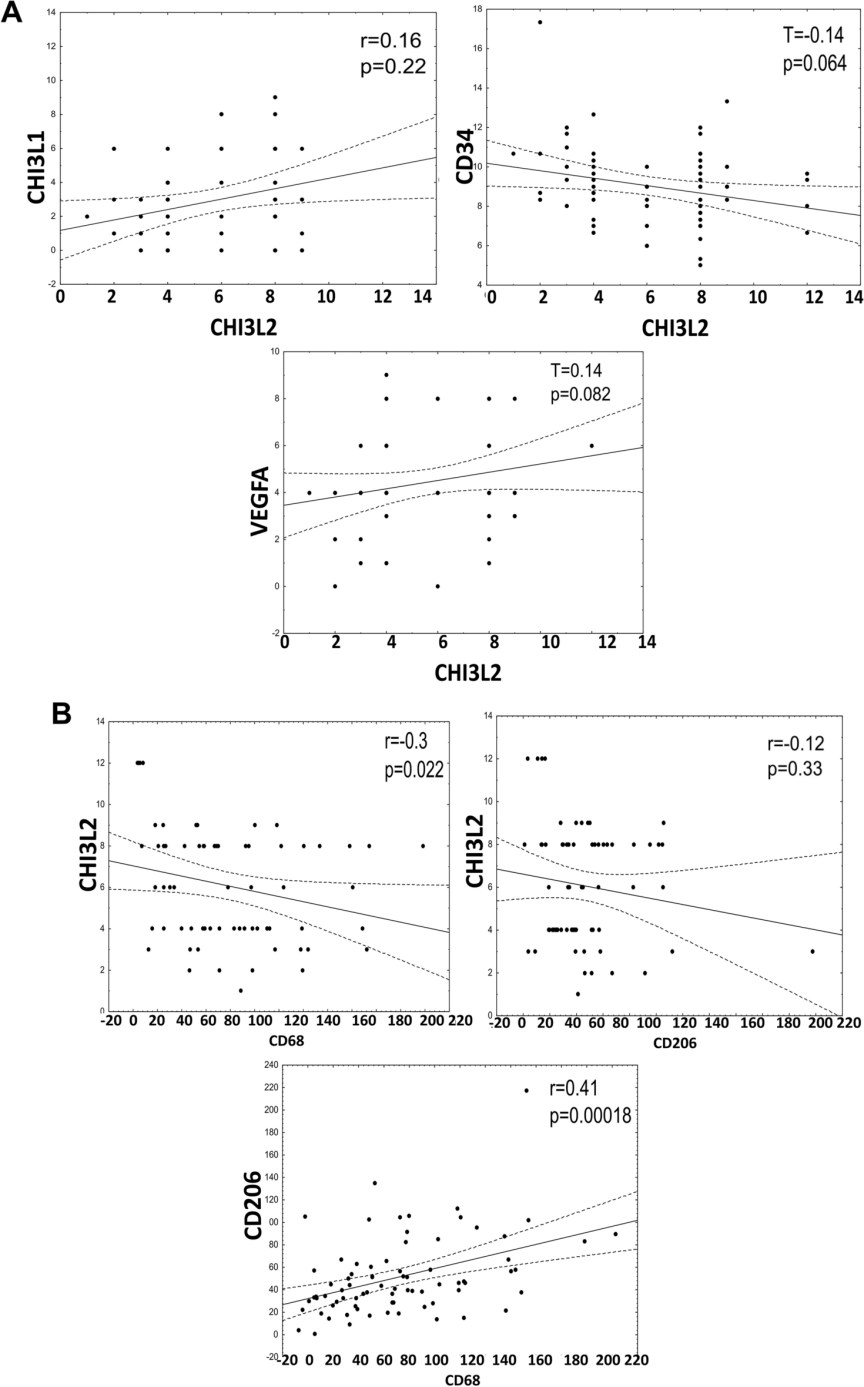
Analysis of immunohistochemical expression and correlations of CHI3L2 and A) angiogenic markers: CHI3L1 (Spearman correlation), CD34 and VEGFA (Kendall’s tau-b correlation); B) analysis of expression of CHI3L2, CD68 and CD206 (Spearman correlation); statistically significant p-values were < 0.05.

The angiogenesis markers VEGFA, VEGFC and VEGFD were expressed in the cytoplasm of the cancer cells. No significant Spearman correlations were found between CHI3L2 expression and angiogenesis markers, including CD31 (r = −0.094, *p* = 0.42), VEGFC (r = 0.02, *p* = 0.9) and VEGFD (r = −0.12, *p* = 0.98) (data not shown). CHI3L2 showed an almost statistically significant correlation with CD34 (T = 0.14, *p* = 0.064) and VEGFA (T = 0.14, *p* = 0.082), while there were no correlations with CHI3L1 (Fig. 3A), as shown by the Kendall’s Tau-b correlations. Spearman correlation showed a significant negative association between CHI3L2 and CD68 expression (Fig. 3B), but no significant correlation was found between CHI3L2 and CD206 expression. The expression of CD68 and CD206 showed a significant moderate positive correlation (Fig. 3B).

**Fig. 4:**
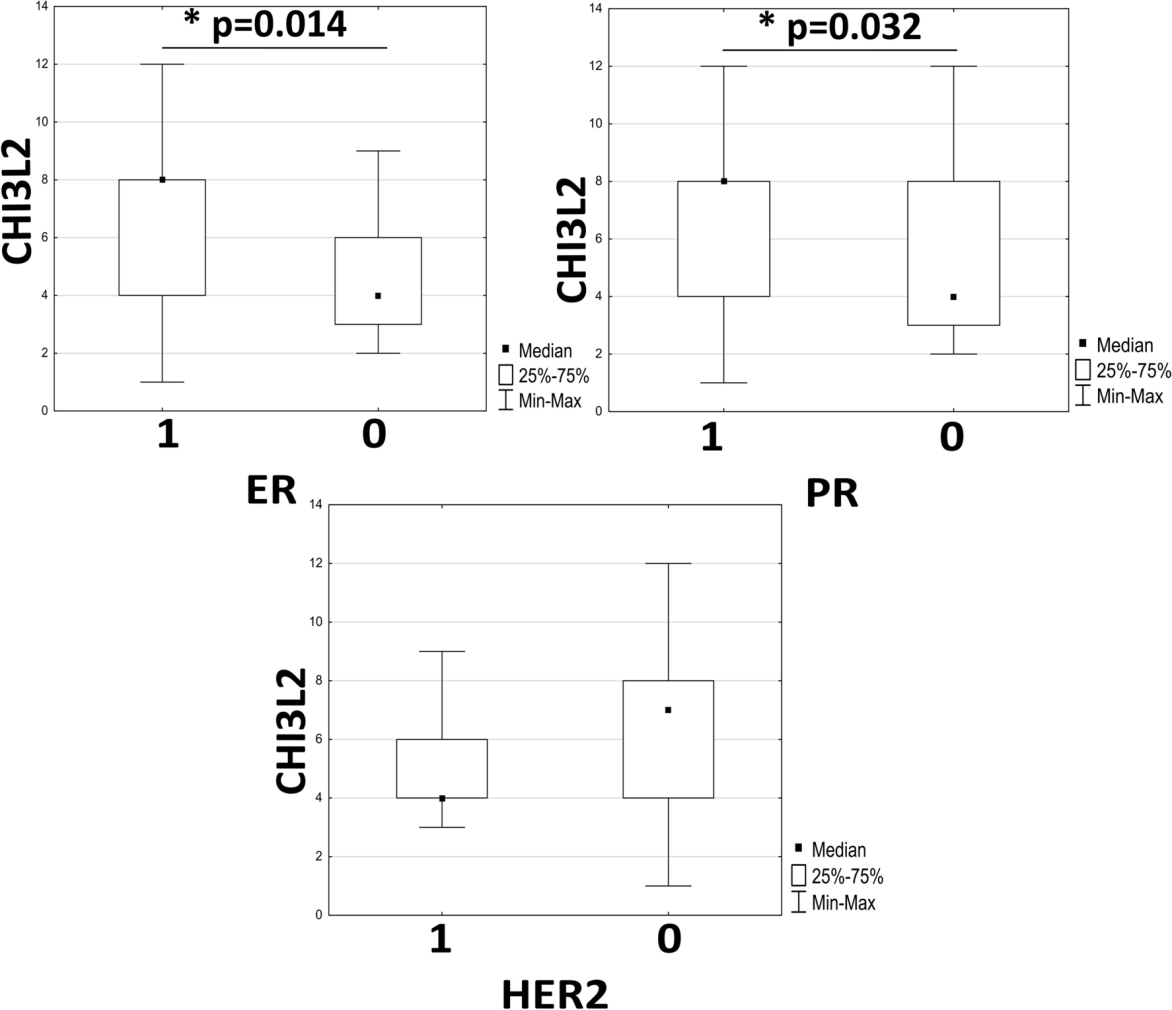
Analysis of immunohistochemical expression and its correlation with estrogen receptor (ER; ER^+^ N = 45, ER^-^ N = 21), progesterone receptor (PR, PR^+^ N = 40, ER^-^ N = 26) and human epidermal growth factor receptor 2 (HER2, HER2^+^ N = 40, HER2^-^ N = 26) status was performed using the Mann–Whitney U test. Expression was expressed as 0 for negative and 1 for positive receptors. Statistically significant p-values were < 0.05.

The cases with the highest CHI3L2 expression showed positive ER and PR expression, which was significantly correlated, while the lowest CHI3L2 expression was associated with positive HER2 expression (Fig. 4).

**Fig. 5:**
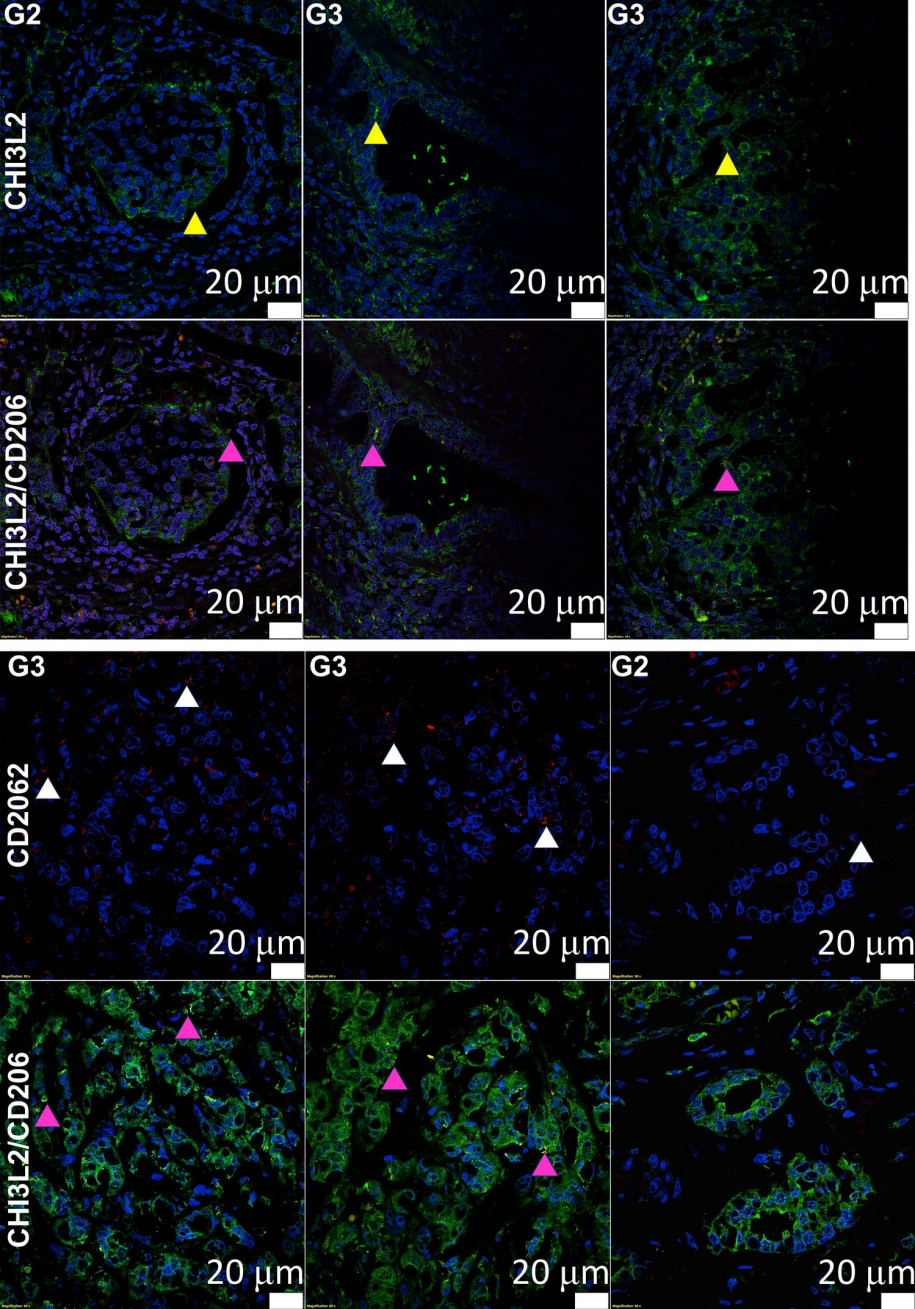
Expression of CHI3L2 in IDC tumors. Immunofluorescence reactions visualized by confocal microscopy showed the expression of CHI3L2 (green; yellow arows) in cancer cells and CD206 (red; white arroheads) as a characteristic feature of TAMs. Co-expression of CHI3L2 and CD206 was also observed (magenta arrows). The nucleus was stained with DAPI (blue). Images from single fluorescent channels is showed in Fig. 1S supplemental material.

The immunofluorescence reaction proves the presence of CHI3L2 in the cytoplasm of cancer cells in IDC tumors. In addition, CHI3L2 expression was also observed in CD206-positive TAMs (Fig. 5).

### CHI3L2 is not a prognostic factor for patient survival

The analysis of patient survival using the proportional Cox hazards model showed no influence of CHI3L2 expression on the survival of patients with IDC (*p* = 0.52, Cox parameter = 0.11). However, an association was found with pT status (tumor size) and PR status (p = 0.011, Cox parameter = −1.5 and *p* = 0.0098, Cox parameter = 1.2, respectively) (Table 5). Other investigated clinicopathologic factors, such as ER, HER2 status, pN, Ki-76, age at treatment initiation, menopausal status and G, showed no significant associations. However, overall patient survival was found to be significantly influenced by pT, which had an unfavorable prognostic value, and PR, which had a favorable prognostic value. Table 5 shows the results of the Cox proportional hazard analysis.

**Table 5:** The results of the proportional Cox hazard analysis of the clinicopathologic patient data showed statistically significant *p*-values at a level of < 0.05 (indicated by bold).

Factor	Level effect	Parameter	*p*-value
Age		−0.03	0.65
Premenopausal Status	1	−0.84	0.12
ER		−0.51	0.29
PR		**1.19**	**0.01**
HER2		0.73	0.06
Triple negative	TN	1.47	0.05
Tumor size (pT)	2	**−1.48**	**0.01**
Lymph node (pN)		0.14	0.79
Grade (G)	2	−0.77	0.16
Grade (G)	3	−0.13	0.81
Ki-67		0.04	0.09
CHI3L2		0.11	0.52

ER: estrogen receptor; PR: progesteron receptor; HER2: human epidermal growth factor receptor 2; TN: triple negative (no expression of ER, PR and HER2 receptor).

### Expression of CHI3L2 positively correlates with the phosphorylation of ERK1/2 and STAT-3 in IDC tumors

**Fig. 6:**
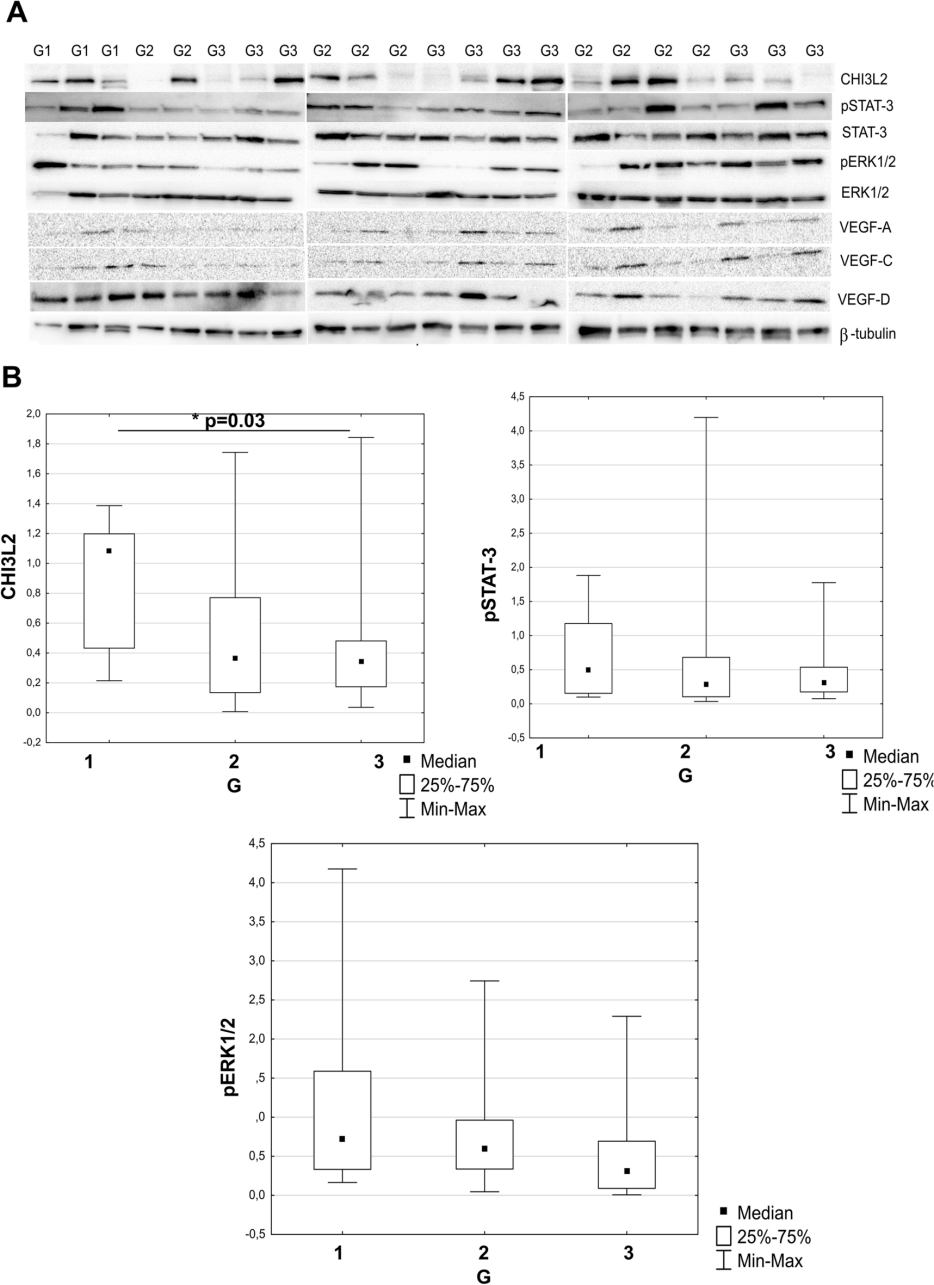
Western blot analysis of CHI3L2 expression in IDC in** A** tumors of different malignancy grade: G1 (n = 3), G2 (n = 9) and G3 (n = 10), with expressions of pSTAT-3, STAT-3, pERK1/2 and ERK1/2;** B** statistical analysis (data from densitometric analysis of Western blots experiments provided in triplicates) on CHI3L2, pSTAT-3 and pERK1/2 expressions in G1-G3 grade tumors (Kruskal–Wallis test, post-hoc Dunn’s test). Raw data from densitrometric analysis is showed in Fig. 2S supplemental material.

**Fig. 7:**
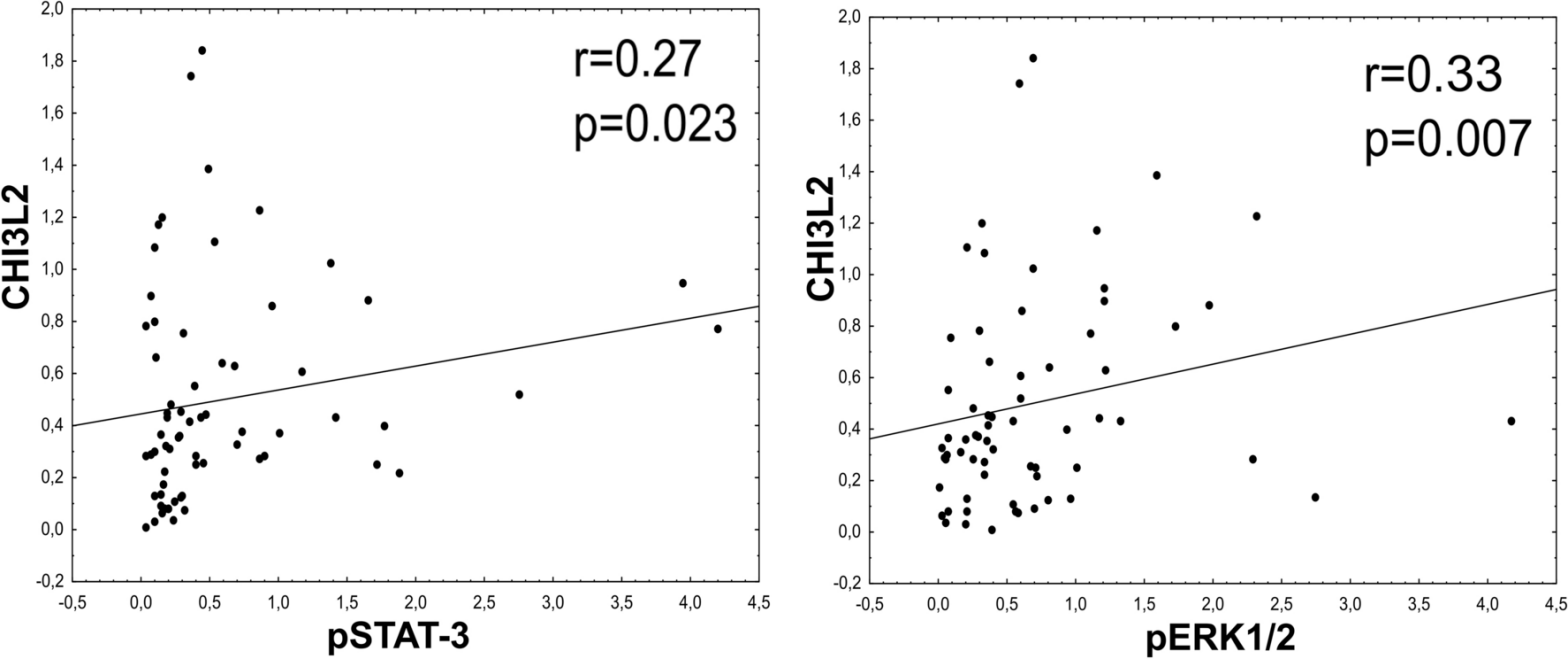
Spearman’s correlations of CHI3L2 and pSTAT-3 and pERK1/2 expressions from Western blot analysis; statistically significant p-values were < 0.05. Western blots were performed in triplicate.

In tumor material, the highest expression of CHI3L2 was observed in G1 IDC tumors, and it decreased with the progression of tumor grades (see Fig. 6A, B). Immunoblotting also showed negative cases for CHI3L2 expression. No correlation was found between CHI3L2 expression and CHI3L1, VEGFA, VEGFC and VEGFD (data not shown). The highest level of CHI3L2 showed a positive and significant correlation with the phosphorylation of STAT-3 and ERK1/2 (Fig. 7).

### Breast *cancer* cells produce CHI3L2 and may stimulate macrophages to express CHI3L2

**Fig. 8:**
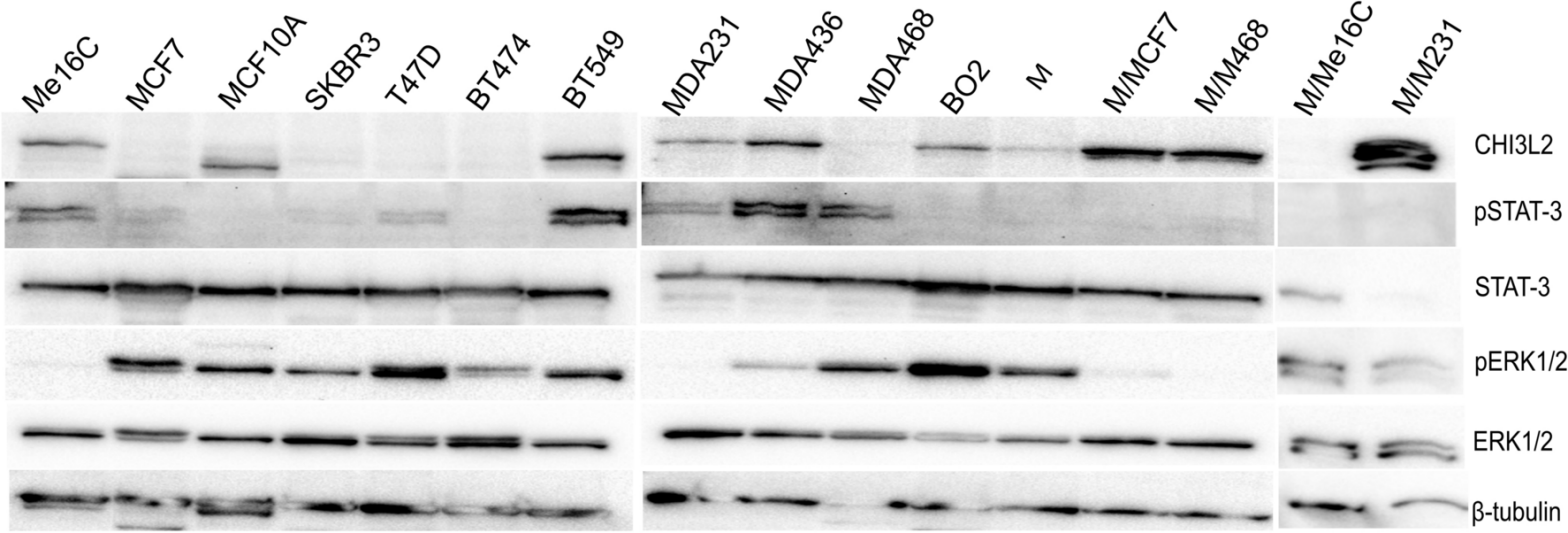
Western blot analysis of CHI3L2, pSTAT-3, STAT-3, pERK1/2 and ERK1/2 in breast cancer cell lines, macrophages (M) and macrophages in co-culture with MCF-7 (M/MCF7), MDA-MB-468 (M/M468), Me16C cells (M/Me16C) and MDA-MB-231 (M/M231) cells (the characteristics of breast cancer cell lines are shown in Table 1).

Expression of CHI3L2 protein was observed in breast cancer cells BT-549, MDA-MB-436 and BO2, as well as in normal breast epithelium and cells with fibrocystic disease: HME1-hTERT (Me16C) and MCF10A (Fig. 8). All analyzed cells expressing CHI3L2 were negative for ER, PR and HER2 and are considered representative of the basal subtype of breast cancer, except for the BO2 cells with metastatic adenocarcinoma features, as shown in Table 1. Macrophages expressed CHI3L2 at low levels, but the expression of this protein was higher in macrophage cocultures with conditioned medium from CHI3L2-negative cancer cells (MCF-7 and MDA-MB-468), and also with CHI3L2-positive MDA-MB-231 cells. Interestingly, macropgahes co-culture with CHI3L2-positive normal epithelial cells (HME1-hTERT (Me16C) didn’t provide to CHI3L2 expresion in macrophages. In addition, the levels of pSTAT-3 and pERK1/2 in breast cancer cells and macrophages were not dependent on CHI3L2 expression (Fig. 8).

### Changes in CHI3L2 levels affect ERK1/2 and STAT-3 phosphorylation

**Fig. 9:**
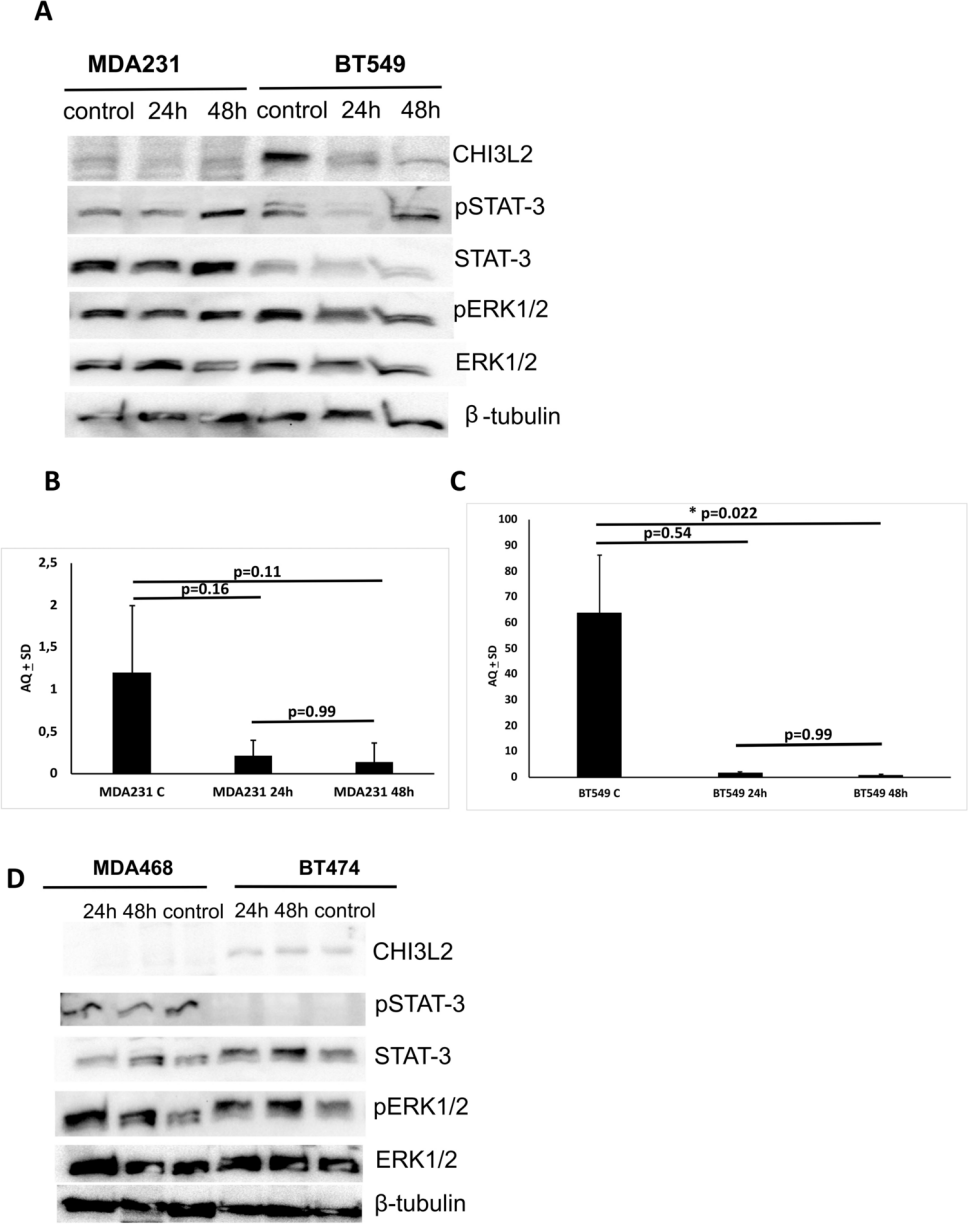
Breast cancer cells models with different status of CHI3L2 after transfection with CHI3L2 siRNA: (**A**) changing in STAT-3 and ERK1/2 phosphorylation; Western blot analysis; (**B**) analysis of CHI3L2 mRNA in MDA-MB-321 and (**C**) BT-549 cells during trnasfection experiments provided in triplicates in ddPCR analysis; and (**D**) with presence of recombinant CHI3L2 protein in MDA-MB-468 and BT474 cells ; Western blot analysis; Kruskal–Wallis test followed by Dunn test

Transfection experiments with CHI3L2 siRNA resulted in a decrease of pERK1/2 in BT-549 cells at 24h and 48h, but no difference was observed in MDA-MB-231 cells. In the case of STAT-3 phosphorylation, the lowest expression levels were detected in BT-549 cells only at 24h post-transfection, whereas an increase was observed at 48h in MDA-MB-231 cells (Fig. 9A). The analysis of transfection with CHI3L2 mRNA levels in MDA-MB-231 and BT-549 cells was confirmed by ddPCR technique (Fig. 9B, C). Consistent with these findings, in experiments where CHI3L2 recombinant protein was added to MDA-MB-468 and BT-474 cell culture media (CHI3L2 status is negative in these two cell lines), an increase in pERK1/2 levels was observed at 24 and 48h compared to control cultures (Fig. 9D). No changes were observed in pSTAT-3 levels.

### CHI3L2 mRNA expression was not significantly different in non-specific breast cancers

**Fig. 10:**
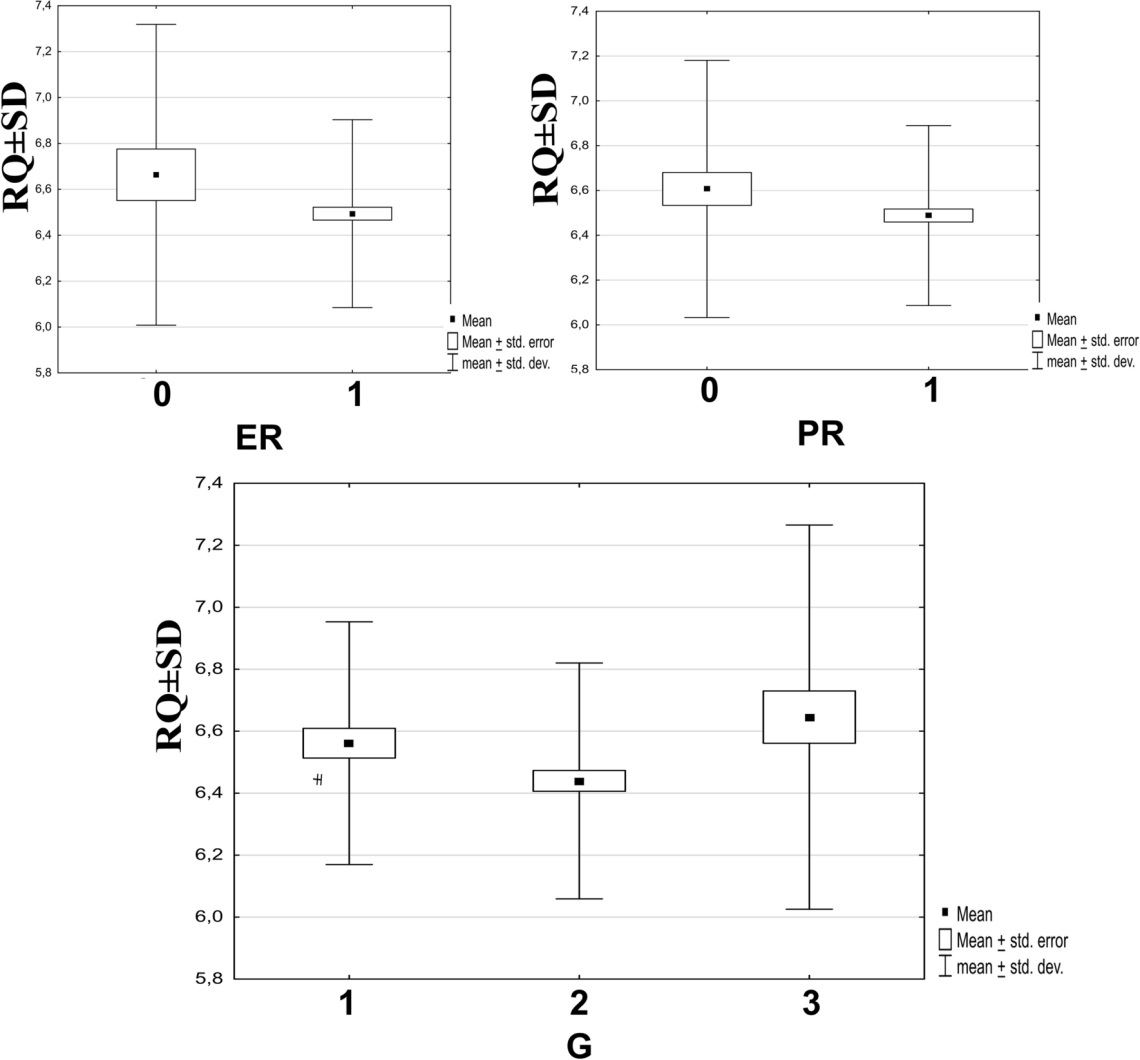
PCR analysis of CHI3L2 mRNA expression level in breast tumors. Different tumor grades: G1 (n = 67), G2 (n = 168), G3 (n = 54); G1 vs G2 p = 0.083; G2vsG3 p = 0.12, G1vs G3 p = 0.9 (Kruskal–Wallis test, post-hoc Dunn’s test); status of estrogen (ER), progesterone (PR) and HER2 receptor; 0: negative expression, 1: positive expression of the receptors; CHI3L2 vs PR p = 0.44, CHI3L2 vs ER p = 0.46 (Mann–Whitney U-test); statistically significant p-values were < 0.05.

Transcriptome analysis performed in breast cancer cases revealed that mRNA levels of CHI3L2 were highest in G3, although there were no significant differences between tumors with different tumor grades. A tendency for the highest CHI3L2 mRNA level was observed in tumors without ER or PR expression. This correlation was not significant, but a trend was observed (Fig. 10).

## Discussion

CHI3L1 and CHI3L2 proteins are both chitinase-like proteins with lack of enzymatic activity. Some proteins in this family have chitinase activity: chitotriosidase and acidic mammalian chitinase (AMCase). In recent years, the role of this chitinases in cancerogenesis has been discussed. CHI3L1 is one of the best known chitinase proteins and is currently known to have a pro-tumor and pro-angiogenic effect in many types of cancer, including breast cancer. On the other hand, little is known about CHI3L2, structural similarity with CHI3L1 in size, nucleotide and amino acid sequences is 47% and 51% homology, respectively, without cross-reactivity^43^. In addition, these proteins share the same NH2-terminal amino acid residues as noted in their alternative names (YKL-40 and YKL-39). Some authors have pointed out that simultaneous production of CHI3L1 and CHI3L2 does not occur and that these proteins have different biological functions, also in ERK patchway phosphorylation^44^.

In fact, only few papers have reported the expression of CHI3L2 in breast cancer, suggesting a possible role of this protein in carcinogenesis. Previous studies have demonstrated the expression of CHI3L2 in cancer cells, including glioblastoma tumors and cell lines such as U-87 MG and HEK293^44^,^45^.

Indeed, CHI3L2 was expressed in glioblastoma tumors in cancer cells and microglia, and its expression was associated with poor patient prognosis^24^,^45^. The presence of this protein in macrophages infiltrating nonspecific breast cancer tumors and its highest levels in developing metastases were reported by Liu and colleagues^24^. In addition, the authors show that the highest concentration of CHI3L2 after neoadjuvant therapy is an unfavorable indicator of treatment efficacy and also stimulates angiogenesis^24^.

In contrast, our team’s results indicate that CHI3L2 is expressed in breast cancer cells in in vitro cultures as well as in cancer cells in tumor material from patients with IDC. In our study, we found evidence that CHI3L2 expression in cancer cells is specific for this type of breast tumor.

In our studies performed on commercially available cancer cell lines, the expression of CHI3L2 was detected in triple negative cells lacking ER, PR and HER2, indicating a basal B breast cancer subtype. Our screening conducted on ten breast cancer cell lines showed that cells characterised as invasive ductal breast carcinoma basal subtype may expressed CHI3L2 protein, like BT-549 cells, and also may be CHI3L2 negative, like luminal BT-474 cells. Moreover, basal B subtype breast cancer cells, like MDA-MB-231, BO2, and MCF10A, which represent different breast cancer tumor type (metastatic adenocarcinoma, and fibrocystic disease), were also CHI3L2^+^. This finding could have clinical implications, and indicate, that CHI3L2 expression may be characteristic for basal B breast cancer subtype. Moreover, our results obtained in functional in vitro model suggest, that CHI3L2 is involved in IDC basal B breast cancer cells, represent in our studies by BT-549 cells, in modulation of ERK1/2 and STAT-3 phosphorylation patchways. Hovewer this findings may be general, due to broad spectrum of proces uder regulation of this signalling patchways, may have far reaching implication in our deeper understanding of IDC biology and therefore may open new areopag for precision medicine. It is worth noting that patients with triple-negative breast cancer have still a poorer prognosis, especcially triple-negative status, from all breast cancer diagnosis.

In contrast to breast cancer cells in in vitro culture, the expression of CHI3L2 in tumors was highest in tumors positive for estrogen receptor (ER) and progesterone receptor (PR) and lowest in HER2 positive tumors.

However, the results from the monolayer in vitro model of cancer cells may not reflect the complex system that occurs in tumors, where interactions between cancer cells, macrophages and stromal cells play a role and dynamics of this interaction are changing during tumor development. This difference in the expression pattern of CHI3L2 between in vitro models and patient material could be due to the above-mentioned interaction.

Although the differences in CHI3L2 mRNA levels were insignificant, this analysis was performed in breast cancer tumors without distinguishing between cancer subtype diagnoses. Analysis of clinicopathologic data showed that CHI3L2 expression had no effect on patient survival or event-free survival. Our analysis of tumor material also showed a decrease in CHI3L2 expression with higher tumor grades (G). It is possible that the overexpression of CHI3L2 in low-grade malignant tumors suggests that the protein may be involved in inhibiting tumor growth or promoting tumor growth only at early stages. Our results are different from those of previous studies, such as Liu et al. and Kzhyshkowska^3,24^,which suggest that CHI3L2 is rather an unfavorable prognostic factor in cancer cells. In our opinion, these discrepancies could be related to different forms of breast cancer, which might differ in terms of CHI3L2 expression. Therefore, it is worth investigating the role of CHI3L2 protein in different types of breast cancer.

Our hypothesis about the positive role of CHI3L2 in patient prognosis is noteworthy, especially considering that CHI3L1 plays a significant role as a poor prognostic factor in various cancers such as glioma and breast cancer^45^. Our previous study in IDC tumors^19^ shows that CHI3L1 supports VEGFD-mediated angiogenesis in this type of cancer^46^. Hovewer in these studies, no significant correlations were found between CHI3L2 and angiogenesis markers even with CHI3L1, suggesting that CHI3L2 is not involved in angiogenesis in IDC and this two similar protein: CHI3L1 and CHI3L2 appear to differ significantly in their function.

Activation of the ERK1/2 kinase pathway, a component of the mitogen-activated protein kinase pathway (MAPK), usually promotes tumor growth, metastasis and angiogenesis. High concentrations of pERK1/2 are observed in various types of cancer such as breast, colon and lung cancer. The combination of RAF-ERK inhibitors may have anti-cancer effects^47,48^. On the other hand, the anti-apoptotic function of pERK1/2 has also been documented in tumor suppressive mechanisms^47^. Certain anticancer drugs such as cisplatin, etoposide or paclitaxel and other sources of damage such as γ-irradiation, UV radiation and natural plant substances can trigger the anti-apoptotic function of pERK1/2.

From this perspective, CHI3L2 could serve as a promising subject to investigate its influence on ERK pathway activation in IDC^47,49,50^. Mechanistically, CHI3L2 has been found to activate ERK1/2^45^,^44^. Consistent with these reports, our results also suggest that CHI3L2 increases pERK1/2 and pSTAT-3 in IDC tissue. Interestingly, opposite roles of pERK1/2 are possible and have been described by Areshkov and team. For example, CHI3L1 leads to a proliferation of cancer cells, while CHI3L2 appears to inhibit the cell cycle^44^. Reults obtained in our transfection studies showed, that decreasing CHI3L2 mRNA lead to decreasing phosphorylation of ERK1/2 in invasive ductal breast cancer cells BT-549, but no effect was in metastatic adenocarcinoma cells MDA-MB-231. Moreover, addition of exogenous CHI3L2 protein incresed level of pERK1/2 in CHI3L2^-^ metastatic adenocarcinoma MDA-MB-436 cells, and also in invasive ductal breast carcinoma CHI3L2^-^ BT-474 cells. This results are consistence, and suport our hypothesis on specific role of CHI3L2 protein in ERK1/2 phosphorylation, depending of breast cancer subtype, and desribed new role of CHI3L2 protein in invasive ductal breast carcinoma. Consisted with our findings from in vitro studies, we showed for the first time positive and signifficant correlation of CHI3L2 expression and phosphorylation of ERK1/2 in IDC tumors. Hovewer, the exact role of CHI3L2-mediated activation of the ERK1/2 signaling pathway remains to be investigated more deeply, and further studies are neddedd to fully understand mechanisms regulated by CHI3L2 in invasive ductal breast carcinoma, also in context of molecular differentated another subtypes of breast cancer tumors.

Another signal transducer and activator of transcription, STAT-3, in turn plays a multifaceted role in carcinogenesis. The promotion of migration, invasion, metastasis, angiogenesis and immune escape of tumors characterizes the tumor-promoting effect of STAT-3 phosphorylation. Conversely, the antitumor effect of pSTAT3 is mediated in part by the reduced efficacy of IL-8 observed in glioblastomas^51^,^52^. In breast cancer, activation of pSTAT-3 could contribute to cancer cell survival. In the review publication, written by Tolomeo and Cascio^52^, the importance of STAT-3 activation for the mechanisms of carcinogenesis is analyzed in detail. Currently, the investigation of the role of CHI3L2 in the effects of pSTAT-3 in IDC is an interesting question to be explored.

In our studies, transfected MDA-MB-231 with CHI3L2 siRNA cells showed increased level of pSTAT-3, contrary to BT-549; addition of recombinant CHI3L2 protein has no effect of pSTAT-3 level in MDA-MB-468 and BT-474. This result suggest, that CHI3L2 may has diferent role in STAT-3 activation, depending of breast cancer subtype, hovewer currently is not enough information in this area. Our results, obtained from analysis of IDC tumors showed positive, signiffcance correlation of CHI3L2 expression level and pSTAT-3, and this findings are in line with results obtained from functional i*n vitro* experiments with BT-549 cells, suggesting for the first time regulation role of STAT-3 activation in this type of breast cancer tumors.

In addition, we also observed the expression of CHI3L2 in TAMs, which is consistent with the results of another study on breast cancer and glioma tumors^45^. Moreover, CHI3L2 was expressed at low levels in macrophage cells in vitro, but its expression was enhanced in co-cultures designed to mimic the tumor microenvironment and TAMs. Interestingly, in our simple co-culture model, CHI3L2-negative cancer cells were found to stimulate macrophage production of this protein, whereas CHI3L2-positive normal breast epithalial cells not. These results showed plasticity of CHI3L2 expession in macrophages, and suggest that CHI3L2 may be involved in communication between cancer cells and immune cells, but further studies are needed to confirm this hypothesis. The specific function of CHI3L2 in the interaction between cancer cells and macrophages remains unclear at this moment. Our results help to clarify previous observations by Liu and colleagues, who detected CHI3L2 expression exclusively in TAMs but not in cancer cells^24^ and indicate, that this situation may also occured. Furthermore, we observed a negative correlation between CHI3L2 expression in tumor cells and infiltration of macrophages, but not exactly TAMs. It is also conceivable that CHI3L2 expression in breast cancer cells and macrophages could promote different pathways of tumor growth. Our team will focus on this question in the future to clarify the mechanism of the role of CHI3L2 in the communication between TAMs and cancer cells in breast tumors, which could be a new key factor in IDC tumor progression^53^. This interaction could be important for predicting breast cancer treatment and categorizing breast cancer types, however, it is crucial to substantiate this hypothesis.

## Conclusions

In this studies, we present for the first time the expression of CHI3L2 protein in cancer cells in IDC and also in breast cancer cells in in vitro culture, and also confirmed CHI3L2 expression possibility in tumor–assiociated macrophages. Previously, CHI3L2 expression in breast tumors had only been observed in TAMs. Higher expression of CHI3L2 was observed in tumors with lower malignancy grade or in non-malignant lesions and higher levels of CHI3L2 protein were detected in ER + and PR + tumors, which may indicate protective, anti-tumor role of this protein. Moreover, positive, significant correlation of CHI3L2 expression with pSTAT-3 and pERK1/2 level was detected in tumor material and also regulation of ERK1/2 and STAT-3 phosphorylation in in vitro studies by CHI3L2 protein was observed, suggesting role of CHI3L2 in this signalling pathways activation in IDC. Conducted research showed that CHI3L2 protein is not involved in angiogenesis and has a different function than CHI3L1. Additionally, provided in vitro experiments showed that CHI3L2-negative breast cancer cells can induce macrophages to produce this protein, which may suggest a potential role of CHI3L2 protein in IDC, possibly contributing to tumor-microenvironment cross-talk, as well as an antitumor protective function.

Taken together, this work presents for the first time a novel role of the CHI3L2 protein in IDC biology, which opens further direction of possibility for development of these findings in precision medicine areas.

### Study limitation

This study analyzed 74 tumor samples from patients IDC diagnosed with and followed their progress over time. A larger study with complete treatment data will validate these results and evaluate the significance of CHI3L2 protein as a predictor of IDC outcome.

## Supplementary Information

Supplementary Information.

## Data Availability

The datasets for this study can be found in the POLISH PLATFORM FOR MEDICAL RESEARCH 10.60956/j7rs-6p79; 10.60956/sase-tr74.
