# Correction: New insights into the role of the CHI3L2 protein in invasive ductal breast carcinoma

**DOI:** 10.1038/s41598-025-18786-1

**Published:** 2025-09-12

**Authors:** Agnieszka Rusak, Ewa Kątnik, Tomasz Górnicki, Christina Schmuttermaier, Krzysztof Kujawa, Aleksandra Piotrowska, Katarzyna Ratajczak-Wielgomas, Alicja Kmiecik, Andrzej Wojnar, Piotr Dzięgiel, Julia Kzhyshkowska

**Affiliations:** 1 Division of Histology and Embryology, Department of Human Morphology and Embryology, Wroclaw Medical University, T. Chalubinskiego St. 6a, 50-368 Wroclaw, Poland; 2 Institute of Transfusion Medicine and Immunology, Medical Faculty Mannheim, Heidelberg University, Ludolf-Krehl Street 13-17, 68167 Mannheim, Germany; 3 German Red Cross Blood Service Baden-Württemberg–Hessen, Ludolf-Krehl Street 13-17, 68167 Mannheim, Germany; 4 Statistical Analysis Centre, Wroclaw Medical University, K. Marcinkowskiego 2-6 St, 50-368 Wroclaw, Poland; 5 Department of Preclinical Sciences, Pharmacology and Diagnostics, Faculty of Medicine, Wroclaw University of Science and Technology, Hoene-Wronskiego 13 C St, 58-376 Wroclaw, Poland; 6 Department of Physiotherapy, University School of Physical Education, I. Paderewskiego 35 Al, 51-612 Wroclaw, Poland

Correction to: *Scientific Reports* 10.1038/s41598-024-77930-5, published online 18 November 2024

The original version of this Article contained an error in Table 1, where the column headings were displaced and some of the data under “tumor type” and “subtype” were incorrect.

In addition, the footnote of the table has been corrected.

The original Table 1 and accompanying legend appear below.

**Table 1:** Expression of CHI3L2 in breast cancer cell lines. The characteristics of the human breast cancer cell lines used in the study are described according to Kao et al. ^27^.

	Cell line CHI3L2	ER	PR	HER2	Tumor type	Subtype
HME1-hTERT (Me16C)	–	NA	–	NA	Basal B	+
MCF-7	+	+	–	Metastatic adenocarcinoma	Luminal	–
MCF10A	–	–	–	Fibrcoystic disease	Basal B	+
SK-BR-3	–	–	+	Adenocarcinoma	Luminal	–
T-47D	+	+	-	Invasive ductal breast carcinoma	Luminal	–
BT-474	+	+	+	Invasive ductal breast carcinoma	Luminal	–
BT-549	–	–	–	Invasive ductal breast carcinoma	Basal B	+
MDA-MB-231	–	–	–	Metastatic adenocarcinoma	Basal B	+
MDA-MB-436	–	–	–	Adenocarcinoma	Basal B	–
MDA-MB-468	–	–	–	Metastatic adenocarcinoma	Basal A	–
BO2	–	–	–	Metastatic adenocarcinoma	NA	+

ER: estrogen receptor, PR: progesterone receptor, HER2: human epidermal growth factor receptor 2.

The original Article has been corrected.

