# Supplementary material for: New insights into the role of the CHI3L2 protein in invasive ductal breast carcinoma
Source: Sci Rep. 2024 Nov 18;14:28529. doi: 10.1038/s41598-024-77930-5 (PMC11574116; doi:10.1038/s41598-024-77930-5)
Supplement: Supplementary file 1 — Supplementary Information. [file 41598_2024_77930_MOESM1_ESM.pdf]

Supplementary Information

(index on the right side)

Tumour material of invasive ductal breast carcinoma

CHI3L2: 40 kDa

Replication 1 | Replication 2 | Replication 3

G1 G1 G1 G2 G2 G3 G3 G3 | G1 G1 G1 G2 G2 G3 G3 G3 | G1 G1 G1 G2 G2 G3 G3 G3

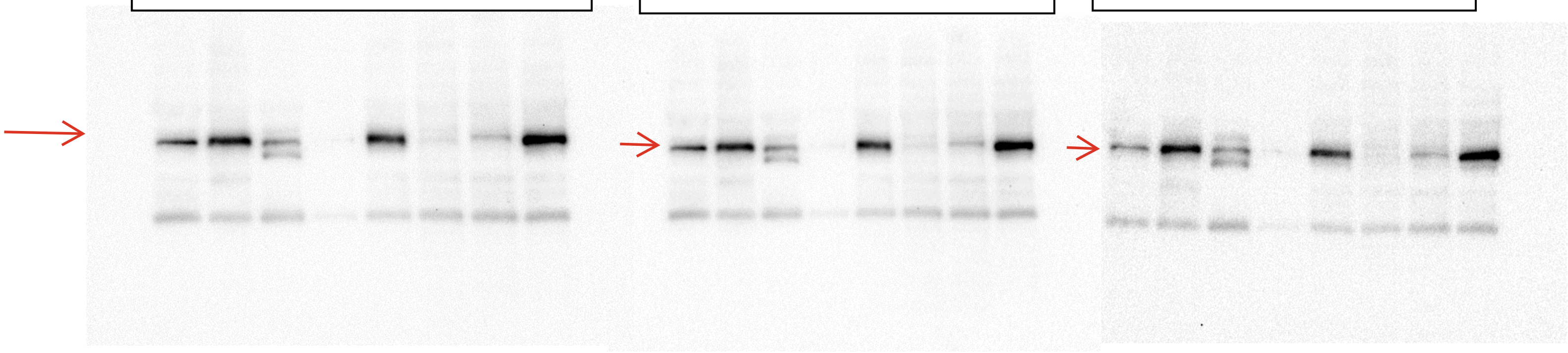

100 kDa
75 kDa
50 kDa
37 kDa
25 kDa
20 kDa
15 kDa

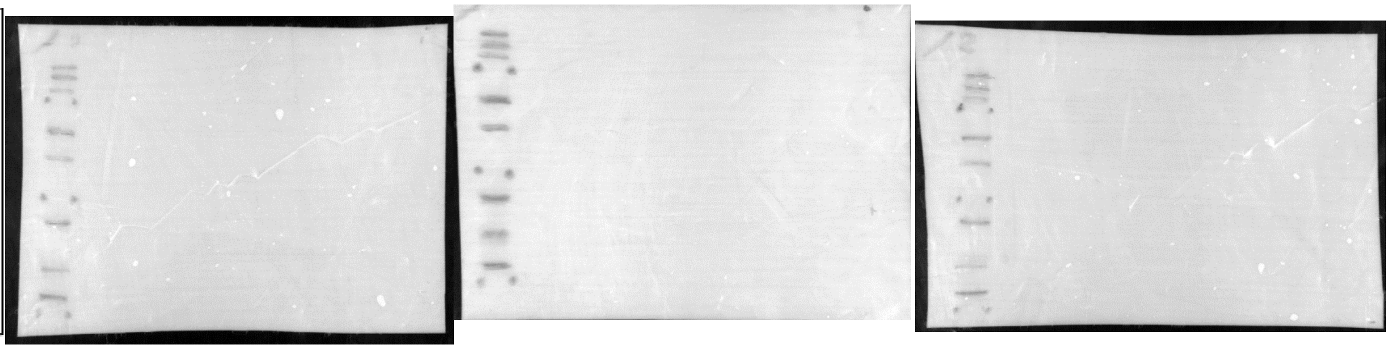

G2 G2 G2 G3 G3 G3 G3 | G2 G2 G2 G3 G3 G3 G3 | G2 G2 G2 G3 G3 G3 G3

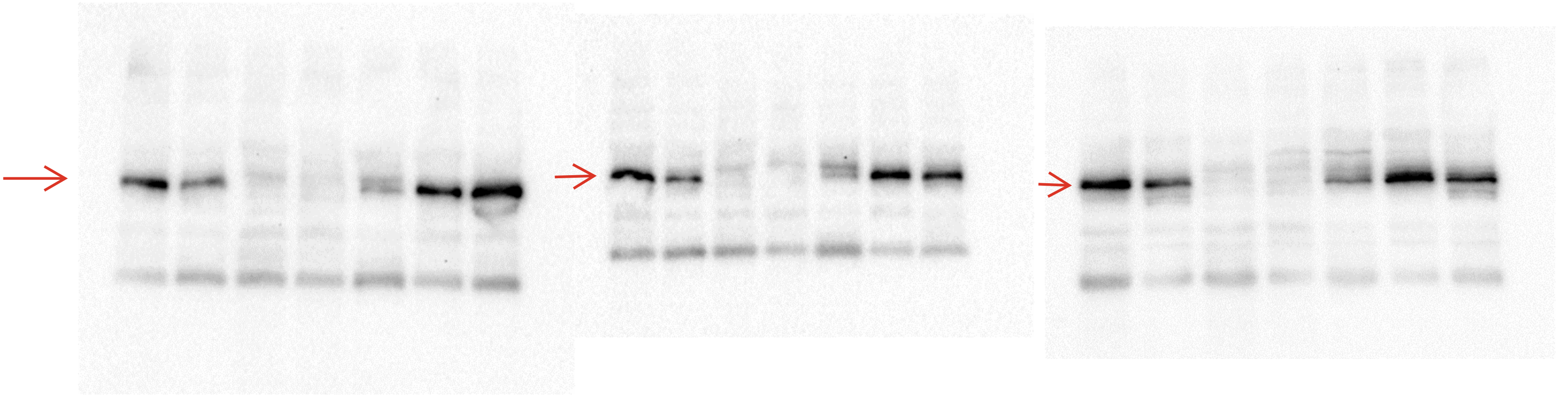

100 kDa
75 kDa
50 kDa
37 kDa
25 kDa
20 kDa
15 kDa

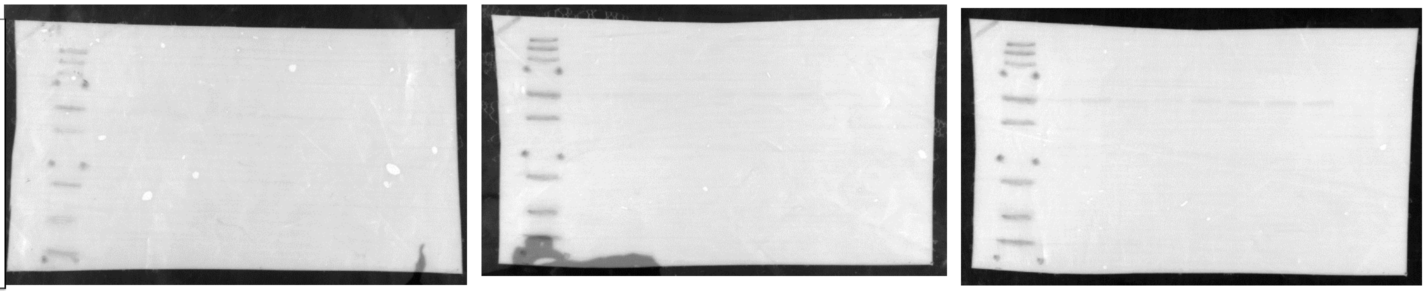

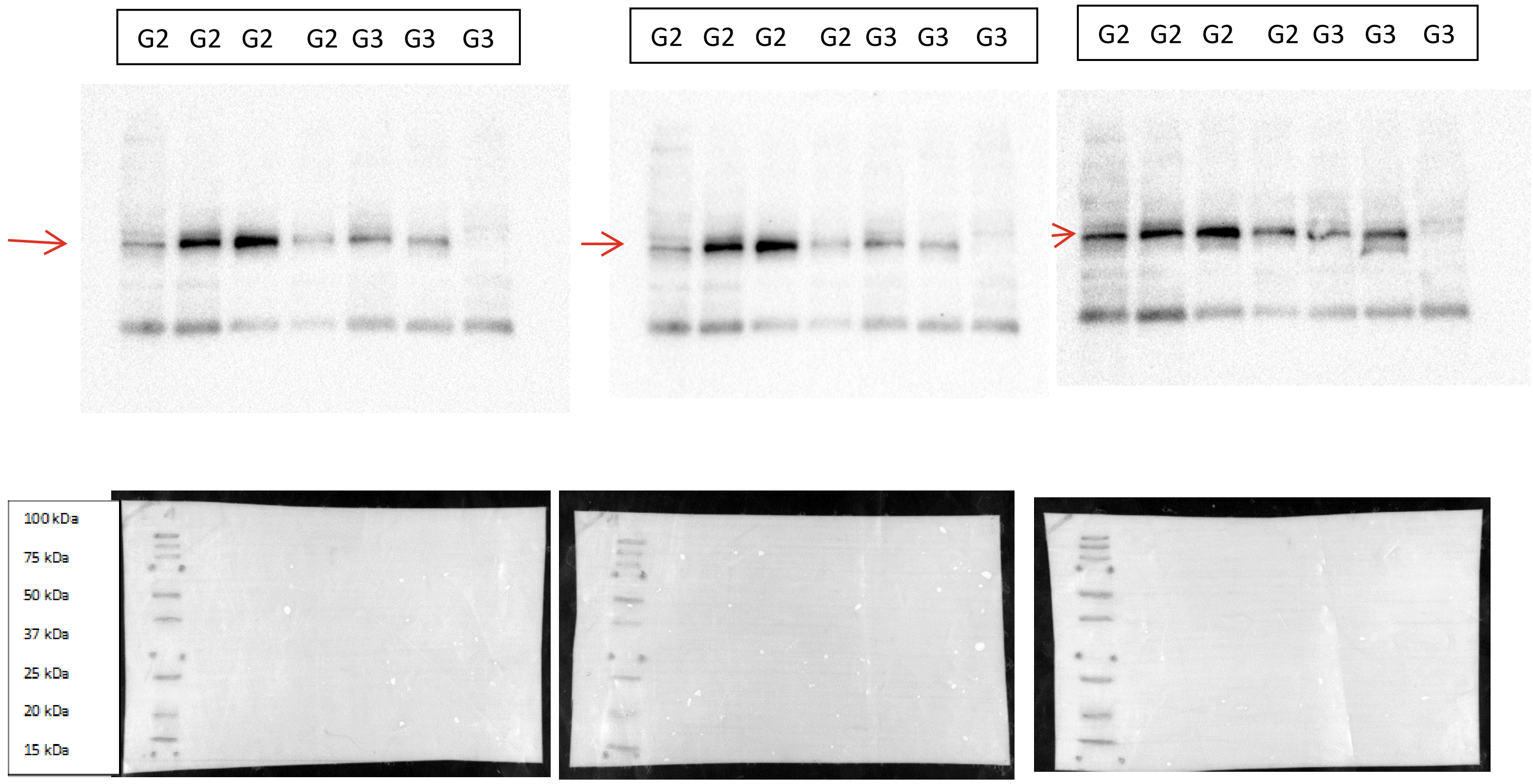

β-tubulin: 50 kDa

Replication 1

Replication 2

Replication 3

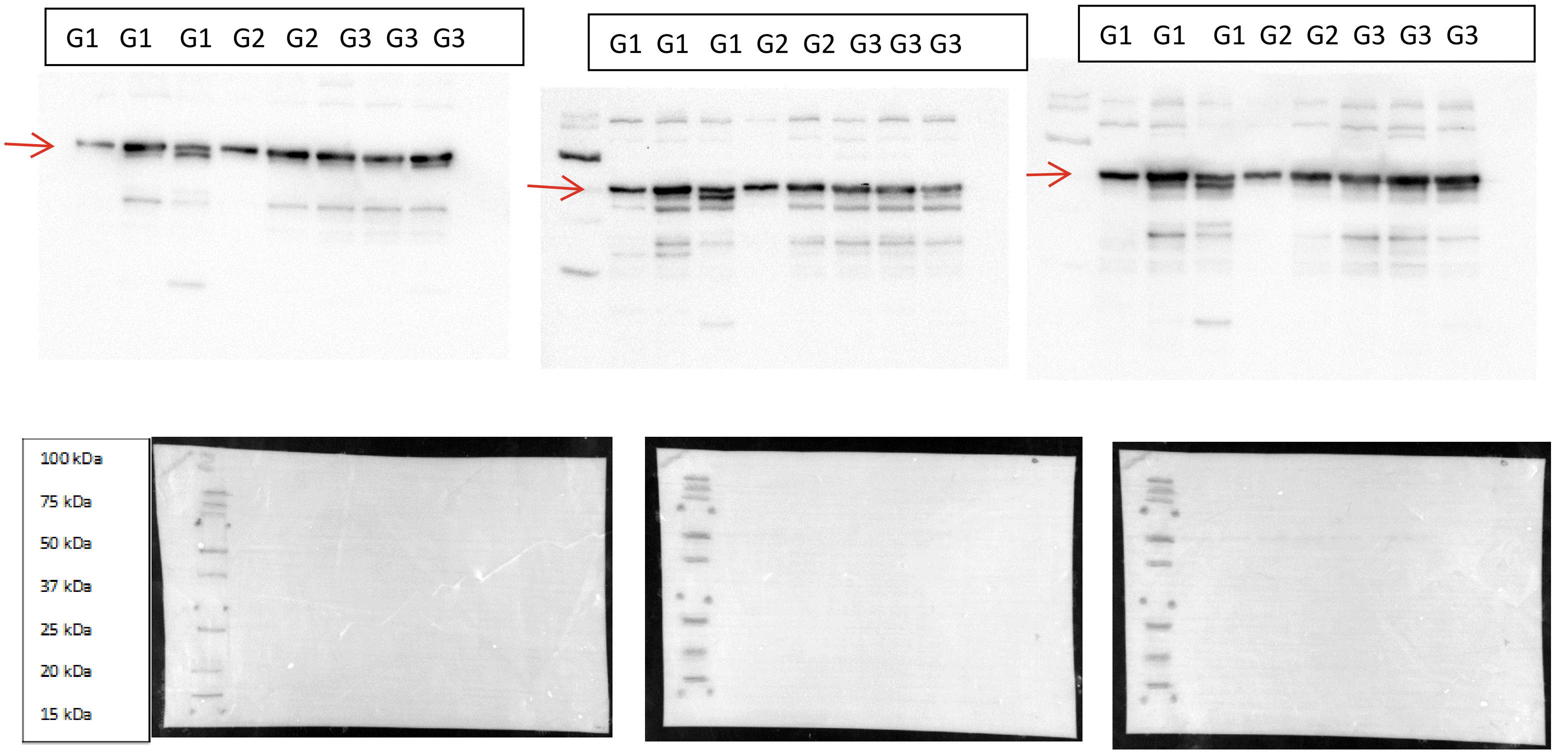

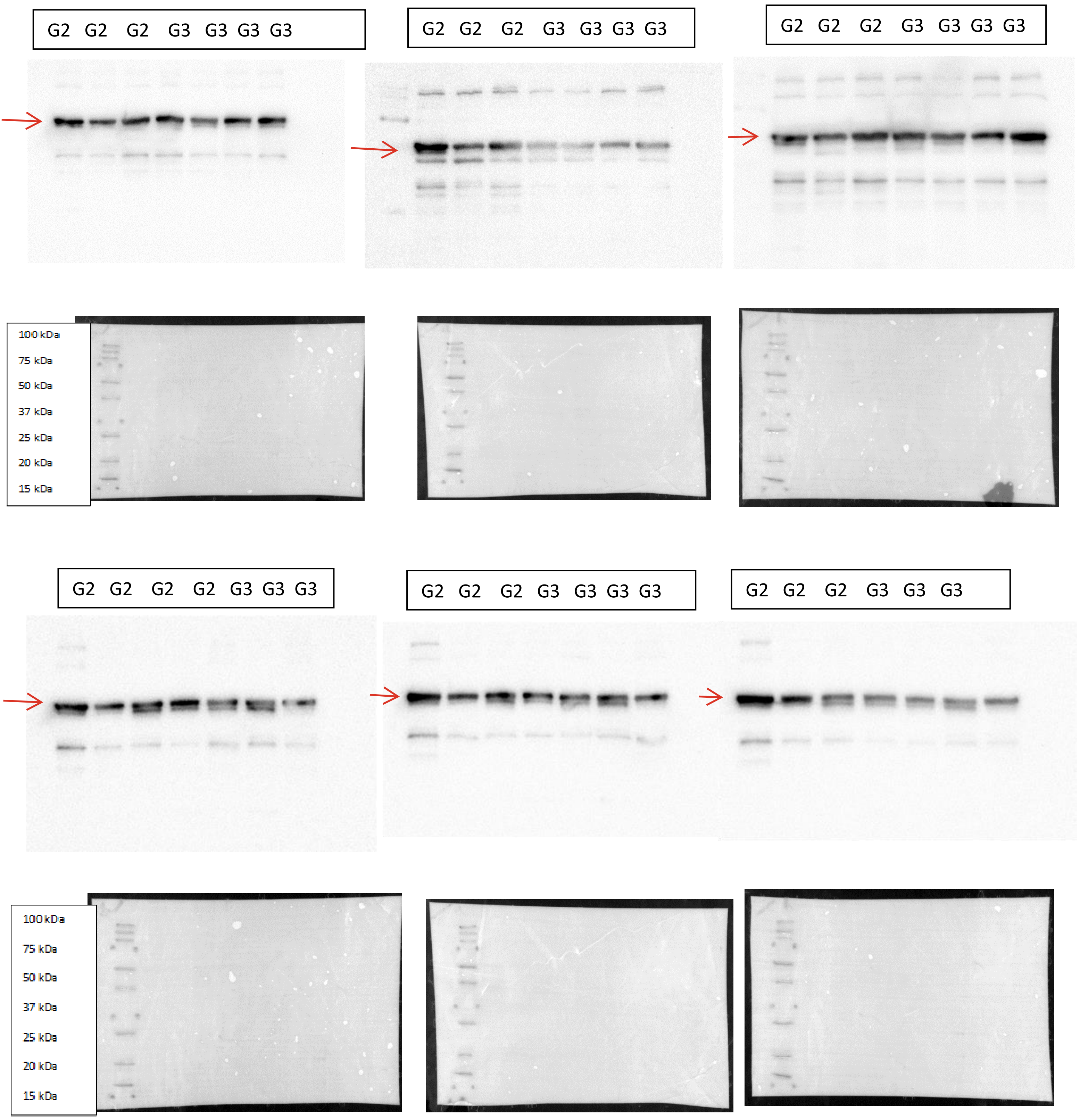
G2 G2 G2 G3 G3 G3 G3
G2 G2 G2 G3 G3 G3 G3
G2 G2 G2 G3 G3 G3 G3
100 kDa
75 kDa
50 kDa
37 kDa
25 kDa
20 kDa
15 kDa
G2 G2 G2 G2 G3 G3 G3
G2 G2 G2 G3 G3 G3 G3
G2 G2 G2 G3 G3 G3
100 kDa
75 kDa
50 kDa
37 kDa
25 kDa
20 kDa
15 kDa

pSTAT-3: 88kDa

Replication 1 Replication 2 Replication 3

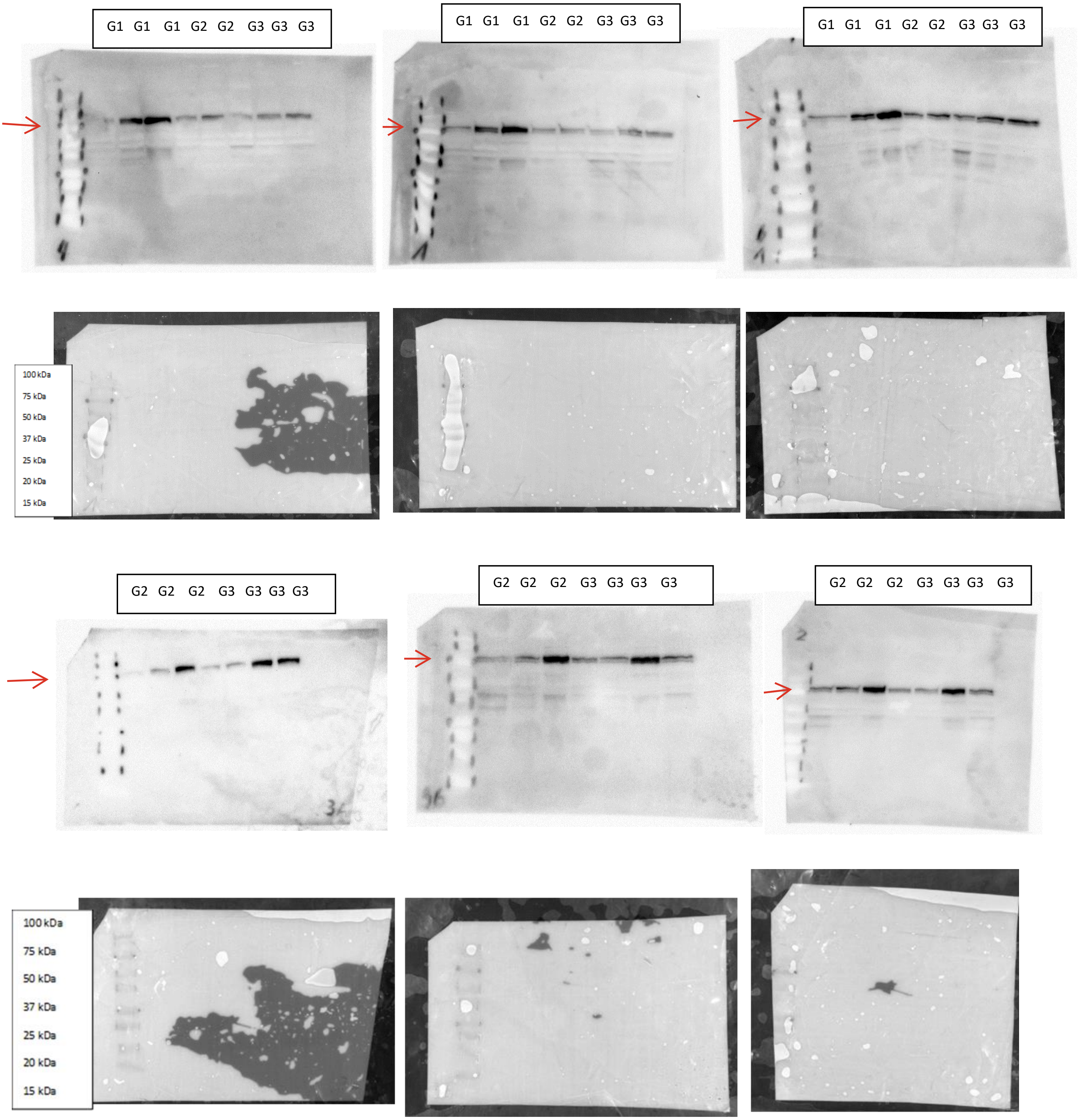

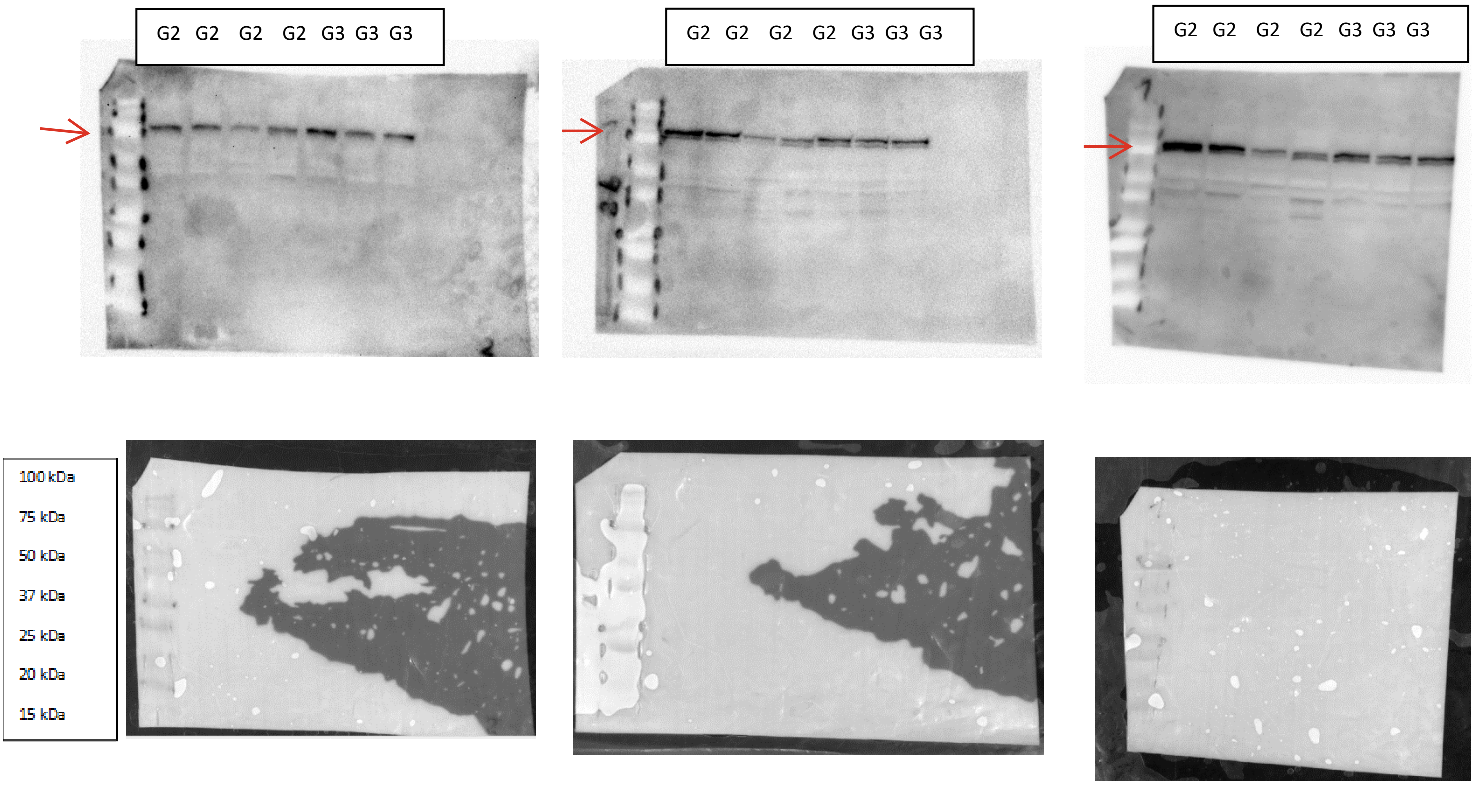
G2 G2 G2 G2 G3 G3 G3
G2 G2 G2 G2 G3 G3 G3
G2 G2 G2 G2 G3 G3 G3
100 kDa
75 kDa
50 kDa
37 kDa
25 kDa
20 kDa
15 kDa

STAT-3: 92 kDa

Replication 1

Replication 2

Replication 3

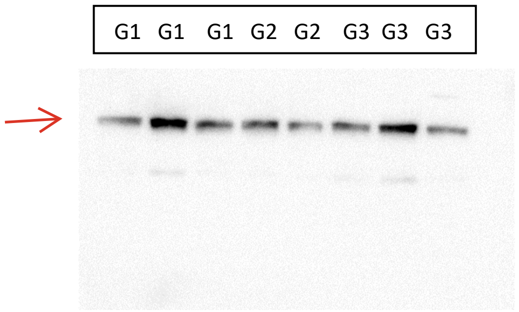

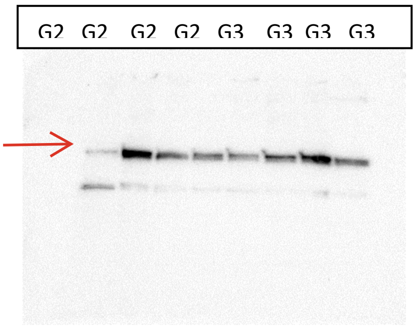

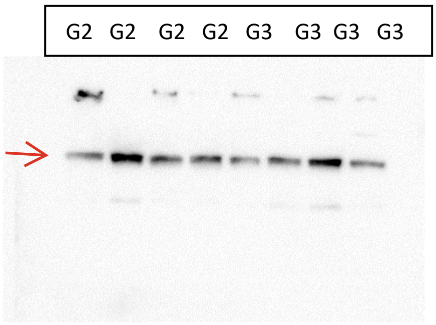

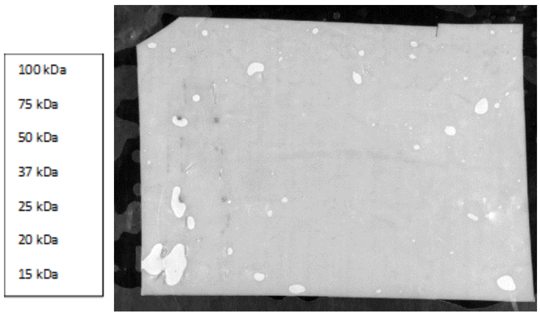

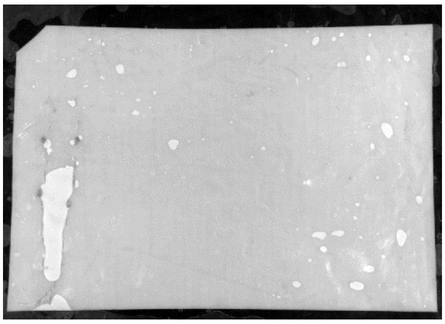

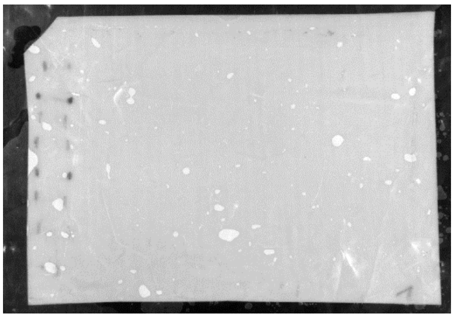

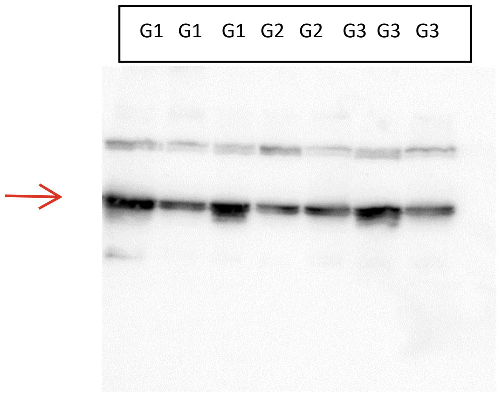

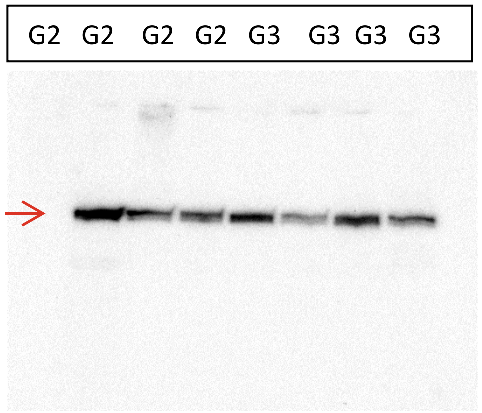

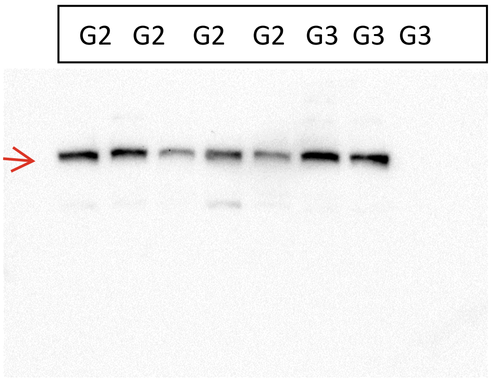

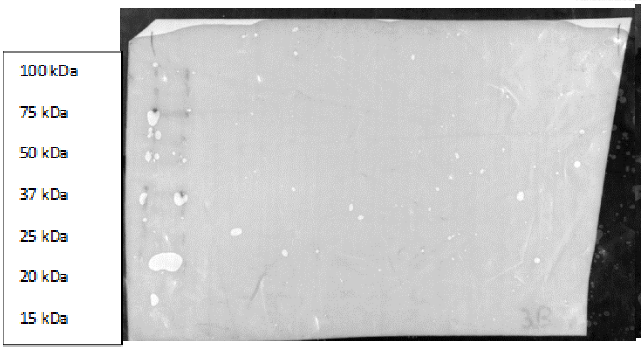

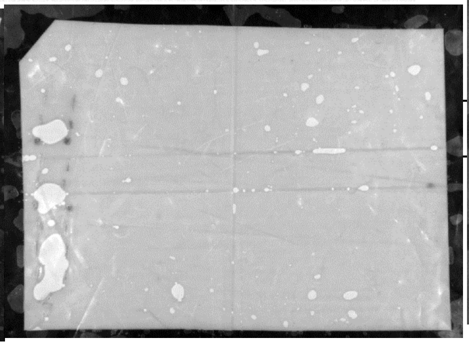

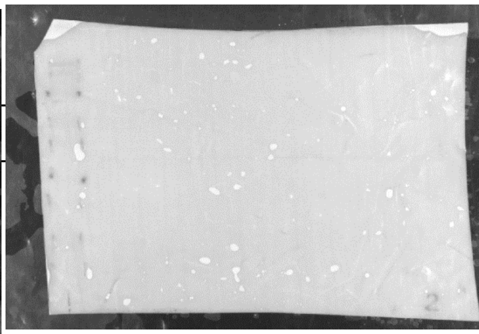

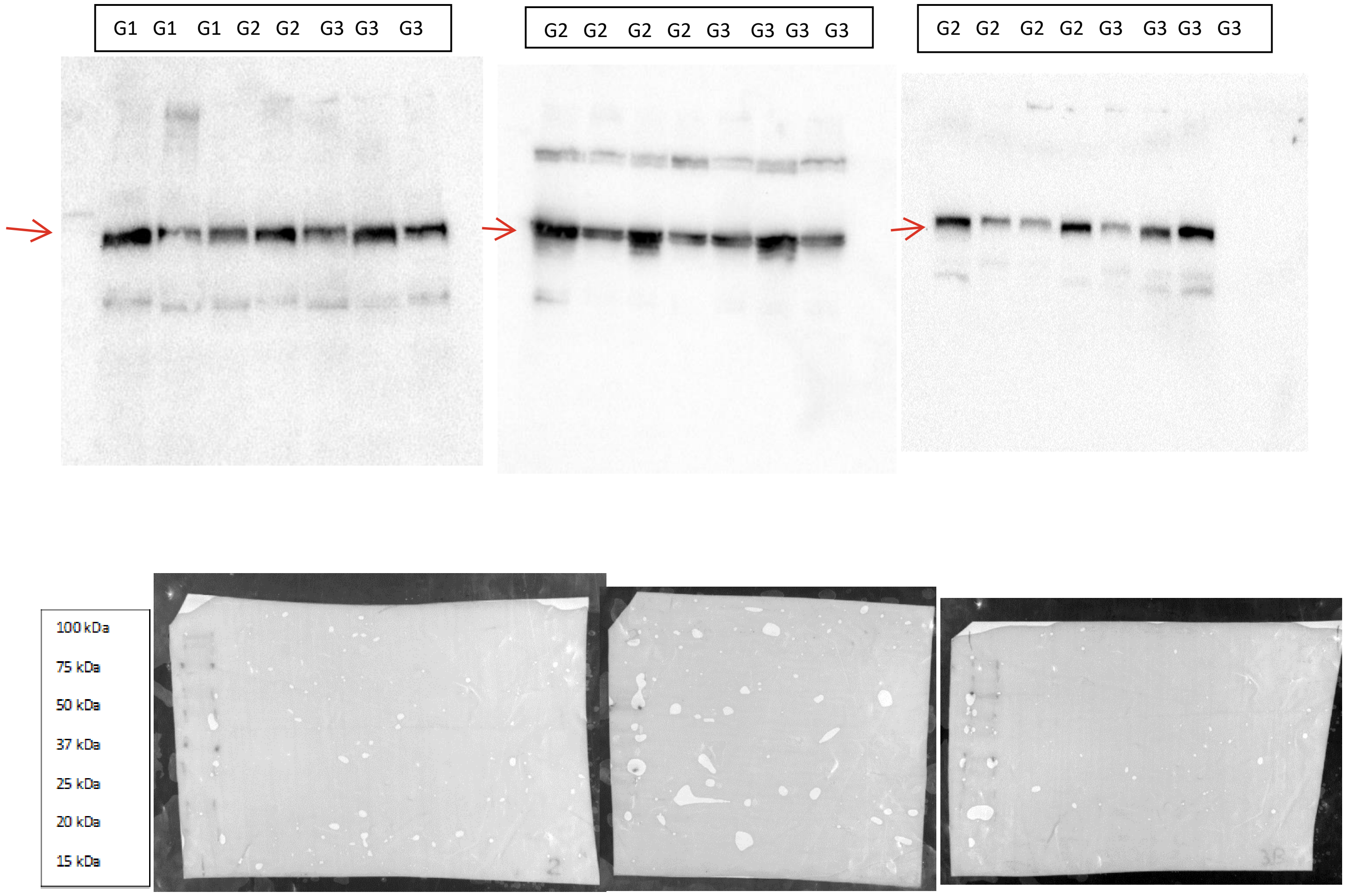

pERK1/2: 42-44 kDa

Replication 1 Replication 2 Replication 3

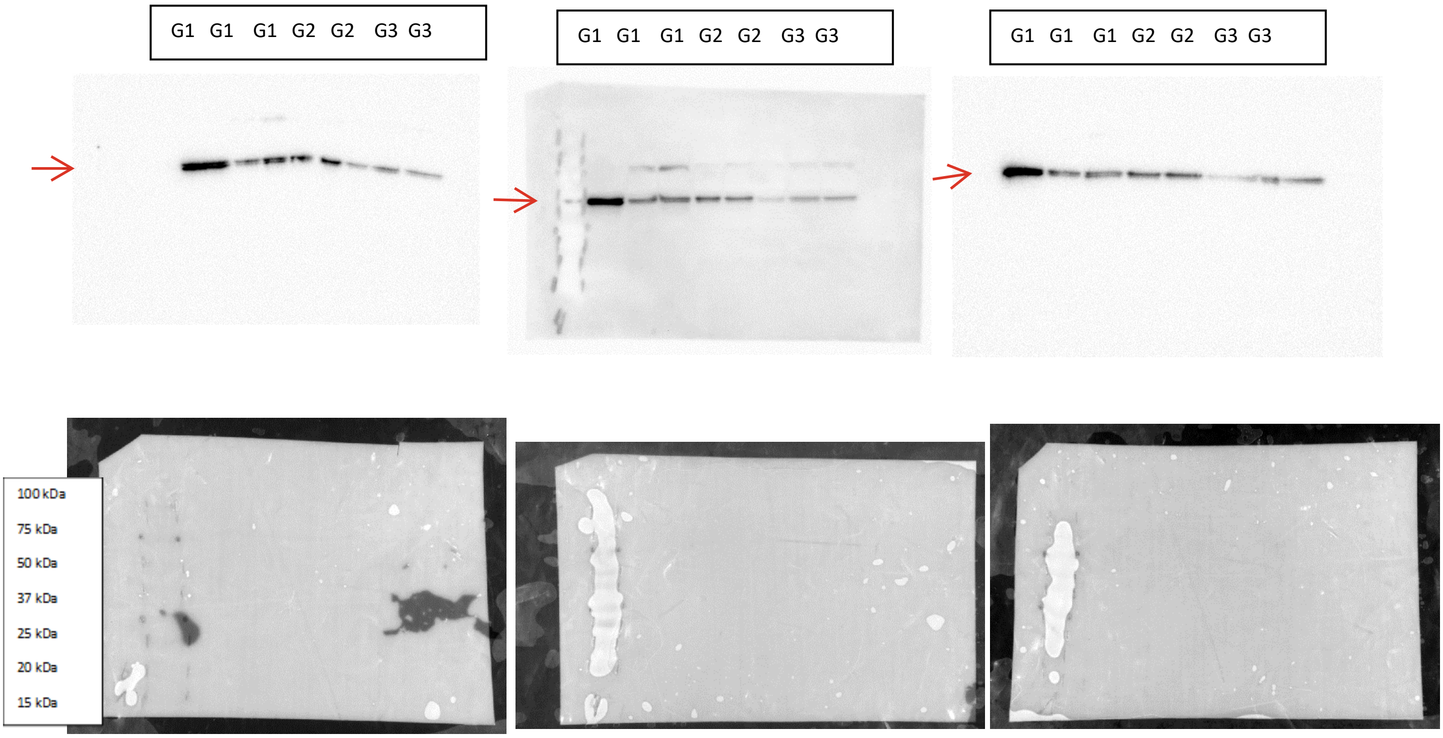

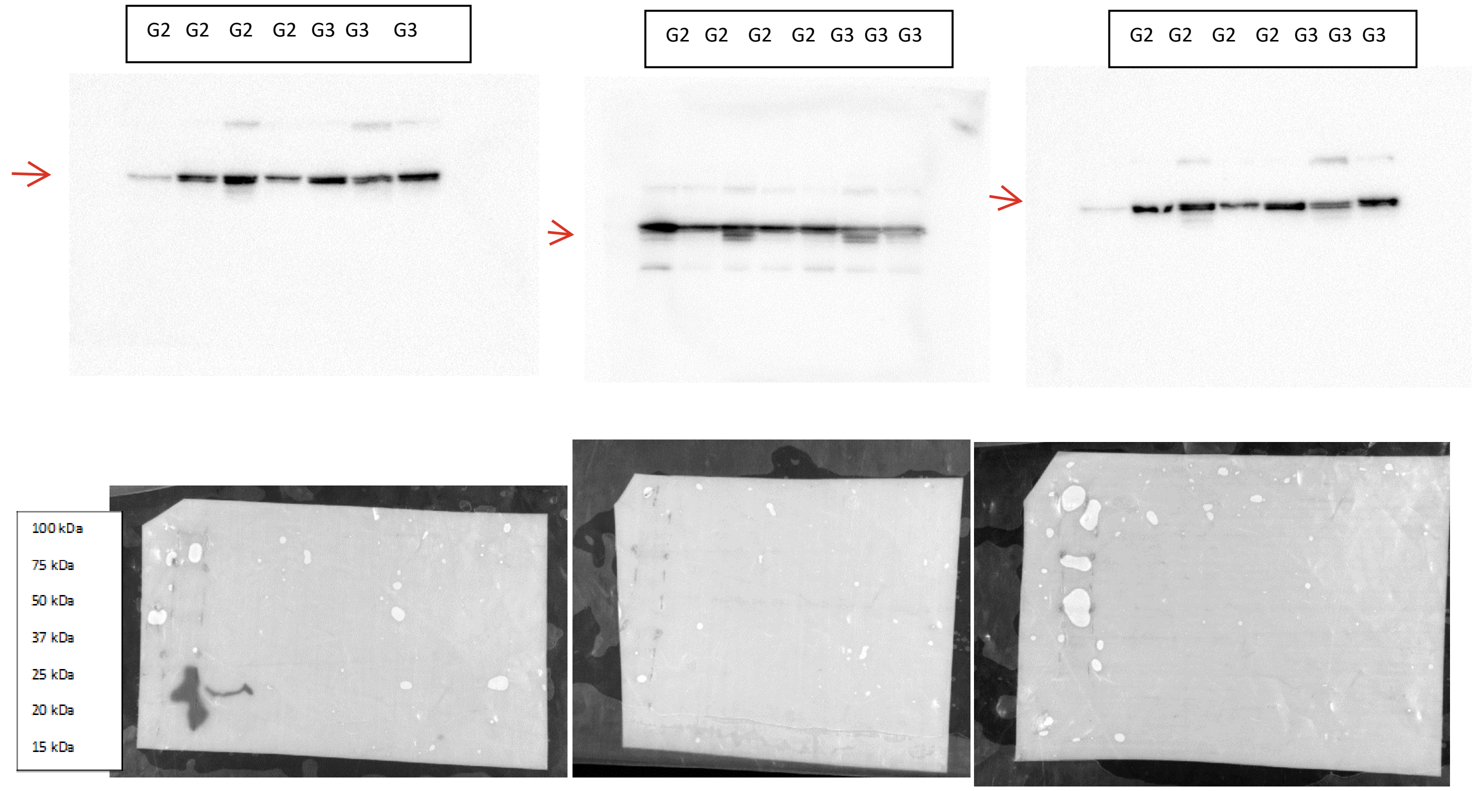
G2 G2 G2 G2 G3 G3 G3
G2 G2 G2 G2 G3 G3 G3
G2 G2 G2 G2 G3 G3 G3
100 kDa
75 kDa
50 kDa
37 kDa
25 kDa
20 kDa
15 kDa

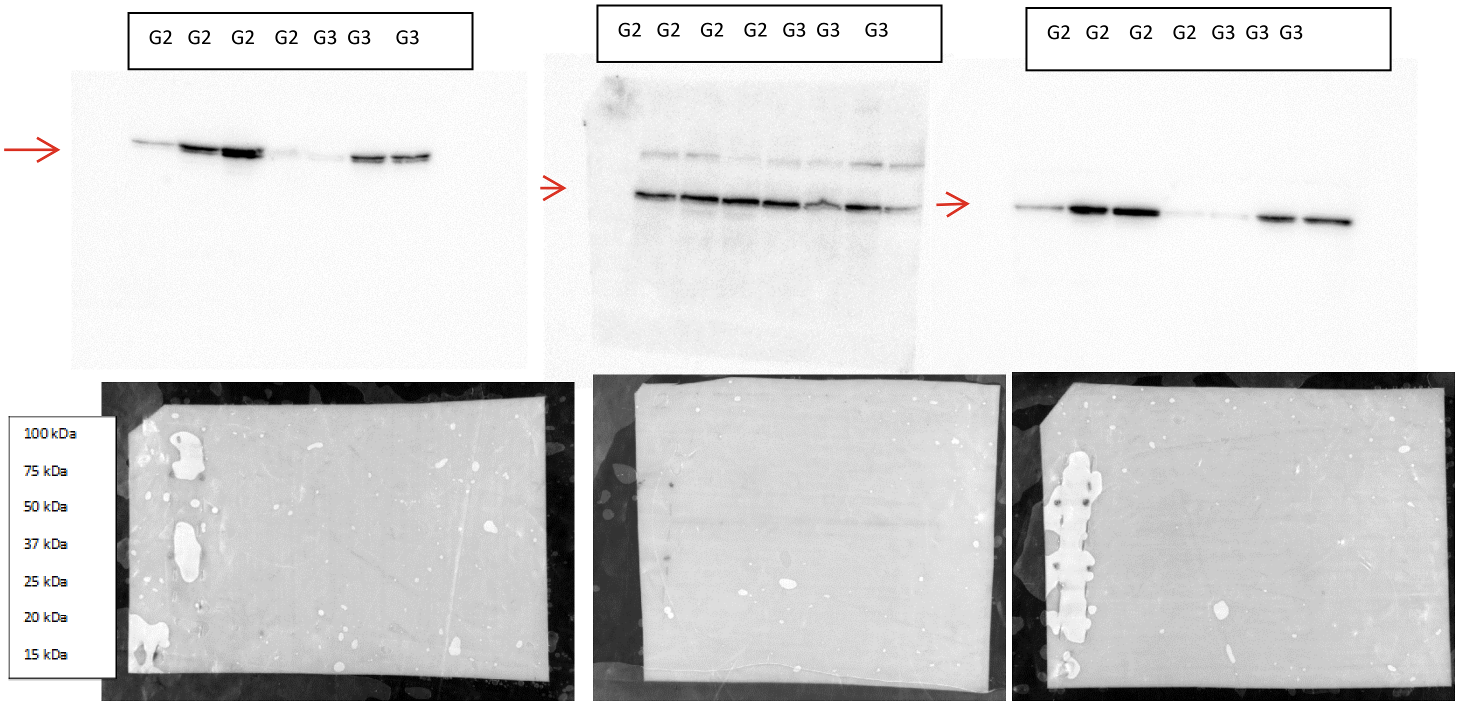
G2 G2 G2 G2 G3 G3 G3
G2 G2 G2 G2 G3 G3 G3
G2 G2 G2 G2 G3 G3 G3
100 kDa
75 kDa
50 kDa
37 kDa
25 kDa
20 kDa
15 kDa

ERK: 42-44kDa

Replication 1 Replication 2 Replication 3

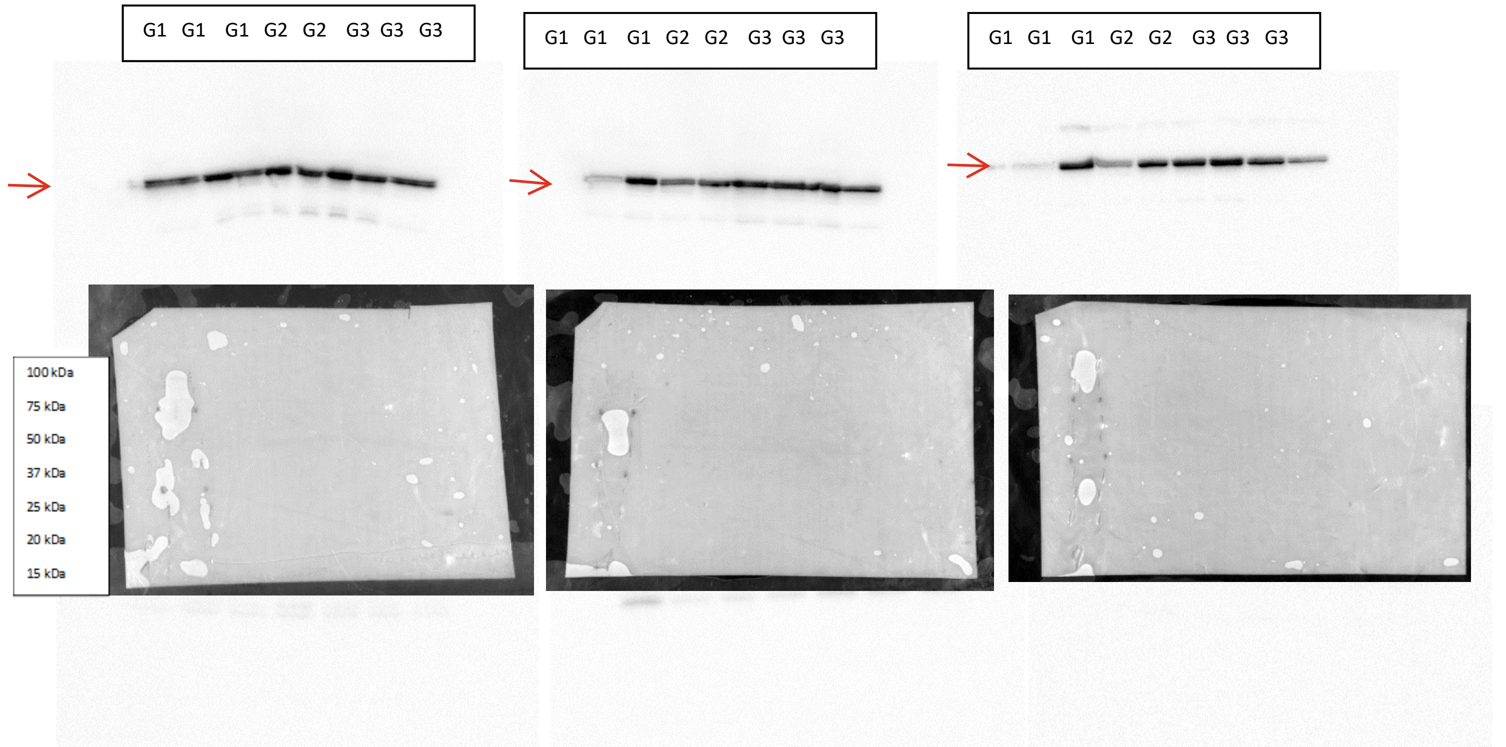

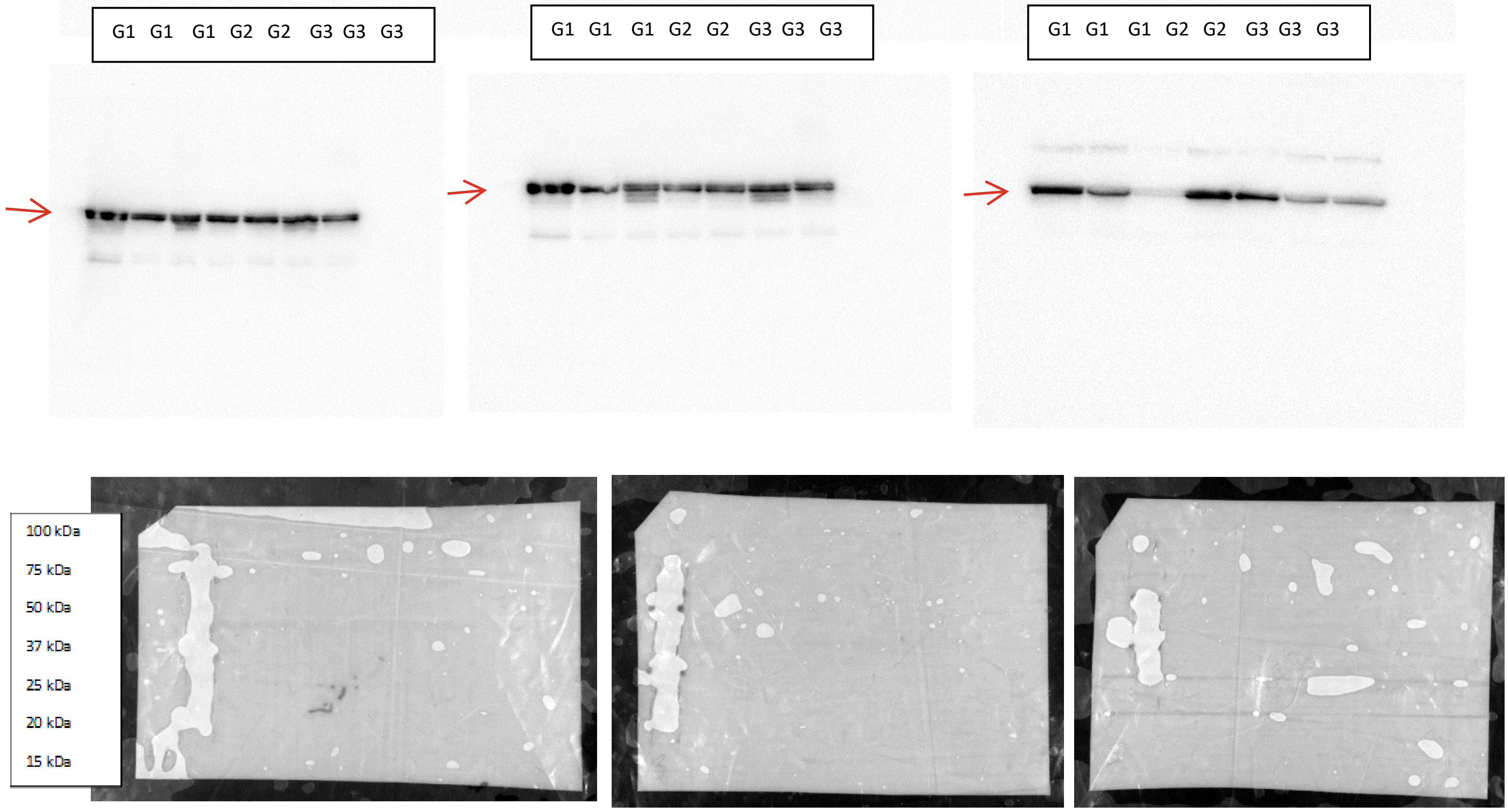

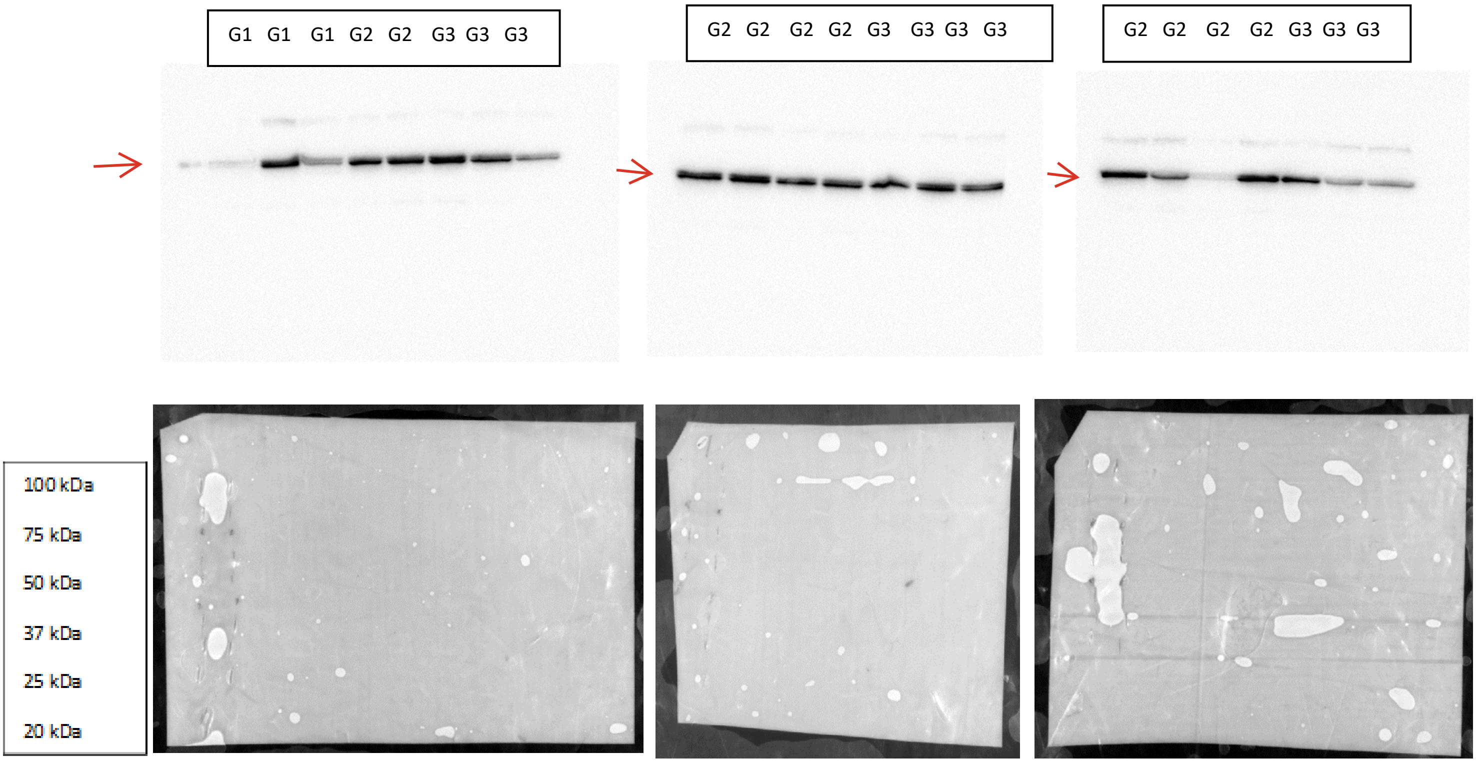
G1 G1 G1 G2 G2 G3 G3 G3
G2 G2 G2 G2 G3 G3 G3 G3
G2 G2 G2 G2 G3 G3 G3
100 kDa
75 kDa
50 kDa
37 kDa
25 kDa
20 kDa

VEGF-A: 23kDa

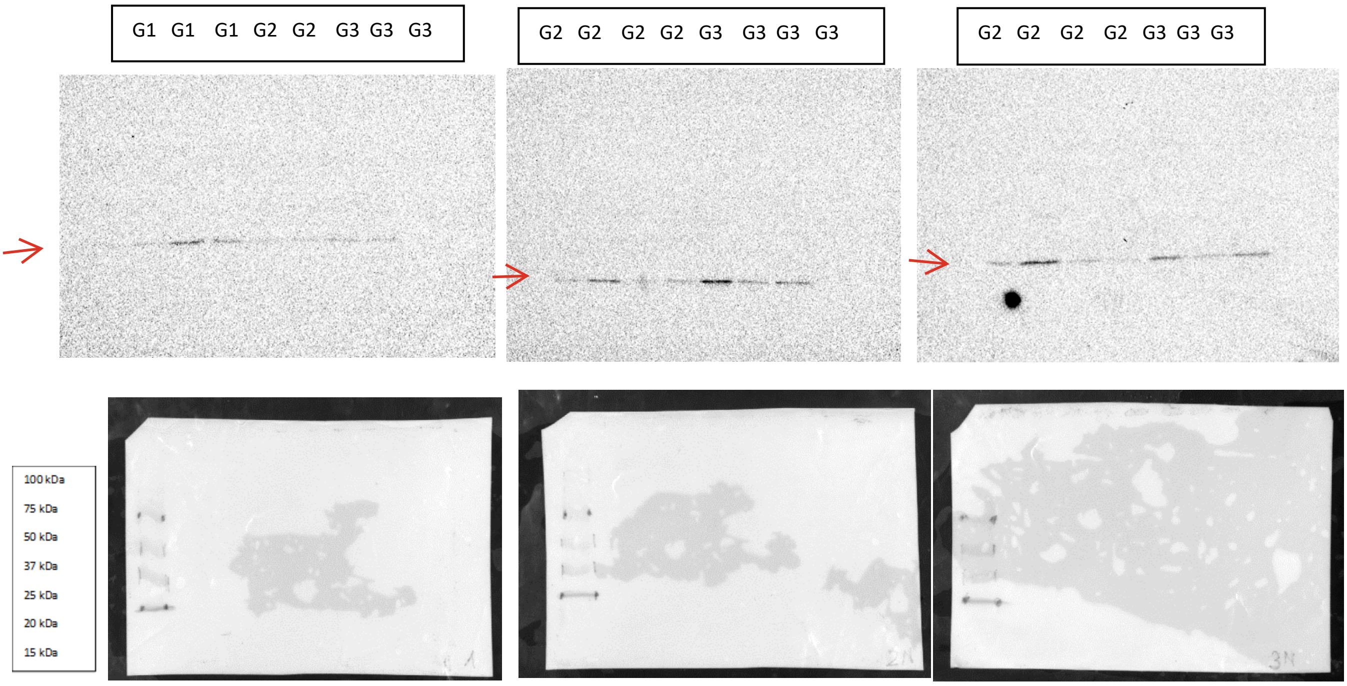
G1 G1 G1 G2 G2 G3 G3 G3
G2 G2 G2 G2 G3 G3 G3 G3
G2 G2 G2 G2 G3 G3 G3
100 kDa
75 kDa
50 kDa
37 kDa
25 kDa
20 kDa
15 kDa

VEGF-C: 20 kDa

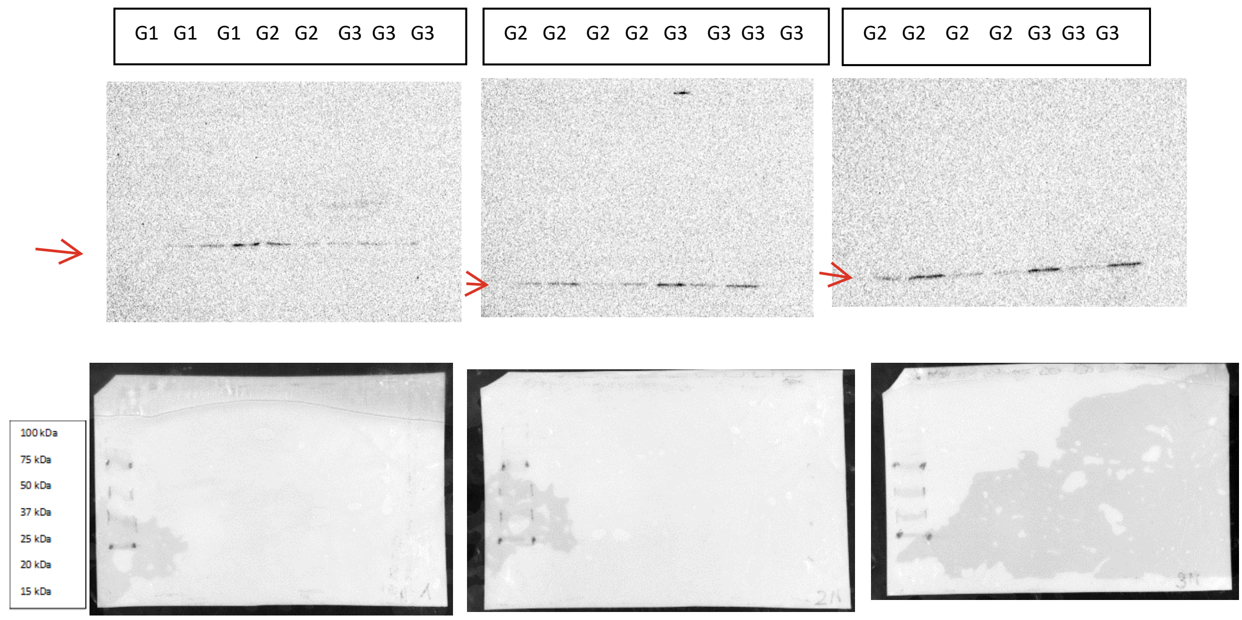

VEGF-D: 40 kDa

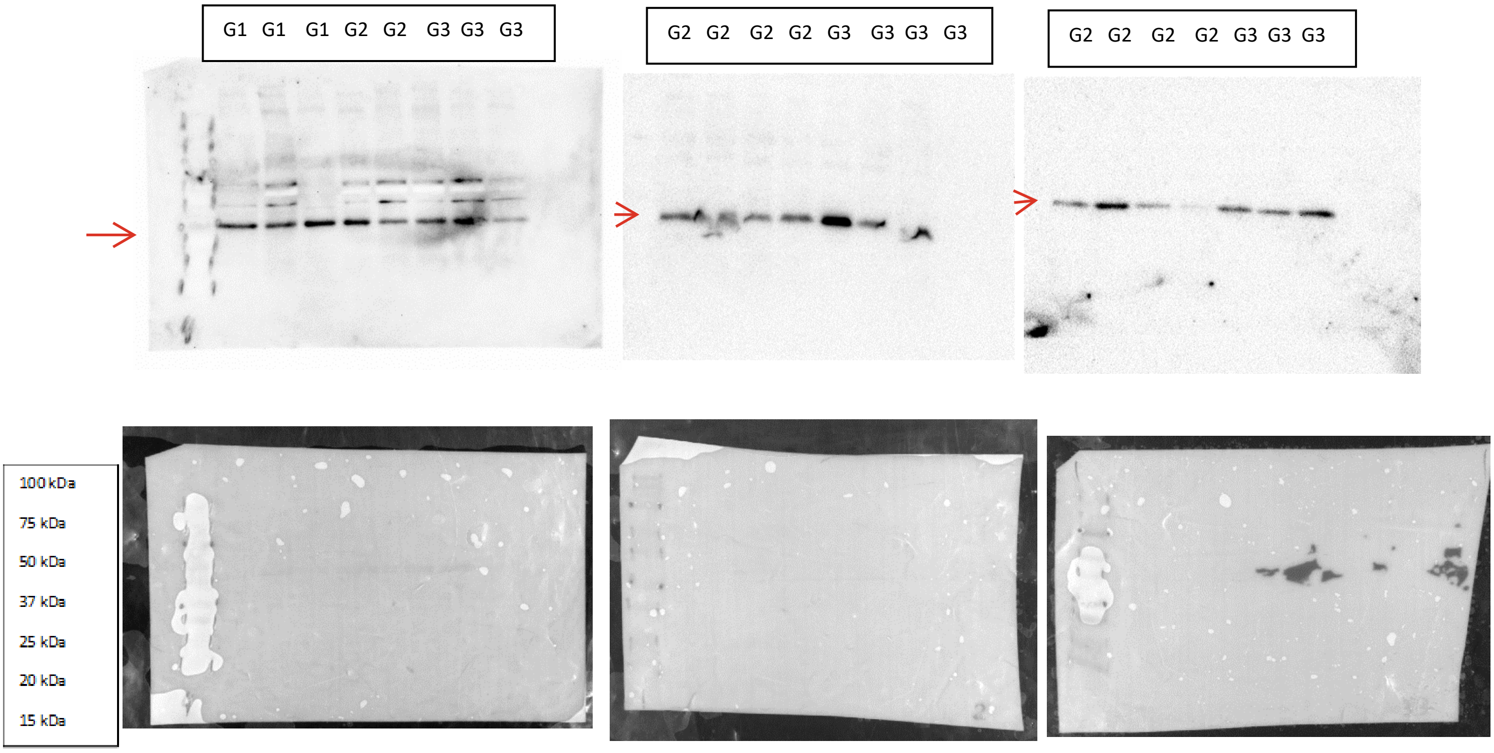

Human breast cancer cell lines

CHI3L2: 40 kDa

Me16c MCF7 MCF10A SKBR3 T47D BT474 BT549

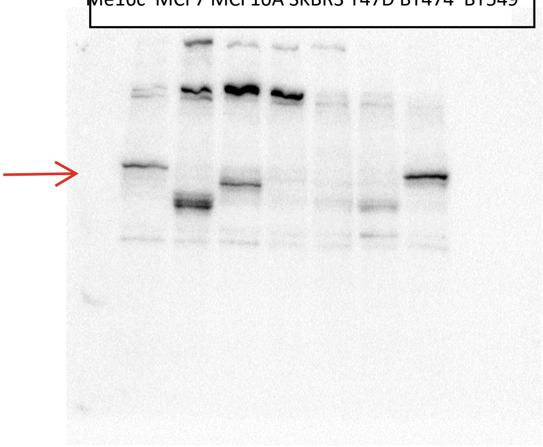

MDA231 MDA436 MDA468 BO2 M M/MCF7 M/M468

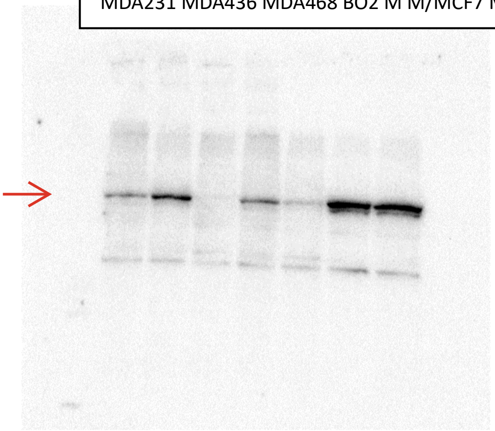

100 kDa
75 kDa
50 kDa
37 kDa
25 kDa
20 kDa
15 kDa

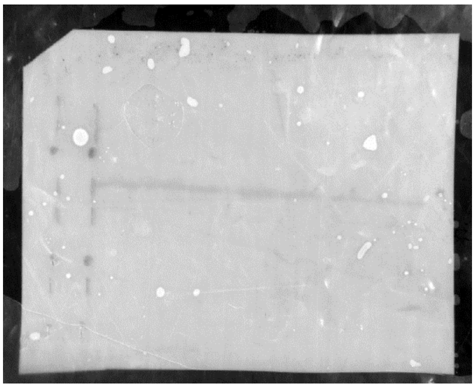

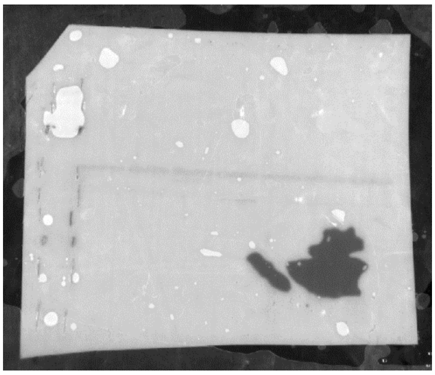

β-tubulin: 50 kDa

Me16c MCF7 MCF10A SKBR3 T47D BT474

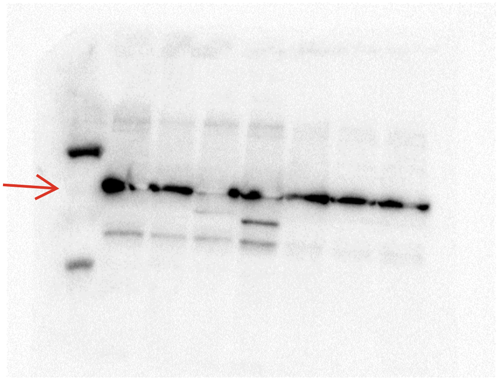

MDA231 MDA436 MDA468 BO2 M M/MCF7 M/M468

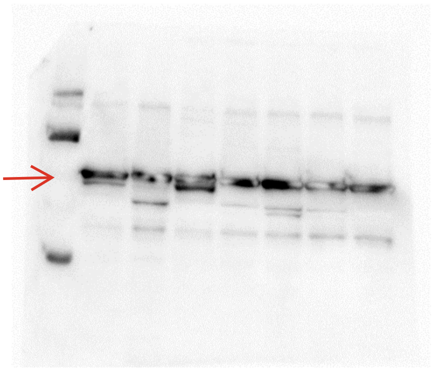

100 kDa
75 kDa
50 kDa
37 kDa
25 kDa
20 kDa
15 kDa

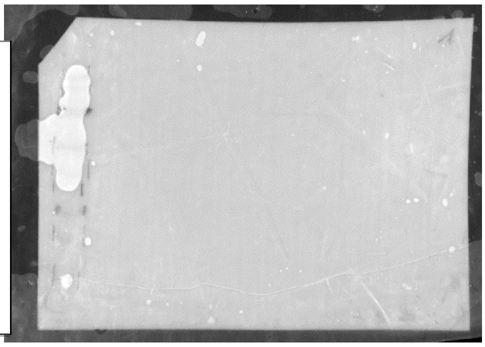

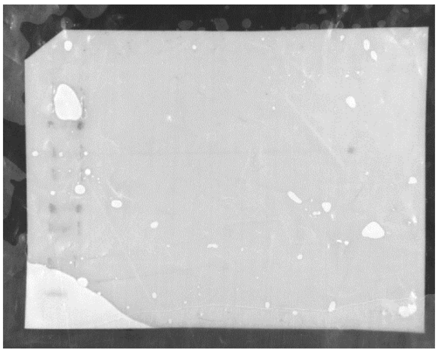

pSTAT-3: 88kDa

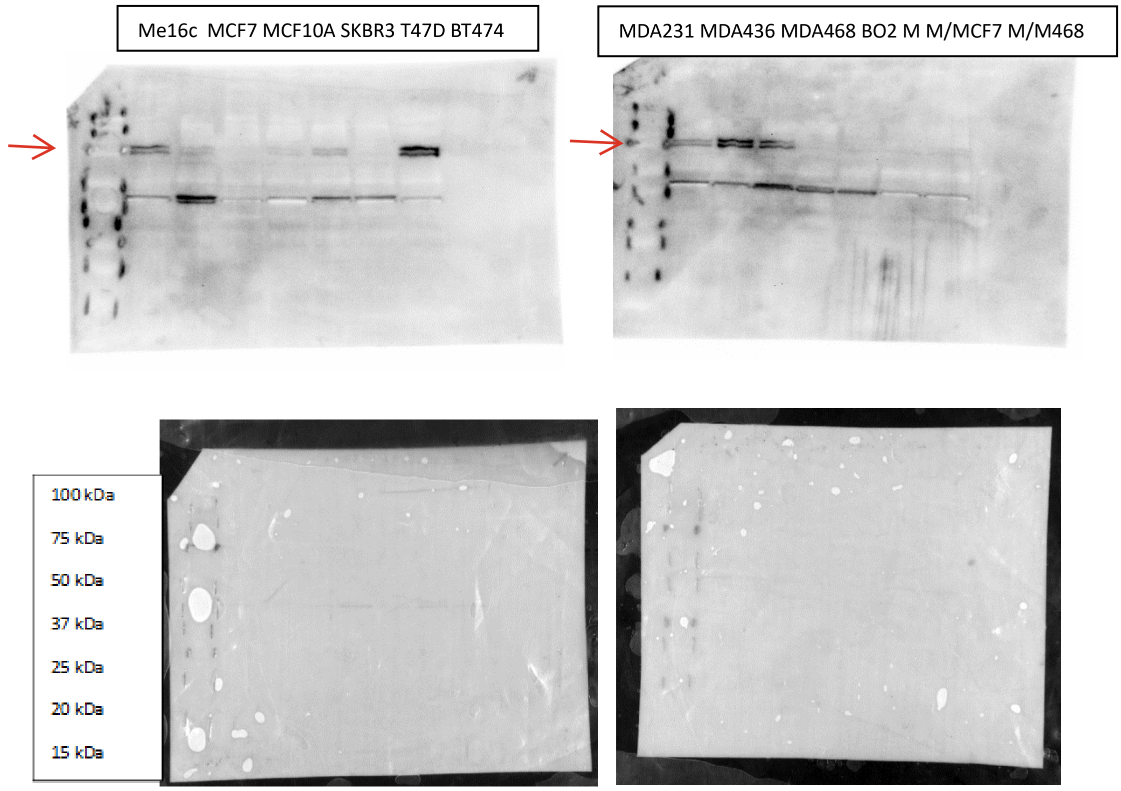

STAT-3: 92kDa

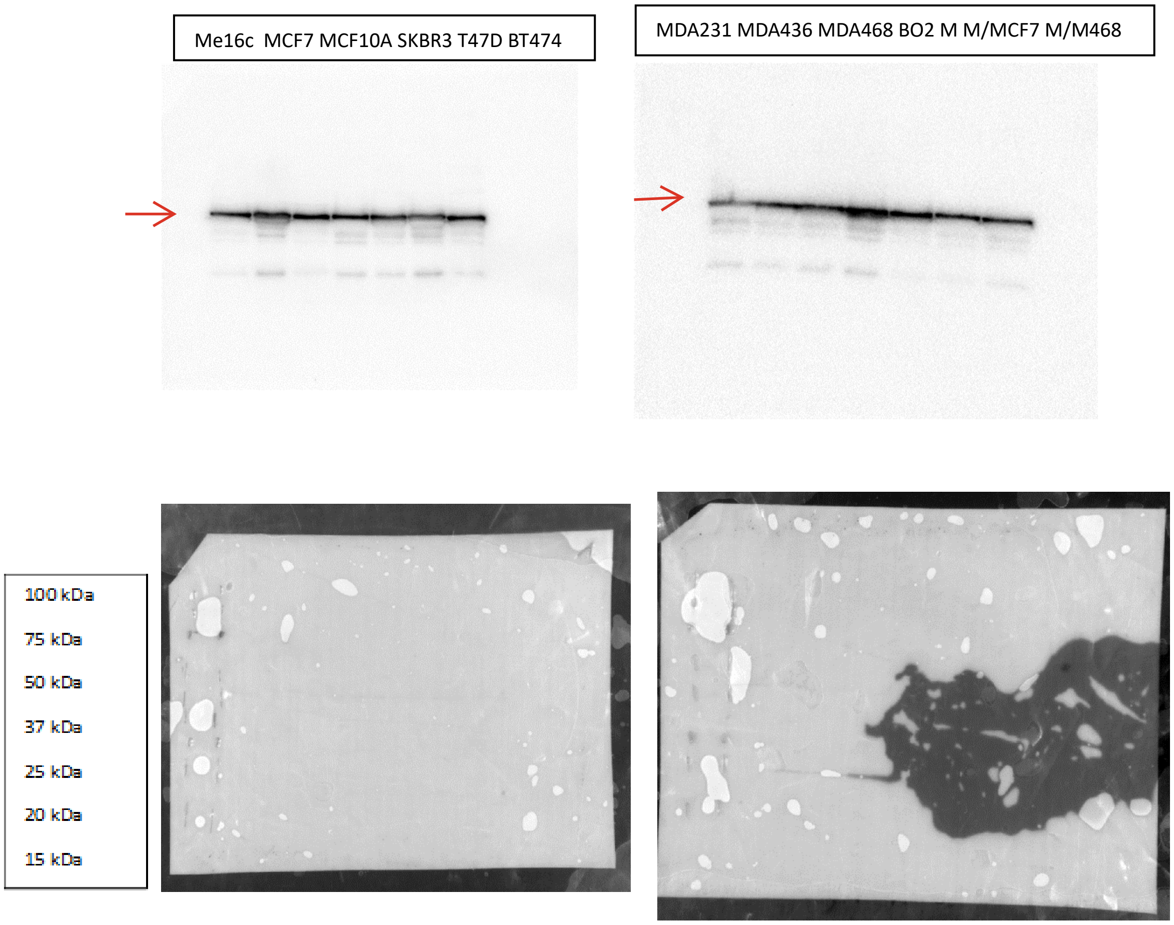

pERK1/2: 42-44kDa

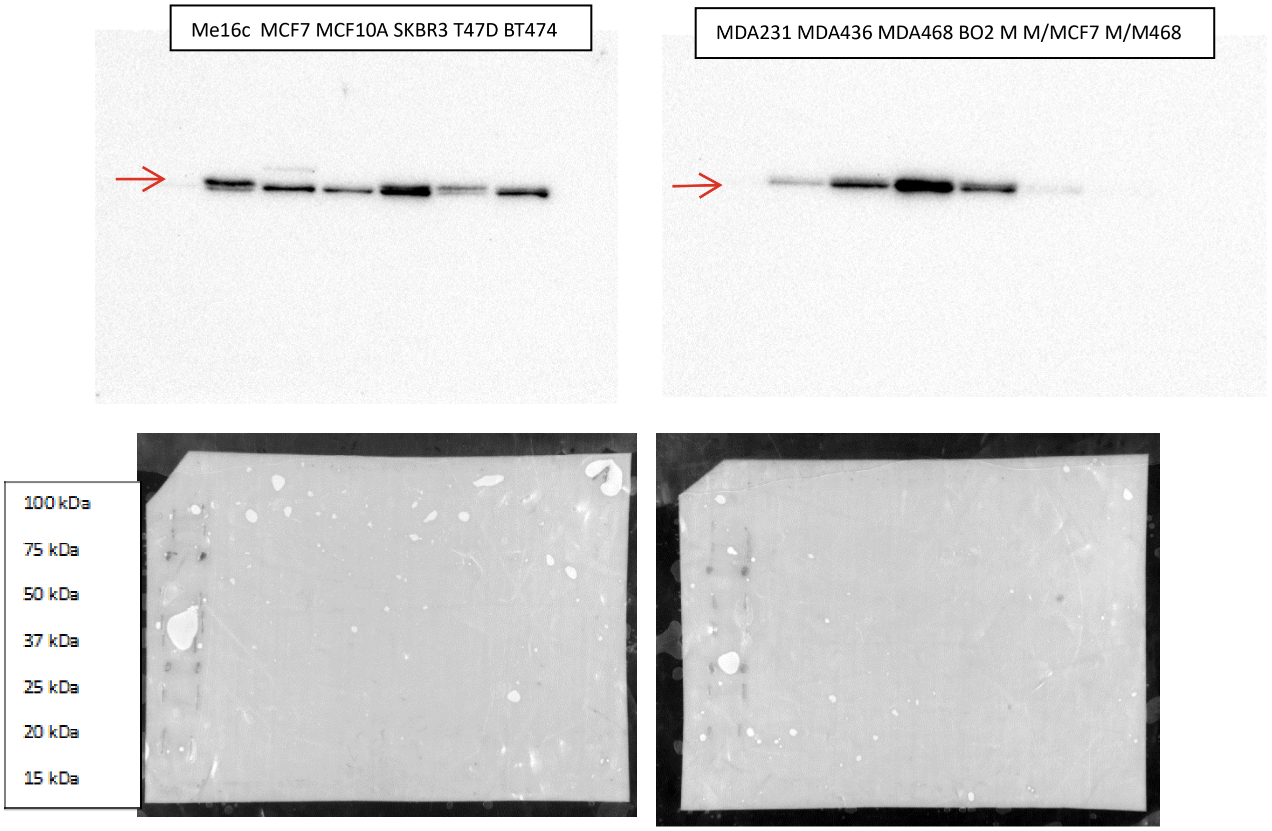

ERK1/2: 42-44kDa

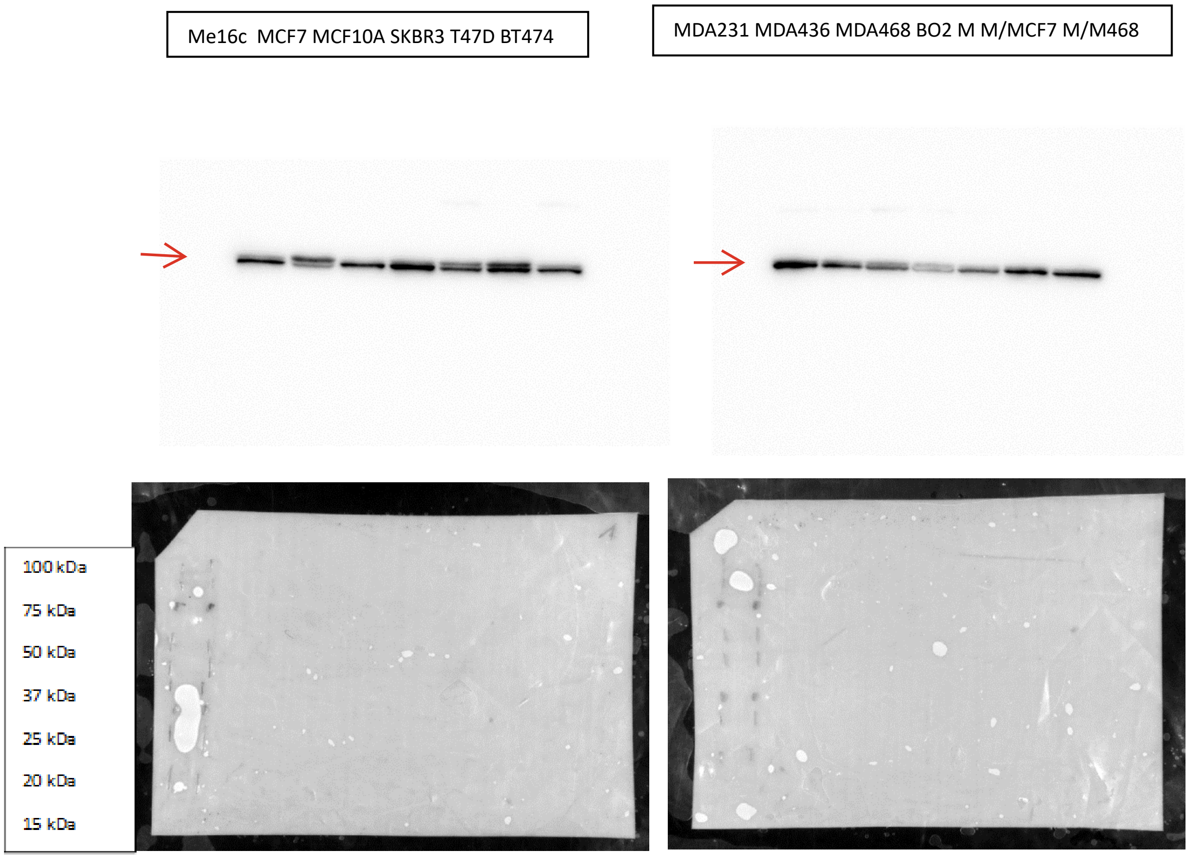

Transfection experiments with CHI3L2 siRNA

Sample legend

1: MDA231

2: MDA231 + siRNA 24h

3: MDA231 + siRNA 48h

4: BT549

5: BT549 + siRNA 24h

6: BT549 + siRNA 48h

CHI3L2: 40 kDa

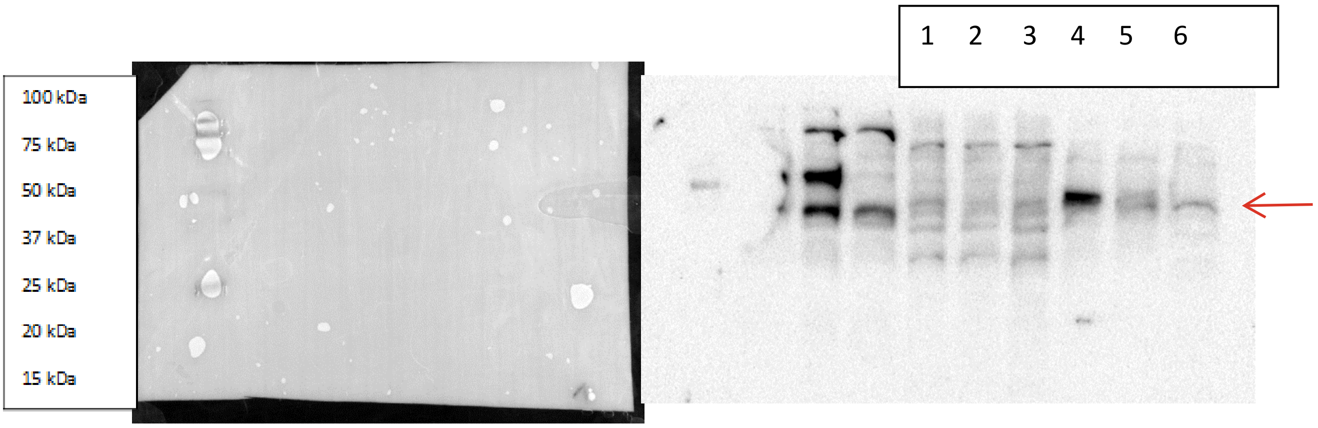

STAT-3: 92 kDa

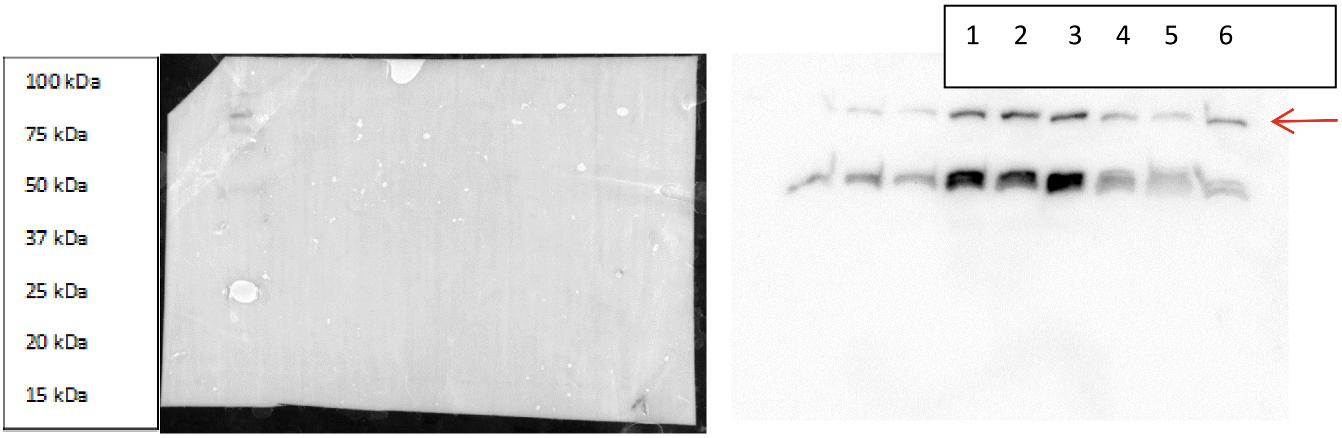

pSTAT3: 88 kDa

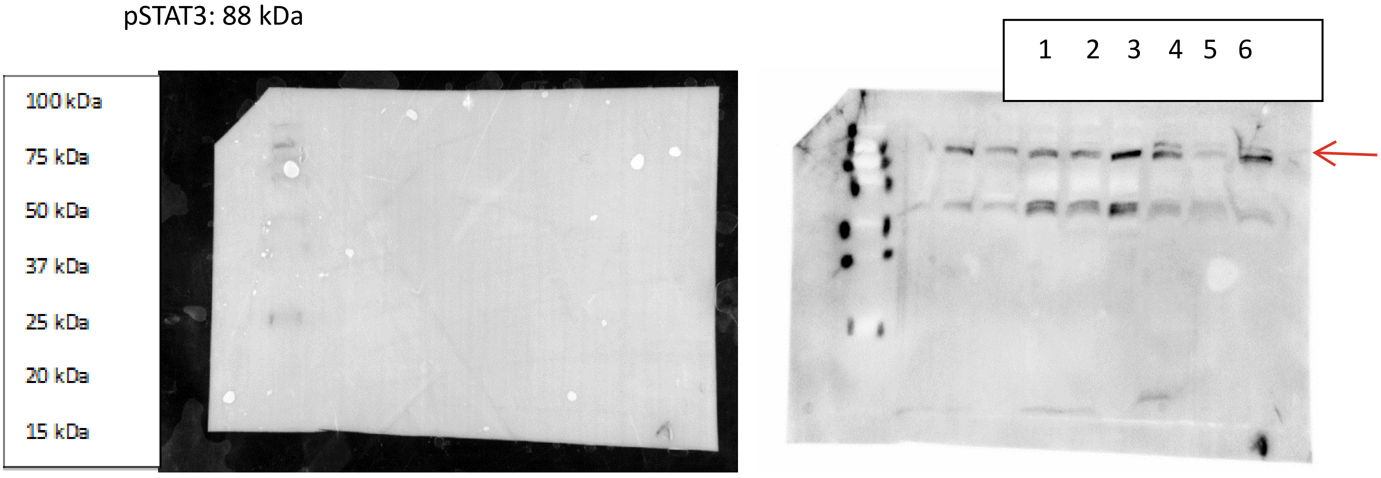

pERK1/2: 42-44 kDa

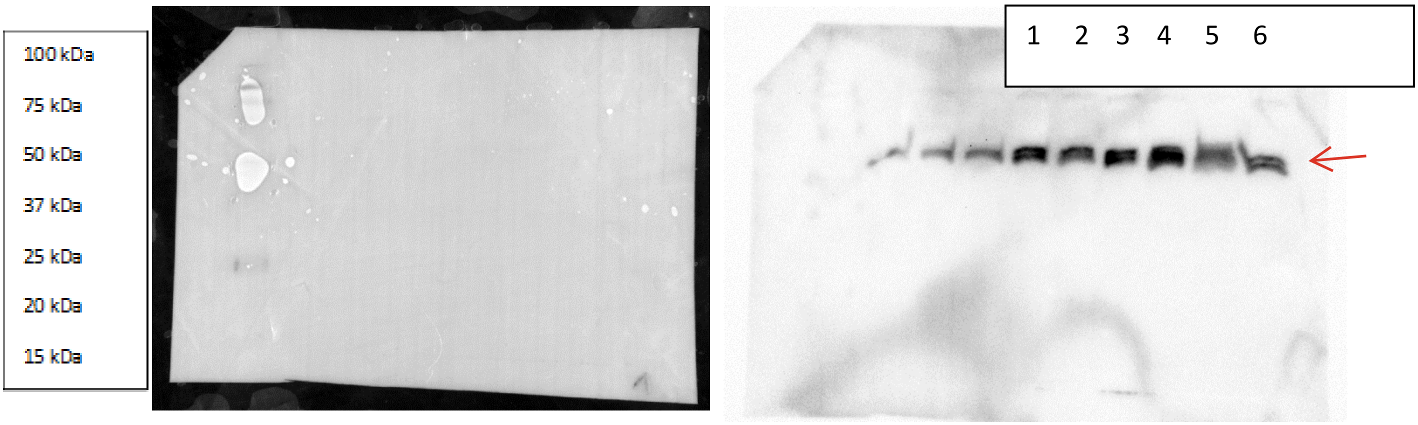

ERK1/2: 42-44 kDa

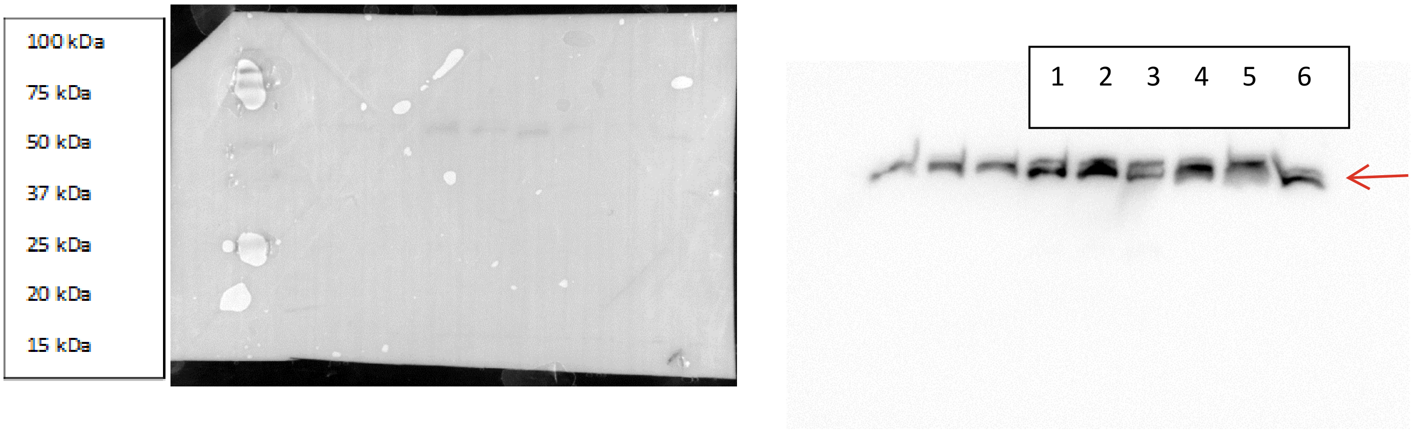

β-tubulin: 50 kDa

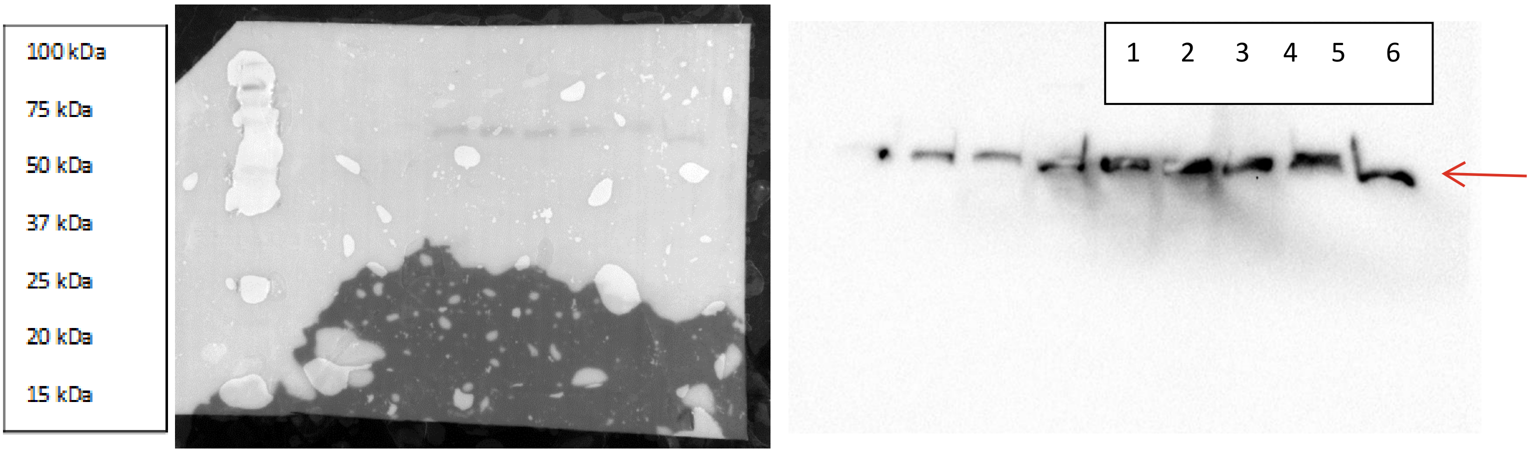

Experiments with CHI3L2 recombinant protein

Sample legend

1: MDA-MB-468 + CHI3L2 24h

2: MDA-MB-468 + CHI3L2 48h

3: MDA-MB-468

4: BT474+ CHI3L2 24h

5: BT474+ CHI3L2 48h

6: BT474

7: Macrophages

8: Macrophages /Me16C

9: Macrophages/M231

CHI3L2: 40 kDa

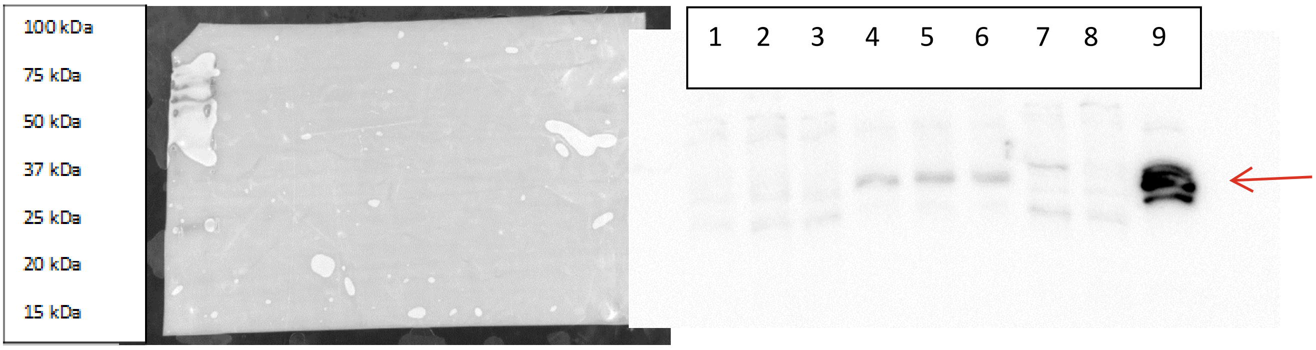

pSTAT-3: 88 kDa

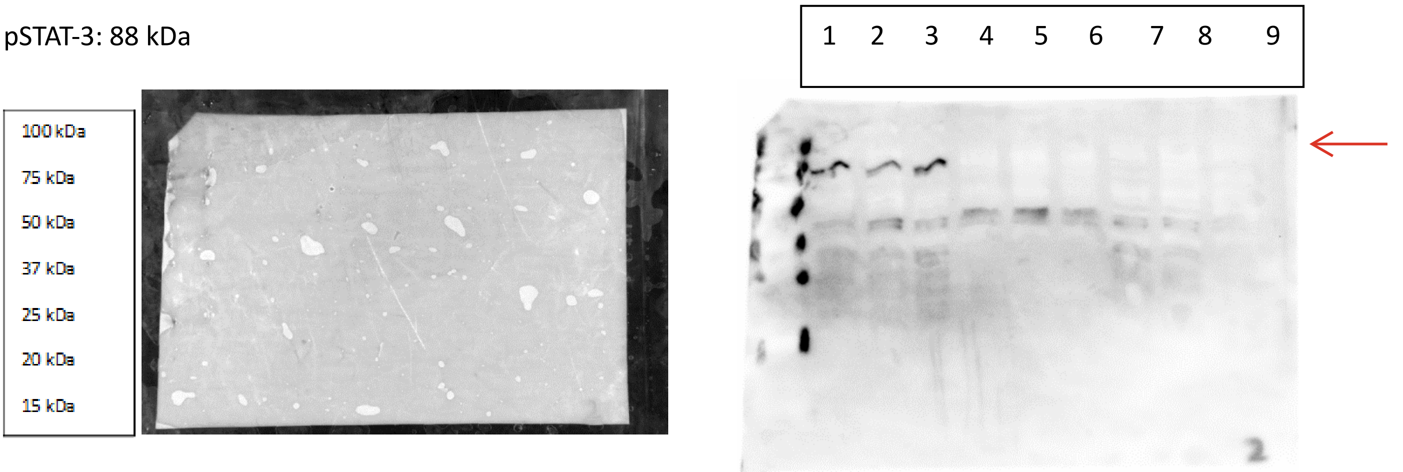

STAT-3: 92 kDa

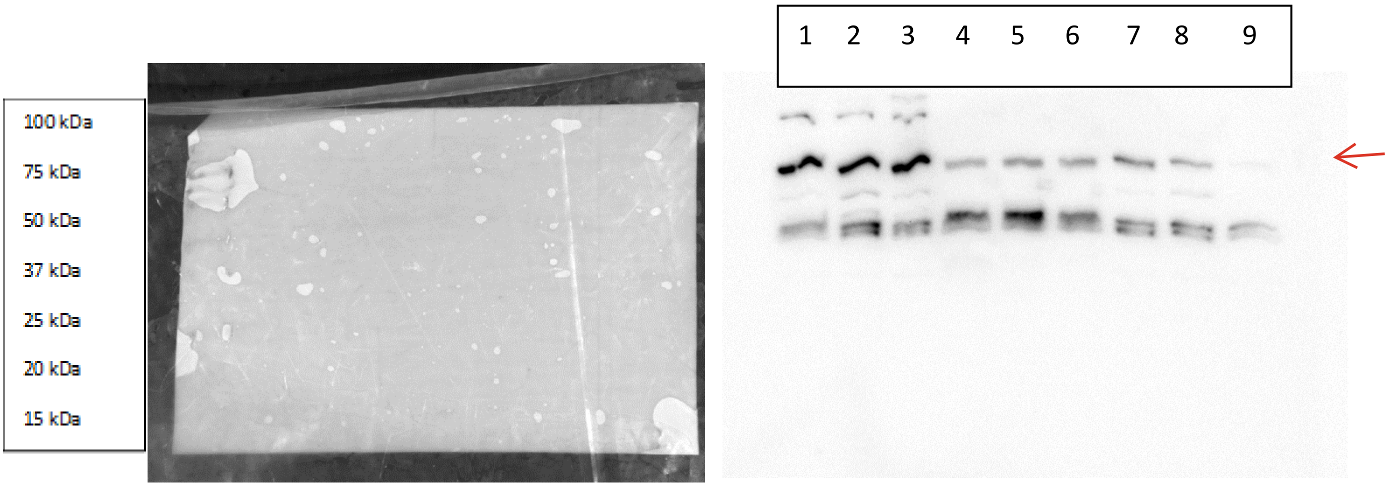

pERK1/2: 42-44 kDa

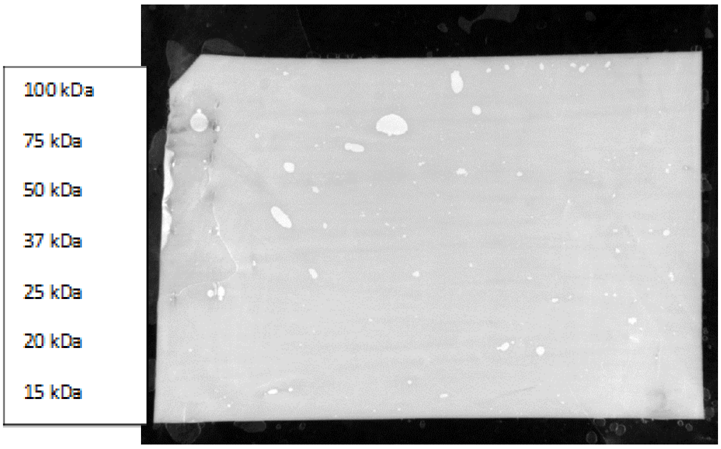

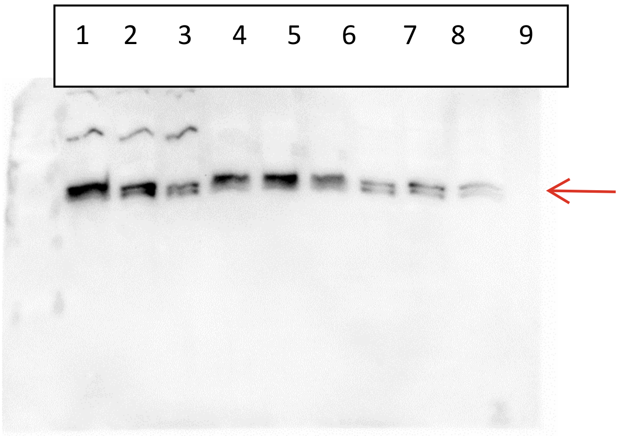

ErK1/2: 42-44 kDa

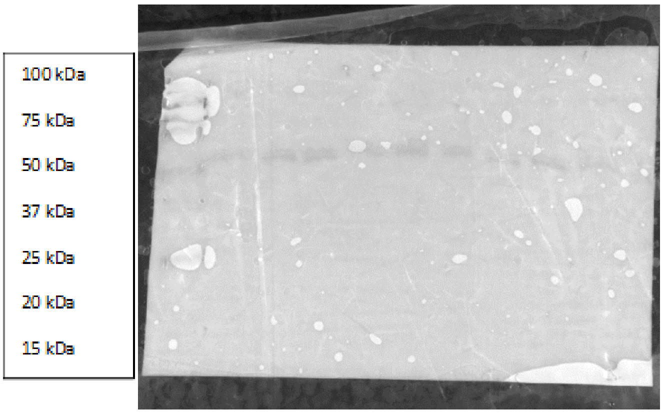

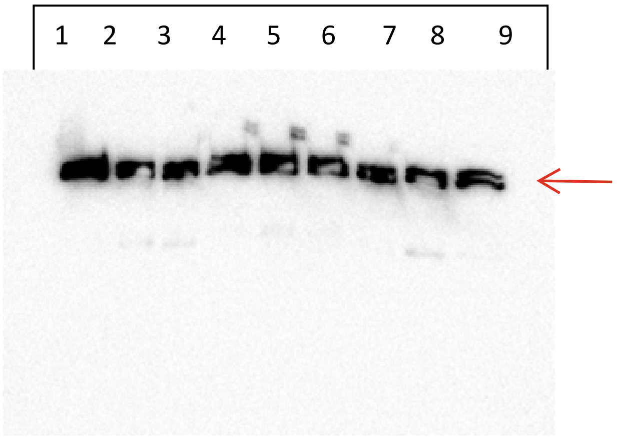

β-tubulin : 50 kDa

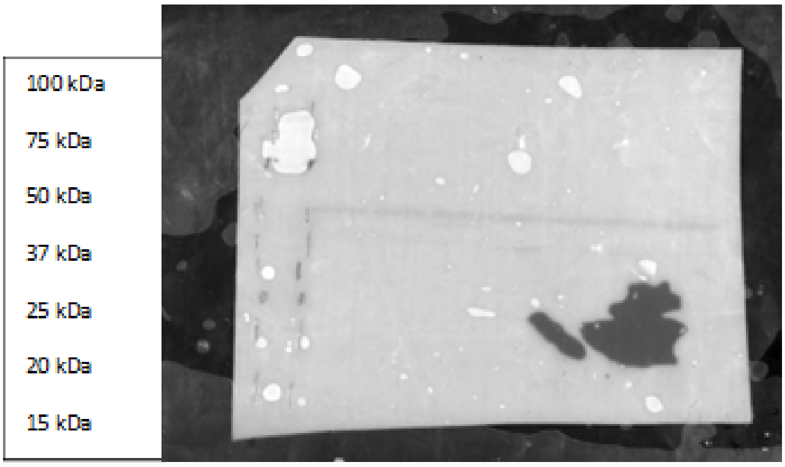

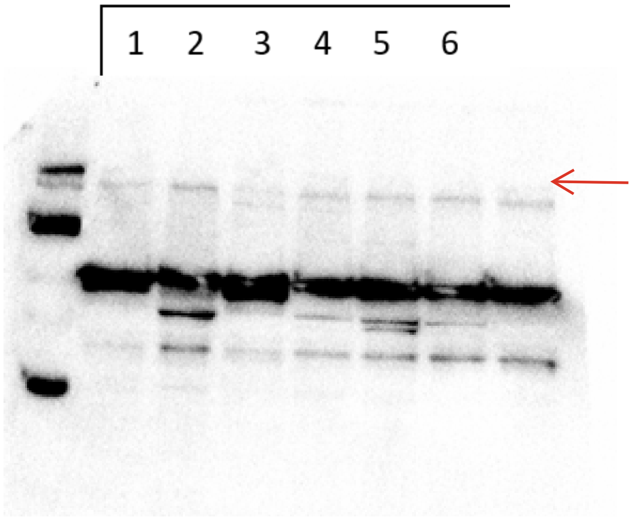

Image Report: 1 żel 1 powt tub UMED 2018-02-14 10hr 53min_Exposure_16.8sec

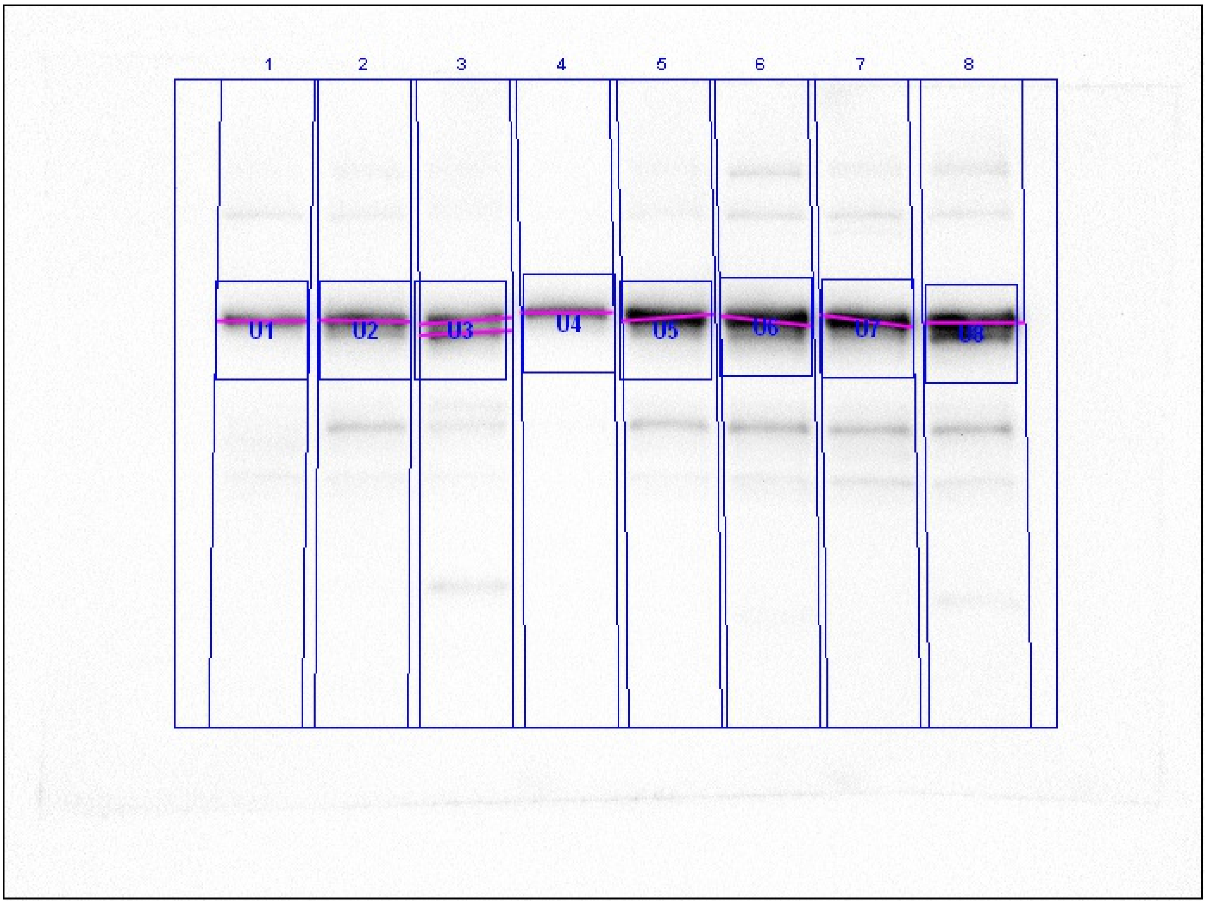

C:\Documents and Settings\Jaga\Pulpit\YKL-39 WB\analiza\żel 1\1 tub\1 żel 1 powt tub UMED 2018-02-14 10hr 53min_Exposure_16.8sec.scn

---

Acquisition Information

Imager	ChemiDoc™ MP
Exposure Time (sec)	16.800 (Signal Accumulation)
Flat Field	Applied (Lens)
Serial Number	731BR01769
Software Version	5.2.1
Application	Chemi Hi Resolution
Excitation Source	No Illumination
Emission Filter	No Filter
Binning	2x2

Image Information

Acquisition Date	2018-02-14 10:53:33
User Name	UMED
Image Area (mm)	X: 97.0 Y: 72.5
Pixel Size (um)	X: 139.4 Y: 139.4
Data Range (Int)	0 - 26024

Analysis Settings

Detection	Lane detection:<br>Automatically detected lanes with manual adjustments<br><br>Band detection:<br>Automatically detected bands with sensitivity: Low<br>Manually adjusted bands<br><br>Lane Background Subtraction:<br>Lane background subtracted with disk size: 10<br><br>Lane width: Variable
Volume Analysis	Background subtraction method: Local<br>Quantity regression method: Linear

Lane And Band Analysis

Lane 1

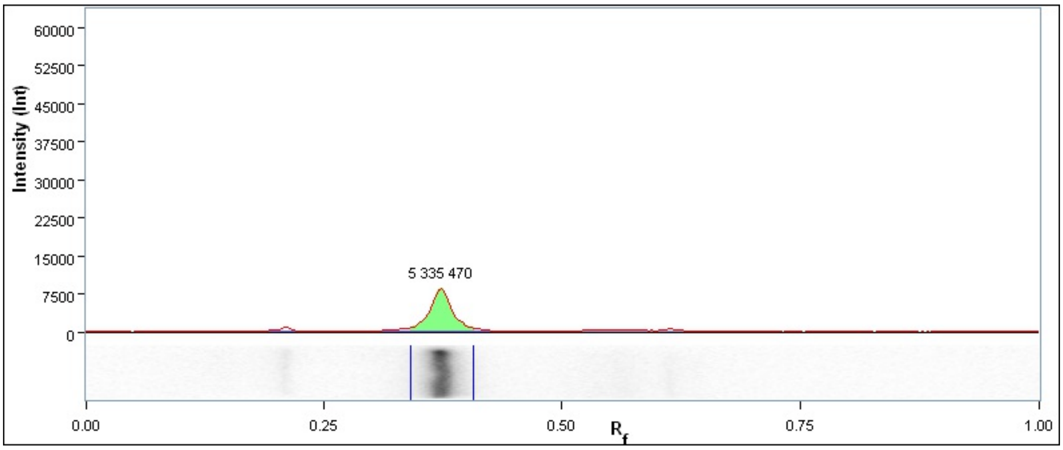

Band No.	Band Label	Mol. Wt. (KDa)	Relative Front	Volume (Int)	Abs. Quant.	Rel. Quant.	Band %	Lane %
1		N/A	0,374	5 335 470	N/A	N/A	100,0	71,9

Band Detection	Automatically detected bands with sensitivity: Low
Lane Background	Lane background subtracted with disk size: 10
Lane Width	7.53 mm

Lane 2

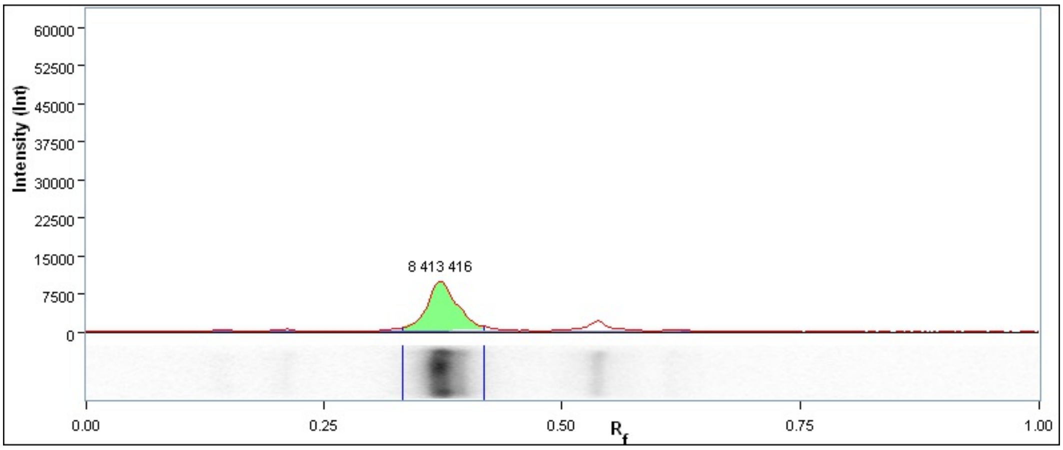

Band No.	Band Label	Mol. Wt. (KDa)	Relative Front	Volume (Int)	Abs. Quant.	Rel. Quant.	Band %	Lane %
1		N/A	0,374	8 413 416	N/A	N/A	100,0	74,2

Band Detection	Automatically detected bands with sensitivity: Low

Lane Background	Lane background subtracted with disk size: 10
Lane Width	7.53 mm

Lane 3

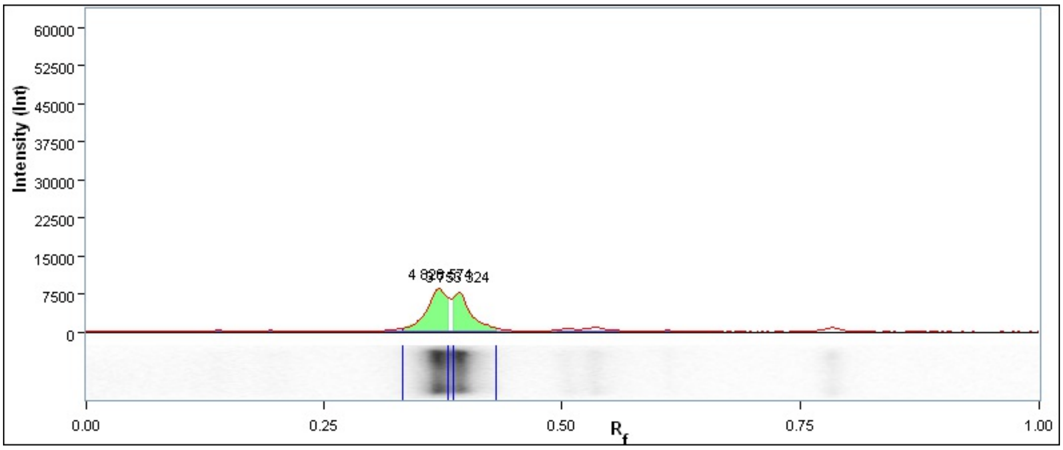

Band No.	Band Label	Mol. Wt. (KDa)	Relative Front	Volume (Int)	Abs. Quant.	Rel. Quant.	Band %	Lane %
1		N/A	0,374	4 826 574	N/A	N/A	56,3	43,3
2		N/A	0,393	3 753 324	N/A	N/A	43,7	33,7

Band Detection	Automatically detected bands with sensitivity: Low
Lane Background	Lane background subtracted with disk size: 10
Lane Width	7.53 mm

Lane 4

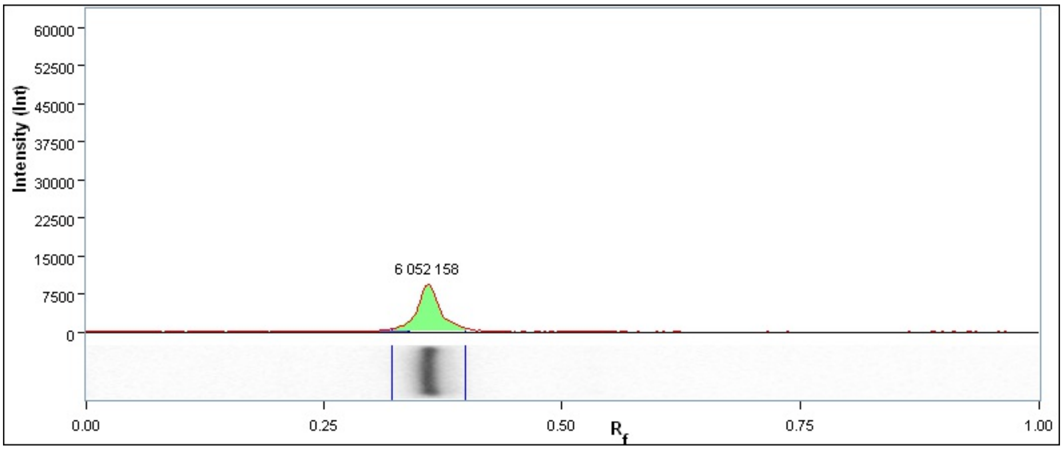

Band No.	Band Label	Mol. Wt. (KDa)	Relative Front	Volume (Int)	Abs. Quant.	Rel. Quant.	Band %	Lane %
1		N/A	0,361	6 052 158	N/A	N/A	100,0	83,8

Band Detection	Automatically detected bands with sensitivity: Low
Lane Background	Lane background subtracted with disk size: 10
Lane Width	7.53 mm

Lane 5

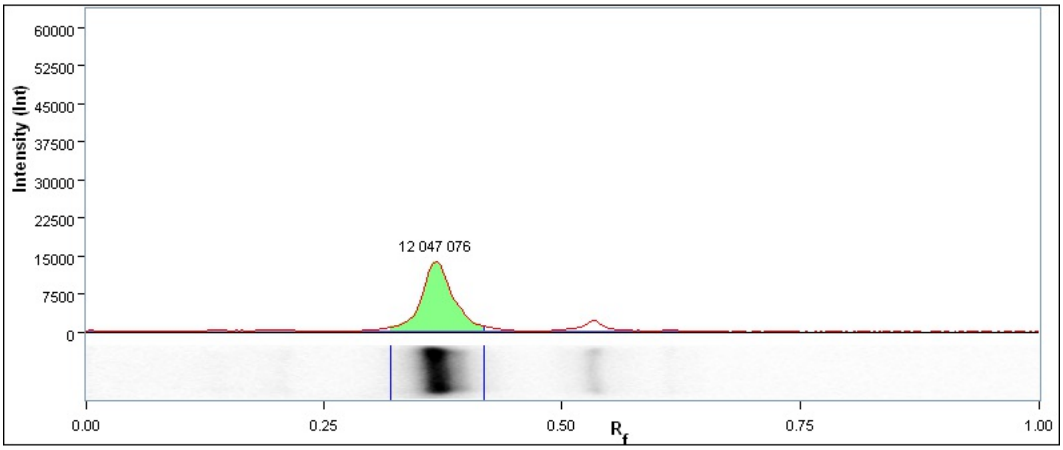

Band No.	Band Label	Mol. Wt. (KDa)	Relative Front	Volume (Int)	Abs. Quant.	Rel. Quant.	Band %	Lane %
1		N/A	0,369	12 047 076	N/A	N/A	100,0	80,3

Band Detection	Automatically detected bands with sensitivity: Low
Lane Background	Lane background subtracted with disk size: 10
Lane Width	7.53 mm

Lane 6

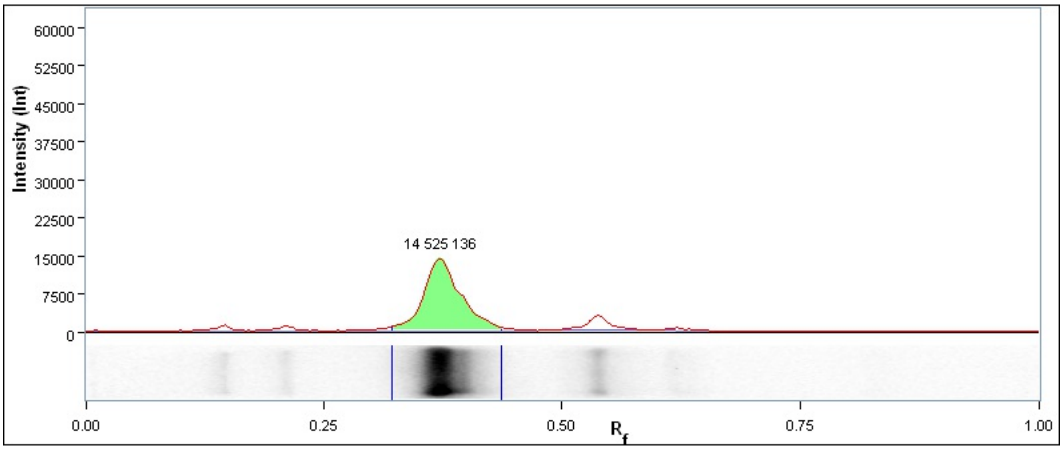

Band No.	Band Label	Mol. Wt. (KDa)	Relative Front	Volume (Int)	Abs. Quant.	Rel. Quant.	Band %	Lane %
1		N/A	0,374	14 525 136	N/A	N/A	100,0	76,4

Band Detection	Automatically detected bands with sensitivity: Low
Lane Background	Lane background subtracted with disk size: 10
Lane Width	7.53 mm

Lane 7

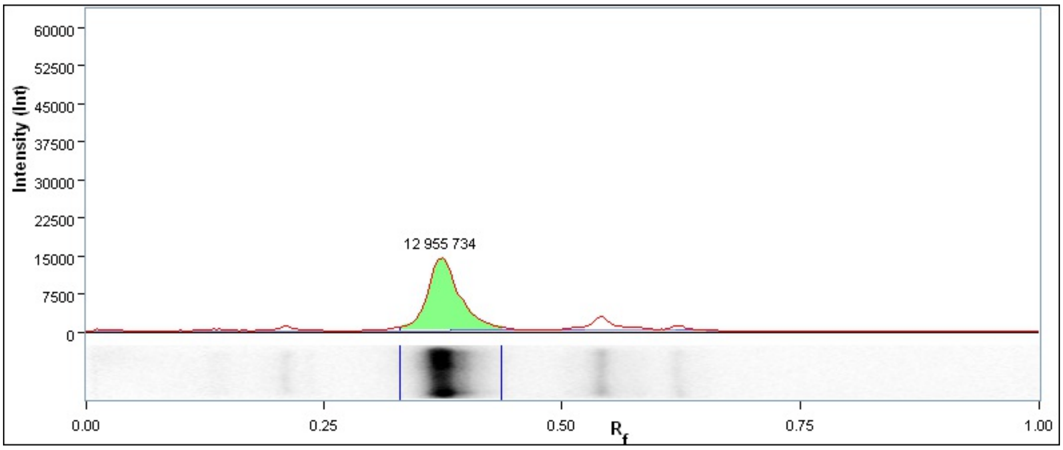

Band No.	Band Label	Mol. Wt. (KDa)	Relative Front	Volume (Int)	Abs. Quant.	Rel. Quant.	Band %	Lane %
1		N/A	0,374	12 955 734	N/A	N/A	100,0	76,6

Band Detection	Automatically detected bands with sensitivity: Low
Lane Background	Lane background subtracted with disk size: 10
Lane Width	7.53 mm

Lane 8

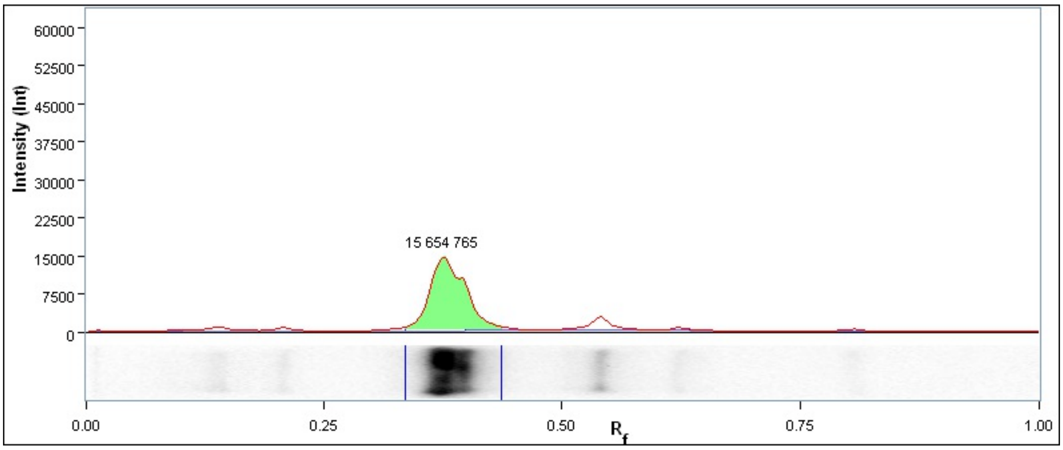

Band No.	Band Label	Mol. Wt. (KDa)	Relative Front	Volume (Int)	Abs. Quant.	Rel. Quant.	Band %	Lane %
1		N/A	0,377	15 654 765	N/A	N/A	100,0	77,5

Band Detection	Automatically detected bands with sensitivity: Low
Lane Background	Lane background subtracted with disk size: 10
Lane Width	8.22 mm

Volume Analysis

No.	Label	Type	Volume (Int)	Adj. Vol. (Int)	Mean Bkgd. (Int)	Abs. Quant.	Rel. Quant.	# of Pixels	Min. Value (Int)	Max. Value (Int)	Mean Value (Int)	Std. Dev.	Area (mm2)
1	U1	Unknown	6 412 800	4 697 951	567,6	N/A	N/A	3 021	0	13 768	2 122,7	2 792,3	58,7
2	U2	Unknown	9 737 924	7 275 323	815,2	N/A	N/A	3 021	0	15 444	3 223,4	3 539,6	58,7
3	U3	Unknown	9 947 944	7 362 292	855,9	N/A	N/A	3 021	0	14 112	3 292,9	3 497,2	58,7
4	U4	Unknown	6 741 460	4 815 465	637,5	N/A	N/A	3 021	0	12 436	2 231,5	2 955,9	58,7
5	U5	Unknown	13 185 080	10 254 872	969,9	N/A	N/A	3 021	0	19 784	4 364,5	5 058,0	58,7
6	U6	Unknown	15 887 160	11 697 033	1 387,0	N/A	N/A	3 021	0	21 256	5 258,9	5 223,1	58,7
7	U7	Unknown	14 483 916	10 436 315	1 339,8	N/A	N/A	3 021	0	20 248	4 794,4	5 206,0	58,7
8	U8	Unknown	16 883 060	12 662 669	1 397,0	N/A	N/A	3 021	0	26 024	5 588,6	5 976,0	58,7

Image Report: 1 żel 1 powt YKL39 UMED 2018-02-13 11hr 00min_Exposure_60.0sec

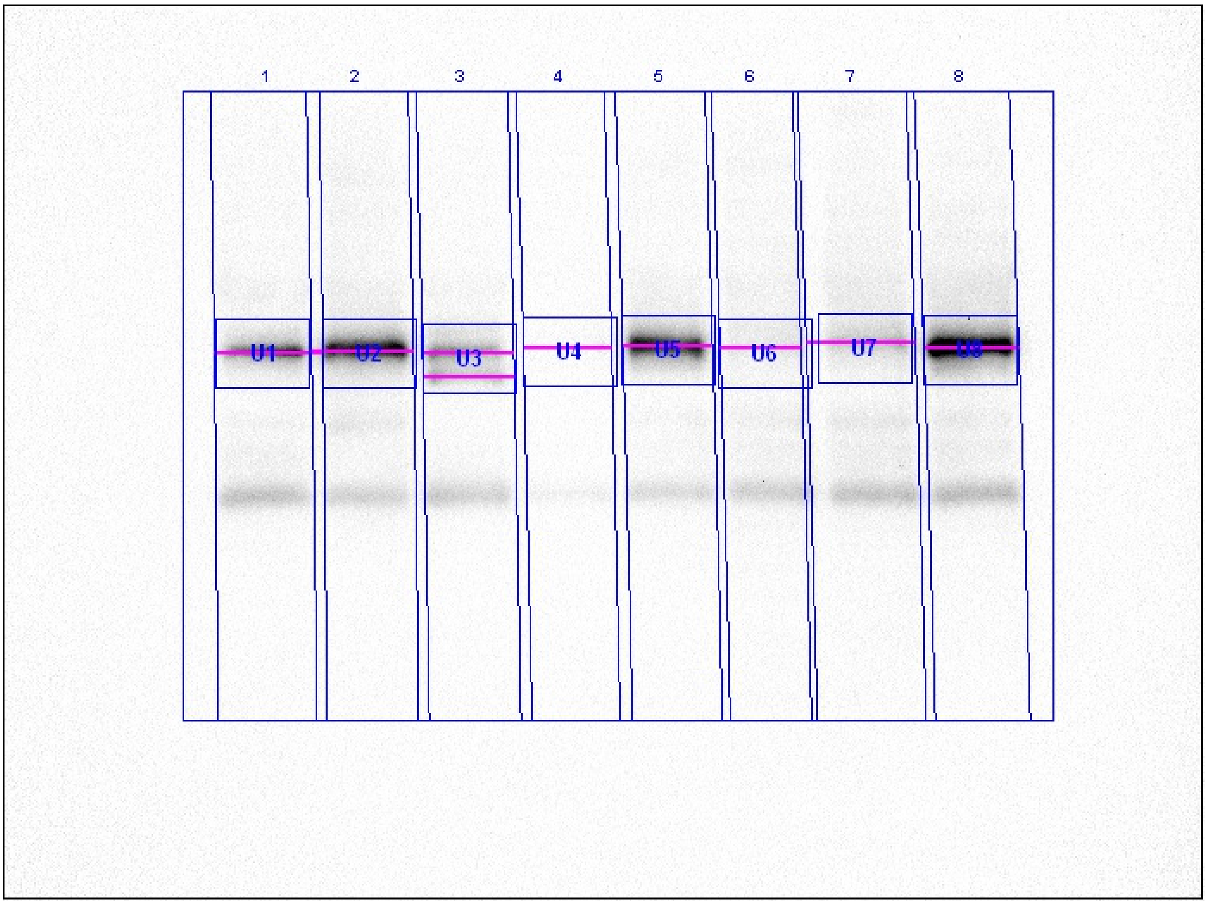

C:\Documents and Settings\Jaga\Pulpit\YKL-39 WB\analiza\powt 1\1 YKL39\1 żel 1 powt YKL39 UMED 2018-02-13 11hr 00min_Exposure_60.0sec.scn

Acquisition Information

Imager	ChemiDoc™ MP
Exposure Time (sec)	60.000 (Signal Accumulation)
Flat Field	Applied (Lens)
Serial Number	731BR01769
Software Version	5.2.1
Application	Chemi Hi Resolution
Excitation Source	No Illumination
Emission Filter	No Filter
Binning	2x2

Image Information

Acquisition Date	2018-02-13 11:01:51
User Name	UMED
Image Area (mm)	X: 97.0 Y: 72.5
Pixel Size (um)	X: 139.4 Y: 139.4
Data Range (Int)	0 - 31556

Analysis Settings

Detection	Lane detection:<br>Automatically detected lanes with manual adjustments<br><br>Band detection:<br>Bands detected with different sensitivity per lane<br>Manually adjusted bands<br><br>Lane Background Subtraction:<br>Lane background subtracted with disk size: 10<br><br>Lane width: Variable
Volume Analysis	Background subtraction method: Local<br>Quantity regression method: Linear

Lane And Band Analysis

Lane 1

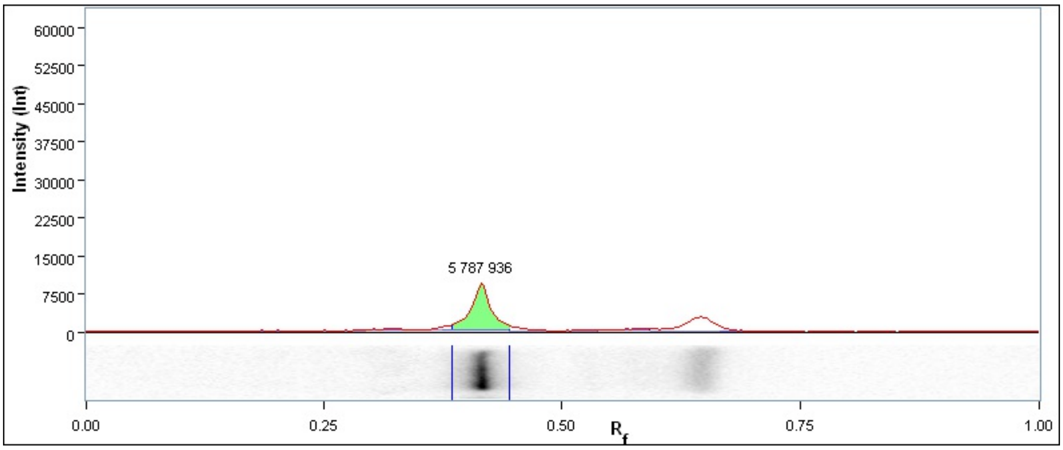

Band No.	Band Label	Mol. Wt. (KDa)	Relative Front	Volume (Int)	Abs. Quant.	Rel. Quant.	Band %	Lane %
1		N/A	0,416	5 787 936	N/A	N/A	100,0	53,8

Band Detection	Automatically detected bands with sensitivity: Low
Lane Background	Lane background subtracted with disk size: 10
Lane Width	8.78 mm

Lane 2

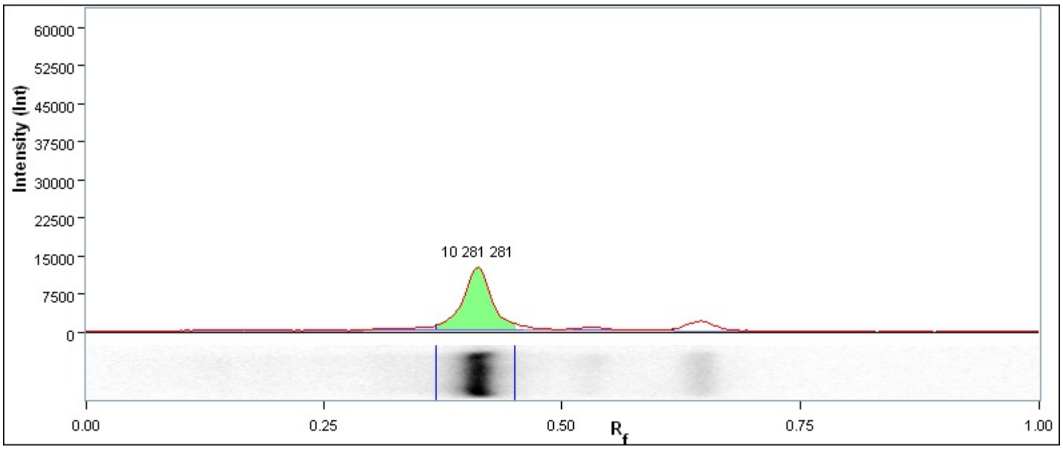

Band No.	Band Label	Mol. Wt. (KDa)	Relative Front	Volume (Int)	Abs. Quant.	Rel. Quant.	Band %	Lane %
1		N/A	0,414	10 281 281	N/A	N/A	100,0	69,8

Band Detection	Automatically detected bands with sensitivity: Low

Lane Background	Lane background subtracted with disk size: 10
Lane Width	8.22 mm

Lane 3

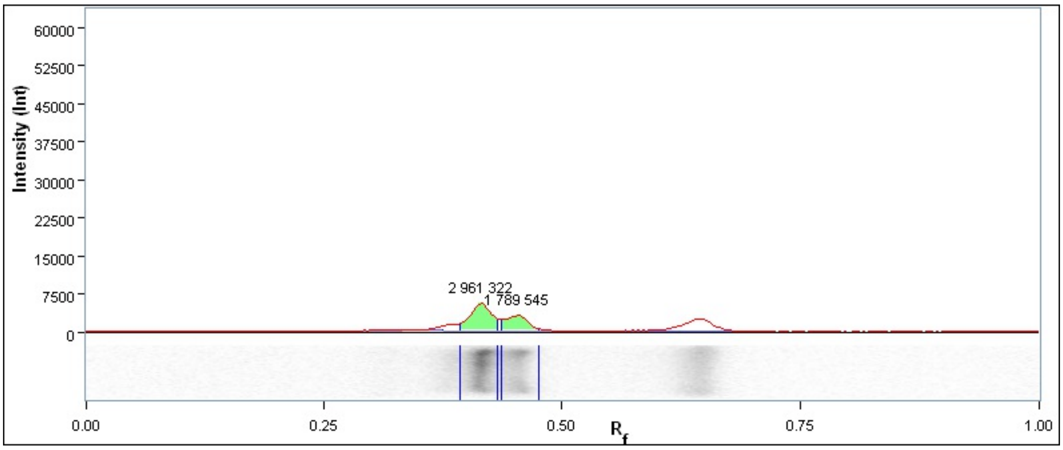

Band No.	Band Label	Mol. Wt. (KDa)	Relative Front	Volume (Int)	Abs. Quant.	Rel. Quant.	Band %	Lane %
1		N/A	0,416	2 961 322	N/A	N/A	62,3	33,9
2		N/A	0,455	1 789 545	N/A	N/A	37,7	20,5

Band Detection	Automatically detected bands with sensitivity: Low
Lane Background	Lane background subtracted with disk size: 10
Lane Width	7.39 mm

Lane 4

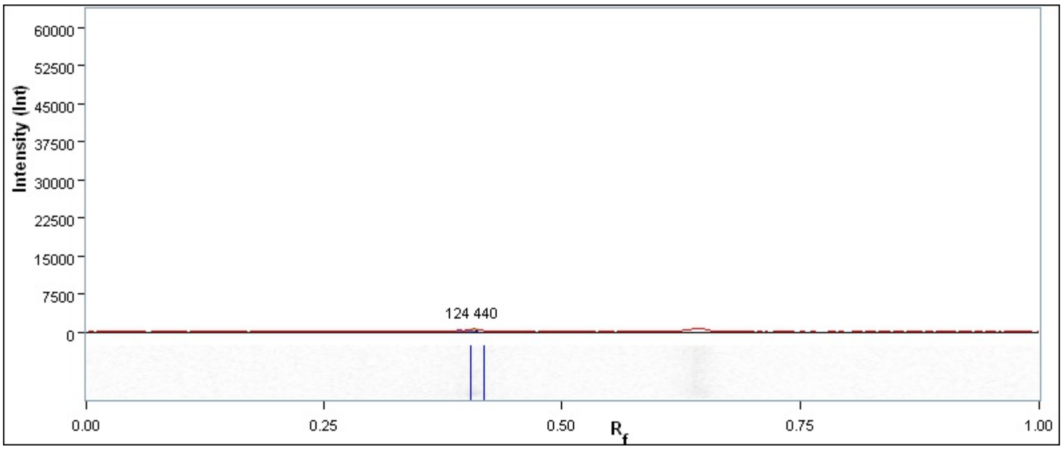

Band No.	Band Label	Mol. Wt. (KDa)	Relative Front	Volume (Int)	Abs. Quant.	Rel. Quant.	Band %	Lane %
1		N/A	0,408	124 440	N/A	N/A	100,0	7,1

Lane Background	Lane background subtracted with disk size: 10
Lane Width	7.11 mm

Lane 5

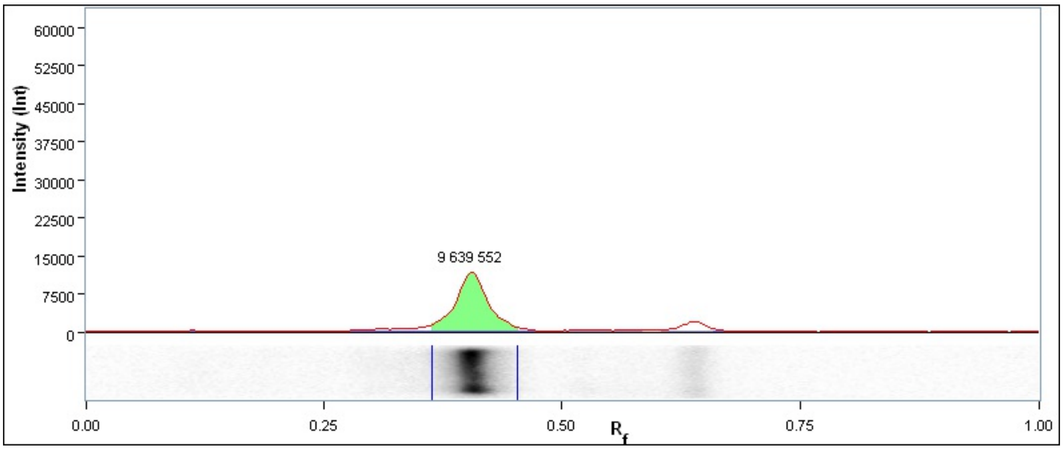

Band No.	Band Label	Mol. Wt. (KDa)	Relative Front	Volume (Int)	Abs. Quant.	Rel. Quant.	Band %	Lane %
1		N/A	0,405	9 639 552	N/A	N/A	100,0	74,8

Band Detection	Automatically detected bands with sensitivity: Low
Lane Background	Lane background subtracted with disk size: 10
Lane Width	7.25 mm

Lane 6

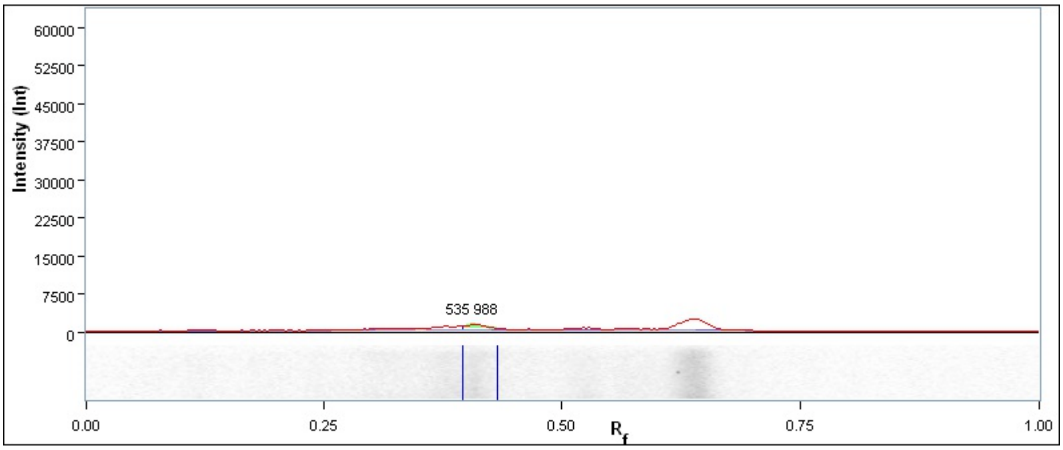

Band No.	Band Label	Mol. Wt. (KDa)	Relative Front	Volume (Int)	Abs. Quant.	Rel. Quant.	Band %	Lane %
1		N/A	0,408	535 988	N/A	N/A	100,0	13,7

Lane Background	Lane background subtracted with disk size: 10
Lane Width	6.55 mm

Lane 7

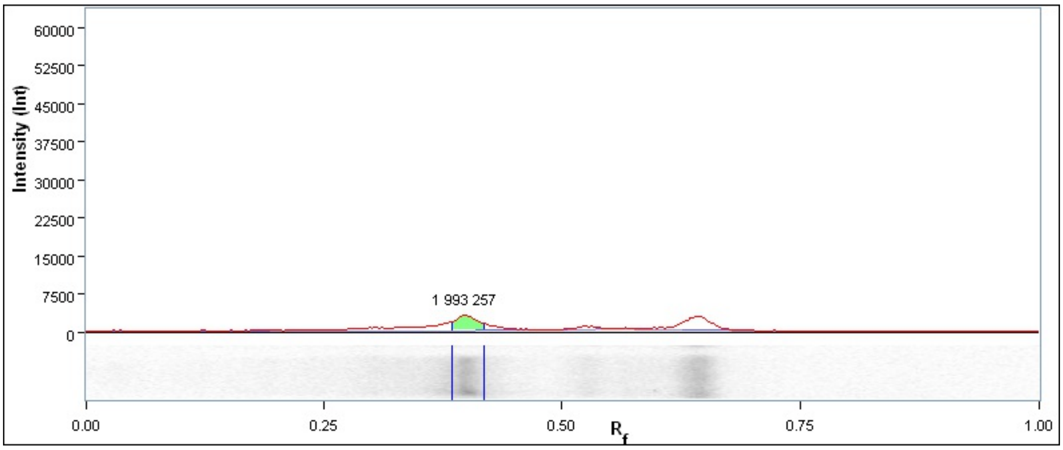

Band No.	Band Label	Mol. Wt. (KDa)	Relative Front	Volume (Int)	Abs. Quant.	Rel. Quant.	Band %	Lane %
1		N/A	0,400	1 993 257	N/A	N/A	100,0	21,1

Band Detection	Automatically detected bands with sensitivity: Low
Lane Background	Lane background subtracted with disk size: 10
Lane Width	8.78 mm

Lane 8

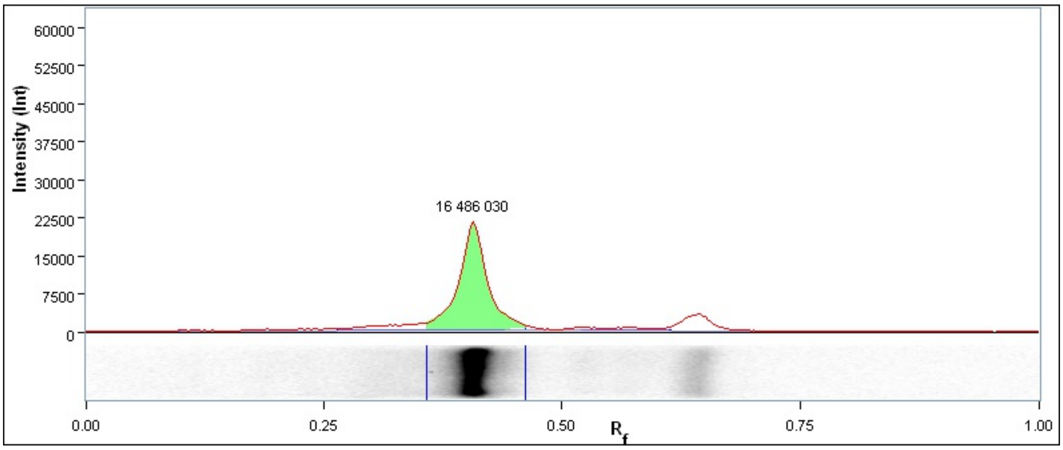

Band No.	Band Label	Mol. Wt. (KDa)	Relative Front	Volume (Int)	Abs. Quant.	Rel. Quant.	Band %	Lane %
1		N/A	0,408	16 486 030	N/A	N/A	100,0	70,6

Band Detection	Automatically detected bands with sensitivity: Low
Lane Background	Lane background subtracted with disk size: 10
Lane Width	7.67 mm

Volume Analysis

No.	Label	Type	Volume (Int)	Adj. Vol. (Int)	Mean Bkgd. (Int)	Abs. Quant.	Rel. Quant.	# of Pixels	Min. Value (Int)	Max. Value (Int)	Mean Value (Int)	Std. Dev.	Area (mm2)
1	U1	Unknown	7 117 380	5 499 270	749,1	N/A	N/A	2 160	0	18 792	3 295,1	3 588,5	42,0
2	U2	Unknown	11 621 504	8 714 279	1 345,9	N/A	N/A	2 160	0	19 276	5 380,3	4 893,8	42,0
3	U3	Unknown	6 160 516	4 447 906	792,9	N/A	N/A	2 160	0	11 200	2 852,1	1 982,5	42,0
4	U4	Unknown	756 348	136 203	287,1	N/A	N/A	2 160	0	2 212	350,2	348,2	42,0
5	U5	Unknown	10 484 952	8 792 907	783,4	N/A	N/A	2 160	0	18 576	4 854,1	4 501,6	42,0
6	U6	Unknown	2 358 272	958 952	647,8	N/A	N/A	2 160	0	3 296	1 091,8	589,9	42,0
7	U7	Unknown	4 284 864	2 318 319	910,4	N/A	N/A	2 160	0	7 616	1 983,7	1 285,3	42,0
8	U8	Unknown	17 656 584	13 993 809	1 695,7	N/A	N/A	2 160	0	31 556	8 174,3	7 453,5	42,0

Image Report: 1 żel 2 powt tub UMED 2018-02-21 10hr 54min_Exposure_16.8sec

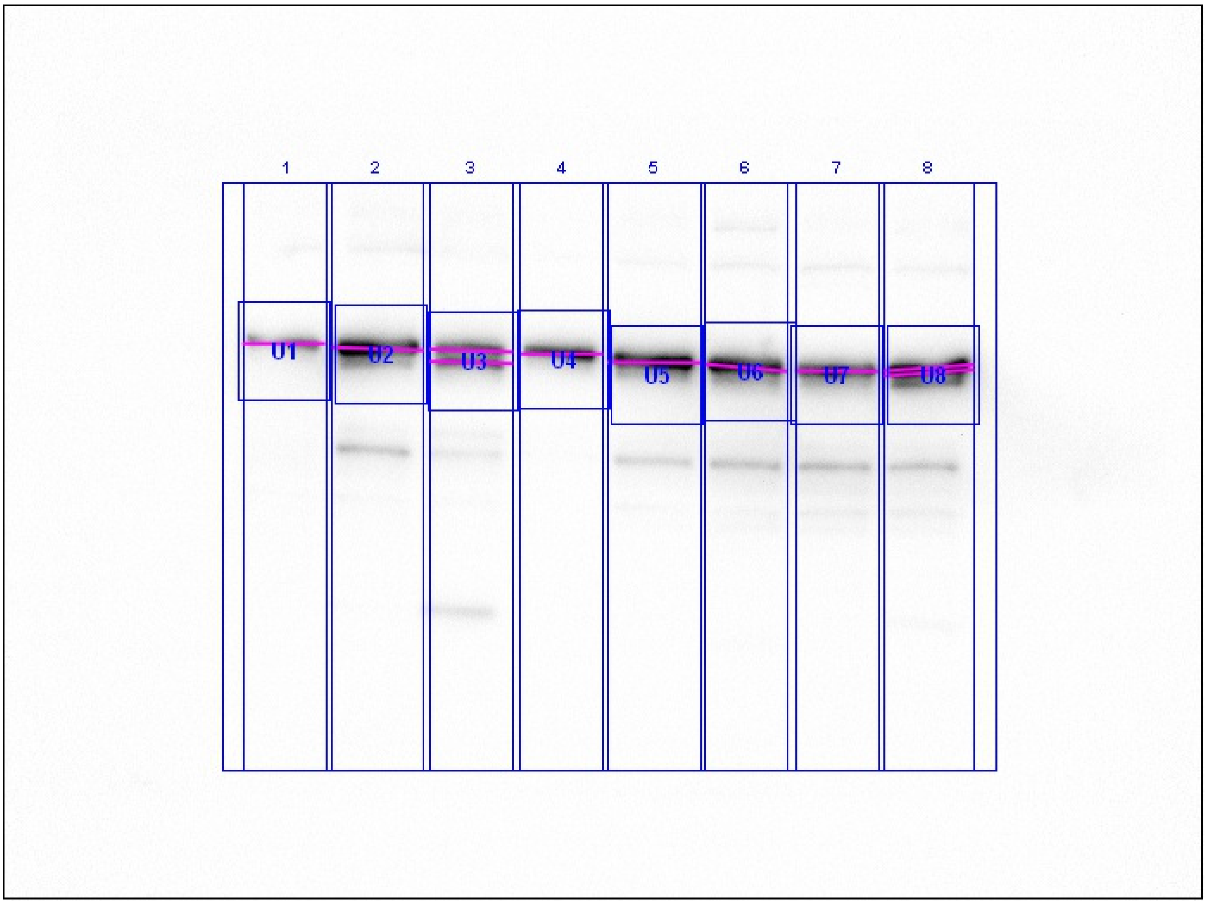

C:\Documents and Settings\Jaga\Pulpit\YKL-39 WB\analiza\żel 1\1 tub\1 żel 2 powt tub UMED 2018-02-21 10hr 54min_Exposure_16.8sec.scn

Acquisition Information

Imager	ChemiDoc™ MP
Exposure Time (sec)	16.800 (Signal Accumulation)
Flat Field	Applied (Lens)
Serial Number	731BR01769
Software Version	5.2.1
Application	Chemi Hi Resolution
Excitation Source	No Illumination
Emission Filter	No Filter
Binning	2x2

Image Information

Acquisition Date	2018-02-21 10:55:16
User Name	UMED
Image Area (mm)	X: 103.0 Y: 77.0
Pixel Size (um)	X: 148.0 Y: 148.0
Data Range (Int)	0 - 41216

Analysis Settings

Detection	Lane detection:<br>Manually created lanes<br><br>Band detection:<br>Automatically detected bands with sensitivity: Low<br>Manually adjusted bands<br><br>Lane Background Subtraction:<br>Lane background subtracted with disk size: 10<br><br>Lane width: Variable
Volume Analysis	Background subtraction method: Local<br>Quantity regression method: Linear

Lane And Band Analysis

Lane 1

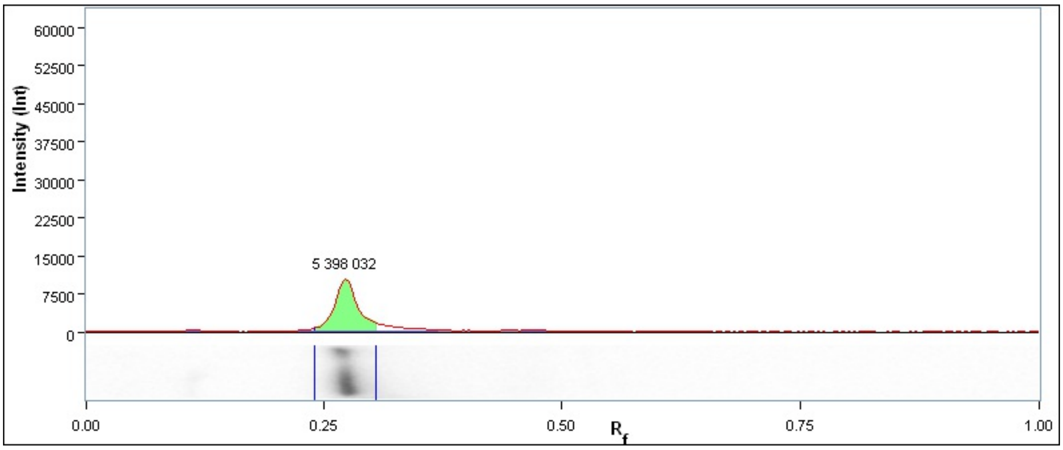

Band No.	Band Label	Mol. Wt. (KDa)	Relative Front	Volume (Int)	Abs. Quant.	Rel. Quant.	Band %	Lane %
1		N/A	0,275	5 398 032	N/A	N/A	100,0	73,4

Band Detection	Automatically detected bands with sensitivity: Low
Lane Background	Lane background subtracted with disk size: 10
Lane Width	7.10 mm

Lane 2

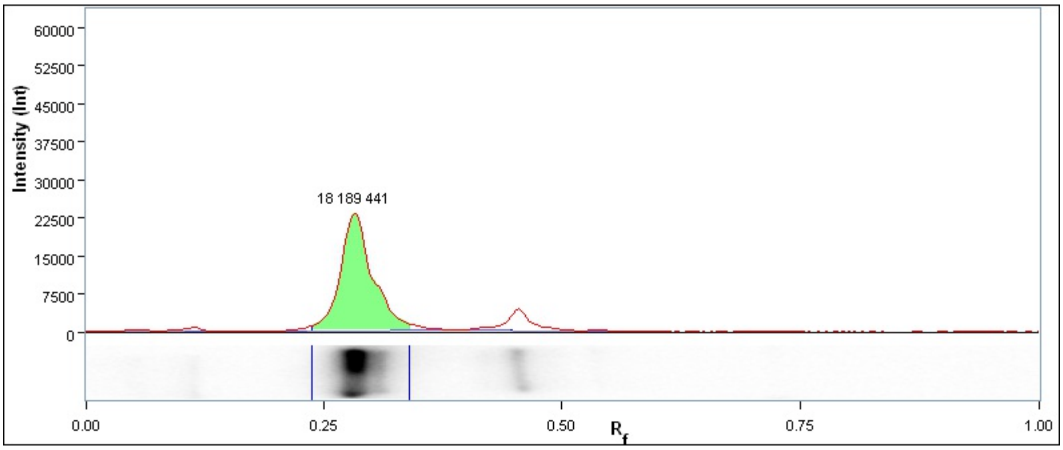

Band No.	Band Label	Mol. Wt. (KDa)	Relative Front	Volume (Int)	Abs. Quant.	Rel. Quant.	Band %	Lane %
1		N/A	0,284	18 189 441	N/A	N/A	100,0	80,2

Band Detection	Automatically detected bands with sensitivity: Low

Lane Background	Lane background subtracted with disk size: 10
Lane Width	7.84 mm

Lane 3

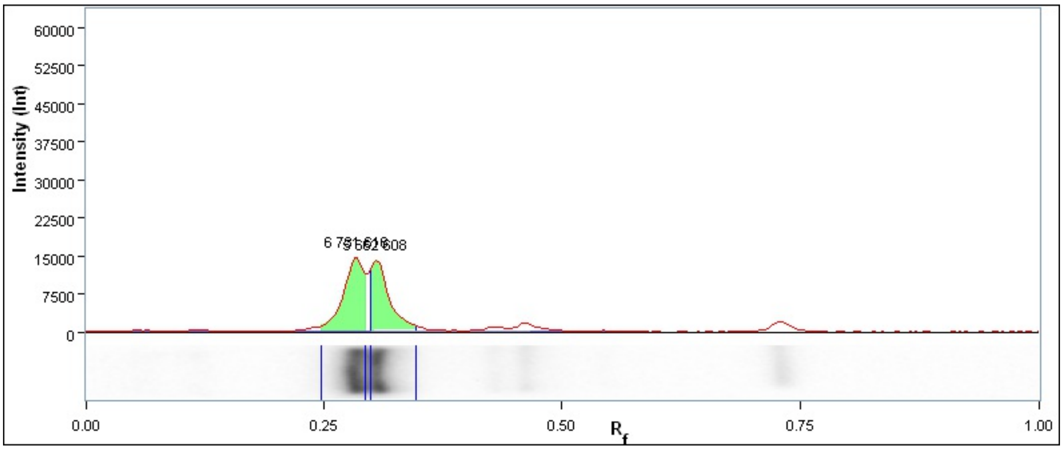

Band No.	Band Label	Mol. Wt. (KDa)	Relative Front	Volume (Int)	Abs. Quant.	Rel. Quant.	Band %	Lane %
1		N/A	0,287	6 731 616	N/A	N/A	54,3	41,3
2		N/A	0,307	5 662 608	N/A	N/A	45,7	34,7

Band Detection	Automatically detected bands with sensitivity: Low
Lane Background	Lane background subtracted with disk size: 10
Lane Width	7.10 mm

Lane 4

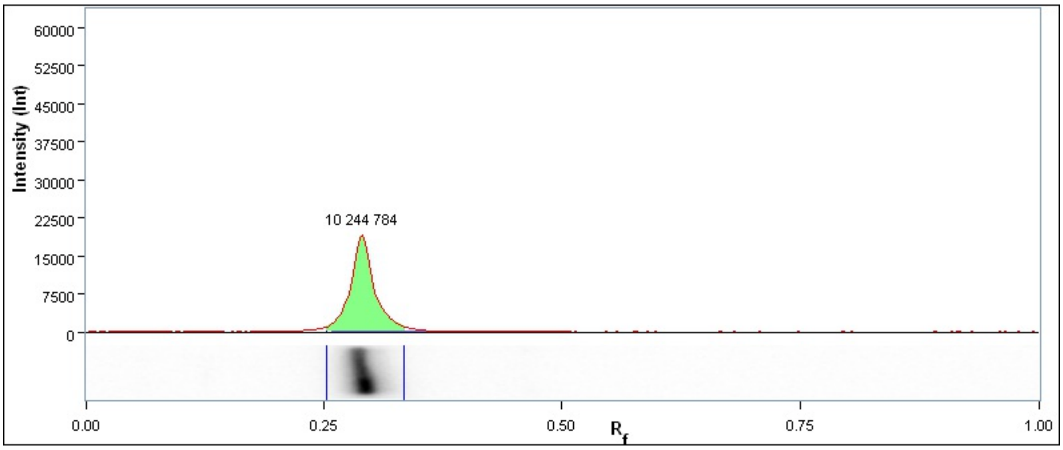

Band No.	Band Label	Mol. Wt. (KDa)	Relative Front	Volume (Int)	Abs. Quant.	Rel. Quant.	Band %	Lane %
1		N/A	0,292	10 244 784	N/A	N/A	100,0	89,9

Band Detection	Automatically detected bands with sensitivity: Low
Lane Background	Lane background subtracted with disk size: 10
Lane Width	7.10 mm

Lane 5

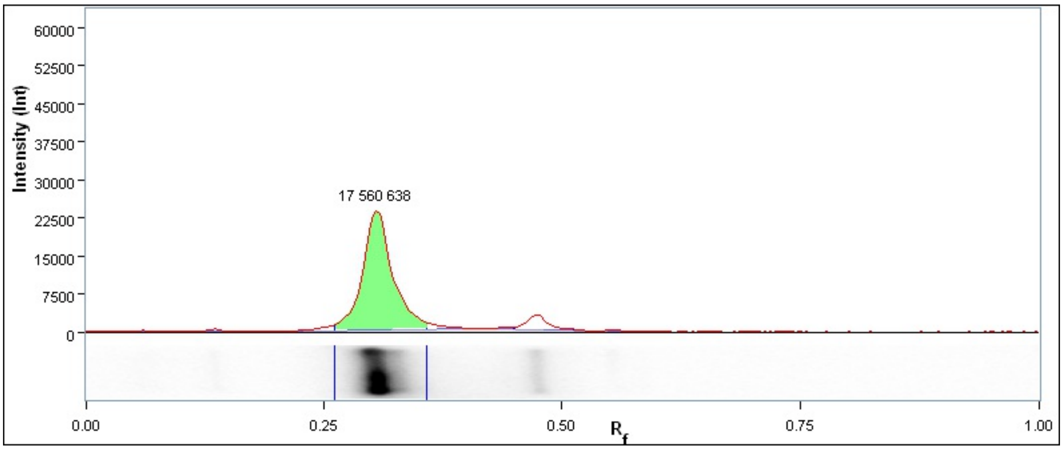

Band No.	Band Label	Mol. Wt. (KDa)	Relative Front	Volume (Int)	Abs. Quant.	Rel. Quant.	Band %	Lane %
1		N/A	0,307	17 560 638	N/A	N/A	100,0	84,0

Band Detection	Automatically detected bands with sensitivity: Low
Lane Background	Lane background subtracted with disk size: 10
Lane Width	7.99 mm

Lane 6

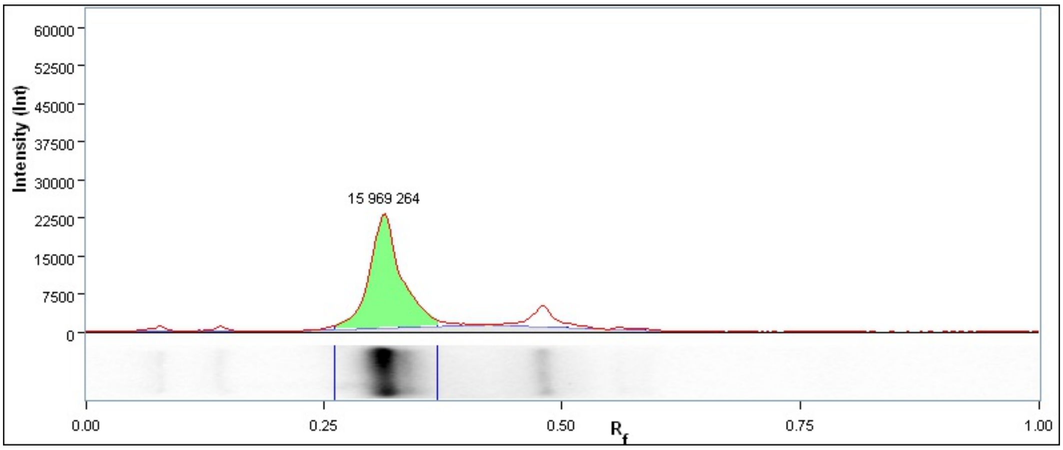

Band No.	Band Label	Mol. Wt. (KDa)	Relative Front	Volume (Int)	Abs. Quant.	Rel. Quant.	Band %	Lane %
1		N/A	0,316	15 969 264	N/A	N/A	100,0	78,4

Band Detection	Automatically detected bands with sensitivity: Low
Lane Background	Lane background subtracted with disk size: 10
Lane Width	7.10 mm

Lane 7

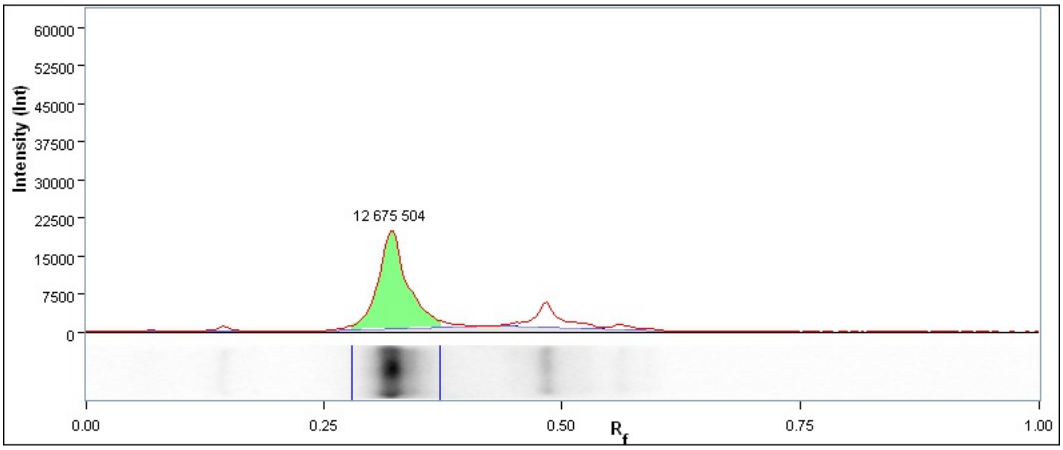

Band No.	Band Label	Mol. Wt. (KDa)	Relative Front	Volume (Int)	Abs. Quant.	Rel. Quant.	Band %	Lane %
1		N/A	0,322	12 675 504	N/A	N/A	100,0	72,0

Band Detection	Automatically detected bands with sensitivity: Low
Lane Background	Lane background subtracted with disk size: 10
Lane Width	7.10 mm

Lane 8

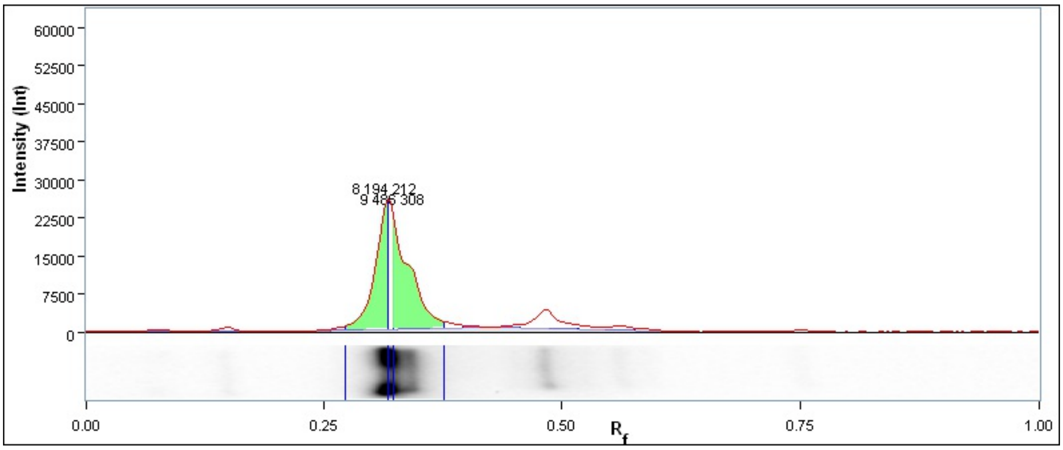

Band No.	Band Label	Mol. Wt. (KDa)	Relative Front	Volume (Int)	Abs. Quant.	Rel. Quant.	Band %	Lane %
1		N/A	0,316	8 194 212	N/A	N/A	46,3	34,2
2		N/A	0,325	9 486 308	N/A	N/A	53,7	39,6

Band Detection	Automatically detected bands with sensitivity: Low
Lane Background	Lane background subtracted with disk size: 10
Lane Width	7.70 mm

Volume Analysis

No.	Label	Type	Volume (Int)	Adj. Vol. (Int)	Mean Bkgd. (Int)	Abs. Quant.	Rel. Quant.	# of Pixels	Min. Value (Int)	Max. Value (Int)	Mean Value (Int)	Std. Dev.	Area (mm2)
1	U1	Unknown	7 336 200	4 203 585	1 036,9	N/A	N/A	3 021	0	18 048	2 428,4	3 260,5	66,2
2	U2	Unknown	19 774 280	15 038 539	1 567,6	N/A	N/A	3 021	0	41 216	6 545,6	8 340,8	66,2
3	U3	Unknown	14 582 612	11 654 670	969,2	N/A	N/A	3 021	0	21 848	4 827,1	5 821,7	66,2
4	U4	Unknown	11 593 196	8 635 205	979,1	N/A	N/A	3 021	0	31 424	3 837,5	6 081,5	66,2
5	U5	Unknown	20 025 580	14 690 386	1 766,0	N/A	N/A	3 021	0	39 784	6 628,8	8 283,0	66,2
6	U6	Unknown	19 812 108	13 316 796	2 150,1	N/A	N/A	3 021	0	36 400	6 558,1	7 368,5	66,2
7	U7	Unknown	16 161 816	10 355 076	1 922,1	N/A	N/A	3 021	0	29 476	5 349,8	6 130,2	66,2

8	U8	Unknown	21 916 604	16 726 364	1 718,1	N/A	N/A	3 021	0	40 996	7 254,8	8 877,6	66,2

Image Report: 1 żel 2 powt YKL-39

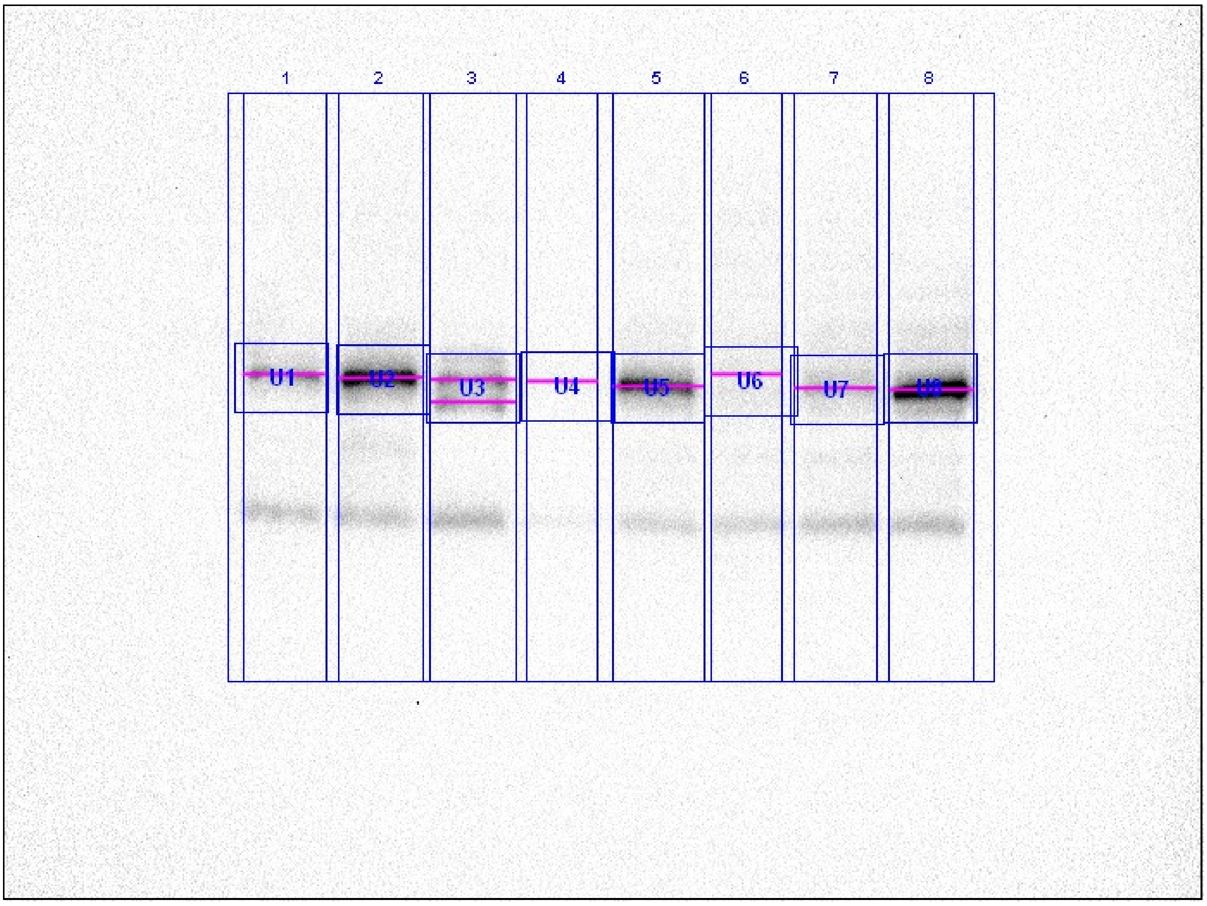

C:\Documents and Settings\Jaga\Pulpit\YKL-39 WB\analiza\powt 1\1 YKL39\1 żel 2 powt YKL-39.scn

Acquisition Information

Imager	ChemiDoc™ MP
Exposure Time (sec)	60.000 (Signal Accumulation)
Flat Field	Applied (Lens)
Serial Number	731BR01769
Software Version	5.2.1
Application	Chemi Hi Resolution
Excitation Source	No Illumination
Emission Filter	No Filter
Binning	2x2

Image Information

Acquisition Date	2018-02-20 10:51:52
User Name	UMED
Image Area (mm)	X: 103.0 Y: 77.0
Pixel Size (um)	X: 148.0 Y: 148.0
Data Range (Int)	0 - 36544

Analysis Settings

Detection	Lane detection:<br>Manually created lanes

	Band detection:<br>Automatically detected bands with sensitivity: Low<br>Manually adjusted bands<br><br>Lane Background Subtraction:<br>Lane background subtracted with disk size: 10<br><br>Lane width: Variable
Volume Analysis	Background subtraction method: Local<br>Quantity regression method: Linear

Lane And Band Analysis

Lane 1

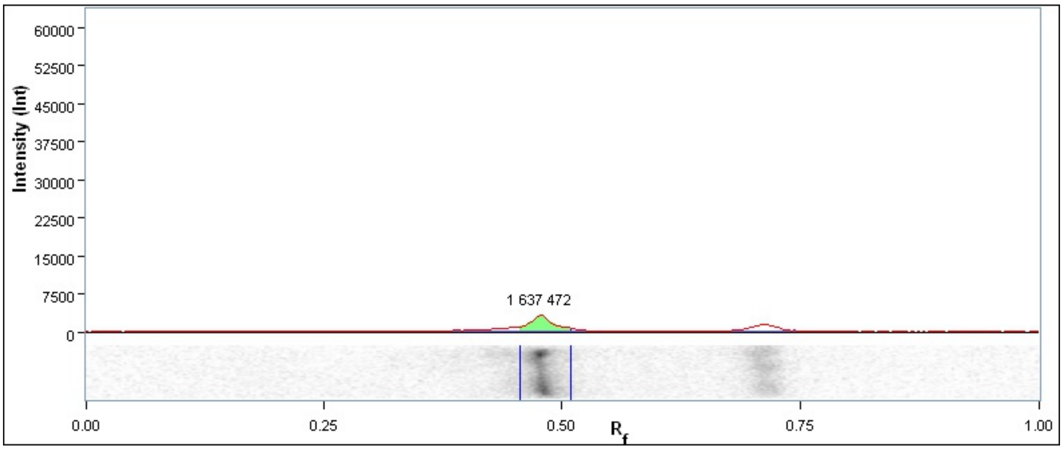

Band No.	Band Label	Mol. Wt. (KDa)	Relative Front	Volume (Int)	Abs. Quant.	Rel. Quant.	Band %	Lane %
1		N/A	0,478	1 637 472	N/A	N/A	100,0	40,0

Band Detection	Automatically detected bands with sensitivity: Low
Lane Background	Lane background subtracted with disk size: 10
Lane Width	7.10 mm

Lane 2

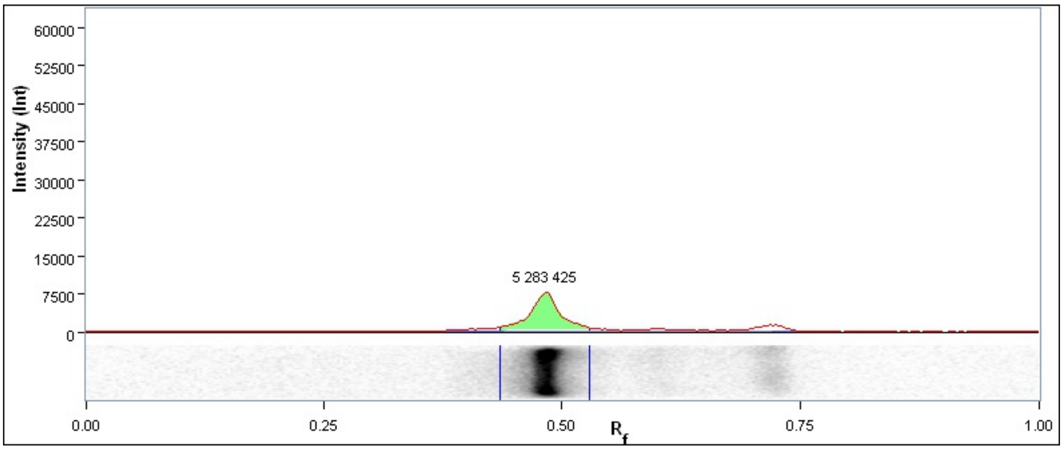

Band No.	Band Label	Mol. Wt. (KDa)	Relative Front	Volume (Int)	Abs. Quant.	Rel. Quant.	Band %	Lane %
1		N/A	0,484	5 283 425	N/A	N/A	100,0	68,1

Band Detection	Automatically detected bands with sensitivity: Low
Lane Background	Lane background subtracted with disk size: 10
Lane Width	7.25 mm

Lane 3

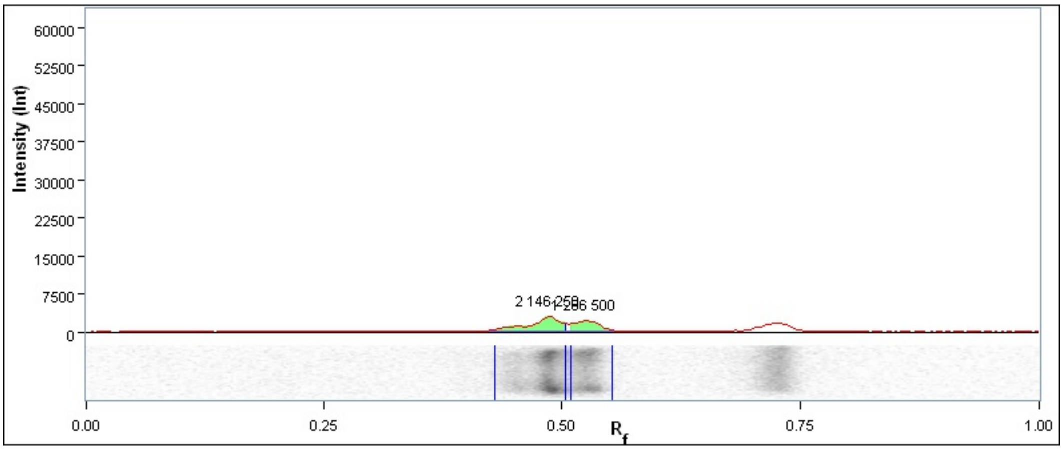

Band No.	Band Label	Mol. Wt. (KDa)	Relative Front	Volume (Int)	Abs. Quant.	Rel. Quant.	Band %	Lane %
1		N/A	0,487	2 146 250	N/A	N/A	62,9	36,9
2		N/A	0,525	1 266 500	N/A	N/A	37,1	21,8

Band Detection	Automatically detected bands with sensitivity: Low
Lane Background	Lane background subtracted with disk size: 10
Lane Width	7.40 mm

Lane 4

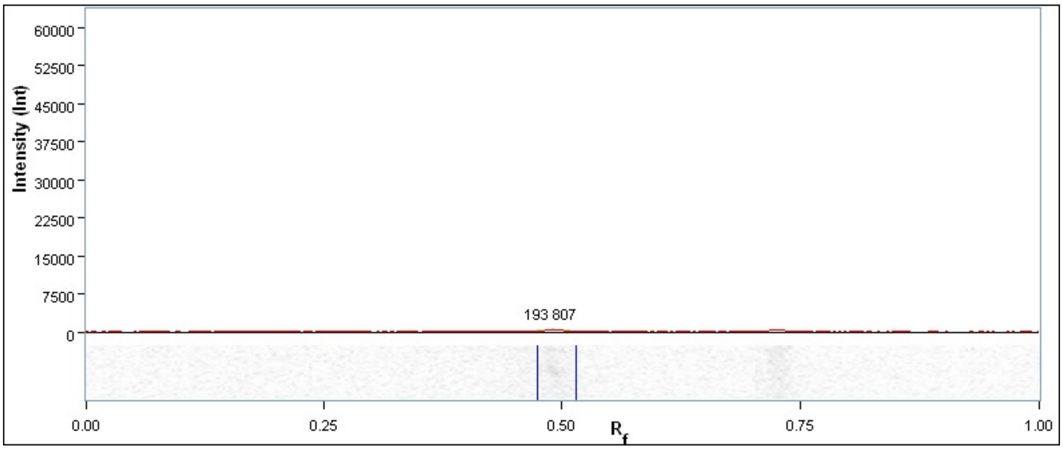

Band No.	Band Label	Mol. Wt. (KDa)	Relative Front	Volume (Int)	Abs. Quant.	Rel. Quant.	Band %	Lane %
1		N/A	0,490	193 807	N/A	N/A	100,0	15,0

Band Detection	Automatically detected bands with sensitivity: Low
Lane Background	Lane background subtracted with disk size: 10
Lane Width	6.07 mm

Lane 5

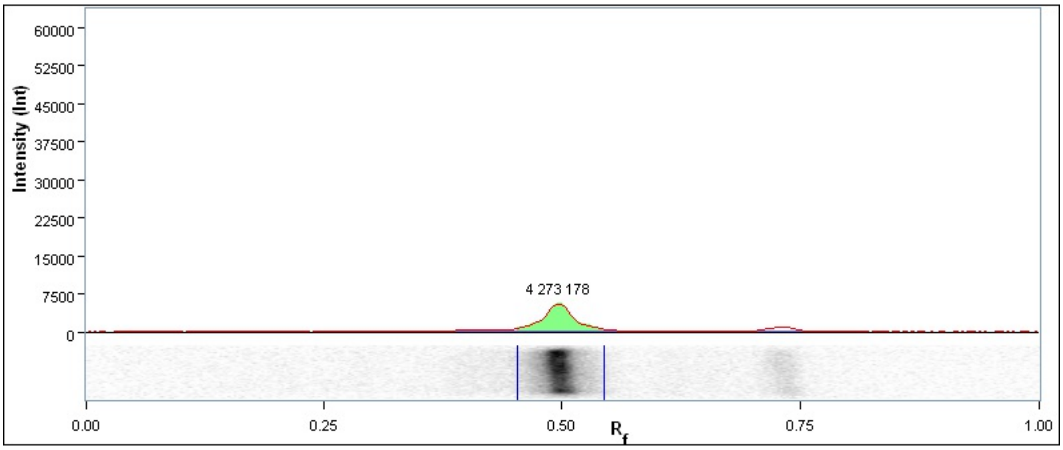

Band No.	Band Label	Mol. Wt. (KDa)	Relative Front	Volume (Int)	Abs. Quant.	Rel. Quant.	Band %	Lane %
1		N/A	0,499	4 273 178	N/A	N/A	100,0	67,6

Band Detection	Automatically detected bands with sensitivity: Low
Lane Background	Lane background subtracted with disk size: 10
Lane Width	7.84 mm

Lane 6

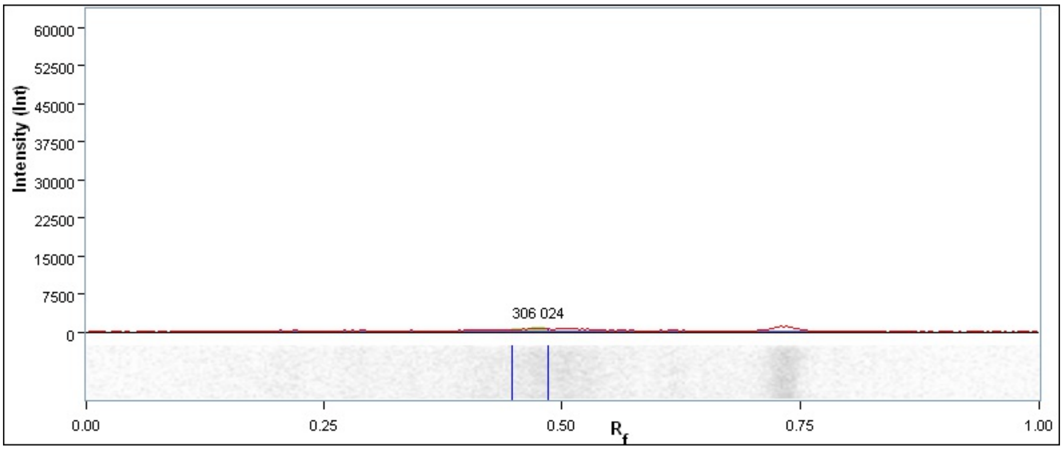

Band No.	Band Label	Mol. Wt. (KDa)	Relative Front	Volume (Int)	Abs. Quant.	Rel. Quant.	Band %	Lane %
1		N/A	0,478	306 024	N/A	N/A	100,0	12,4

Band Detection	Automatically detected bands with sensitivity: Low
Lane Background	Lane background subtracted with disk size: 10
Lane Width	6.07 mm

Lane 7

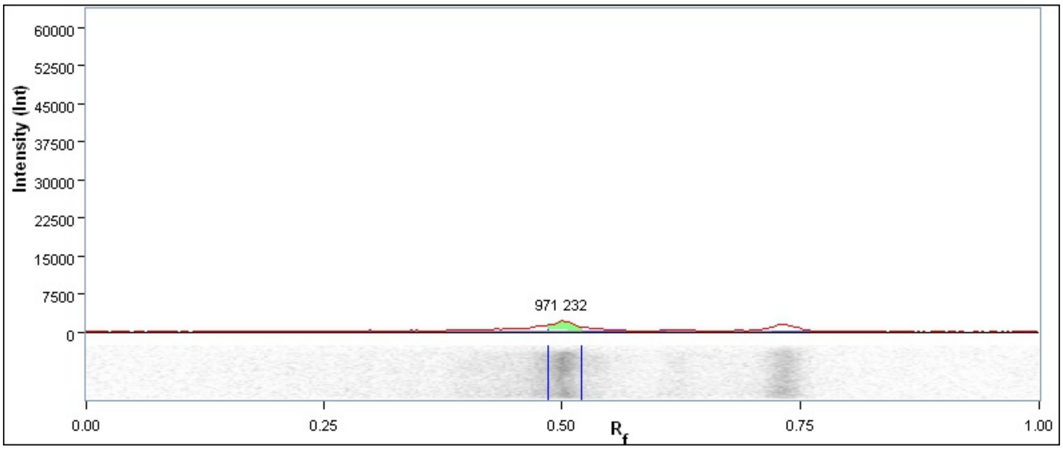

Band No.	Band Label	Mol. Wt. (KDa)	Relative Front	Volume (Int)	Abs. Quant.	Rel. Quant.	Band %	Lane %
1		N/A	0,501	971 232	N/A	N/A	100,0	25,8

Band Detection	Automatically detected bands with sensitivity: Low
Lane Background	Lane background subtracted with disk size: 10
Lane Width	7.10 mm

Lane 8

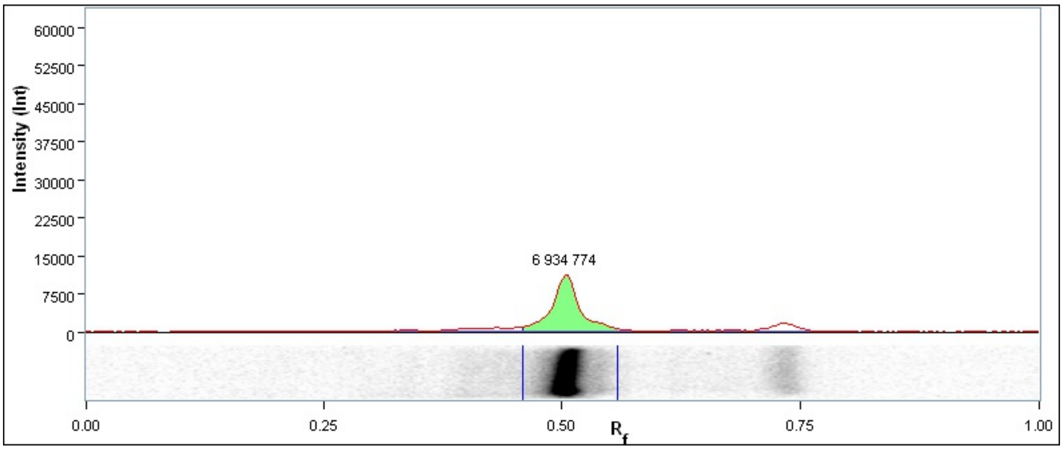

Band No.	Band Label	Mol. Wt. (KDa)	Relative Front	Volume (Int)	Abs. Quant.	Rel. Quant.	Band %	Lane %
1		N/A	0,504	6 934 774	N/A	N/A	100,0	67,4

Band Detection	Automatically detected bands with sensitivity: Low
Lane Background	Lane background subtracted with disk size: 10
Lane Width	7.25 mm

Volume Analysis

No.	Label	Type	Volume (Int)	Adj. Vol. (Int)	Mean Bkgd. (Int)	Abs. Quant.	Rel. Quant.	# of Pixels	Min. Value (Int)	Max. Value (Int)	Mean Value (Int)	Std. Dev.	Area (mm2)
1	U1	Unknown	2 654 244	1 819 224	386,6	N/A	N/A	2 160	0	7 628	1 228,8	1 121,9	47,3
2	U2	Unknown	6 239 580	4 654 320	733,9	N/A	N/A	2 160	0	12 864	2 888,7	2 650,2	47,3
3	U3	Unknown	3 737 504	2 500 499	572,7	N/A	N/A	2 160	0	5 820	1 730,3	1 129,9	47,3
4	U4	Unknown	663 332	-67 828	338,5	N/A	N/A	2 160	0	1 696	307,1	316,2	47,3
5	U5	Unknown	4 888 684	3 976 309	422,4	N/A	N/A	2 160	0	9 092	2 263,3	2 069,3	47,3
6	U6	Unknown	1 389 776	483 431	419,6	N/A	N/A	2 160	0	1 924	643,4	412,8	47,3
7	U7	Unknown	2 445 720	1 334 175	514,6	N/A	N/A	2 160	0	3 944	1 132,3	768,0	47,3
8	U8	Unknown	7 747 948	6 280 723	679,3	N/A	N/A	2 160	0	19 652	3 587,0	3 889,5	47,3

Image Report: 1 żel 3 powt tub UMED 2018-01-26 11hr 11min_Exposure_16.8sec

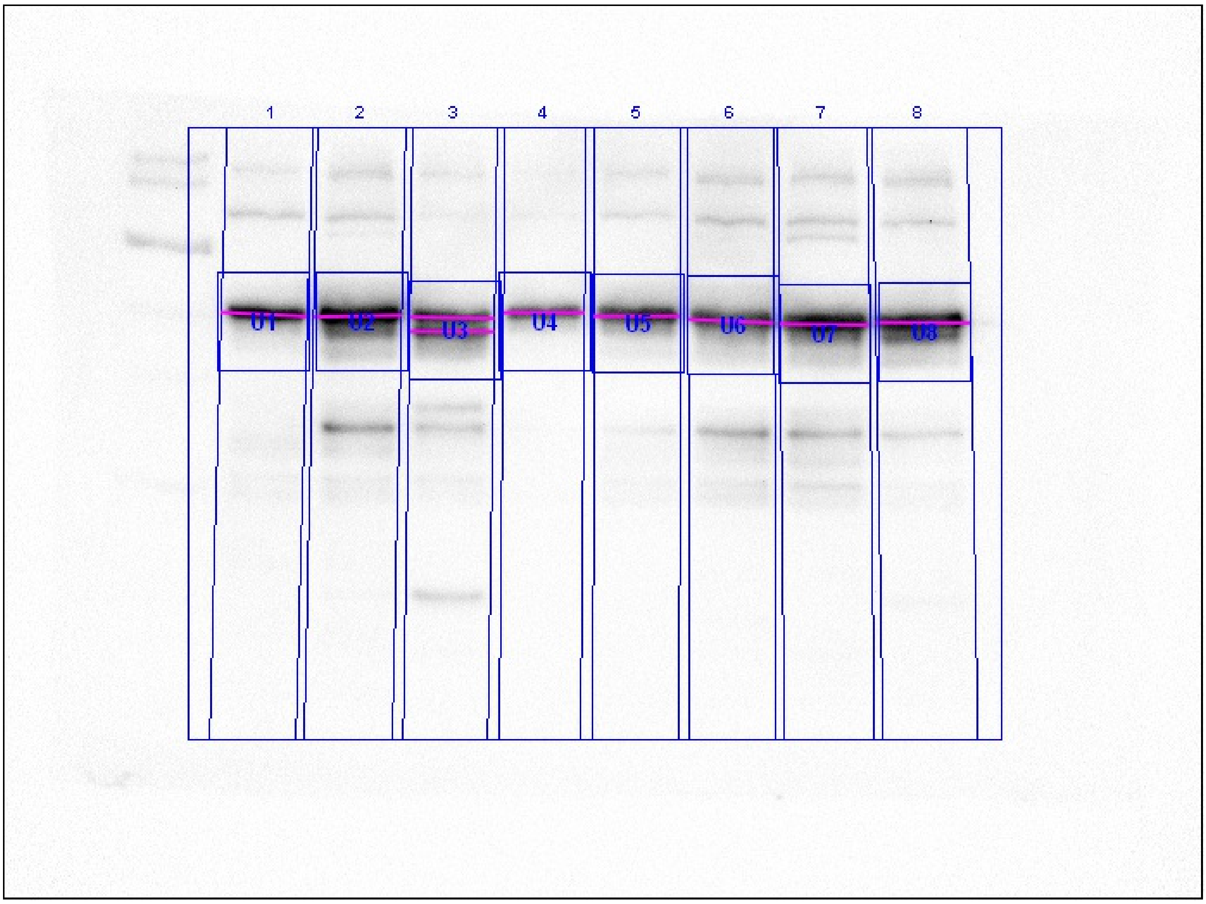

C:\Documents and Settings\Jaga\Pulpit\YKL-39 WB\analiza\żel 1\1 tub\1 żel 3 powt tub UMED 2018-01-26 11hr 11min_Exposure_16.8sec.scn

Acquisition Information

Imager	ChemiDoc™ MP
Exposure Time (sec)	16.800 (Signal Accumulation)
Flat Field	Applied (Lens)
Serial Number	731BR01769
Software Version	5.2.1
Application	Chemi Hi Resolution
Excitation Source	No Illumination
Emission Filter	No Filter
Binning	2x2

Image Information

Acquisition Date	2018-01-26 11:11:58
User Name	UMED
Image Area (mm)	X: 103.0 Y: 77.0
Pixel Size (um)	X: 148.0 Y: 148.0
Data Range (Int)	0 - 24324

Analysis Settings

Detection	Lane detection:<br>Automatically detected lanes with manual adjustments<br><br>Band detection:<br>Automatically detected bands with sensitivity: Low<br>Manually adjusted bands<br><br>Lane Background Subtraction:<br>Lane background subtracted with disk size: 10<br><br>Lane width: Variable
Volume Analysis	Background subtraction method: Local<br>Quantity regression method: Linear

Lane And Band Analysis

Lane 1

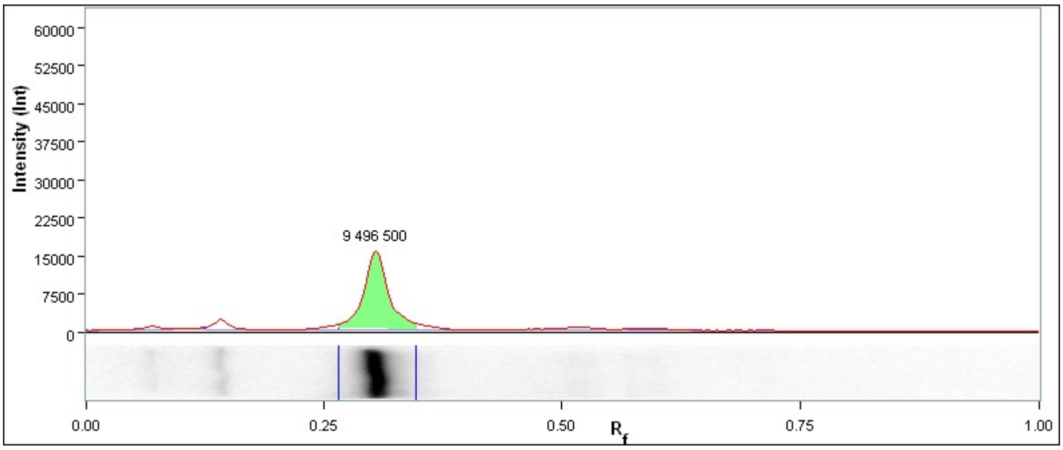

Band No.	Band Label	Mol. Wt. (KDa)	Relative Front	Volume (Int)	Abs. Quant.	Rel. Quant.	Band %	Lane %
1		N/A	0,306	9 496 500	N/A	N/A	100,0	75,1

Band Detection	Automatically detected bands with sensitivity: Low
Lane Background	Lane background subtracted with disk size: 10
Lane Width	7.40 mm

Lane 2

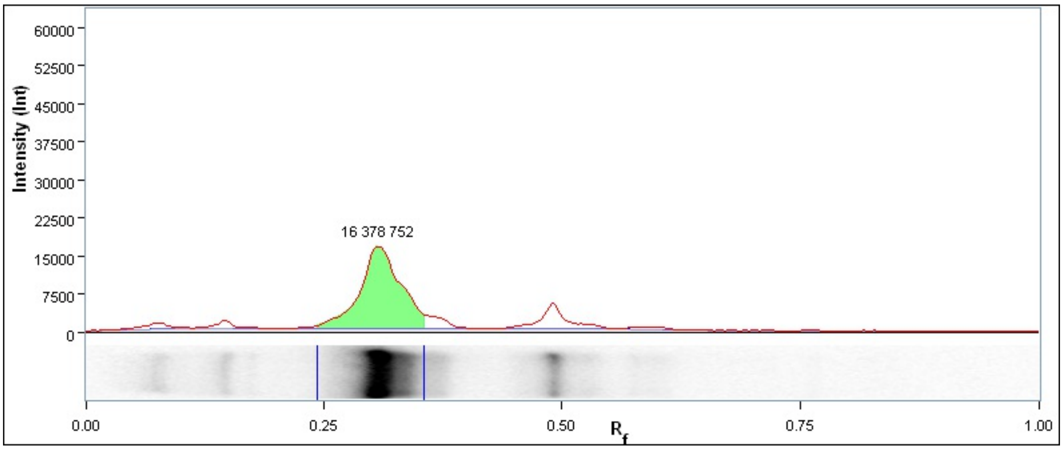

Band No.	Band Label	Mol. Wt. (KDa)	Relative Front	Volume (Int)	Abs. Quant.	Rel. Quant.	Band %	Lane %
1		N/A	0,309	16 378 752	N/A	N/A	100,0	69,7

Band Detection	Automatically detected bands with sensitivity: Low

Lane Background	Lane background subtracted with disk size: 10
Lane Width	7.55 mm

Lane 3

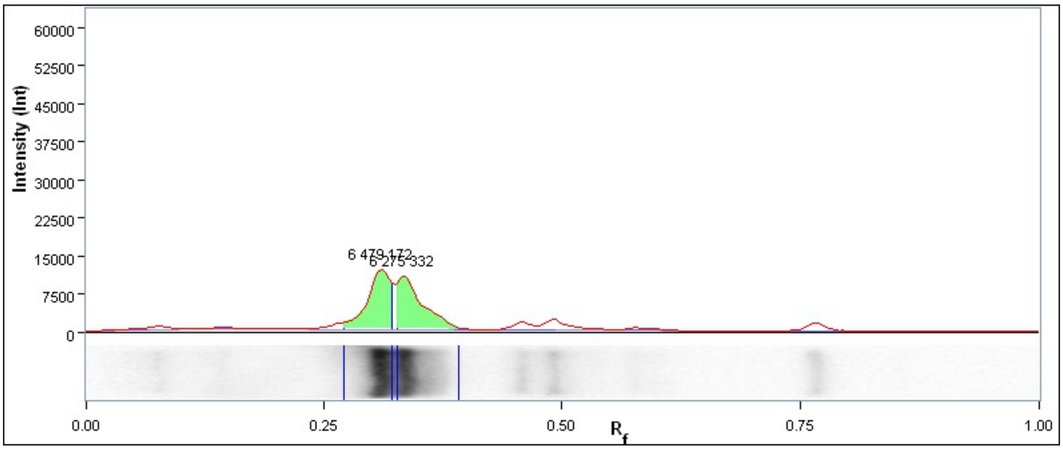

Band No.	Band Label	Mol. Wt. (KDa)	Relative Front	Volume (Int)	Abs. Quant.	Rel. Quant.	Band %	Lane %
1		N/A	0,312	6 479 172	N/A	N/A	50,8	36,3
2		N/A	0,334	6 275 332	N/A	N/A	49,2	35,1

Band Detection	Automatically detected bands with sensitivity: Low
Lane Background	Lane background subtracted with disk size: 10
Lane Width	7.25 mm

Lane 4

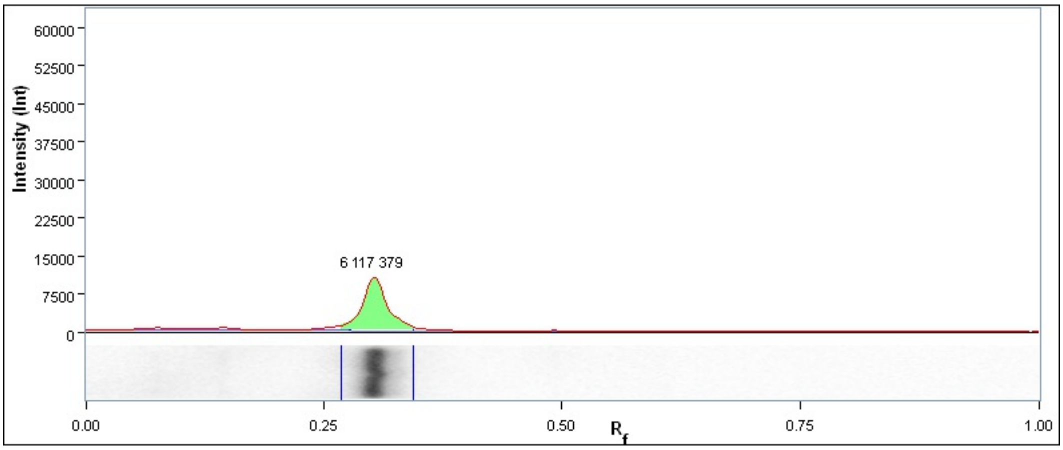

Band No.	Band Label	Mol. Wt. (KDa)	Relative Front	Volume (Int)	Abs. Quant.	Rel. Quant.	Band %	Lane %
1		N/A	0,303	6 117 379	N/A	N/A	100,0	76,1

Band Detection	Automatically detected bands with sensitivity: Low
Lane Background	Lane background subtracted with disk size: 10
Lane Width	6.96 mm

Lane 5

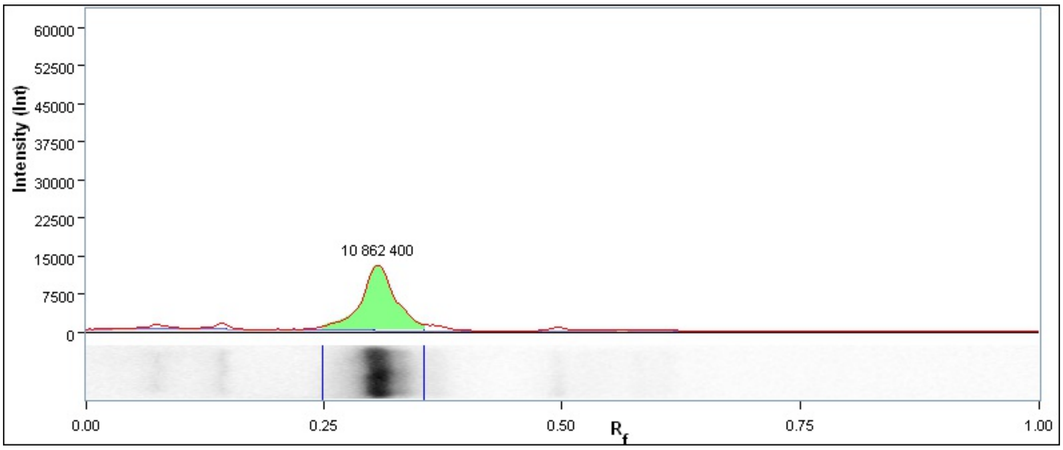

Band No.	Band Label	Mol. Wt. (KDa)	Relative Front	Volume (Int)	Abs. Quant.	Rel. Quant.	Band %	Lane %
1		N/A	0,309	10 862 400	N/A	N/A	100,0	78,9

Band Detection	Automatically detected bands with sensitivity: Low
Lane Background	Lane background subtracted with disk size: 10
Lane Width	7.40 mm

Lane 6

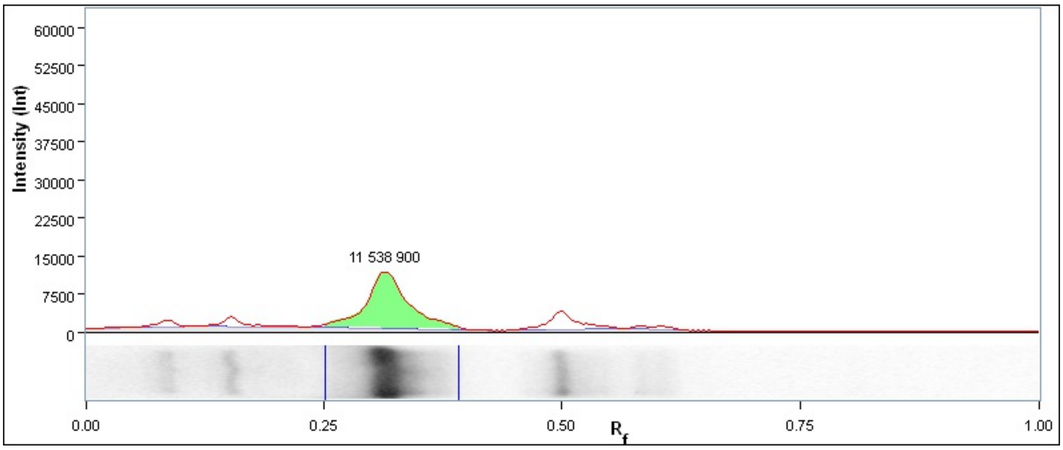

Band No.	Band Label	Mol. Wt. (KDa)	Relative Front	Volume (Int)	Abs. Quant.	Rel. Quant.	Band %	Lane %
1		N/A	0,317	11 538 900	N/A	N/A	100,0	69,1

Band Detection	Automatically detected bands with sensitivity: Low
Lane Background	Lane background subtracted with disk size: 10
Lane Width	7.40 mm

Lane 7

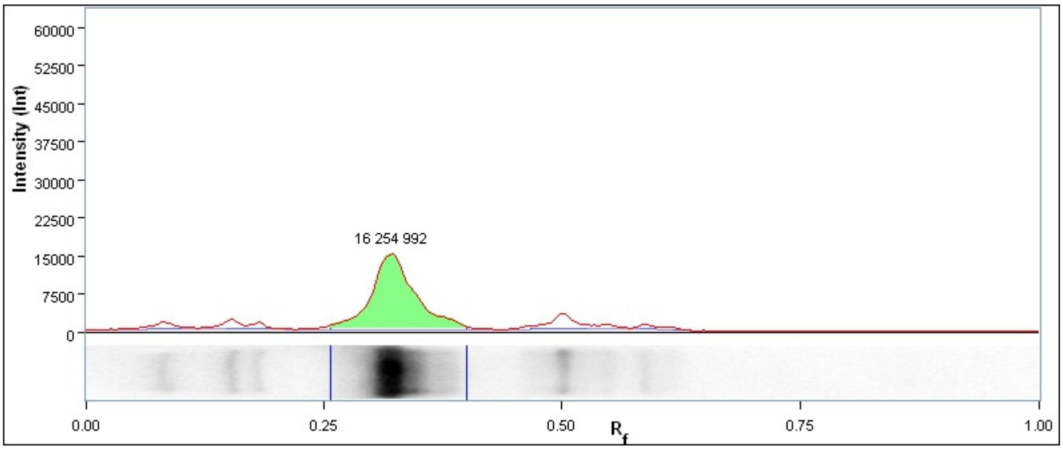

Band No.	Band Label	Mol. Wt. (KDa)	Relative Front	Volume (Int)	Abs. Quant.	Rel. Quant.	Band %	Lane %
1		N/A	0,323	16 254 992	N/A	N/A	100,0	74,5

Band Detection	Automatically detected bands with sensitivity: Low
Lane Background	Lane background subtracted with disk size: 10
Lane Width	7.70 mm

Lane 8

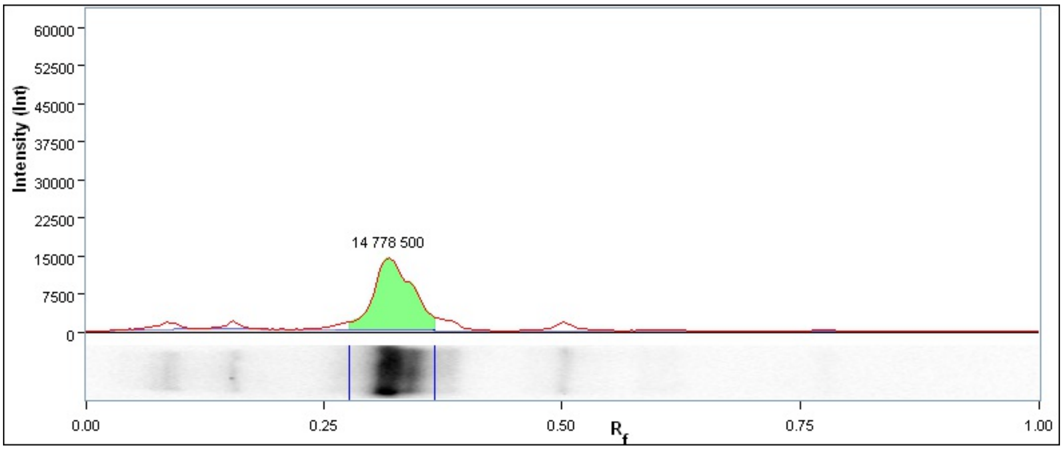

Band No.	Band Label	Mol. Wt. (KDa)	Relative Front	Volume (Int)	Abs. Quant.	Rel. Quant.	Band %	Lane %
1		N/A	0,320	14 778 500	N/A	N/A	100,0	72,5

Band Detection	Automatically detected bands with sensitivity: Low
Lane Background	Lane background subtracted with disk size: 10
Lane Width	8.14 mm

Volume Analysis

No.	Label	Type	Volume (Int)	Adj. Vol. (Int)	Mean Bkgd. (Int)	Abs. Quant.	Rel. Quant.	# of Pixels	Min. Value (Int)	Max. Value (Int)	Mean Value (Int)	Std. Dev.	Area (mm2)
1	U1	Unknown	12 122 632	8 420 504	1 225,5	N/A	N/A	3 021	0	21 836	4 012,8	5 013,8	66,2
2	U2	Unknown	20 399 608	13 797 752	2 185,3	N/A	N/A	3 021	316	24 324	6 752,6	5 661,6	66,2
3	U3	Unknown	15 698 924	10 918 839	1 582,3	N/A	N/A	3 021	0	17 156	5 196,6	4 508,8	66,2
4	U4	Unknown	8 169 820	5 187 068	987,3	N/A	N/A	3 021	0	14 092	2 704,3	3 238,1	66,2
5	U5	Unknown	13 050 412	8 911 426	1 370,1	N/A	N/A	3 021	0	18 156	4 319,9	4 324,7	66,2
6	U6	Unknown	14 590 820	8 347 222	2 066,7	N/A	N/A	3 021	192	15 952	4 829,8	3 830,5	66,2
7	U7	Unknown	18 480 568	11 949 274	2 162,0	N/A	N/A	3 021	112	21 108	6 117,4	5 267,6	66,2
8	U8	Unknown	17 594 088	12 535 585	1 674,4	N/A	N/A	3 021	0	21 648	5 823,9	5 280,0	66,2

Image Report: 1 żel 3 powt YKL39 UMED 2018-01-24 11hr 40min_Exposure_60.0sec

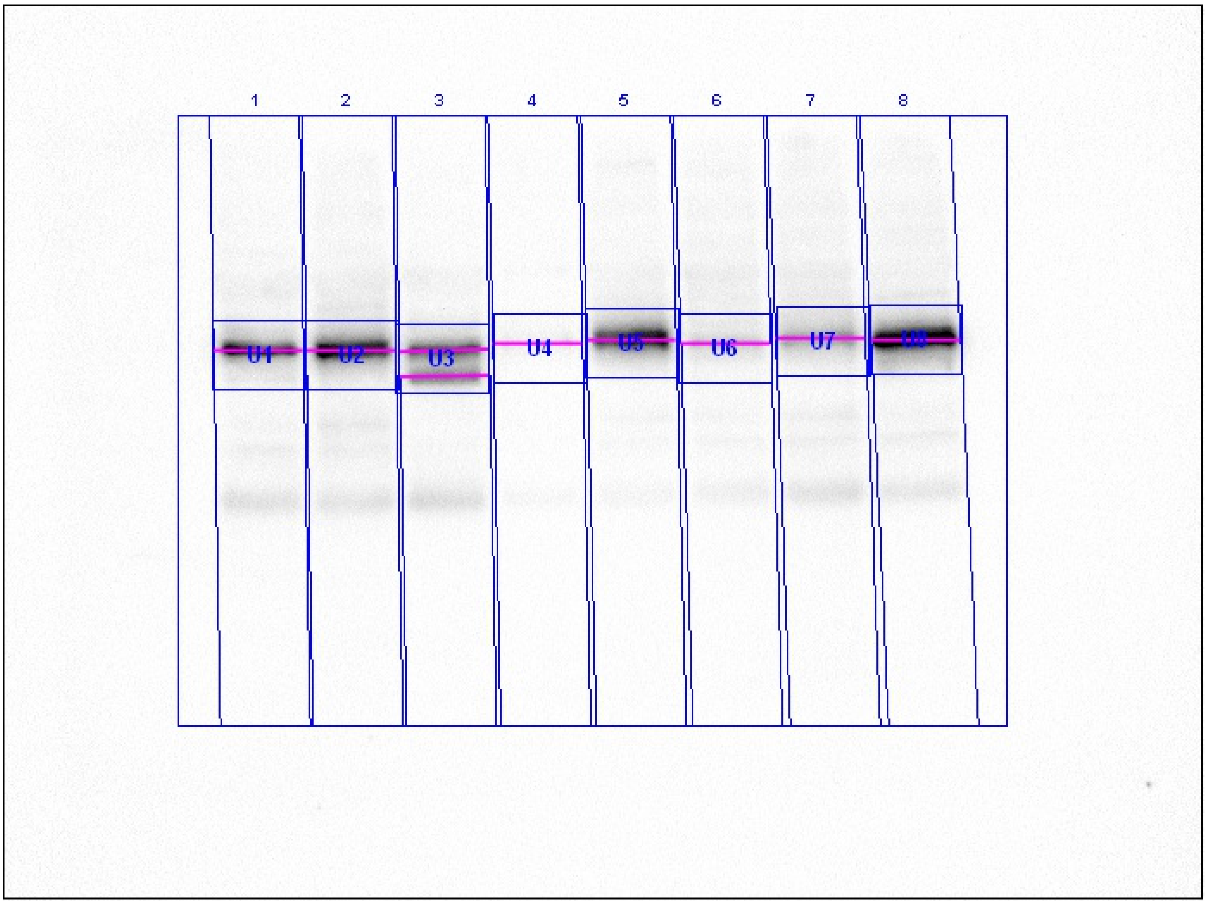

C:\Documents and Settings\Jaga\Pulpit\YKL-39 WB\analiza\powt 1\1 YKL39\1 żel 3 powt YKL39 UMED 2018-01-24 11hr 40min_Exposure_60.0sec.scn

Acquisition Information

Imager	ChemiDoc™ MP
Exposure Time (sec)	60.000 (Signal Accumulation)
Flat Field	Applied (Lens)
Serial Number	731BR01769
Software Version	5.2.1
Application	Chemi Hi Resolution
Excitation Source	No Illumination
Emission Filter	No Filter
Binning	2x2

Image Information

Acquisition Date	2018-01-24 11:41:09
User Name	UMED
Image Area (mm)	X: 102.0 Y: 76.2
Pixel Size (um)	X: 146.6 Y: 146.6
Data Range (Int)	0 - 52820

Analysis Settings

Detection	Lane detection:<br>Automatically detected lanes with manual adjustments<br><br>Band detection:<br>Automatically detected bands with sensitivity: Low<br>Manually adjusted bands<br><br>Lane Background Subtraction:<br>Lane background subtracted with disk size: 10<br><br>Lane width: 7.62 mm
Volume Analysis	Background subtraction method: Local<br>Quantity regression method: Linear

Lane And Band Analysis

Lane 1

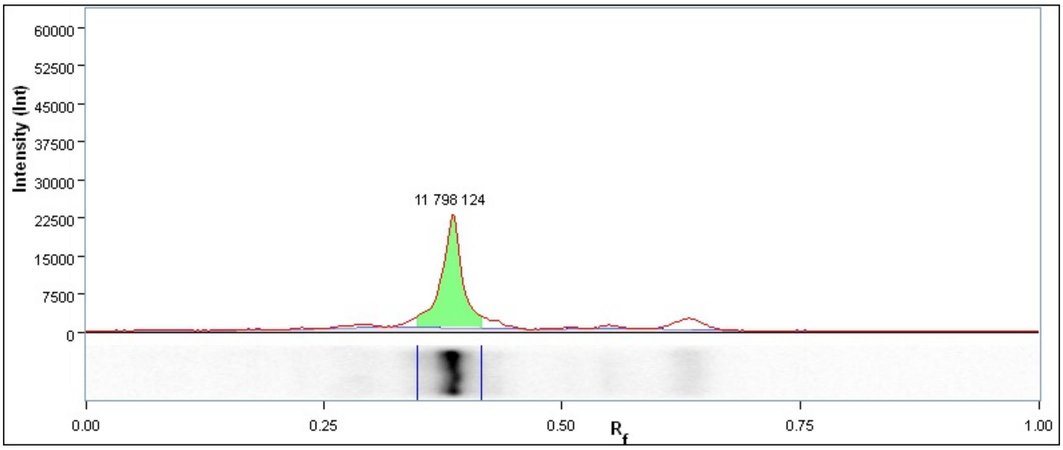

Band No.	Band Label	Mol. Wt. (KDa)	Relative Front	Volume (Int)	Abs. Quant.	Rel. Quant.	Band %	Lane %
1		N/A	0,386	11 798 124	N/A	N/A	100,0	68,7

Band Detection	Automatically detected bands with sensitivity: Low
Lane Background	Lane background subtracted with disk size: 10
Lane Width	7.62 mm

Lane 2

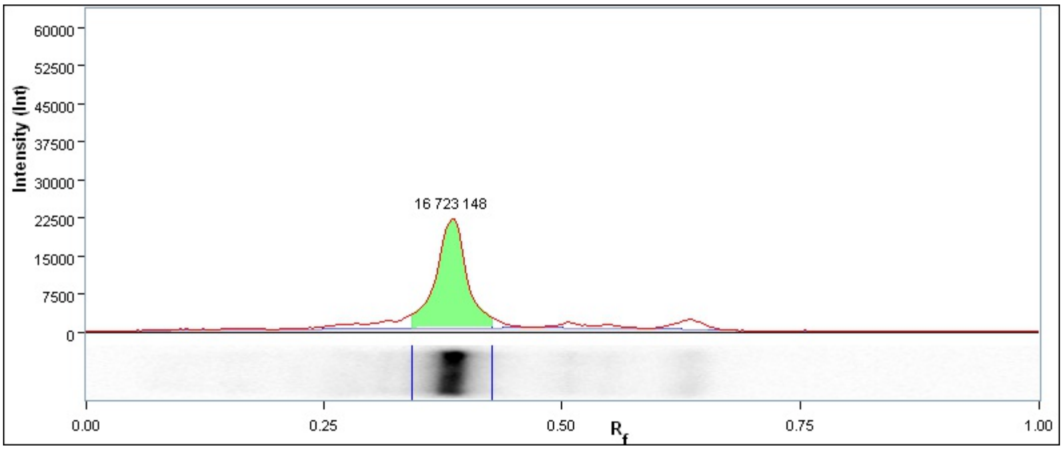

Band No.	Band Label	Mol. Wt. (KDa)	Relative Front	Volume (Int)	Abs. Quant.	Rel. Quant.	Band %	Lane %
1		N/A	0,386	16 723 148	N/A	N/A	100,0	72,4

Band Detection	Automatically detected bands with sensitivity: Low

Lane Background	Lane background subtracted with disk size: 10
Lane Width	7.62 mm

Lane 3

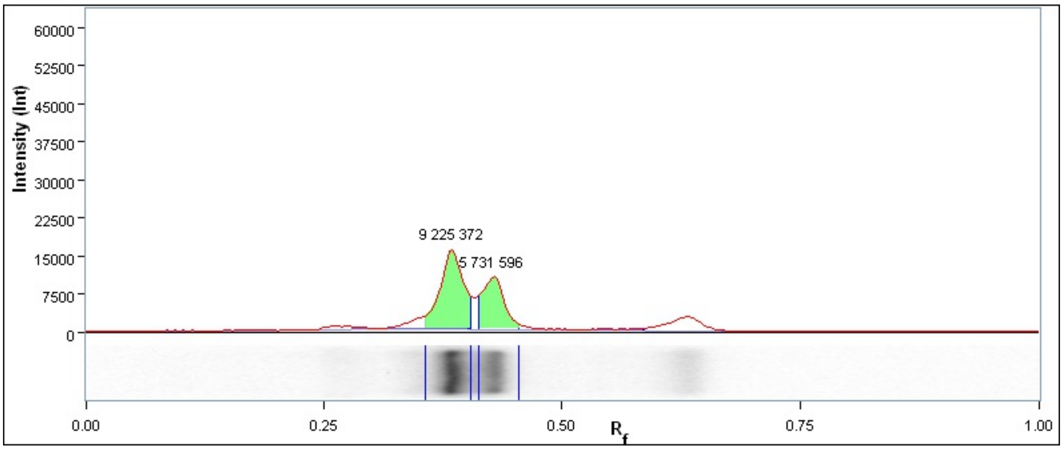

Band No.	Band Label	Mol. Wt. (KDa)	Relative Front	Volume (Int)	Abs. Quant.	Rel. Quant.	Band %	Lane %
1		N/A	0,386	9 225 372	N/A	N/A	61,7	44,5
2		N/A	0,428	5 731 596	N/A	N/A	38,3	27,6

Band Detection	Automatically detected bands with sensitivity: Low
Lane Background	Lane background subtracted with disk size: 10
Lane Width	7.62 mm

Lane 4

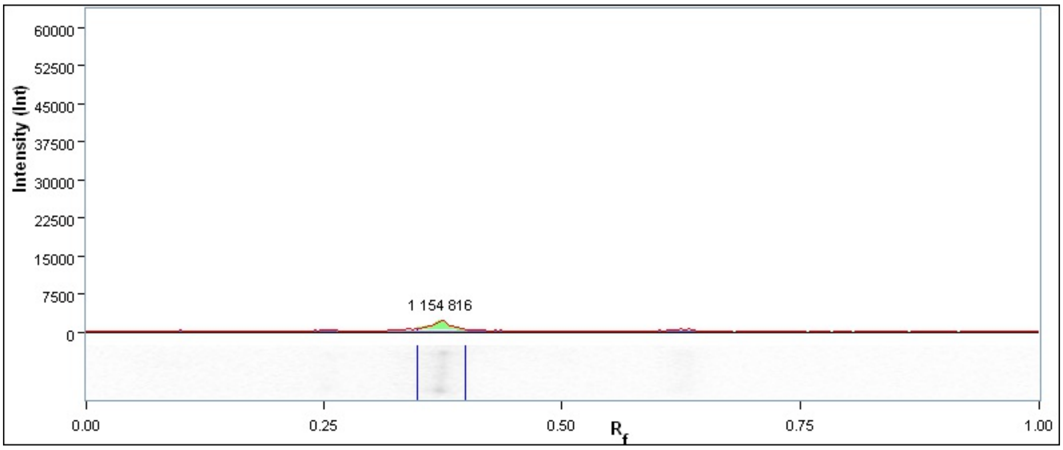

Band No.	Band Label	Mol. Wt. (KDa)	Relative Front	Volume (Int)	Abs. Quant.	Rel. Quant.	Band %	Lane %
1		N/A	0,375	1 154 816	N/A	N/A	100,0	38,5

Band Detection	Automatically detected bands with sensitivity: Low
Lane Background	Lane background subtracted with disk size: 10
Lane Width	7.62 mm

Lane 5

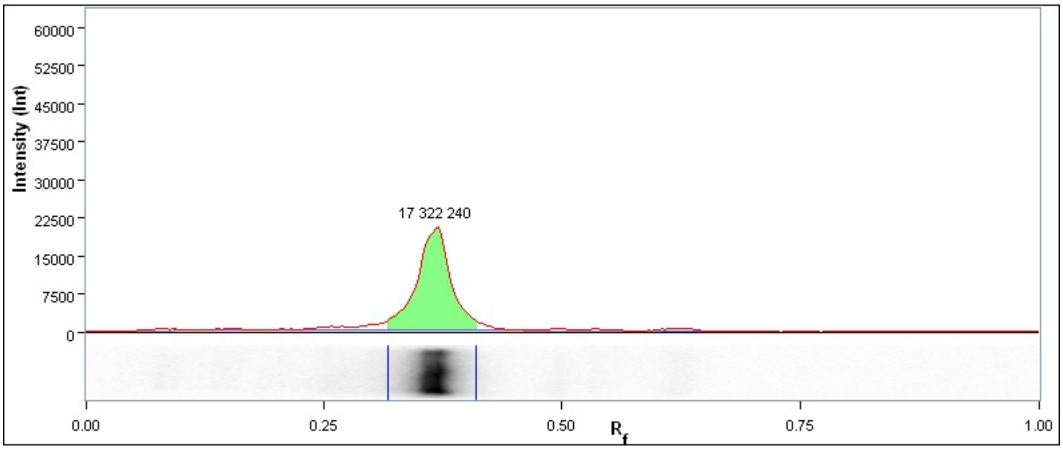

Band No.	Band Label	Mol. Wt. (KDa)	Relative Front	Volume (Int)	Abs. Quant.	Rel. Quant.	Band %	Lane %
1		N/A	0,369	17 322 240	N/A	N/A	100,0	81,7

Band Detection	Automatically detected bands with sensitivity: Low
Lane Background	Lane background subtracted with disk size: 10
Lane Width	7.62 mm

Lane 6

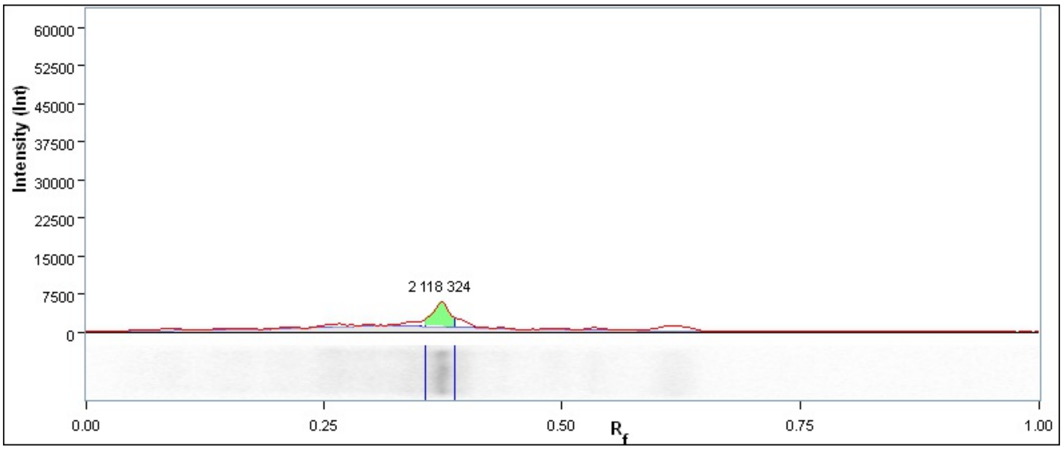

Band No.	Band Label	Mol. Wt. (KDa)	Relative Front	Volume (Int)	Abs. Quant.	Rel. Quant.	Band %	Lane %
1		N/A	0,375	2 118 324	N/A	N/A	100,0	33,9

Band Detection	Automatically detected bands with sensitivity: Low
Lane Background	Lane background subtracted with disk size: 10
Lane Width	7.62 mm

Lane 7

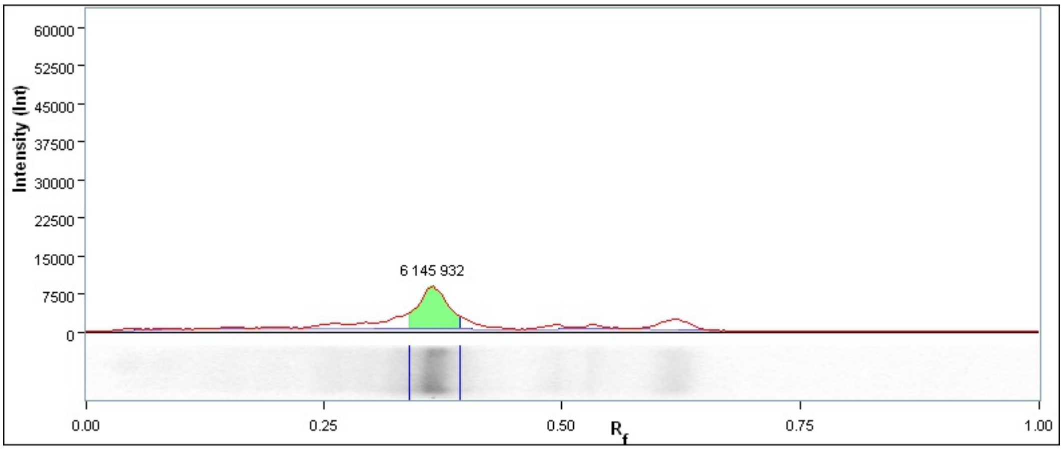

Band No.	Band Label	Mol. Wt. (KDa)	Relative Front	Volume (Int)	Abs. Quant.	Rel. Quant.	Band %	Lane %
1		N/A	0,366	6 145 932	N/A	N/A	100,0	46,1

Band Detection	Automatically detected bands with sensitivity: Low
Lane Background	Lane background subtracted with disk size: 10
Lane Width	7.62 mm

Lane 8

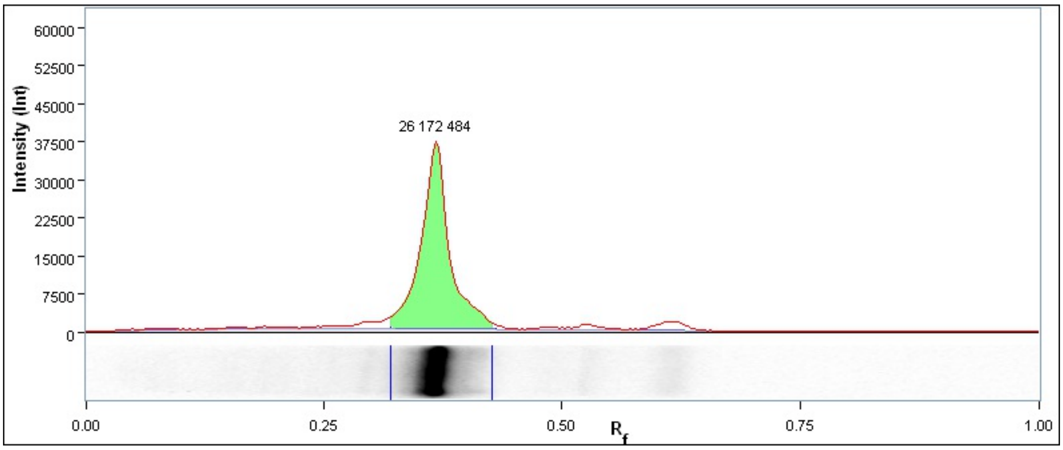

Band No.	Band Label	Mol. Wt. (KDa)	Relative Front	Volume (Int)	Abs. Quant.	Rel. Quant.	Band %	Lane %
1		N/A	0,369	26 172 484	N/A	N/A	100,0	84,4

Band Detection	Automatically detected bands with sensitivity: Low
Lane Background	Lane background subtracted with disk size: 10
Lane Width	7.62 mm

Volume Analysis

No.	Label	Type	Volume (Int)	Adj. Vol. (Int)	Mean Bkgd. (Int)	Abs. Quant.	Rel. Quant.	# of Pixels	Min. Value (Int)	Max. Value (Int)	Mean Value (Int)	Std. Dev.	Area (mm2)
1	U1	Unknown	15 059 776	11 674 471	1 567,3	N/A	N/A	2 160	0	43 780	6 972,1	7 528,5	46,4
2	U2	Unknown	19 532 856	14 940 741	2 126,0	N/A	N/A	2 160	140	37 304	9 043,0	8 480,7	46,4
3	U3	Unknown	17 882 756	13 368 761	2 089,8	N/A	N/A	2 160	120	23 668	8 279,1	5 547,3	46,4
4	U4	Unknown	2 131 088	485 888	761,7	N/A	N/A	2 160	0	5 240	986,6	775,3	46,4
5	U5	Unknown	18 758 648	15 533 633	1 493,1	N/A	N/A	2 160	0	32 348	8 684,6	8 053,6	46,4
6	U6	Unknown	5 611 360	2 512 120	1 434,8	N/A	N/A	2 160	0	9 692	2 597,9	1 777,4	46,4
7	U7	Unknown	9 889 544	5 365 739	2 094,4	N/A	N/A	2 160	0	15 972	4 578,5	2 945,3	46,4
8	U8	Unknown	28 275 136	23 097 751	2 396,9	N/A	N/A	2 160	0	52 820	13 090,3	12 381,9	46,4

Image Report: 2 żel 1 powt tub UMED 2018-02-14 11hr 01min_Exposure_16.8sec

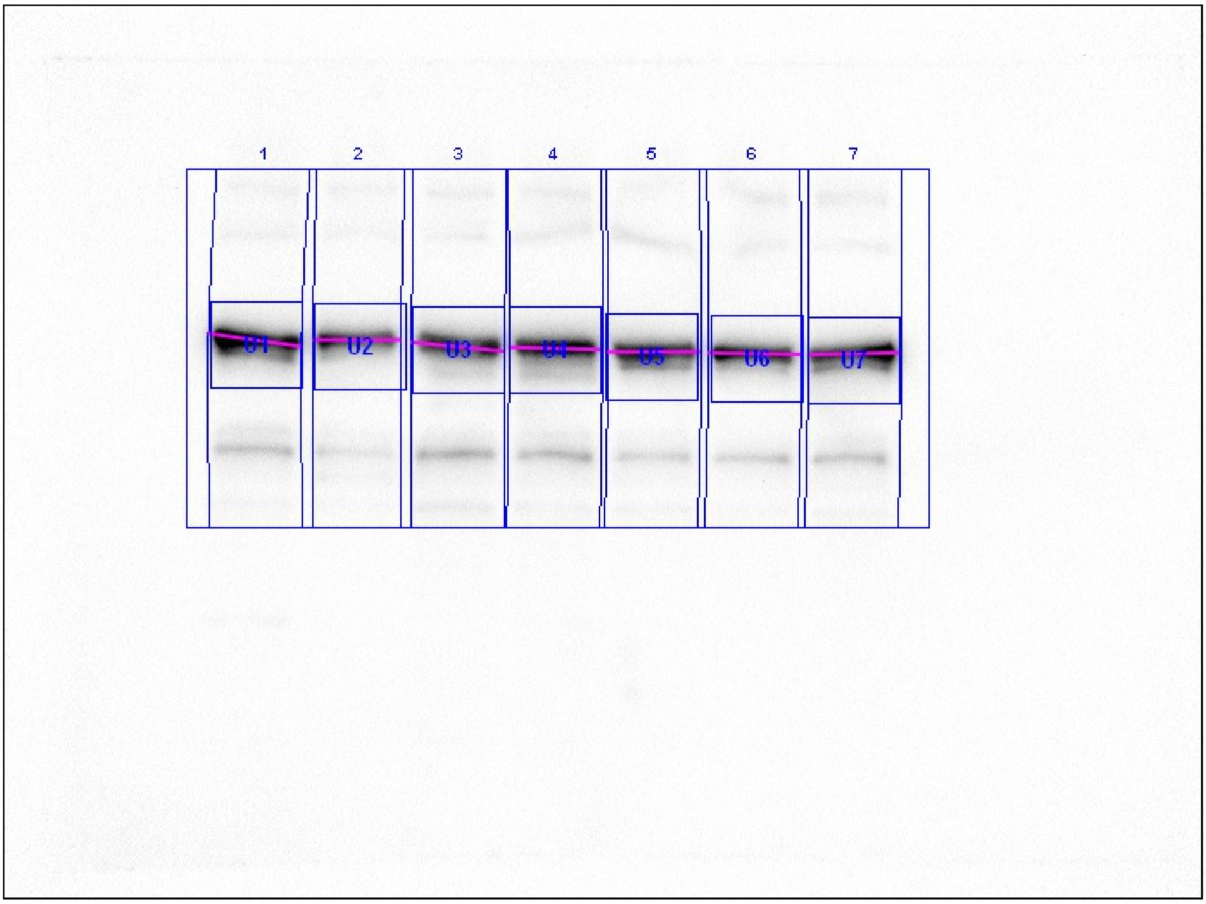

C:\Documents and Settings\Jaga\Pulpit\YKL-39 WB\analiza\żel 2\2 tub\2 żel 1 powt tub UMED 2018-02-14 11hr 01min_Exposure_16.8sec.scn

Acquisition Information

Imager	ChemiDoc™ MP
Exposure Time (sec)	16.800 (Signal Accumulation)
Flat Field	Applied (Lens)
Serial Number	731BR01769
Software Version	5.2.1
Application	Chemi Hi Resolution
Excitation Source	No Illumination
Emission Filter	No Filter
Binning	2x2

Image Information

Acquisition Date	2018-02-14 11:01:58
User Name	UMED
Image Area (mm)	X: 97.0 Y: 72.5
Pixel Size (um)	X: 139.4 Y: 139.4
Data Range (Int)	0 - 47568

Analysis Settings

Detection	Lane detection:<br>Automatically detected lanes with manual adjustments<br><br>Band detection:<br>Automatically detected bands with sensitivity: Low<br>Manually adjusted bands<br><br>Lane Background Subtraction:<br>Lane background subtracted with disk size: 10<br><br>Lane width: Variable
Volume Analysis	Background subtraction method: Local<br>Quantity regression method: Linear

Lane And Band Analysis

Lane 1

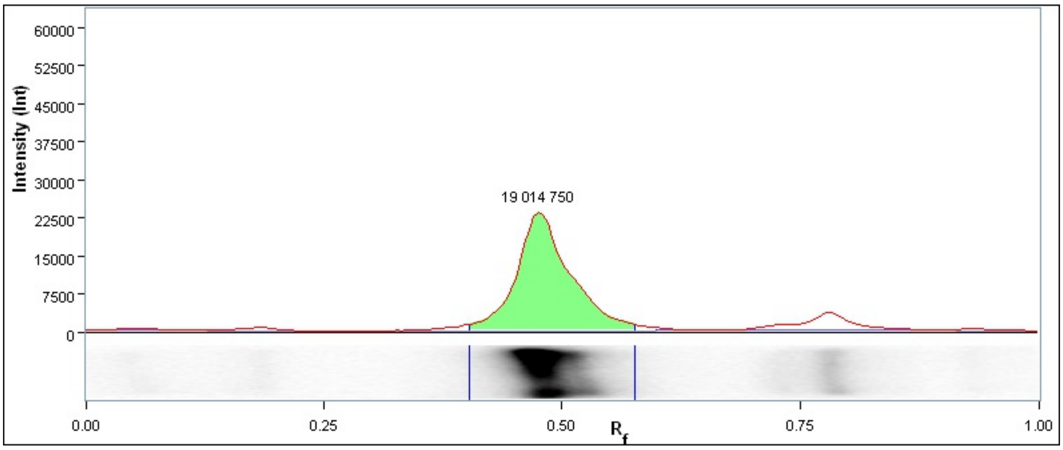

Band No.	Band Label	Mol. Wt. (KDa)	Relative Front	Volume (Int)	Abs. Quant.	Rel. Quant.	Band %	Lane %
1		N/A	0,478	19 014 750	N/A	N/A	100,0	80,5

Band Detection	Automatically detected bands with sensitivity: Low
Lane Background	Lane background subtracted with disk size: 10
Lane Width	7.53 mm

Lane 2

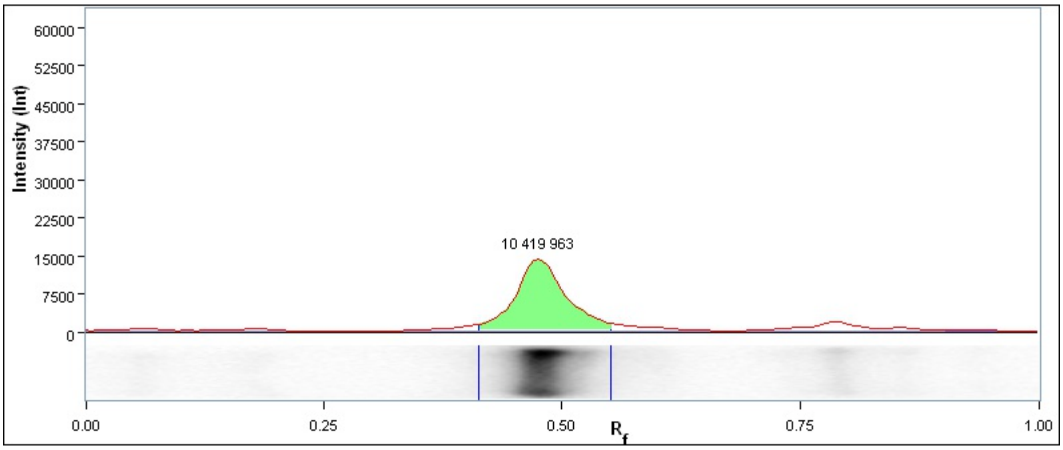

Band No.	Band Label	Mol. Wt. (KDa)	Relative Front	Volume (Int)	Abs. Quant.	Rel. Quant.	Band %	Lane %
1		N/A	0,478	10 419 963	N/A	N/A	100,0	74,6

Band Detection	Automatically detected bands with sensitivity: Low

Lane Background	Lane background subtracted with disk size: 10
Lane Width	7.11 mm

Lane 3

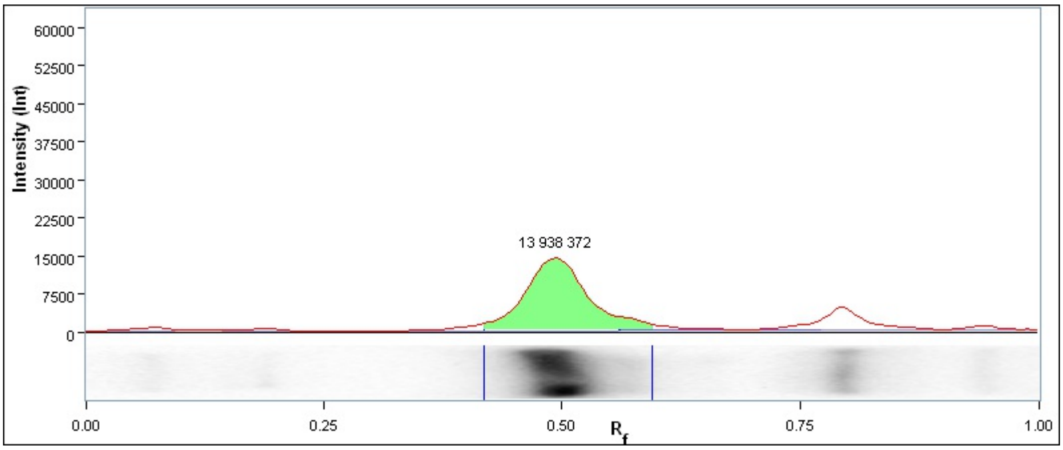

Band No.	Band Label	Mol. Wt. (KDa)	Relative Front	Volume (Int)	Abs. Quant.	Rel. Quant.	Band %	Lane %
1		N/A	0,498	13 938 372	N/A	N/A	100,0	73,5

Band Detection	Automatically detected bands with sensitivity: Low
Lane Background	Lane background subtracted with disk size: 10
Lane Width	7.53 mm

Lane 4

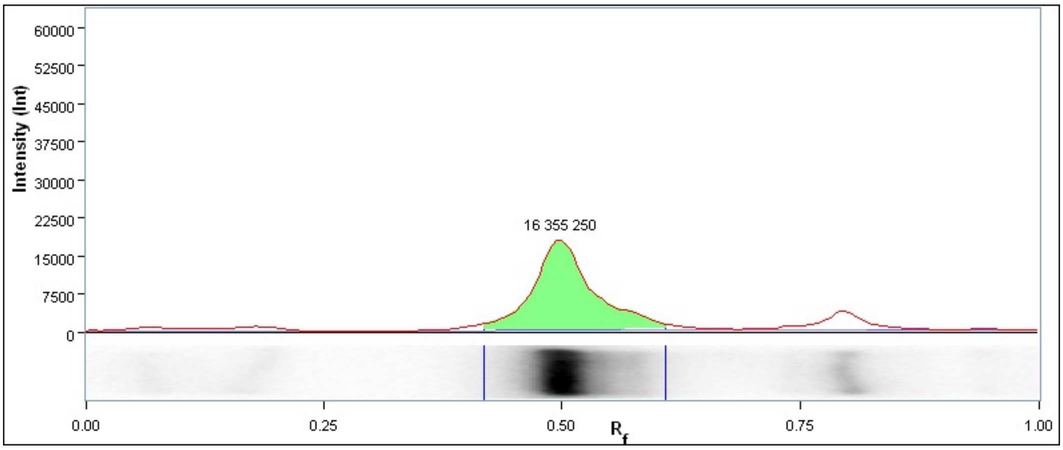

Band No.	Band Label	Mol. Wt. (KDa)	Relative Front	Volume (Int)	Abs. Quant.	Rel. Quant.	Band %	Lane %
1		N/A	0,502	16 355 250	N/A	N/A	100,0	77,4

Band Detection	Automatically detected bands with sensitivity: Low
Lane Background	Lane background subtracted with disk size: 10
Lane Width	7.53 mm

Lane 5

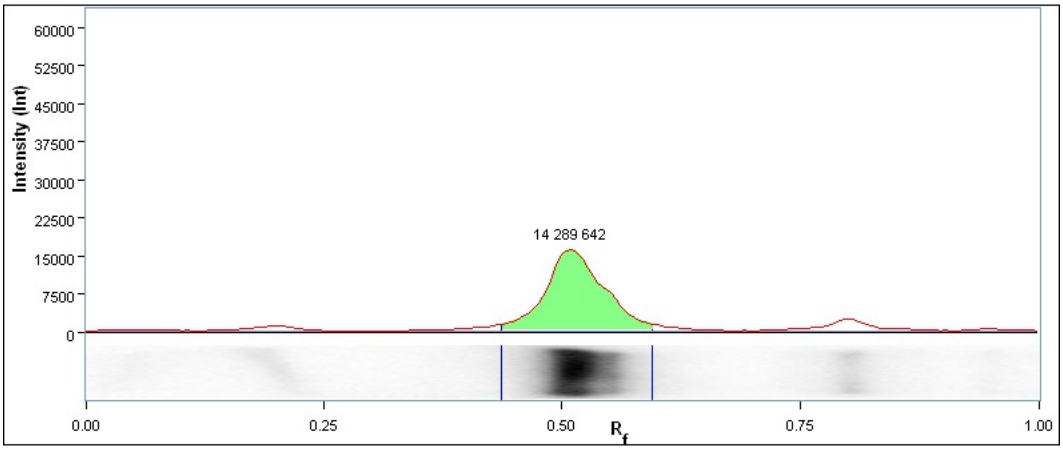

Band No.	Band Label	Mol. Wt. (KDa)	Relative Front	Volume (Int)	Abs. Quant.	Rel. Quant.	Band %	Lane %
1		N/A	0,512	14 289 642	N/A	N/A	100,0	79,0

Band Detection	Automatically detected bands with sensitivity: Low
Lane Background	Lane background subtracted with disk size: 10
Lane Width	7.53 mm

Lane 6

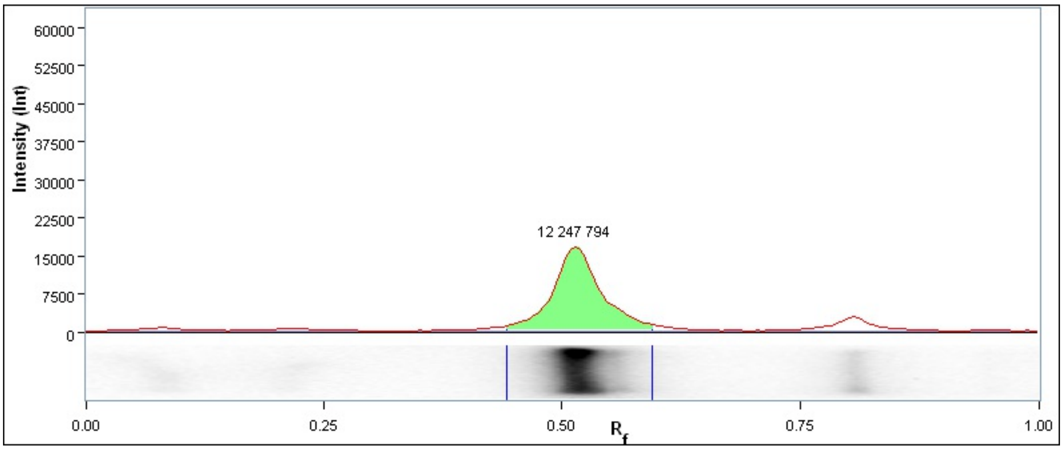

Band No.	Band Label	Mol. Wt. (KDa)	Relative Front	Volume (Int)	Abs. Quant.	Rel. Quant.	Band %	Lane %
1		N/A	0,517	12 247 794	N/A	N/A	100,0	79,6

Band Detection	Automatically detected bands with sensitivity: Low
Lane Background	Lane background subtracted with disk size: 10
Lane Width	7.53 mm

Lane 7

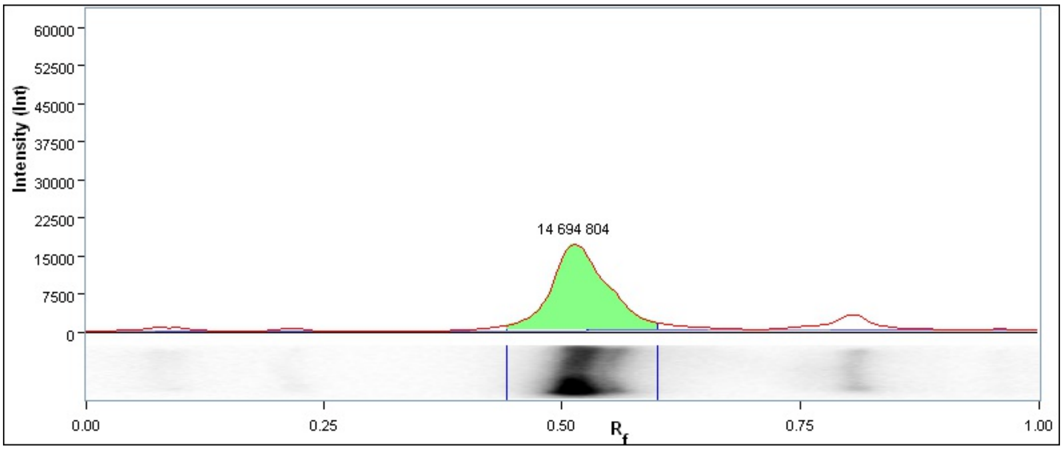

Band No.	Band Label	Mol. Wt. (KDa)	Relative Front	Volume (Int)	Abs. Quant.	Rel. Quant.	Band %	Lane %
1		N/A	0,517	14 694 804	N/A	N/A	100,0	77,9

Band Detection	Automatically detected bands with sensitivity: Low
Lane Background	Lane background subtracted with disk size: 10
Lane Width	7.53 mm

Volume Analysis

No.	Label	Type	Volume (Int)	Adj. Vol. (Int)	Mean Bkgd. (Int)	Abs. Quant.	Rel. Quant.	# of Pixels	Min. Value (Int)	Max. Value (Int)	Mean Value (Int)	Std. Dev.	Area (mm2)
1	U1	Unknown	20 514 688	15 951 792	1 721,8	N/A	N/A	2 650	88	47 568	7 741,4	8 731,4	51,5
2	U2	Unknown	12 310 576	8 657 665	1 378,5	N/A	N/A	2 650	0	23 840	4 645,5	4 905,5	51,5
3	U3	Unknown	15 494 120	11 418 723	1 537,9	N/A	N/A	2 650	104	22 616	5 846,8	5 456,5	51,5
4	U4	Unknown	18 004 248	13 206 486	1 810,5	N/A	N/A	2 650	400	25 764	6 794,1	6 270,0	51,5
5	U5	Unknown	15 728 460	11 345 814	1 653,8	N/A	N/A	2 650	0	22 880	5 935,3	6 054,6	51,5
6	U6	Unknown	13 646 120	10 127 879	1 327,6	N/A	N/A	2 650	0	28 808	5 149,5	5 878,6	51,5
7	U7	Unknown	16 464 948	12 268 307	1 583,6	N/A	N/A	2 650	0	27 372	6 213,2	6 352,9	51,5

Image Report: 2 żel 1 powt YKL39 UMED 2018-02-13 11hr 08min_Exposure_60.0sec

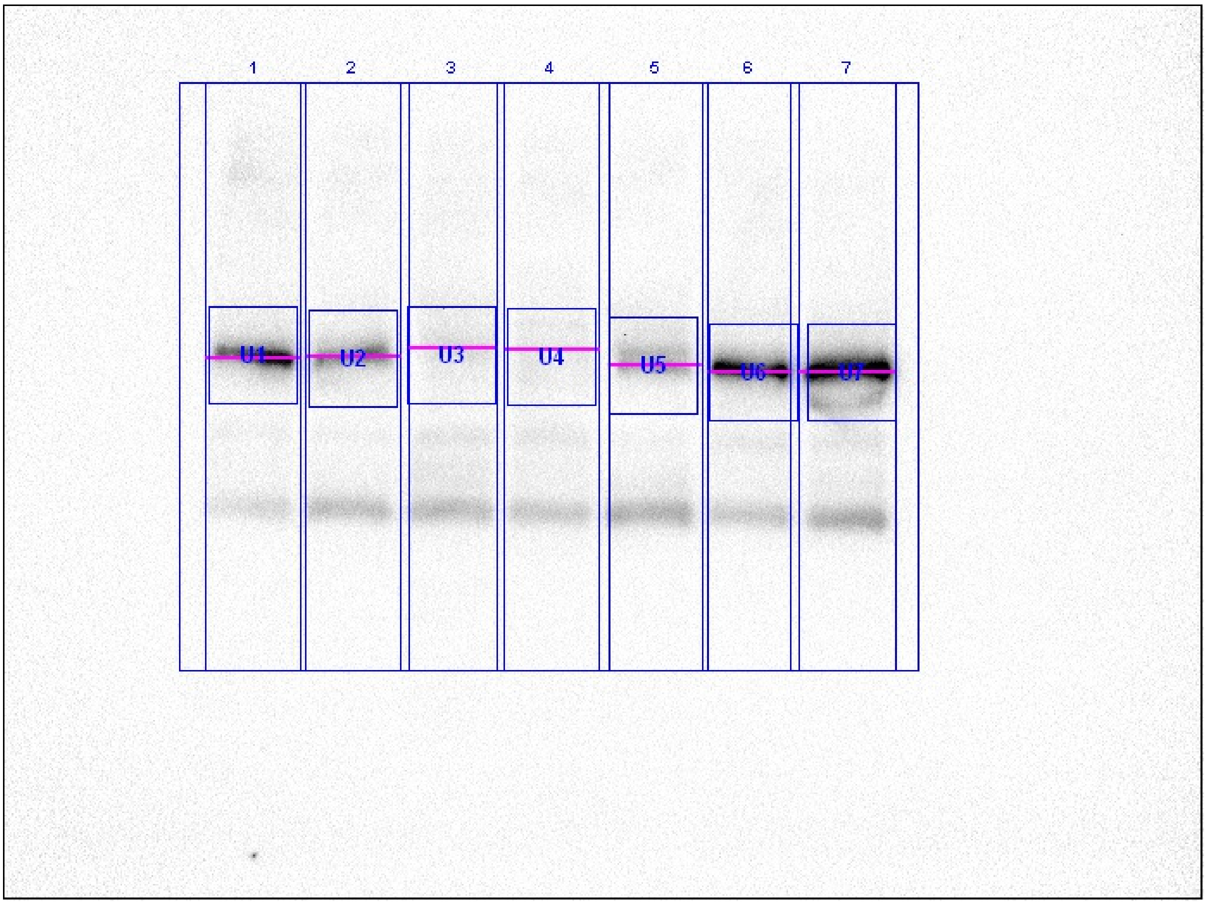

C:\Documents and Settings\Jaga\Pulpit\YKL-39 WB\analiza\żel 2\2 YKL39\2 żel 1 powt YKL39 UMED 2018-02-13 11hr 08min_Exposure_60.0sec.scn

Acquisition Information

Imager	ChemiDoc™ MP
Exposure Time (sec)	60.000 (Signal Accumulation)
Flat Field	Applied (Lens)
Serial Number	731BR01769
Software Version	5.2.1
Application	Chemi Hi Resolution
Excitation Source	No Illumination
Emission Filter	No Filter
Binning	2x2

Image Information

Acquisition Date	2018-02-13 11:09:57
User Name	UMED
Image Area (mm)	X: 97.0 Y: 72.5
Pixel Size (um)	X: 139.4 Y: 139.4
Data Range (Int)	0 - 24020

Analysis Settings

Detection	Lane detection:<br>Manually created lanes<br><br>Band detection:<br>Automatically detected bands with sensitivity: Low<br>Manually adjusted bands<br><br>Lane Background Subtraction:<br>Lane background subtracted with disk size: 10<br><br>Lane width: Variable
Volume Analysis	Background subtraction method: Local<br>Quantity regression method: Linear

Lane And Band Analysis

Lane 1

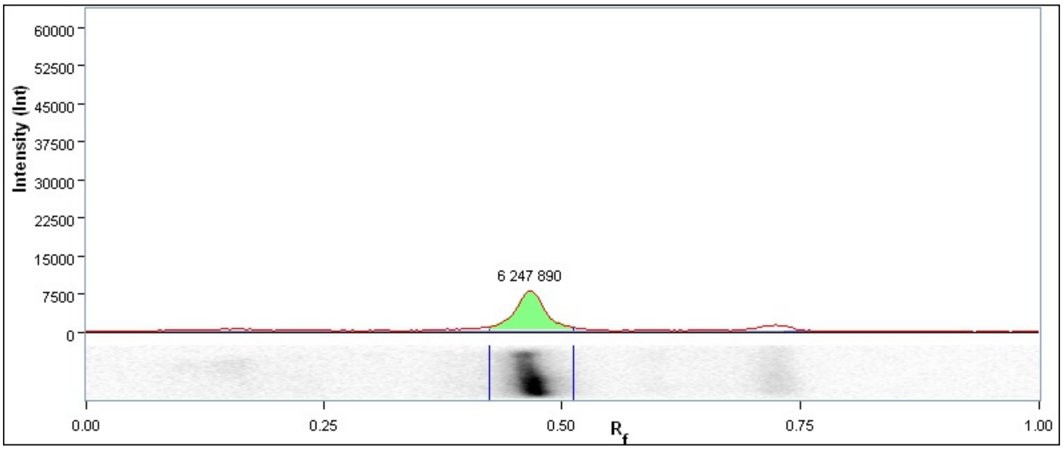

Band No.	Band Label	Mol. Wt. (KDa)	Relative Front	Volume (Int)	Abs. Quant.	Rel. Quant.	Band %	Lane %
1		N/A	0,469	6 247 890	N/A	N/A	100,0	65,5

Band Detection	Automatically detected bands with sensitivity: Low
Lane Background	Lane background subtracted with disk size: 10
Lane Width	7.67 mm

Lane 2

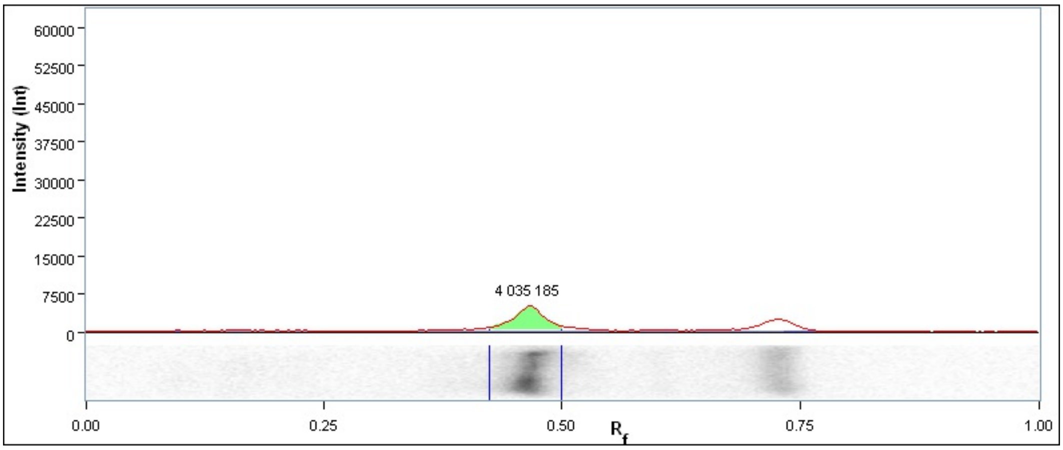

Band No.	Band Label	Mol. Wt. (KDa)	Relative Front	Volume (Int)	Abs. Quant.	Rel. Quant.	Band %	Lane %
1		N/A	0,466	4 035 185	N/A	N/A	100,0	49,8

Band Detection	Automatically detected bands with sensitivity: Low

Lane Background	Lane background subtracted with disk size: 10
Lane Width	7.67 mm

Lane 3

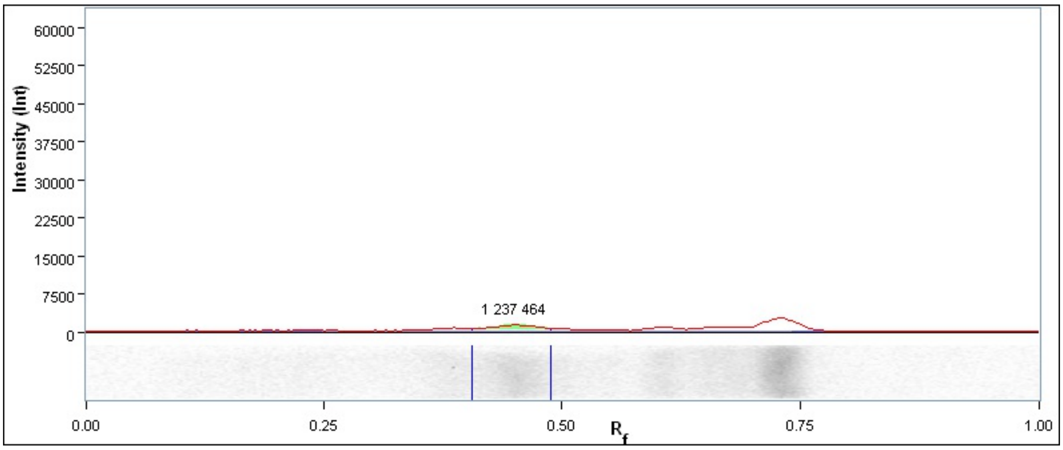

Band No.	Band Label	Mol. Wt. (KDa)	Relative Front	Volume (Int)	Abs. Quant.	Rel. Quant.	Band %	Lane %
1		N/A	0,452	1 237 464	N/A	N/A	100,0	19,2

Band Detection	Automatically detected bands with sensitivity: Low
Lane Background	Lane background subtracted with disk size: 10
Lane Width	7.11 mm

Lane 4

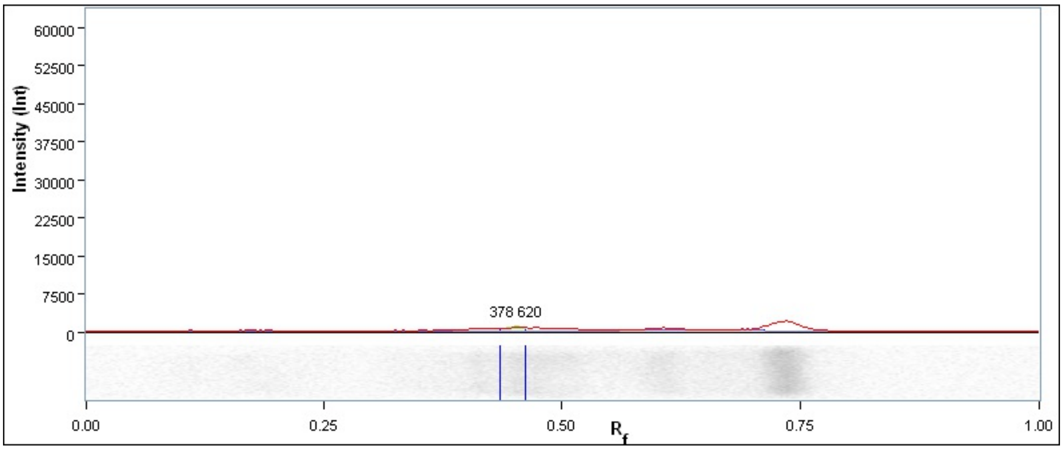

Band No.	Band Label	Mol. Wt. (KDa)	Relative Front	Volume (Int)	Abs. Quant.	Rel. Quant.	Band %	Lane %
1		N/A	0,455	378 620	N/A	N/A	100,0	8,2

Band Detection	Automatically detected bands with sensitivity: Low
Lane Background	Lane background subtracted with disk size: 10
Lane Width	7.67 mm

Lane 5

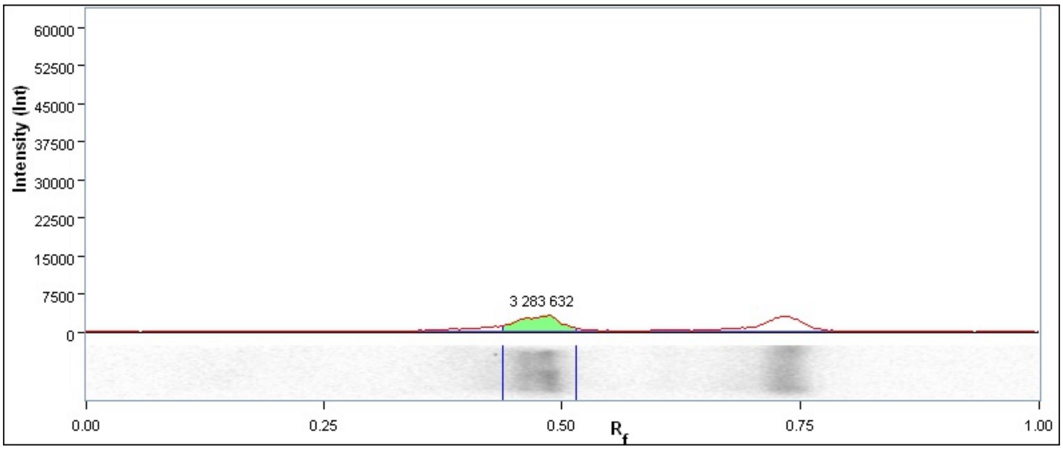

Band No.	Band Label	Mol. Wt. (KDa)	Relative Front	Volume (Int)	Abs. Quant.	Rel. Quant.	Band %	Lane %
1		N/A	0,481	3 283 632	N/A	N/A	100,0	36,1

Band Detection	Automatically detected bands with sensitivity: Low
Lane Background	Lane background subtracted with disk size: 10
Lane Width	7.53 mm

Lane 6

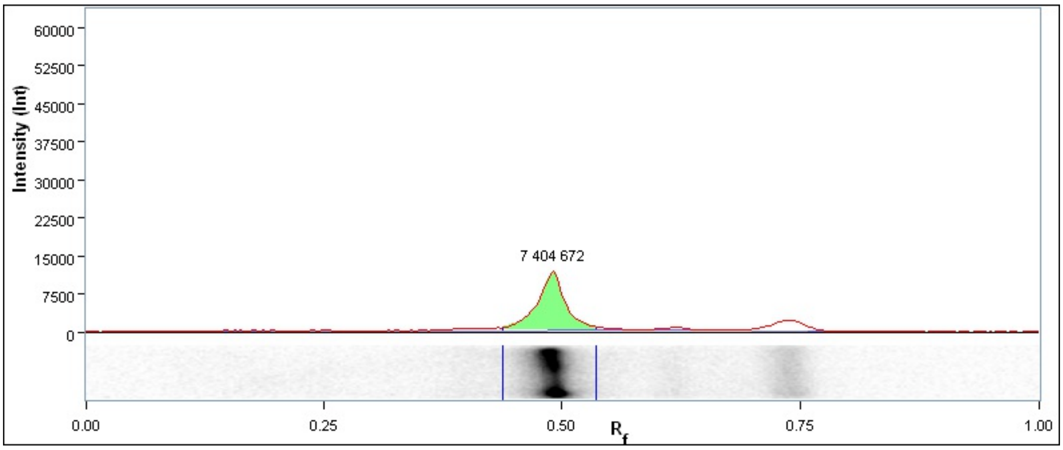

Band No.	Band Label	Mol. Wt. (KDa)	Relative Front	Volume (Int)	Abs. Quant.	Rel. Quant.	Band %	Lane %
1		N/A	0,493	7 404 672	N/A	N/A	100,0	67,1

Band Detection	Automatically detected bands with sensitivity: Low
Lane Background	Lane background subtracted with disk size: 10
Lane Width	6.69 mm

Lane 7

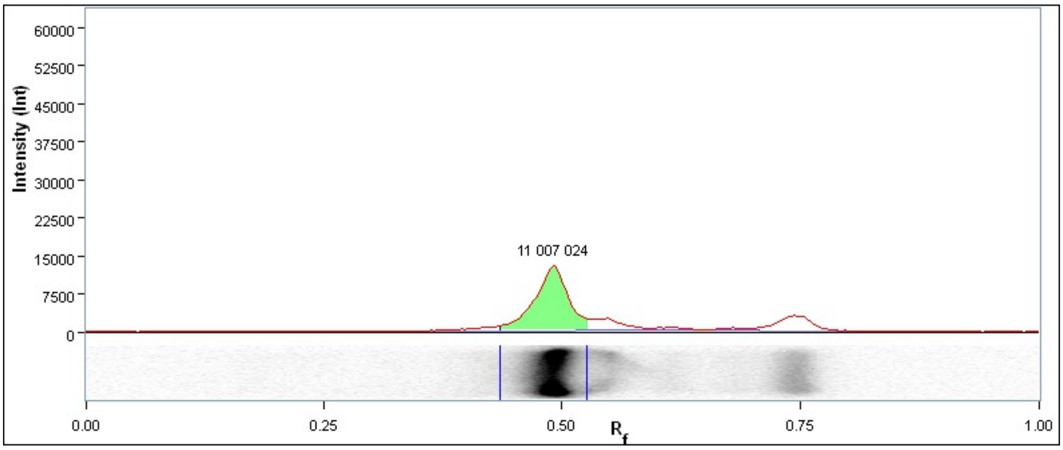

Band No.	Band Label	Mol. Wt. (KDa)	Relative Front	Volume (Int)	Abs. Quant.	Rel. Quant.	Band %	Lane %
1		N/A	0,493	11 007 024	N/A	N/A	100,0	62,0

Band Detection	Automatically detected bands with sensitivity: Low
Lane Background	Lane background subtracted with disk size: 10
Lane Width	7.80 mm

Volume Analysis

No.	Label	Type	Volume (Int)	Adj. Vol. (Int)	Mean Bkgd. (Int)	Abs. Quant.	Rel. Quant.	# of Pixels	Min. Value (Int)	Max. Value (Int)	Mean Value (Int)	Std. Dev.	Area (mm2)
1	U1	Unknown	7 762 124	5 709 158	718,8	N/A	N/A	2 856	0	16 020	2 717,8	3 155,0	55,5
2	U2	Unknown	5 608 264	3 938 211	584,8	N/A	N/A	2 856	0	9 224	1 963,7	1 852,1	55,5
3	U3	Unknown	2 766 124	1 247 204	531,8	N/A	N/A	2 856	0	6 796	968,5	604,8	55,5
4	U4	Unknown	2 401 768	1 038 225	477,4	N/A	N/A	2 856	0	2 528	841,0	461,8	55,5
5	U5	Unknown	4 674 784	3 182 590	522,5	N/A	N/A	2 856	0	9 116	1 636,8	1 337,4	55,5
6	U6	Unknown	9 349 960	6 693 670	930,1	N/A	N/A	2 856	0	22 700	3 273,8	3 797,6	55,5
7	U7	Unknown	13 867 160	9 274 817	1 608,0	N/A	N/A	2 856	0	24 020	4 855,4	4 419,0	55,5

Image Report: 2 żel 2 powt tub

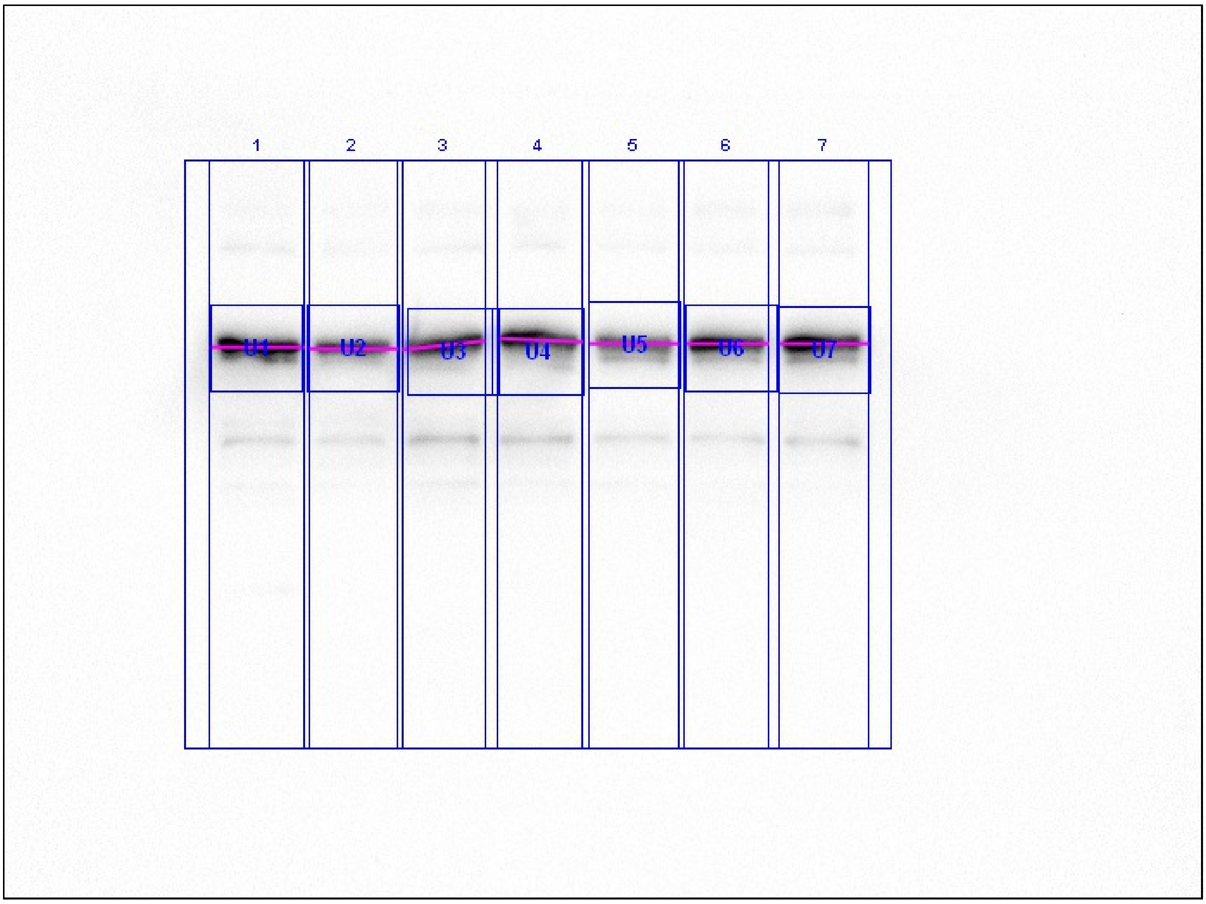

C:\Documents and Settings\Jaga\Pulpit\YKL-39 WB\analiza\żel 2\2 tub\2 żel 2 powt tub.scn

Acquisition Information

Imager	ChemiDoc™ MP
Exposure Time (sec)	16.800 (Signal Accumulation)
Flat Field	Applied (Lens)
Serial Number	731BR01769
Software Version	5.2.1
Application	Chemi Hi Resolution
Excitation Source	No Illumination
Emission Filter	No Filter
Binning	2x2

Image Information

Acquisition Date	2018-02-21 11:02:04
User Name	UMED
Image Area (mm)	X: 103.0 Y: 77.0
Pixel Size (um)	X: 148.0 Y: 148.0
Data Range (Int)	0 - 39376

Analysis Settings

Detection	Lane detection:<br>Manually created lanes

	Band detection:<br>Automatically detected bands with sensitivity: Low<br>Manually adjusted bands<br><br>Lane Background Subtraction:<br>Lane background subtracted with disk size: 10<br><br>Lane width: Variable
Volume Analysis	Background subtraction method: Local<br>Quantity regression method: Linear

Lane And Band Analysis

Lane 1

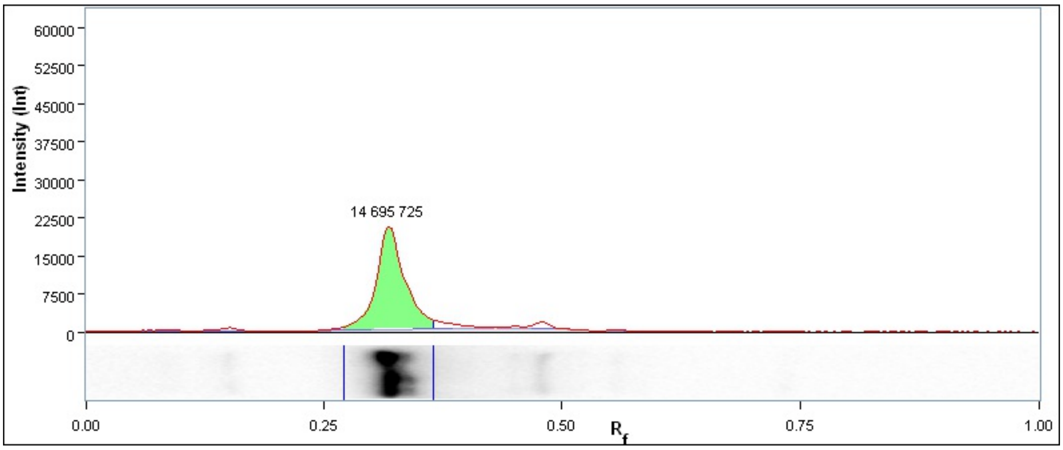

Band No.	Band Label	Mol. Wt. (KDa)	Relative Front	Volume (Int)	Abs. Quant.	Rel. Quant.	Band %	Lane %
1		N/A	0,320	14 695 725	N/A	N/A	100,0	83,2

Band Detection	Automatically detected bands with sensitivity: Low
Lane Background	Lane background subtracted with disk size: 10
Lane Width	8.14 mm

Lane 2

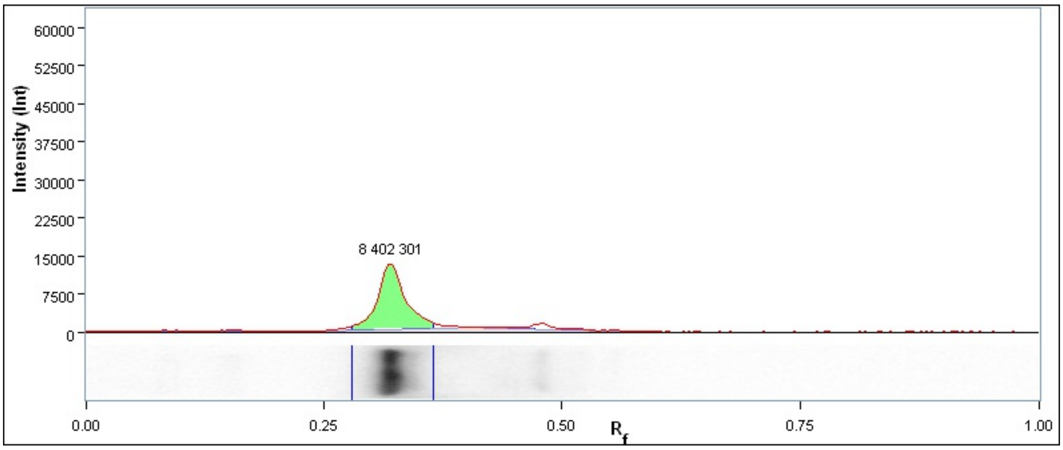

Band No.	Band Label	Mol. Wt. (KDa)	Relative Front	Volume (Int)	Abs. Quant.	Rel. Quant.	Band %	Lane %
1		N/A	0,323	8 402 301	N/A	N/A	100,0	80,5

Band Detection	Automatically detected bands with sensitivity: Low
Lane Background	Lane background subtracted with disk size: 10
Lane Width	7.55 mm

Lane 3

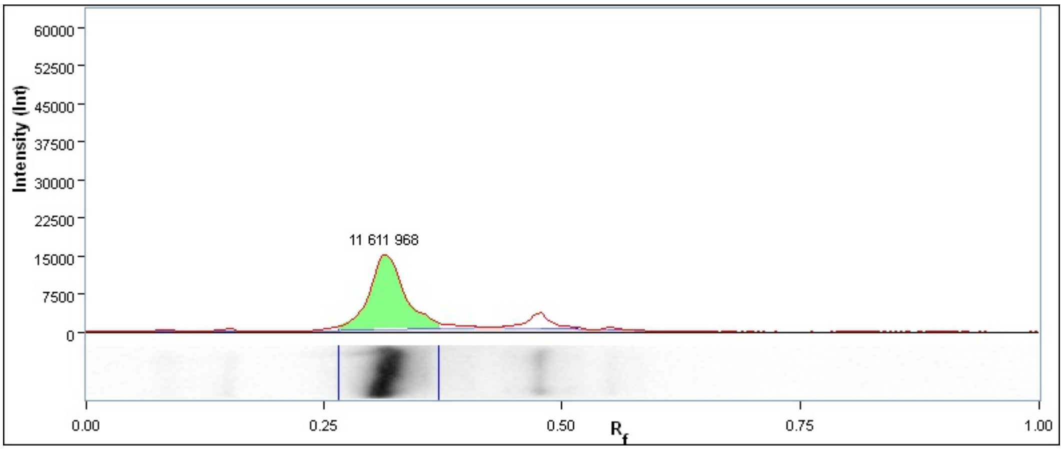

Band No.	Band Label	Mol. Wt. (KDa)	Relative Front	Volume (Int)	Abs. Quant.	Rel. Quant.	Band %	Lane %
1		N/A	0,317	11 611 968	N/A	N/A	100,0	78,4

Band Detection	Automatically detected bands with sensitivity: Low
Lane Background	Lane background subtracted with disk size: 10
Lane Width	7.10 mm

Lane 4

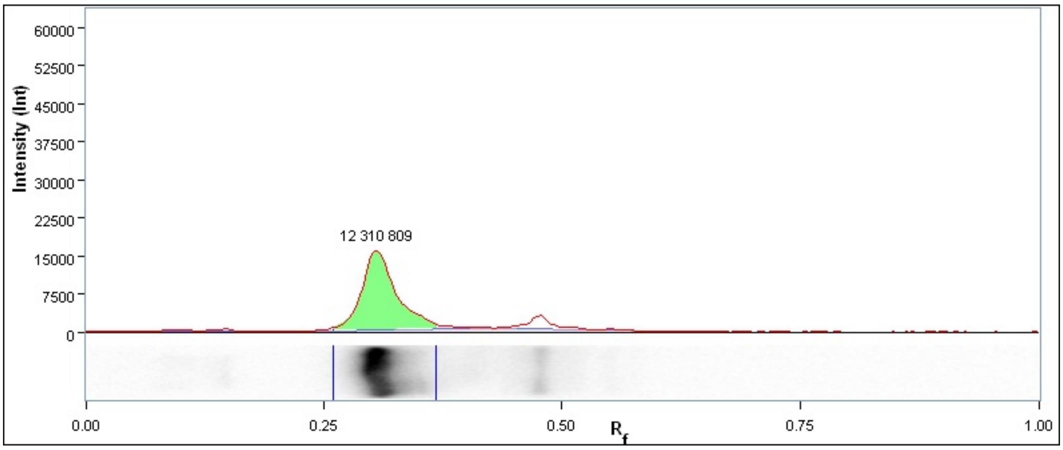

Band No.	Band Label	Mol. Wt. (KDa)	Relative Front	Volume (Int)	Abs. Quant.	Rel. Quant.	Band %	Lane %
1		N/A	0,308	12 310 809	N/A	N/A	100,0	81,0

Band Detection	Automatically detected bands with sensitivity: Low
Lane Background	Lane background subtracted with disk size: 10
Lane Width	7.25 mm

Lane 5

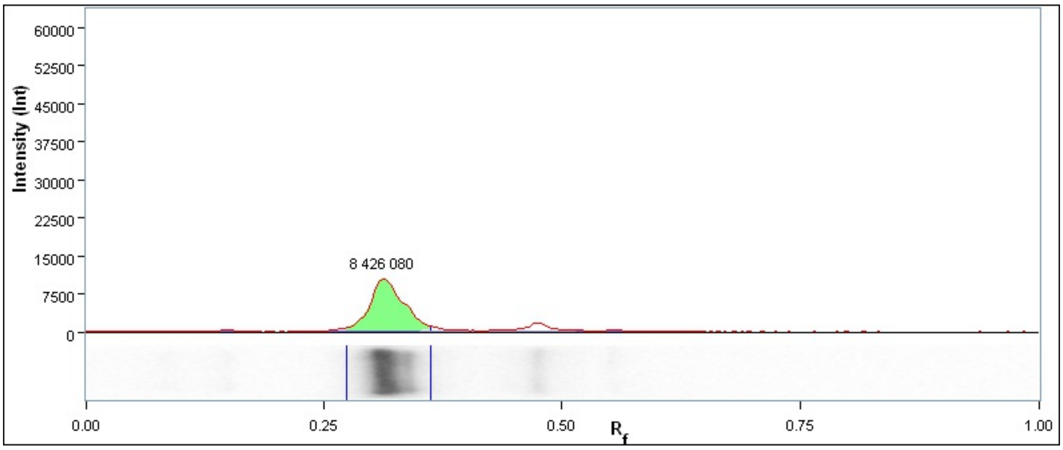

Band No.	Band Label	Mol. Wt. (KDa)	Relative Front	Volume (Int)	Abs. Quant.	Rel. Quant.	Band %	Lane %
1		N/A	0,314	8 426 080	N/A	N/A	100,0	79,1

Band Detection	Automatically detected bands with sensitivity: Low
Lane Background	Lane background subtracted with disk size: 10
Lane Width	7.70 mm

Lane 6

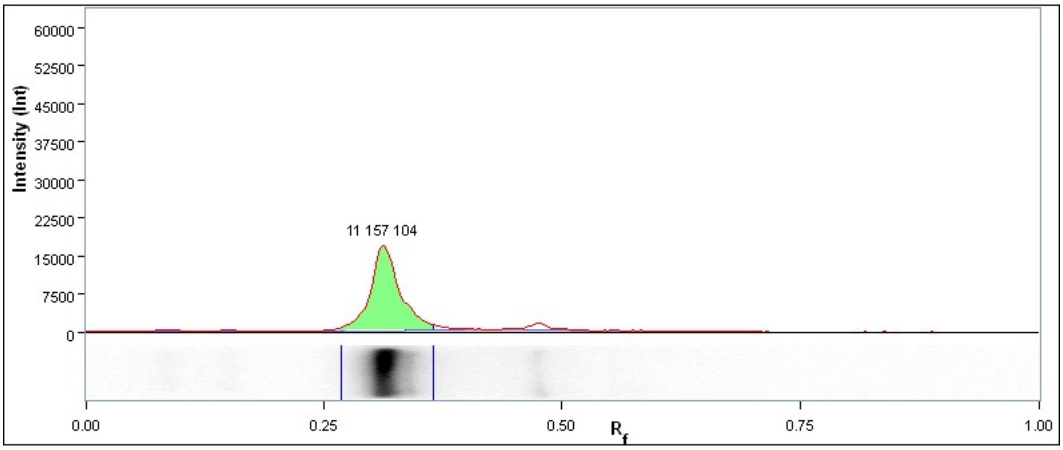

Band No.	Band Label	Mol. Wt. (KDa)	Relative Front	Volume (Int)	Abs. Quant.	Rel. Quant.	Band %	Lane %
1		N/A	0,314	11 157 104	N/A	N/A	100,0	86,2

Band Detection	Automatically detected bands with sensitivity: Low
Lane Background	Lane background subtracted with disk size: 10
Lane Width	7.25 mm

Lane 7

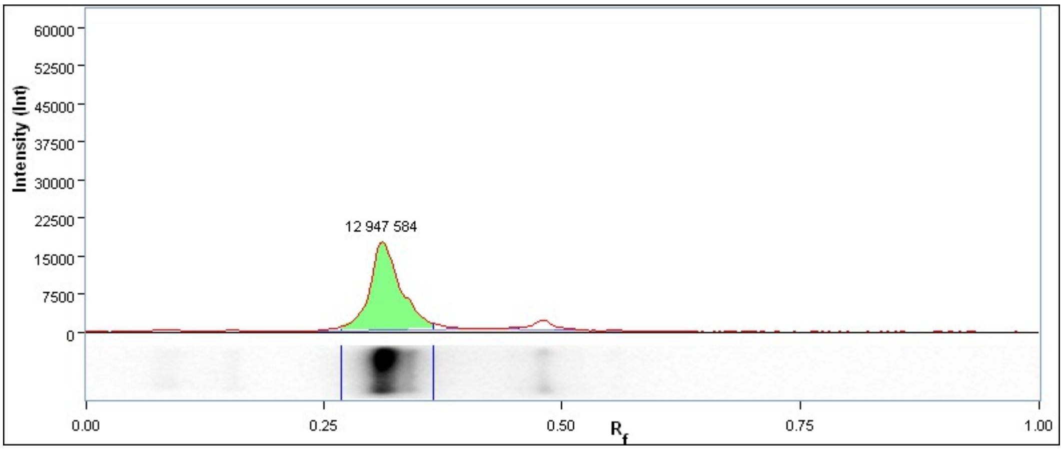

Band No.	Band Label	Mol. Wt. (KDa)	Relative Front	Volume (Int)	Abs. Quant.	Rel. Quant.	Band %	Lane %
1		N/A	0,314	12 947 584	N/A	N/A	100,0	83,2

Band Detection	Automatically detected bands with sensitivity: Low
Lane Background	Lane background subtracted with disk size: 10
Lane Width	7.70 mm

Volume Analysis

No.	Label	Type	Volume (Int)	Adj. Vol. (Int)	Mean Bkgd. (Int)	Abs. Quant.	Rel. Quant.	# of Pixels	Min. Value (Int)	Max. Value (Int)	Mean Value (Int)	Std. Dev.	Area (mm2)
1	U1	Unknown	16 975 180	12 441 459	1 710,8	N/A	N/A	2 650	0	39 376	6 405,7	7 369,5	58,0
2	U2	Unknown	10 762 144	6 388 130	1 650,6	N/A	N/A	2 650	0	19 068	4 061,2	4 354,4	58,0
3	U3	Unknown	14 013 004	7 805 240	2 342,6	N/A	N/A	2 650	56	20 972	5 287,9	5 231,6	58,0
4	U4	Unknown	14 446 304	11 152 127	1 243,1	N/A	N/A	2 650	68	25 404	5 451,4	5 655,2	58,0
5	U5	Unknown	9 551 188	7 415 238	806,0	N/A	N/A	2 650	0	14 752	3 604,2	3 916,0	58,0
6	U6	Unknown	12 825 252	10 036 342	1 052,4	N/A	N/A	2 650	0	25 920	4 839,7	5 750,1	58,0
7	U7	Unknown	14 815 224	12 084 210	1 030,6	N/A	N/A	2 650	0	31 772	5 590,7	6 435,2	58,0

Image Report: 2 żel 2 powt YKL39 UMED 2018-02-20 10hr 58min_Exposure_60.0sec

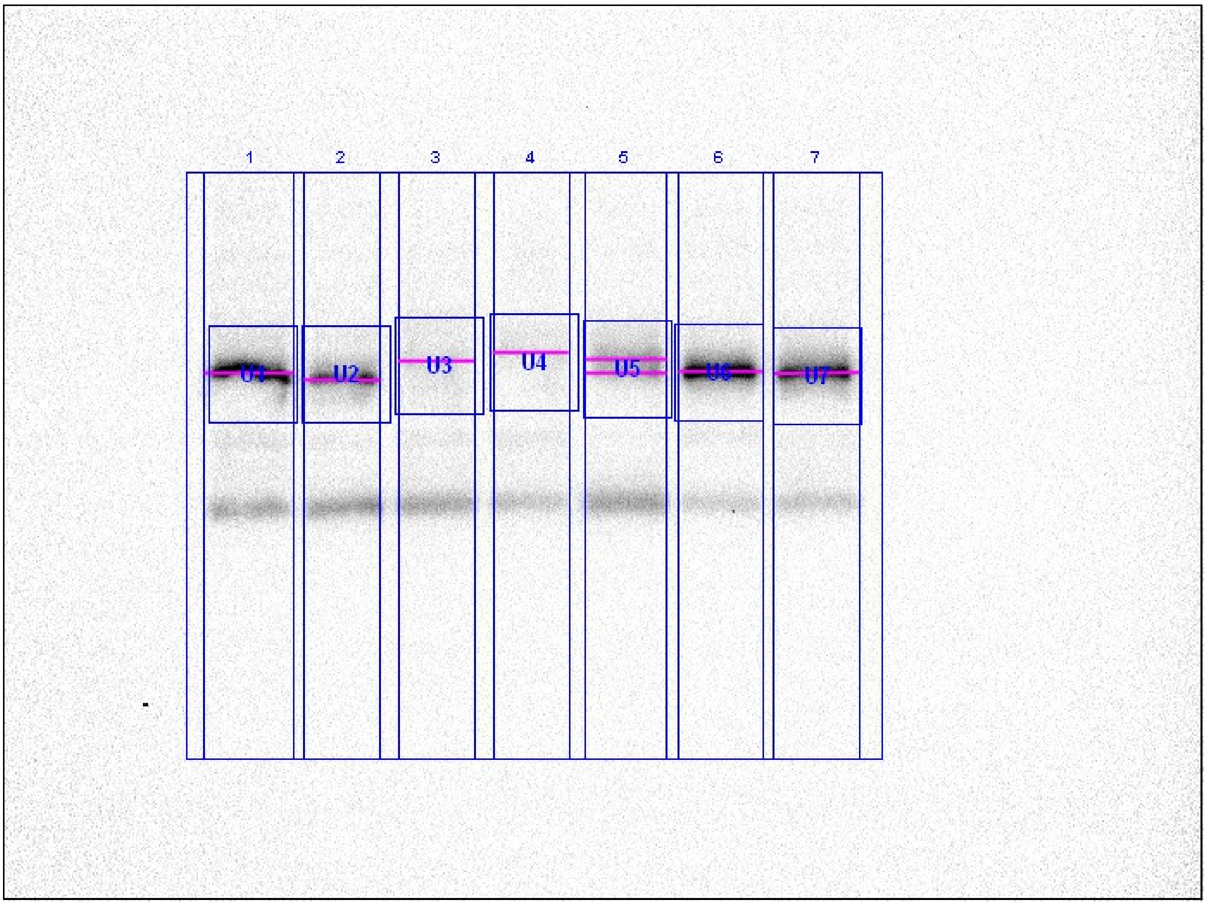

C:\Documents and Settings\Jaga\Pulpit\YKL-39 WB\analiza\żel 2\2 YKL39\2 żel 2 powt YKL39 UMED 2018-02-20 10hr 58min_Exposure_60.0sec.scn

Acquisition Information

Imager	ChemiDoc™ MP
Exposure Time (sec)	60.000 (Signal Accumulation)
Flat Field	Applied (Lens)
Serial Number	731BR01769
Software Version	5.2.1
Application	Chemi Hi Resolution
Excitation Source	No Illumination
Emission Filter	No Filter
Binning	2x2

Image Information

Acquisition Date	2018-02-20 10:59:39
User Name	UMED
Image Area (mm)	X: 103.0 Y: 77.0
Pixel Size (um)	X: 148.0 Y: 148.0
Data Range (Int)	0 - 19936

Analysis Settings

Detection	Lane detection:<br>Manually created lanes<br><br>Band detection:<br>Automatically detected bands with sensitivity: Low<br>Manually adjusted bands<br><br>Lane Background Subtraction:<br>Lane background subtracted with disk size: 10<br><br>Lane width: Variable
Volume Analysis	Background subtraction method: Local<br>Quantity regression method: Linear

Lane And Band Analysis

Lane 1

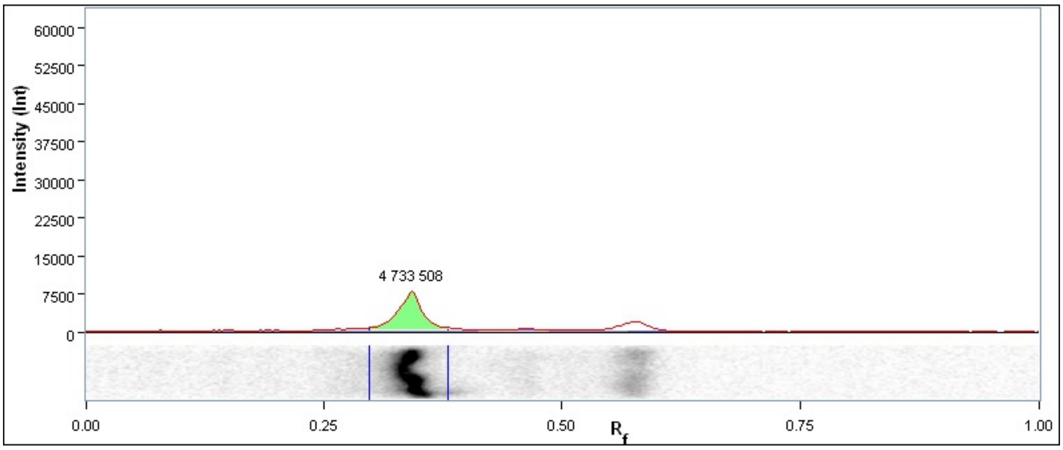

Band No.	Band Label	Mol. Wt. (KDa)	Relative Front	Volume (Int)	Abs. Quant.	Rel. Quant.	Band %	Lane %
1		N/A	0,344	4 733 508	N/A	N/A	100,0	60,6

Band Detection	Automatically detected bands with sensitivity: Low
Lane Background	Lane background subtracted with disk size: 10
Lane Width	7.70 mm

Lane 2

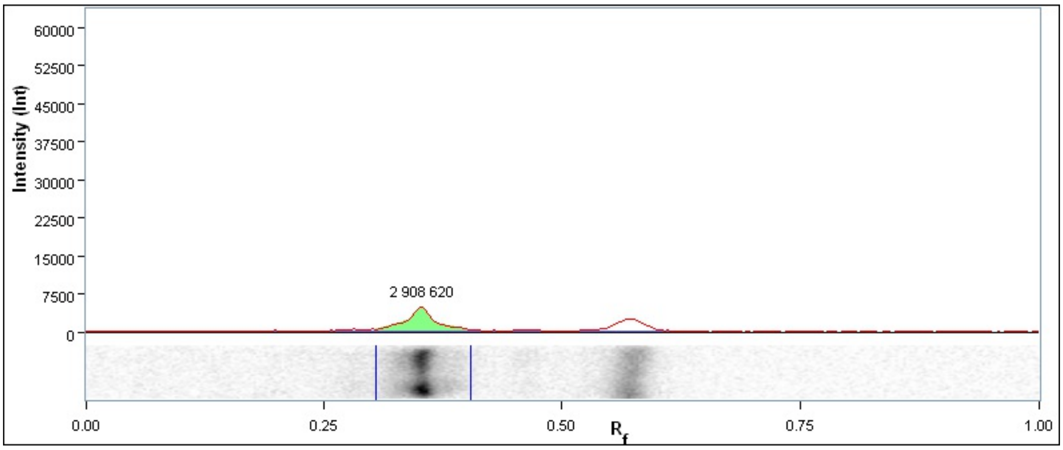

Band No.	Band Label	Mol. Wt. (KDa)	Relative Front	Volume (Int)	Abs. Quant.	Rel. Quant.	Band %	Lane %
1		N/A	0,356	2 908 620	N/A	N/A	100,0	51,4

Band Detection	Automatically detected bands with sensitivity: Low

Lane Background	Lane background subtracted with disk size: 10
Lane Width	6.51 mm

Lane 3

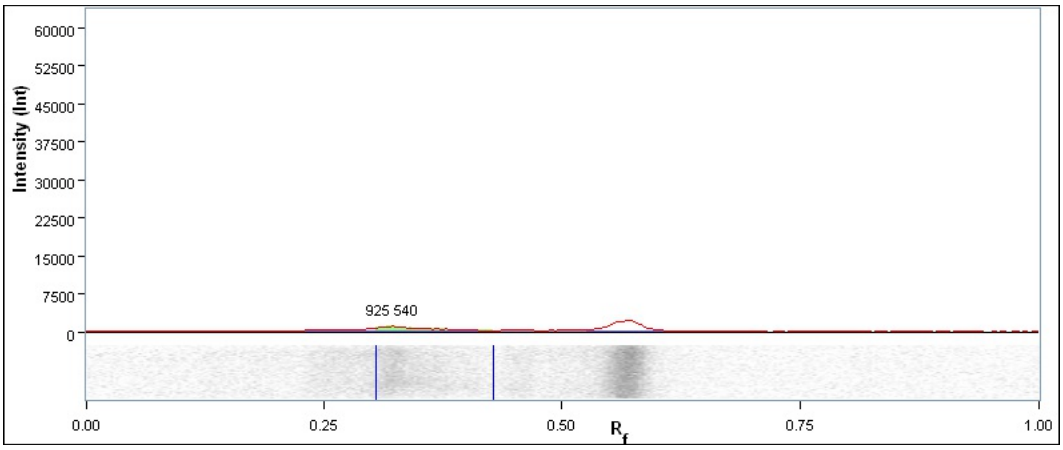

Band No.	Band Label	Mol. Wt. (KDa)	Relative Front	Volume (Int)	Abs. Quant.	Rel. Quant.	Band %	Lane %
1		N/A	0,324	925 540	N/A	N/A	100,0	24,9

Band Detection	Automatically detected bands with sensitivity: Low
Lane Background	Lane background subtracted with disk size: 10
Lane Width	6.51 mm

Lane 4

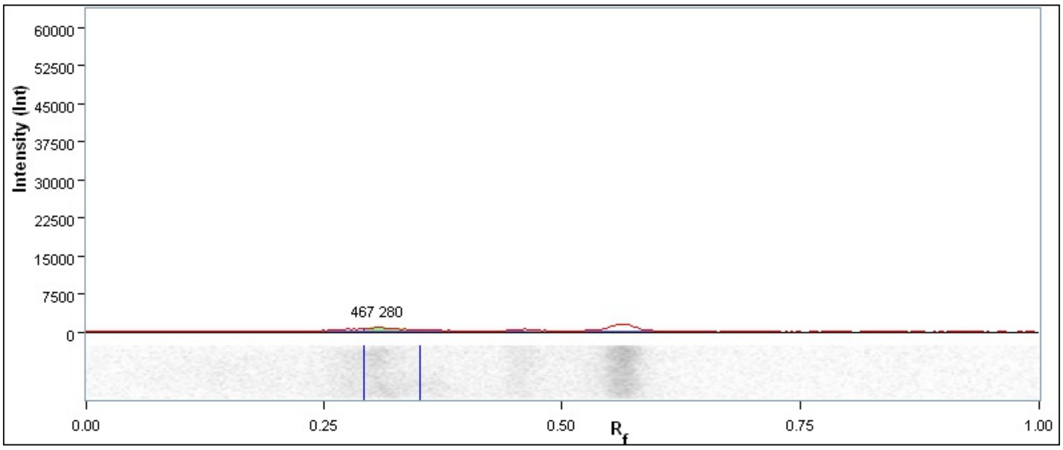

Band No.	Band Label	Mol. Wt. (KDa)	Relative Front	Volume (Int)	Abs. Quant.	Rel. Quant.	Band %	Lane %
1		N/A	0,309	467 280	N/A	N/A	100,0	19,2

Band Detection	Automatically detected bands with sensitivity: Low
Lane Background	Lane background subtracted with disk size: 10
Lane Width	6.51 mm

Lane 5

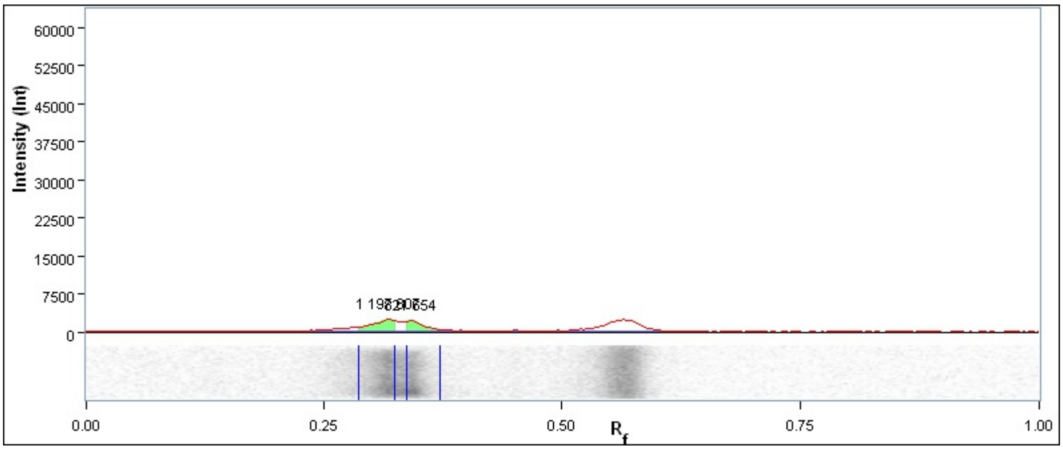

Band No.	Band Label	Mol. Wt. (KDa)	Relative Front	Volume (Int)	Abs. Quant.	Rel. Quant.	Band %	Lane %
1		N/A	0,321	1 197 607	N/A	N/A	59,3	21,6
2		N/A	0,344	821 654	N/A	N/A	40,7	14,8

Band Detection	Automatically detected bands with sensitivity: Low
Lane Background	Lane background subtracted with disk size: 10
Lane Width	6.96 mm

Lane 6

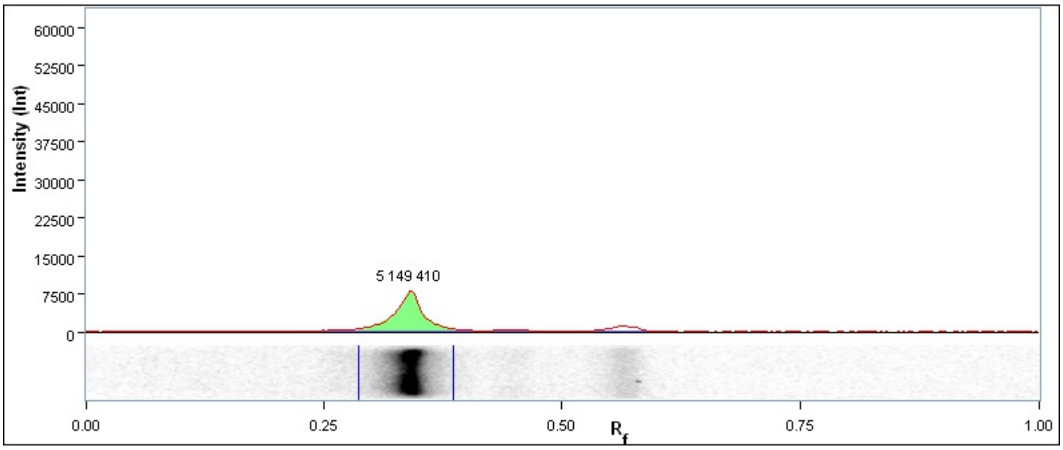

Band No.	Band Label	Mol. Wt. (KDa)	Relative Front	Volume (Int)	Abs. Quant.	Rel. Quant.	Band %	Lane %
1		N/A	0,341	5 149 410	N/A	N/A	100,0	70,4

Band Detection	Automatically detected bands with sensitivity: Low
Lane Background	Lane background subtracted with disk size: 10
Lane Width	7.25 mm

Lane 7

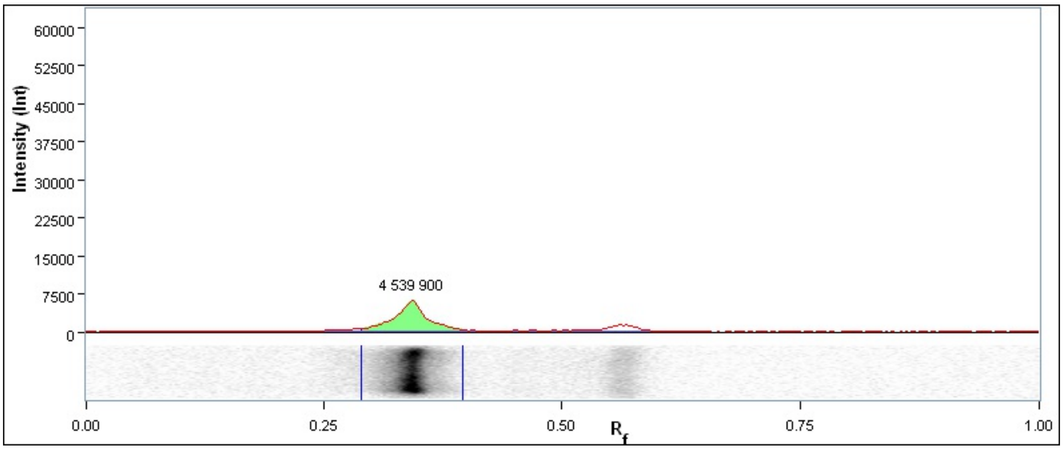

Band No.	Band Label	Mol. Wt. (KDa)	Relative Front	Volume (Int)	Abs. Quant.	Rel. Quant.	Band %	Lane %
1		N/A	0,344	4 539 900	N/A	N/A	100,0	66,6

Band Detection	Automatically detected bands with sensitivity: Low
Lane Background	Lane background subtracted with disk size: 10
Lane Width	7.40 mm

Volume Analysis

No.	Label	Type	Volume (Int)	Adj. Vol. (Int)	Mean Bkgd. (Int)	Abs. Quant.	Rel. Quant.	# of Pixels	Min. Value (Int)	Max. Value (Int)	Mean Value (Int)	Std. Dev.	Area (mm2)
1	U1	Unknown	6 289 876	4 557 568	606,6	N/A	N/A	2 856	0	15 828	2 202,3	2 753,5	62,5
2	U2	Unknown	3 967 212	2 757 264	423,7	N/A	N/A	2 856	0	9 492	1 389,1	1 463,0	62,5
3	U3	Unknown	2 012 872	1 003 735	353,3	N/A	N/A	2 856	0	2 564	704,8	472,7	62,5
4	U4	Unknown	1 555 100	732 048	288,2	N/A	N/A	2 856	0	2 096	544,5	420,9	62,5
5	U5	Unknown	3 435 752	2 426 300	353,4	N/A	N/A	2 856	0	4 784	1 203,0	1 007,8	62,5
6	U6	Unknown	6 138 204	4 827 536	458,9	N/A	N/A	2 856	0	12 048	2 149,2	2 597,2	62,5
7	U7	Unknown	5 470 280	4 302 779	408,8	N/A	N/A	2 856	0	9 960	1 915,4	2 022,1	62,5

Image Report: 2 żel 3 powt tub UMED 2018-01-26 11hr 19min_Exposure_16.8sec

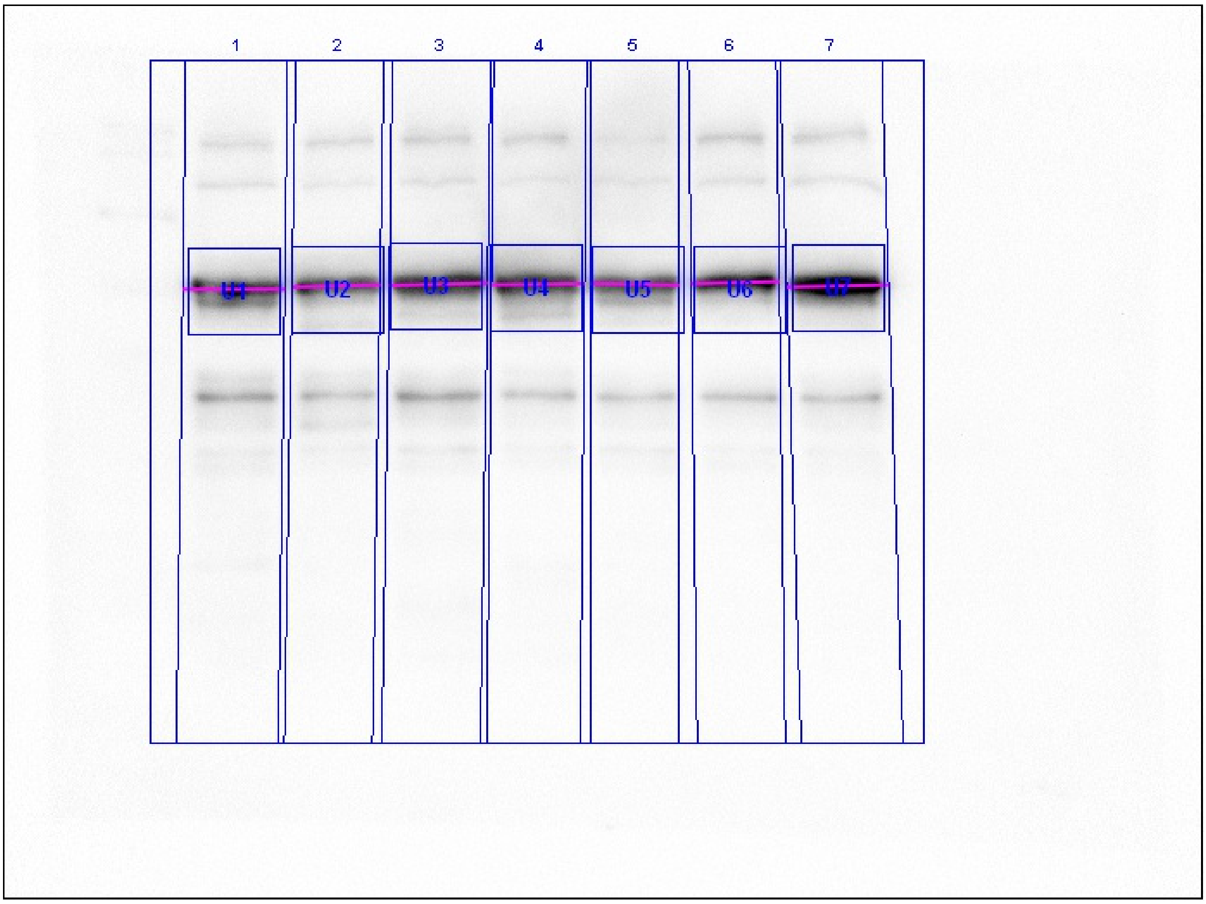

C:\Documents and Settings\Jaga\Pulpit\YKL-39 WB\analiza\żel 2\2 tub\2 żel 3 powt tub UMED 2018-01-26 11hr 19min_Exposure_16.8sec.scn

Acquisition Information

Imager	ChemiDoc™ MP
Exposure Time (sec)	16.800 (Signal Accumulation)
Flat Field	Applied (Lens)
Serial Number	731BR01769
Software Version	5.2.1
Application	Chemi Hi Resolution
Excitation Source	No Illumination
Emission Filter	No Filter
Binning	2x2

Image Information

Acquisition Date	2018-01-26 11:20:08
User Name	UMED
Image Area (mm)	X: 97.0 Y: 72.5
Pixel Size (um)	X: 139.4 Y: 139.4
Data Range (Int)	0 - 51760

Analysis Settings

Detection	Lane detection:<br>Automatically detected lanes with manual adjustments<br><br>Band detection:<br>Automatically detected bands with sensitivity: Low<br>Manually adjusted bands<br><br>Lane Background Subtraction:<br>Lane background subtracted with disk size: 10<br><br>Lane width: Variable
Volume Analysis	Background subtraction method: Local<br>Quantity regression method: Linear

Lane And Band Analysis

Lane 1

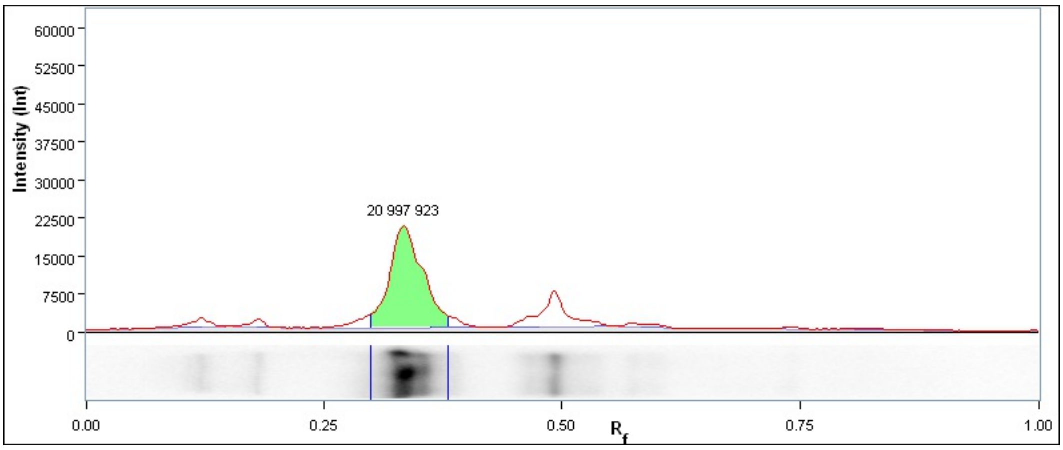

Band No.	Band Label	Mol. Wt. (KDa)	Relative Front	Volume (Int)	Abs. Quant.	Rel. Quant.	Band %	Lane %
1		N/A	0,336	20 997 923	N/A	N/A	100,0	64,2

Band Detection	Automatically detected bands with sensitivity: Low
Lane Background	Lane background subtracted with disk size: 10
Lane Width	8.22 mm

Lane 2

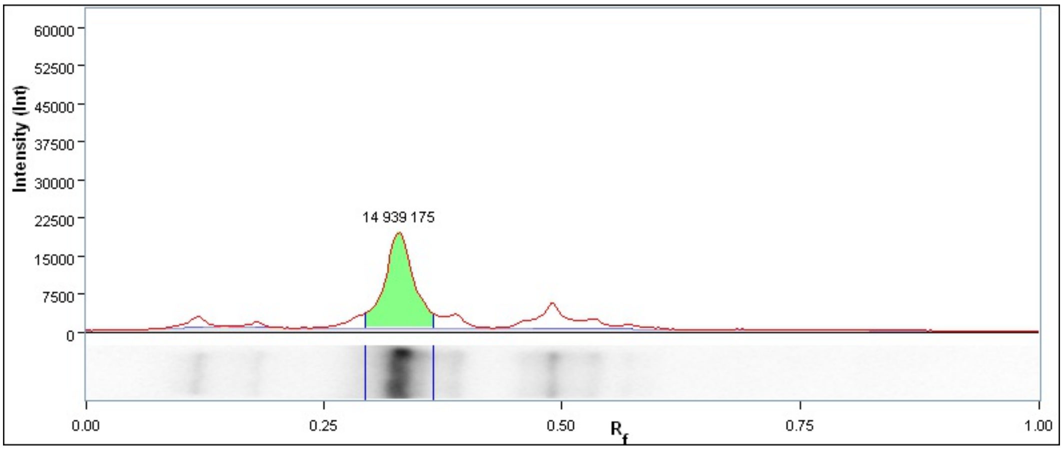

Band No.	Band Label	Mol. Wt. (KDa)	Relative Front	Volume (Int)	Abs. Quant.	Rel. Quant.	Band %	Lane %
1		N/A	0,331	14 939 175	N/A	N/A	100,0	57,8

Band Detection	Automatically detected bands with sensitivity: Low

Lane Background	Lane background subtracted with disk size: 10
Lane Width	7.11 mm

Lane 3

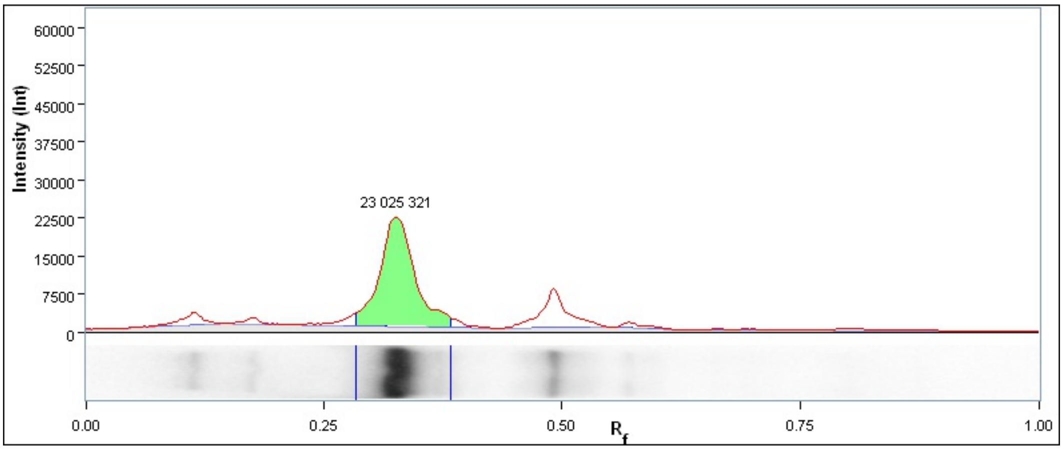

Band No.	Band Label	Mol. Wt. (KDa)	Relative Front	Volume (Int)	Abs. Quant.	Rel. Quant.	Band %	Lane %
1		N/A	0,328	23 025 321	N/A	N/A	100,0	66,0

Band Detection	Automatically detected bands with sensitivity: Low
Lane Background	Lane background subtracted with disk size: 10
Lane Width	7.94 mm

Lane 4

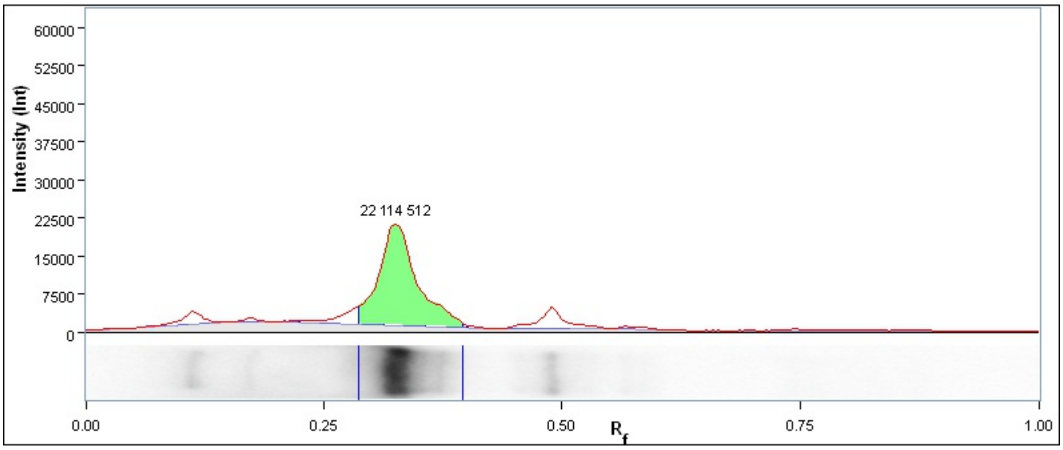

Band No.	Band Label	Mol. Wt. (KDa)	Relative Front	Volume (Int)	Abs. Quant.	Rel. Quant.	Band %	Lane %
1		N/A	0,328	22 114 512	N/A	N/A	100,0	72,9

Band Detection	Automatically detected bands with sensitivity: Low
Lane Background	Lane background subtracted with disk size: 10
Lane Width	7.53 mm

Lane 5

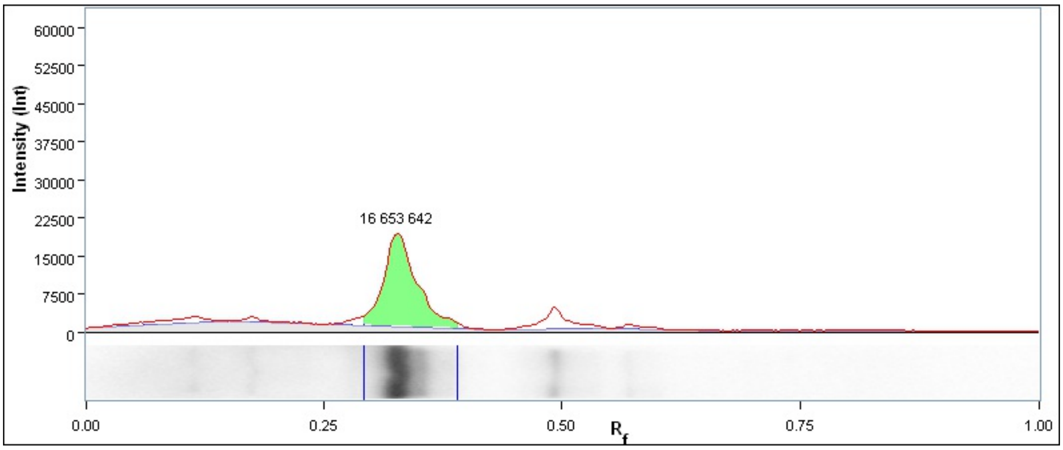

Band No.	Band Label	Mol. Wt. (KDa)	Relative Front	Volume (Int)	Abs. Quant.	Rel. Quant.	Band %	Lane %
1		N/A	0,328	16 653 642	N/A	N/A	100,0	69,8

Band Detection	Automatically detected bands with sensitivity: Low
Lane Background	Lane background subtracted with disk size: 10
Lane Width	7.11 mm

Lane 6

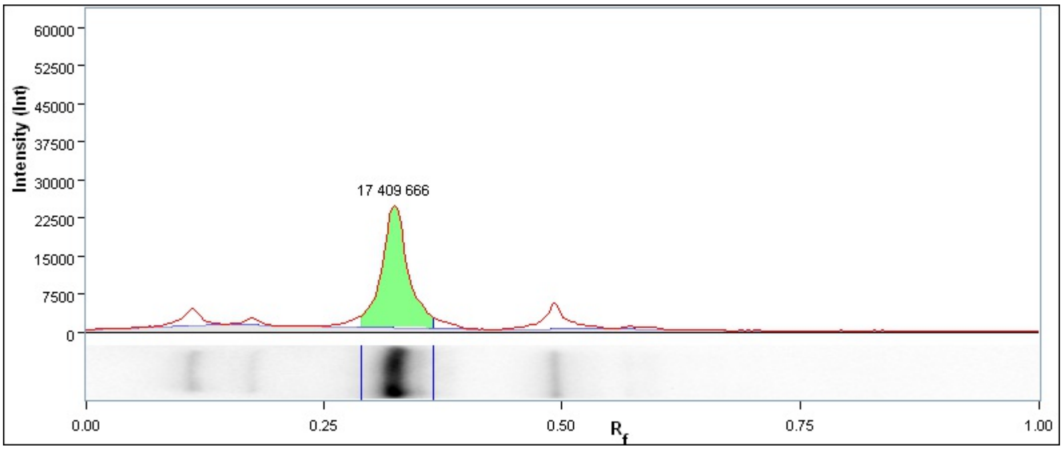

Band No.	Band Label	Mol. Wt. (KDa)	Relative Front	Volume (Int)	Abs. Quant.	Rel. Quant.	Band %	Lane %
1		N/A	0,326	17 409 666	N/A	N/A	100,0	68,0

Band Detection	Automatically detected bands with sensitivity: Low
Lane Background	Lane background subtracted with disk size: 10
Lane Width	7.11 mm

Lane 7

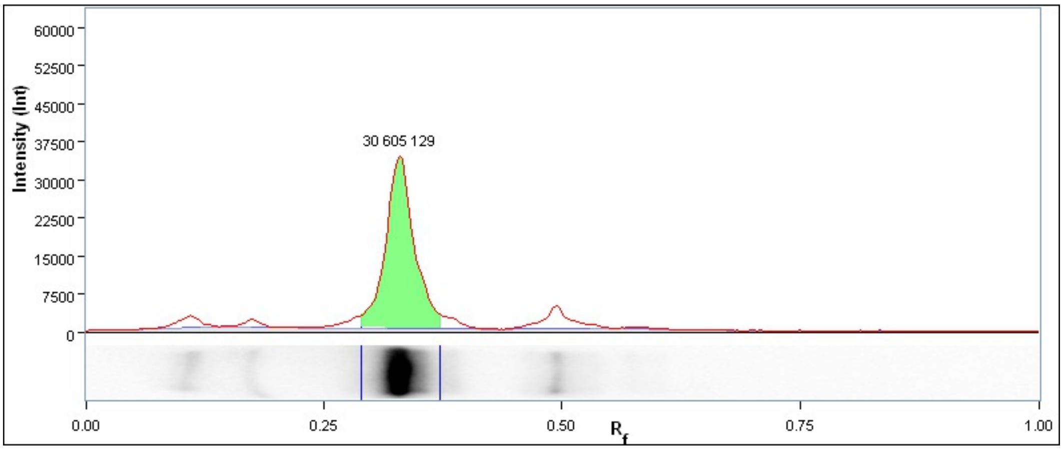

Band No.	Band Label	Mol. Wt. (KDa)	Relative Front	Volume (Int)	Abs. Quant.	Rel. Quant.	Band %	Lane %
1		N/A	0,331	30 605 129	N/A	N/A	100,0	75,9

Band Detection	Automatically detected bands with sensitivity: Low
Lane Background	Lane background subtracted with disk size: 10
Lane Width	8.22 mm

Volume Analysis

No.	Label	Type	Volume (Int)	Adj. Vol. (Int)	Mean Bkgd. (Int)	Abs. Quant.	Rel. Quant.	# of Pixels	Min. Value (Int)	Max. Value (Int)	Mean Value (Int)	Std. Dev.	Area (mm2)
1	U1	Unknown	24 537 896	16 311 236	3 104,4	N/A	N/A	2 650	636	36 384	9 259,6	7 830,0	51,5
2	U2	Unknown	20 461 212	11 925 587	3 221,0	N/A	N/A	2 650	1 056	28 568	7 721,2	6 103,6	51,5
3	U3	Unknown	26 205 560	14 503 968	4 415,7	N/A	N/A	2 650	1 384	28 200	9 888,9	7 832,3	51,5
4	U4	Unknown	26 838 144	15 154 471	4 408,9	N/A	N/A	2 650	1 004	28 972	10 127,6	6 844,6	51,5
5	U5	Unknown	20 666 096	12 707 465	3 003,3	N/A	N/A	2 650	600	24 400	7 798,5	6 283,5	51,5
6	U6	Unknown	21 706 200	14 317 849	2 788,1	N/A	N/A	2 650	544	36 568	8 191,0	8 190,4	51,5
7	U7	Unknown	33 580 124	24 174 340	3 549,4	N/A	N/A	2 650	944	51 760	12 671,7	12 776,0	51,5

Image Report: 2 żel 3 powt YKL39 UMED 2018-01-24 11hr 50min_Exposure_60.0sec

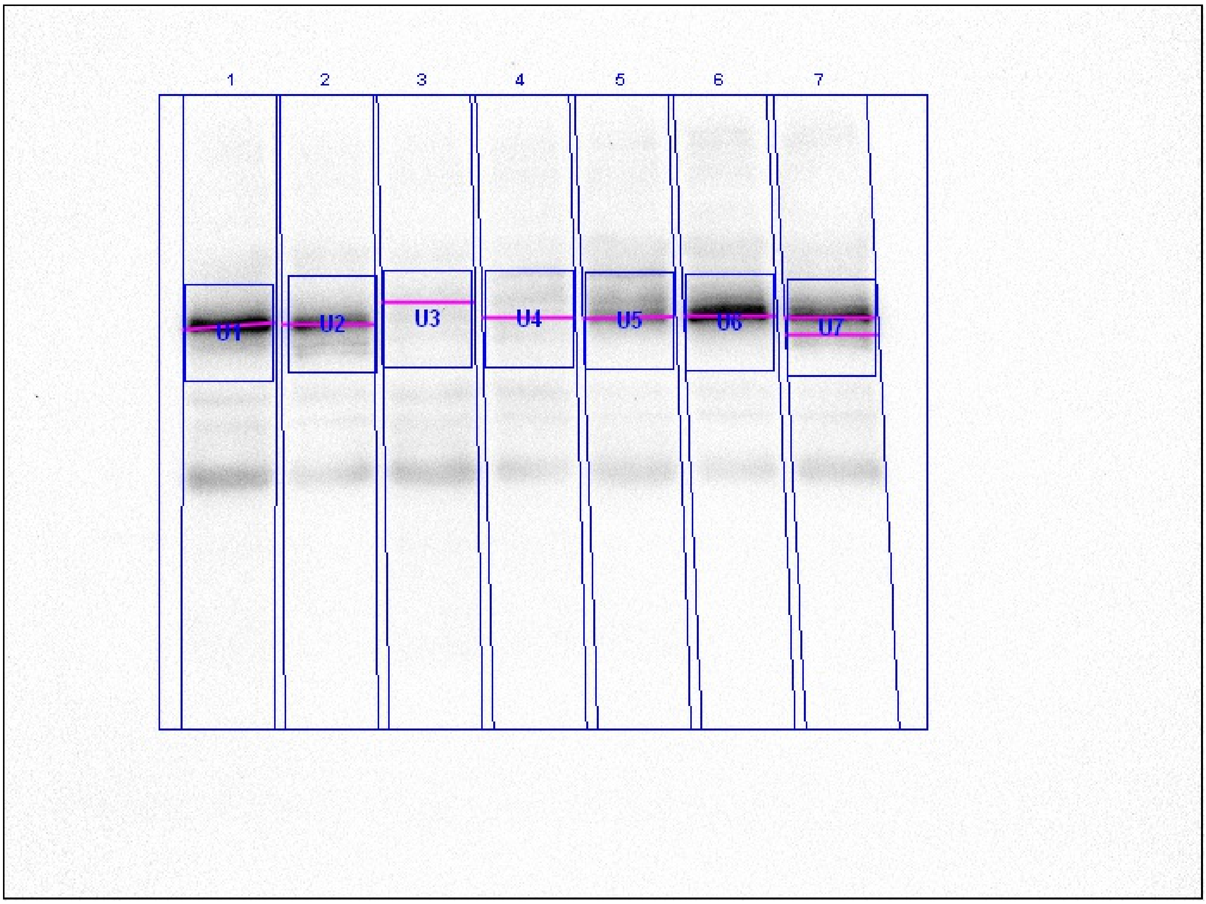

C:\Documents and Settings\Jaga\Pulpit\YKL-39 WB\analiza\żel 2\2 YKL39\2 żel 3 powt YKL39 UMED 2018-01-24 11hr 50min_Exposure_60.0sec.scn

Acquisition Information

Imager	ChemiDoc™ MP
Exposure Time (sec)	60.000 (Signal Accumulation)
Flat Field	Applied (Lens)
Serial Number	731BR01769
Software Version	5.2.1
Application	Chemi Hi Resolution
Excitation Source	No Illumination
Emission Filter	No Filter
Binning	2x2

Image Information

Acquisition Date	2018-01-24 11:51:33
User Name	UMED
Image Area (mm)	X: 97.0 Y: 72.5
Pixel Size (um)	X: 139.4 Y: 139.4
Data Range (Int)	0 - 29124

Analysis Settings

Detection	Lane detection:<br>Automatically detected lanes with manual adjustments<br><br>Band detection:<br>Automatically detected bands with sensitivity: Low<br>Manually adjusted bands<br><br>Lane Background Subtraction:<br>Lane background subtracted with disk size: 10<br><br>Lane width: 7.53 mm
Volume Analysis	Background subtraction method: Local<br>Quantity regression method: Linear

Lane And Band Analysis

Lane 1

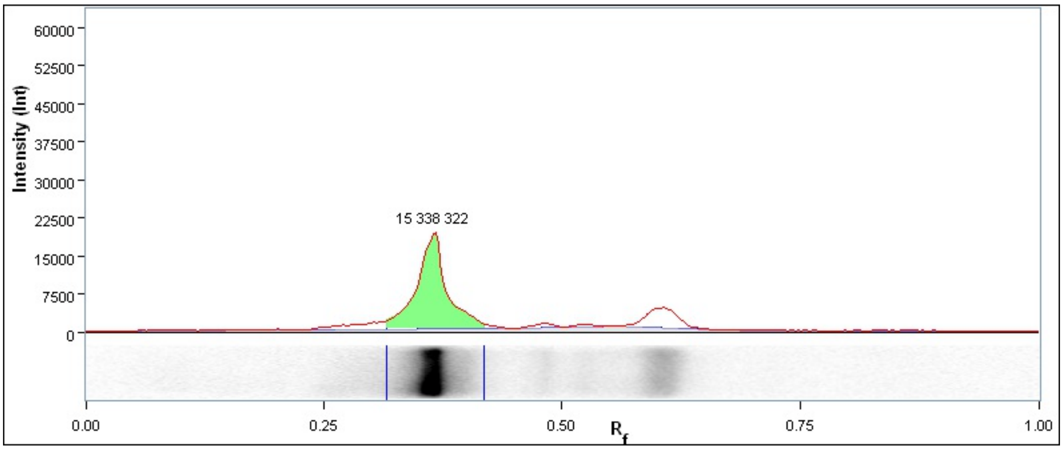

Band No.	Band Label	Mol. Wt. (KDa)	Relative Front	Volume (Int)	Abs. Quant.	Rel. Quant.	Band %	Lane %
1		N/A	0,366	15 338 322	N/A	N/A	100,0	67,8

Band Detection	Automatically detected bands with sensitivity: Low
Lane Background	Lane background subtracted with disk size: 10
Lane Width	7.53 mm

Lane 2

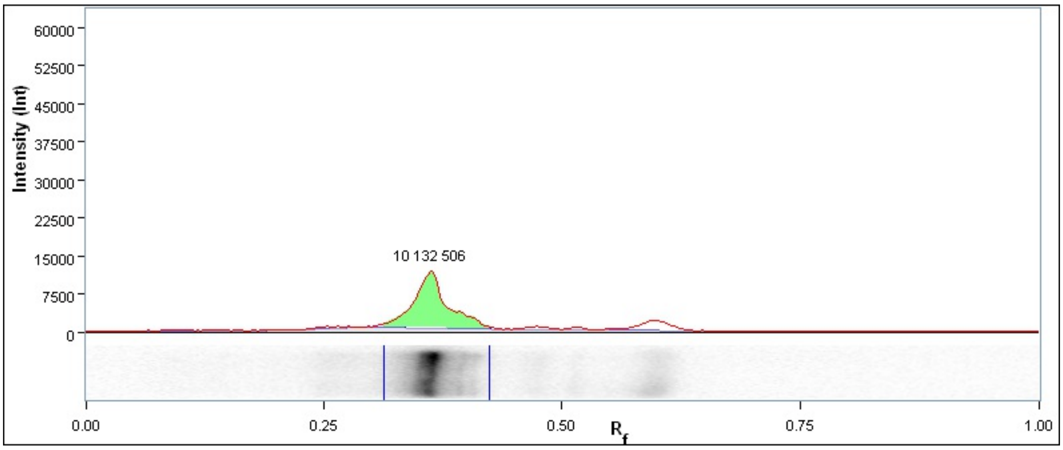

Band No.	Band Label	Mol. Wt. (KDa)	Relative Front	Volume (Int)	Abs. Quant.	Rel. Quant.	Band %	Lane %
1		N/A	0,363	10 132 506	N/A	N/A	100,0	72,6

Band Detection	Automatically detected bands with sensitivity: Low

Lane Background	Lane background subtracted with disk size: 10
Lane Width	7.53 mm

Lane 3

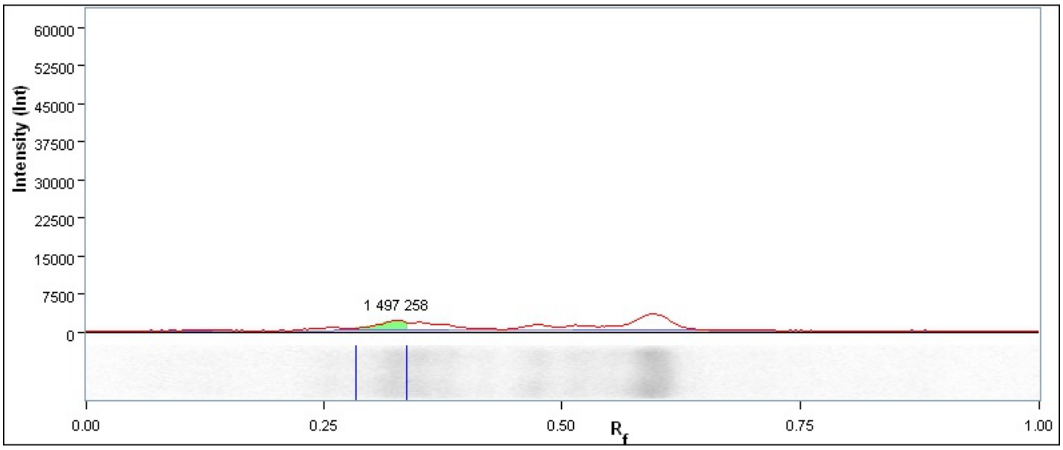

Band No.	Band Label	Mol. Wt. (KDa)	Relative Front	Volume (Int)	Abs. Quant.	Rel. Quant.	Band %	Lane %
1		N/A	0,328	1 497 258	N/A	N/A	100,0	15,6

Band Detection	Automatically detected bands with sensitivity: Low
Lane Background	Lane background subtracted with disk size: 10
Lane Width	7.53 mm

Lane 4

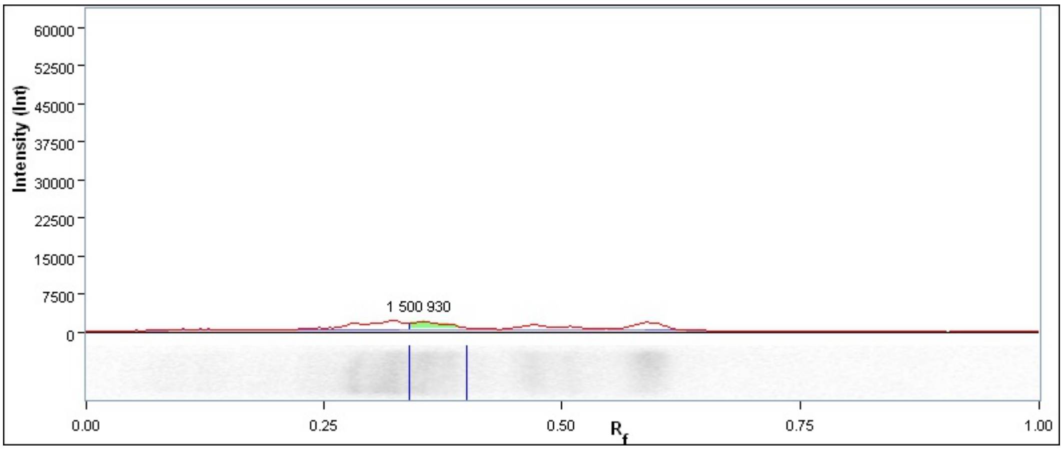

Band No.	Band Label	Mol. Wt. (KDa)	Relative Front	Volume (Int)	Abs. Quant.	Rel. Quant.	Band %	Lane %
1		N/A	0,352	1 500 930	N/A	N/A	100,0	20,4

Band Detection	Automatically detected bands with sensitivity: Low
Lane Background	Lane background subtracted with disk size: 10
Lane Width	7.53 mm

Lane 5

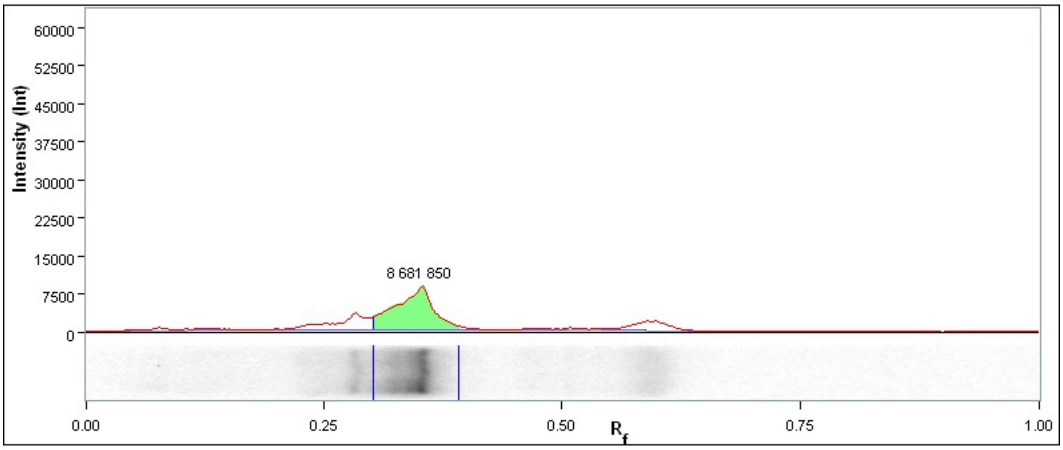

Band No.	Band Label	Mol. Wt. (KDa)	Relative Front	Volume (Int)	Abs. Quant.	Rel. Quant.	Band %	Lane %
1		N/A	0,352	8 681 850	N/A	N/A	100,0	56,1

Band Detection	Automatically detected bands with sensitivity: Low
Lane Background	Lane background subtracted with disk size: 10
Lane Width	7.53 mm

Lane 6

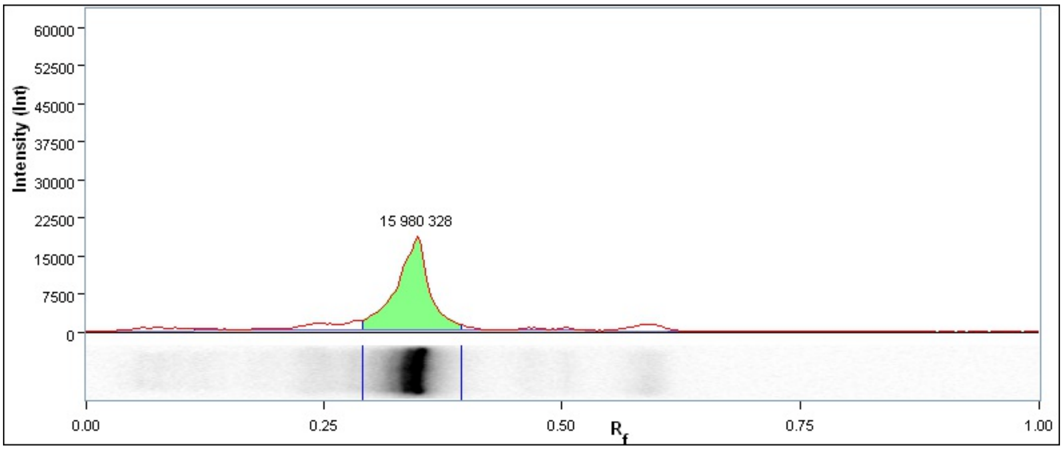

Band No.	Band Label	Mol. Wt. (KDa)	Relative Front	Volume (Int)	Abs. Quant.	Rel. Quant.	Band %	Lane %
1		N/A	0,350	15 980 328	N/A	N/A	100,0	73,1

Band Detection	Automatically detected bands with sensitivity: Low
Lane Background	Lane background subtracted with disk size: 10
Lane Width	7.53 mm

Lane 7

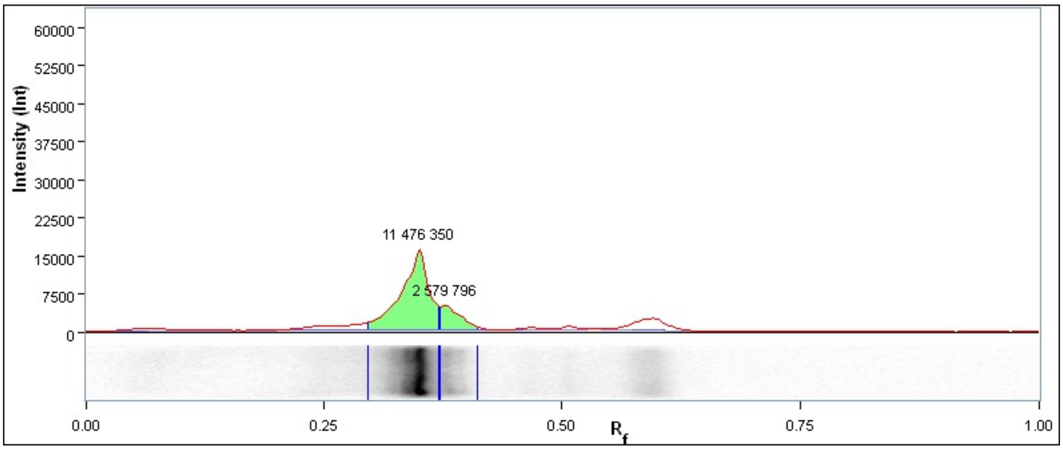

Band No.	Band Label	Mol. Wt. (KDa)	Relative Front	Volume (Int)	Abs. Quant.	Rel. Quant.	Band %	Lane %
1		N/A	0,352	11 476 350	N/A	N/A	81,6	57,8
2		N/A	0,379	2 579 796	N/A	N/A	18,4	13,0

Band Detection	Automatically detected bands with sensitivity: Low
Lane Background	Lane background subtracted with disk size: 10
Lane Width	7.53 mm

Volume Analysis

No.	Label	Type	Volume (Int)	Adj. Vol. (Int)	Mean Bkgd. (Int)	Abs. Quant.	Rel. Quant.	# of Pixels	Min. Value (Int)	Max. Value (Int)	Mean Value (Int)	Std. Dev.	Area (mm2)
1	U1	Unknown	18 005 184	12 768 171	1 833,7	N/A	N/A	2 856	0	29 124	6 304,3	6 321,2	55,5
2	U2	Unknown	12 832 784	9 512 959	1 162,4	N/A	N/A	2 856	0	20 908	4 493,3	3 949,0	55,5
3	U3	Unknown	4 589 828	1 958 011	921,5	N/A	N/A	2 856	0	8 476	1 607,1	747,0	55,5
4	U4	Unknown	5 238 512	2 637 089	910,9	N/A	N/A	2 856	0	4 432	1 834,2	858,0	55,5
5	U5	Unknown	11 444 108	6 987 752	1 560,3	N/A	N/A	2 856	0	14 984	4 007,0	3 023,6	55,5
6	U6	Unknown	17 756 144	12 884 070	1 705,9	N/A	N/A	2 856	0	26 760	6 217,1	6 373,3	55,5
7	U7	Unknown	15 453 284	10 452 402	1 751,0	N/A	N/A	2 856	0	25 216	5 410,8	4 838,0	55,5

Image Report: 3 zel 2 powt tub UMED 2018-03-01 10hr 48min_Exposure_16.8sec

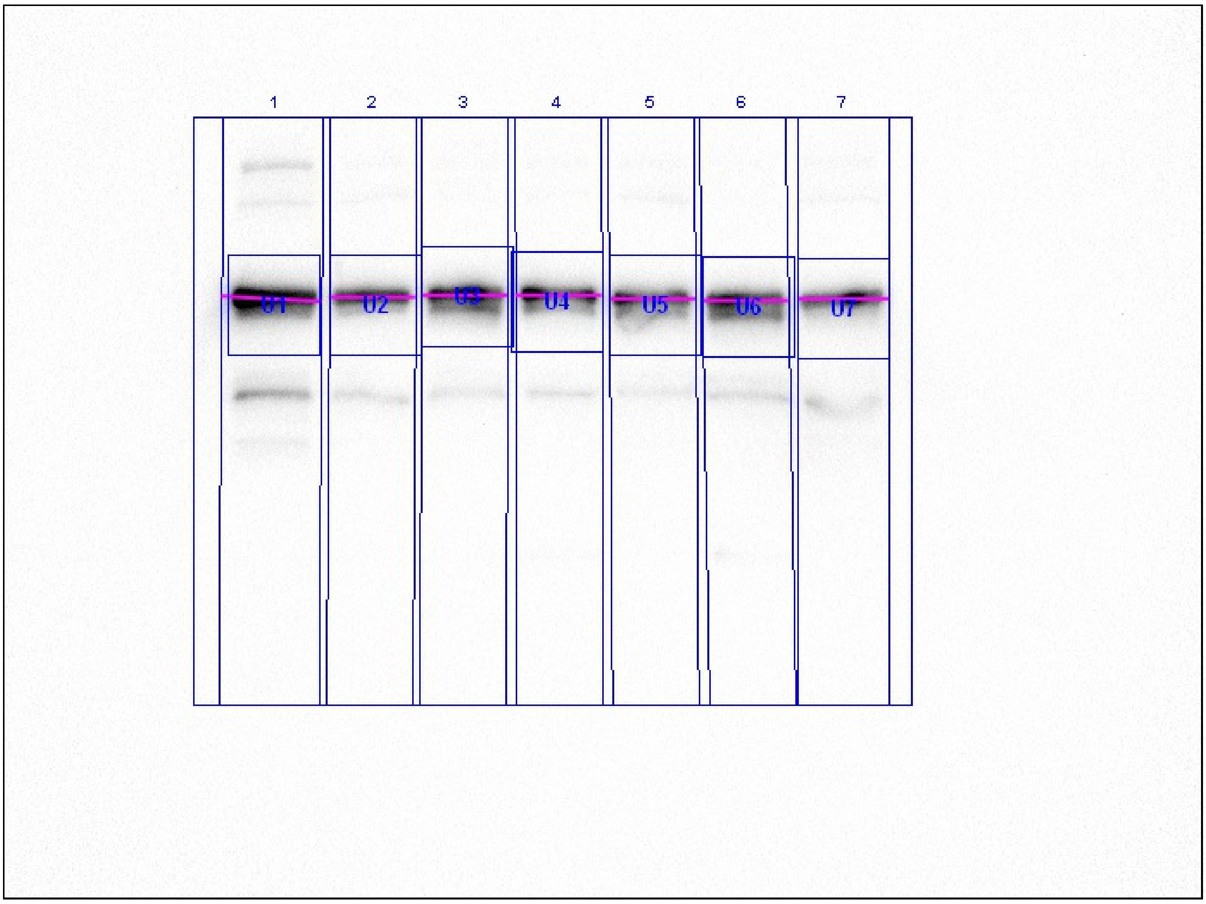

C:\Documents and Settings\Jaga\Pulpit\YKL-39 WB\analiza\żel 3\3 tub\3 zel 2 powt tub UMED 2018-03-01 10hr 48min_Exposure_16.8sec.scn

---

Acquisition Information

Imager	ChemiDoc™ MP
Exposure Time (sec)	16.800 (Signal Accumulation)
Flat Field	Applied (Lens)
Serial Number	731BR01769
Software Version	5.2.1
Application	Chemi Hi Resolution
Excitation Source	No Illumination
Emission Filter	No Filter
Binning	2x2

Image Information

Acquisition Date	2018-03-01 10:49:03
User Name	UMED
Image Area (mm)	X: 102.0 Y: 76.2
Pixel Size (um)	X: 146.6 Y: 146.6
Data Range (Int)	0 - 39152

Analysis Settings

Detection	Lane detection:<br>Automatically detected lanes with manual adjustments<br><br>Band detection:<br>Automatically detected bands with sensitivity: Low<br>Manually adjusted bands<br><br>Lane Background Subtraction:<br>Lane background subtracted with disk size: 10<br><br>Lane width: Variable
Volume Analysis	Background subtraction method: Local<br>Quantity regression method: Linear

Lane And Band Analysis

Lane 1

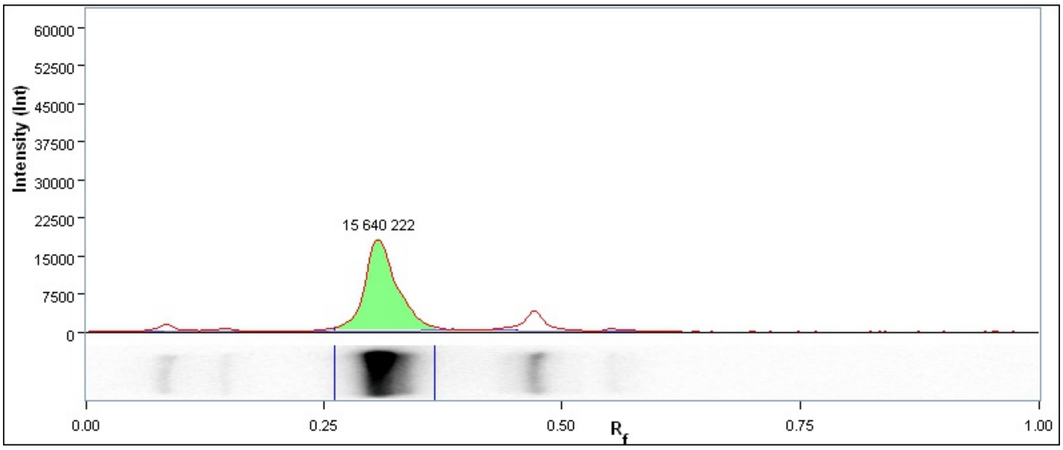

Band No.	Band Label	Mol. Wt. (KDa)	Relative Front	Volume (Int)	Abs. Quant.	Rel. Quant.	Band %	Lane %
1		N/A	0,310	15 640 222	N/A	N/A	100,0	78,5

Band Detection	Automatically detected bands with sensitivity: Low
Lane Background	Lane background subtracted with disk size: 10
Lane Width	8.50 mm

Lane 2

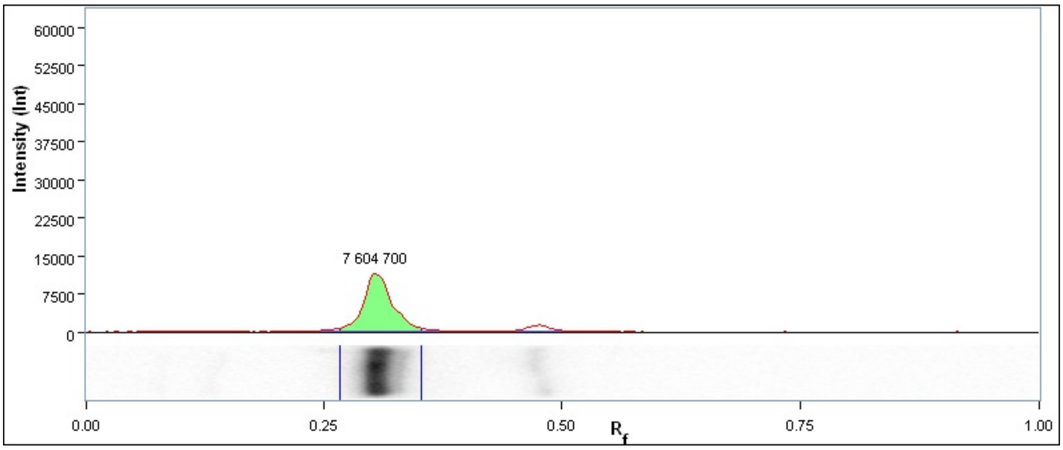

Band No.	Band Label	Mol. Wt. (KDa)	Relative Front	Volume (Int)	Abs. Quant.	Rel. Quant.	Band %	Lane %
1		N/A	0,307	7 604 700	N/A	N/A	100,0	79,7

Band Detection	Automatically detected bands with sensitivity: Low

Lane Background	Lane background subtracted with disk size: 10
Lane Width	7.33 mm

Lane 3

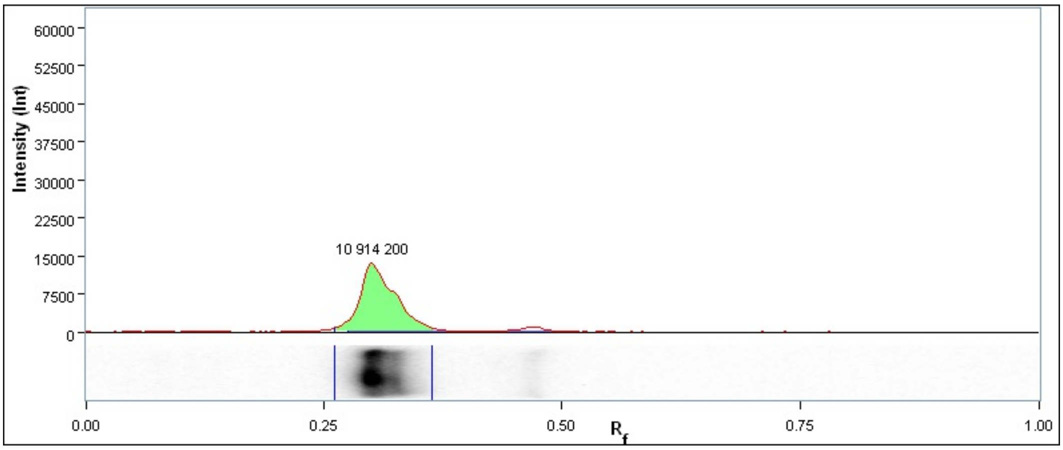

Band No.	Band Label	Mol. Wt. (KDa)	Relative Front	Volume (Int)	Abs. Quant.	Rel. Quant.	Band %	Lane %
1		N/A	0,304	10 914 200	N/A	N/A	100,0	88,2

Band Detection	Automatically detected bands with sensitivity: Low
Lane Background	Lane background subtracted with disk size: 10
Lane Width	7.33 mm

Lane 4

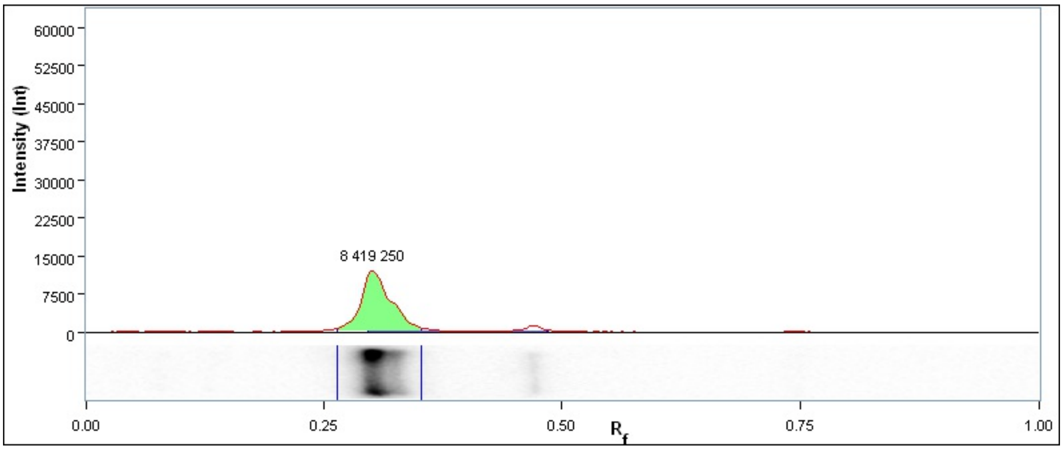

Band No.	Band Label	Mol. Wt. (KDa)	Relative Front	Volume (Int)	Abs. Quant.	Rel. Quant.	Band %	Lane %
1		N/A	0,304	8 419 250	N/A	N/A	100,0	85,0

Band Detection	Automatically detected bands with sensitivity: Low
Lane Background	Lane background subtracted with disk size: 10
Lane Width	7.33 mm

Lane 5

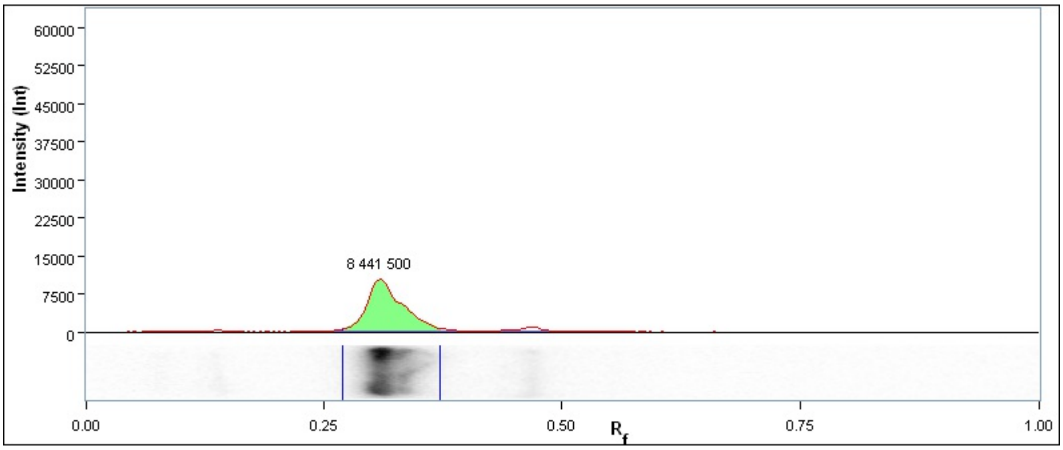

Band No.	Band Label	Mol. Wt. (KDa)	Relative Front	Volume (Int)	Abs. Quant.	Rel. Quant.	Band %	Lane %
1		N/A	0,310	8 441 500	N/A	N/A	100,0	85,4

Band Detection	Automatically detected bands with sensitivity: Low
Lane Background	Lane background subtracted with disk size: 10
Lane Width	7.33 mm

Lane 6

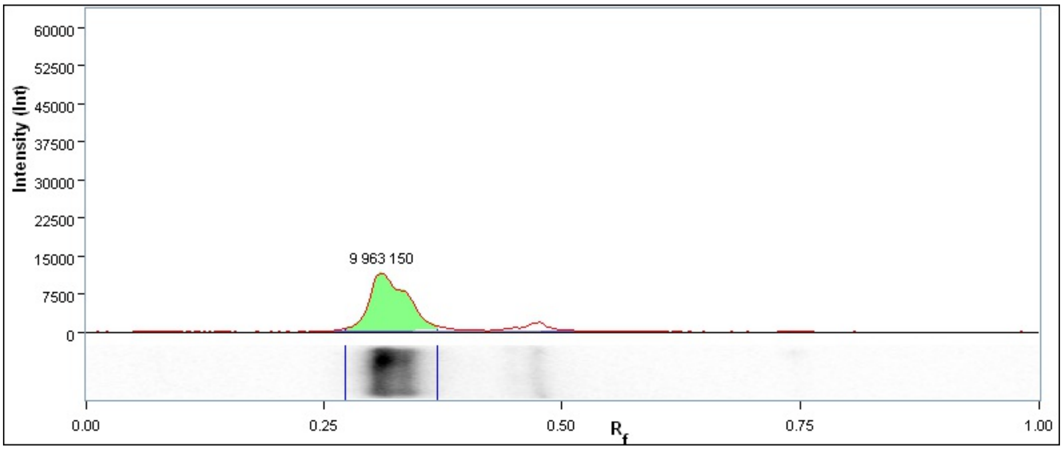

Band No.	Band Label	Mol. Wt. (KDa)	Relative Front	Volume (Int)	Abs. Quant.	Rel. Quant.	Band %	Lane %
1		N/A	0,313	9 963 150	N/A	N/A	100,0	82,9

Band Detection	Automatically detected bands with sensitivity: Low
Lane Background	Lane background subtracted with disk size: 10
Lane Width	7.33 mm

Lane 7

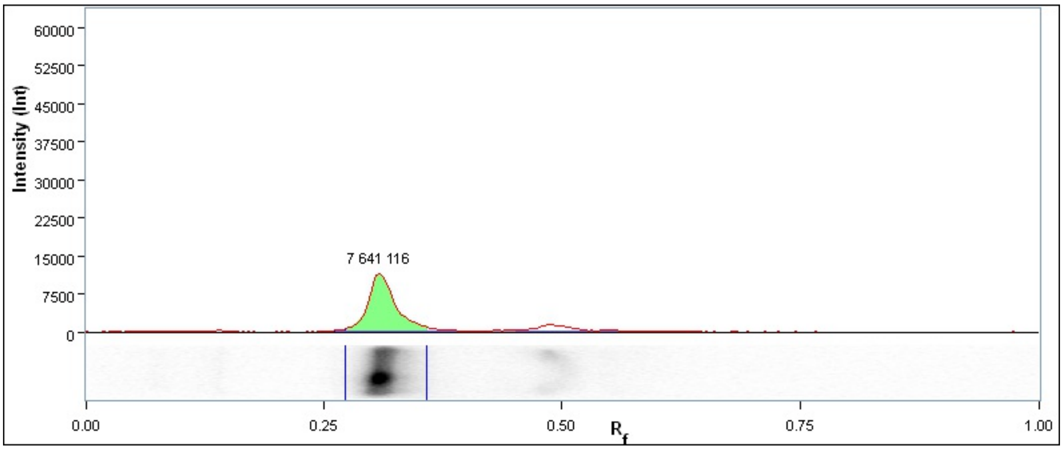

Band No.	Band Label	Mol. Wt. (KDa)	Relative Front	Volume (Int)	Abs. Quant.	Rel. Quant.	Band %	Lane %
1		N/A	0,310	7 641 116	N/A	N/A	100,0	76,7

Band Detection	Automatically detected bands with sensitivity: Low
Lane Background	Lane background subtracted with disk size: 10
Lane Width	7.77 mm

Volume Analysis

No.	Label	Type	Volume (Int)	Adj. Vol. (Int)	Mean Bkgd. (Int)	Abs. Quant.	Rel. Quant.	# of Pixels	Min. Value (Int)	Max. Value (Int)	Mean Value (Int)	Std. Dev.	Area (mm2)
1	U1	Unknown	16 815 112	13 682 951	1 018,9	N/A	N/A	3 074	0	39 152	5 470,1	7 217,4	66,0
2	U2	Unknown	8 656 812	6 770 355	613,7	N/A	N/A	3 074	0	16 248	2 816,1	3 875,5	66,0
3	U3	Unknown	11 855 336	9 985 854	608,2	N/A	N/A	3 074	0	22 620	3 856,6	4 898,2	66,0
4	U4	Unknown	9 426 984	7 412 507	655,3	N/A	N/A	3 074	0	24 540	3 066,7	4 217,7	66,0
5	U5	Unknown	9 370 860	7 349 800	657,5	N/A	N/A	3 074	0	17 440	3 048,4	3 641,7	66,0
6	U6	Unknown	11 280 604	9 275 649	652,2	N/A	N/A	3 074	0	20 516	3 669,7	4 337,3	66,0
7	U7	Unknown	8 645 472	7 025 882	526,9	N/A	N/A	3 074	0	22 912	2 812,5	3 958,9	66,0

Image Report: 3 żel 2 powt YKL39 UMED 2018-02-28 11hr 01min_Exposure_60.0sec

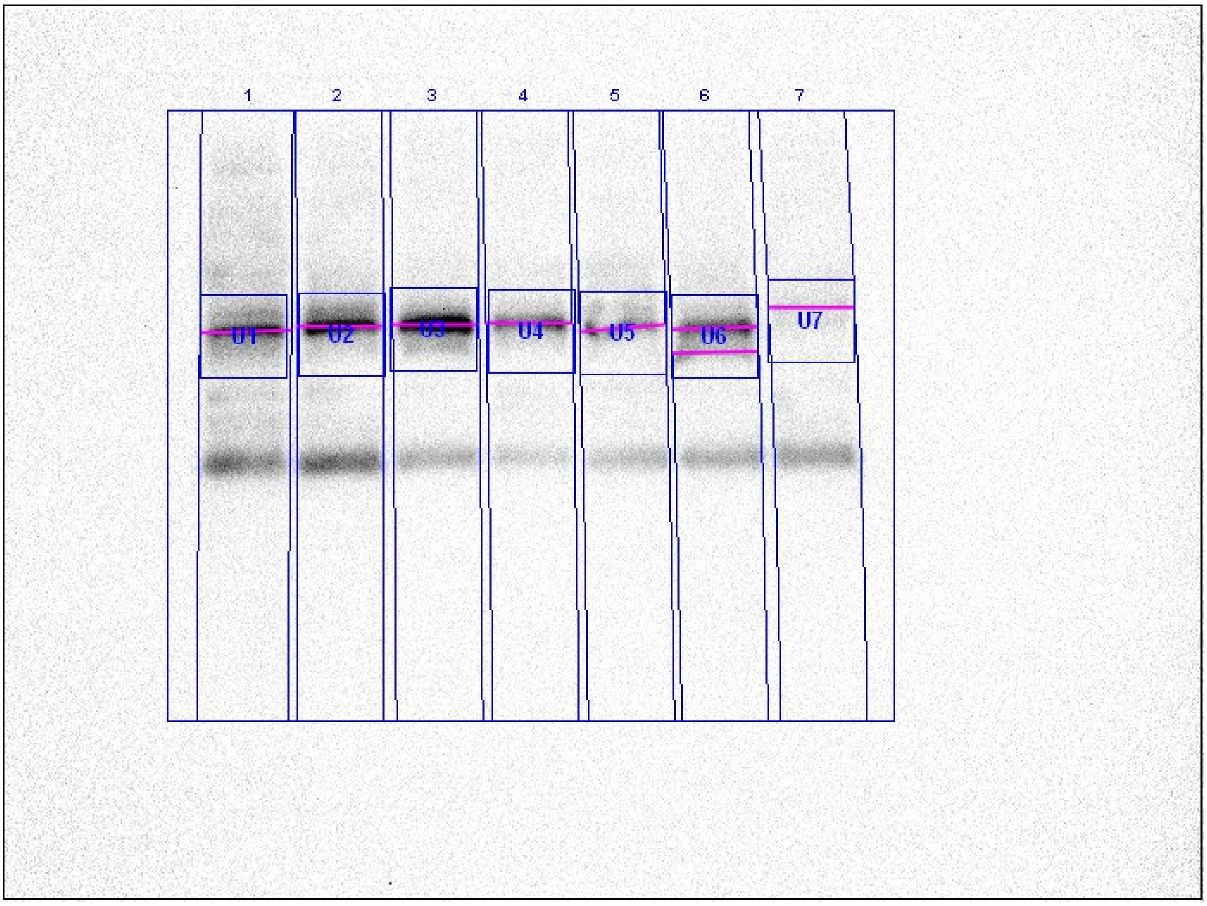

C:\Documents and Settings\Jaga\Pulpit\YKL-39 WB\analiza\żel 3\3 YKL39\3 żel 2 powt YKL39 UMED 2018-02-28 11hr 01min_Exposure_60.0sec.scn

Acquisition Information

Imager	ChemiDoc™ MP
Exposure Time (sec)	60.000 (Signal Accumulation)
Flat Field	Applied (Lens)
Serial Number	731BR01769
Software Version	5.2.1
Application	Chemi Hi Resolution
Excitation Source	No Illumination
Emission Filter	No Filter
Binning	2x2

Image Information

Acquisition Date	2018-02-28 11:03:00
User Name	UMED
Image Area (mm)	X: 103.0 Y: 77.0
Pixel Size (um)	X: 148.0 Y: 148.0
Data Range (Int)	0 - 13932

Analysis Settings

Detection	Lane detection:<br>Automatically detected lanes with manual adjustments<br><br>Band detection:<br>Automatically detected bands with sensitivity: Low<br>Manually adjusted bands<br><br>Lane Background Subtraction:<br>Lane background subtracted with disk size: 10<br><br>Lane width: Variable
Volume Analysis	Background subtraction method: Local<br>Quantity regression method: Linear

Lane And Band Analysis

Lane 1

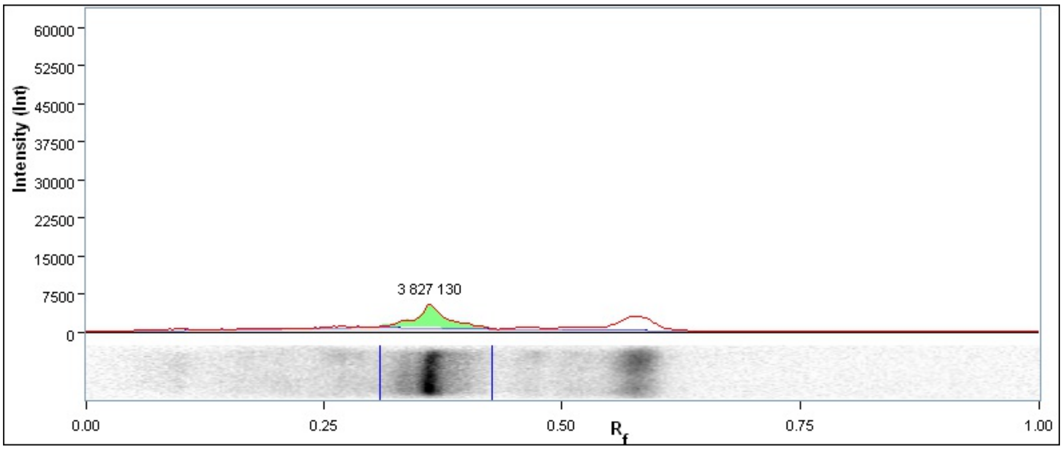

Band No.	Band Label	Mol. Wt. (KDa)	Relative Front	Volume (Int)	Abs. Quant.	Rel. Quant.	Band %	Lane %
1		N/A	0,363	3 827 130	N/A	N/A	100,0	43,8

Band Detection	Automatically detected bands with sensitivity: Low
Lane Background	Lane background subtracted with disk size: 10
Lane Width	7.84 mm

Lane 2

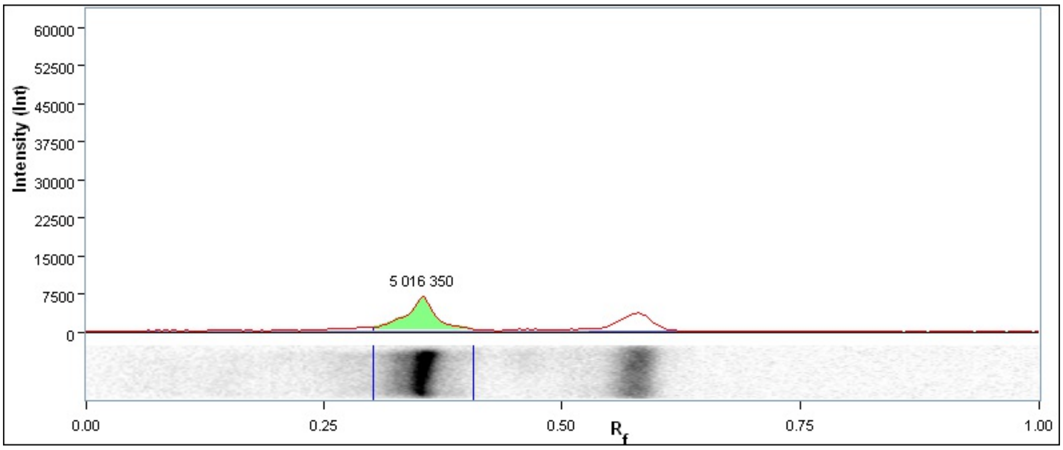

Band No.	Band Label	Mol. Wt. (KDa)	Relative Front	Volume (Int)	Abs. Quant.	Rel. Quant.	Band %	Lane %
1		N/A	0,355	5 016 350	N/A	N/A	100,0	49,0

Band Detection	Automatically detected bands with sensitivity: Low

Lane Background	Lane background subtracted with disk size: 10
Lane Width	7.40 mm

Lane 3

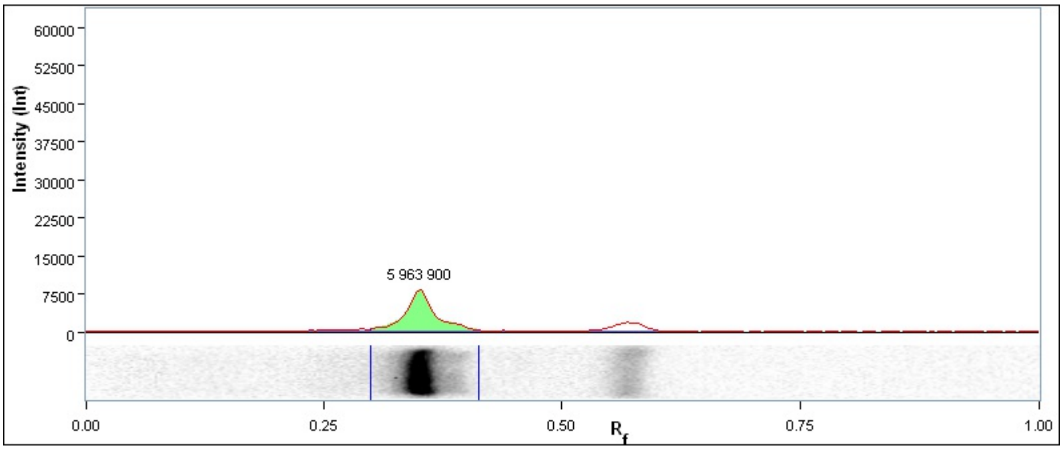

Band No.	Band Label	Mol. Wt. (KDa)	Relative Front	Volume (Int)	Abs. Quant.	Rel. Quant.	Band %	Lane %
1		N/A	0,352	5 963 900	N/A	N/A	100,0	68,4

Band Detection	Automatically detected bands with sensitivity: Low
Lane Background	Lane background subtracted with disk size: 10
Lane Width	7.40 mm

Lane 4

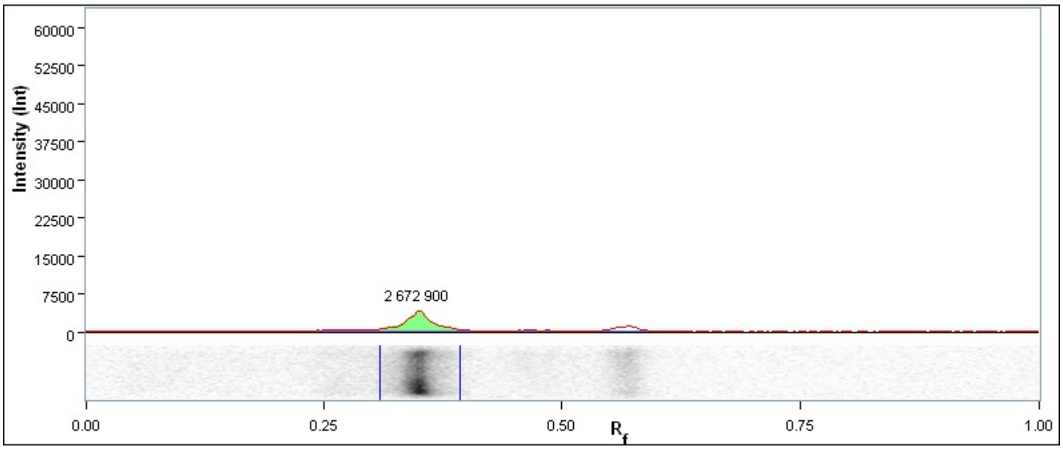

Band No.	Band Label	Mol. Wt. (KDa)	Relative Front	Volume (Int)	Abs. Quant.	Rel. Quant.	Band %	Lane %
1		N/A	0,349	2 672 900	N/A	N/A	100,0	58,2

Band Detection	Automatically detected bands with sensitivity: Low
Lane Background	Lane background subtracted with disk size: 10
Lane Width	7.40 mm

Lane 5

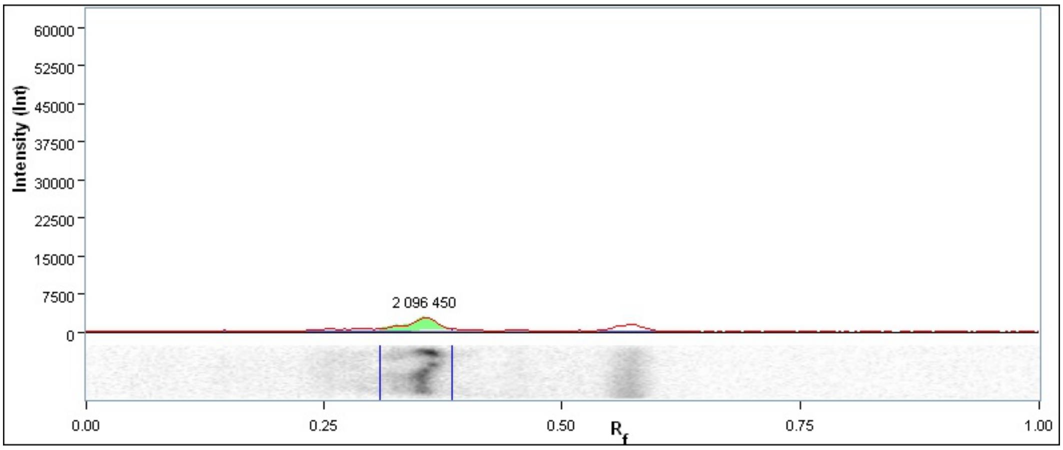

Band No.	Band Label	Mol. Wt. (KDa)	Relative Front	Volume (Int)	Abs. Quant.	Rel. Quant.	Band %	Lane %
1		N/A	0,358	2 096 450	N/A	N/A	100,0	44,1

Band Detection	Automatically detected bands with sensitivity: Low
Lane Background	Lane background subtracted with disk size: 10
Lane Width	7.40 mm

Lane 6

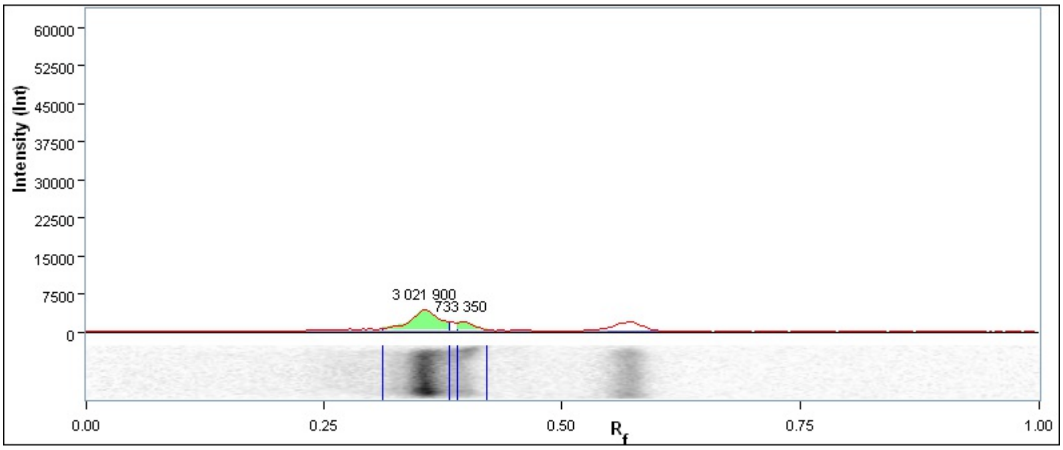

Band No.	Band Label	Mol. Wt. (KDa)	Relative Front	Volume (Int)	Abs. Quant.	Rel. Quant.	Band %	Lane %
1		N/A	0,358	3 021 900	N/A	N/A	80,5	45,5
2		N/A	0,397	733 350	N/A	N/A	19,5	11,1

Band Detection	Automatically detected bands with sensitivity: Low
Lane Background	Lane background subtracted with disk size: 10
Lane Width	7.40 mm

Lane 7

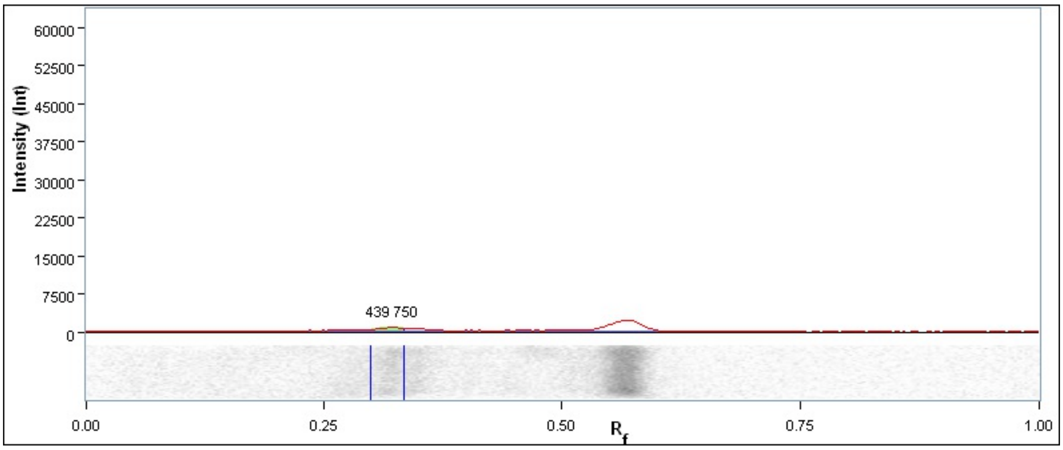

Band No.	Band Label	Mol. Wt. (KDa)	Relative Front	Volume (Int)	Abs. Quant.	Rel. Quant.	Band %	Lane %
1		N/A	0,324	439 750	N/A	N/A	100,0	10,6

Band Detection	Automatically detected bands with sensitivity: Low
Lane Background	Lane background subtracted with disk size: 10
Lane Width	7.40 mm

Volume Analysis

No.	Label	Type	Volume (Int)	Adj. Vol. (Int)	Mean Bkgd. (Int)	Abs. Quant.	Rel. Quant.	# of Pixels	Min. Value (Int)	Max. Value (Int)	Mean Value (Int)	Std. Dev.	Area (mm2)
1	U1	Unknown	5 977 176	3 923 592	855,7	N/A	N/A	2 400	0	9 416	2 490,5	1 709,1	52,6
2	U2	Unknown	6 020 140	4 318 924	708,8	N/A	N/A	2 400	0	11 560	2 508,4	2 358,1	52,6
3	U3	Unknown	6 625 432	5 175 832	604,0	N/A	N/A	2 400	0	13 260	2 760,6	2 902,1	52,6
4	U4	Unknown	3 414 764	2 363 756	437,9	N/A	N/A	2 400	0	7 476	1 422,8	1 406,4	52,6
5	U5	Unknown	3 038 504	1 899 752	474,5	N/A	N/A	2 400	0	7 108	1 266,0	1 112,8	52,6
6	U6	Unknown	4 810 780	3 458 380	563,5	N/A	N/A	2 400	0	7 436	2 004,5	1 494,4	52,6
7	U7	Unknown	1 652 068	567 028	452,1	N/A	N/A	2 400	0	2 508	688,4	461,8	52,6

Image Report: 3 żel 1 powt tub UMED 2018-03-07 11hr 10min_Exposure_16.8sec

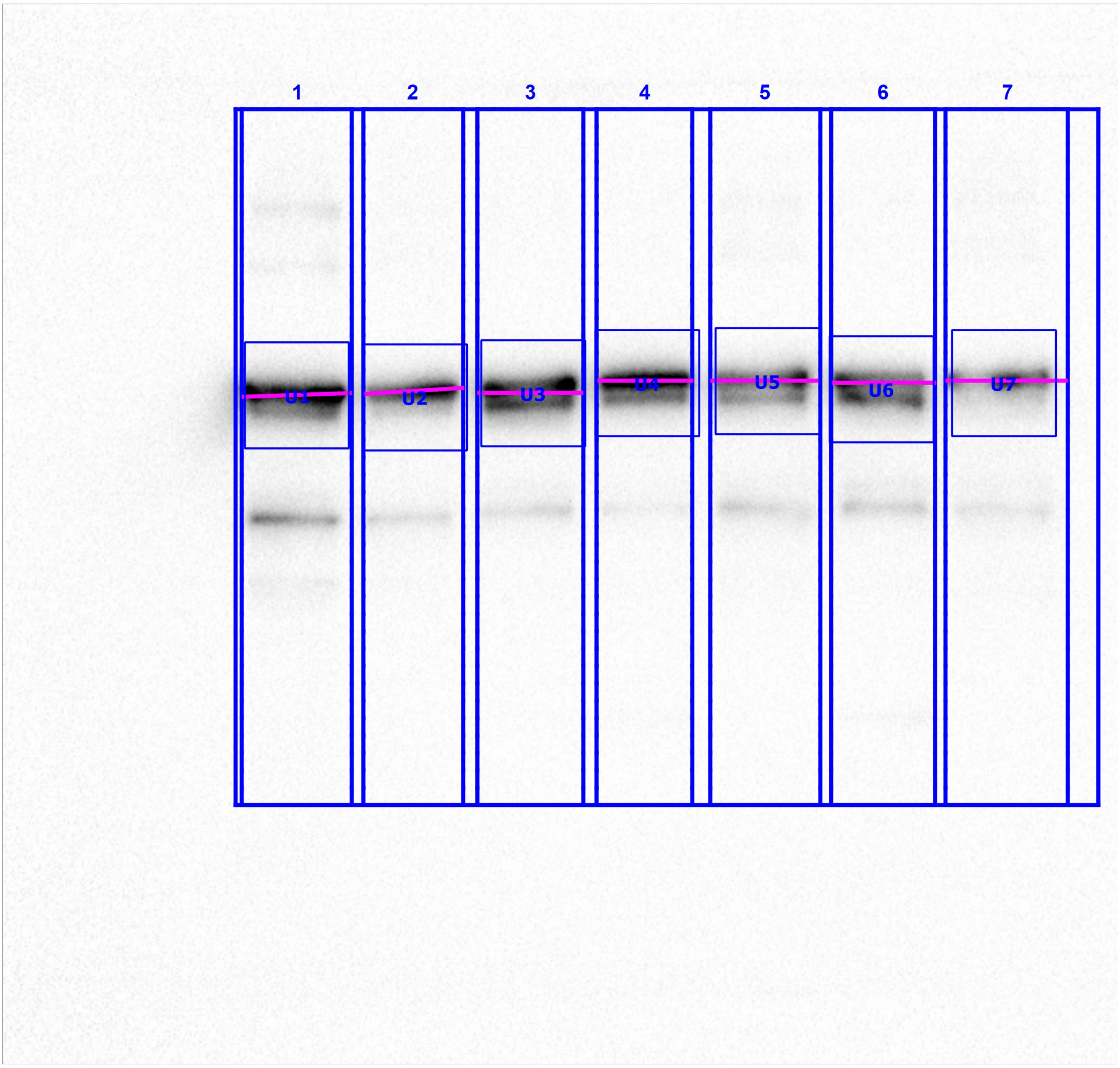

C:\Users\rusak\OneDrive\Dokumenty\Badania\CHI3L2 in BC\BC westerny ilościowo\analizaCHI3L2\analiza\żel 3\3 tub\3 żel 1 powt tub UMED 2018-03-07 11hr 10min_Exposure_16.8sec.scn

Acquisition Information

Imager	ChemiDoc MP
Exposure Time (sec)	16.800 (Signal Accumulation)

Flat Field	Applied (Lens)
Serial Number	731BR01769
Software Version	5.2.1
Application	Chemi Hi Resolution
Excitation Source	No Illumination
Emission Filter	No Filter
Binning	2x2

Image Information

Acquisition Date	7/3/2018 11:11:08 AM
User Name	UMED
Image Area (mm)	X: 97.0 Y: 72.5
Pixel Size (µm)	X: 139.4 Y: 139.4
Data Range (Int)	0 - 18152

Analysis Settings

Detection	Lane detection:<br>Manually created lanes<br><br>Band detection:<br>Automatically detected bands with sensitivity: Low<br>Manually adjusted bands<br><br>Lane Background Subtraction:<br>Lane background subtracted with disk size: 10<br><br>Lane width: Variable
Volume Analysis	Background subtraction method: Local<br>Quantity regression method: Linear

Lane Statistics

Lane No.	Adj. Total Band Vol. (Int)	Total Band Vol. (Int)	Adj. Total Lane Vol. (Int)	Total Lane Vol. (Int)	Bkgd. Vol. (Int)	Norm. Factor
1	10 684 008	11 255 004	14 615 640	18 292 230	3 676 590	N/A
2	6 087 074	6 361 327	7 559 769	9 931 467	2 371 698	N/A
3	9 606 636	9 985 404	11 133 772	13 368 680	2 234 908	N/A
4	9 249 694	9 514 351	10 509 529	12 311 462	1 801 933	N/A
5	6 728 076	7 095 222	8 748 378	11 369 970	2 621 592	N/A
6	3 813 729	4 006 050	9 349 320	11 958 684	2 609 364	N/A
7	4 832 580	5 148 360	6 683 640	9 743 820	3 060 180	N/A

Lane And Band Analysis

Lane 1

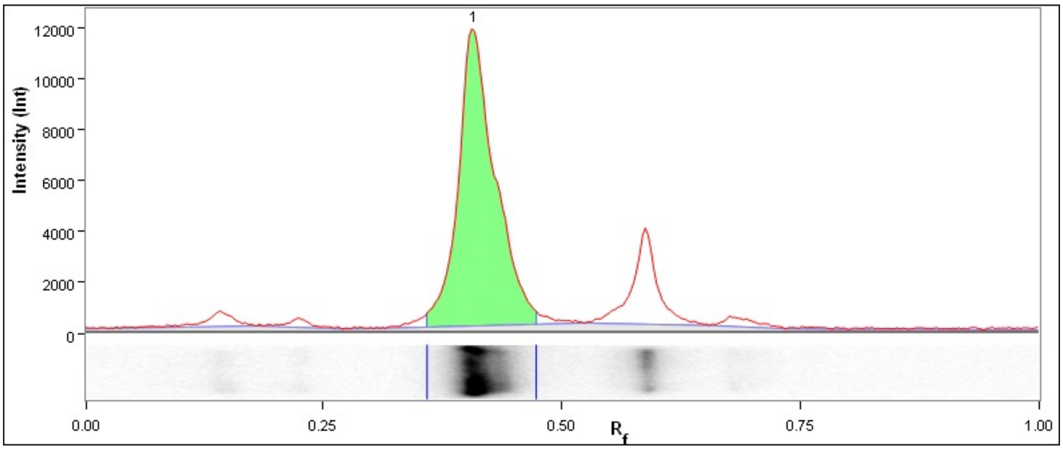

Band No.	Band Label	Mol. Wt. (KDa)	Relative Front	Adj. Volume (Int)	Volume (Int)	Abs. Quant.	Rel. Quant.	Band %	Lane %
1		N/A	0,411	10 684 008	11 255 004	N/A	N/A	100,0	73,1

Band Detection	Automatically detected bands with sensitivity: Low
Lane Background	Lane background subtracted with disk size: 10
Lane Width	7.53 mm

Lane 2

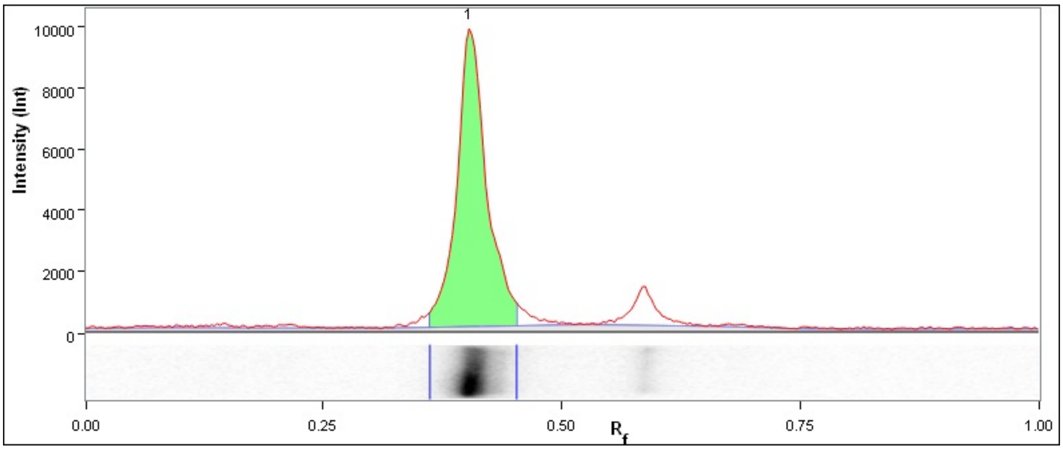

Band No.	Band Label	Mol. Wt. (KDa)	Relative Front	Adj. Volume (Int)	Volume (Int)	Abs. Quant.	Rel. Quant.	Band %	Lane %
1		N/A	0,405	6 087 074	6 361 327	N/A	N/A	100,0	80,5

Band Detection	Automatically detected bands with sensitivity: Low
Lane Background	Lane background subtracted with disk size: 10
Lane Width	6.83 mm

Lane 3

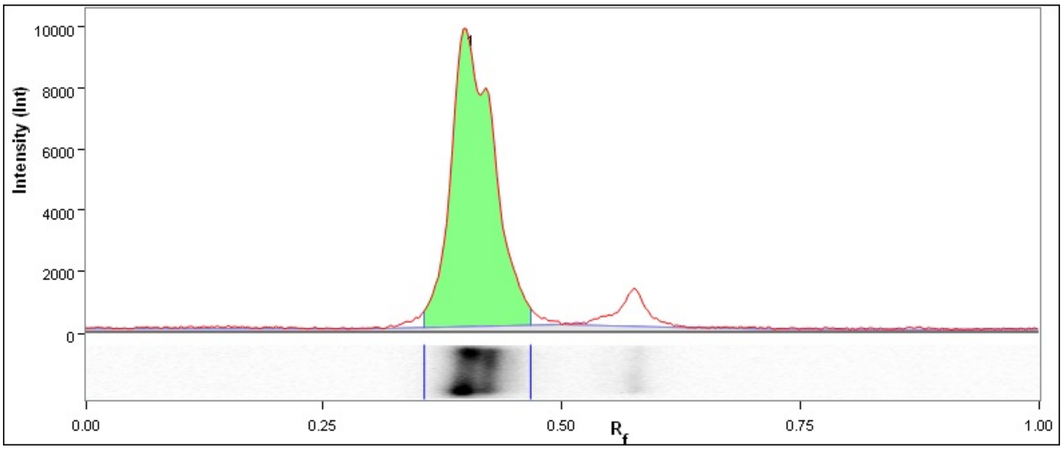

Band No.	Band Label	Mol. Wt. (KDa)	Relative Front	Adj. Volume (Int)	Volume (Int)	Abs. Quant.	Rel. Quant.	Band %	Lane %
1		N/A	0,408	9 606 636	9 985 404	N/A	N/A	100,0	86,3

Band Detection	Automatically detected bands with sensitivity: Low
Lane Background	Lane background subtracted with disk size: 10
Lane Width	7.25 mm

Lane 4

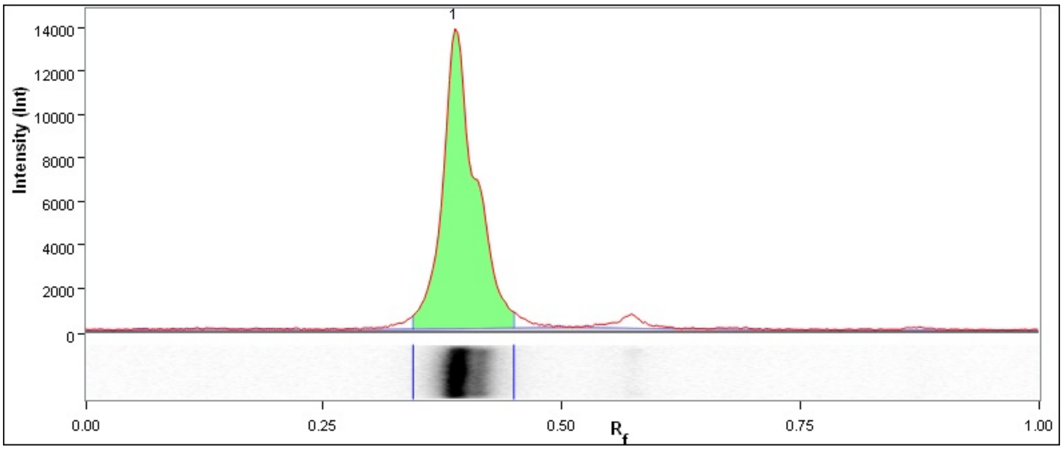

Band No.	Band Label	Mol. Wt. (KDa)	Relative Front	Adj. Volume (Int)	Volume (Int)	Abs. Quant.	Rel. Quant.	Band %	Lane %
1		N/A	0,390	9 249 694	9 514 351	N/A	N/A	100,0	88,0

Band Detection	Automatically detected bands with sensitivity: Low
Lane Background	Lane background subtracted with disk size: 10
Lane Width	6.55 mm

Lane 5

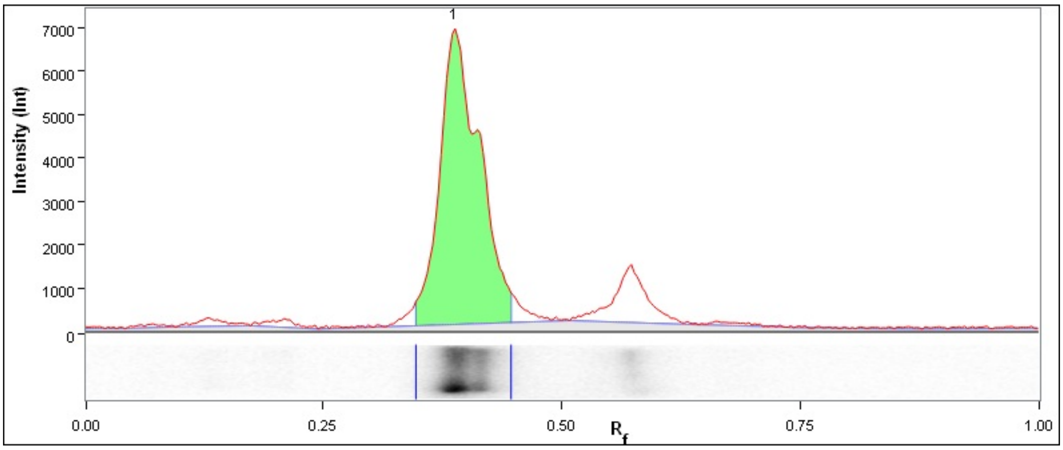

Band No.	Band Label	Mol. Wt. (KDa)	Relative Front	Adj. Volume (Int)	Volume (Int)	Abs. Quant.	Rel. Quant.	Band %	Lane %
1		N/A	0,390	6 728 076	7 095 222	N/A	N/A	100,0	76,9

Band Detection	Automatically detected bands with sensitivity: Low
Lane Background	Lane background subtracted with disk size: 10
Lane Width	7.53 mm

Lane 6

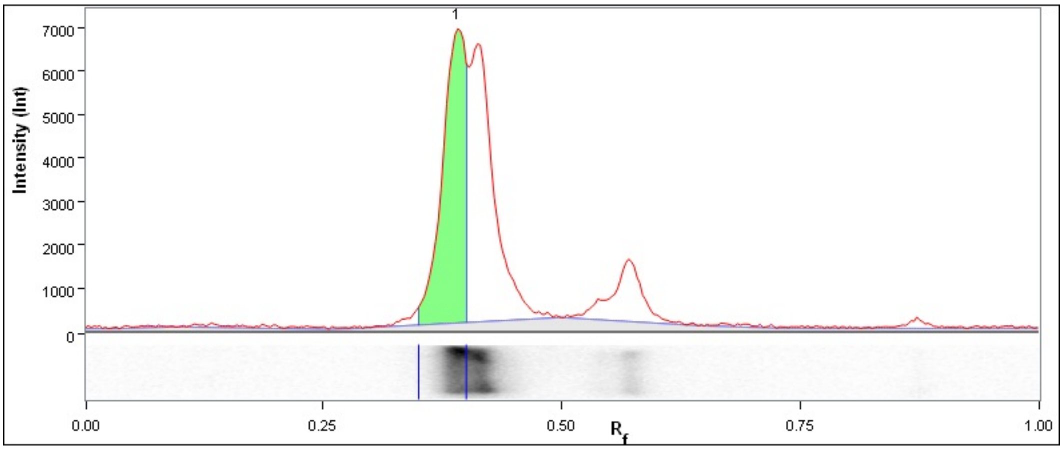

Band No.	Band Label	Mol. Wt. (KDa)	Relative Front	Adj. Volume (Int)	Volume (Int)	Abs. Quant.	Rel. Quant.	Band %	Lane %
1		N/A	0,393	3 813 729	4 006 050	N/A	N/A	100,0	40,8

Band Detection	Automatically detected bands with sensitivity: Low
Lane Background	Lane background subtracted with disk size: 10
Lane Width	7.11 mm

Lane 7

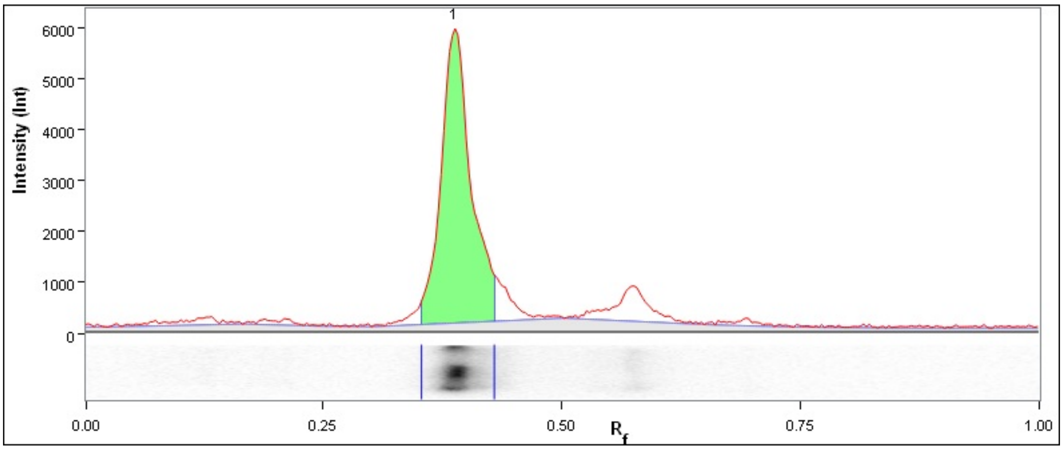

Band No.	Band Label	Mol. Wt. (KDa)	Relative Front	Adj. Volume (Int)	Volume (Int)	Abs. Quant.	Rel. Quant.	Band %	Lane %
1		N/A	0,390	4 832 580	5 148 360	N/A	N/A	100,0	72,3

Band Detection	Automatically detected bands with sensitivity: Low
Lane Background	Lane background subtracted with disk size: 10
Lane Width	8.36 mm

Volume Analysis

No.	Label	Type	Volume (Int)	Adj. Vol. (Int)	Mean Bkgd. (Int)	Abs. Quant.	Rel. Quant.	# of Pixels	Min. Value (Int)	Max. Value (Int)	Mean Value (Int)	Std. Dev.	Area (mm2)
1	U1	Unknown	11 250 224	7 539 192	1 399,3	N/A	N/A	2 652	0	17 356	4 242,2	4 281,5	51,5
2	U2	Unknown	6 929 024	5 113 591	684,6	N/A	N/A	2 652	0	14 612	2 612,8	3 160,0	51,5
3	U3	Unknown	10 175 104	7 604 381	969,4	N/A	N/A	2 652	0	16 916	3 836,8	3 837,1	51,5
4	U4	Unknown	10 143 140	8 155 251	749,6	N/A	N/A	2 652	0	18 152	3 824,7	4 341,3	51,5
5	U5	Unknown	7 294 980	5 099 983	827,7	N/A	N/A	2 652	0	13 236	2 750,7	2 713,9	51,5
6	U6	Unknown	8 370 384	6 498 830	705,7	N/A	N/A	2 652	0	13 872	3 156,3	3 080,3	51,5
7	U7	Unknown	5 510 468	3 434 432	782,8	N/A	N/A	2 652	0	12 796	2 077,9	2 362,3	51,5

Image Report: 3 żel 1 powt YKL39 UMED 2018-03-06 11hr 04min_Exposure_60.0sec

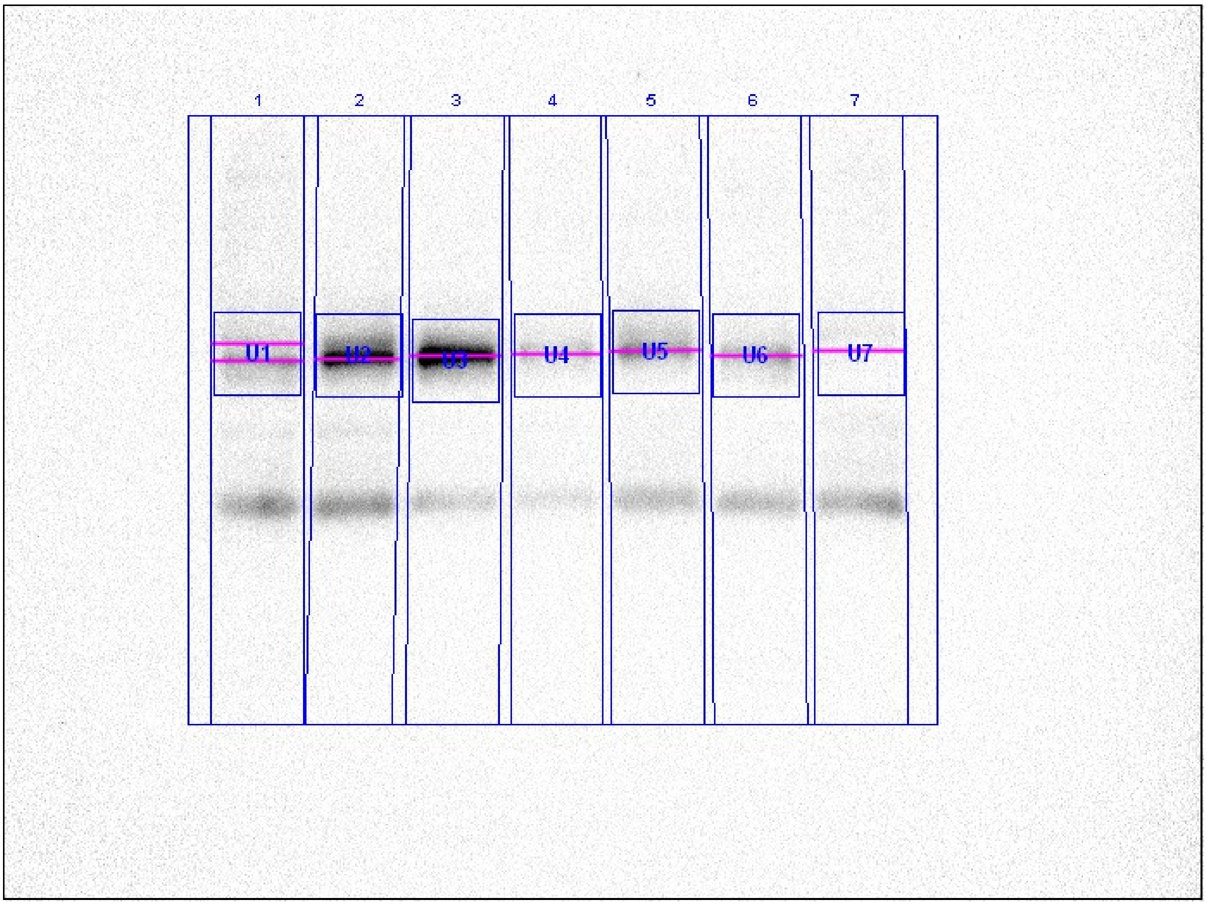

C:\Documents and Settings\Jaga\Pulpit\YKL-39 WB\analiza\żel 3\3 YKL39\3 żel 1 powt YKL39 UMED 2018-03-06 11hr 04min_Exposure_60.0sec.scn

Acquisition Information

Imager	ChemiDoc™ MP
Exposure Time (sec)	60.000 (Signal Accumulation)
Flat Field	Applied (Lens)
Serial Number	731BR01769
Software Version	5.2.1
Application	Chemi Hi Resolution
Excitation Source	No Illumination
Emission Filter	No Filter
Binning	2x2

Image Information

Acquisition Date	2018-03-06 11:05:21
User Name	UMED
Image Area (mm)	X: 97.0 Y: 72.5
Pixel Size (um)	X: 139.4 Y: 139.4
Data Range (Int)	0 - 17288

Analysis Settings

Detection	Lane detection:<br>Automatically detected lanes with manual adjustments<br><br>Band detection:<br>Automatically detected bands with sensitivity: Low<br>Manually adjusted bands<br><br>Lane Background Subtraction:<br>Lane background subtracted with disk size: 10<br><br>Lane width: Variable
Volume Analysis	Background subtraction method: Local<br>Quantity regression method: Linear

Lane And Band Analysis

Lane 1

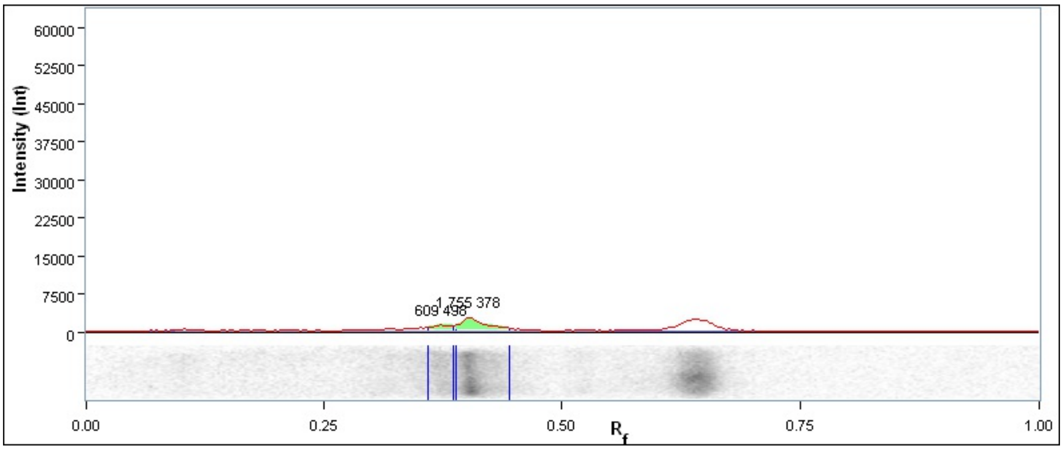

Band No.	Band Label	Mol. Wt. (KDa)	Relative Front	Volume (Int)	Abs. Quant.	Rel. Quant.	Band %	Lane %
1		N/A	0,376	609 498	N/A	N/A	25,8	9,0
2		N/A	0,404	1 755 378	N/A	N/A	74,2	25,8

Band Detection	Automatically detected bands with sensitivity: Low
Lane Background	Lane background subtracted with disk size: 10
Lane Width	7.53 mm

Lane 2

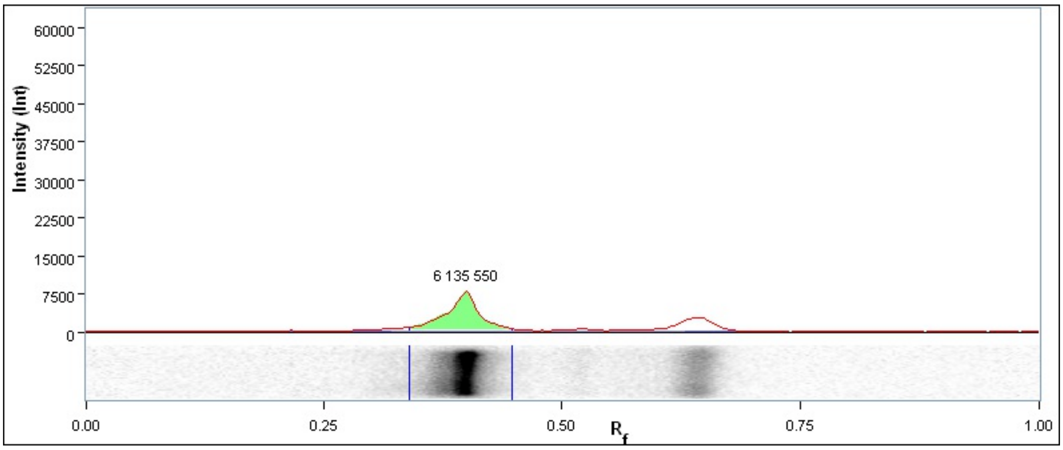

Band No.	Band Label	Mol. Wt. (KDa)	Relative Front	Volume (Int)	Abs. Quant.	Rel. Quant.	Band %	Lane %
1		N/A	0,401	6 135 550	N/A	N/A	100,0	61,1

Band Detection	Automatically detected bands with sensitivity: Low
Lane Background	Lane background subtracted with disk size: 10
Lane Width	6.97 mm

Lane 3

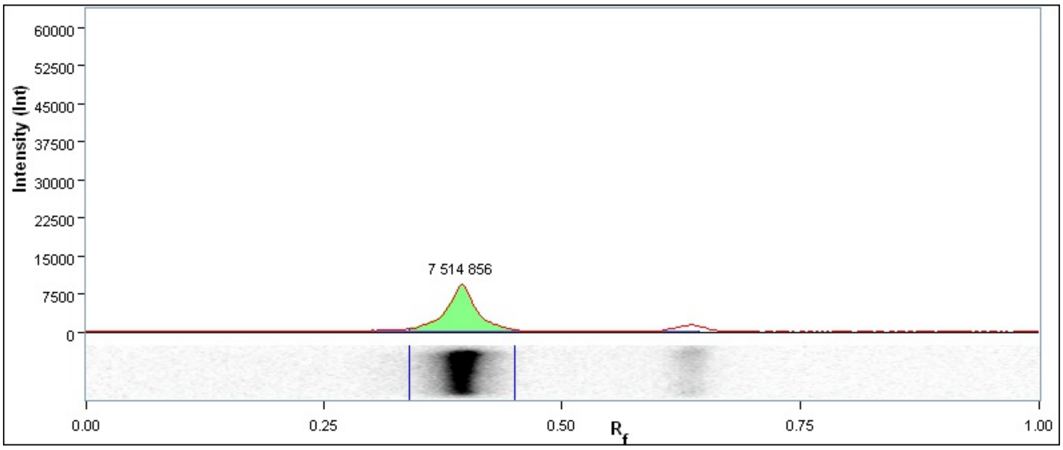

Band No.	Band Label	Mol. Wt. (KDa)	Relative Front	Volume (Int)	Abs. Quant.	Rel. Quant.	Band %	Lane %
1		N/A	0,395	7 514 856	N/A	N/A	100,0	77,0

Band Detection	Automatically detected bands with sensitivity: Low
Lane Background	Lane background subtracted with disk size: 10
Lane Width	7.53 mm

Lane 4

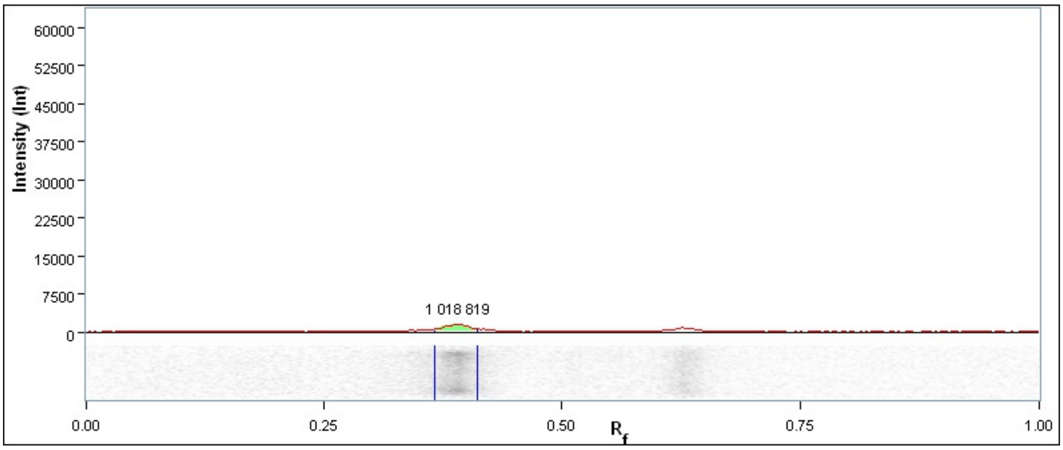

Band No.	Band Label	Mol. Wt. (KDa)	Relative Front	Volume (Int)	Abs. Quant.	Rel. Quant.	Band %	Lane %
1		N/A	0,393	1 018 819	N/A	N/A	100,0	32,2

Band Detection	Automatically detected bands with sensitivity: Low
Lane Background	Lane background subtracted with disk size: 10
Lane Width	7.39 mm

Lane 5

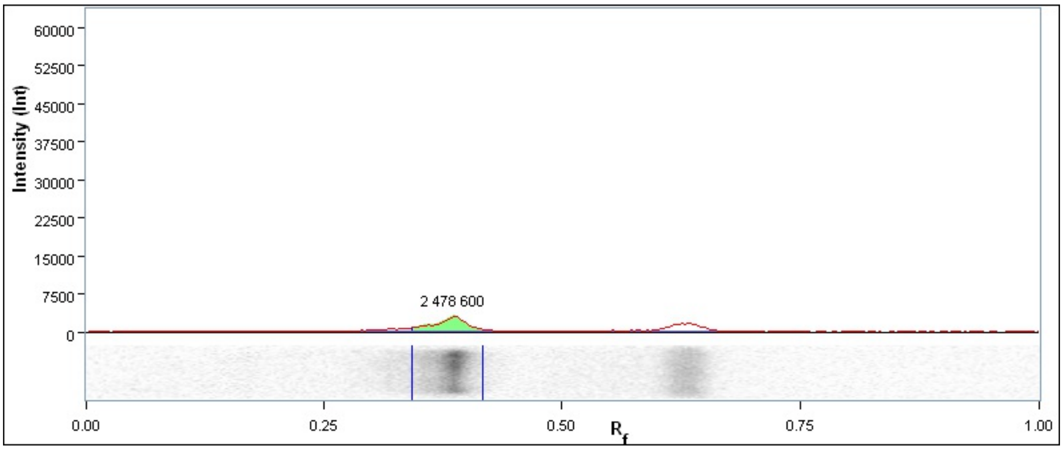

Band No.	Band Label	Mol. Wt. (KDa)	Relative Front	Volume (Int)	Abs. Quant.	Rel. Quant.	Band %	Lane %
1		N/A	0,387	2 478 600	N/A	N/A	100,0	44,7

Band Detection	Automatically detected bands with sensitivity: Low
Lane Background	Lane background subtracted with disk size: 10
Lane Width	7.53 mm

Lane 6

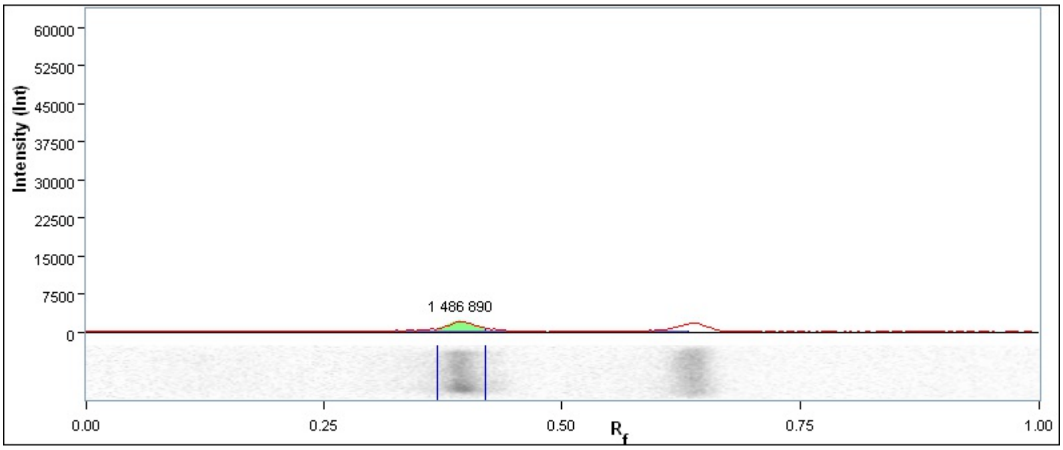

Band No.	Band Label	Mol. Wt. (KDa)	Relative Front	Volume (Int)	Abs. Quant.	Rel. Quant.	Band %	Lane %
1		N/A	0,395	1 486 890	N/A	N/A	100,0	33,4

Band Detection	Automatically detected bands with sensitivity: Low
Lane Background	Lane background subtracted with disk size: 10
Lane Width	7.53 mm

Lane 7

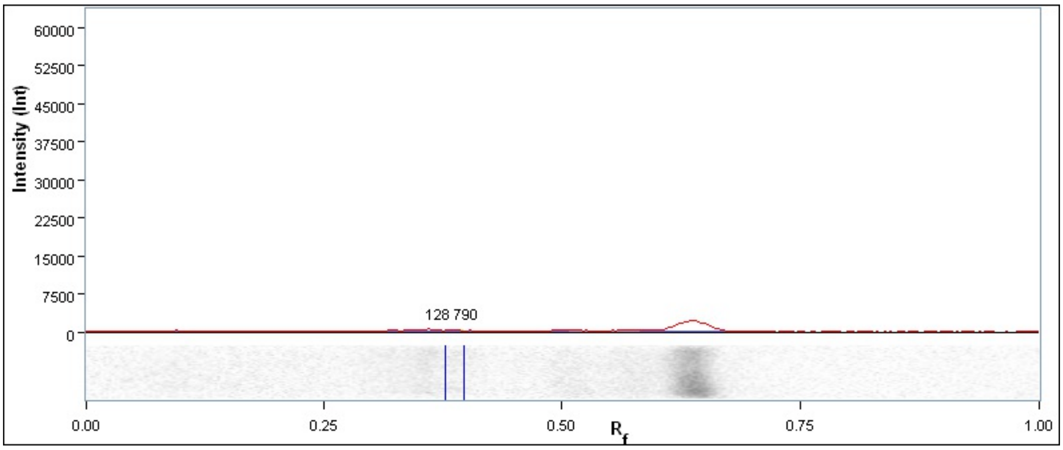

Band No.	Band Label	Mol. Wt. (KDa)	Relative Front	Volume (Int)	Abs. Quant.	Rel. Quant.	Band %	Lane %
1		N/A	0,387	128 790	N/A	N/A	100,0	3,6

Band Detection	Automatically detected bands with sensitivity: Low
Lane Background	Lane background subtracted with disk size: 10
Lane Width	7.53 mm

Volume Analysis

No.	Label	Type	Volume (Int)	Adj. Vol. (Int)	Mean Bkgd. (Int)	Abs. Quant.	Rel. Quant.	# of Pixels	Min. Value (Int)	Max. Value (Int)	Mean Value (Int)	Std. Dev.	Area (mm2)
1	U1	Unknown	3 544 984	2 038 312	627,8	N/A	N/A	2 400	0	5 660	1 477,1	922,3	46,6
2	U2	Unknown	7 145 564	5 390 684	731,2	N/A	N/A	2 400	0	13 672	2 977,3	2 690,8	46,6
3	U3	Unknown	7 988 104	6 376 120	671,7	N/A	N/A	2 400	0	17 288	3 328,4	3 506,8	46,6
4	U4	Unknown	1 778 856	1 062 360	298,5	N/A	N/A	2 400	0	3 620	741,2	645,9	46,6
5	U5	Unknown	3 296 888	2 194 040	459,5	N/A	N/A	2 400	0	5 872	1 373,7	1 160,5	46,6
6	U6	Unknown	2 382 332	1 655 324	302,9	N/A	N/A	2 400	0	4 620	992,6	859,6	46,6
7	U7	Unknown	1 016 152	312 952	293,0	N/A	N/A	2 400	0	2 316	423,4	370,0	46,6

Image Report: 3 żel 3 powt tub UMED 2018-03-01 10hr 55min_Exposure_16.8sec

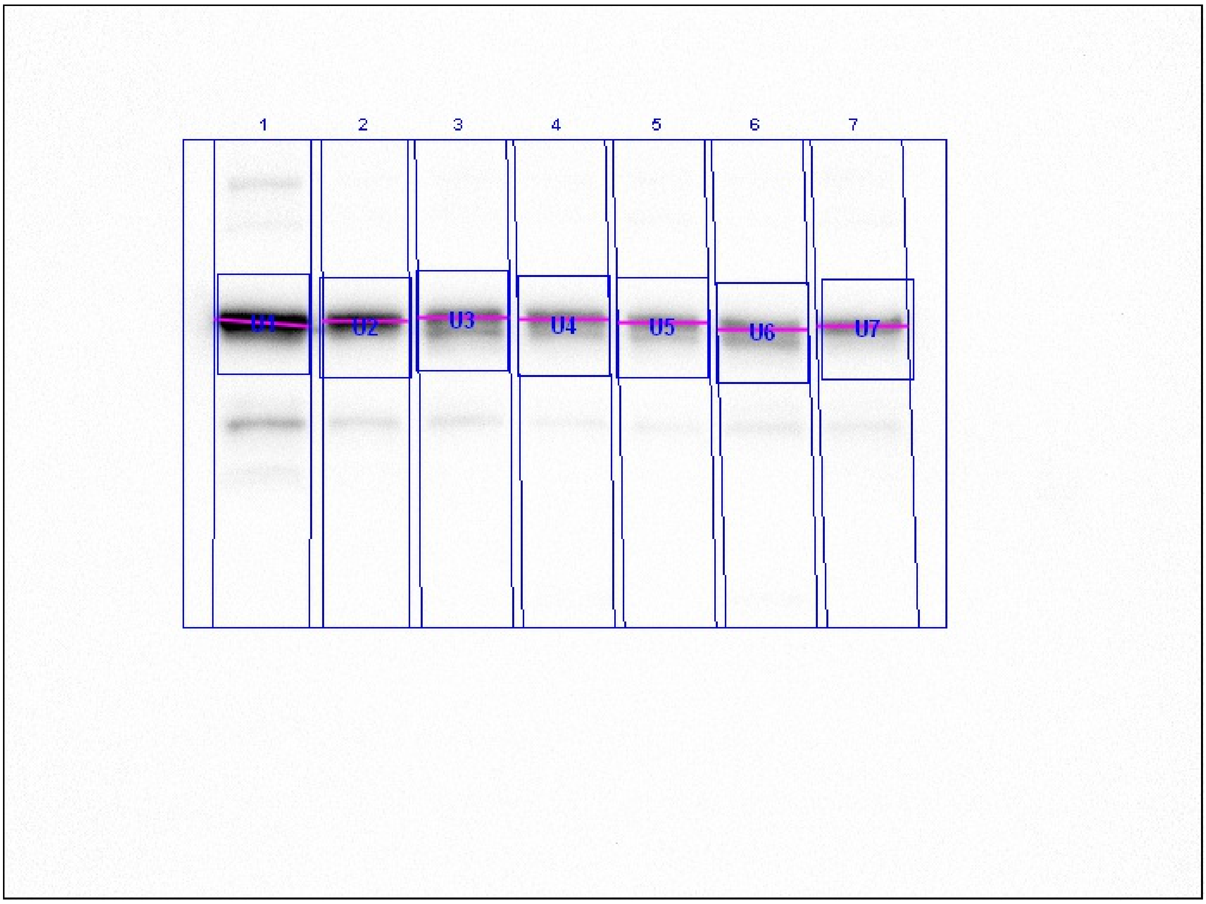

C:\Documents and Settings\Jaga\Pulpit\YKL-39 WB\analiza\żel 3\3 tub\3 żel 3 powt tub UMED 2018-03-01 10hr 55min_Exposure_16.8sec.scn

Acquisition Information

Imager	ChemiDoc™ MP
Exposure Time (sec)	16.800 (Signal Accumulation)
Flat Field	Applied (Lens)
Serial Number	731BR01769
Software Version	5.2.1
Application	Chemi Hi Resolution
Excitation Source	No Illumination
Emission Filter	No Filter
Binning	2x2

Image Information

Acquisition Date	2018-03-01 10:55:55
User Name	UMED
Image Area (mm)	X: 97.0 Y: 72.5
Pixel Size (um)	X: 139.4 Y: 139.4
Data Range (Int)	0 - 33812

Analysis Settings

Detection	Lane detection:<br>Automatically detected lanes with manual adjustments<br><br>Band detection:<br>Automatically detected bands with sensitivity: Low<br>Manually adjusted bands<br><br>Lane Background Subtraction:<br>Lane background subtracted with disk size: 10<br><br>Lane width: Variable
Volume Analysis	Background subtraction method: Local<br>Quantity regression method: Linear

Lane And Band Analysis

Lane 1

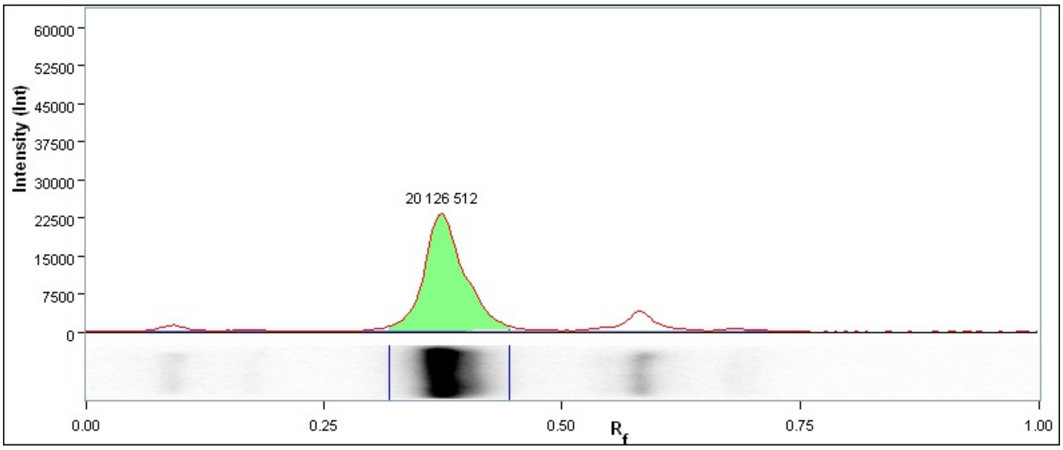

Band No.	Band Label	Mol. Wt. (KDa)	Relative Front	Volume (Int)	Abs. Quant.	Rel. Quant.	Band %	Lane %
1		N/A	0,377	20 126 512	N/A	N/A	100,0	80,3

Band Detection	Automatically detected bands with sensitivity: Low
Lane Background	Lane background subtracted with disk size: 10
Lane Width	7.80 mm

Lane 2

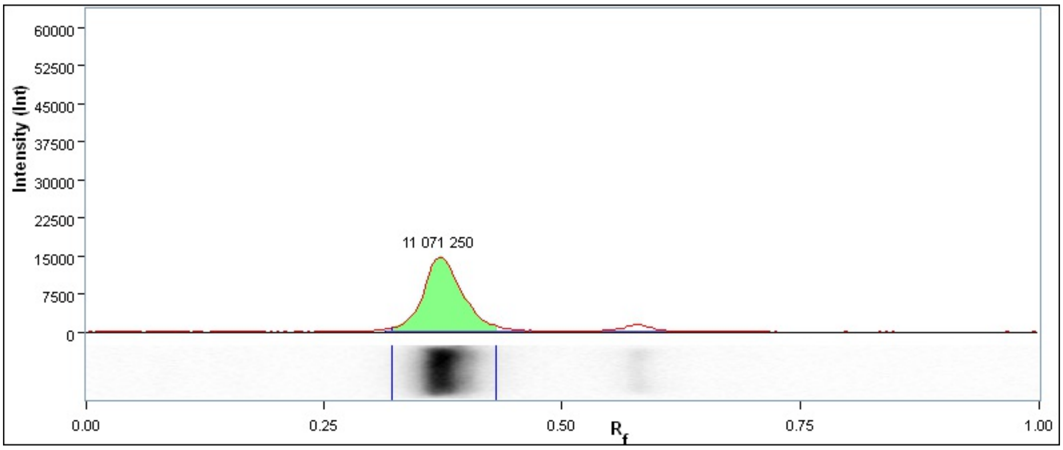

Band No.	Band Label	Mol. Wt. (KDa)	Relative Front	Volume (Int)	Abs. Quant.	Rel. Quant.	Band %	Lane %
1		N/A	0,373	11 071 250	N/A	N/A	100,0	85,8

Band Detection	Automatically detected bands with sensitivity: Low

Lane Background	Lane background subtracted with disk size: 10
Lane Width	6.97 mm

Lane 3

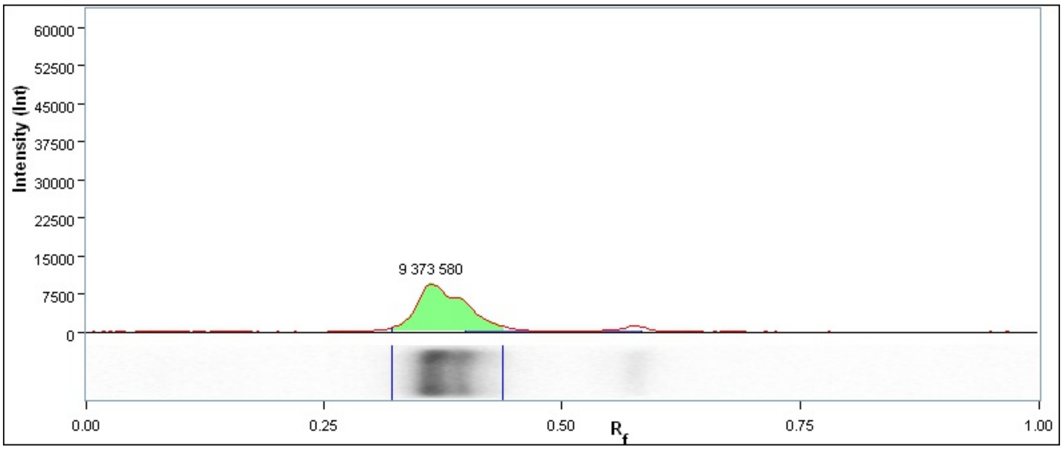

Band No.	Band Label	Mol. Wt. (KDa)	Relative Front	Volume (Int)	Abs. Quant.	Rel. Quant.	Band %	Lane %
1		N/A	0,366	9 373 580	N/A	N/A	100,0	84,7

Band Detection	Automatically detected bands with sensitivity: Low
Lane Background	Lane background subtracted with disk size: 10
Lane Width	7.39 mm

Lane 4

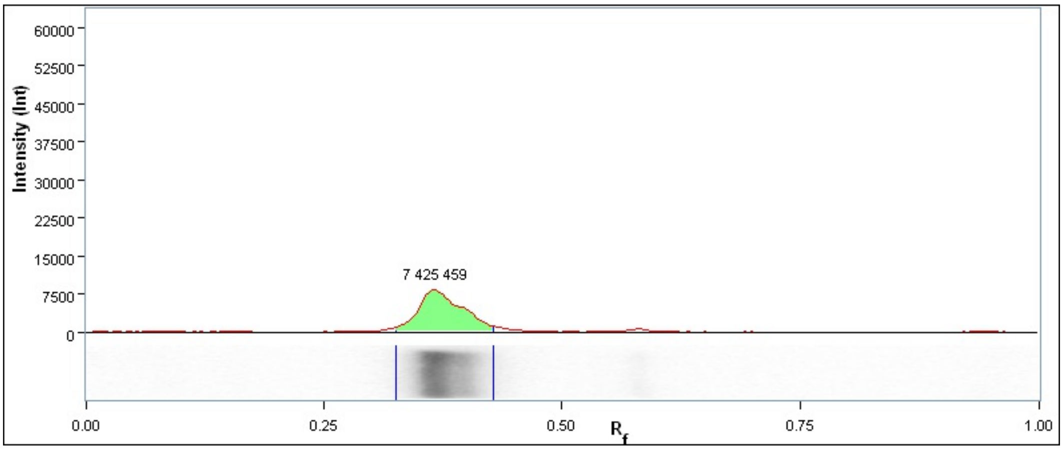

Band No.	Band Label	Mol. Wt. (KDa)	Relative Front	Volume (Int)	Abs. Quant.	Rel. Quant.	Band %	Lane %
1		N/A	0,370	7 425 459	N/A	N/A	100,0	85,3

Band Detection	Automatically detected bands with sensitivity: Low
Lane Background	Lane background subtracted with disk size: 10
Lane Width	7.39 mm

Lane 5

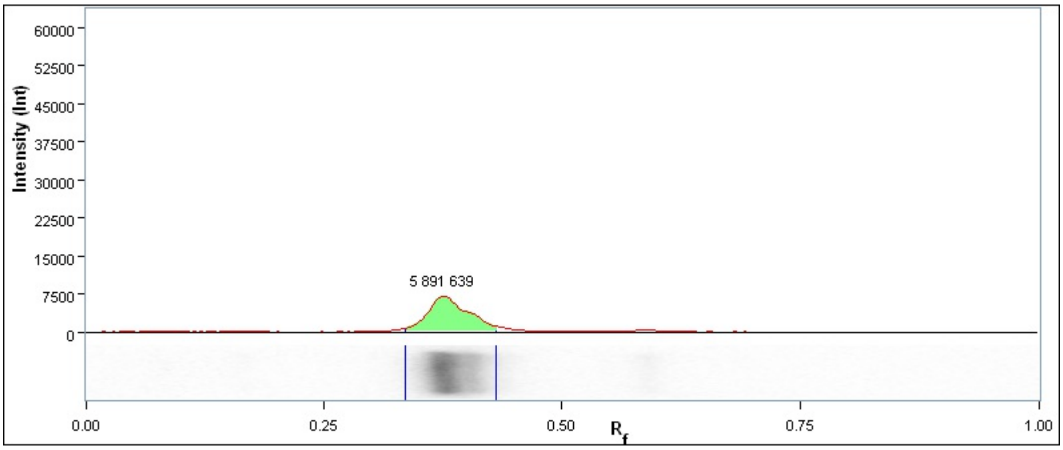

Band No.	Band Label	Mol. Wt. (KDa)	Relative Front	Volume (Int)	Abs. Quant.	Rel. Quant.	Band %	Lane %
1		N/A	0,377	5 891 639	N/A	N/A	100,0	83,1

Band Detection	Automatically detected bands with sensitivity: Low
Lane Background	Lane background subtracted with disk size: 10
Lane Width	7.39 mm

Lane 6

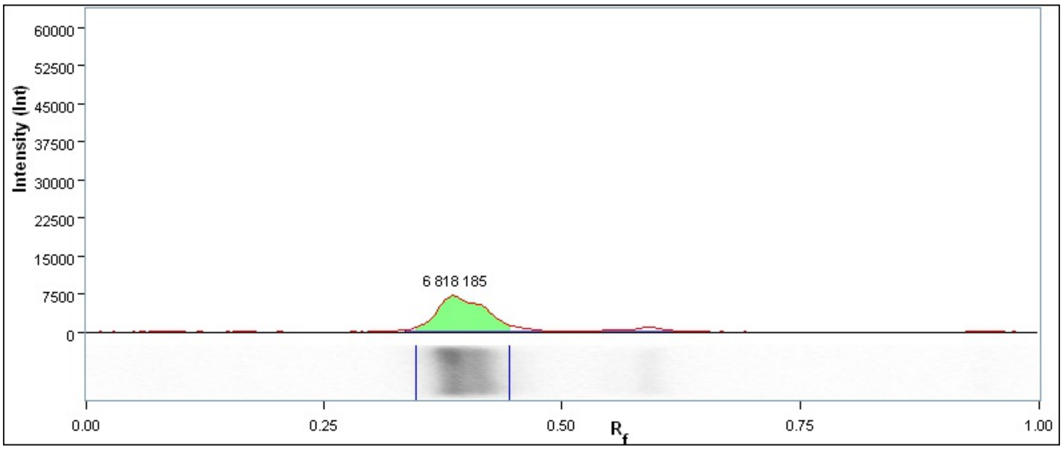

Band No.	Band Label	Mol. Wt. (KDa)	Relative Front	Volume (Int)	Abs. Quant.	Rel. Quant.	Band %	Lane %
1		N/A	0,391	6 818 185	N/A	N/A	100,0	81,8

Band Detection	Automatically detected bands with sensitivity: Low
Lane Background	Lane background subtracted with disk size: 10
Lane Width	7.39 mm

Lane 7

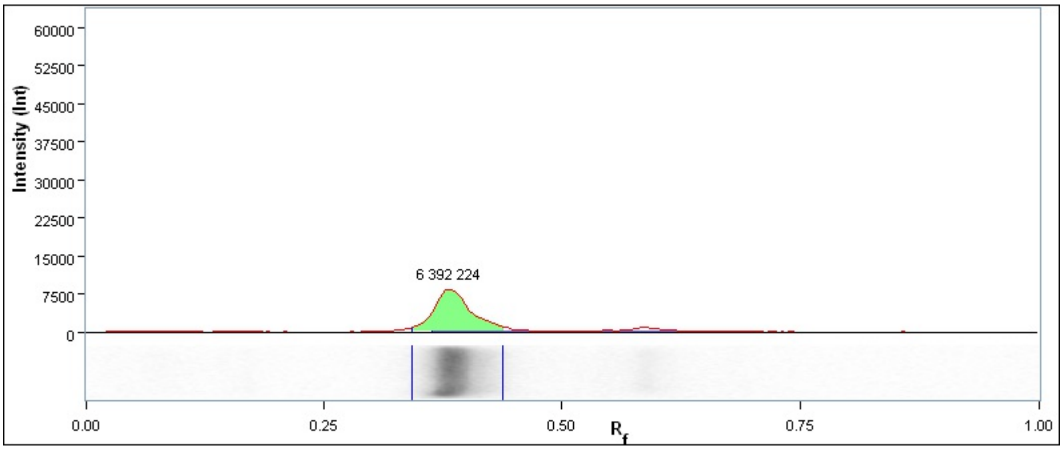

Band No.	Band Label	Mol. Wt. (KDa)	Relative Front	Volume (Int)	Abs. Quant.	Rel. Quant.	Band %	Lane %
1		N/A	0,384	6 392 224	N/A	N/A	100,0	80,3

Band Detection	Automatically detected bands with sensitivity: Low
Lane Background	Lane background subtracted with disk size: 10
Lane Width	7.39 mm

Volume Analysis

No.	Label	Type	Volume (Int)	Adj. Vol. (Int)	Mean Bkgd. (Int)	Abs. Quant.	Rel. Quant.	# of Pixels	Min. Value (Int)	Max. Value (Int)	Mean Value (Int)	Std. Dev.	Area (mm2)
1	U1	Unknown	20 994 736	15 755 334	1 704,4	N/A	N/A	3 074	0	33 812	6 829,8	8 382,9	59,7
2	U2	Unknown	12 322 092	9 784 056	825,6	N/A	N/A	3 074	0	21 256	4 008,5	5 297,9	59,7
3	U3	Unknown	10 216 060	8 361 867	603,2	N/A	N/A	3 074	0	14 388	3 323,4	3 633,9	59,7
4	U4	Unknown	8 344 444	6 676 269	542,7	N/A	N/A	3 074	0	12 096	2 714,5	3 089,9	59,7
5	U5	Unknown	6 744 448	5 373 553	446,0	N/A	N/A	3 074	0	11 408	2 194,0	2 706,7	59,7
6	U6	Unknown	7 912 080	6 326 005	516,0	N/A	N/A	3 074	0	10 676	2 573,9	2 791,1	59,7
7	U7	Unknown	7 207 224	5 256 023	634,7	N/A	N/A	3 074	0	11 468	2 344,6	2 928,1	59,7

Image Report: 3 żel 3 powt YKL39 UMED 2018-02-28 11hr 09min_Exposure_60.0sec

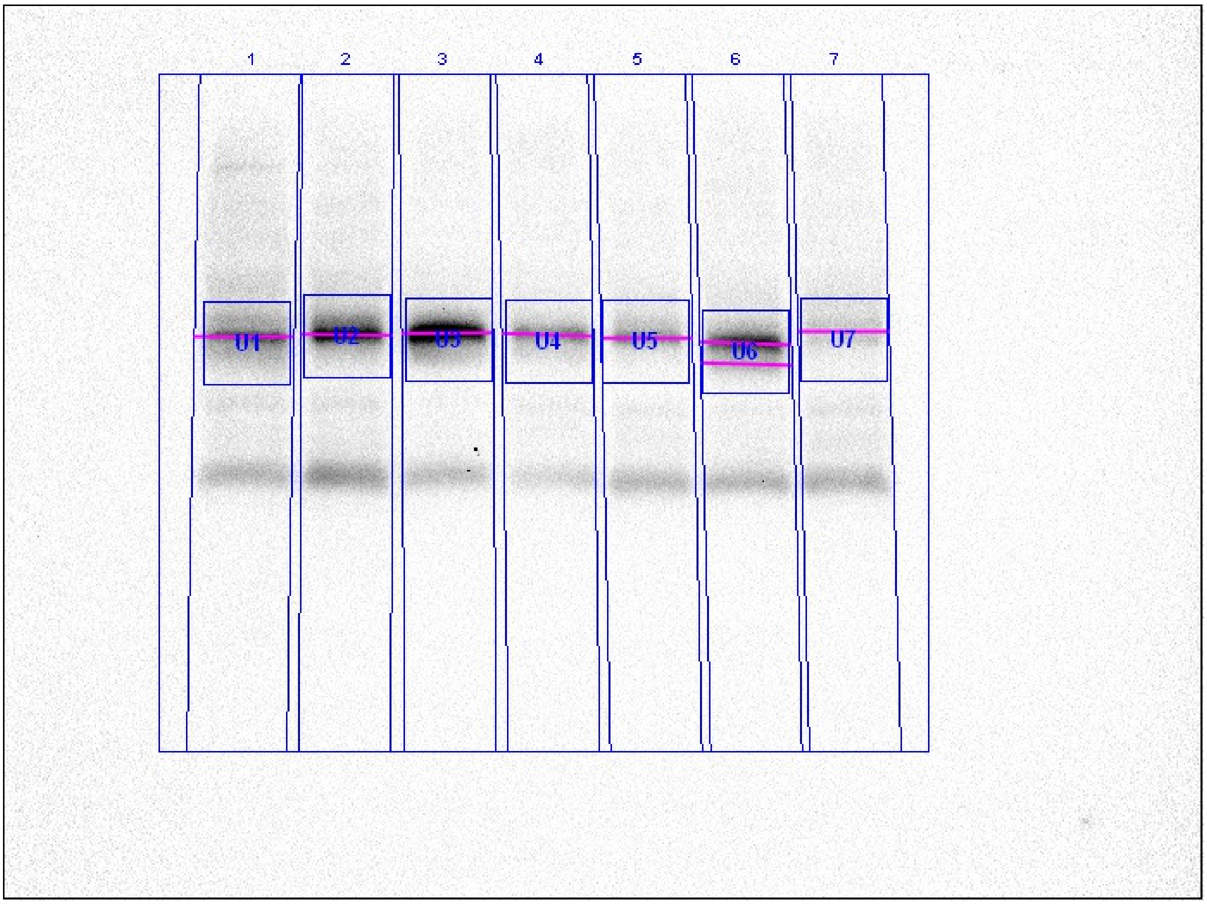

C:\Documents and Settings\Jaga\Pulpit\YKL-39 WB\analiza\żel 3\3 YKL39\3 żel 3 powt YKL39 UMED 2018-02-28 11hr 09min_Exposure_60.0sec.scn

Acquisition Information

Imager	ChemiDoc™ MP
Exposure Time (sec)	60.000 (Signal Accumulation)
Flat Field	Applied (Lens)
Serial Number	731BR01769
Software Version	5.2.1
Application	Chemi Hi Resolution
Excitation Source	No Illumination
Emission Filter	No Filter
Binning	2x2

Image Information

Acquisition Date	2018-02-28 11:10:40
User Name	UMED
Image Area (mm)	X: 97.0 Y: 72.5
Pixel Size (um)	X: 139.4 Y: 139.4
Data Range (Int)	0 - 43928

Analysis Settings

Detection	Lane detection:<br>Automatically detected lanes with manual adjustments<br><br>Band detection:<br>Automatically detected bands with sensitivity: Low<br>Manually adjusted bands<br><br>Lane Background Subtraction:<br>Lane background subtracted with disk size: 10<br><br>Lane width: Variable
Volume Analysis	Background subtraction method: Local<br>Quantity regression method: Linear

Lane And Band Analysis

Lane 1

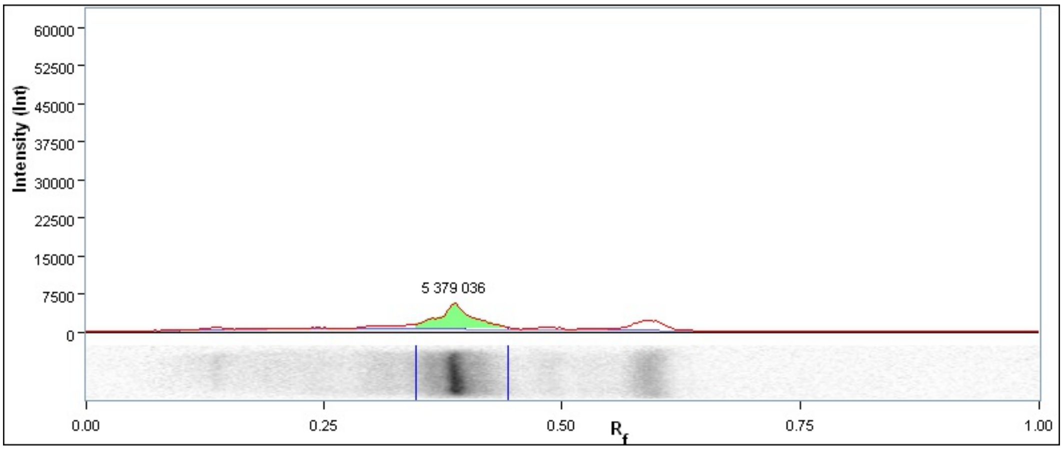

Band No.	Band Label	Mol. Wt. (KDa)	Relative Front	Volume (Int)	Abs. Quant.	Rel. Quant.	Band %	Lane %
1		N/A	0,388	5 379 036	N/A	N/A	100,0	53,0

Band Detection	Automatically detected bands with sensitivity: Low
Lane Background	Lane background subtracted with disk size: 10
Lane Width	8.08 mm

Lane 2

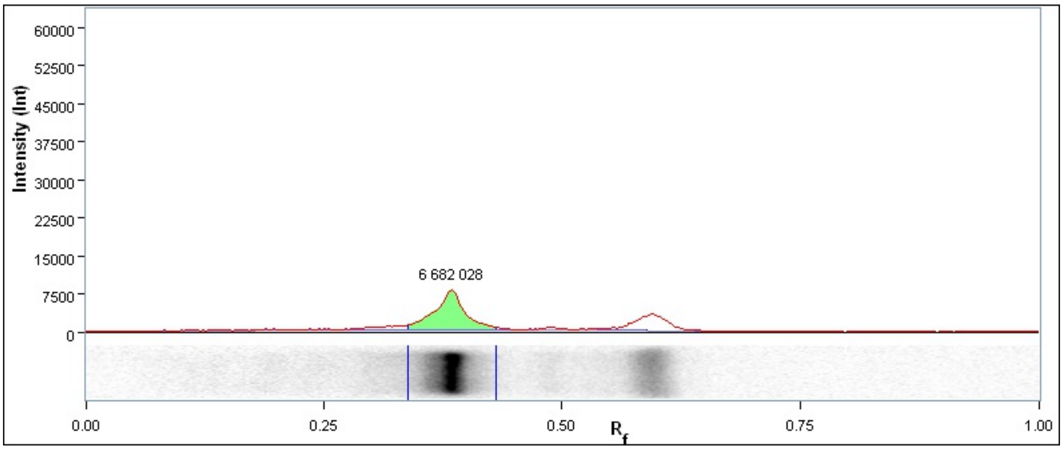

Band No.	Band Label	Mol. Wt. (KDa)	Relative Front	Volume (Int)	Abs. Quant.	Rel. Quant.	Band %	Lane %
1		N/A	0,386	6 682 028	N/A	N/A	100,0	54,4

Band Detection	Automatically detected bands with sensitivity: Low

Lane Background	Lane background subtracted with disk size: 10
Lane Width	7.39 mm

Lane 3

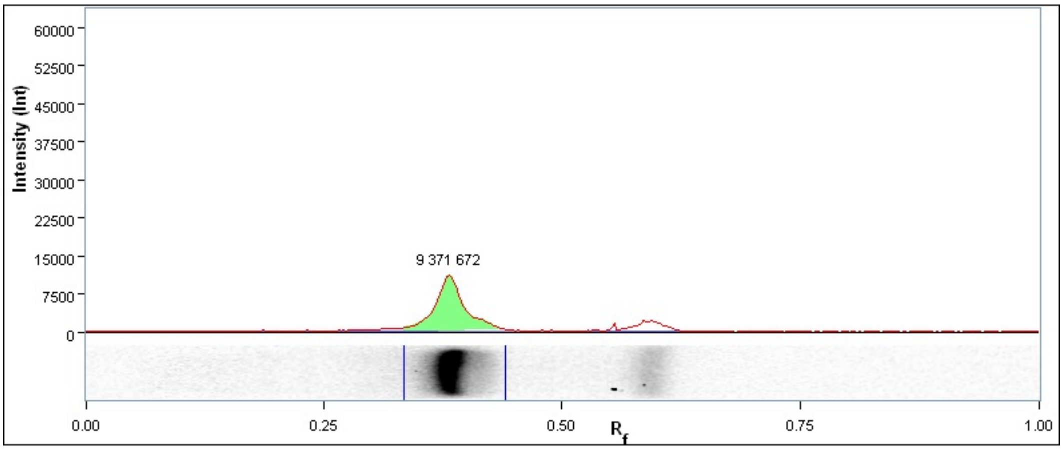

Band No.	Band Label	Mol. Wt. (KDa)	Relative Front	Volume (Int)	Abs. Quant.	Rel. Quant.	Band %	Lane %
1		N/A	0,383	9 371 672	N/A	N/A	100,0	72,3

Band Detection	Automatically detected bands with sensitivity: Low
Lane Background	Lane background subtracted with disk size: 10
Lane Width	7.39 mm

Lane 4

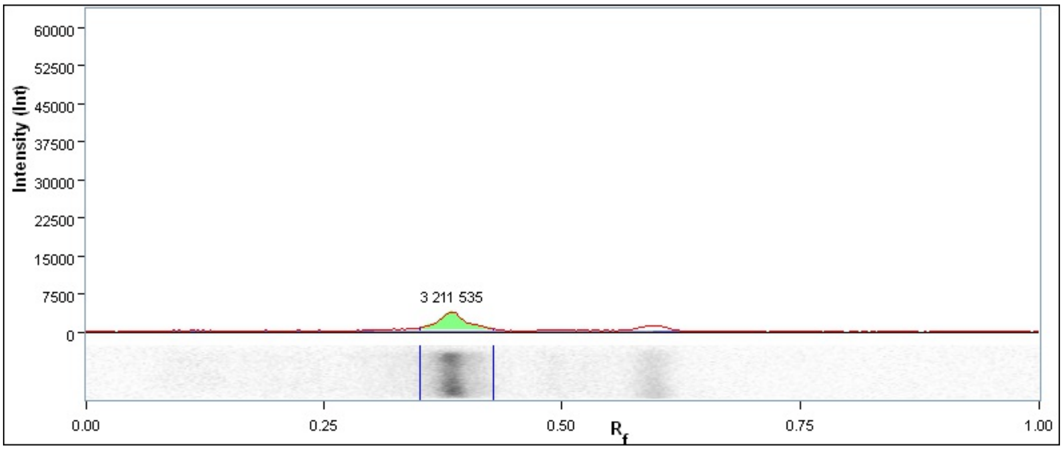

Band No.	Band Label	Mol. Wt. (KDa)	Relative Front	Volume (Int)	Abs. Quant.	Rel. Quant.	Band %	Lane %
1		N/A	0,386	3 211 535	N/A	N/A	100,0	53,8

Band Detection	Automatically detected bands with sensitivity: Low
Lane Background	Lane background subtracted with disk size: 10
Lane Width	7.39 mm

Lane 5

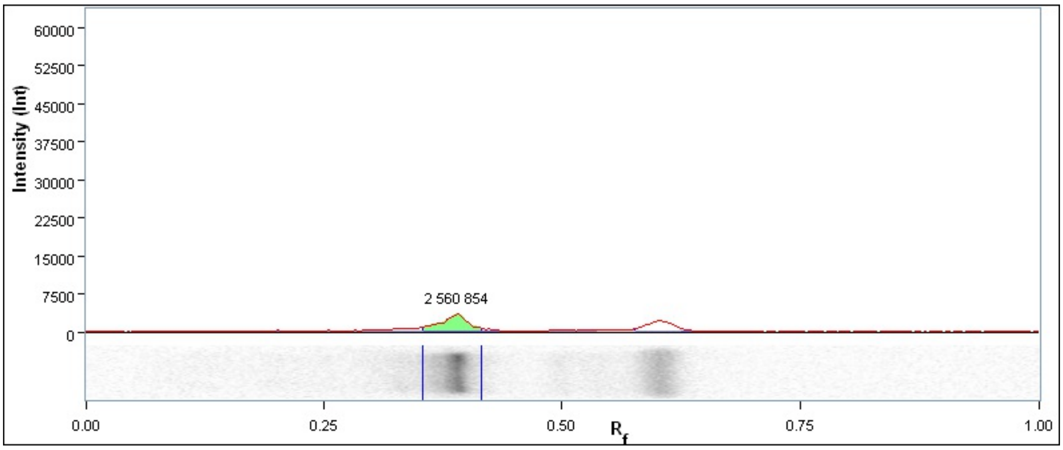

Band No.	Band Label	Mol. Wt. (KDa)	Relative Front	Volume (Int)	Abs. Quant.	Rel. Quant.	Band %	Lane %
1		N/A	0,391	2 560 854	N/A	N/A	100,0	41,1

Band Detection	Automatically detected bands with sensitivity: Low
Lane Background	Lane background subtracted with disk size: 10
Lane Width	7.39 mm

Lane 6

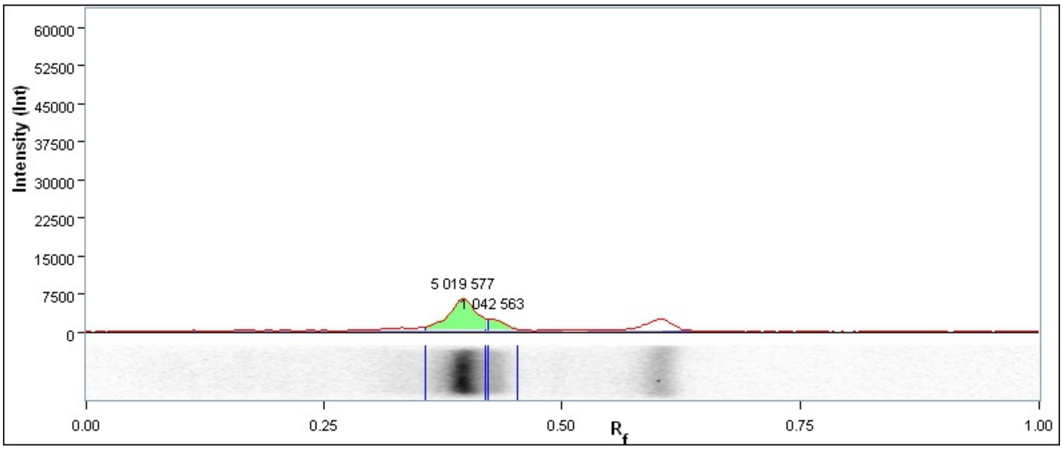

Band No.	Band Label	Mol. Wt. (KDa)	Relative Front	Volume (Int)	Abs. Quant.	Rel. Quant.	Band %	Lane %
1		N/A	0,398	5 019 577	N/A	N/A	82,8	51,0
2		N/A	0,429	1 042 563	N/A	N/A	17,2	10,6

Band Detection	Automatically detected bands with sensitivity: Low
Lane Background	Lane background subtracted with disk size: 10
Lane Width	7.39 mm

Lane 7

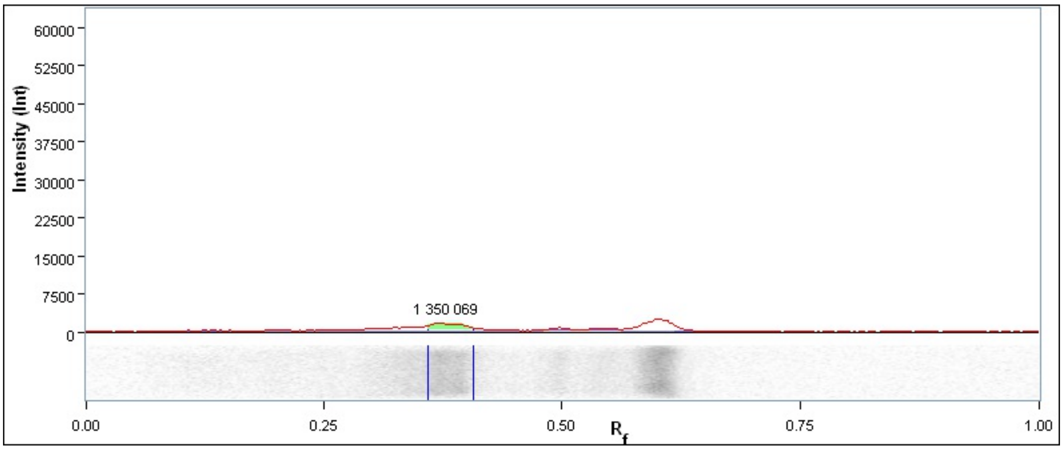

Band No.	Band Label	Mol. Wt. (KDa)	Relative Front	Volume (Int)	Abs. Quant.	Rel. Quant.	Band %	Lane %
1		N/A	0,381	1 350 069	N/A	N/A	100,0	22,3

Band Detection	Automatically detected bands with sensitivity: Low
Lane Background	Lane background subtracted with disk size: 10
Lane Width	7.39 mm

Volume Analysis

No.	Label	Type	Volume (Int)	Adj. Vol. (Int)	Mean Bkgd. (Int)	Abs. Quant.	Rel. Quant.	# of Pixels	Min. Value (Int)	Max. Value (Int)	Mean Value (Int)	Std. Dev.	Area (mm2)
1	U1	Unknown	7 352 684	4 490 300	1 192,7	N/A	N/A	2 400	0	9 724	3 063,6	1 826,4	46,6
2	U2	Unknown	8 306 684	6 132 380	906,0	N/A	N/A	2 400	0	13 708	3 461,1	2 969,3	46,6
3	U3	Unknown	10 018 196	7 928 036	870,9	N/A	N/A	2 400	0	18 560	4 174,2	4 118,1	46,6
4	U4	Unknown	4 179 164	2 782 508	581,9	N/A	N/A	2 400	0	6 772	1 741,3	1 455,4	46,6
5	U5	Unknown	3 635 424	2 376 048	524,7	N/A	N/A	2 400	0	7 052	1 514,8	1 355,9	46,6
6	U6	Unknown	7 083 280	5 556 160	636,3	N/A	N/A	2 400	0	10 576	2 951,4	2 416,2	46,6
7	U7	Unknown	2 914 192	1 498 864	589,7	N/A	N/A	2 400	0	3 364	1 214,2	722,5	46,6

Image Report: Histologia 2021-09-17 13hr 50min_Exposure_120.0sec stat3 3a

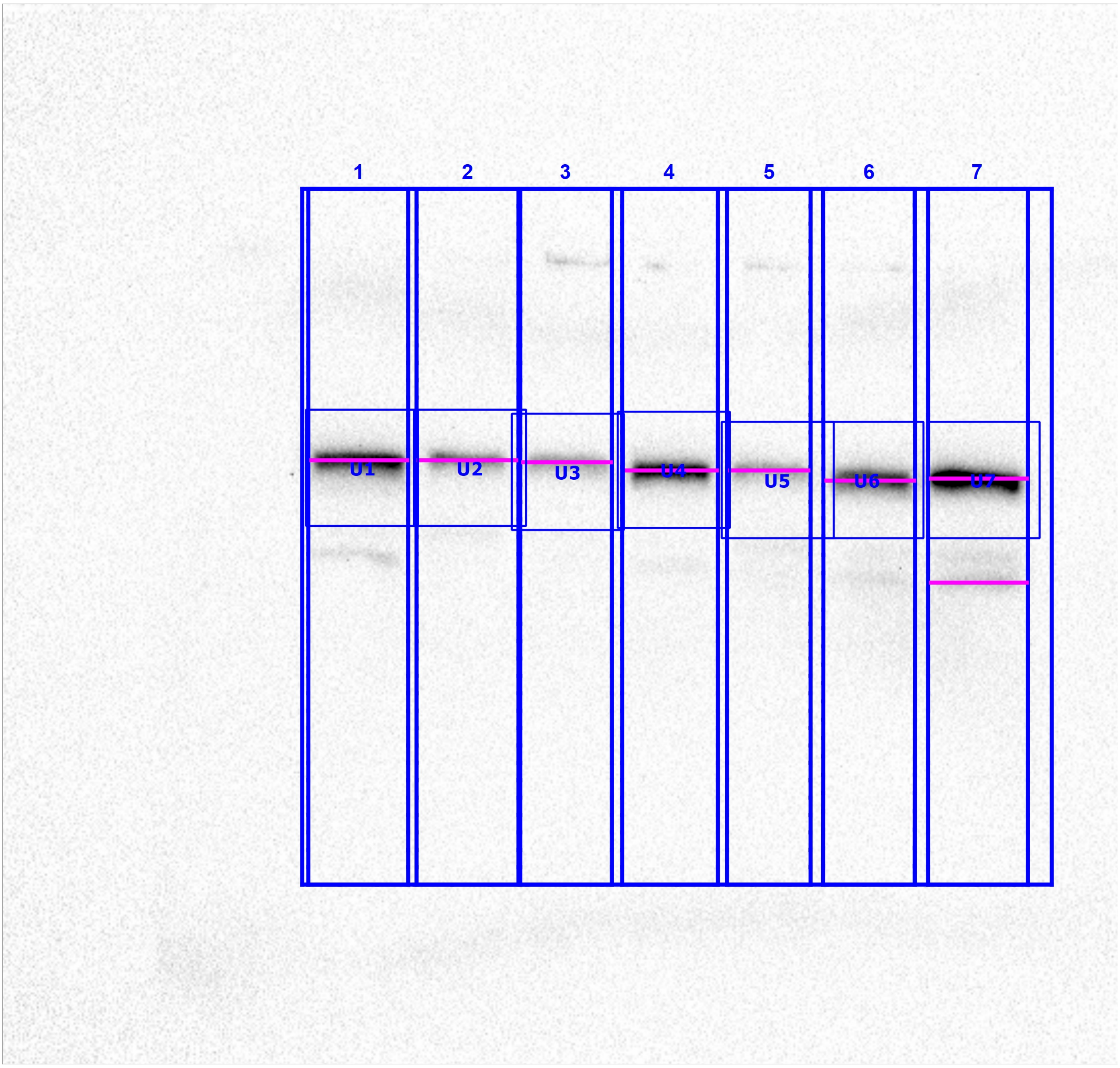

C:\Users\rusak\OneDrive\Dokumenty\Badania\CHI3L2 in BC\BC westerny ilościowo\stat-3 3 BC\Histologia 2021-09-17 13hr 50min_Exposure_120.0sec stat3 3a.scn

Acquisition Information

Imager	ChemiDoc MP
Exposure Time (sec)	119.993 (Signal Accumulation)
Flat Field	Applied (Lens)

Serial Number	731BR01769
Software Version	5.0
Application	Chemi Hi Resolution
Excitation Source	No Illumination
Emission Filter	No Filter
Binning	2x2

Image Information

Acquisition Date	17/9/2021 1:53:03 PM
User Name	Histologia
Image Area (mm)	X: 112.4 Y: 84.0
Pixel Size (μm)	X: 161.5 Y: 161.5
Data Range (Int)	0 - 49548

Analysis Settings

Detection	Lane detection:<br>Manually created lanes<br><br>Band detection:<br>Automatically detected bands with sensitivity: Low<br><br>Lane Background Subtraction:<br>Lane background subtracted with disk size: 10<br><br>Lane width: Variable
Volume Analysis	Background subtraction method: Local<br>Quantity regression method: Linear

Lane Statistics

Lane No.	Adj. Total Band Vol. (Int)	Total Band Vol. (Int)	Adj. Total Lane Vol. (Int)	Total Lane Vol. (Int)	Bkgd. Vol. (Int)	Norm. Factor
1	7 161 203	7 716 226	10 267 754	15 526 924	5 259 170	N/A
2	2 716 850	3 012 250	5 576 800	10 161 000	4 584 200	N/A
3	2 171 970	2 424 375	4 428 000	8 534 655	4 106 655	N/A
4	6 046 879	6 440 269	8 333 711	12 673 409	4 339 698	N/A
5	2 018 348	2 325 151	4 122 960	8 159 697	4 036 737	N/A
6	5 128 155	5 649 030	8 222 445	13 016 070	4 793 625	N/A
7	11 160 534	12 157 194	14 932 603	20 457 598	5 524 995	N/A

Lane And Band Analysis

Lane 1

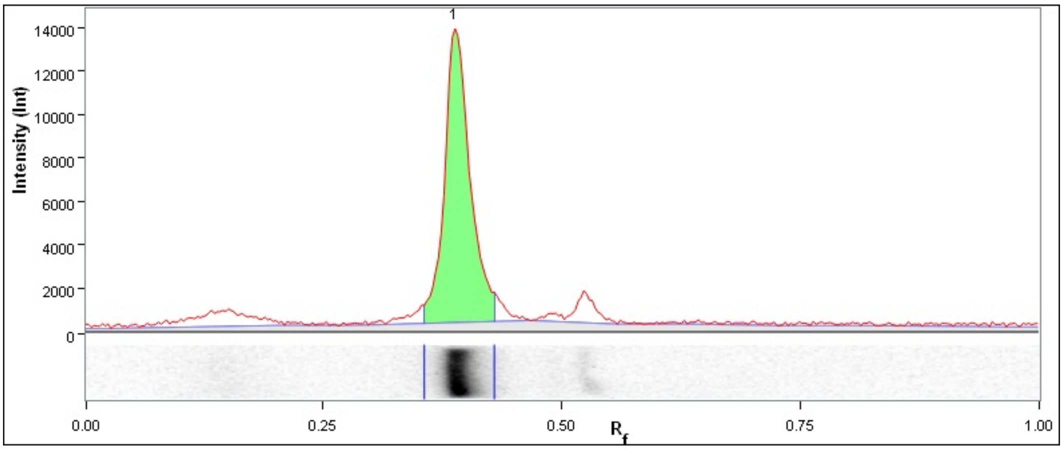

Band No.	Band Label	Mol. Wt. (KDa)	Relative Front	Adj. Volume (Int)	Volume (Int)	Abs. Quant.	Rel. Quant.	Band %	Lane %
1		N/A	0,390	7 161 203	7 716 226	N/A	N/A	100,0	69,7

Band Detection	Automatically detected bands with sensitivity: Low
Lane Background	Lane background subtracted with disk size: 10
Lane Width	7.92 mm

Lane 2

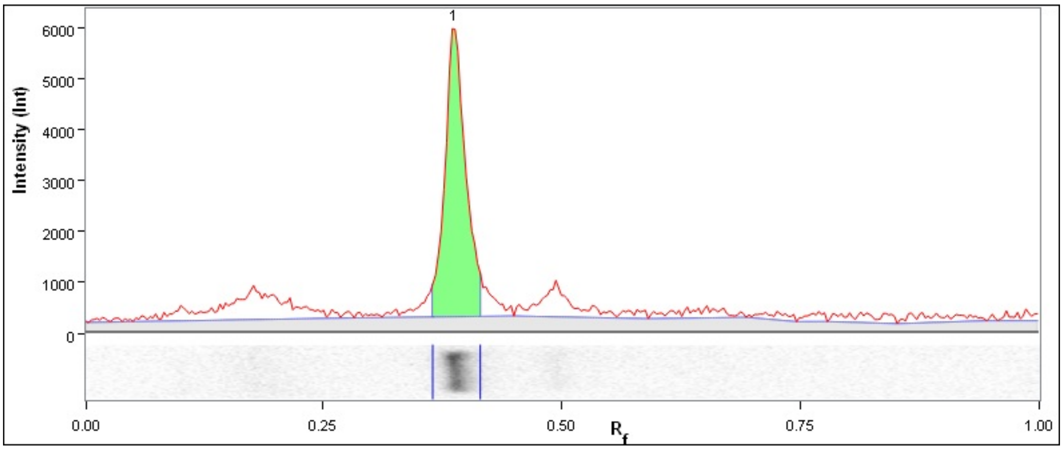

Band No.	Band Label	Mol. Wt. (KDa)	Relative Front	Adj. Volume (Int)	Volume (Int)	Abs. Quant.	Rel. Quant.	Band %	Lane %
1		N/A	0,390	2 716 850	3 012 250	N/A	N/A	100,0	48,7

Band Detection	Automatically detected bands with sensitivity: Low
Lane Background	Lane background subtracted with disk size: 10
Lane Width	8.08 mm

Lane 3

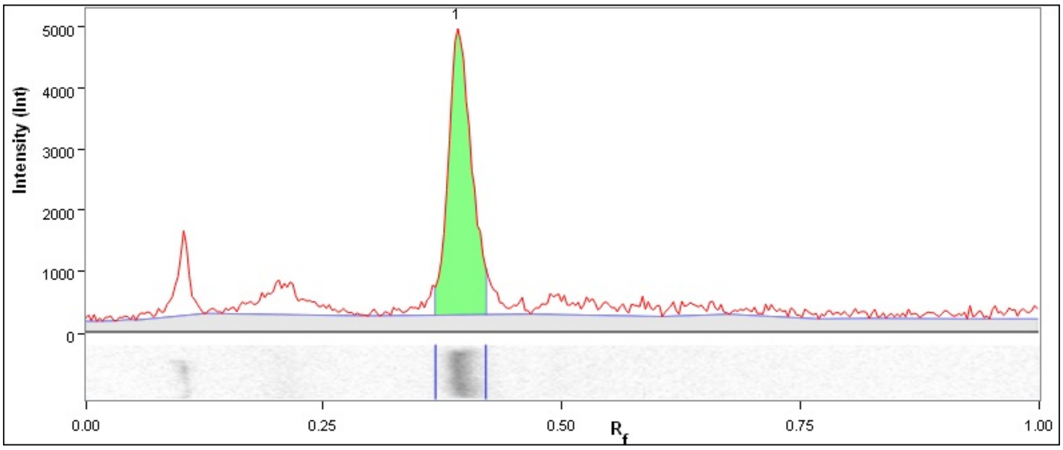

Band No.	Band Label	Mol. Wt. (KDa)	Relative Front	Adj. Volume (Int)	Volume (Int)	Abs. Quant.	Rel. Quant.	Band %	Lane %
1		N/A	0,393	2 171 970	2 424 375	N/A	N/A	100,0	49,1

Band Detection	Automatically detected bands with sensitivity: Low
Lane Background	Lane background subtracted with disk size: 10
Lane Width	7.27 mm

Lane 4

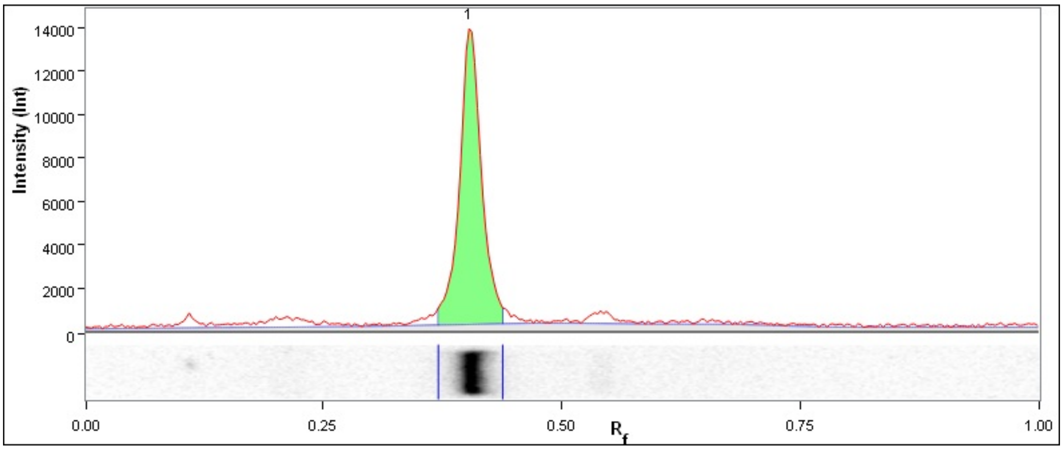

Band No.	Band Label	Mol. Wt. (KDa)	Relative Front	Adj. Volume (Int)	Volume (Int)	Abs. Quant.	Rel. Quant.	Band %	Lane %
1		N/A	0,405	6 046 879	6 440 269	N/A	N/A	100,0	72,6

Band Detection	Automatically detected bands with sensitivity: Low
Lane Background	Lane background subtracted with disk size: 10
Lane Width	7.59 mm

Lane 5

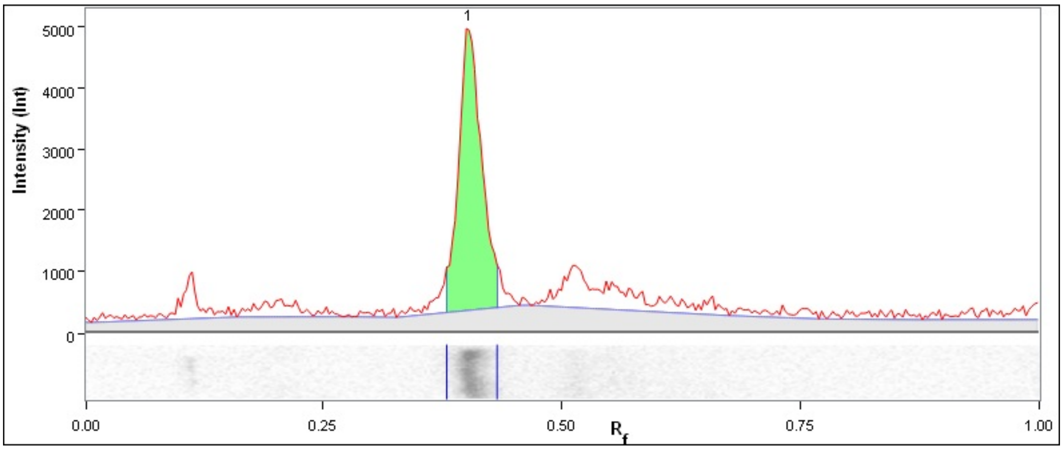

Band No.	Band Label	Mol. Wt. (KDa)	Relative Front	Adj. Volume (Int)	Volume (Int)	Abs. Quant.	Rel. Quant.	Band %	Lane %
1		N/A	0,405	2 018 348	2 325 151	N/A	N/A	100,0	49,0

Band Detection	Automatically detected bands with sensitivity: Low
Lane Background	Lane background subtracted with disk size: 10
Lane Width	6.62 mm

Lane 6

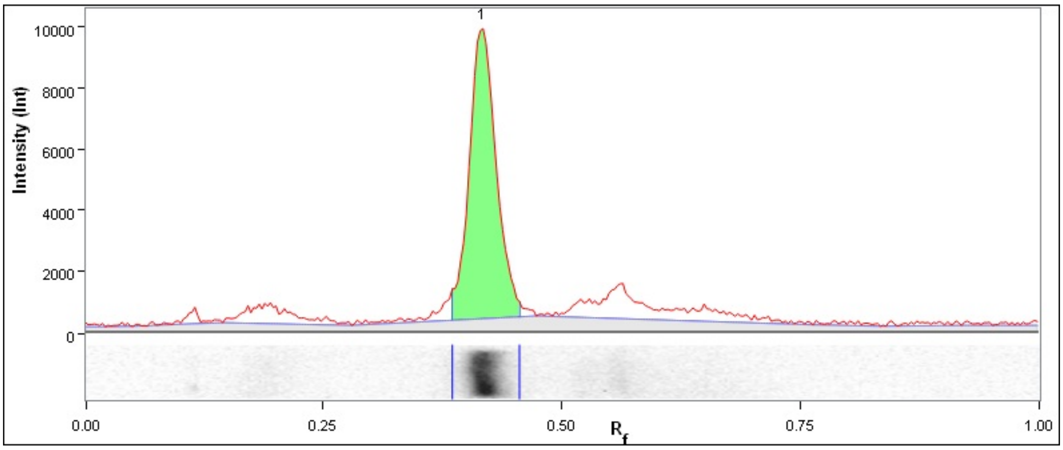

Band No.	Band Label	Mol. Wt. (KDa)	Relative Front	Adj. Volume (Int)	Volume (Int)	Abs. Quant.	Rel. Quant.	Band %	Lane %
1		N/A	0,419	5 128 155	5 649 030	N/A	N/A	100,0	62,4

Band Detection	Automatically detected bands with sensitivity: Low
Lane Background	Lane background subtracted with disk size: 10
Lane Width	7.27 mm

Lane 7

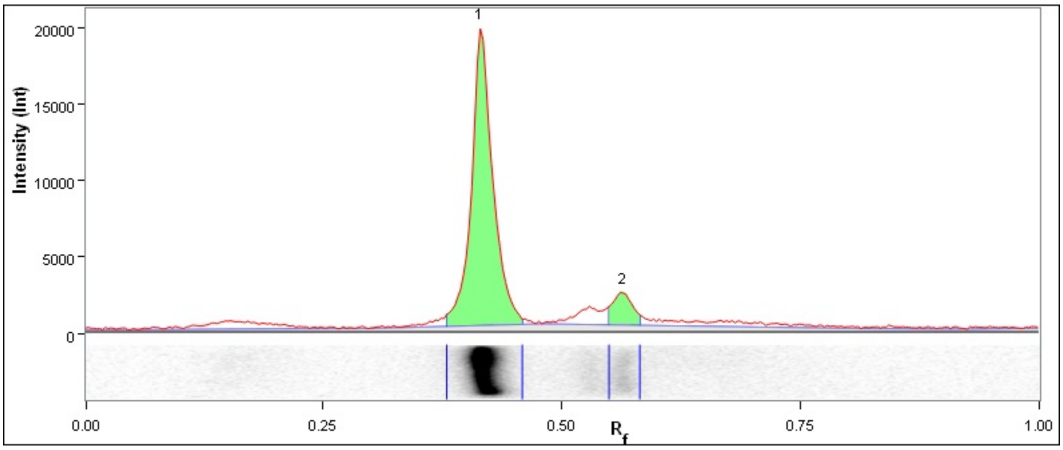

Band No.	Band Label	Mol. Wt. (KDa)	Relative Front	Adj. Volume (Int)	Volume (Int)	Abs. Quant.	Rel. Quant.	Band %	Lane %
1		N/A	0,416	10 104 290	10 791 613	N/A	N/A	90,5	67,7
2		N/A	0,566	1 056 244	1 365 581	N/A	N/A	9,5	7,1

Band Detection	Automatically detected bands with sensitivity: Low
Lane Background	Lane background subtracted with disk size: 10
Lane Width	7.92 mm

Volume Analysis

No.	Label	Type	Volume (Int)	Adj. Vol. (Int)	Mean Bkgd. (Int)	Abs. Quant.	Rel. Quant.	# of Pixels	Min. Value (Int)	Max. Value (Int)	Mean Value (Int)	Std. Dev.	Area (mm2)
1	U1	Unknown	9 193 504	7 114 669	663,1	N/A	N/A	3 135	0	19 488	2 932,5	4 054,8	81,8
2	U2	Unknown	4 315 720	2 088 055	710,6	N/A	N/A	3 135	0	11 240	1 376,6	1 926,4	81,8
3	U3	Unknown	3 597 964	1 890 874	544,5	N/A	N/A	3 135	0	7 684	1 147,7	1 474,5	81,8
4	U4	Unknown	7 611 464	5 920 434	539,4	N/A	N/A	3 135	0	19 384	2 427,9	4 054,3	81,8
5	U5	Unknown	3 835 032	830 217	958,5	N/A	N/A	3 135	0	7 632	1 223,3	1 457,2	81,8
6	U6	Unknown	7 048 300	4 544 480	798,7	N/A	N/A	3 135	0	14 476	2 248,3	3 180,1	81,8
7	U7	Unknown	12 006 700	9 556 175	781,7	N/A	N/A	3 135	0	39 316	3 829,9	6 461,3	81,8

Image Report: Histologia 2021-09-17 13hr 50min_Exposure_120.0sec stat3 3c

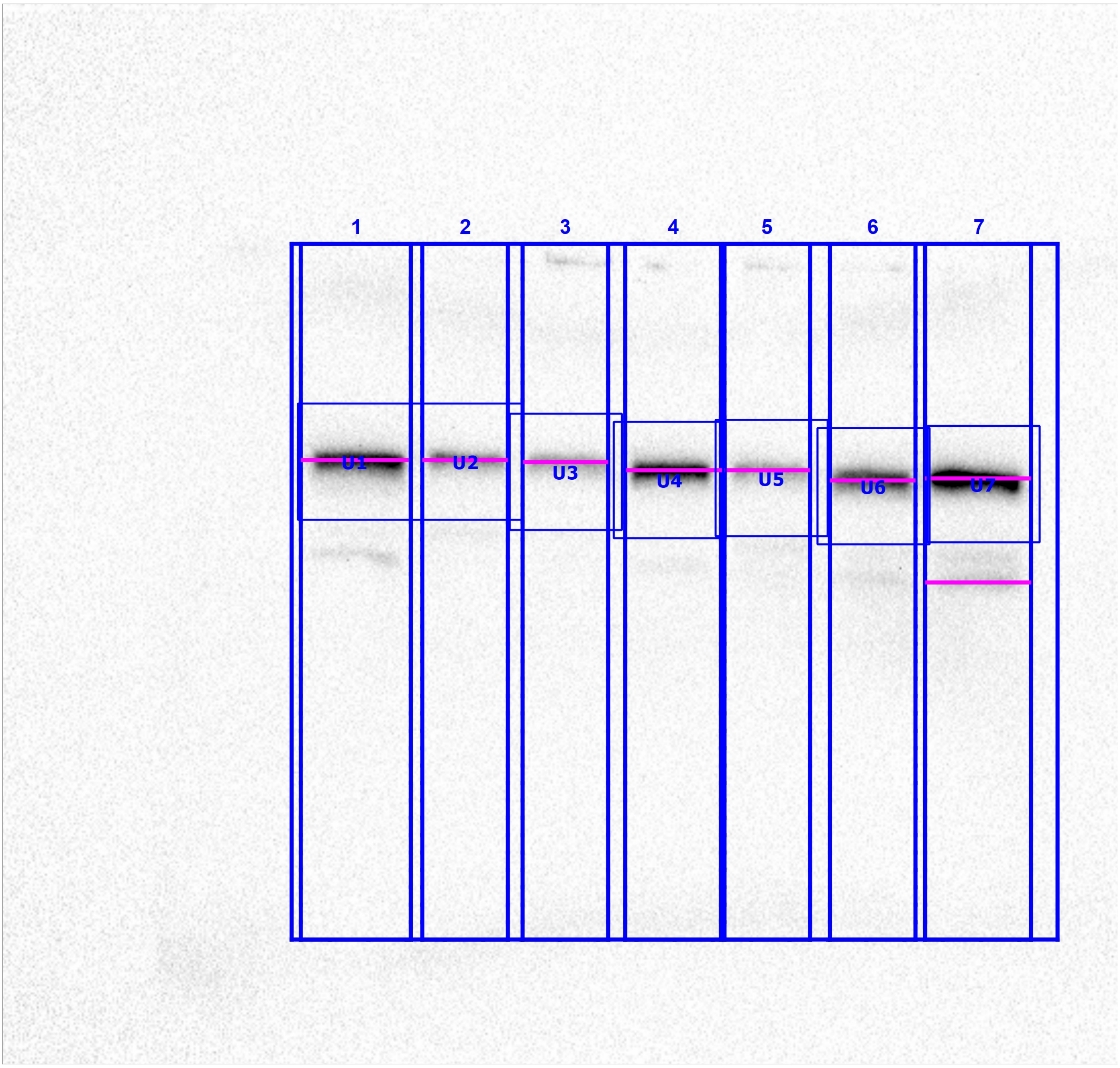

C:\Users\rusak\OneDrive\Dokumenty\Badania\CHI3L2 in BC\BC westerny ilościowo\analiza STAT3\stat-3 3\Histologia 2021-09-17 13hr 50min_Exposure_120.0sec stat3 3c.scn

---

Acquisition Information

Imager	ChemiDoc MP
Exposure Time (sec)	119.993 (Signal Accumulation)
Flat Field	Applied (Lens)

Serial Number	731BR01769
Software Version	5.0
Application	Chemi Hi Resolution
Excitation Source	No Illumination
Emission Filter	No Filter
Binning	2x2

Image Information

Acquisition Date	17/9/2021 1:53:03 PM
User Name	Histologia
Image Area (mm)	X: 112.4 Y: 84.0
Pixel Size (µm)	X: 161.5 Y: 161.5
Data Range (Int)	0 - 49548

Analysis Settings

Detection	Lane detection:<br>Manually created lanes<br><br>Band detection:<br>Automatically detected bands with sensitivity: Low<br><br>Lane Background Subtraction:<br>Lane background subtracted with disk size: 10<br><br>Lane width: Variable
Volume Analysis	Background subtraction method: Local<br>Quantity regression method: Linear

Lane Statistics

Lane No.	Adj. Total Band Vol. (Int)	Total Band Vol. (Int)	Adj. Total Lane Vol. (Int)	Total Lane Vol. (Int)	Bkgd. Vol. (Int)	Norm. Factor
1	7 249 608	7 874 928	10 304 496	16 483 986	6 179 490	N/A
2	2 685 060	2 970 114	5 402 250	9 287 628	3 885 378	N/A
3	2 152 836	2 402 022	4 739 826	8 505 084	3 765 258	N/A
4	6 057 360	6 444 499	8 615 053	13 142 657	4 527 604	N/A
5	2 033 892	2 345 868	4 337 382	8 731 338	4 393 956	N/A
6	5 156 760	5 638 542	8 176 308	12 916 596	4 740 288	N/A
7	11 244 272	12 295 504	15 184 104	21 312 408	6 128 304	N/A

Lane And Band Analysis

Lane 1

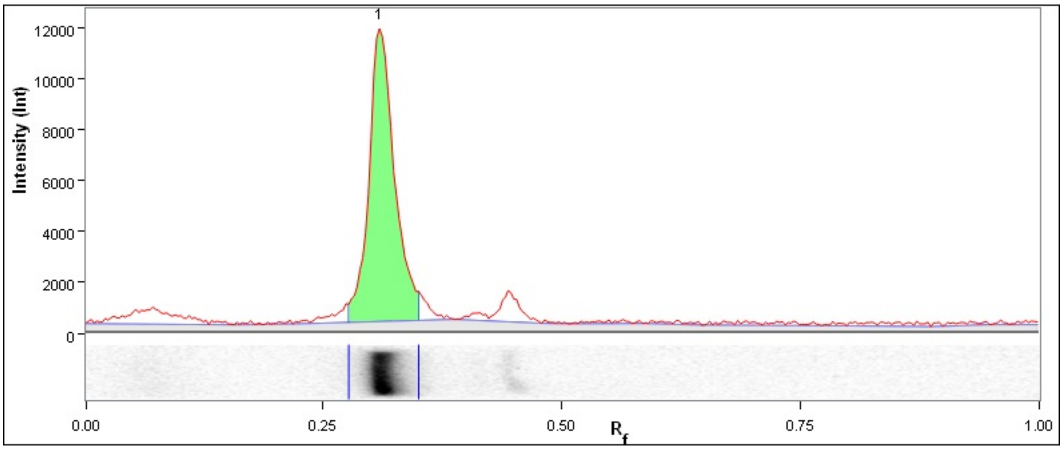

Band No.	Band Label	Mol. Wt. (KDa)	Relative Front	Adj. Volume (Int)	Volume (Int)	Abs. Quant.	Rel. Quant.	Band %	Lane %
1		N/A	0,311	7 249 608	7 874 928	N/A	N/A	100,0	70,4

Band Detection	Automatically detected bands with sensitivity: Low
Lane Background	Lane background subtracted with disk size: 10
Lane Width	8.72 mm

Lane 2

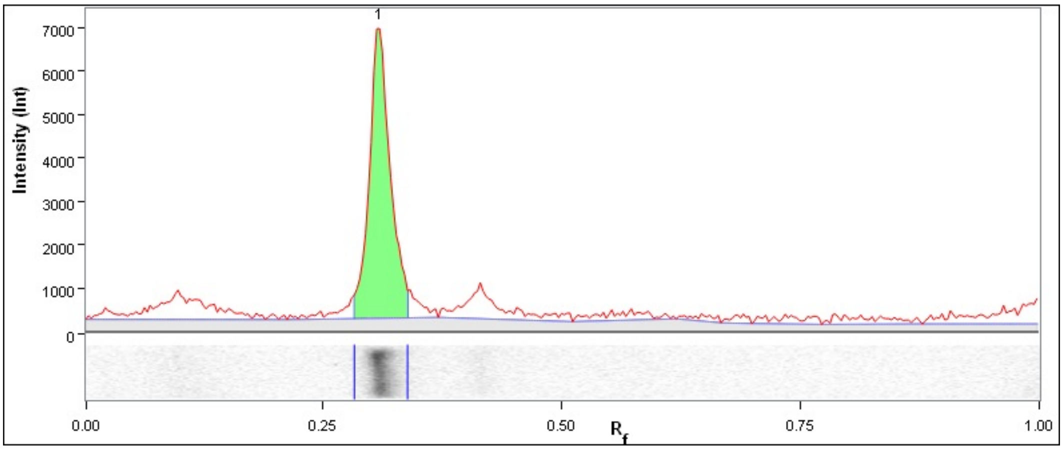

Band No.	Band Label	Mol. Wt. (KDa)	Relative Front	Adj. Volume (Int)	Volume (Int)	Abs. Quant.	Rel. Quant.	Band %	Lane %
1		N/A	0,311	2 685 060	2 970 114	N/A	N/A	100,0	49,7

Band Detection	Automatically detected bands with sensitivity: Low
Lane Background	Lane background subtracted with disk size: 10
Lane Width	6.78 mm

Lane 3

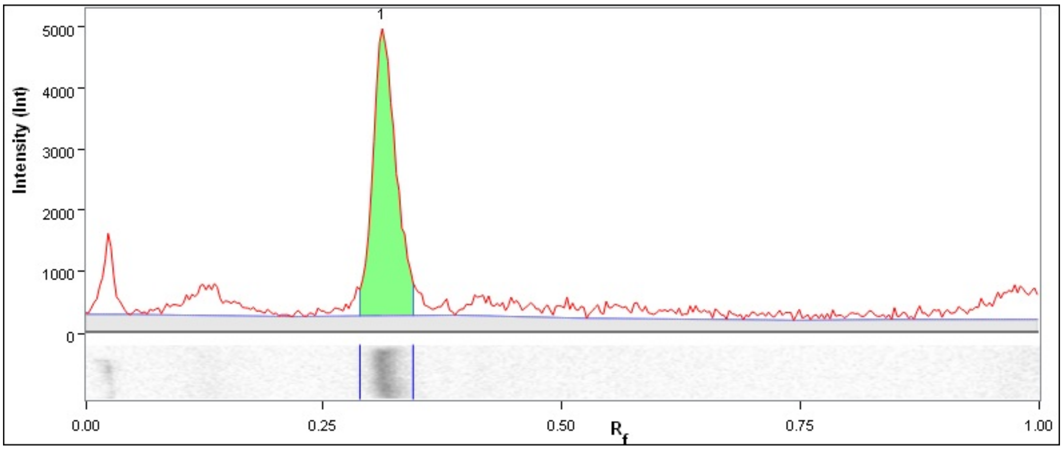

Band No.	Band Label	Mol. Wt. (KDa)	Relative Front	Adj. Volume (Int)	Volume (Int)	Abs. Quant.	Rel. Quant.	Band %	Lane %
1		N/A	0,314	2 152 836	2 402 022	N/A	N/A	100,0	45,4

Band Detection	Automatically detected bands with sensitivity: Low
Lane Background	Lane background subtracted with disk size: 10
Lane Width	6.78 mm

Lane 4

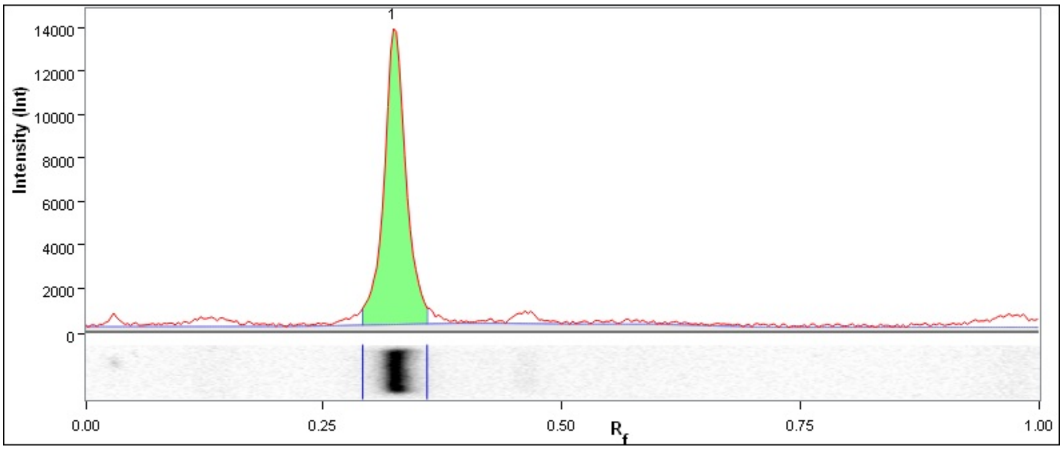

Band No.	Band Label	Mol. Wt. (KDa)	Relative Front	Adj. Volume (Int)	Volume (Int)	Abs. Quant.	Rel. Quant.	Band %	Lane %
1		N/A	0,326	6 057 360	6 444 499	N/A	N/A	100,0	70,3

Band Detection	Automatically detected bands with sensitivity: Low
Lane Background	Lane background subtracted with disk size: 10
Lane Width	7.59 mm

Lane 5

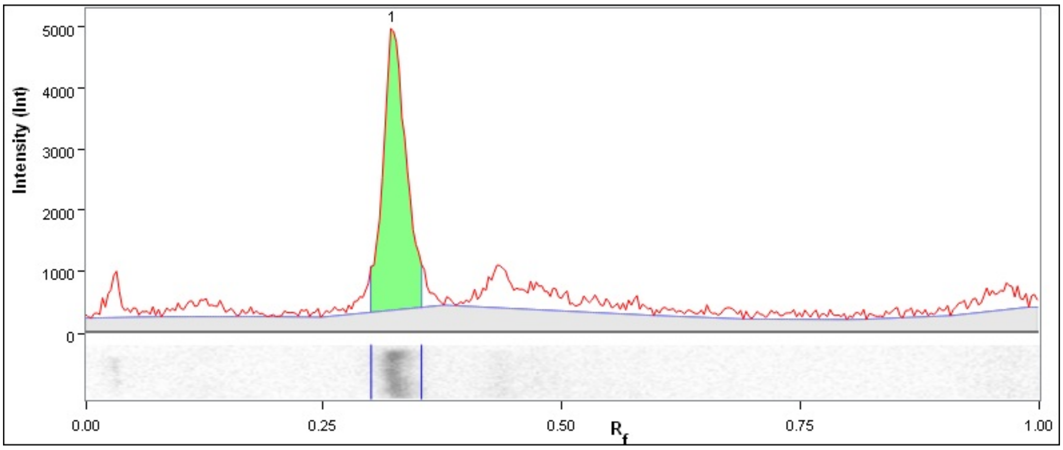

Band No.	Band Label	Mol. Wt. (KDa)	Relative Front	Adj. Volume (Int)	Volume (Int)	Abs. Quant.	Rel. Quant.	Band %	Lane %
1		N/A	0,326	2 033 892	2 345 868	N/A	N/A	100,0	46,9

Band Detection	Automatically detected bands with sensitivity: Low
Lane Background	Lane background subtracted with disk size: 10
Lane Width	6.78 mm

Lane 6

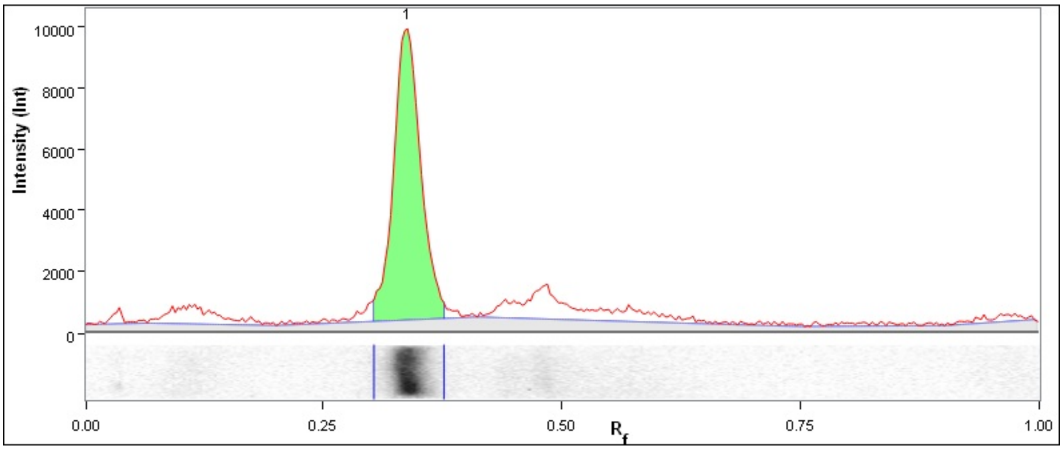

Band No.	Band Label	Mol. Wt. (KDa)	Relative Front	Adj. Volume (Int)	Volume (Int)	Abs. Quant.	Rel. Quant.	Band %	Lane %
1		N/A	0,340	5 156 760	5 638 542	N/A	N/A	100,0	63,1

Band Detection	Automatically detected bands with sensitivity: Low
Lane Background	Lane background subtracted with disk size: 10
Lane Width	6.78 mm

Lane 7

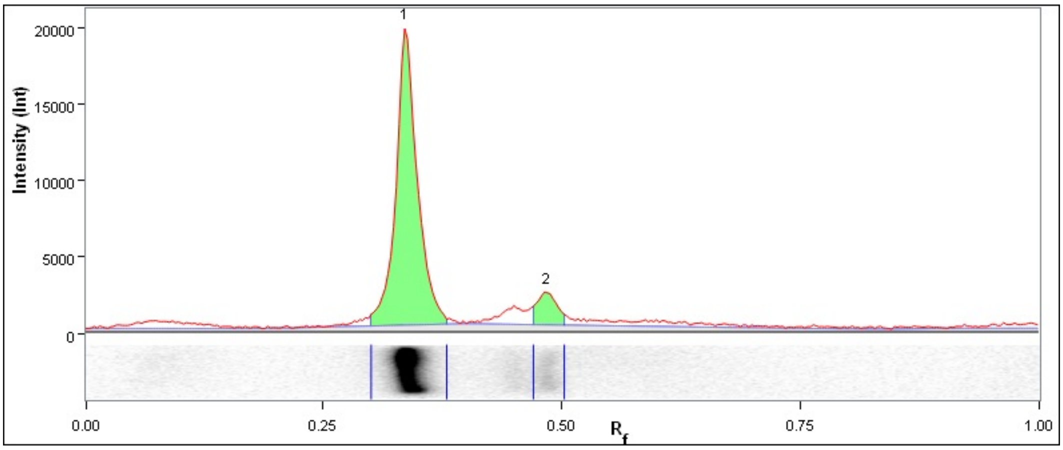

Band No.	Band Label	Mol. Wt. (KDa)	Relative Front	Adj. Volume (Int)	Volume (Int)	Abs. Quant.	Rel. Quant.	Band %	Lane %
1		N/A	0,337	10 171 044	10 900 864	N/A	N/A	90,5	67,0
2		N/A	0,487	1 073 228	1 394 640	N/A	N/A	9,5	7,1

Band Detection	Automatically detected bands with sensitivity: Low
Lane Background	Lane background subtracted with disk size: 10
Lane Width	8.40 mm

Volume Analysis

No.	Label	Type	Volume (Int)	Adj. Vol. (Int)	Mean Bkgd. (Int)	Abs. Quant.	Rel. Quant.	# of Pixels	Min. Value (Int)	Max. Value (Int)	Mean Value (Int)	Std. Dev.	Area (mm2)
1	U1	Unknown	9 147 896	7 260 901	601,9	N/A	N/A	3 135	0	19 488	2 918,0	4 062,3	81,8
2	U2	Unknown	4 280 224	2 268 269	641,8	N/A	N/A	3 135	0	11 240	1 365,3	1 931,5	81,8
3	U3	Unknown	3 594 660	1 919 800	534,2	N/A	N/A	3 135	0	7 684	1 146,6	1 475,0	81,8
4	U4	Unknown	7 635 004	5 922 194	546,4	N/A	N/A	3 135	0	19 384	2 435,4	4 050,8	81,8
5	U5	Unknown	3 733 100	1 630 725	670,6	N/A	N/A	3 135	0	7 632	1 190,8	1 440,6	81,8
6	U6	Unknown	7 250 808	3 813 088	1 096,6	N/A	N/A	3 135	0	14 476	2 312,9	3 171,3	81,8
7	U7	Unknown	12 055 200	9 389 680	850,2	N/A	N/A	3 135	0	39 316	3 845,4	6 454,1	81,8

Image Report: Histologia 2021-09-21 13hr 43min_Exposure_120.0sec stat3 1a

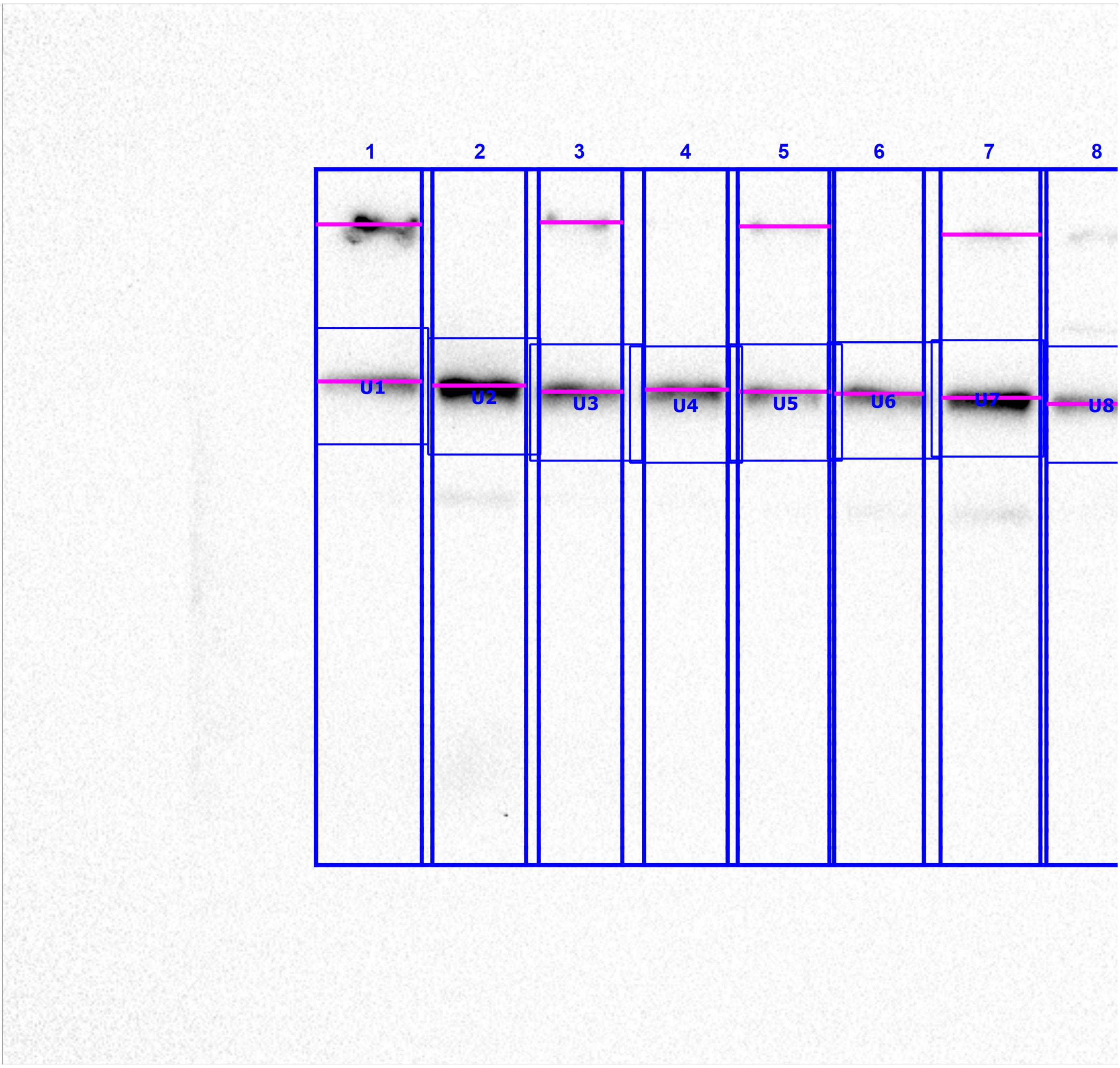

C:\Users\rusak\OneDrive\Dokumenty\Badania\CHI3L2 in BC\BC westerny ilościowo\stat3 1 BC\stat3 1\Histologia 2021-09-21 13hr 43min_Exposure_120.0sec stat3 1a.scn

Acquisition Information

Imager	ChemiDoc MP
Exposure Time (sec)	119.993 (Signal Accumulation)
Flat Field	Applied (Lens)

Serial Number	731BR01769
Software Version	5.0
Application	Chemi Hi Resolution
Excitation Source	No Illumination
Emission Filter	No Filter
Binning	2x2

Image Information

Acquisition Date	21/9/2021 1:46:08 PM
User Name	Histologia
Image Area (mm)	X: 112.0 Y: 83.7
Pixel Size (µm)	X: 160.9 Y: 160.9
Data Range (Int)	0 - 49324

Analysis Settings

Detection	Lane detection:<br>Manually created lanes<br><br>Band detection:<br>Automatically detected bands with sensitivity: Low<br><br>Lane Background Subtraction:<br>Lane background subtracted with disk size: 10<br><br>Lane width: Variable
Volume Analysis	Background subtraction method: Local<br>Quantity regression method: Linear

Lane Statistics

Lane No.	Adj. Total Band Vol. (Int)	Total Band Vol. (Int)	Adj. Total Lane Vol. (Int)	Total Lane Vol. (Int)	Bkgd. Vol. (Int)	Norm. Factor
1	9 974 068	10 657 244	12 077 312	16 637 192	4 559 880	N/A
2	11 843 758	12 198 004	15 264 134	18 679 956	3 415 822	N/A
3	6 660 573	7 003 989	8 571 009	11 295 377	2 724 368	N/A
4	5 723 190	5 978 989	7 234 122	10 030 486	2 796 364	N/A
5	4 081 140	4 351 725	5 857 515	8 589 150	2 731 635	N/A
6	4 822 268	5 036 284	6 712 904	9 402 668	2 689 764	N/A
7	10 004 036	10 422 594	12 188 064	15 791 720	3 603 656	N/A
8	3 921 550	4 144 250	6 145 200	9 363 800	3 218 600	N/A

Lane And Band Analysis

Lane 1

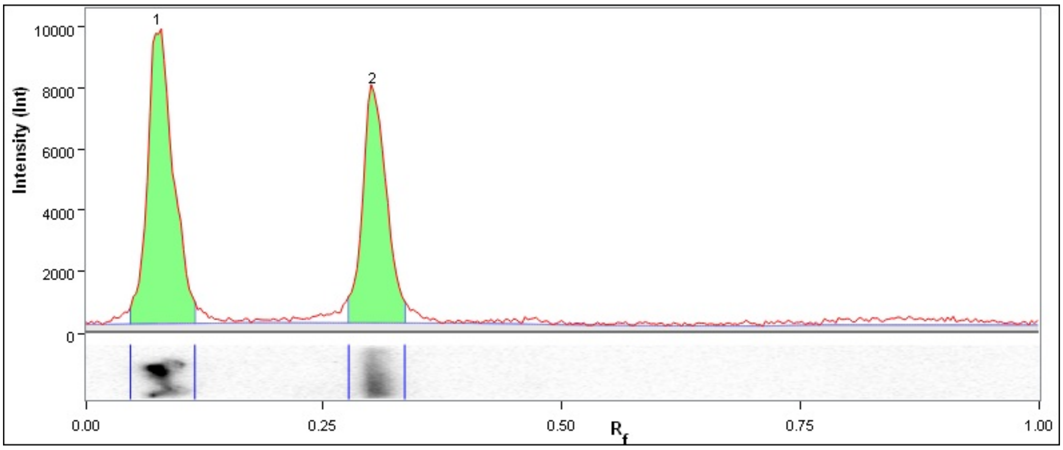

Band No.	Band Label	Mol. Wt. (KDa)	Relative Front	Adj. Volume (Int)	Volume (Int)	Abs. Quant.	Rel. Quant.	Band %	Lane %
1		N/A	0,079	5 716 672	6 065 852	N/A	N/A	57,3	47,3
2		N/A	0,305	4 257 396	4 591 392	N/A	N/A	42,7	35,3

Band Detection	Automatically detected bands with sensitivity: Low
Lane Background	Lane background subtracted with disk size: 10
Lane Width	8.37 mm

Lane 2

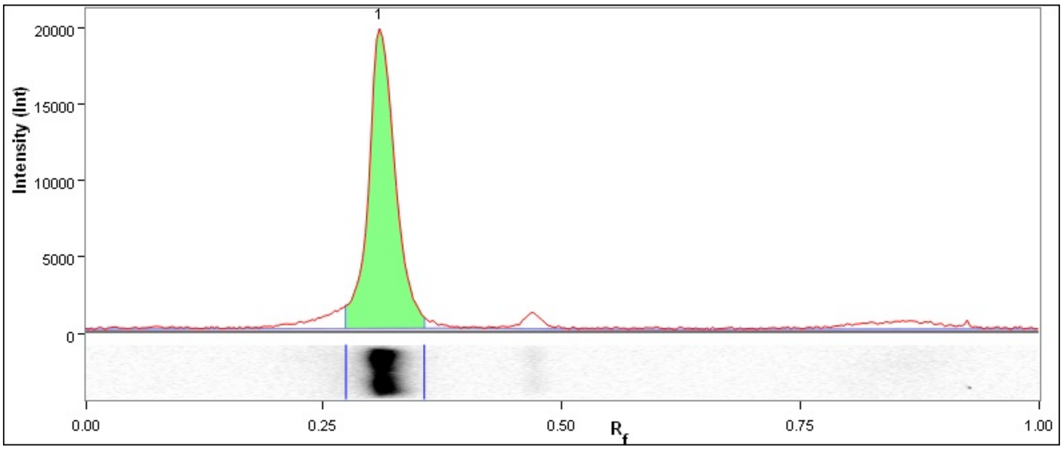

Band No.	Band Label	Mol. Wt. (KDa)	Relative Front	Adj. Volume (Int)	Volume (Int)	Abs. Quant.	Rel. Quant.	Band %	Lane %
1		N/A	0,311	11 843 758	12 198 004	N/A	N/A	100,0	77,6

Band Detection	Automatically detected bands with sensitivity: Low
Lane Background	Lane background subtracted with disk size: 10
Lane Width	7.40 mm

Lane 3

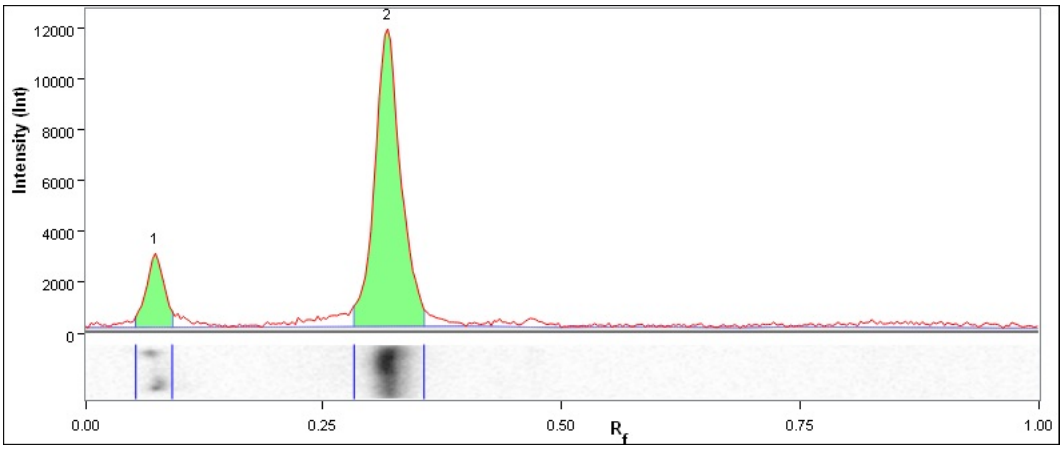

Band No.	Band Label	Mol. Wt. (KDa)	Relative Front	Adj. Volume (Int)	Volume (Int)	Abs. Quant.	Rel. Quant.	Band %	Lane %
1		N/A	0,076	972 766	1 080 063	N/A	N/A	14,6	11,3
2		N/A	0,320	5 687 807	5 923 926	N/A	N/A	85,4	66,4

Band Detection	Automatically detected bands with sensitivity: Low
Lane Background	Lane background subtracted with disk size: 10
Lane Width	6.60 mm

Lane 4

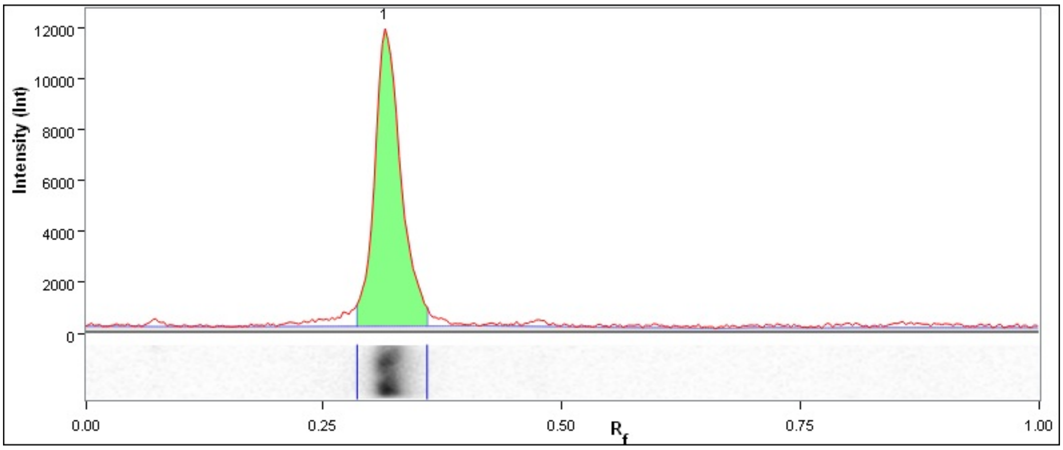

Band No.	Band Label	Mol. Wt. (KDa)	Relative Front	Adj. Volume (Int)	Volume (Int)	Abs. Quant.	Rel. Quant.	Band %	Lane %
1		N/A	0,317	5 723 190	5 978 989	N/A	N/A	100,0	79,1

Band Detection	Automatically detected bands with sensitivity: Low
Lane Background	Lane background subtracted with disk size: 10
Lane Width	6.60 mm

Lane 5

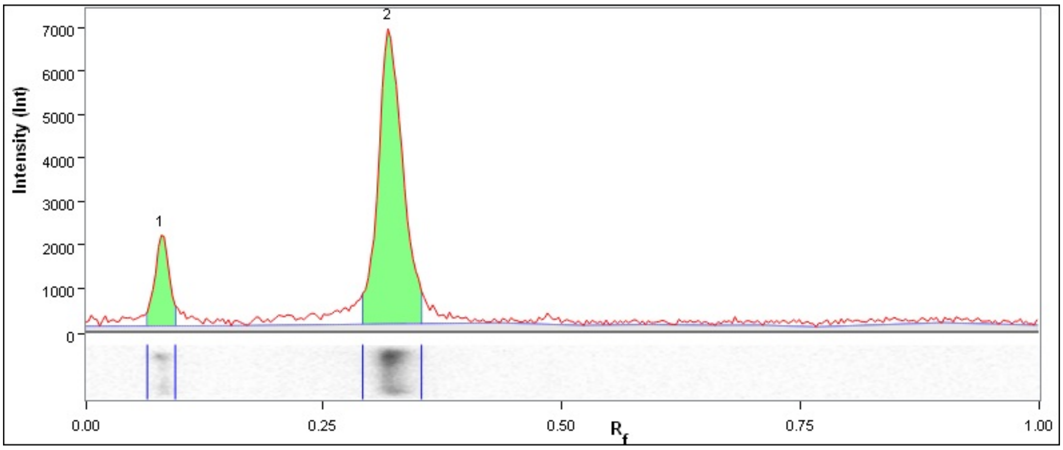

Band No.	Band Label	Mol. Wt. (KDa)	Relative Front	Adj. Volume (Int)	Volume (Int)	Abs. Quant.	Rel. Quant.	Band %	Lane %
1		N/A	0,082	630 360	704 160	N/A	N/A	15,4	10,8
2		N/A	0,320	3 450 780	3 647 565	N/A	N/A	84,6	58,9

Band Detection	Automatically detected bands with sensitivity: Low
Lane Background	Lane background subtracted with disk size: 10
Lane Width	7.24 mm

Lane 6

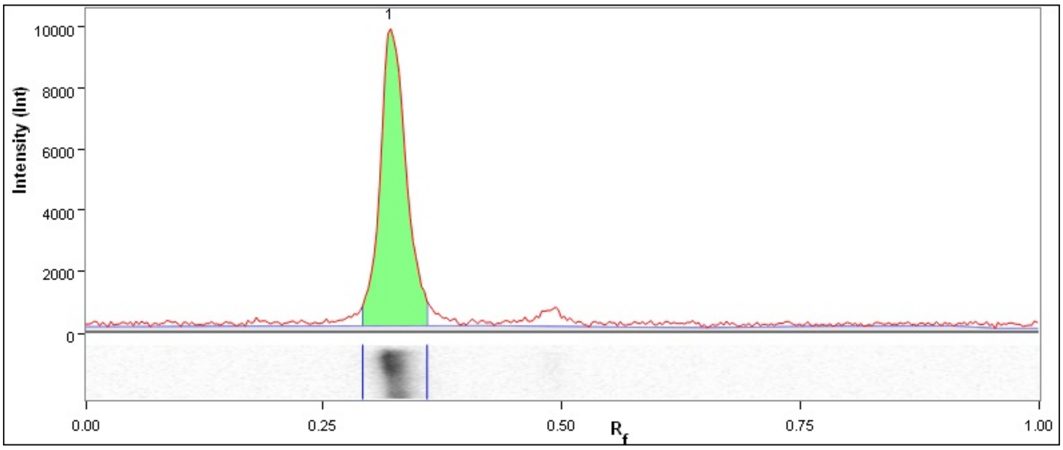

Band No.	Band Label	Mol. Wt. (KDa)	Relative Front	Adj. Volume (Int)	Volume (Int)	Abs. Quant.	Rel. Quant.	Band %	Lane %
1		N/A	0,323	4 822 268	5 036 284	N/A	N/A	100,0	71,8

Band Detection	Automatically detected bands with sensitivity: Low
Lane Background	Lane background subtracted with disk size: 10
Lane Width	7.08 mm

Lane 7

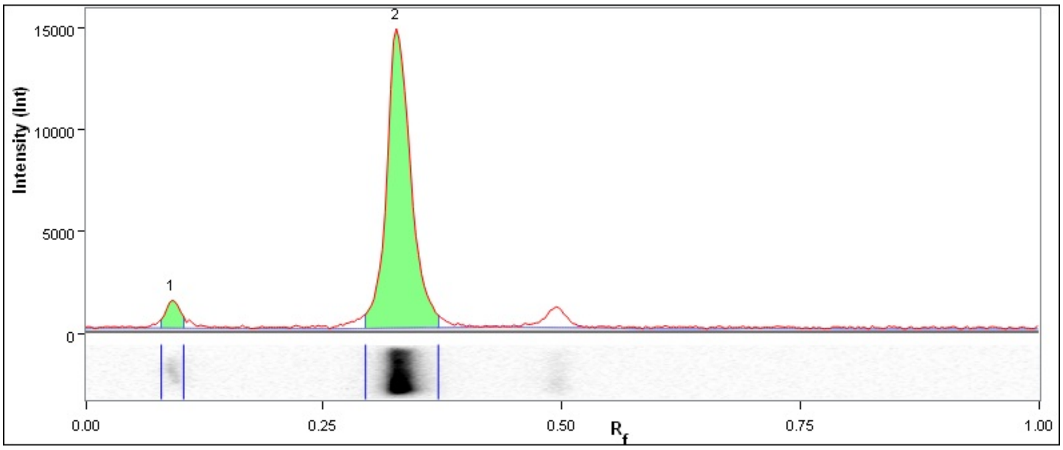

Band No.	Band Label	Mol. Wt. (KDa)	Relative Front	Adj. Volume (Int)	Volume (Int)	Abs. Quant.	Rel. Quant.	Band %	Lane %
1		N/A	0,094	533 120	635 089	N/A	N/A	5,3	4,4
2		N/A	0,328	9 470 916	9 787 505	N/A	N/A	94,7	77,7

Band Detection	Automatically detected bands with sensitivity: Low
Lane Background	Lane background subtracted with disk size: 10
Lane Width	7.89 mm

Lane 8

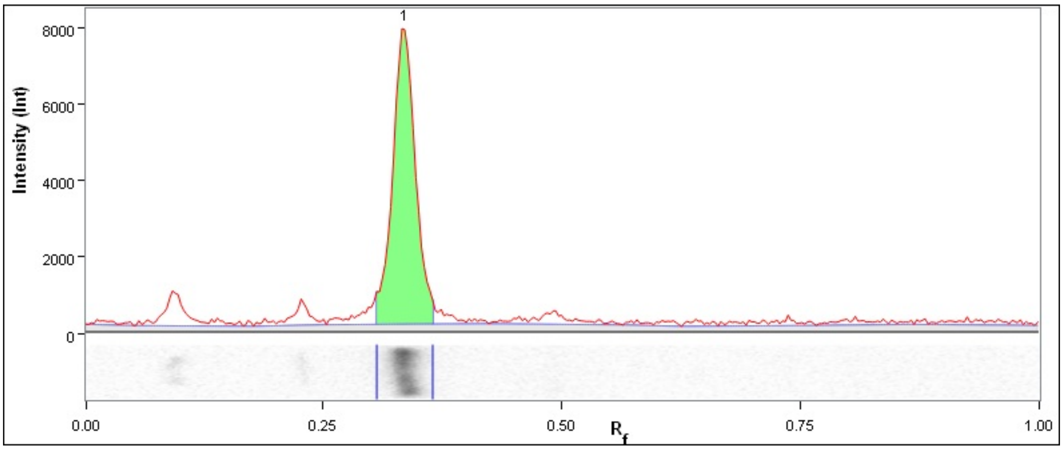

Band No.	Band Label	Mol. Wt. (KDa)	Relative Front	Adj. Volume (Int)	Volume (Int)	Abs. Quant.	Rel. Quant.	Band %	Lane %
1		N/A	0,337	3 921 550	4 144 250	N/A	N/A	100,0	63,8

Band Detection	Automatically detected bands with sensitivity: Low
Lane Background	Lane background subtracted with disk size: 10
Lane Width	8.05 mm

Volume Analysis

No.	Label	Type	Volume (Int)	Adj. Vol. (Int)	Mean Bkgd. (Int)	Abs. Quant.	Rel. Quant.	# of Pixels	Min. Value (Int)	Max. Value (Int)	Mean Value (Int)	Std. Dev.	Area (mm2)
1	U1	Unknown	5 820 340	3 905 185	610,9	N/A	N/A	3 135	0	13 916	1 856,6	2 629,4	81,2
2	U2	Unknown	13 997 372	10 213 427	1 207,0	N/A	N/A	3 135	0	35 484	4 464,9	6 928,1	81,2

3	U3	Unknown	7 395 468	4 596 188	892,9	N/A	N/A	3 135	0	17 344	2 359,0	3 489,6	81,2
4	U4	Unknown	7 298 624	5 119 909	695,0	N/A	N/A	3 135	0	18 296	2 328,1	3 507,8	81,2
5	U5	Unknown	4 851 548	2 468 453	760,2	N/A	N/A	3 135	0	13 748	1 547,5	2 244,5	81,2
6	U6	Unknown	6 228 112	4 673 152	496,0	N/A	N/A	3 135	0	15 040	1 986,6	3 035,1	81,2
7	U7	Unknown	10 803 460	8 984 940	580,1	N/A	N/A	3 135	0	33 636	3 446,1	5 876,9	81,2
8	U8	Unknown	5 035 376	3 681 276	431,9	N/A	N/A	3 135	0	12 632	1 606,2	2 463,0	81,2

Image Report: Histologia 2021-09-21 13hr 43min_Exposure_120.0sec stat3 1c

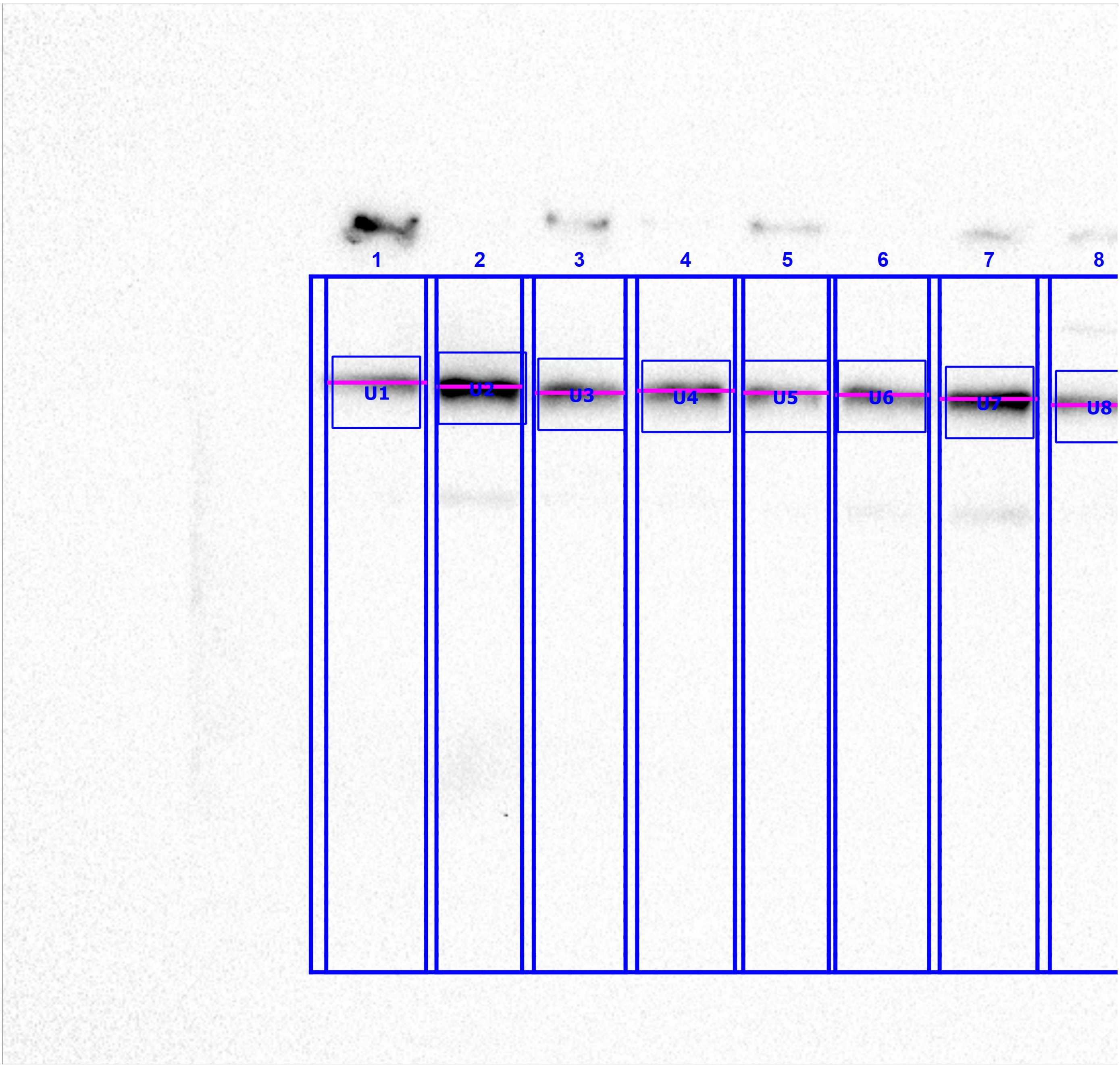

C:\Users\rusak\OneDrive\Dokumenty\Badania\CHI3L2 in BC\BC westerny ilościowo\analiza STAT3\Histologia 2021-09-21 13hr 43min_Exposure_120.0sec stat3 1c.scn

Acquisition Information

Imager	ChemiDoc MP
Exposure Time (sec)	119.993 (Signal Accumulation)
Flat Field	Applied (Lens)

Serial Number	731BR01769
Software Version	5.0
Application	Chemi Hi Resolution
Excitation Source	No Illumination
Emission Filter	No Filter
Binning	2x2

Image Information

Acquisition Date	21/9/2021 1:46:08 PM
User Name	Histologia
Image Area (mm)	X: 112.0 Y: 83.7
Pixel Size (μm)	X: 160.9 Y: 160.9
Data Range (Int)	0 - 49324

Analysis Settings

Detection	Lane detection:<br>Manually created lanes<br><br>Band detection:<br>Automatically detected bands with sensitivity: Low<br><br>Lane Background Subtraction:<br>Lane background subtracted with disk size: 10<br><br>Lane width: Variable
Volume Analysis	Background subtraction method: Local<br>Quantity regression method: Linear

Lane Statistics

Lane No.	Adj. Total Band Vol. (Int)	Total Band Vol. (Int)	Adj. Total Lane Vol. (Int)	Total Lane Vol. (Int)	Bkgd. Vol. (Int)	Norm. Factor
1	4 223 996	4 540 977	6 316 541	10 401 916	4 085 375	N/A
2	11 550 378	11 885 538	14 740 068	17 898 510	3 158 442	N/A
3	5 852 970	6 107 895	7 846 425	10 848 150	3 001 725	N/A
4	5 959 152	6 263 664	7 576 272	10 930 704	3 354 432	N/A
5	3 313 086	3 495 030	4 851 378	7 518 840	2 667 462	N/A
6	4 970 024	5 187 558	6 873 918	9 684 104	2 810 186	N/A
7	9 423 936	9 739 632	11 503 344	14 948 160	3 444 816	N/A
8	3 898 244	4 117 862	5 937 330	8 992 137	3 054 807	N/A

Lane And Band Analysis

Lane 1

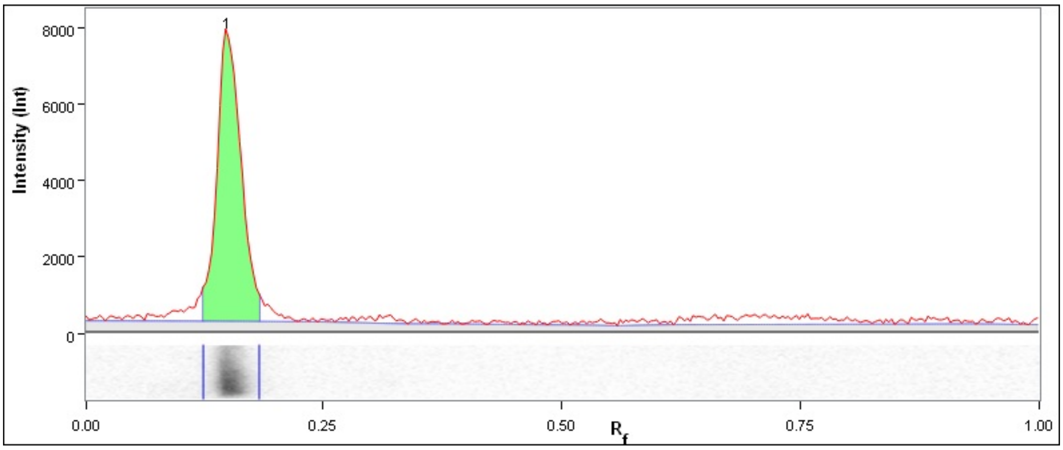

Band No.	Band Label	Mol. Wt. (KDa)	Relative Front	Adj. Volume (Int)	Volume (Int)	Abs. Quant.	Rel. Quant.	Band %	Lane %
1		N/A	0,152	4 223 996	4 540 977	N/A	N/A	100,0	66,9

Band Detection	Automatically detected bands with sensitivity: Low
Lane Background	Lane background subtracted with disk size: 10
Lane Width	7.89 mm

Lane 2

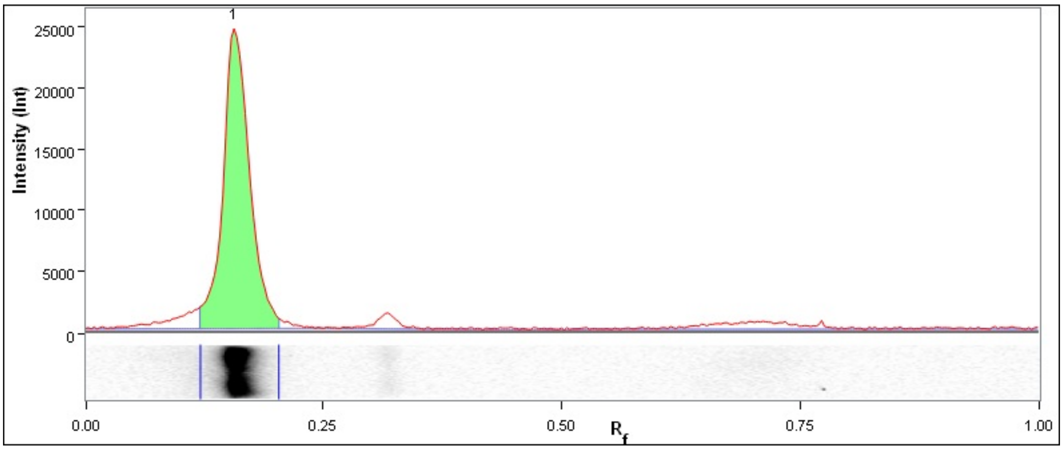

Band No.	Band Label	Mol. Wt. (KDa)	Relative Front	Adj. Volume (Int)	Volume (Int)	Abs. Quant.	Rel. Quant.	Band %	Lane %
1		N/A	0,158	11 550 378	11 885 538	N/A	N/A	100,0	78,4

Band Detection	Automatically detected bands with sensitivity: Low
Lane Background	Lane background subtracted with disk size: 10
Lane Width	6.76 mm

Lane 3

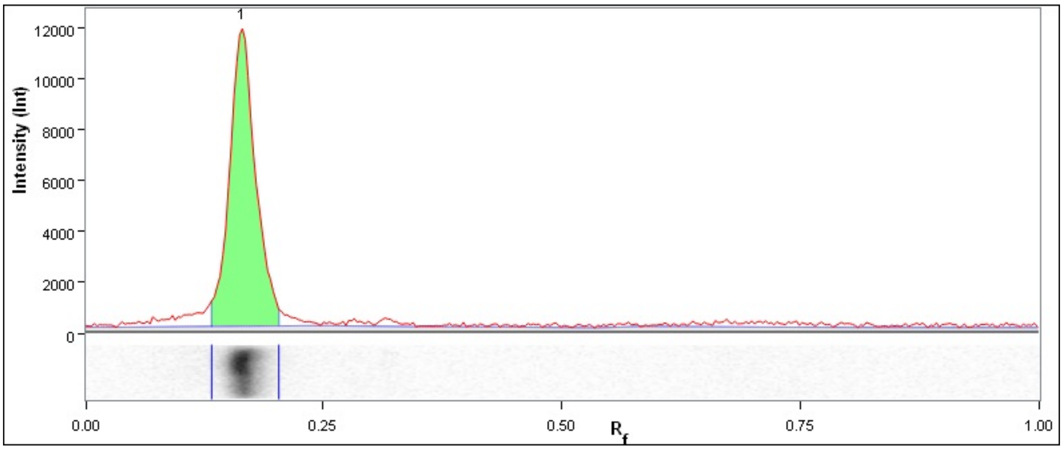

Band No.	Band Label	Mol. Wt. (KDa)	Relative Front	Adj. Volume (Int)	Volume (Int)	Abs. Quant.	Rel. Quant.	Band %	Lane %
1		N/A	0,167	5 852 970	6 107 895	N/A	N/A	100,0	74,6

Band Detection	Automatically detected bands with sensitivity: Low
Lane Background	Lane background subtracted with disk size: 10
Lane Width	7.24 mm

Lane 4

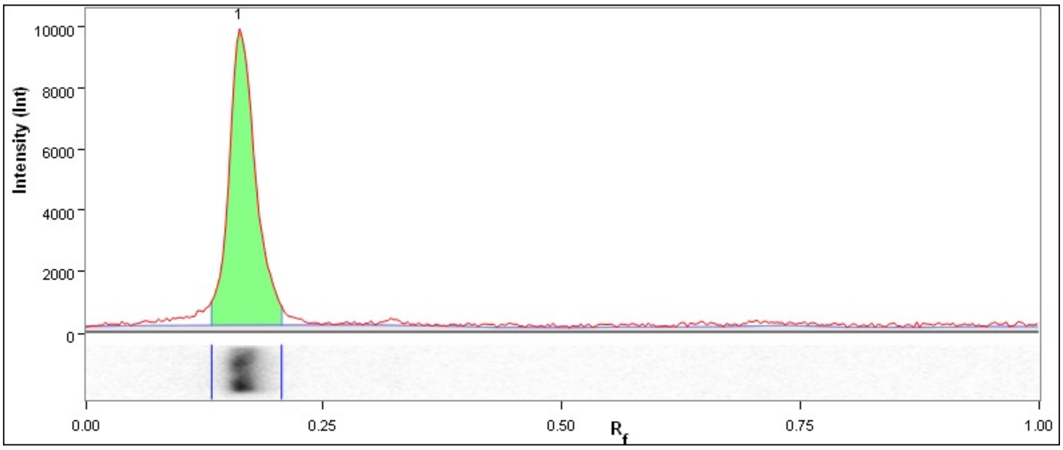

Band No.	Band Label	Mol. Wt. (KDa)	Relative Front	Adj. Volume (Int)	Volume (Int)	Abs. Quant.	Rel. Quant.	Band %	Lane %
1		N/A	0,164	5 959 152	6 263 664	N/A	N/A	100,0	78,7

Band Detection	Automatically detected bands with sensitivity: Low
Lane Background	Lane background subtracted with disk size: 10
Lane Width	7.72 mm

Lane 5

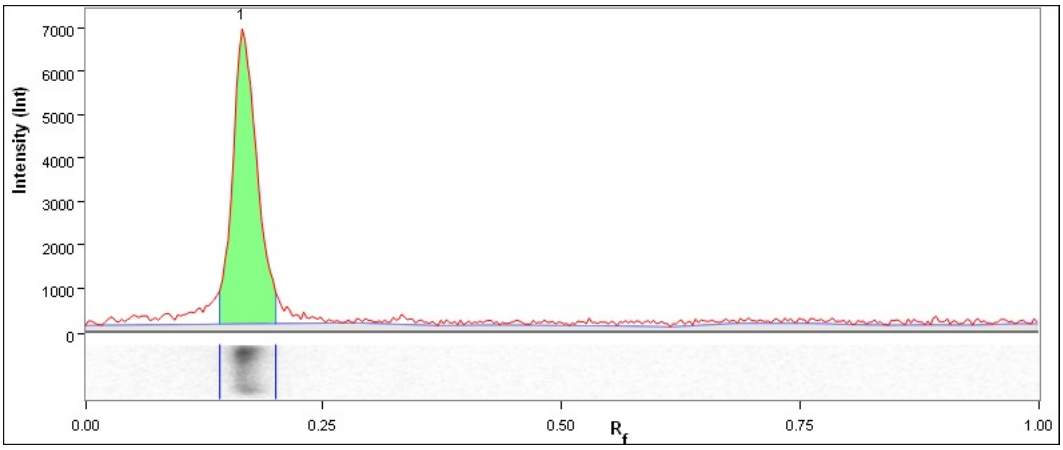

Band No.	Band Label	Mol. Wt. (KDa)	Relative Front	Adj. Volume (Int)	Volume (Int)	Abs. Quant.	Rel. Quant.	Band %	Lane %
1		N/A	0,167	3 313 086	3 495 030	N/A	N/A	100,0	68,3

Band Detection	Automatically detected bands with sensitivity: Low
Lane Background	Lane background subtracted with disk size: 10
Lane Width	6.76 mm

Lane 6

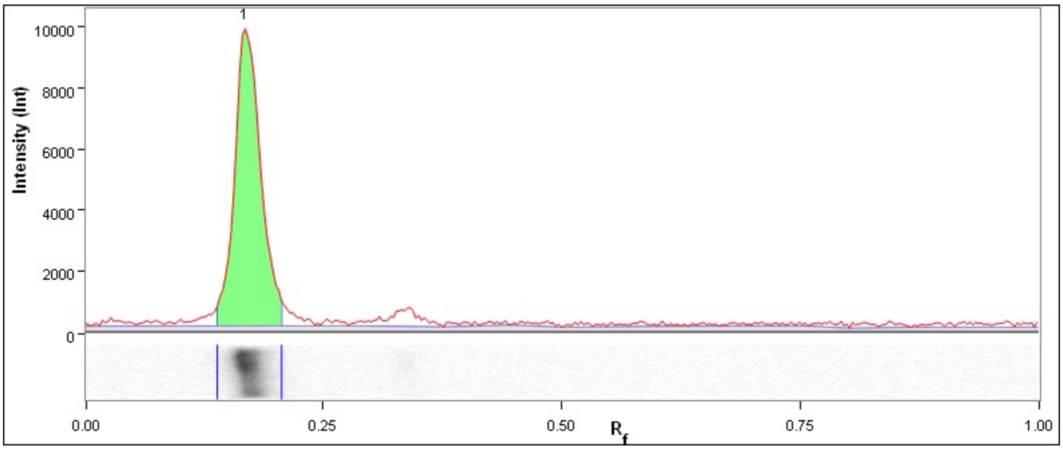

Band No.	Band Label	Mol. Wt. (KDa)	Relative Front	Adj. Volume (Int)	Volume (Int)	Abs. Quant.	Rel. Quant.	Band %	Lane %
1		N/A	0,170	4 970 024	5 187 558	N/A	N/A	100,0	72,3

Band Detection	Automatically detected bands with sensitivity: Low
Lane Background	Lane background subtracted with disk size: 10
Lane Width	7.40 mm

Lane 7

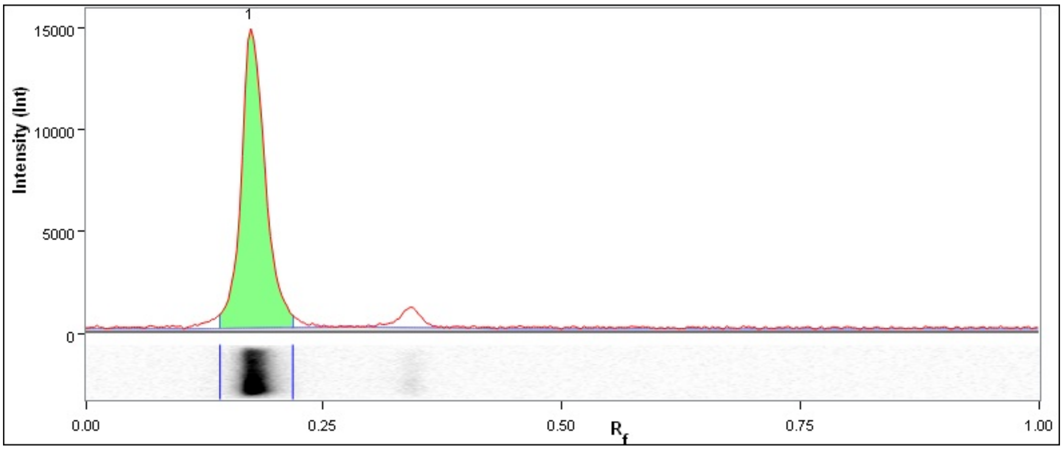

Band No.	Band Label	Mol. Wt. (KDa)	Relative Front	Adj. Volume (Int)	Volume (Int)	Abs. Quant.	Rel. Quant.	Band %	Lane %
1		N/A	0,176	9 423 936	9 739 632	N/A	N/A	100,0	81,9

Band Detection	Automatically detected bands with sensitivity: Low
Lane Background	Lane background subtracted with disk size: 10
Lane Width	7.72 mm

Lane 8

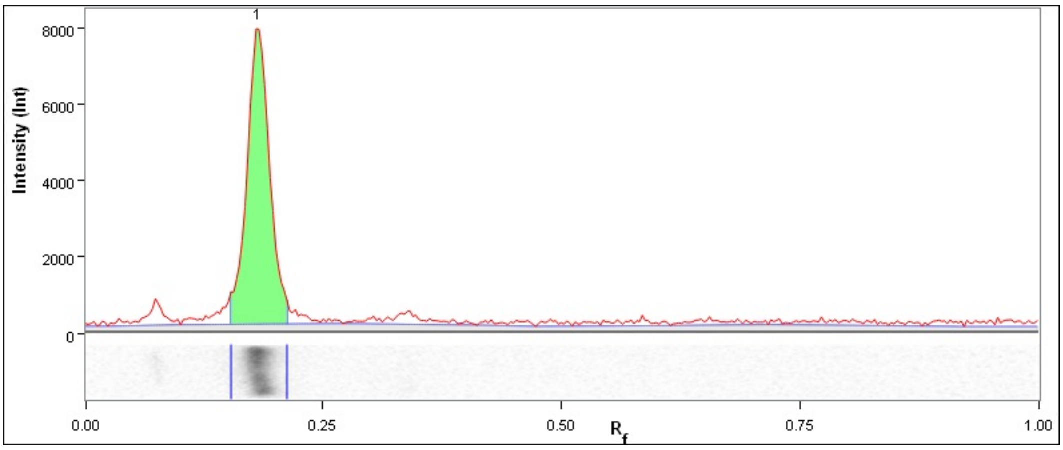

Band No.	Band Label	Mol. Wt. (KDa)	Relative Front	Adj. Volume (Int)	Volume (Int)	Abs. Quant.	Rel. Quant.	Band %	Lane %
1		N/A	0,185	3 898 244	4 117 862	N/A	N/A	100,0	65,7

Band Detection	Automatically detected bands with sensitivity: Low
Lane Background	Lane background subtracted with disk size: 10
Lane Width	7.89 mm

Volume Analysis

No.	Label	Type	Volume (Int)	Adj. Vol. (Int)	Mean Bkgd. (Int)	Abs. Quant.	Rel. Quant.	# of Pixels	Min. Value (Int)	Max. Value (Int)	Mean Value (Int)	Std. Dev.	Area (mm2)
1	U1	Unknown	4 652 340	2 932 878	1 142,5	N/A	N/A	1 505	0	13 916	3 091,3	3 253,2	39,0
2	U2	Unknown	12 295 148	9 546 341	1 826,5	N/A	N/A	1 505	0	35 484	8 169,5	8 493,0	39,0
3	U3	Unknown	6 290 992	4 705 286	1 053,6	N/A	N/A	1 505	0	17 344	4 180,1	4 283,7	39,0

4	U4	Unknown	6 357 244	4 915 981	957,6	N/A	N/A	1 505	0	18 296	4 224,1	4 277,1	39,0
5	U5	Unknown	3 933 944	2 804 592	750,4	N/A	N/A	1 505	0	13 748	2 613,9	2 804,8	39,0
6	U6	Unknown	5 342 532	3 985 624	901,6	N/A	N/A	1 505	0	15 040	3 549,9	3 739,9	39,0
7	U7	Unknown	9 748 224	7 898 165	1 229,3	N/A	N/A	1 505	0	33 636	6 477,2	7 320,8	39,0
8	U8	Unknown	4 181 664	2 615 523	1 040,6	N/A	N/A	1 505	0	12 632	2 778,5	3 040,1	39,0

Image Report: Histologia 2021-09-22 15hr 42min_Exposure_120.0sec stat3 2a

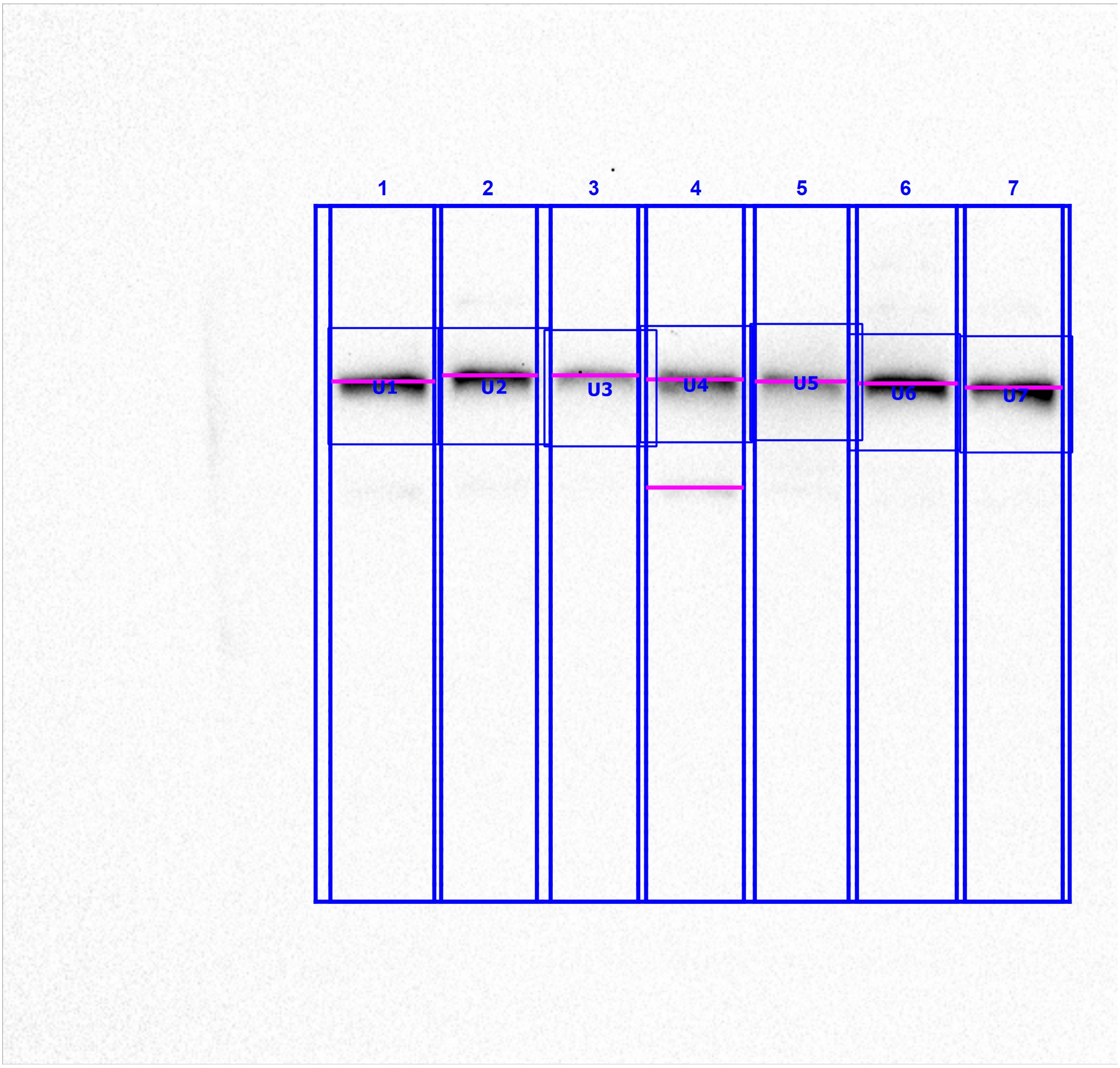

C:\Users\rusak\OneDrive\Dokumenty\Badania\CHI3L2 in BC\BC westerny ilościowo\stat3 2 BC\stat3 2\Histologia 2021-09-22 15hr 42min_Exposure_120.0sec stat3 2a.scn

Acquisition Information

Imager	ChemiDoc MP
Exposure Time (sec)	119.993 (Signal Accumulation)
Flat Field	Applied (Lens)

Serial Number	731BR01769
Software Version	5.0
Application	Chemi Hi Resolution
Excitation Source	No Illumination
Emission Filter	No Filter
Binning	2x2

Image Information

Acquisition Date	22/9/2021 3:44:28 PM
User Name	Histologia
Image Area (mm)	X: 112.0 Y: 83.7
Pixel Size (µm)	X: 160.9 Y: 160.9
Data Range (Int)	0 - 49880

Analysis Settings

Detection	Lane detection:<br>Manually created lanes<br><br>Band detection:<br>Automatically detected bands with sensitivity: Low<br><br>Lane Background Subtraction:<br>Lane background subtracted with disk size: 10<br><br>Lane width: Variable
Volume Analysis	Background subtraction method: Local<br>Quantity regression method: Linear

Lane Statistics

Lane No.	Adj. Total Band Vol. (Int)	Total Band Vol. (Int)	Adj. Total Lane Vol. (Int)	Total Lane Vol. (Int)	Bkgd. Vol. (Int)	Norm. Factor
1	10 853 463	11 228 313	13 039 731	17 467 143	4 427 412	N/A
2	9 961 932	10 347 896	11 995 011	15 827 908	3 832 897	N/A
3	4 280 177	4 504 938	6 190 925	9 415 151	3 224 226	N/A
4	9 627 984	10 320 336	11 482 896	15 764 112	4 281 216	N/A
5	6 913 754	7 179 312	10 684 374	14 263 864	3 579 490	N/A
6	13 699 469	14 333 088	16 562 098	21 602 728	5 040 630	N/A
7	12 273 024	12 833 616	14 262 144	18 931 536	4 669 392	N/A

Lane And Band Analysis

Lane 1

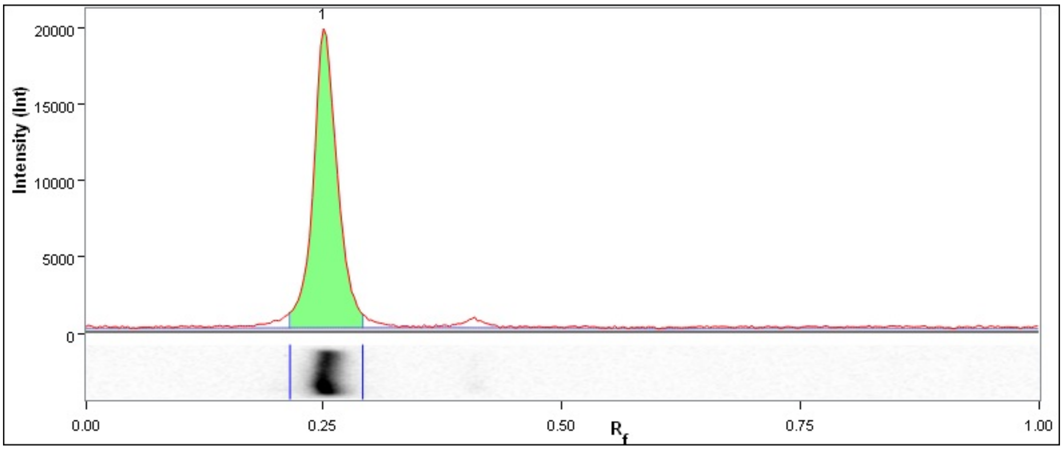

Band No.	Band Label	Mol. Wt. (KDa)	Relative Front	Adj. Volume (Int)	Volume (Int)	Abs. Quant.	Rel. Quant.	Band %	Lane %
1		N/A	0,252	10 853 463	11 228 313	N/A	N/A	100,0	83,2

Band Detection	Automatically detected bands with sensitivity: Low
Lane Background	Lane background subtracted with disk size: 10
Lane Width	8.21 mm

Lane 2

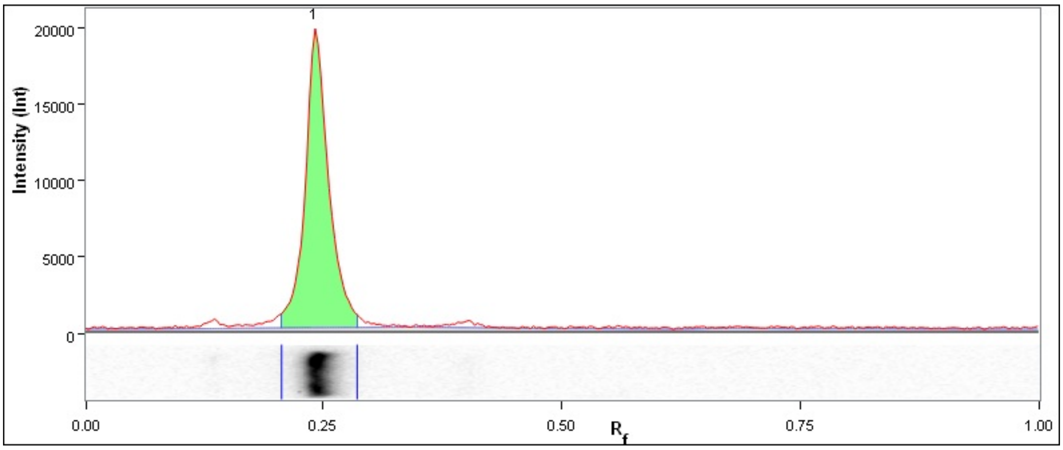

Band No.	Band Label	Mol. Wt. (KDa)	Relative Front	Adj. Volume (Int)	Volume (Int)	Abs. Quant.	Rel. Quant.	Band %	Lane %
1		N/A	0,243	9 961 932	10 347 896	N/A	N/A	100,0	83,1

Band Detection	Automatically detected bands with sensitivity: Low
Lane Background	Lane background subtracted with disk size: 10
Lane Width	7.56 mm

Lane 3

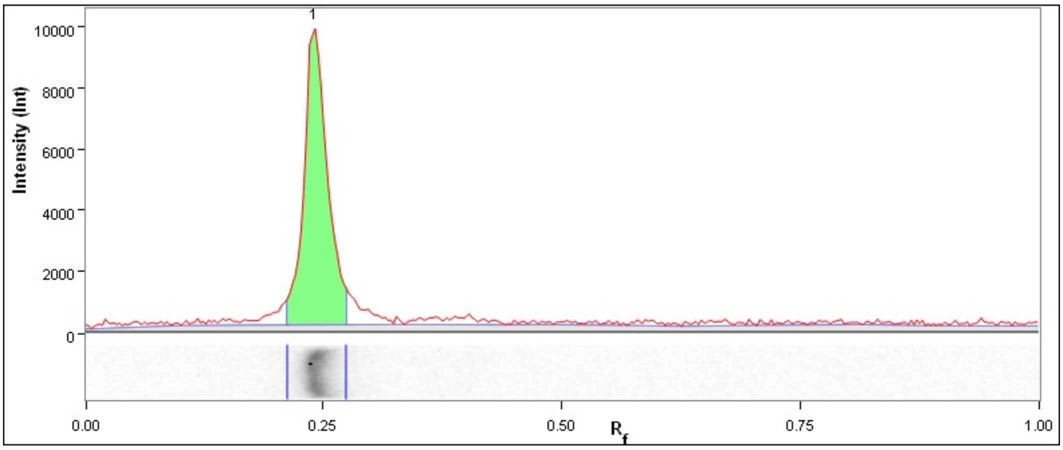

Band No.	Band Label	Mol. Wt. (KDa)	Relative Front	Adj. Volume (Int)	Volume (Int)	Abs. Quant.	Rel. Quant.	Band %	Lane %
1		N/A	0,243	4 280 177	4 504 938	N/A	N/A	100,0	69,1

Band Detection	Automatically detected bands with sensitivity: Low
Lane Background	Lane background subtracted with disk size: 10
Lane Width	6.92 mm

Lane 4

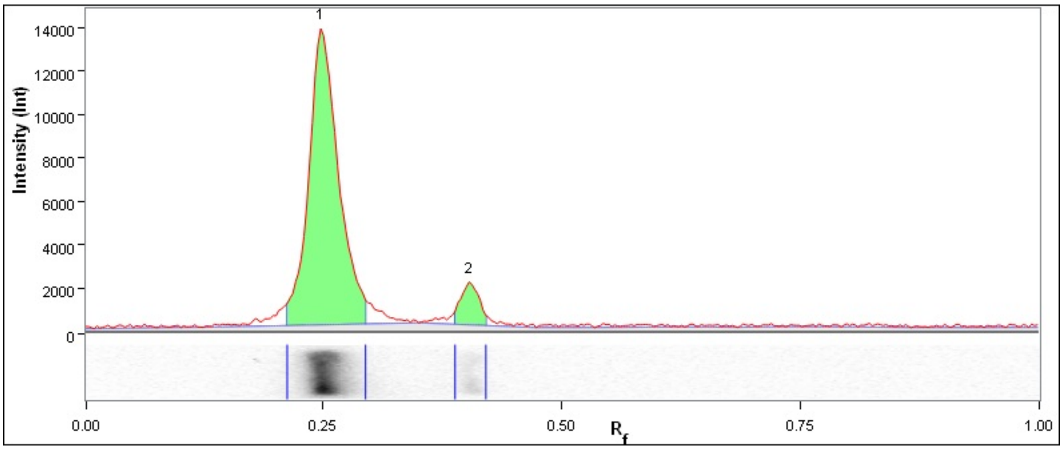

Band No.	Band Label	Mol. Wt. (KDa)	Relative Front	Adj. Volume (Int)	Volume (Int)	Abs. Quant.	Rel. Quant.	Band %	Lane %
1		N/A	0,249	8 832 288	9 325 248	N/A	N/A	91,7	76,9
2		N/A	0,405	795 696	995 088	N/A	N/A	8,3	6,9

Band Detection	Automatically detected bands with sensitivity: Low
Lane Background	Lane background subtracted with disk size: 10
Lane Width	7.72 mm

Lane 5

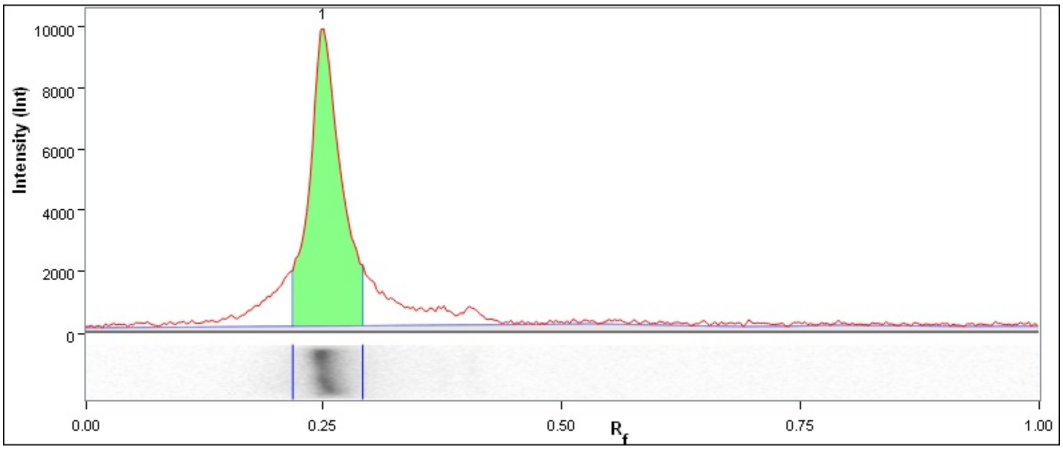

Band No.	Band Label	Mol. Wt. (KDa)	Relative Front	Adj. Volume (Int)	Volume (Int)	Abs. Quant.	Rel. Quant.	Band %	Lane %
1		N/A	0,252	6 913 754	7 179 312	N/A	N/A	100,0	64,7

Band Detection	Automatically detected bands with sensitivity: Low
Lane Background	Lane background subtracted with disk size: 10
Lane Width	7.40 mm

Lane 6

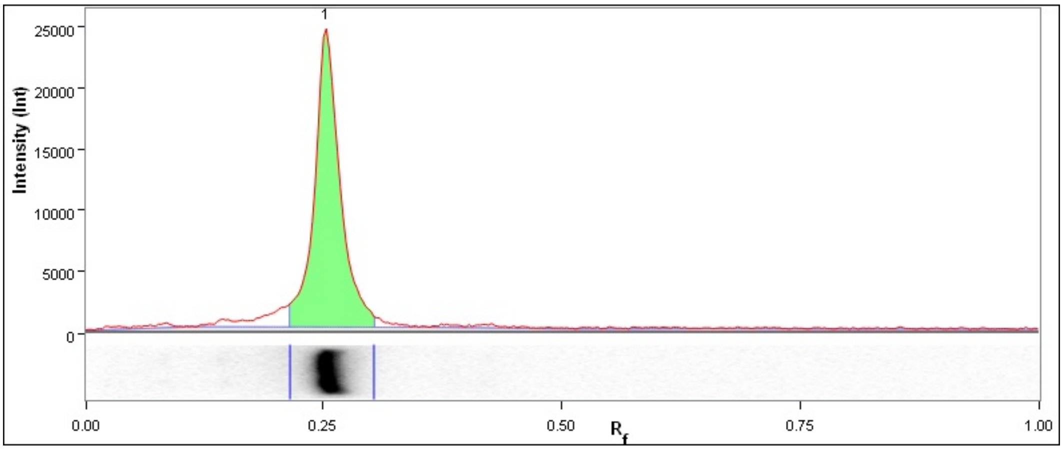

Band No.	Band Label	Mol. Wt. (KDa)	Relative Front	Adj. Volume (Int)	Volume (Int)	Abs. Quant.	Rel. Quant.	Band %	Lane %
1		N/A	0,255	13 699 469	14 333 088	N/A	N/A	100,0	82,7

Band Detection	Automatically detected bands with sensitivity: Low
Lane Background	Lane background subtracted with disk size: 10
Lane Width	7.89 mm

Lane 7

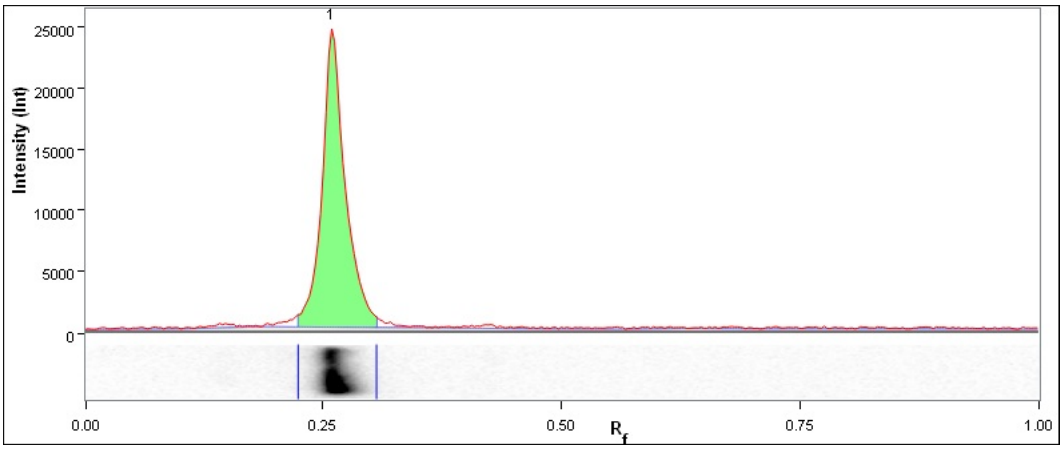

Band No.	Band Label	Mol. Wt. (KDa)	Relative Front	Adj. Volume (Int)	Volume (Int)	Abs. Quant.	Rel. Quant.	Band %	Lane %
1		N/A	0,261	12 273 024	12 833 616	N/A	N/A	100,0	86,1

Band Detection	Automatically detected bands with sensitivity: Low
Lane Background	Lane background subtracted with disk size: 10
Lane Width	7.72 mm

Volume Analysis

No.	Label	Type	Volume (Int)	Adj. Vol. (Int)	Mean Bkgd. (Int)	Abs. Quant.	Rel. Quant.	# of Pixels	Min. Value (Int)	Max. Value (Int)	Mean Value (Int)	Std. Dev.	Area (mm2)
1	U1	Unknown	12 442 128	10 577 408	594,8	N/A	N/A	3 135	0	32 696	3 968,8	6 475,1	81,2
2	U2	Unknown	11 584 444	9 439 389	684,2	N/A	N/A	3 135	0	33 004	3 695,2	6 178,3	81,2
3	U3	Unknown	6 112 812	3 089 682	964,3	N/A	N/A	3 135	0	48 088	1 949,9	2 848,0	81,2
4	U4	Unknown	10 935 832	8 087 492	908,6	N/A	N/A	3 135	0	23 312	3 488,3	4 664,7	81,2
5	U5	Unknown	10 367 180	5 191 680	1 650,9	N/A	N/A	3 135	0	16 312	3 306,9	3 151,8	81,2
6	U6	Unknown	16 447 416	12 743 771	1 181,4	N/A	N/A	3 135	0	37 648	5 246,4	7 663,3	81,2
7	U7	Unknown	14 189 332	12 101 972	665,8	N/A	N/A	3 135	0	49 880	4 526,1	7 804,0	81,2

Image Report: Histologia 2021-09-22 15hr 42min_Exposure_120.0sec stat3 2c

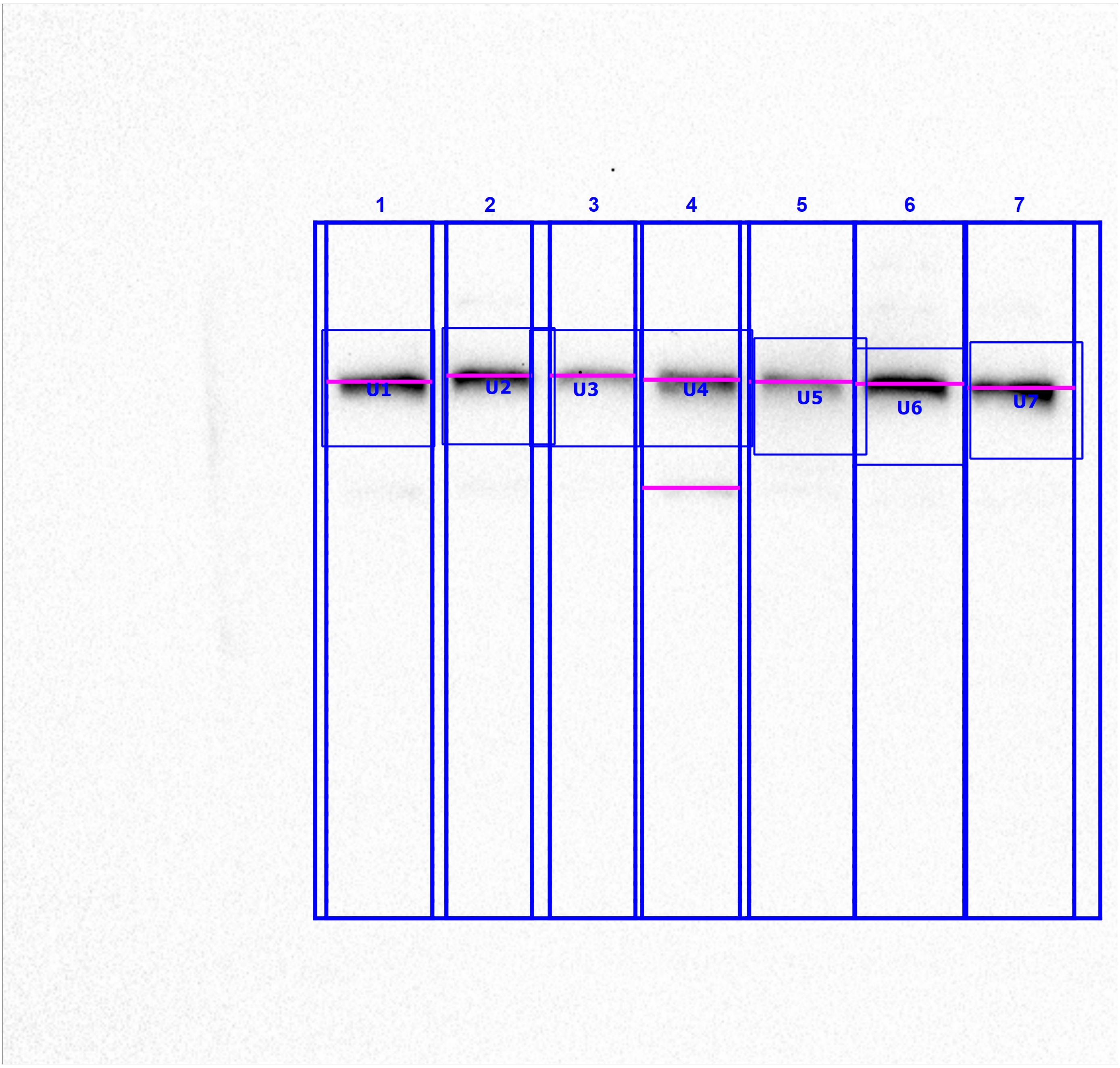

C:\Users\rusak\OneDrive\Dokumenty\Badania\CHI3L2 in BC\BC westerny ilościowo\analiza STAT3\stat3 2\Histologia 2021-09-22 15hr 42min_Exposure_120.0sec stat3 2c.scn

Acquisition Information

Imager	ChemiDoc MP
Exposure Time (sec)	119.993 (Signal Accumulation)
Flat Field	Applied (Lens)

Serial Number	731BR01769
Software Version	5.0
Application	Chemi Hi Resolution
Excitation Source	No Illumination
Emission Filter	No Filter
Binning	2x2

Image Information

Acquisition Date	22/9/2021 3:44:28 PM
User Name	Histologia
Image Area (mm)	X: 112.0 Y: 83.7
Pixel Size (μm)	X: 160.9 Y: 160.9
Data Range (Int)	0 - 49880

Analysis Settings

Detection	Lane detection:<br>Manually created lanes<br><br>Band detection:<br>Automatically detected bands with sensitivity: Low<br><br>Lane Background Subtraction:<br>Lane background subtracted with disk size: 10<br><br>Lane width: Variable
Volume Analysis	Background subtraction method: Local<br>Quantity regression method: Linear

Lane Statistics

Lane No.	Adj. Total Band Vol. (Int)	Total Band Vol. (Int)	Adj. Total Lane Vol. (Int)	Total Lane Vol. (Int)	Bkgd. Vol. (Int)	Norm. Factor
1	10 803 676	11 226 748	12 894 336	17 579 328	4 684 992	N/A
2	9 718 464	10 090 038	11 587 842	15 014 748	3 426 906	N/A
3	4 236 162	4 458 720	6 039 222	9 287 082	3 247 860	N/A
4	9 553 776	10 206 576	11 431 968	15 621 024	4 189 056	N/A
5	7 258 316	7 611 916	11 105 692	15 561 416	4 455 724	N/A
6	13 943 556	14 627 898	16 816 248	22 511 520	5 695 272	N/A
7	12 419 596	13 006 465	14 608 231	19 672 169	5 063 938	N/A

Lane And Band Analysis

Lane 1

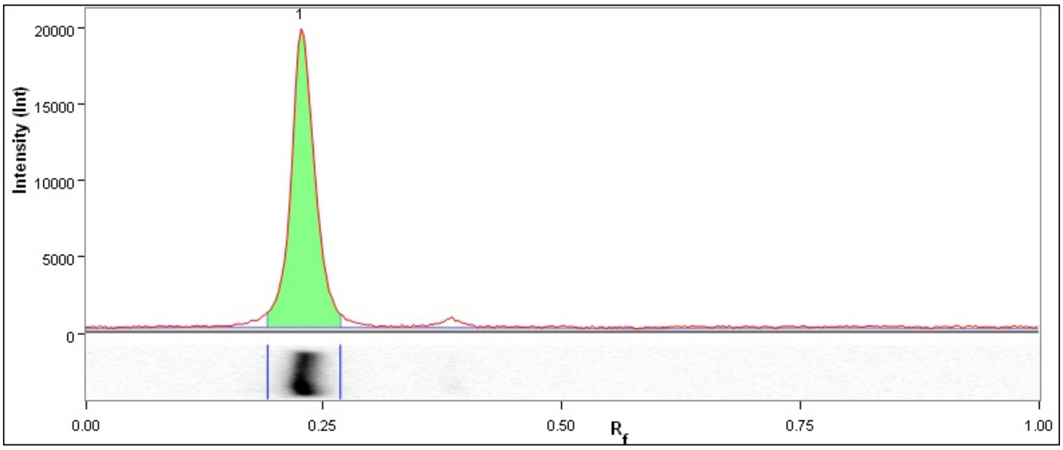

Band No.	Band Label	Mol. Wt. (KDa)	Relative Front	Adj. Volume (Int)	Volume (Int)	Abs. Quant.	Rel. Quant.	Band %	Lane %
1		N/A	0,229	10 803 676	11 226 748	N/A	N/A	100,0	83,8

Band Detection	Automatically detected bands with sensitivity: Low
Lane Background	Lane background subtracted with disk size: 10
Lane Width	8.37 mm

Lane 2

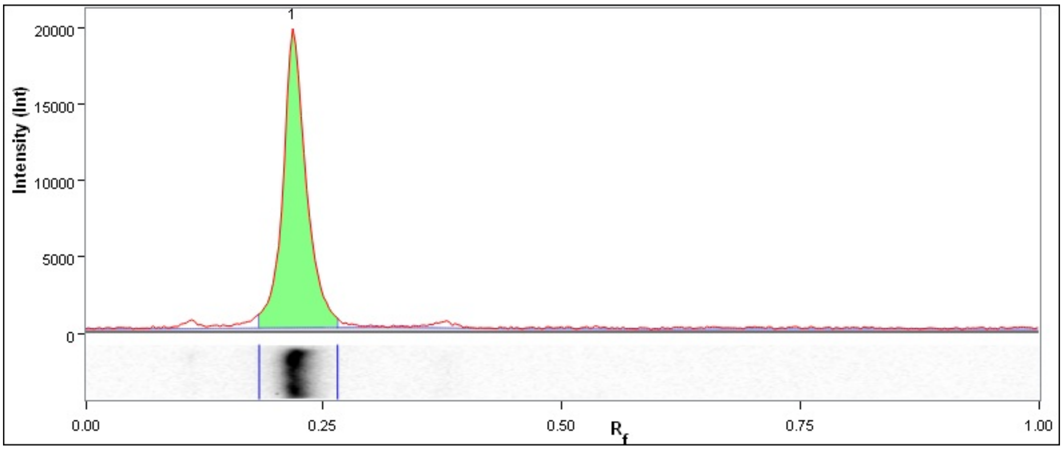

Band No.	Band Label	Mol. Wt. (KDa)	Relative Front	Adj. Volume (Int)	Volume (Int)	Abs. Quant.	Rel. Quant.	Band %	Lane %
1		N/A	0,220	9 718 464	10 090 038	N/A	N/A	100,0	83,9

Band Detection	Automatically detected bands with sensitivity: Low
Lane Background	Lane background subtracted with disk size: 10
Lane Width	6.76 mm

Lane 3

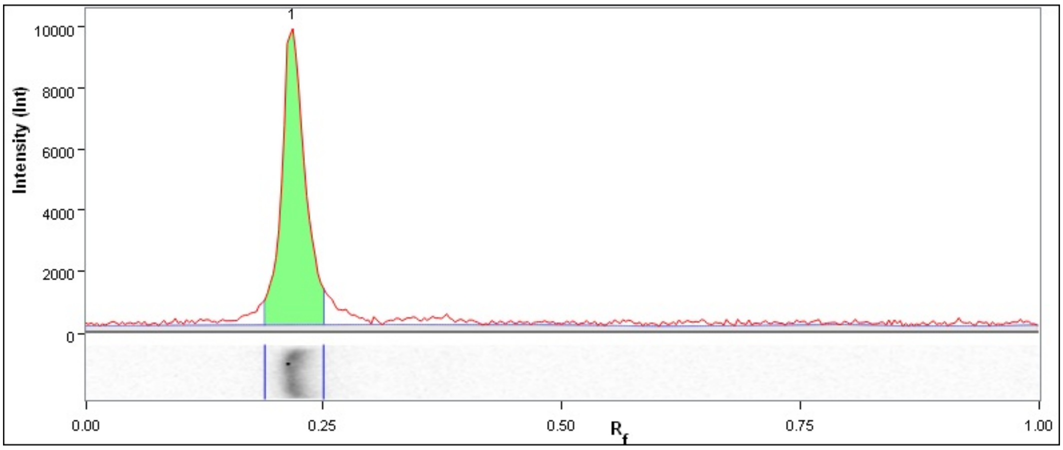

Band No.	Band Label	Mol. Wt. (KDa)	Relative Front	Adj. Volume (Int)	Volume (Int)	Abs. Quant.	Rel. Quant.	Band %	Lane %
1		N/A	0,220	4 236 162	4 458 720	N/A	N/A	100,0	70,1

Band Detection	Automatically detected bands with sensitivity: Low
Lane Background	Lane background subtracted with disk size: 10
Lane Width	6.76 mm

Lane 4

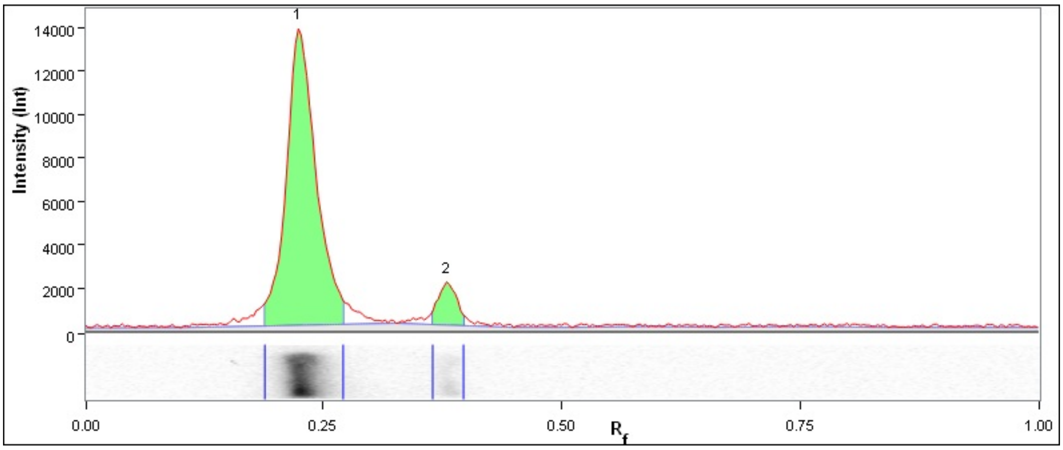

Band No.	Band Label	Mol. Wt. (KDa)	Relative Front	Adj. Volume (Int)	Volume (Int)	Abs. Quant.	Rel. Quant.	Band %	Lane %
1		N/A	0,226	8 759 232	9 219 744	N/A	N/A	91,7	76,6
2		N/A	0,381	794 544	986 832	N/A	N/A	8,3	7,0

Band Detection	Automatically detected bands with sensitivity: Low
Lane Background	Lane background subtracted with disk size: 10
Lane Width	7.72 mm

Lane 5

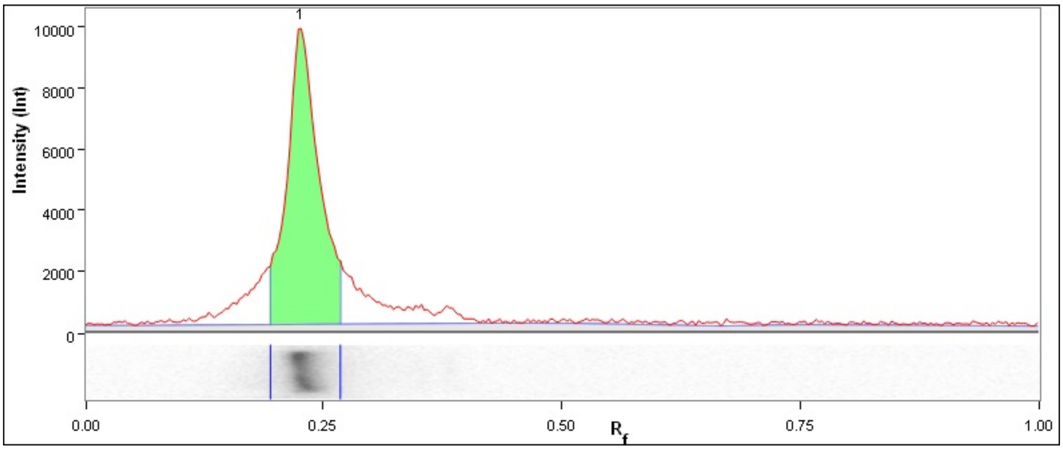

Band No.	Band Label	Mol. Wt. (KDa)	Relative Front	Adj. Volume (Int)	Volume (Int)	Abs. Quant.	Rel. Quant.	Band %	Lane %
1		N/A	0,229	7 258 316	7 611 916	N/A	N/A	100,0	65,4

Band Detection	Automatically detected bands with sensitivity: Low
Lane Background	Lane background subtracted with disk size: 10
Lane Width	8.37 mm

Lane 6

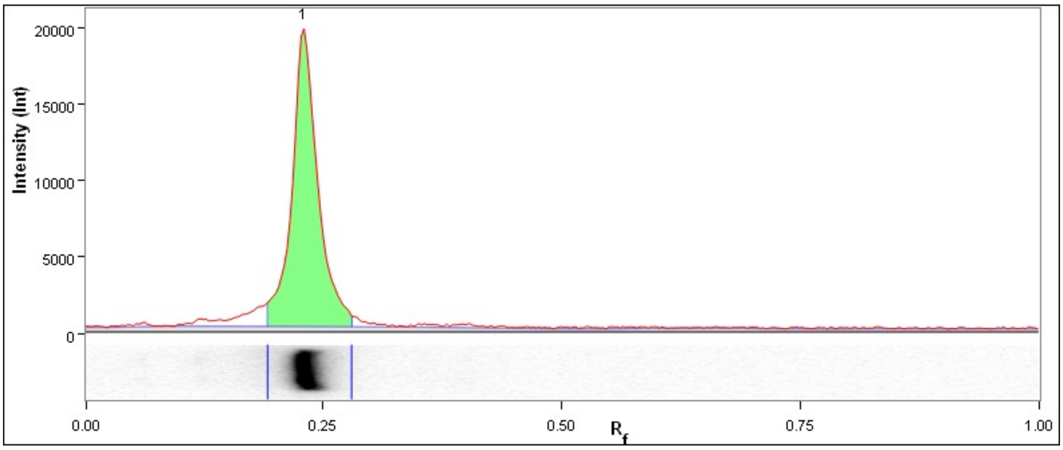

Band No.	Band Label	Mol. Wt. (KDa)	Relative Front	Adj. Volume (Int)	Volume (Int)	Abs. Quant.	Rel. Quant.	Band %	Lane %
1		N/A	0,232	13 943 556	14 627 898	N/A	N/A	100,0	82,9

Band Detection	Automatically detected bands with sensitivity: Low
Lane Background	Lane background subtracted with disk size: 10
Lane Width	8.69 mm

Lane 7

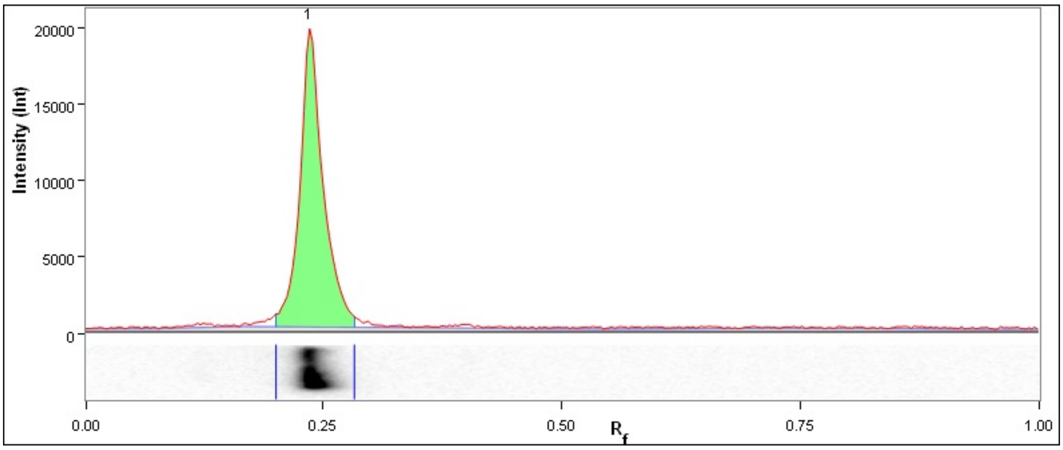

Band No.	Band Label	Mol. Wt. (KDa)	Relative Front	Adj. Volume (Int)	Volume (Int)	Abs. Quant.	Rel. Quant.	Band %	Lane %
1		N/A	0,238	12 419 596	13 006 465	N/A	N/A	100,0	85,0

Band Detection	Automatically detected bands with sensitivity: Low
Lane Background	Lane background subtracted with disk size: 10
Lane Width	8.53 mm

Volume Analysis

No.	Label	Type	Volume (Int)	Adj. Vol. (Int)	Mean Bkgd. (Int)	Abs. Quant.	Rel. Quant.	# of Pixels	Min. Value (Int)	Max. Value (Int)	Mean Value (Int)	Std. Dev.	Area (mm2)
1	U1	Unknown	12 322 724	10 314 729	640,5	N/A	N/A	3 135	0	32 696	3 930,7	6 491,2	81,2
2	U2	Unknown	11 564 196	8 952 631	833,0	N/A	N/A	3 135	0	33 004	3 688,7	6 181,1	81,2
3	U3	Unknown	6 174 348	2 206 593	1 265,6	N/A	N/A	3 135	0	48 088	1 969,5	2 885,9	81,2
4	U4	Unknown	10 952 192	8 102 917	908,9	N/A	N/A	3 135	0	23 312	3 493,5	4 661,5	81,2
5	U5	Unknown	10 595 464	3 766 554	2 178,3	N/A	N/A	3 135	0	17 160	3 379,7	3 218,9	81,2
6	U6	Unknown	15 906 448	11 377 913	1 444,5	N/A	N/A	3 135	0	37 648	5 073,8	7 748,5	81,2
7	U7	Unknown	13 904 320	10 968 035	936,6	N/A	N/A	3 135	0	49 880	4 435,2	7 830,4	81,2

Image Report: Histologia 2023-01-24 13hr 48min_Exposure_60.0sec stat3 1b

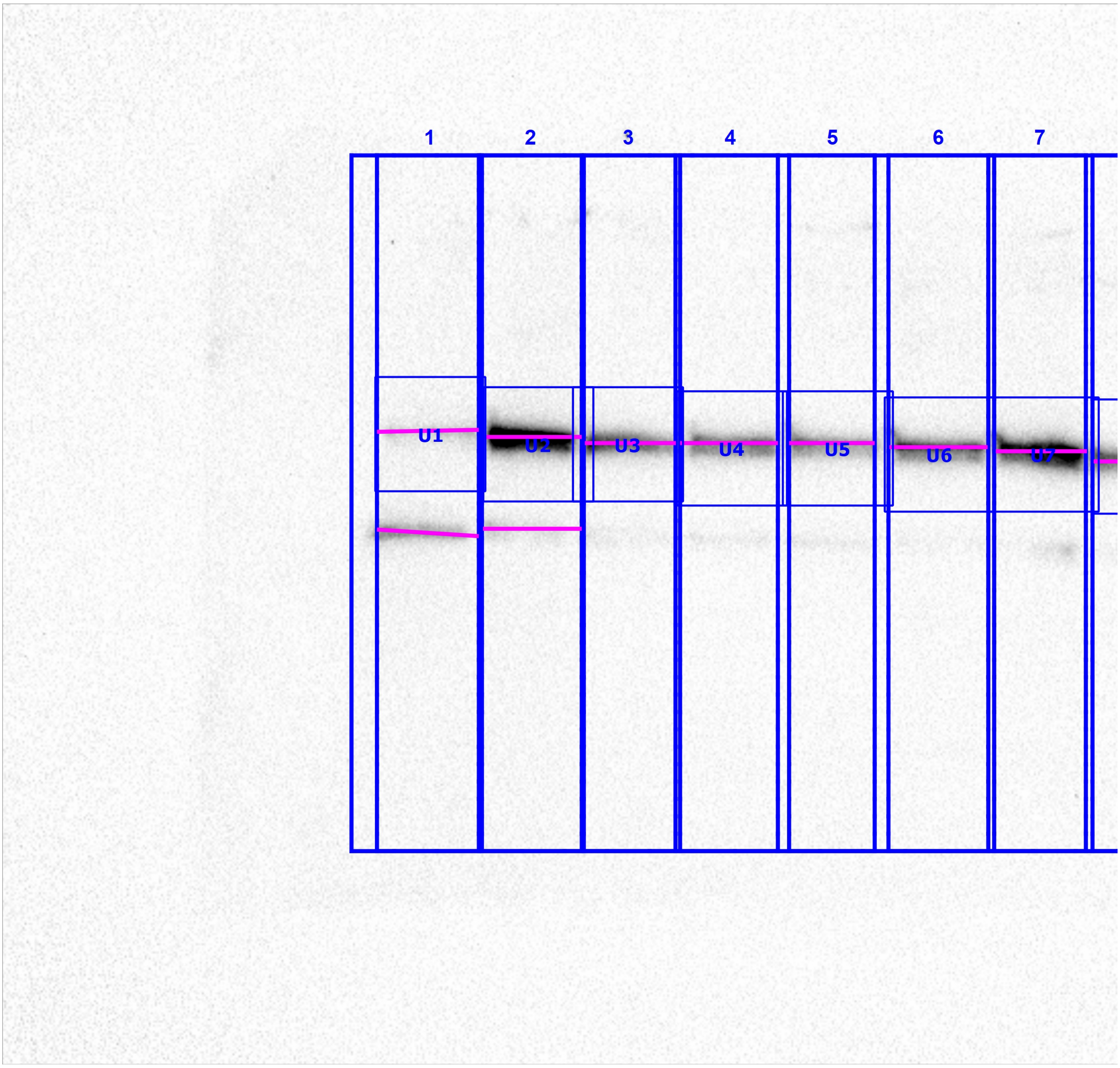

C:\Users\rusak\OneDrive\Dokumenty\Badania\CHI3L2 in BC\BC westerny ilościowo\STAT3 24.1.23 BC\1\Histologia 2023-01-24 13hr 48min_Exposure_60.0sec stat3 1b.scn

Acquisition Information

Imager	ChemiDoc MP
Exposure Time (sec)	60.000 (Signal Accumulation)
Flat Field	Applied (Lens)

Serial Number	731BR01769
Software Version	5.0
Application	Chemi Hi Resolution
Excitation Source	No Illumination
Emission Filter	No Filter
Binning	2x2

Image Information

Acquisition Date	24/1/2023 1:49:48 PM
User Name	Histologia
Image Area (mm)	X: 114.0 Y: 85.2
Pixel Size (μm)	X: 163.8 Y: 163.8
Data Range (Int)	0 - 25624

Analysis Settings

Detection	Lane detection:<br>Manually created lanes<br><br>Band detection:<br>Automatically detected bands with sensitivity: Low<br><br>Lane Background Subtraction:<br>Lane background subtracted with disk size: 10<br><br>Lane width: Variable
Volume Analysis	Background subtraction method: Local<br>Quantity regression method: Linear

Lane Statistics

Lane No.	Adj. Total Band Vol. (Int)	Total Band Vol. (Int)	Adj. Total Lane Vol. (Int)	Total Lane Vol. (Int)	Bkgd. Vol. (Int)	Norm. Factor
1	2 582 350	3 214 200	4 003 000	9 587 950	5 584 950	N/A
2	8 834 406	9 559 067	10 560 137	15 566 810	5 006 673	N/A
3	3 648 780	3 950 955	5 156 145	8 809 965	3 653 820	N/A
4	3 299 568	3 577 008	4 736 448	8 003 664	3 267 216	N/A
5	2 447 214	2 662 968	3 918 348	6 843 984	2 925 636	N/A
6	4 233 012	4 553 570	5 715 458	9 434 215	3 718 757	N/A
7	6 961 005	7 330 005	8 689 455	12 164 310	3 474 855	N/A
8	3 703 271	4 002 332	5 122 953	8 501 642	3 378 689	N/A

Lane And Band Analysis

Lane 1

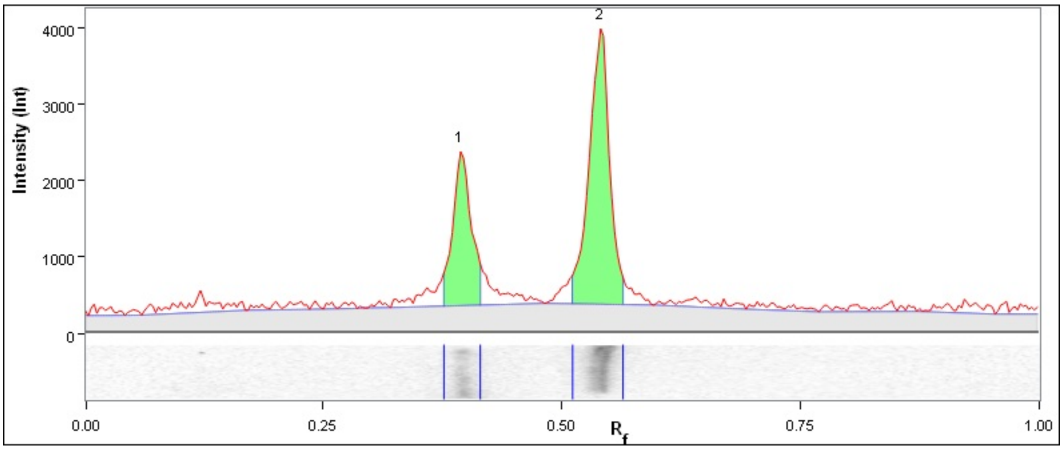

Band No.	Band Label	Mol. Wt. (KDa)	Relative Front	Adj. Volume (Int)	Volume (Int)	Abs. Quant.	Rel. Quant.	Band %	Lane %
1		N/A	0,396	877 650	1 145 900	N/A	N/A	34,0	21,9
2		N/A	0,543	1 704 700	2 068 300	N/A	N/A	66,0	42,6

Band Detection	Automatically detected bands with sensitivity: Low
Lane Background	Lane background subtracted with disk size: 10
Lane Width	8.19 mm

Lane 2

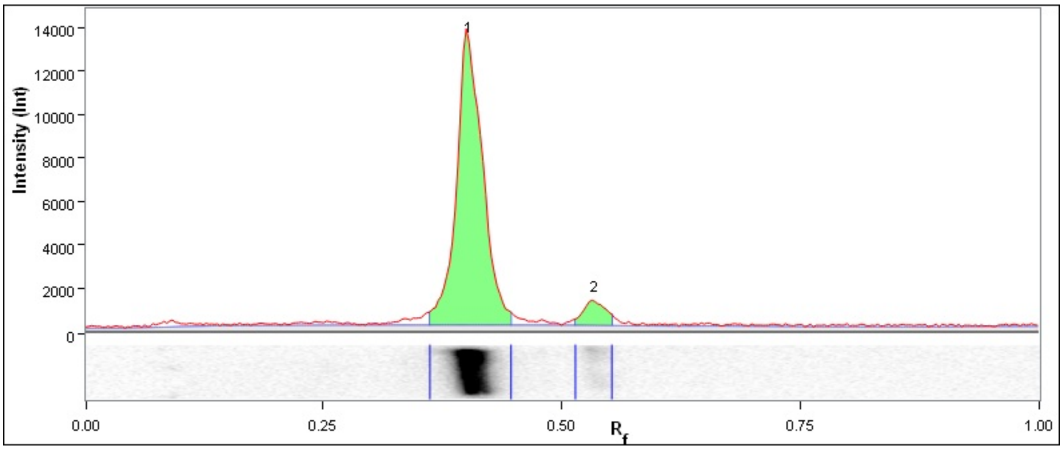

Band No.	Band Label	Mol. Wt. (KDa)	Relative Front	Adj. Volume (Int)	Volume (Int)	Abs. Quant.	Rel. Quant.	Band %	Lane %
1		N/A	0,405	8 248 219	8 760 416	N/A	N/A	93,4	78,1
2		N/A	0,537	586 187	798 651	N/A	N/A	6,6	5,6

Band Detection	Automatically detected bands with sensitivity: Low
Lane Background	Lane background subtracted with disk size: 10
Lane Width	8.03 mm

Lane 3

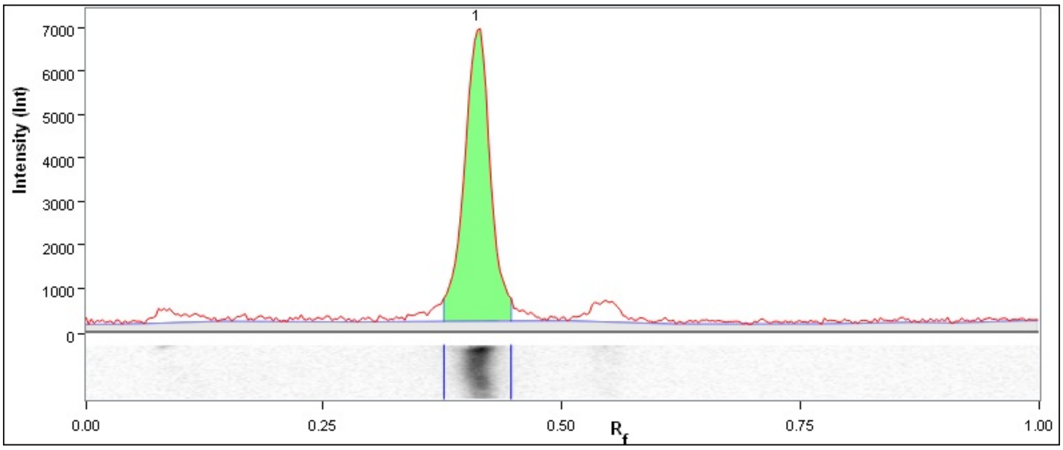

Band No.	Band Label	Mol. Wt. (KDa)	Relative Front	Adj. Volume (Int)	Volume (Int)	Abs. Quant.	Rel. Quant.	Band %	Lane %
1		N/A	0,413	3 648 780	3 950 955	N/A	N/A	100,0	70,8

Band Detection	Automatically detected bands with sensitivity: Low
Lane Background	Lane background subtracted with disk size: 10
Lane Width	7.37 mm

Lane 4

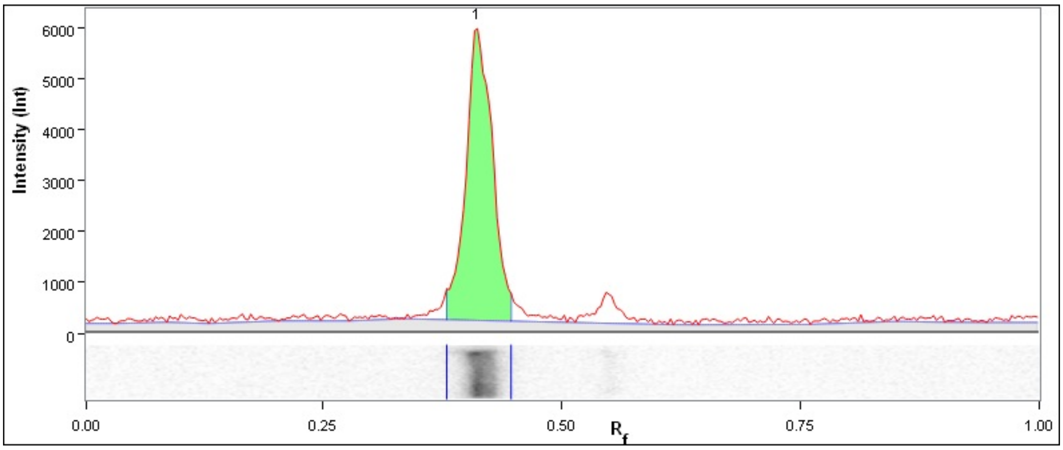

Band No.	Band Label	Mol. Wt. (KDa)	Relative Front	Adj. Volume (Int)	Volume (Int)	Abs. Quant.	Rel. Quant.	Band %	Lane %
1		N/A	0,413	3 299 568	3 577 008	N/A	N/A	100,0	69,7

Band Detection	Automatically detected bands with sensitivity: Low
Lane Background	Lane background subtracted with disk size: 10
Lane Width	7.86 mm

Lane 5

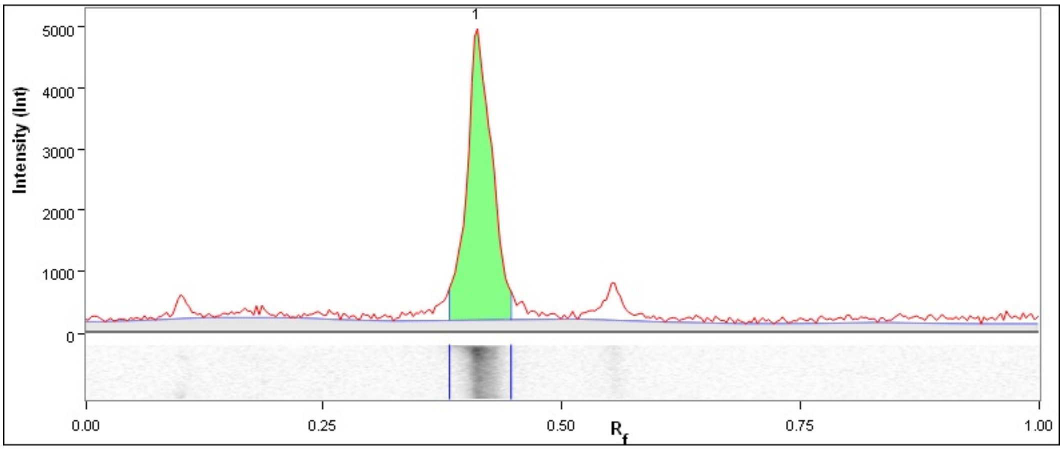

Band No.	Band Label	Mol. Wt. (KDa)	Relative Front	Adj. Volume (Int)	Volume (Int)	Abs. Quant.	Rel. Quant.	Band %	Lane %
1		N/A	0,413	2 447 214	2 662 968	N/A	N/A	100,0	62,5

Band Detection	Automatically detected bands with sensitivity: Low
Lane Background	Lane background subtracted with disk size: 10
Lane Width	6.88 mm

Lane 6

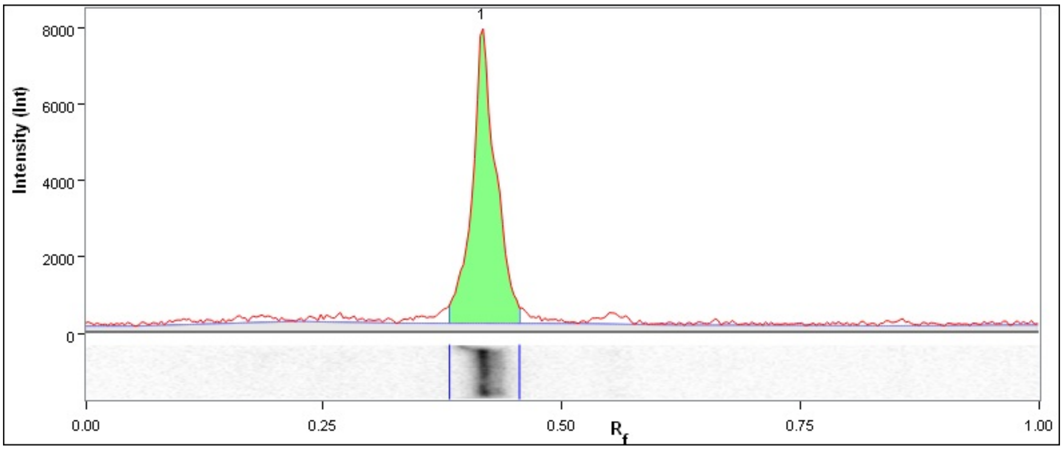

Band No.	Band Label	Mol. Wt. (KDa)	Relative Front	Adj. Volume (Int)	Volume (Int)	Abs. Quant.	Rel. Quant.	Band %	Lane %
1		N/A	0,419	4 233 012	4 553 570	N/A	N/A	100,0	74,1

Band Detection	Automatically detected bands with sensitivity: Low
Lane Background	Lane background subtracted with disk size: 10
Lane Width	8.03 mm

Lane 7

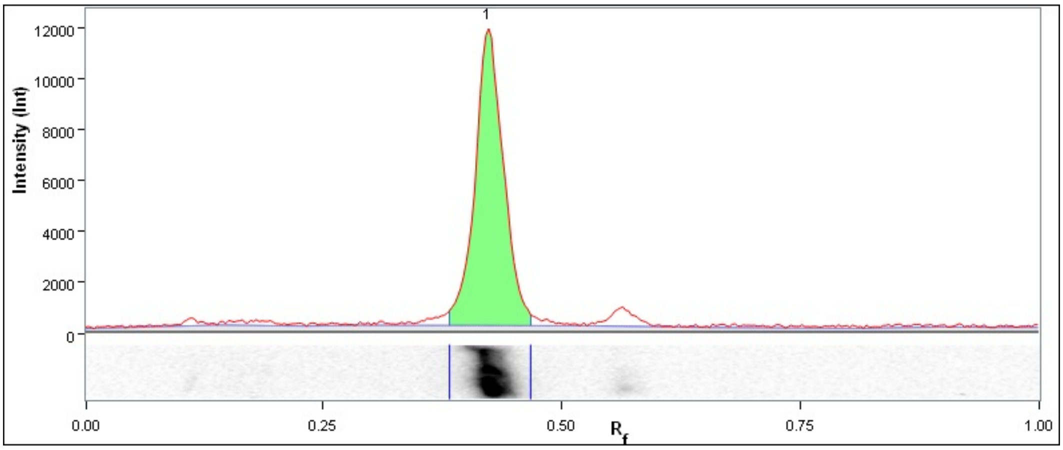

Band No.	Band Label	Mol. Wt. (KDa)	Relative Front	Adj. Volume (Int)	Volume (Int)	Abs. Quant.	Rel. Quant.	Band %	Lane %
1		N/A	0,425	6 961 005	7 330 005	N/A	N/A	100,0	80,1

Band Detection	Automatically detected bands with sensitivity: Low
Lane Background	Lane background subtracted with disk size: 10
Lane Width	7.37 mm

Lane 8

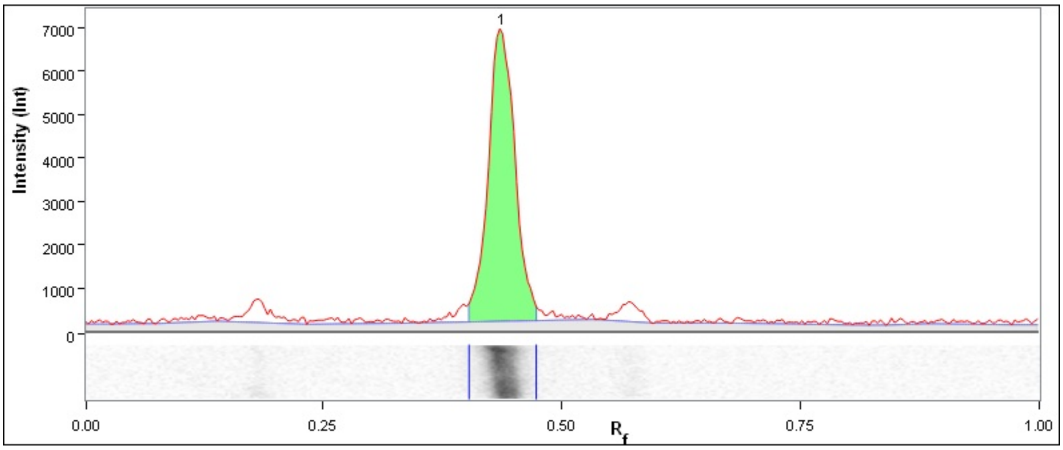

Band No.	Band Label	Mol. Wt. (KDa)	Relative Front	Adj. Volume (Int)	Volume (Int)	Abs. Quant.	Rel. Quant.	Band %	Lane %
1		N/A	0,440	3 703 271	4 002 332	N/A	N/A	100,0	72,3

Band Detection	Automatically detected bands with sensitivity: Low
Lane Background	Lane background subtracted with disk size: 10
Lane Width	7.70 mm

Volume Analysis

No.	Label	Type	Volume (Int)	Adj. Vol. (Int)	Mean Bkgd. (Int)	Abs. Quant.	Rel. Quant.	# of Pixels	Min. Value (Int)	Max. Value (Int)	Mean Value (Int)	Std. Dev.	Area (mm2)
1	U1	Unknown	2 501 796	213 978	756,6	N/A	N/A	3 024	0	4 688	827,3	731,7	81,1
2	U2	Unknown	10 228 640	6 859 040	1 114,3	N/A	N/A	3 024	0	25 624	3 382,5	4 840,8	81,1
3	U3	Unknown	5 142 084	2 330 304	929,8	N/A	N/A	3 024	0	13 468	1 700,4	2 226,8	81,1

4	U4	Unknown	4 486 192	2 421 610	682,7	N/A	N/A	3 024	0	8 780	1 483,5	1 897,8	81,1
5	U5	Unknown	3 945 024	1 374 948	849,9	N/A	N/A	3 024	0	9 048	1 304,6	1 568,6	81,1
6	U6	Unknown	5 576 808	2 885 124	890,1	N/A	N/A	3 024	0	12 696	1 844,2	2 409,3	81,1
7	U7	Unknown	8 748 568	5 764 204	986,9	N/A	N/A	3 024	0	22 696	2 893,0	4 041,4	81,1
8	U8	Unknown	4 802 484	3 068 328	573,5	N/A	N/A	3 024	0	9 484	1 588,1	2 171,3	81,1

Image Report: Histologia 2023-01-24 13hr 50min_Exposure_60.0sec stat3 2b

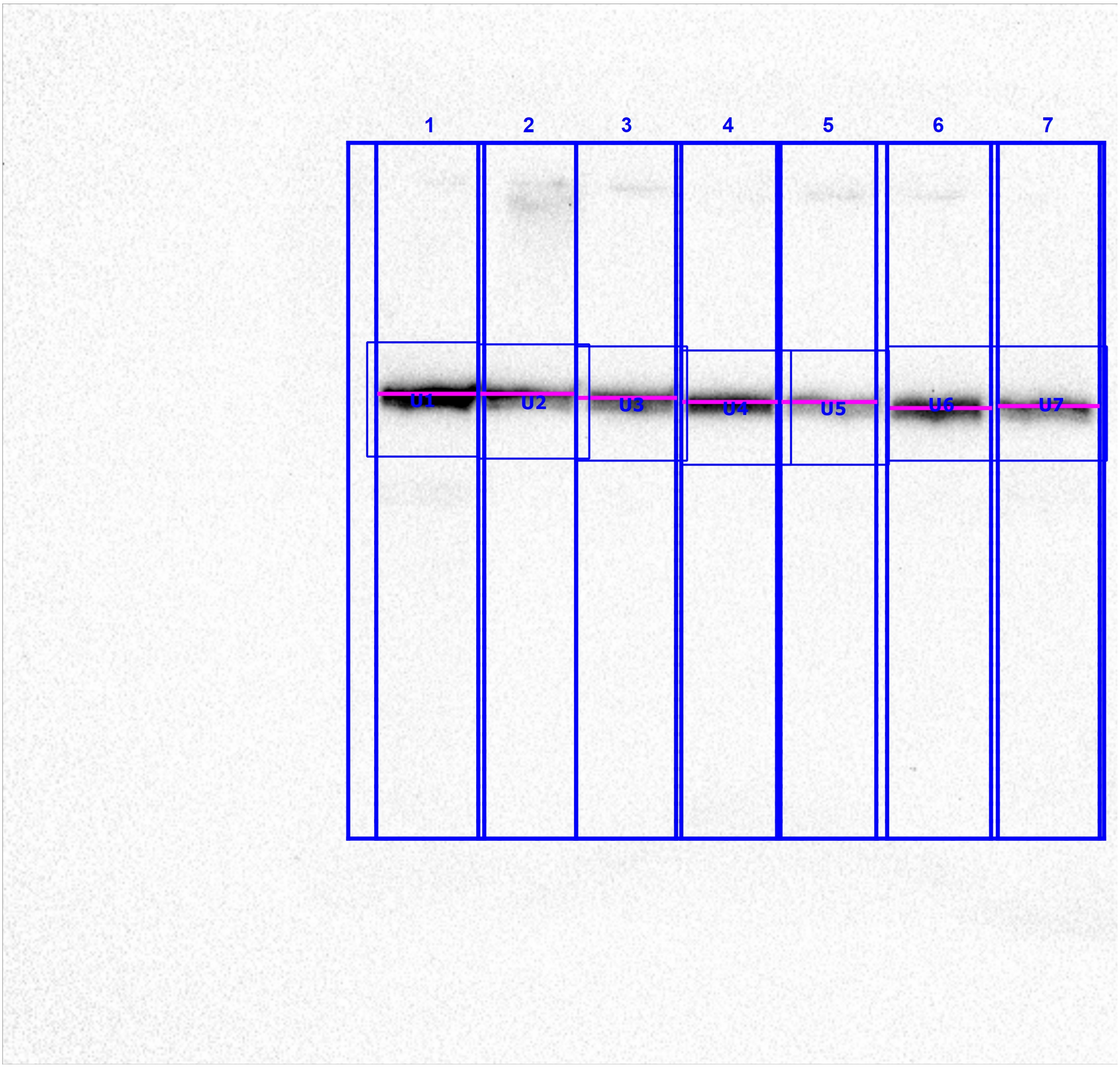

C:\Users\rusak\OneDrive\Dokumenty\Badania\CHI3L2 in BC\BC westerny ilościowo\STAT3 24.1.23 BC\2\Histologia 2023-01-24 13hr 50min_Exposure_60.0sec stat3 2b.scn

Acquisition Information

Imager	ChemiDoc MP
Exposure Time (sec)	60.000 (Signal Accumulation)
Flat Field	Applied (Lens)

Serial Number	731BR01769
Software Version	5.0
Application	Chemi Hi Resolution
Excitation Source	No Illumination
Emission Filter	No Filter
Binning	2x2

Image Information

Acquisition Date	24/1/2023 1:51:53 PM
User Name	Histologia
Image Area (mm)	X: 114.0 Y: 85.2
Pixel Size (μm)	X: 163.8 Y: 163.8
Data Range (Int)	0 - 24676

Analysis Settings

Detection	Lane detection:<br>Manually created lanes<br><br>Band detection:<br>Automatically detected bands with sensitivity: Low<br><br>Lane Background Subtraction:<br>Lane background subtracted with disk size: 10<br><br>Lane width: Variable
Volume Analysis	Background subtraction method: Local<br>Quantity regression method: Linear

Lane Statistics

Lane No.	Adj. Total Band Vol. (Int)	Total Band Vol. (Int)	Adj. Total Lane Vol. (Int)	Total Lane Vol. (Int)	Bkgd. Vol. (Int)	Norm. Factor
1	9 295 935	9 729 846	11 140 494	15 854 685	4 714 191	N/A
2	5 022 624	5 312 160	7 558 464	10 984 656	3 426 192	N/A
3	4 614 183	4 878 048	6 320 167	9 612 673	3 292 506	N/A
4	5 292 811	5 536 553	6 729 413	9 873 196	3 143 783	N/A
5	2 777 277	3 008 705	4 157 620	7 381 397	3 223 777	N/A
6	5 618 058	5 926 761	7 282 239	10 662 366	3 380 127	N/A
7	4 065 600	4 384 850	5 538 650	9 193 200	3 654 550	N/A

Lane And Band Analysis

Lane 1

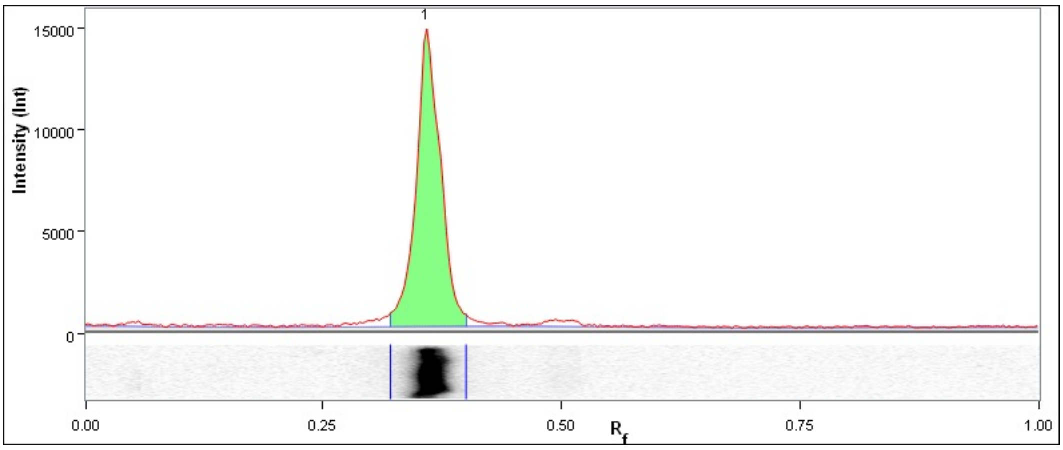

Band No.	Band Label	Mol. Wt. (KDa)	Relative Front	Adj. Volume (Int)	Volume (Int)	Abs. Quant.	Rel. Quant.	Band %	Lane %
1		N/A	0,361	9 295 935	9 729 846	N/A	N/A	100,0	83,4

Band Detection	Automatically detected bands with sensitivity: Low
Lane Background	Lane background subtracted with disk size: 10
Lane Width	8.68 mm

Lane 2

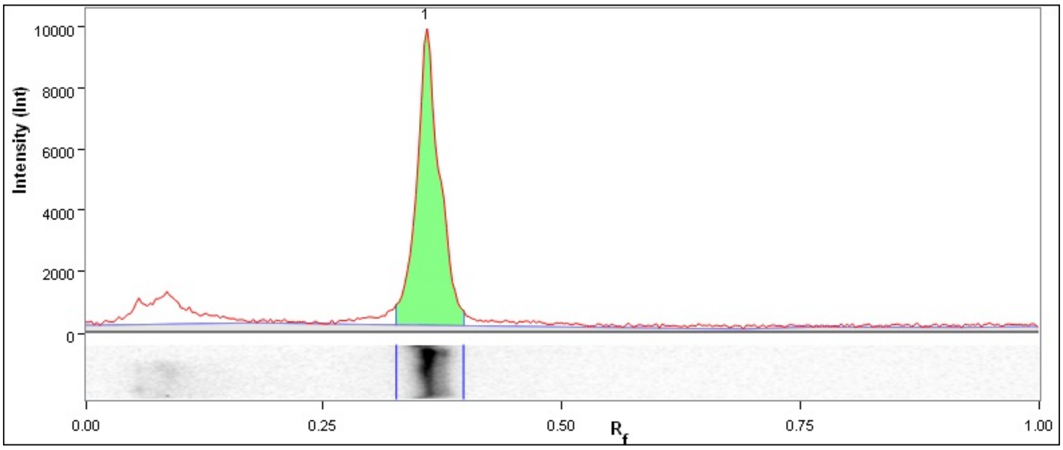

Band No.	Band Label	Mol. Wt. (KDa)	Relative Front	Adj. Volume (Int)	Volume (Int)	Abs. Quant.	Rel. Quant.	Band %	Lane %
1		N/A	0,361	5 022 624	5 312 160	N/A	N/A	100,0	66,5

Band Detection	Automatically detected bands with sensitivity: Low
Lane Background	Lane background subtracted with disk size: 10
Lane Width	7.86 mm

Lane 3

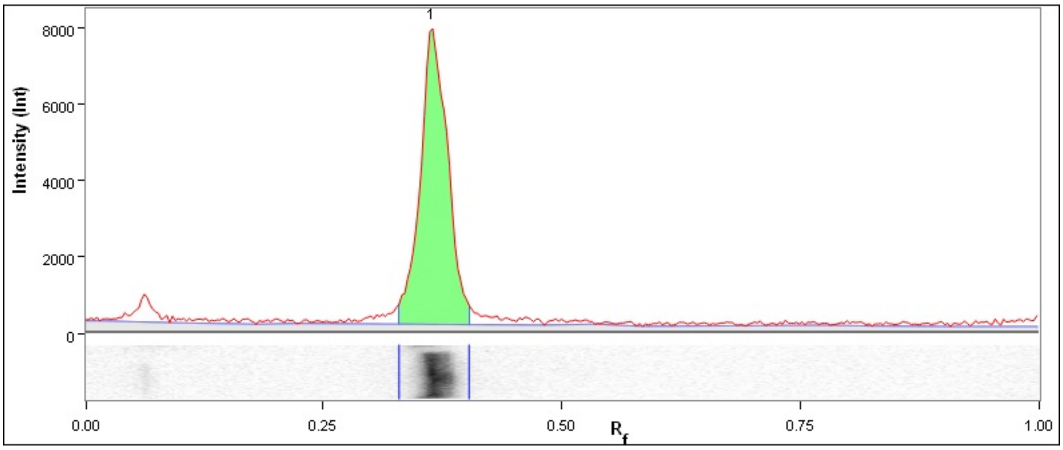

Band No.	Band Label	Mol. Wt. (KDa)	Relative Front	Adj. Volume (Int)	Volume (Int)	Abs. Quant.	Rel. Quant.	Band %	Lane %
1		N/A	0,367	4 614 183	4 878 048	N/A	N/A	100,0	73,0

Band Detection	Automatically detected bands with sensitivity: Low
Lane Background	Lane background subtracted with disk size: 10
Lane Width	8.03 mm

Lane 4

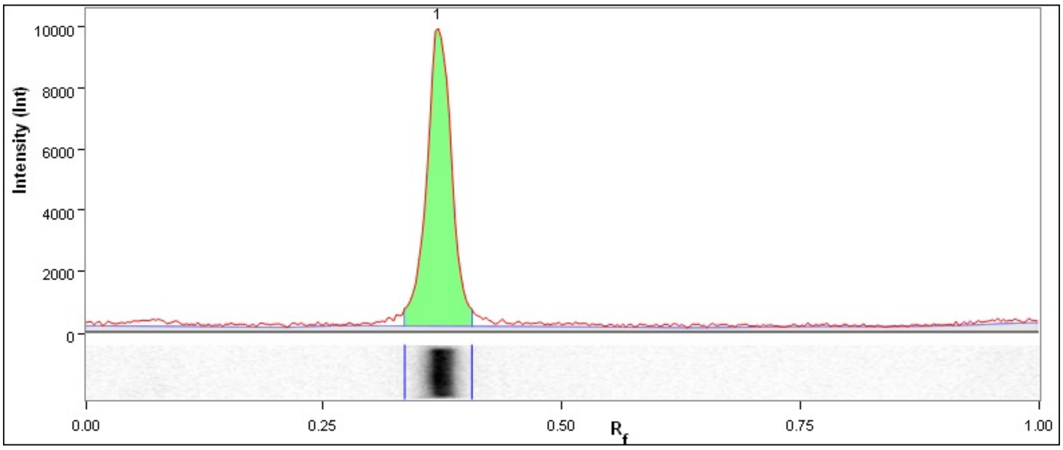

Band No.	Band Label	Mol. Wt. (KDa)	Relative Front	Adj. Volume (Int)	Volume (Int)	Abs. Quant.	Rel. Quant.	Band %	Lane %
1		N/A	0,372	5 292 811	5 536 553	N/A	N/A	100,0	78,7

Band Detection	Automatically detected bands with sensitivity: Low
Lane Background	Lane background subtracted with disk size: 10
Lane Width	7.70 mm

Lane 5

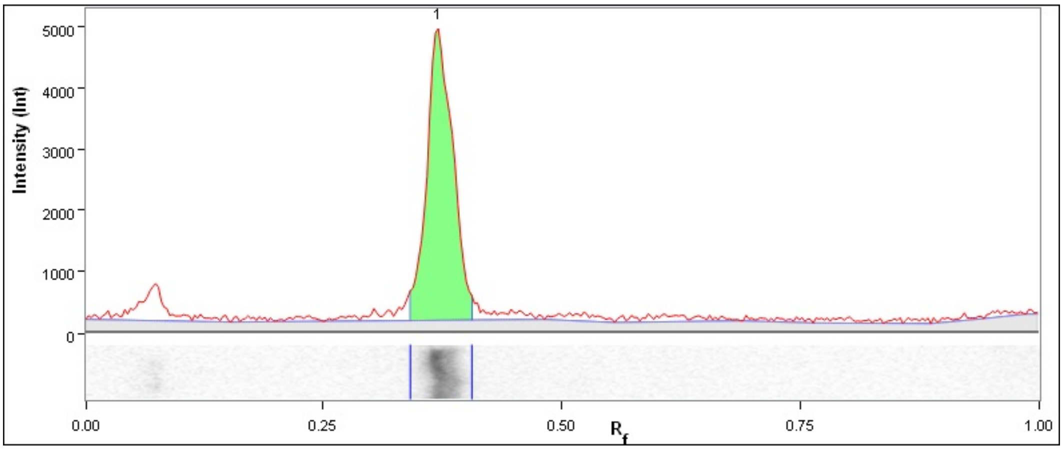

Band No.	Band Label	Mol. Wt. (KDa)	Relative Front	Adj. Volume (Int)	Volume (Int)	Abs. Quant.	Rel. Quant.	Band %	Lane %
1		N/A	0,372	2 777 277	3 008 705	N/A	N/A	100,0	66,8

Band Detection	Automatically detected bands with sensitivity: Low
Lane Background	Lane background subtracted with disk size: 10
Lane Width	7.70 mm

Lane 6

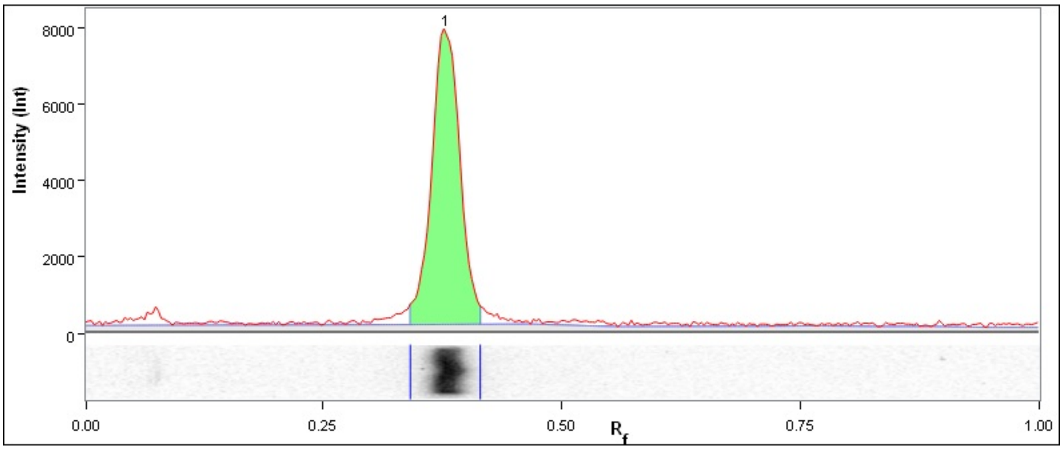

Band No.	Band Label	Mol. Wt. (KDa)	Relative Front	Adj. Volume (Int)	Volume (Int)	Abs. Quant.	Rel. Quant.	Band %	Lane %
1		N/A	0,381	5 618 058	5 926 761	N/A	N/A	100,0	77,1

Band Detection	Automatically detected bands with sensitivity: Low
Lane Background	Lane background subtracted with disk size: 10
Lane Width	8.35 mm

Lane 7

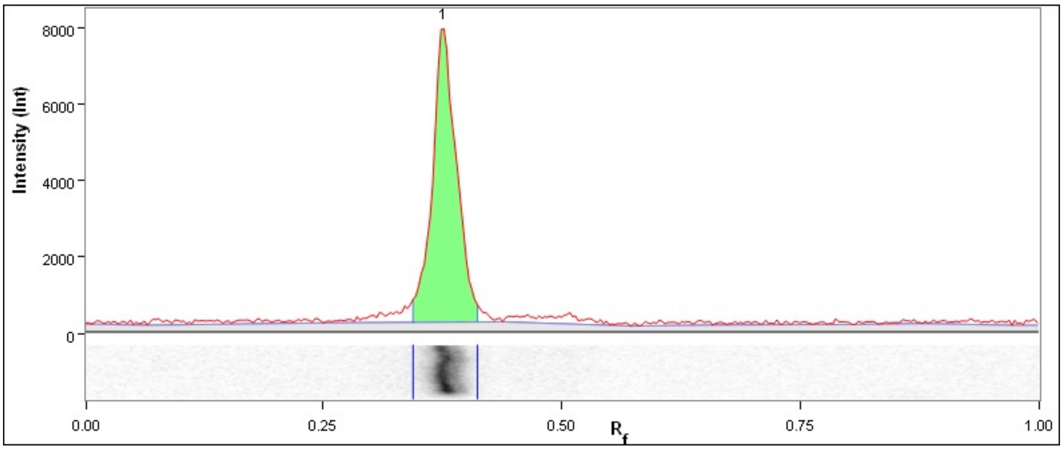

Band No.	Band Label	Mol. Wt. (KDa)	Relative Front	Adj. Volume (Int)	Volume (Int)	Abs. Quant.	Rel. Quant.	Band %	Lane %
1		N/A	0,378	4 065 600	4 384 850	N/A	N/A	100,0	73,4

Band Detection	Automatically detected bands with sensitivity: Low
Lane Background	Lane background subtracted with disk size: 10
Lane Width	8.19 mm

Volume Analysis

No.	Label	Type	Volume (Int)	Adj. Vol. (Int)	Mean Bkgd. (Int)	Abs. Quant.	Rel. Quant.	# of Pixels	Min. Value (Int)	Max. Value (Int)	Mean Value (Int)	Std. Dev.	Area (mm2)
1	U1	Unknown	10 349 624	7 453 334	957,8	N/A	N/A	3 024	0	24 676	3 422,5	5 147,6	81,1
2	U2	Unknown	6 383 576	2 608 166	1 248,5	N/A	N/A	3 024	0	17 364	2 111,0	2 946,8	81,1
3	U3	Unknown	5 854 352	2 906 762	974,7	N/A	N/A	3 024	0	11 660	1 936,0	2 573,7	81,1
4	U4	Unknown	6 540 888	4 206 090	772,1	N/A	N/A	3 024	0	13 532	2 163,0	3 209,7	81,1
5	U5	Unknown	3 989 016	1 914 174	686,1	N/A	N/A	3 024	0	7 972	1 319,1	1 646,1	81,1
6	U6	Unknown	6 782 516	4 799 852	655,6	N/A	N/A	3 024	0	12 484	2 242,9	3 105,6	81,1
7	U7	Unknown	5 246 536	3 682 750	517,1	N/A	N/A	3 024	0	11 764	1 735,0	2 381,7	81,1

Image Report: Histologia 2023-01-24 13hr 52min_Exposure_60.0sec stat3 3b

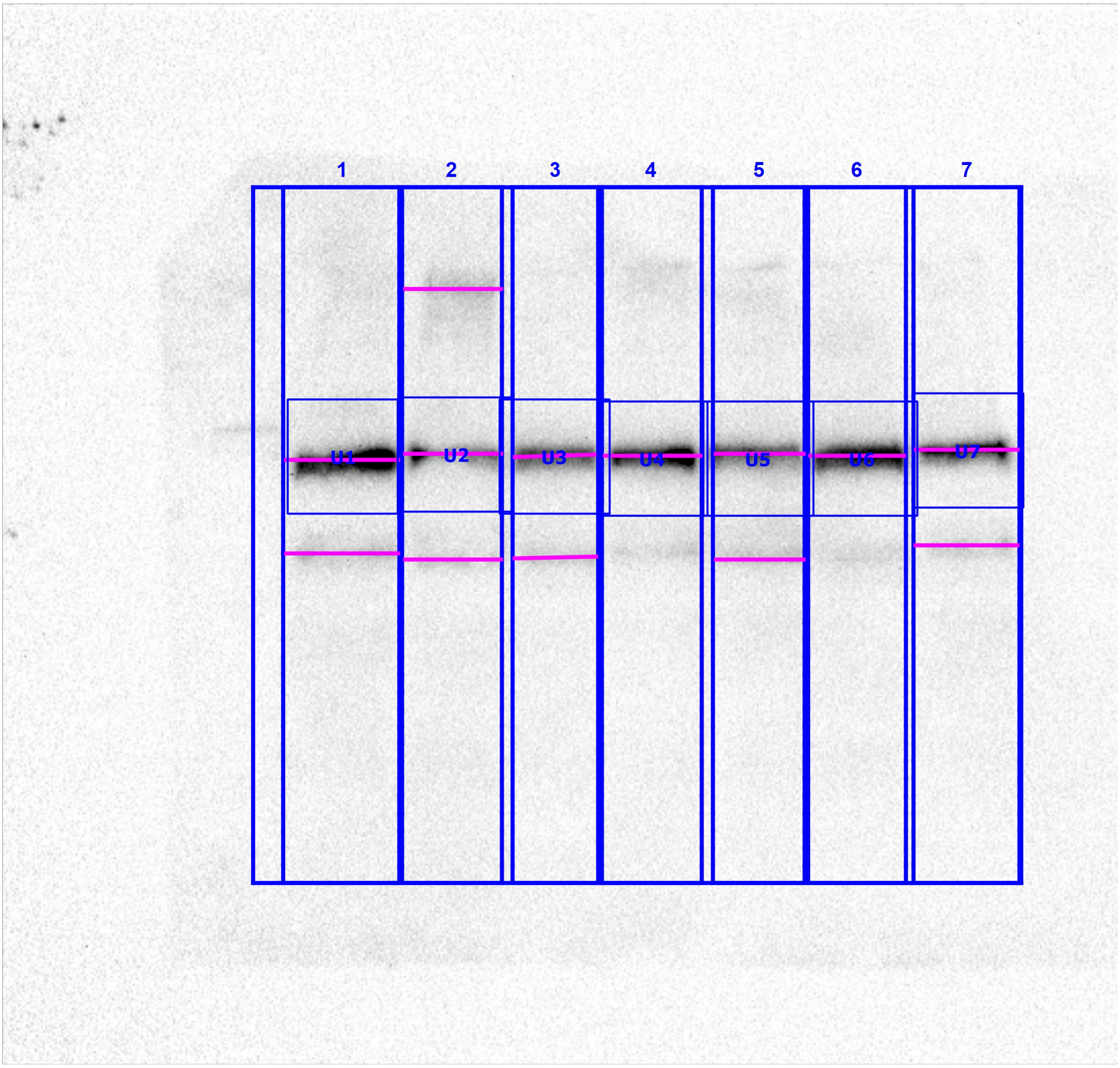

C:\Users\rusak\OneDrive\Dokumenty\Badania\CHI3L2 in BC\BC westerny ilościowo\STAT3 24.1.23 BC\3\Histologia 2023-01-24 13hr 52min_Exposure_60.0sec stat3 3b.scn

Acquisition Information

Imager	ChemiDoc MP
Exposure Time (sec)	60.000 (Signal Accumulation)
Flat Field	Applied (Lens)

Serial Number	731BR01769
Software Version	5.0
Application	Chemi Hi Resolution
Excitation Source	No Illumination
Emission Filter	No Filter
Binning	2x2

Image Information

Acquisition Date	24/1/2023 1:53:49 PM
User Name	Histologia
Image Area (mm)	X: 114.0 Y: 85.2
Pixel Size (μm)	X: 163.8 Y: 163.8
Data Range (Int)	0 - 21204

Analysis Settings

Detection	Lane detection:<br>Manually created lanes<br><br>Band detection:<br>Automatically detected bands with sensitivity: Low<br><br>Lane Background Subtraction:<br>Lane background subtracted with disk size: 10<br><br>Lane width: Variable
Volume Analysis	Background subtraction method: Local<br>Quantity regression method: Linear

Lane Statistics

Lane No.	Adj. Total Band Vol. (Int)	Total Band Vol. (Int)	Adj. Total Lane Vol. (Int)	Total Lane Vol. (Int)	Bkgd. Vol. (Int)	Norm. Factor
1	8 217 234	10 149 021	11 069 742	23 022 756	11 953 014	N/A
2	4 307 982	6 061 888	7 545 706	17 228 743	9 683 037	N/A
3	4 008 900	5 130 636	5 776 428	12 976 152	7 199 724	N/A
4	5 186 160	6 098 981	8 268 652	16 046 667	7 778 015	N/A
5	4 179 375	5 723 730	6 753 780	14 832 405	8 078 625	N/A
6	5 590 224	6 588 864	8 326 848	16 319 664	7 992 816	N/A
7	5 167 240	6 206 460	7 408 336	14 225 900	6 817 564	N/A

Lane And Band Analysis

Lane 1

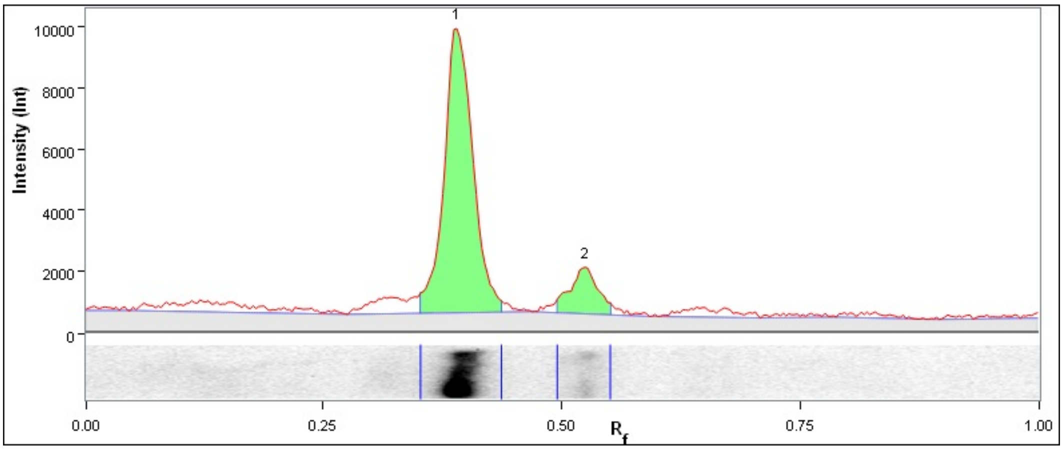

Band No.	Band Label	Mol. Wt. (KDa)	Relative Front	Adj. Volume (Int)	Volume (Int)	Abs. Quant.	Rel. Quant.	Band %	Lane %
1		N/A	0,392	7 088 634	8 273 892	N/A	N/A	86,3	64,0
2		N/A	0,526	1 128 600	1 875 129	N/A	N/A	13,7	10,2

Band Detection	Automatically detected bands with sensitivity: Low
Lane Background	Lane background subtracted with disk size: 10
Lane Width	9.34 mm

Lane 2

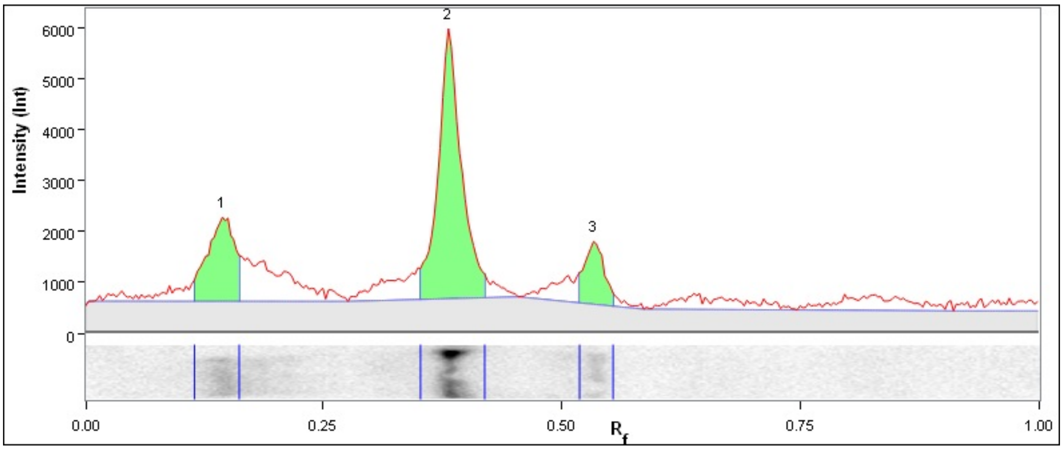

Band No.	Band Label	Mol. Wt. (KDa)	Relative Front	Adj. Volume (Int)	Volume (Int)	Abs. Quant.	Rel. Quant.	Band %	Lane %
1		N/A	0,146	1 012 046	1 555 162	N/A	N/A	23,5	13,4
2		N/A	0,383	2 748 459	3 586 310	N/A	N/A	63,8	36,4
3		N/A	0,535	547 477	920 416	N/A	N/A	12,7	7,3

Band Detection	Automatically detected bands with sensitivity: Low
Lane Background	Lane background subtracted with disk size: 10
Lane Width	8.03 mm

Lane 3

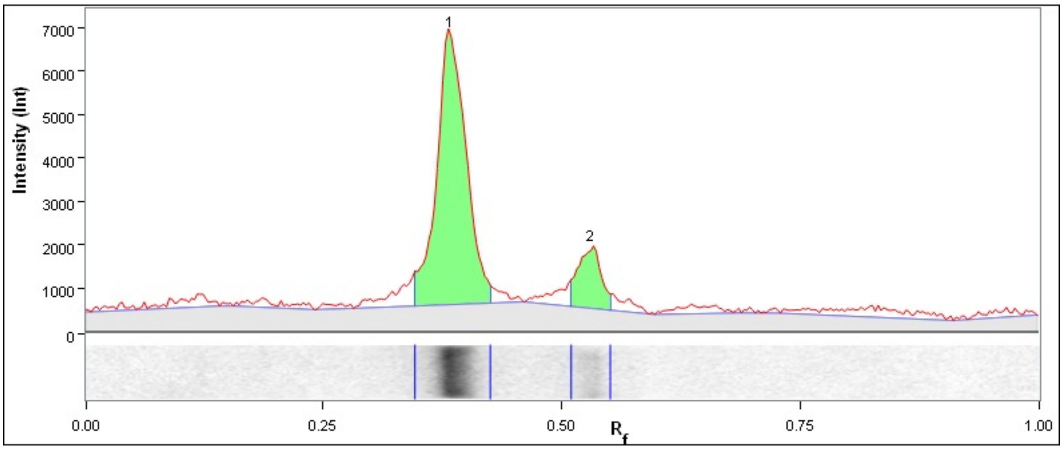

Band No.	Band Label	Mol. Wt. (KDa)	Relative Front	Adj. Volume (Int)	Volume (Int)	Abs. Quant.	Rel. Quant.	Band %	Lane %
1		N/A	0,386	3 401 034	4 168 248	N/A	N/A	84,8	58,9
2		N/A	0,532	607 866	962 388	N/A	N/A	15,2	10,5

Band Detection	Automatically detected bands with sensitivity: Low
Lane Background	Lane background subtracted with disk size: 10
Lane Width	6.88 mm

Lane 4

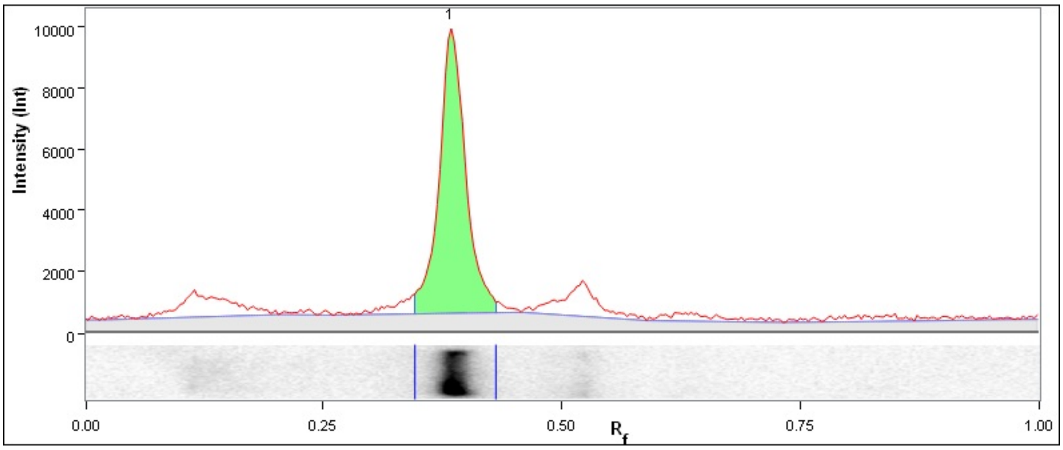

Band No.	Band Label	Mol. Wt. (KDa)	Relative Front	Adj. Volume (Int)	Volume (Int)	Abs. Quant.	Rel. Quant.	Band %	Lane %
1		N/A	0,386	5 186 160	6 098 981	N/A	N/A	100,0	62,7

Band Detection	Automatically detected bands with sensitivity: Low
Lane Background	Lane background subtracted with disk size: 10
Lane Width	8.03 mm

Lane 5

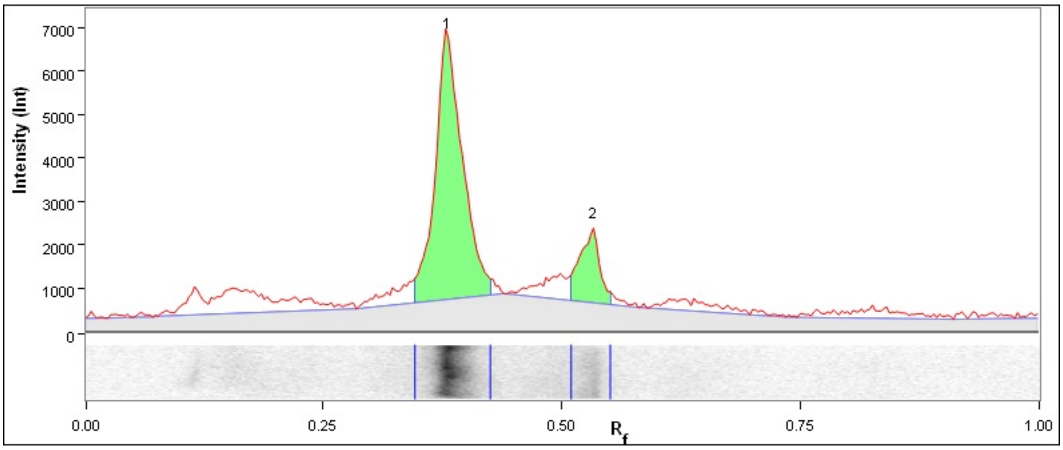

Band No.	Band Label	Mol. Wt. (KDa)	Relative Front	Adj. Volume (Int)	Volume (Int)	Abs. Quant.	Rel. Quant.	Band %	Lane %
1		N/A	0,383	3 474 945	4 521 150	N/A	N/A	83,1	51,5
2		N/A	0,535	704 430	1 202 580	N/A	N/A	16,9	10,4

Band Detection	Automatically detected bands with sensitivity: Low
Lane Background	Lane background subtracted with disk size: 10
Lane Width	7.37 mm

Lane 6

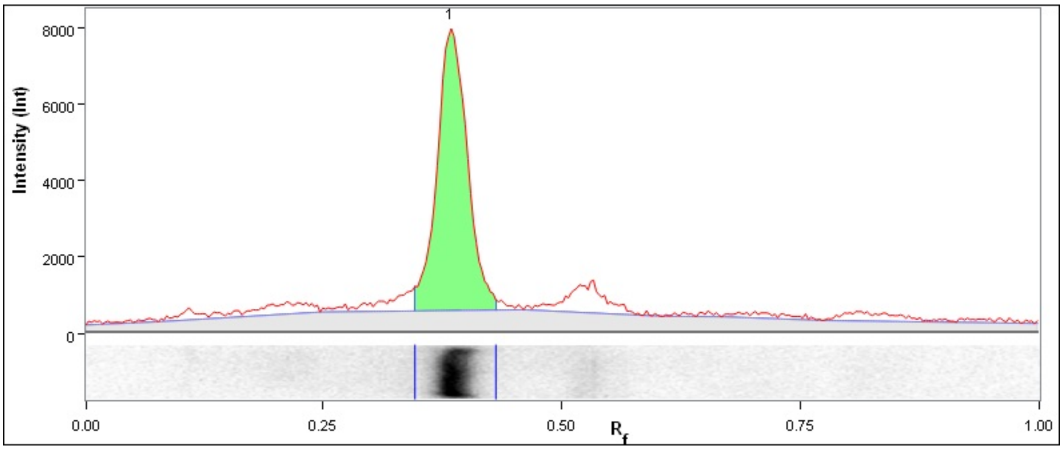

Band No.	Band Label	Mol. Wt. (KDa)	Relative Front	Adj. Volume (Int)	Volume (Int)	Abs. Quant.	Rel. Quant.	Band %	Lane %
1		N/A	0,386	5 590 224	6 588 864	N/A	N/A	100,0	67,1

Band Detection	Automatically detected bands with sensitivity: Low
Lane Background	Lane background subtracted with disk size: 10
Lane Width	7.86 mm

Lane 7

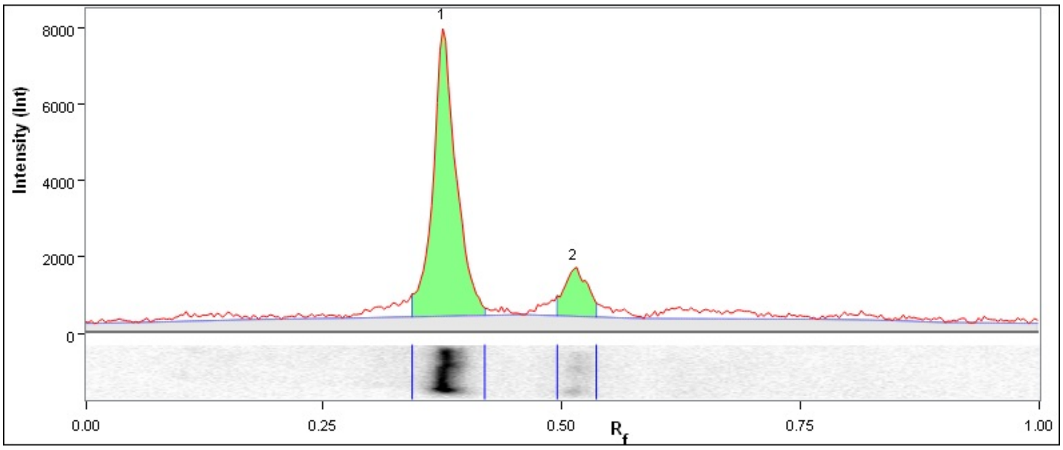

Band No.	Band Label	Mol. Wt. (KDa)	Relative Front	Adj. Volume (Int)	Volume (Int)	Abs. Quant.	Rel. Quant.	Band %	Lane %
1		N/A	0,377	4 410 588	5 082 376	N/A	N/A	85,4	59,5
2		N/A	0,515	756 652	1 124 084	N/A	N/A	14,6	10,2

Band Detection	Automatically detected bands with sensitivity: Low
Lane Background	Lane background subtracted with disk size: 10
Lane Width	8.52 mm

Volume Analysis

No.	Label	Type	Volume (Int)	Adj. Vol. (Int)	Mean Bkgd. (Int)	Abs. Quant.	Rel. Quant.	# of Pixels	Min. Value (Int)	Max. Value (Int)	Mean Value (Int)	Std. Dev.	Area (mm2)
1	U1	Unknown	9 713 544	6 314 406	1 124,1	N/A	N/A	3 024	0	21 204	3 212,2	3 780,5	81,1
2	U2	Unknown	5 531 904	1 937 070	1 188,8	N/A	N/A	3 024	0	14 388	1 829,3	1 697,8	81,1
3	U3	Unknown	6 082 872	2 402 718	1 217,0	N/A	N/A	3 024	0	9 316	2 011,5	1 892,5	81,1
4	U4	Unknown	7 593 396	4 069 140	1 165,4	N/A	N/A	3 024	0	18 592	2 511,0	2 963,9	81,1
5	U5	Unknown	6 677 200	2 294 506	1 449,3	N/A	N/A	3 024	0	9 860	2 208,1	1 896,0	81,1
6	U6	Unknown	8 257 128	4 711 704	1 172,4	N/A	N/A	3 024	0	12 596	2 730,5	2 900,5	81,1
7	U7	Unknown	6 241 668	4 145 226	693,3	N/A	N/A	3 024	0	14 004	2 064,0	2 583,0	81,1

Image Report: Histologia 2023-01-19 14hr 27min_Exposure_16.8sec pstat3 2c

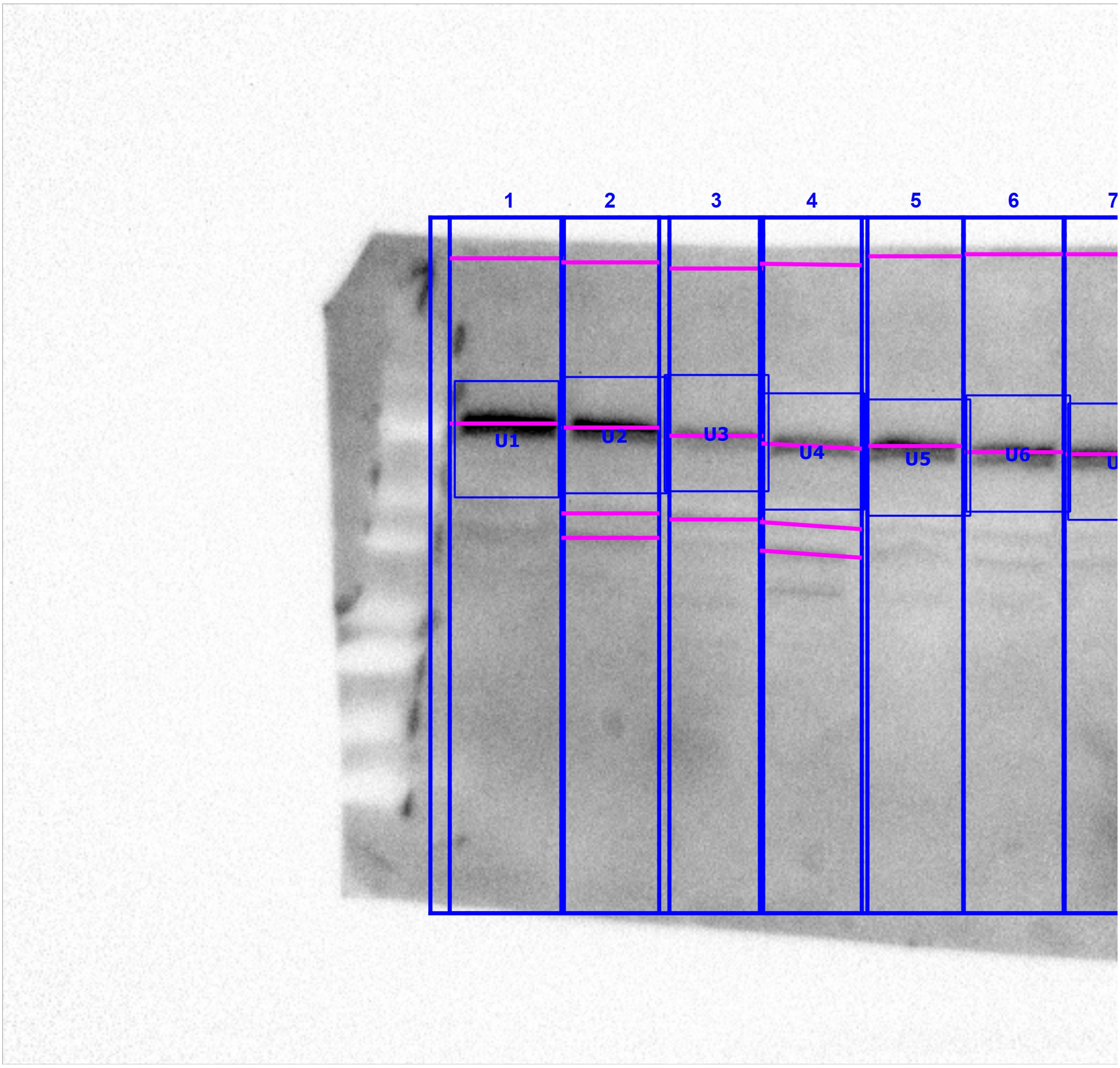

C:\Users\rusak\OneDrive\Dokumenty\Badania\CHI3L2 in BC\BC westerny ilościowo\pSTAT3 19.1.23\2\Histologia 2023-01-19 14hr 27min_Exposure_16.8sec pstat3 2c.scn

Acquisition Information

Imager	ChemiDoc MP
Exposure Time (sec)	16.800 (Signal Accumulation)
Flat Field	Applied (Lens)

Serial Number	731BR01769
Software Version	5.0
Application	Chemi Hi Resolution
Excitation Source	No Illumination
Emission Filter	No Filter
Binning	2x2

Image Information

Acquisition Date	19/1/2023 2:28:34 PM
User Name	Histologia
Image Area (mm)	X: 114.0 Y: 85.2
Pixel Size (μm)	X: 163.8 Y: 163.8
Data Range (Int)	0 - 15340

Analysis Settings

Detection	Lane detection:<br>Manually created lanes<br><br>Band detection:<br>Automatically detected bands with sensitivity: Low<br><br>Lane Background Subtraction:<br>Lane background subtracted with disk size: 10<br><br>Lane width: Variable
Volume Analysis	Background subtraction method: Local<br>Quantity regression method: Linear

Lane Statistics

Lane No.	Adj. Total Band Vol. (Int)	Total Band Vol. (Int)	Adj. Total Lane Vol. (Int)	Total Lane Vol. (Int)	Bkgd. Vol. (Int)	Norm. Factor
1	8 745 968	27 282 752	14 449 232	72 086 392	57 637 160	N/A
2	7 071 360	24 591 792	10 128 912	58 403 808	48 274 896	N/A
3	4 738 874	17 675 316	7 886 102	48 751 858	40 865 756	N/A
4	5 690 750	20 083 650	8 004 800	48 699 750	40 694 950	N/A
5	5 540 407	16 542 872	8 203 615	42 629 282	34 425 667	N/A
6	4 621 141	16 587 774	6 663 314	43 938 545	37 275 231	N/A
7	4 256 581	18 051 943	6 812 519	42 989 611	36 177 092	N/A

Lane And Band Analysis

Lane 1

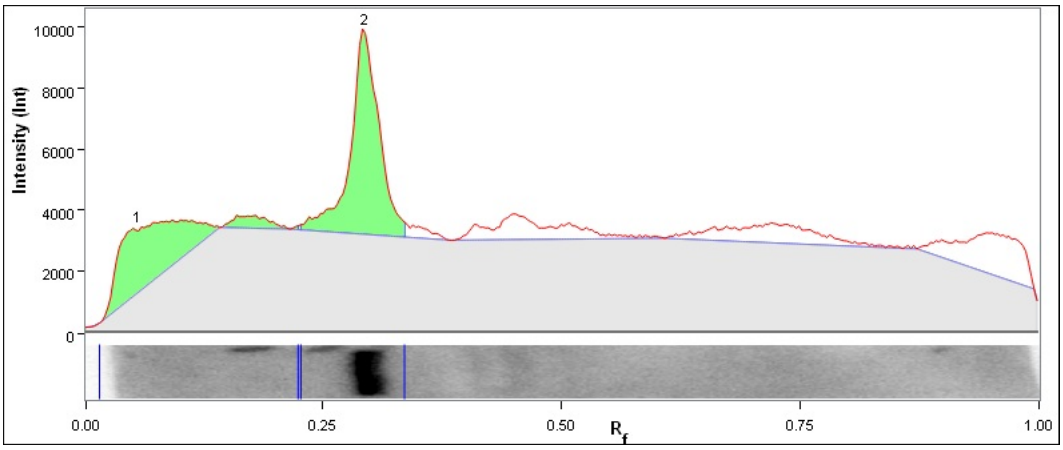

Band No.	Band Label	Mol. Wt. (KDa)	Relative Front	Adj. Volume (Int)	Volume (Int)	Abs. Quant.	Rel. Quant.	Band %	Lane %
1		N/A	0,059	3 772 160	14 709 184	N/A	N/A	43,1	26,1
2		N/A	0,296	4 973 808	12 573 568	N/A	N/A	56,9	34,4

Band Detection	Automatically detected bands with sensitivity: Low
Lane Background	Lane background subtracted with disk size: 10
Lane Width	9.17 mm

Lane 2

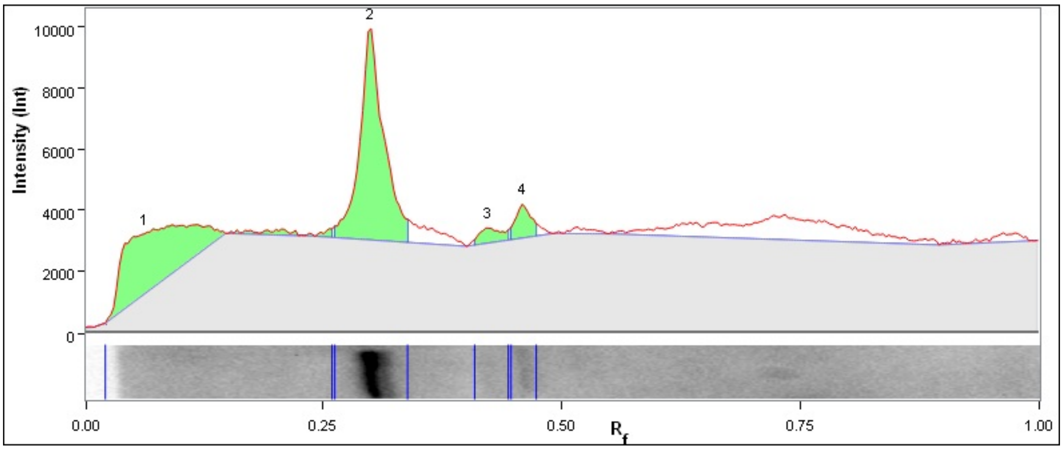

Band No.	Band Label	Mol. Wt. (KDa)	Relative Front	Adj. Volume (Int)	Volume (Int)	Abs. Quant.	Rel. Quant.	Band %	Lane %
1		N/A	0,065	2 953 824	12 907 680	N/A	N/A	41,8	29,2
2		N/A	0,302	3 518 544	7 615 536	N/A	N/A	49,8	34,7
3		N/A	0,425	236 256	2 153 088	N/A	N/A	3,3	2,3
4		N/A	0,460	362 736	1 915 488	N/A	N/A	5,1	3,6

Band Detection	Automatically detected bands with sensitivity: Low
Lane Background	Lane background subtracted with disk size: 10
Lane Width	7.86 mm

Lane 3

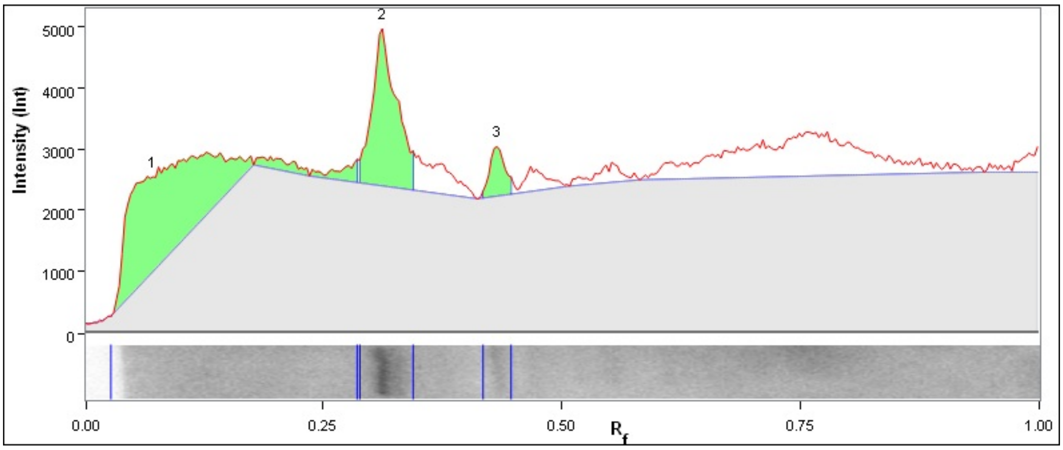

Band No.	Band Label	Mol. Wt. (KDa)	Relative Front	Adj. Volume (Int)	Volume (Int)	Abs. Quant.	Rel. Quant.	Band %	Lane %
1		N/A	0,073	3 059 230	12 190 230	N/A	N/A	64,6	38,8
2		N/A	0,314	1 401 298	3 915 474	N/A	N/A	29,6	17,8
3		N/A	0,434	278 346	1 569 612	N/A	N/A	5,9	3,5

Band Detection	Automatically detected bands with sensitivity: Low
Lane Background	Lane background subtracted with disk size: 10
Lane Width	7.53 mm

Lane 4

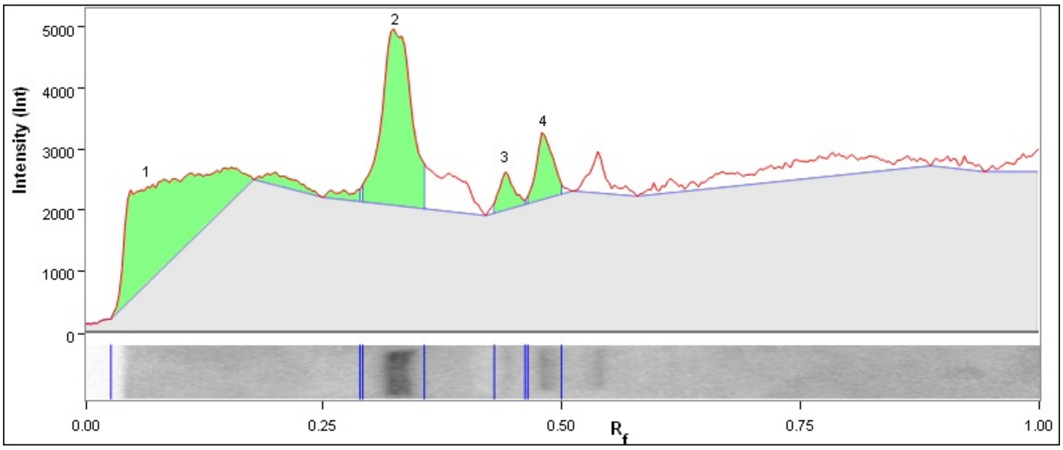

Band No.	Band Label	Mol. Wt. (KDa)	Relative Front	Adj. Volume (Int)	Volume (Int)	Abs. Quant.	Rel. Quant.	Band %	Lane %
1		N/A	0,067	3 031 900	11 816 750	N/A	N/A	53,3	37,9
2		N/A	0,328	2 024 450	4 693 500	N/A	N/A	35,6	25,3
3		N/A	0,443	214 750	1 568 500	N/A	N/A	3,8	2,7
4		N/A	0,484	419 650	2 004 900	N/A	N/A	7,4	5,2

Band Detection	Automatically detected bands with sensitivity: Low
Lane Background	Lane background subtracted with disk size: 10
Lane Width	8.19 mm

Lane 5

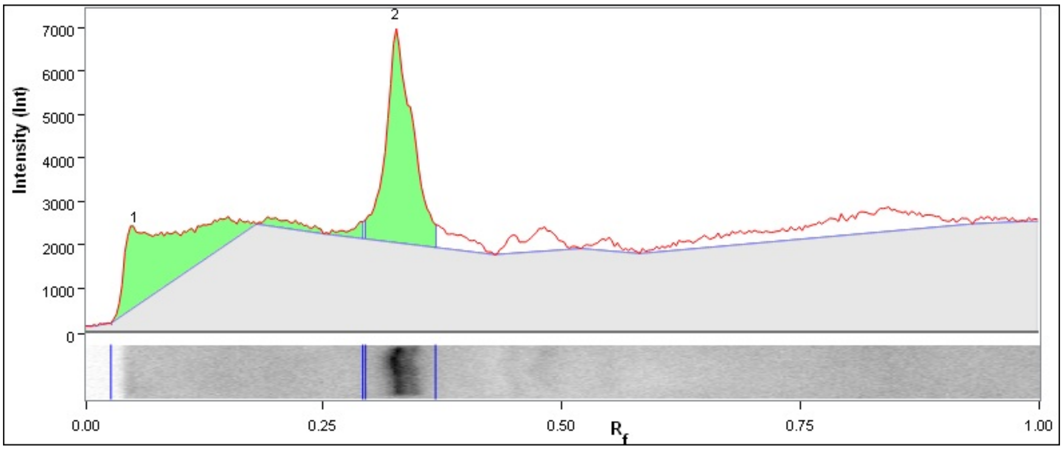

Band No.	Band Label	Mol. Wt. (KDa)	Relative Front	Adj. Volume (Int)	Volume (Int)	Abs. Quant.	Rel. Quant.	Band %	Lane %
1		N/A	0,056	2 701 607	10 943 574	N/A	N/A	48,8	32,9
2		N/A	0,328	2 838 800	5 599 298	N/A	N/A	51,2	34,6

Band Detection	Automatically detected bands with sensitivity: Low
Lane Background	Lane background subtracted with disk size: 10
Lane Width	7.70 mm

Lane 6

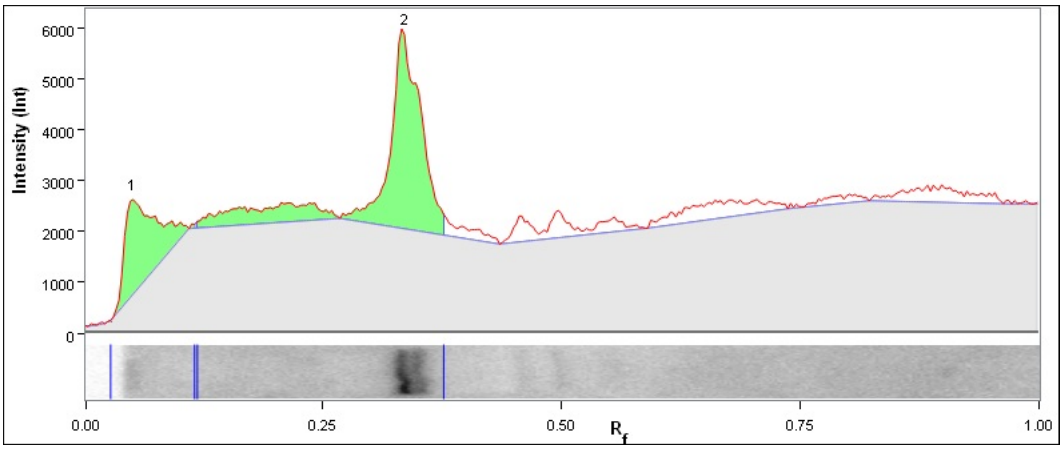

Band No.	Band Label	Mol. Wt. (KDa)	Relative Front	Adj. Volume (Int)	Volume (Int)	Abs. Quant.	Rel. Quant.	Band %	Lane %
1		N/A	0,053	1 266 013	3 226 356	N/A	N/A	27,4	19,0
2		N/A	0,337	3 355 128	13 361 418	N/A	N/A	72,6	50,4

Band Detection	Automatically detected bands with sensitivity: Low
Lane Background	Lane background subtracted with disk size: 10
Lane Width	8.03 mm

Lane 7

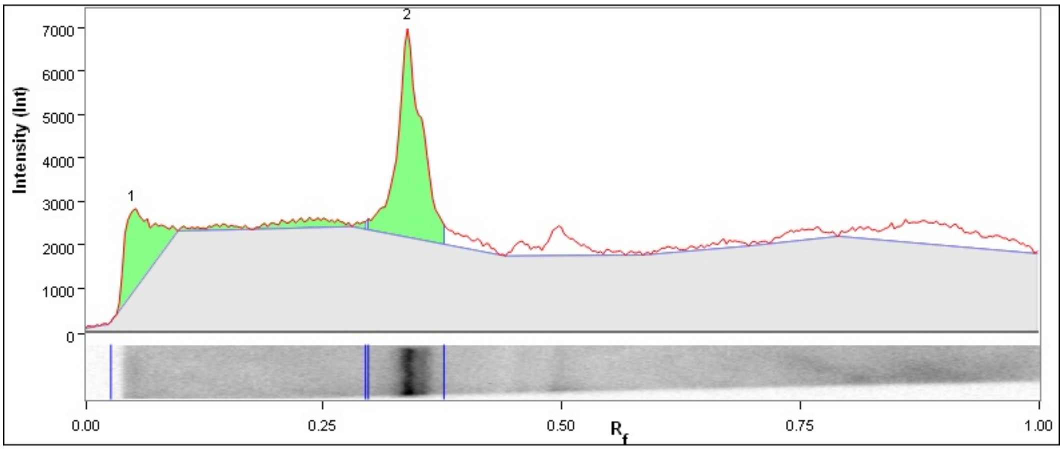

Band No.	Band Label	Mol. Wt. (KDa)	Relative Front	Adj. Volume (Int)	Volume (Int)	Abs. Quant.	Rel. Quant.	Band %	Lane %
1		N/A	0,053	1 577 212	12 023 179	N/A	N/A	37,1	23,2
2		N/A	0,340	2 679 369	6 028 764	N/A	N/A	62,9	39,3

Band Detection	Automatically detected bands with sensitivity: Low
Lane Background	Lane background subtracted with disk size: 10
Lane Width	8.03 mm

Volume Analysis

No.	Label	Type	Volume (Int)	Adj. Vol. (Int)	Mean Bkgd. (Int)	Abs. Quant.	Rel. Quant.	# of Pixels	Min. Value (Int)	Max. Value (Int)	Mean Value (Int)	Std. Dev.	Area (mm2)
1	U1	Unknown	15 081 088	3 012 968	4 151,4	N/A	N/A	2 907	2 308	14 268	5 187,9	2 456,7	78,0
2	U2	Unknown	13 151 992	2 790 651	3 564,3	N/A	N/A	2 907	2 372	15 340	4 524,2	2 035,7	78,0
3	U3	Unknown	10 090 628	760 585	3 209,5	N/A	N/A	2 907	2 068	6 804	3 471,1	786,5	78,0
4	U4	Unknown	9 384 580	1 410 256	2 743,1	N/A	N/A	2 907	1 460	7 268	3 228,3	1 059,7	78,0
5	U5	Unknown	9 815 372	2 078 999	2 661,3	N/A	N/A	2 907	1 188	10 312	3 376,5	1 566,4	78,0
6	U6	Unknown	9 326 856	1 388 050	2 730,9	N/A	N/A	2 907	1 296	9 256	3 208,4	1 344,0	78,0
7	U7	Unknown	9 320 988	3 796 631	1 900,4	N/A	N/A	2 907	0	10 684	3 206,4	1 841,7	78,0

Image Report: Histologia 2023-01-13 14hr 43min_Exposure_16.8sec pstat3 3b

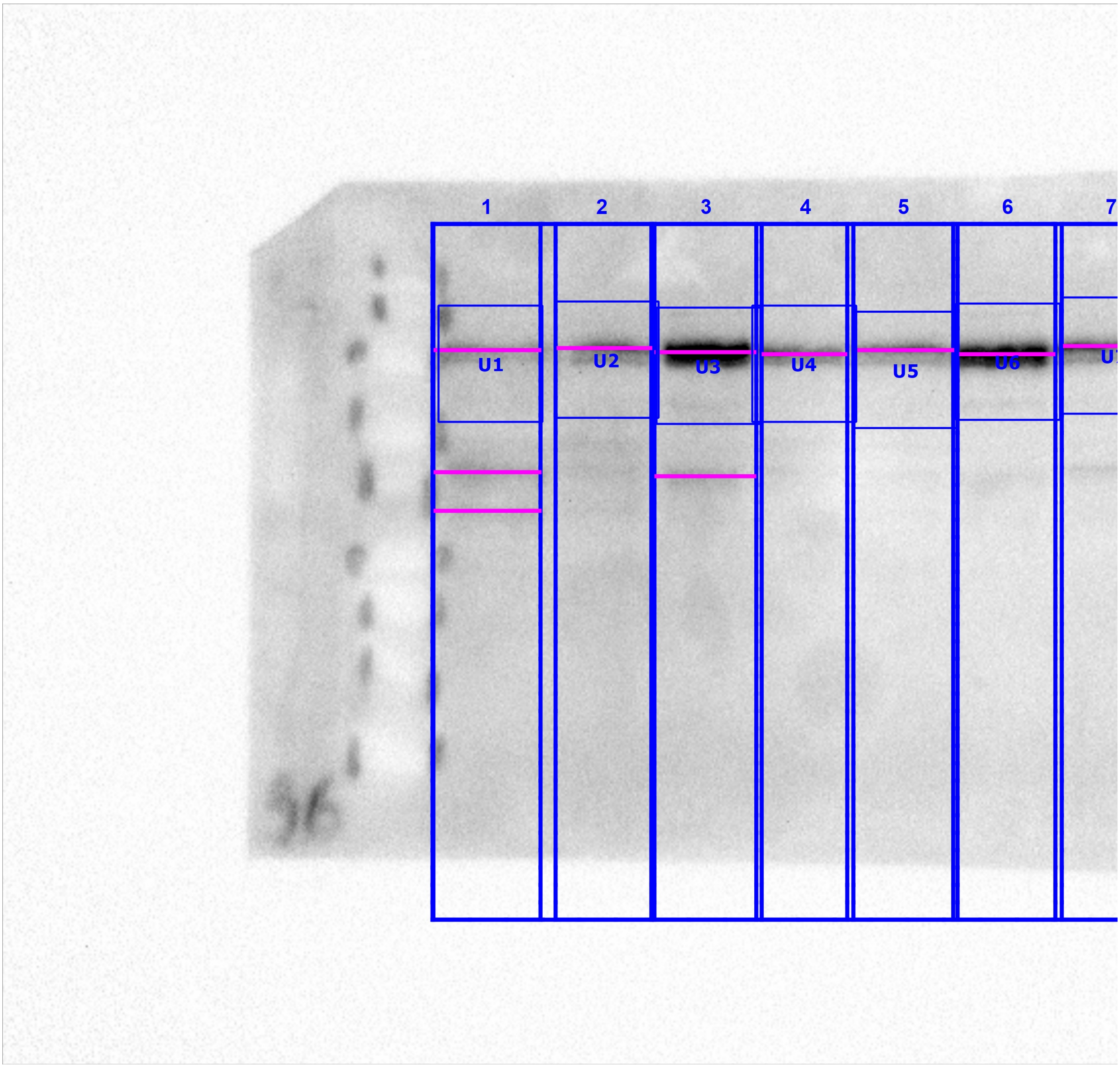

C:\Users\rusak\OneDrive\Dokumenty\Badania\CHI3L2 in BC\BC westerny ilościowo\p-STAT3 13.01.23\3\Histologia 2023-01-13 14hr 43min_Exposure_16.8sec pstat3 3b.scn

Acquisition Information

Imager	ChemiDoc MP
Exposure Time (sec)	16.800 (Signal Accumulation)
Flat Field	Applied (Lens)

Serial Number	731BR01769
Software Version	5.0
Application	Chemi Hi Resolution
Excitation Source	No Illumination
Emission Filter	No Filter
Binning	2x2

Image Information

Acquisition Date	13/1/2023 2:44:17 PM
User Name	Histologia
Image Area (mm)	X: 114.0 Y: 85.2
Pixel Size (µm)	X: 163.8 Y: 163.8
Data Range (Int)	0 - 17592

Analysis Settings

Detection	Lane detection:<br>Manually created lanes<br><br>Band detection:<br>Automatically detected bands with sensitivity: Low<br><br>Lane Background Subtraction:<br>Lane background subtracted with disk size: 10<br><br>Lane width: Variable
Volume Analysis	Background subtraction method: Local<br>Quantity regression method: Linear

Lane Statistics

Lane No.	Adj. Total Band Vol. (Int)	Total Band Vol. (Int)	Adj. Total Lane Vol. (Int)	Total Lane Vol. (Int)	Bkgd. Vol. (Int)	Norm. Factor
1	2 686 040	8 758 091	8 846 866	42 786 741	33 939 875	N/A
2	2 078 434	4 508 334	8 843 050	37 661 805	28 818 755	N/A
3	6 620 450	15 025 300	12 679 250	41 306 600	28 627 350	N/A
4	1 955 730	3 555 006	5 326 692	25 077 486	19 750 794	N/A
5	2 289 672	4 031 818	5 583 109	25 347 455	19 764 346	N/A
6	5 780 928	7 622 736	11 166 192	27 771 552	16 605 360	N/A
7	2 903 664	4 519 536	8 918 832	25 329 408	16 410 576	N/A

Lane And Band Analysis

Lane 1

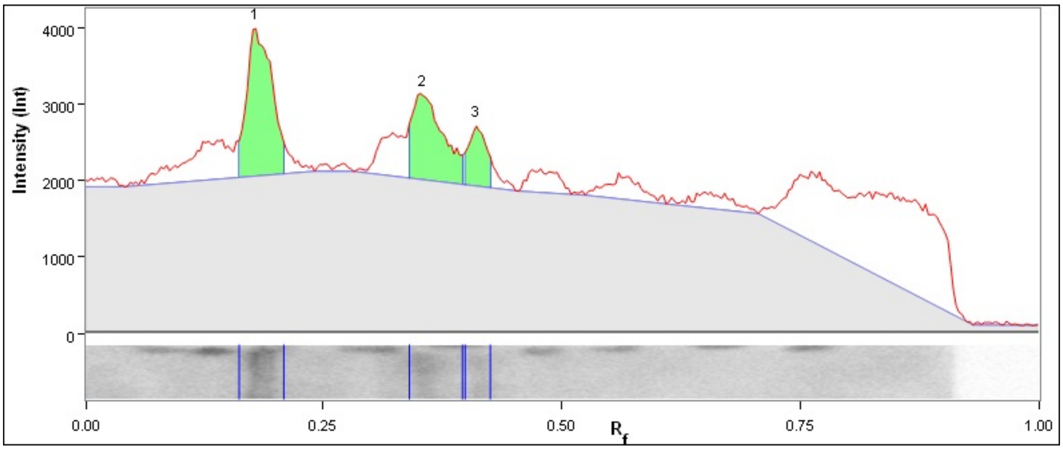

Band No.	Band Label	Mol. Wt. (KDa)	Relative Front	Adj. Volume (Int)	Volume (Int)	Abs. Quant.	Rel. Quant.	Band %	Lane %
1		N/A	0,181	1 331 625	3 595 255	N/A	N/A	49,6	15,1
2		N/A	0,357	970 430	3 537 750	N/A	N/A	36,1	11,0
3		N/A	0,412	383 985	1 625 086	N/A	N/A	14,3	4,3

Band Detection	Automatically detected bands with sensitivity: Low
Lane Background	Lane background subtracted with disk size: 10
Lane Width	8.68 mm

Lane 2

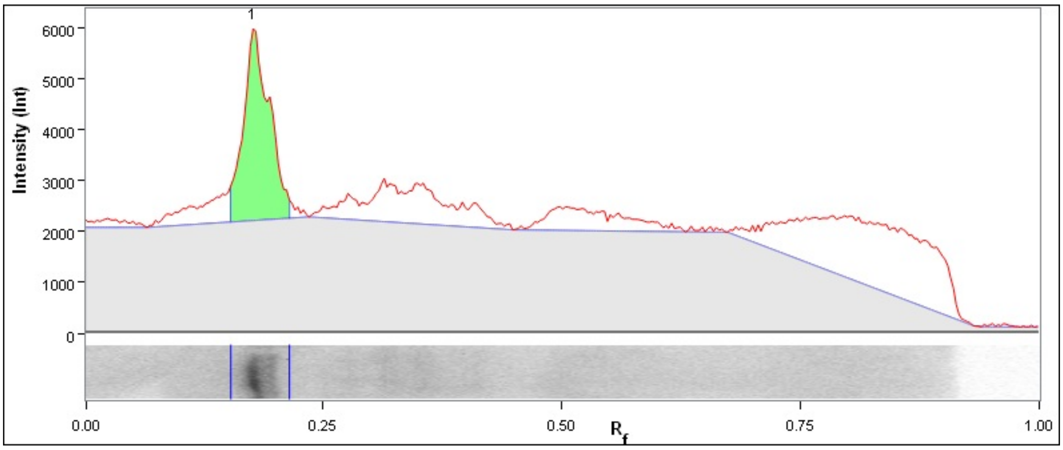

Band No.	Band Label	Mol. Wt. (KDa)	Relative Front	Adj. Volume (Int)	Volume (Int)	Abs. Quant.	Rel. Quant.	Band %	Lane %
1		N/A	0,178	2 078 434	4 508 334	N/A	N/A	100,0	23,5

Band Detection	Automatically detected bands with sensitivity: Low
Lane Background	Lane background subtracted with disk size: 10
Lane Width	7.70 mm

Lane 3

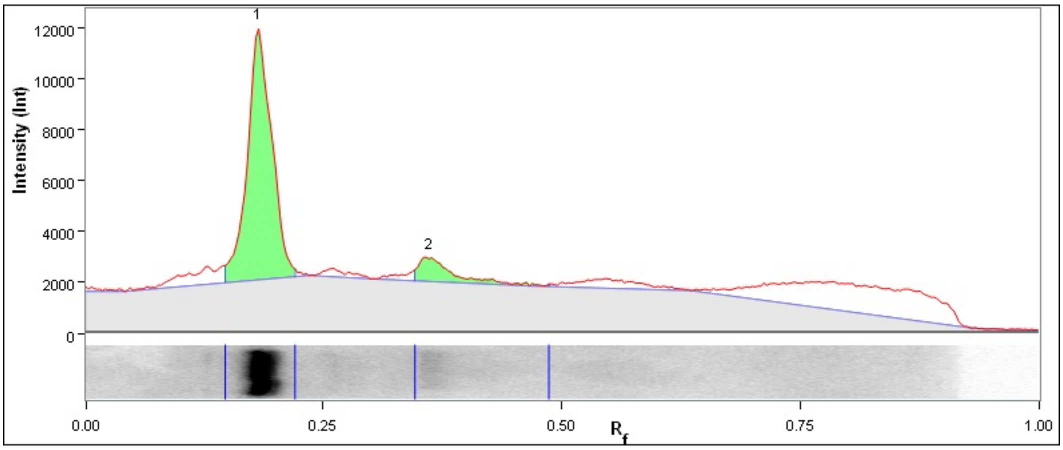

Band No.	Band Label	Mol. Wt. (KDa)	Relative Front	Adj. Volume (Int)	Volume (Int)	Abs. Quant.	Rel. Quant.	Band %	Lane %
1		N/A	0,184	5 804 550	8 877 100	N/A	N/A	87,7	45,8
2		N/A	0,363	815 900	6 148 200	N/A	N/A	12,3	6,4

Band Detection	Automatically detected bands with sensitivity: Low
Lane Background	Lane background subtracted with disk size: 10
Lane Width	8.19 mm

Lane 4

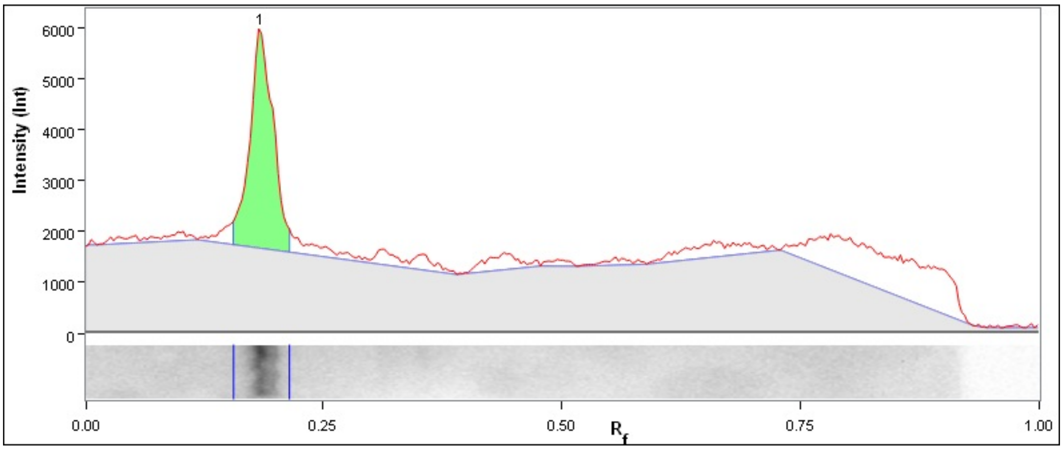

Band No.	Band Label	Mol. Wt. (KDa)	Relative Front	Adj. Volume (Int)	Volume (Int)	Abs. Quant.	Rel. Quant.	Band %	Lane %
1		N/A	0,187	1 955 730	3 555 006	N/A	N/A	100,0	36,7

Band Detection	Automatically detected bands with sensitivity: Low
Lane Background	Lane background subtracted with disk size: 10
Lane Width	6.88 mm

Lane 5

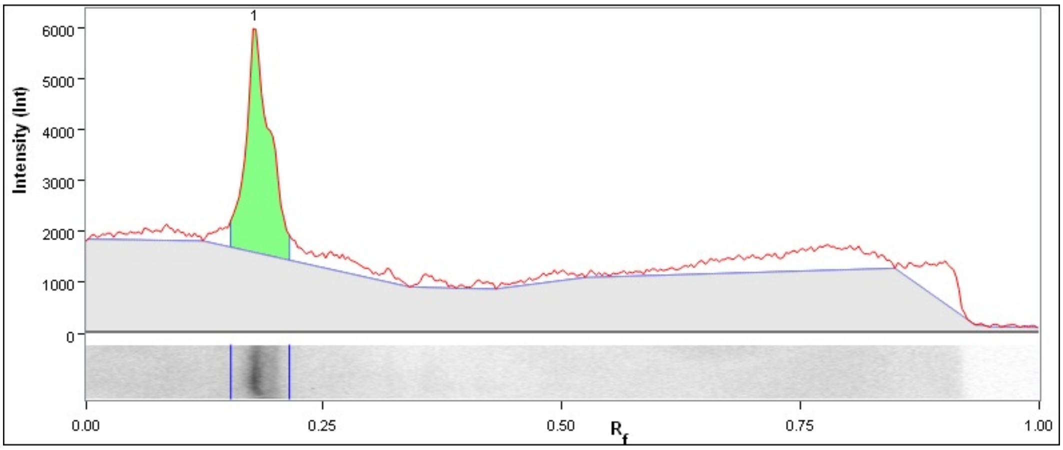

Band No.	Band Label	Mol. Wt. (KDa)	Relative Front	Adj. Volume (Int)	Volume (Int)	Abs. Quant.	Rel. Quant.	Band %	Lane %
1		N/A	0,181	2 289 672	4 031 818	N/A	N/A	100,0	41,0

Band Detection	Automatically detected bands with sensitivity: Low
Lane Background	Lane background subtracted with disk size: 10
Lane Width	8.03 mm

Lane 6

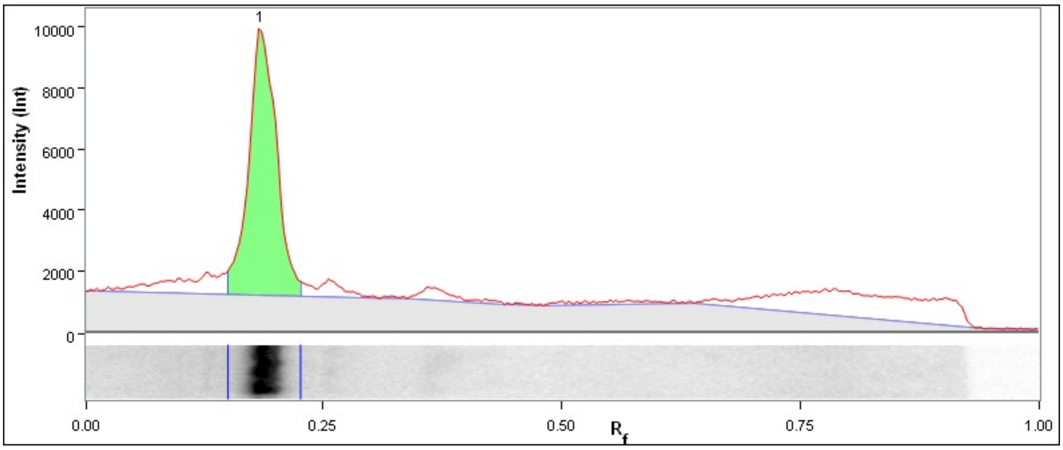

Band No.	Band Label	Mol. Wt. (KDa)	Relative Front	Adj. Volume (Int)	Volume (Int)	Abs. Quant.	Rel. Quant.	Band %	Lane %
1		N/A	0,187	5 780 928	7 622 736	N/A	N/A	100,0	51,8

Band Detection	Automatically detected bands with sensitivity: Low
Lane Background	Lane background subtracted with disk size: 10
Lane Width	7.86 mm

Lane 7

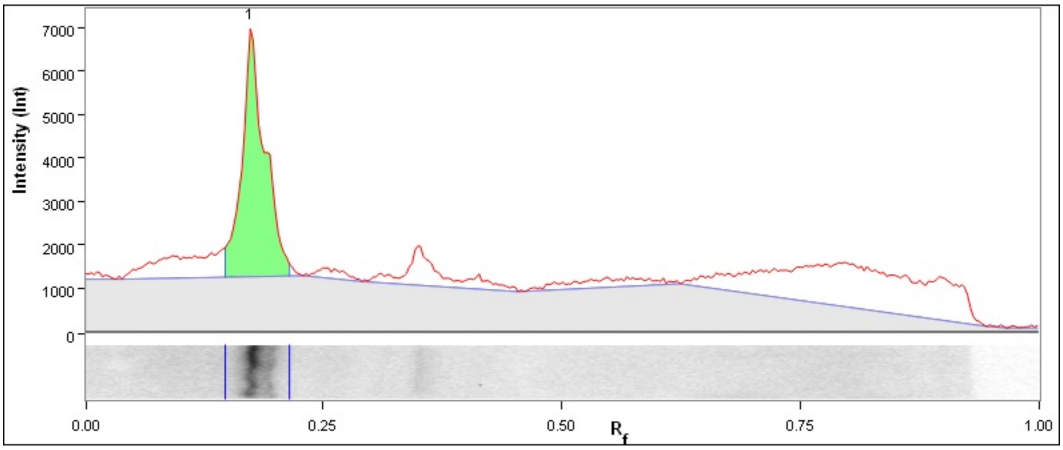

Band No.	Band Label	Mol. Wt. (KDa)	Relative Front	Adj. Volume (Int)	Volume (Int)	Abs. Quant.	Rel. Quant.	Band %	Lane %
1		N/A	0,175	2 903 664	4 519 536	N/A	N/A	100,0	32,6

Band Detection	Automatically detected bands with sensitivity: Low
Lane Background	Lane background subtracted with disk size: 10
Lane Width	7.86 mm

Volume Analysis

No.	Label	Type	Volume (Int)	Adj. Vol. (Int)	Mean Bkgd. (Int)	Abs. Quant.	Rel. Quant.	# of Pixels	Min. Value (Int)	Max. Value (Int)	Mean Value (Int)	Std. Dev.	Area (mm2)
1	U1	Unknown	9 189 964	548 933	2 972,5	N/A	N/A	2 907	1 532	7 496	3 161,3	939,0	78,0
2	U2	Unknown	9 570 436	1 339 926	2 831,3	N/A	N/A	2 907	1 472	8 572	3 292,2	1 208,6	78,0
3	U3	Unknown	13 281 660	5 296 078	2 747,0	N/A	N/A	2 907	1 464	17 592	4 568,9	3 449,1	78,0
4	U4	Unknown	7 795 720	1 068 341	2 314,2	N/A	N/A	2 907	692	9 208	2 681,7	1 373,9	78,0
5	U5	Unknown	7 406 872	743 605	2 292,1	N/A	N/A	2 907	676	10 504	2 547,9	1 439,6	78,0
6	U6	Unknown	10 896 880	3 831 919	2 430,3	N/A	N/A	2 907	812	15 152	3 748,5	3 109,7	78,0
7	U7	Unknown	7 491 528	1 916 008	1 918,0	N/A	N/A	2 907	484	10 092	2 577,1	1 656,1	78,0

Image Report: Histologia 2023-01-13 14hr 38min_Exposure_16.8sec pstat3 1c

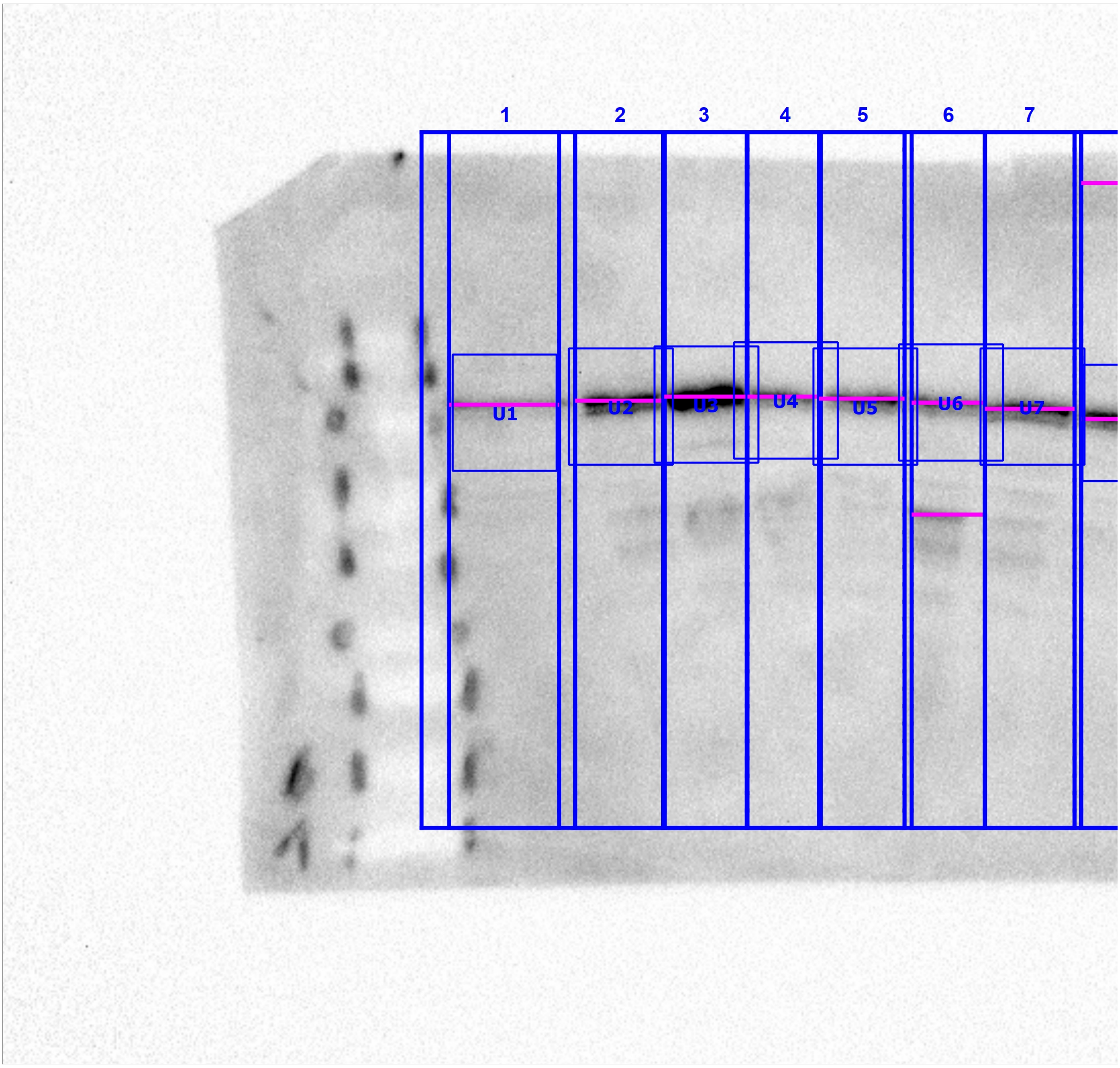

C:\Users\rusak\OneDrive\Dokumenty\Badania\CHI3L2 in BC\BC westerny ilościowo\p-STAT3 13.01.23\1\Histologia 2023-01-13 14hr 38min_Exposure_16.8sec pstat3 1c.scn

Acquisition Information

Imager	ChemiDoc MP
Exposure Time (sec)	16.800 (Signal Accumulation)
Flat Field	Applied (Lens)

Serial Number	731BR01769
Software Version	5.0
Application	Chemi Hi Resolution
Excitation Source	No Illumination
Emission Filter	No Filter
Binning	2x2

Image Information

Acquisition Date	13/1/2023 2:39:16 PM
User Name	Histologia
Image Area (mm)	X: 114.0 Y: 85.2
Pixel Size (μm)	X: 163.8 Y: 163.8
Data Range (Int)	0 - 19128

Analysis Settings

Detection	Lane detection:<br>Manually created lanes<br><br>Band detection:<br>Automatically detected bands with sensitivity: Low<br><br>Lane Background Subtraction:<br>Lane background subtracted with disk size: 10<br><br>Lane width: Variable
Volume Analysis	Background subtraction method: Local<br>Quantity regression method: Linear

Lane Statistics

Lane No.	Adj. Total Band Vol. (Int)	Total Band Vol. (Int)	Adj. Total Lane Vol. (Int)	Total Lane Vol. (Int)	Bkgd. Vol. (Int)	Norm. Factor
1	970 002	2 499 606	6 272 424	37 123 866	30 851 442	N/A
2	2 557 940	4 377 472	5 741 208	27 084 772	21 343 564	N/A
3	5 203 269	6 680 376	8 542 719	25 148 498	16 605 779	N/A
4	1 358 100	2 178 036	4 610 628	17 468 604	12 857 976	N/A
5	1 857 828	2 953 188	5 835 102	21 030 492	15 195 390	N/A
6	1 924 164	3 415 320	7 029 972	20 188 044	13 158 072	N/A
7	2 403 984	3 809 696	6 062 628	24 952 664	18 890 036	N/A
8	5 667 116	13 568 516	7 312 760	27 379 404	20 066 644	N/A

Lane And Band Analysis

Lane 1

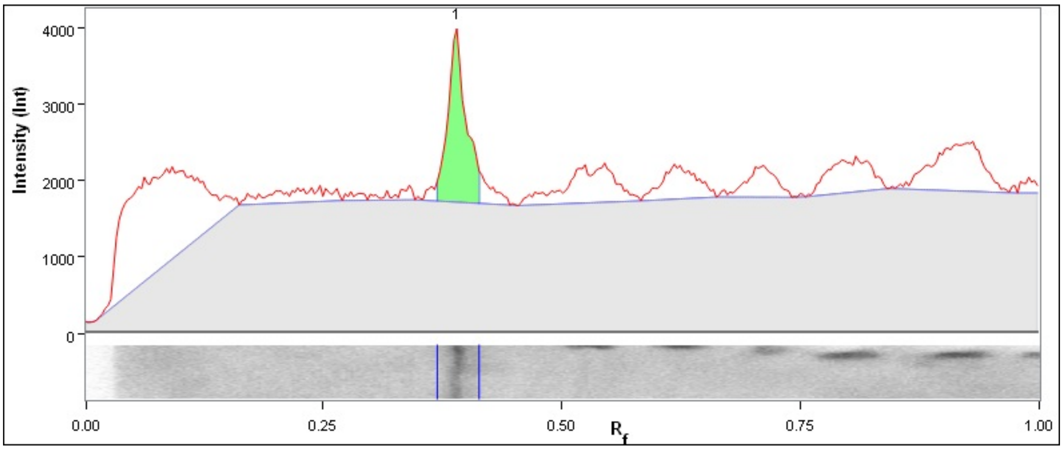

Band No.	Band Label	Mol. Wt. (KDa)	Relative Front	Adj. Volume (Int)	Volume (Int)	Abs. Quant.	Rel. Quant.	Band %	Lane %
1		N/A	0,392	970 002	2 499 606	N/A	N/A	100,0	15,5

Band Detection	Automatically detected bands with sensitivity: Low
Lane Background	Lane background subtracted with disk size: 10
Lane Width	8.84 mm

Lane 2

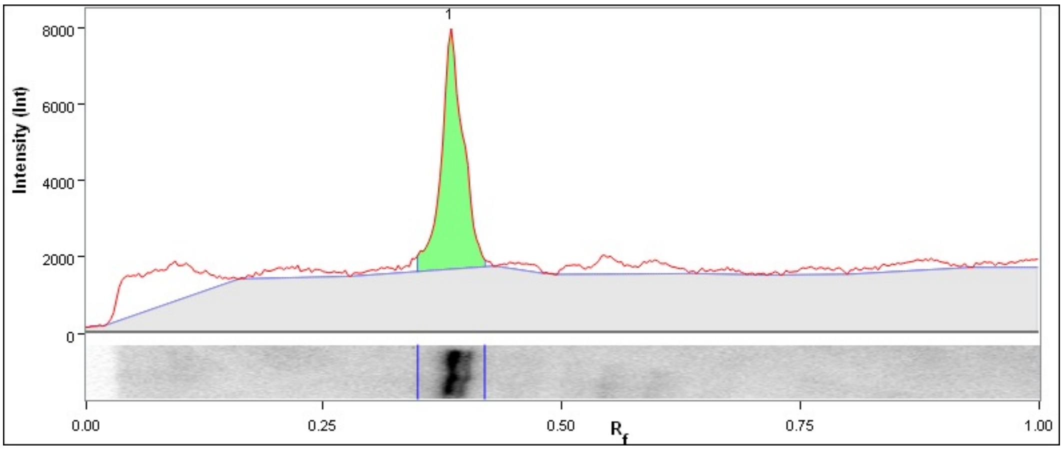

Band No.	Band Label	Mol. Wt. (KDa)	Relative Front	Adj. Volume (Int)	Volume (Int)	Abs. Quant.	Rel. Quant.	Band %	Lane %
1		N/A	0,386	2 557 940	4 377 472	N/A	N/A	100,0	44,6

Band Detection	Automatically detected bands with sensitivity: Low
Lane Background	Lane background subtracted with disk size: 10
Lane Width	7.21 mm

Lane 3

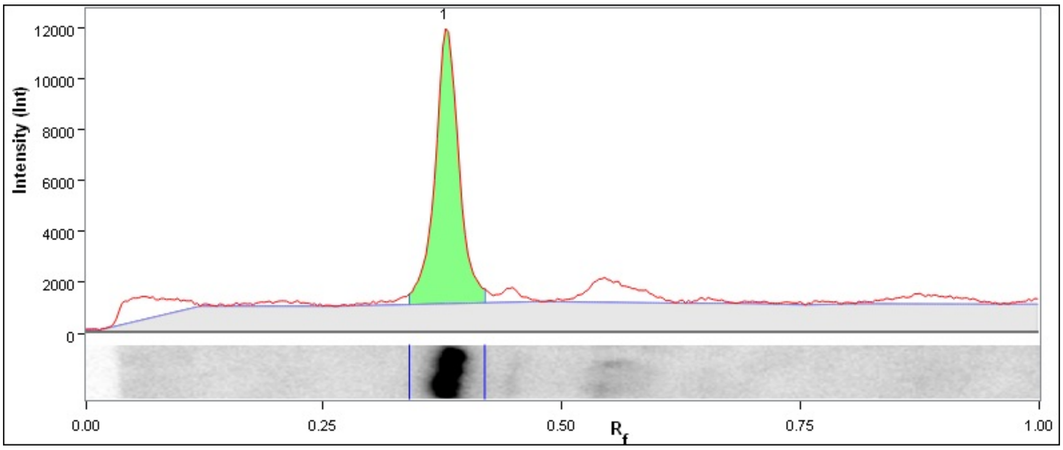

Band No.	Band Label	Mol. Wt. (KDa)	Relative Front	Adj. Volume (Int)	Volume (Int)	Abs. Quant.	Rel. Quant.	Band %	Lane %
1		N/A	0,380	5 203 269	6 680 376	N/A	N/A	100,0	60,9

Band Detection	Automatically detected bands with sensitivity: Low
Lane Background	Lane background subtracted with disk size: 10
Lane Width	6.72 mm

Lane 4

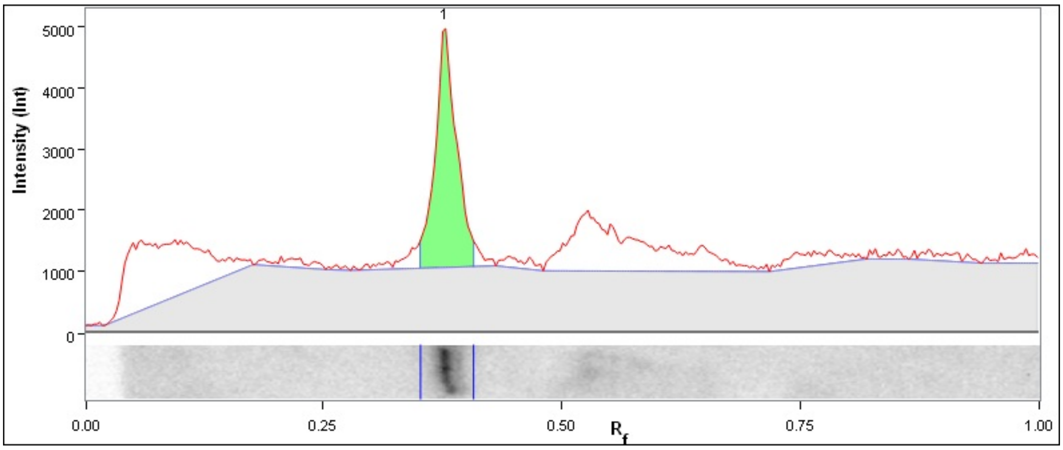

Band No.	Band Label	Mol. Wt. (KDa)	Relative Front	Adj. Volume (Int)	Volume (Int)	Abs. Quant.	Rel. Quant.	Band %	Lane %
1		N/A	0,380	1 358 100	2 178 036	N/A	N/A	100,0	29,5

Band Detection	Automatically detected bands with sensitivity: Low
Lane Background	Lane background subtracted with disk size: 10
Lane Width	5.90 mm

Lane 5

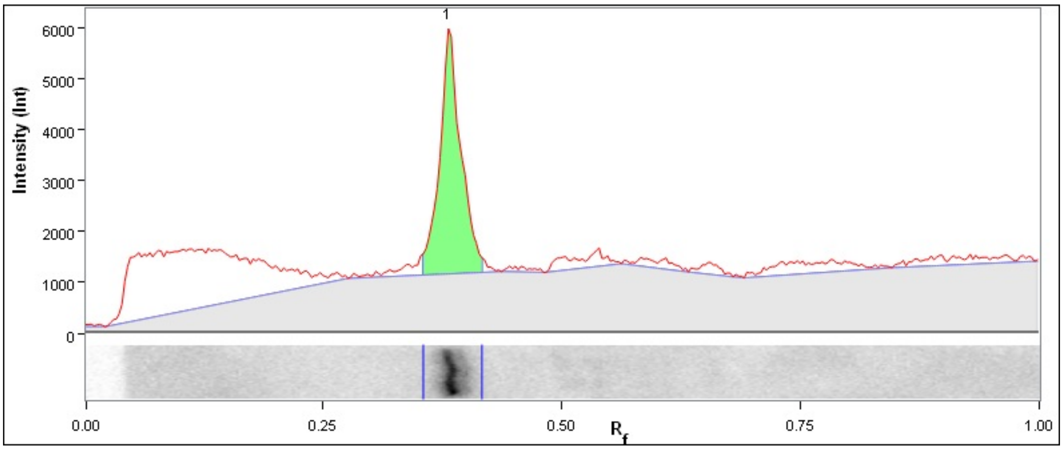

Band No.	Band Label	Mol. Wt. (KDa)	Relative Front	Adj. Volume (Int)	Volume (Int)	Abs. Quant.	Rel. Quant.	Band %	Lane %
1		N/A	0,383	1 857 828	2 953 188	N/A	N/A	100,0	31,8

Band Detection	Automatically detected bands with sensitivity: Low
Lane Background	Lane background subtracted with disk size: 10
Lane Width	6.88 mm

Lane 6

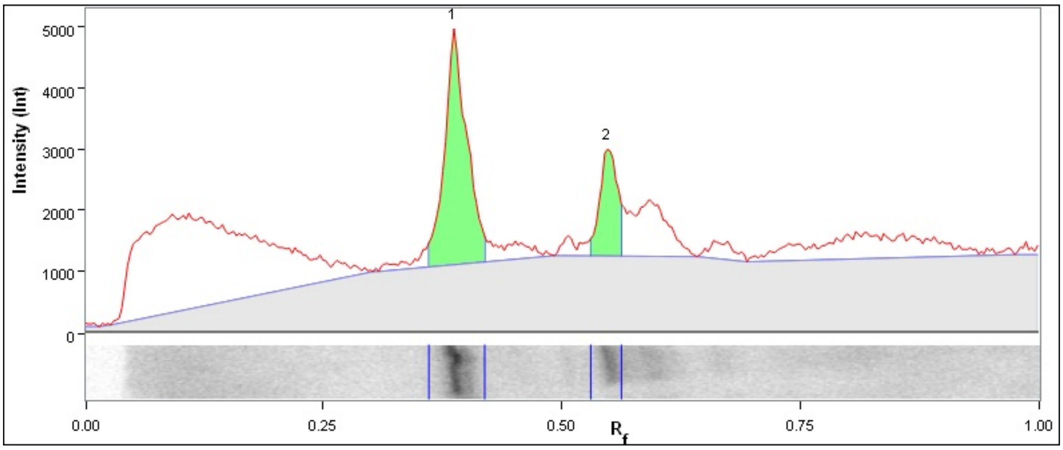

Band No.	Band Label	Mol. Wt. (KDa)	Relative Front	Adj. Volume (Int)	Volume (Int)	Abs. Quant.	Rel. Quant.	Band %	Lane %
1		N/A	0,389	1 409 652	2 317 104	N/A	N/A	73,3	20,1
2		N/A	0,550	514 512	1 098 216	N/A	N/A	26,7	7,3

Band Detection	Automatically detected bands with sensitivity: Low
Lane Background	Lane background subtracted with disk size: 10
Lane Width	5.90 mm

Lane 7

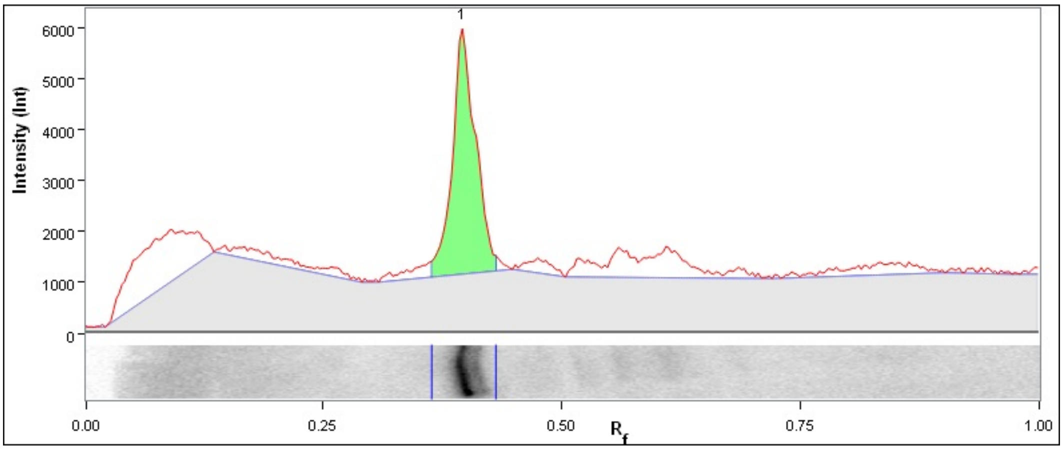

Band No.	Band Label	Mol. Wt. (KDa)	Relative Front	Adj. Volume (Int)	Volume (Int)	Abs. Quant.	Rel. Quant.	Band %	Lane %
1		N/A	0,398	2 403 984	3 809 696	N/A	N/A	100,0	39,7

Band Detection	Automatically detected bands with sensitivity: Low
Lane Background	Lane background subtracted with disk size: 10
Lane Width	7.21 mm

Lane 8

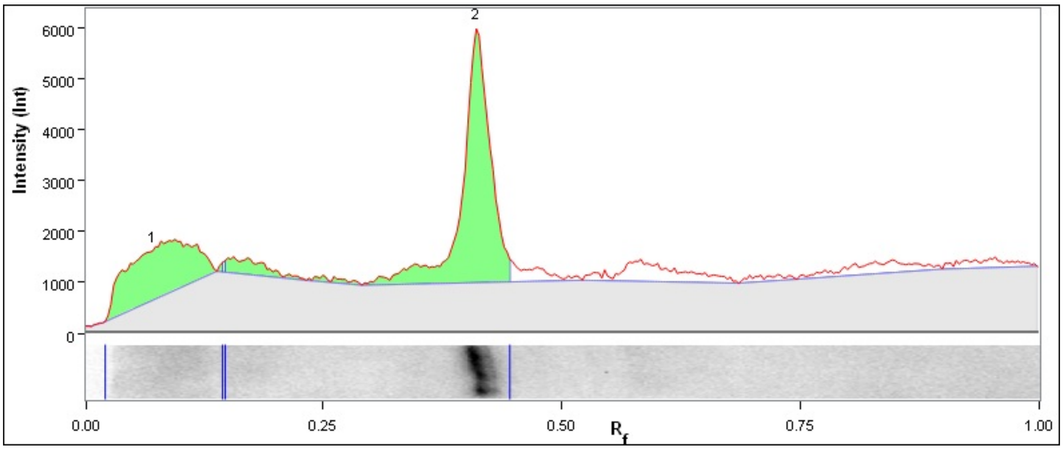

Band No.	Band Label	Mol. Wt. (KDa)	Relative Front	Adj. Volume (Int)	Volume (Int)	Abs. Quant.	Rel. Quant.	Band %	Lane %
1		N/A	0,073	1 807 780	3 636 724	N/A	N/A	31,9	24,7
2		N/A	0,412	3 859 336	9 931 792	N/A	N/A	68,1	52,8

Band Detection	Automatically detected bands with sensitivity: Low
Lane Background	Lane background subtracted with disk size: 10
Lane Width	8.52 mm

Volume Analysis

No.	Label	Type	Volume (Int)	Adj. Vol. (Int)	Mean Bkgd. (Int)	Abs. Quant.	Rel. Quant.	# of Pixels	Min. Value (Int)	Max. Value (Int)	Mean Value (Int)	Std. Dev.	Area (mm2)
1	U1	Unknown	6 291 952	304 378	2 059,7	N/A	N/A	2 907	864	5 656	2 164,4	668,1	78,0
2	U2	Unknown	7 841 404	1 419 471	2 209,1	N/A	N/A	2 907	804	13 608	2 697,4	1 851,1	78,0

3	U3	Unknown	9 890 016	4 386 484	1 893,2	N/A	N/A	2 907	544	19 128	3 402,1	3 526,9	78,0
4	U4	Unknown	5 889 496	-85 605	2 055,4	N/A	N/A	2 907	472	15 584	2 026,0	1 541,2	78,0
5	U5	Unknown	5 767 260	1 182 868	1 577,0	N/A	N/A	2 907	420	9 040	1 983,9	1 333,4	78,0
6	U6	Unknown	5 818 732	687 666	1 765,1	N/A	N/A	2 907	376	7 796	2 001,6	1 167,6	78,0
7	U7	Unknown	6 718 740	1 719 440	1 719,7	N/A	N/A	2 907	496	9 904	2 311,2	1 567,2	78,0
8	U8	Unknown	6 770 580	2 266 157	1 549,5	N/A	N/A	2 907	392	9 564	2 329,1	1 699,0	78,0

Image Report: Histologia 2023-01-19 14hr 30min_Exposure_16.8sec pstat3 3c

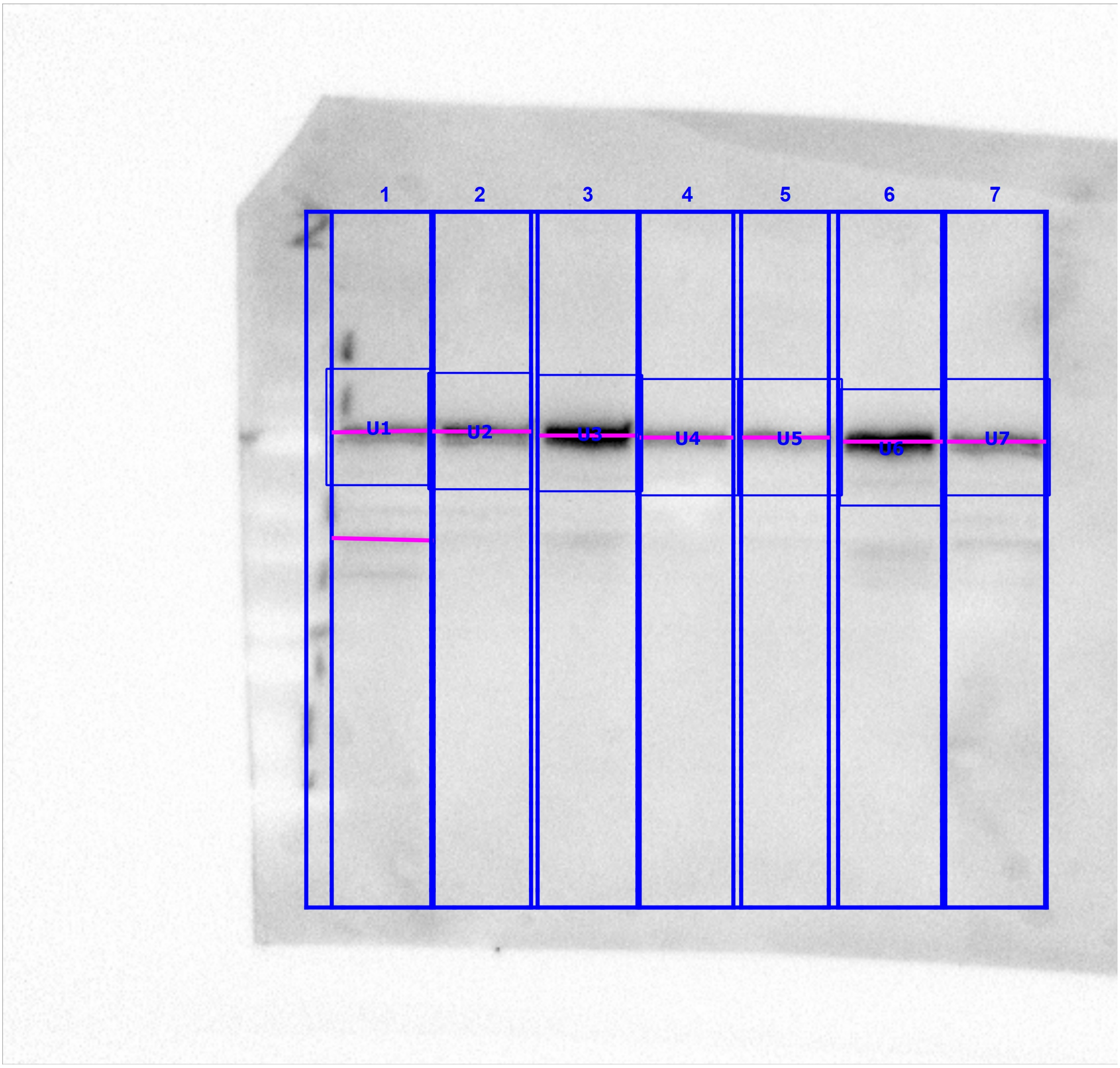

C:\Users\rusak\OneDrive\Dokumenty\Badania\CHI3L2 in BC\BC westerny ilościowo\pSTAT3 19.1.23\3\Histologia 2023-01-19 14hr 30min_Exposure_16.8sec pstat3 3c.scn

Acquisition Information

Imager	ChemiDoc MP
Exposure Time (sec)	16.800 (Signal Accumulation)
Flat Field	Applied (Lens)

Serial Number	731BR01769
Software Version	5.0
Application	Chemi Hi Resolution
Excitation Source	No Illumination
Emission Filter	No Filter
Binning	2x2

Image Information

Acquisition Date	19/1/2023 2:30:47 PM
User Name	Histologia
Image Area (mm)	X: 114.0 Y: 85.2
Pixel Size (µm)	X: 163.8 Y: 163.8
Data Range (Int)	0 - 24868

Analysis Settings

Detection	Lane detection:<br>Manually created lanes<br><br>Band detection:<br>Automatically detected bands with sensitivity: Low<br><br>Lane Background Subtraction:<br>Lane background subtracted with disk size: 10<br><br>Lane width: Variable
Volume Analysis	Background subtraction method: Local<br>Quantity regression method: Linear

Lane Statistics

Lane No.	Adj. Total Band Vol. (Int)	Total Band Vol. (Int)	Adj. Total Lane Vol. (Int)	Total Lane Vol. (Int)	Bkgd. Vol. (Int)	Norm. Factor
1	3 629 150	7 647 500	7 874 150	52 079 100	44 204 950	N/A
2	4 353 601	7 218 582	8 532 370	44 343 236	35 810 866	N/A
3	9 073 526	12 631 563	12 186 986	48 690 026	36 503 040	N/A
4	2 989 770	4 606 716	5 973 008	34 952 686	28 979 678	N/A
5	2 265 111	3 978 360	4 512 893	32 931 593	28 418 700	N/A
6	8 055 246	11 005 800	11 960 622	46 145 922	34 185 300	N/A
7	3 782 898	6 318 942	9 078 181	44 633 316	35 555 135	N/A

Lane And Band Analysis

Lane 1

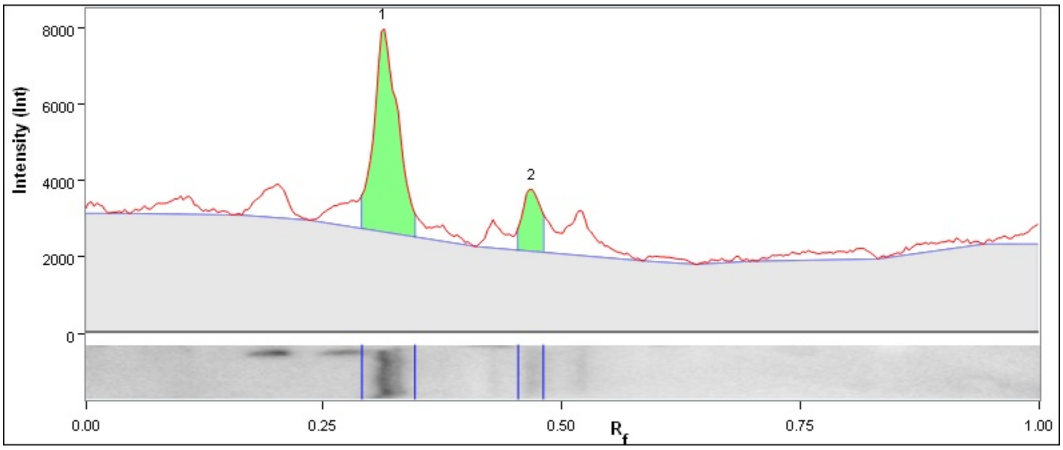

Band No.	Band Label	Mol. Wt. (KDa)	Relative Front	Adj. Volume (Int)	Volume (Int)	Abs. Quant.	Rel. Quant.	Band %	Lane %
1		N/A	0,316	2 951 500	5 808 600	N/A	N/A	81,3	37,5
2		N/A	0,471	677 650	1 838 900	N/A	N/A	18,7	8,6

Band Detection	Automatically detected bands with sensitivity: Low
Lane Background	Lane background subtracted with disk size: 10
Lane Width	8.19 mm

Lane 2

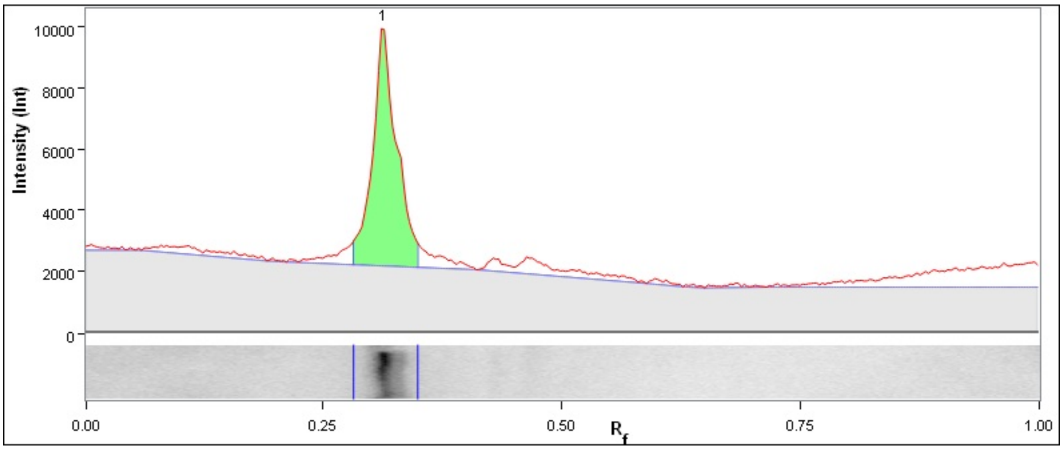

Band No.	Band Label	Mol. Wt. (KDa)	Relative Front	Adj. Volume (Int)	Volume (Int)	Abs. Quant.	Rel. Quant.	Band %	Lane %
1		N/A	0,316	4 353 601	7 218 582	N/A	N/A	100,0	51,0

Band Detection	Automatically detected bands with sensitivity: Low
Lane Background	Lane background subtracted with disk size: 10
Lane Width	8.03 mm

Lane 3

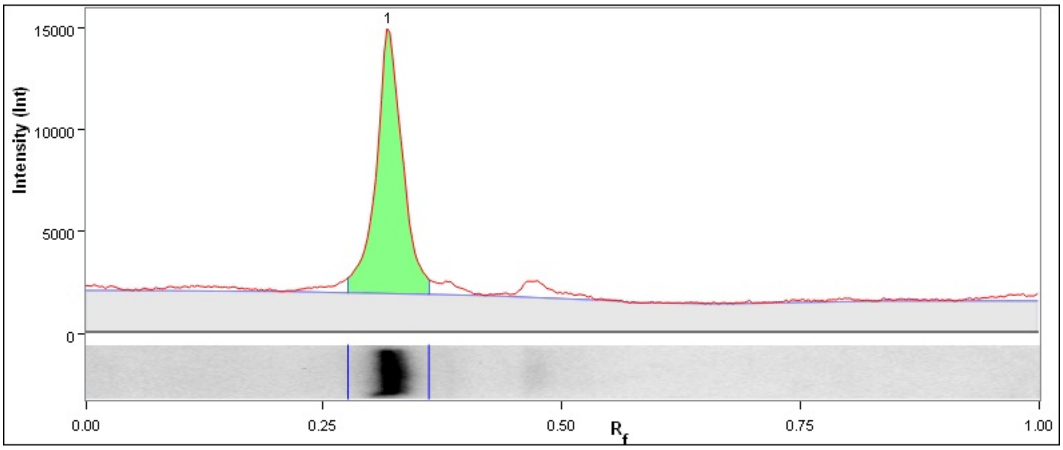

Band No.	Band Label	Mol. Wt. (KDa)	Relative Front	Adj. Volume (Int)	Volume (Int)	Abs. Quant.	Rel. Quant.	Band %	Lane %
1		N/A	0,322	9 073 526	12 631 563	N/A	N/A	100,0	74,5

Band Detection	Automatically detected bands with sensitivity: Low
Lane Background	Lane background subtracted with disk size: 10
Lane Width	8.03 mm

Lane 4

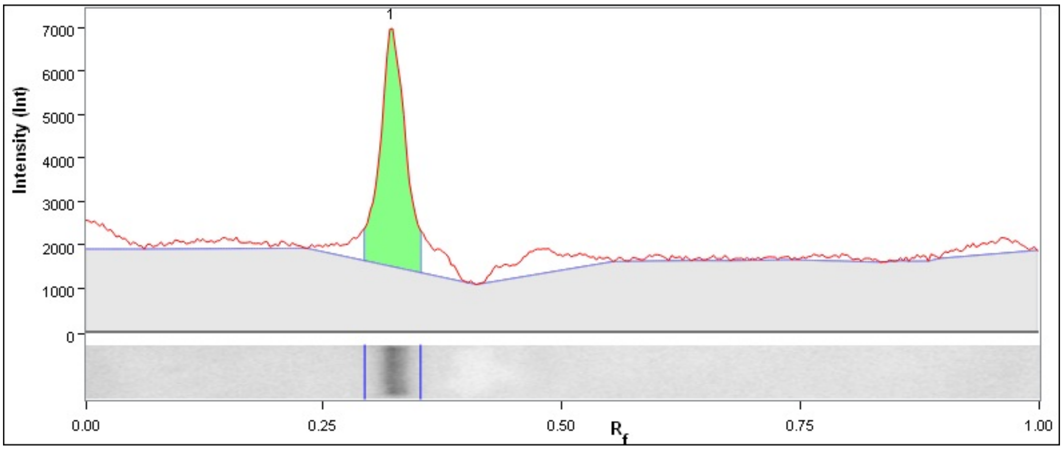

Band No.	Band Label	Mol. Wt. (KDa)	Relative Front	Adj. Volume (Int)	Volume (Int)	Abs. Quant.	Rel. Quant.	Band %	Lane %
1		N/A	0,325	2 989 770	4 606 716	N/A	N/A	100,0	50,1

Band Detection	Automatically detected bands with sensitivity: Low
Lane Background	Lane background subtracted with disk size: 10
Lane Width	7.53 mm

Lane 5

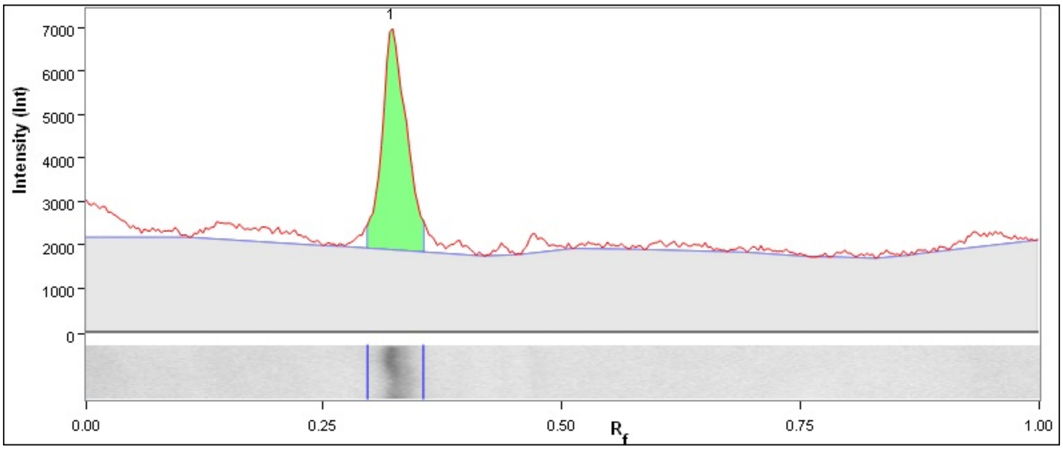

Band No.	Band Label	Mol. Wt. (KDa)	Relative Front	Adj. Volume (Int)	Volume (Int)	Abs. Quant.	Rel. Quant.	Band %	Lane %
1		N/A	0,325	2 265 111	3 978 360	N/A	N/A	100,0	50,2

Band Detection	Automatically detected bands with sensitivity: Low
Lane Background	Lane background subtracted with disk size: 10
Lane Width	7.04 mm

Lane 6

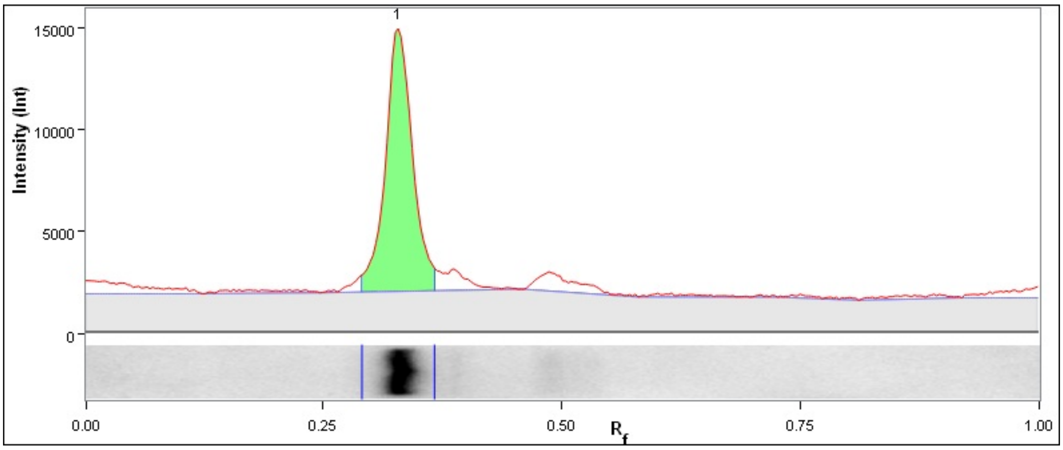

Band No.	Band Label	Mol. Wt. (KDa)	Relative Front	Adj. Volume (Int)	Volume (Int)	Abs. Quant.	Rel. Quant.	Band %	Lane %
1		N/A	0,330	8 055 246	11 005 800	N/A	N/A	100,0	67,3

Band Detection	Automatically detected bands with sensitivity: Low
Lane Background	Lane background subtracted with disk size: 10
Lane Width	8.35 mm

Lane 7

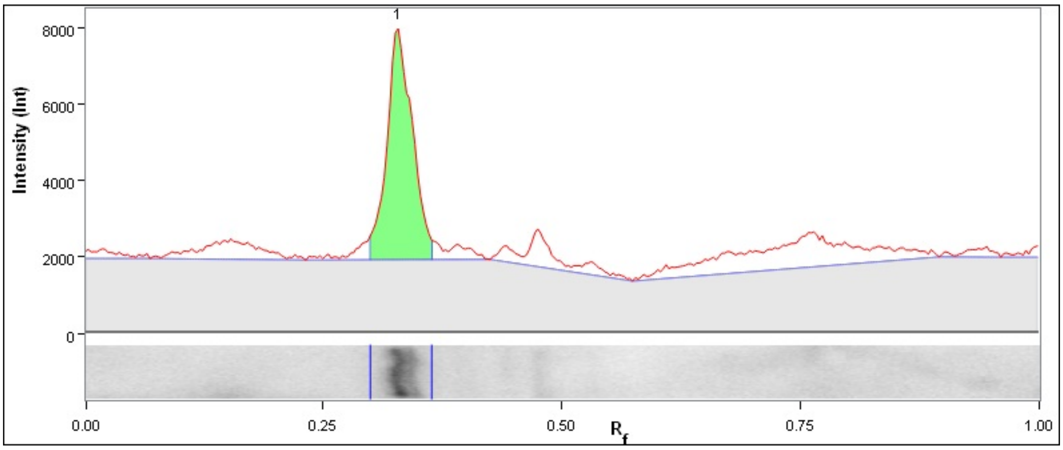

Band No.	Band Label	Mol. Wt. (KDa)	Relative Front	Adj. Volume (Int)	Volume (Int)	Abs. Quant.	Rel. Quant.	Band %	Lane %
1		N/A	0,330	3 782 898	6 318 942	N/A	N/A	100,0	41,7

Band Detection	Automatically detected bands with sensitivity: Low
Lane Background	Lane background subtracted with disk size: 10
Lane Width	8.03 mm

Volume Analysis

No.	Label	Type	Volume (Int)	Adj. Vol. (Int)	Mean Bkgd. (Int)	Abs. Quant.	Rel. Quant.	# of Pixels	Min. Value (Int)	Max. Value (Int)	Mean Value (Int)	Std. Dev.	Area (mm2)
1	U1	Unknown	12 052 068	2 831 540	3 171,8	N/A	N/A	2 907	1 756	11 636	4 145,9	1 792,8	78,0
2	U2	Unknown	12 200 720	2 880 508	3 206,1	N/A	N/A	2 907	1 864	16 692	4 197,0	2 422,0	78,0
3	U3	Unknown	16 903 128	7 935 244	3 084,9	N/A	N/A	2 907	1 576	24 868	5 814,6	5 017,3	78,0
4	U4	Unknown	8 813 764	1 698 062	2 447,8	N/A	N/A	2 907	676	9 452	3 031,9	1 721,5	78,0
5	U5	Unknown	8 443 084	1 474 371	2 397,2	N/A	N/A	2 907	1 164	8 720	2 904,4	1 464,9	78,0
6	U6	Unknown	15 150 544	7 795 041	2 530,3	N/A	N/A	2 907	1 308	20 088	5 211,7	4 420,7	78,0
7	U7	Unknown	10 889 312	3 061 501	2 692,7	N/A	N/A	2 907	1 444	12 620	3 745,9	2 127,9	78,0

Image Report: Histologia 2023-01-13 14hr 41min_Exposure_16.8sec pstat3 2b

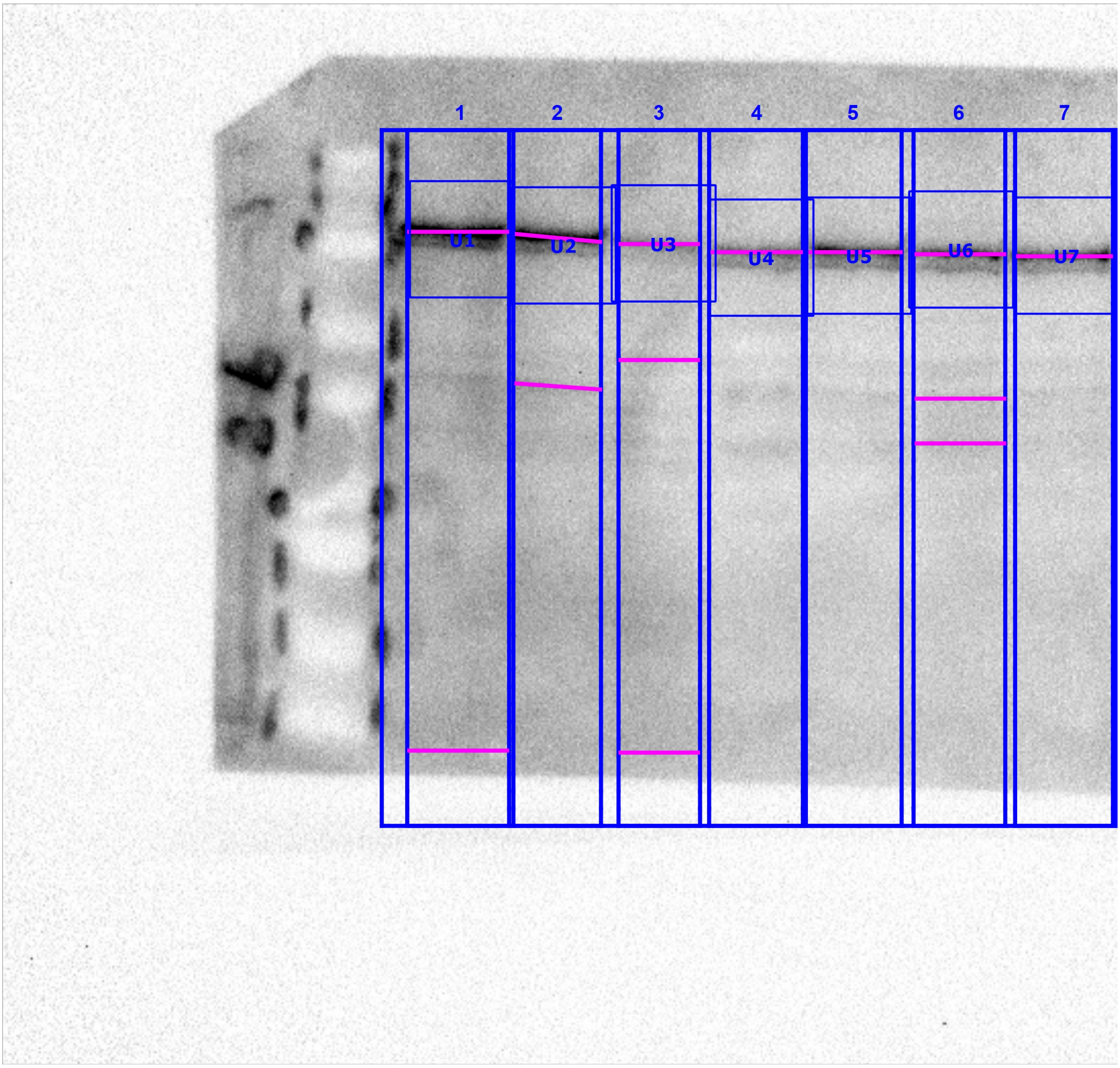

C:\Users\rusak\OneDrive\Dokumenty\Badania\CHI3L2 in BC\BC westerny ilościowo\p-STAT3 13.01.23\2\Histologia 2023-01-13 14hr 41min_Exposure_16.8sec pstat3 2b.scn

Acquisition Information

Imager	ChemiDoc MP
Exposure Time (sec)	16.800 (Signal Accumulation)
Flat Field	Applied (Lens)

Serial Number	731BR01769
Software Version	5.0
Application	Chemi Hi Resolution
Excitation Source	No Illumination
Emission Filter	No Filter
Binning	2x2

Image Information

Acquisition Date	13/1/2023 2:41:50 PM
User Name	Histologia
Image Area (mm)	X: 114.0 Y: 85.2
Pixel Size (μm)	X: 163.8 Y: 163.8
Data Range (Int)	0 - 9528

Analysis Settings

Detection	Lane detection:<br>Manually created lanes<br><br>Band detection:<br>Automatically detected bands with sensitivity: Low<br><br>Lane Background Subtraction:<br>Lane background subtracted with disk size: 10<br><br>Lane width: Variable
Volume Analysis	Background subtraction method: Local<br>Quantity regression method: Linear

Lane Statistics

Lane No.	Adj. Total Band Vol. (Int)	Total Band Vol. (Int)	Adj. Total Lane Vol. (Int)	Total Lane Vol. (Int)	Bkgd. Vol. (Int)	Norm. Factor
1	4 464 900	8 855 550	7 202 800	33 928 700	26 725 900	N/A
2	2 021 903	4 649 547	5 471 234	25 790 196	20 318 962	N/A
3	2 984 760	9 847 240	3 924 960	20 368 840	16 443 880	N/A
4	1 385 060	2 458 056	4 822 226	18 809 354	13 987 128	N/A
5	1 927 000	3 158 494	4 861 492	18 861 100	13 999 608	N/A
6	2 197 080	5 653 890	5 175 990	19 422 900	14 246 910	N/A
7	1 610 448	2 819 520	4 401 840	19 142 304	14 740 464	N/A

Lane And Band Analysis

Lane 1

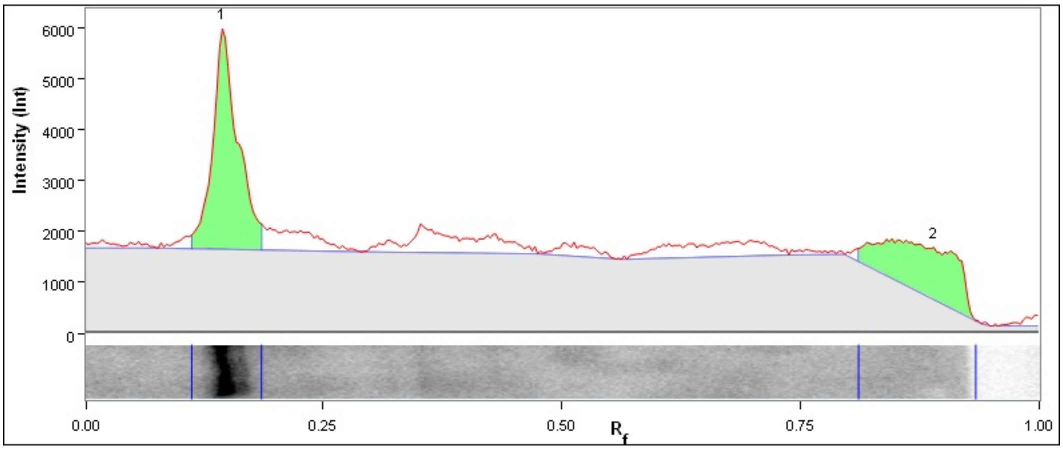

Band No.	Band Label	Mol. Wt. (KDa)	Relative Front	Adj. Volume (Int)	Volume (Int)	Abs. Quant.	Rel. Quant.	Band %	Lane %
1		N/A	0,146	2 622 200	5 044 300	N/A	N/A	58,7	36,4
2		N/A	0,892	1 842 700	3 811 250	N/A	N/A	41,3	25,6

Band Detection	Automatically detected bands with sensitivity: Low
Lane Background	Lane background subtracted with disk size: 10
Lane Width	8.19 mm

Lane 2

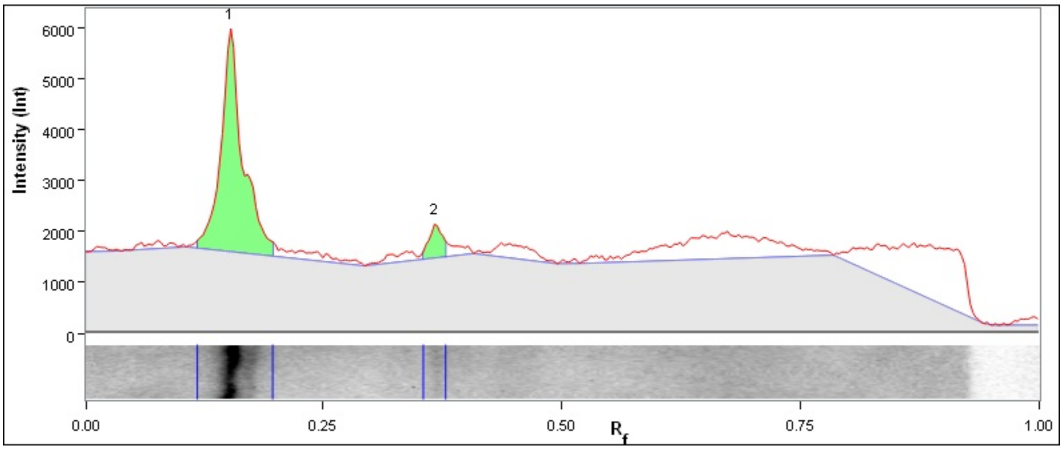

Band No.	Band Label	Mol. Wt. (KDa)	Relative Front	Adj. Volume (Int)	Volume (Int)	Abs. Quant.	Rel. Quant.	Band %	Lane %
1		N/A	0,155	1 847 237	3 872 064	N/A	N/A	91,4	33,8
2		N/A	0,368	174 666	777 483	N/A	N/A	8,6	3,2

Band Detection	Automatically detected bands with sensitivity: Low
Lane Background	Lane background subtracted with disk size: 10
Lane Width	7.04 mm

Lane 3

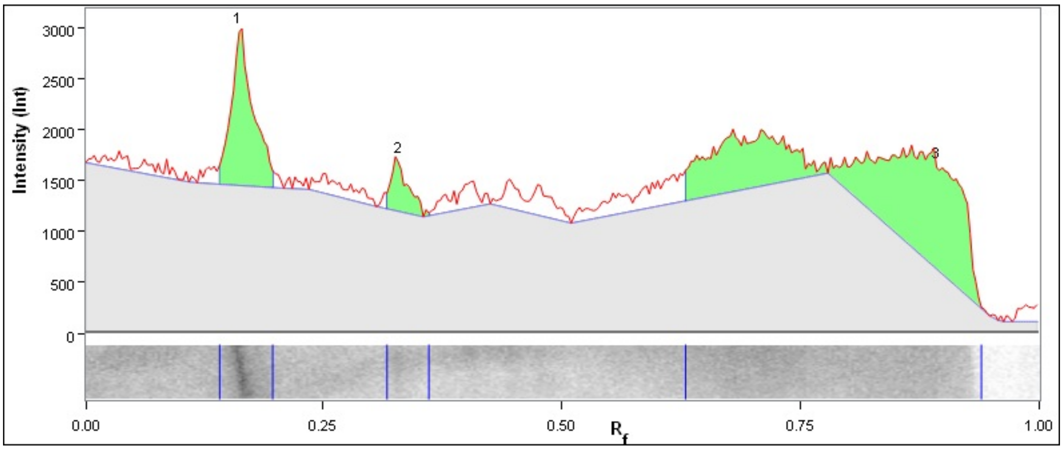

Band No.	Band Label	Mol. Wt. (KDa)	Relative Front	Adj. Volume (Int)	Volume (Int)	Abs. Quant.	Rel. Quant.	Band %	Lane %
1		N/A	0,164	572 840	1 731 640	N/A	N/A	19,2	14,6
2		N/A	0,330	154 640	908 200	N/A	N/A	5,2	3,9
3		N/A	0,895	2 257 280	7 207 400	N/A	N/A	75,6	57,5

Band Detection	Automatically detected bands with sensitivity: Low
Lane Background	Lane background subtracted with disk size: 10
Lane Width	6.55 mm

Lane 4

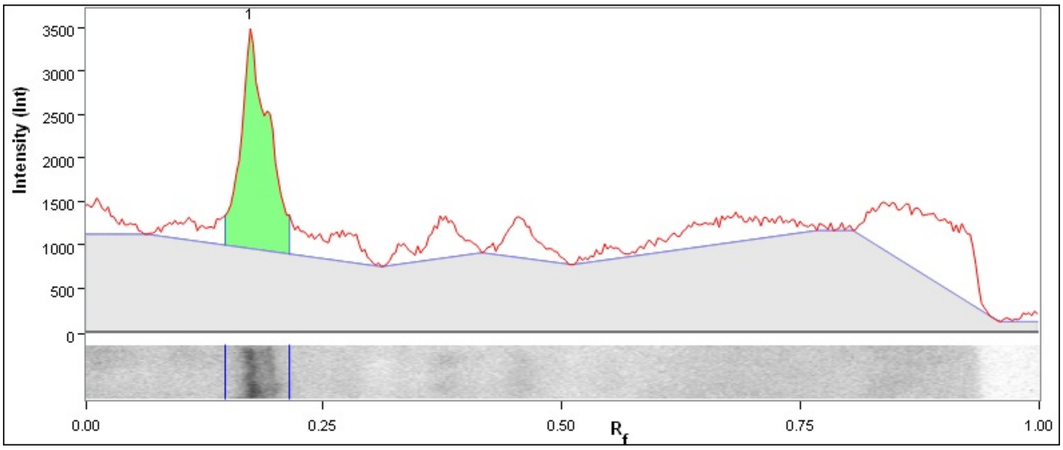

Band No.	Band Label	Mol. Wt. (KDa)	Relative Front	Adj. Volume (Int)	Volume (Int)	Abs. Quant.	Rel. Quant.	Band %	Lane %
1		N/A	0,175	1 385 060	2 458 056	N/A	N/A	100,0	28,7

Band Detection	Automatically detected bands with sensitivity: Low
Lane Background	Lane background subtracted with disk size: 10
Lane Width	7.53 mm

Lane 5

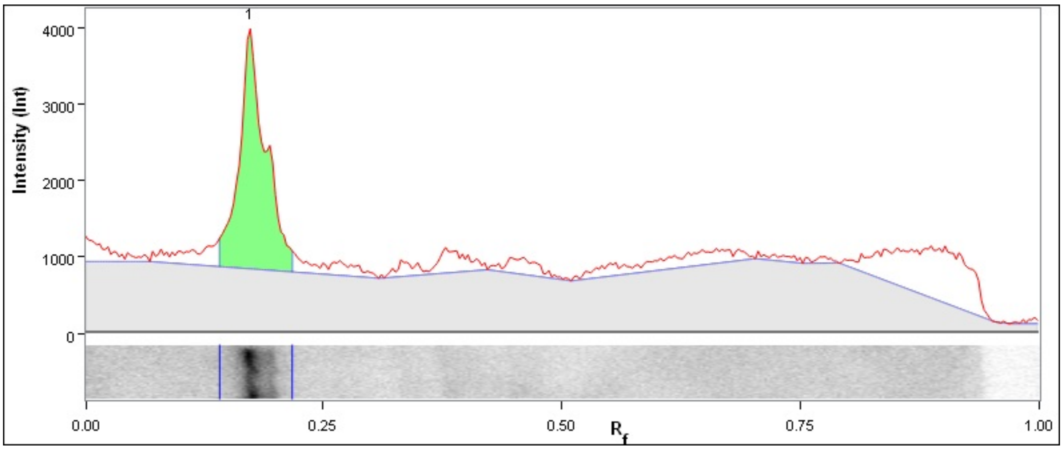

Band No.	Band Label	Mol. Wt. (KDa)	Relative Front	Adj. Volume (Int)	Volume (Int)	Abs. Quant.	Rel. Quant.	Band %	Lane %
1		N/A	0,175	1 927 000	3 158 494	N/A	N/A	100,0	39,6

Band Detection	Automatically detected bands with sensitivity: Low
Lane Background	Lane background subtracted with disk size: 10
Lane Width	7.70 mm

Lane 6

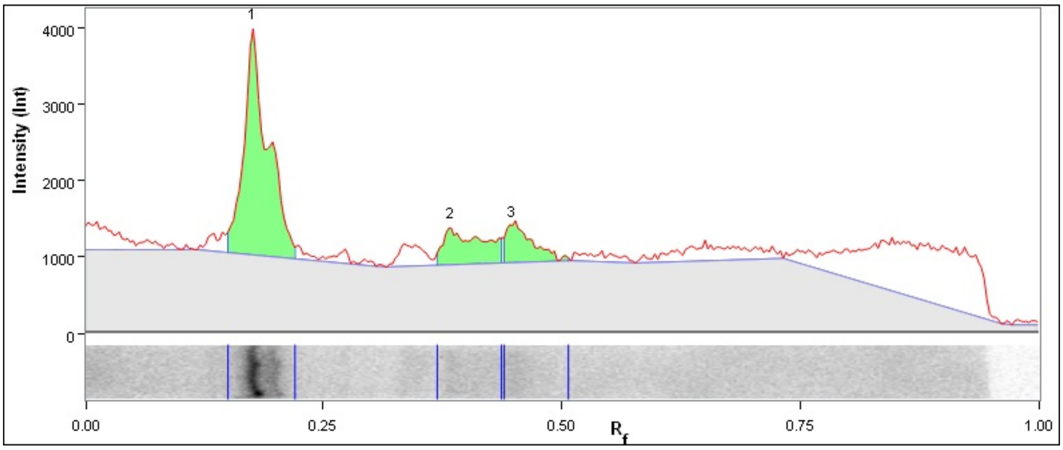

Band No.	Band Label	Mol. Wt. (KDa)	Relative Front	Adj. Volume (Int)	Volume (Int)	Abs. Quant.	Rel. Quant.	Band %	Lane %
1		N/A	0,178	1 503 945	2 767 275	N/A	N/A	68,5	29,1
2		N/A	0,386	395 055	1 473 525	N/A	N/A	18,0	7,6
3		N/A	0,450	298 080	1 413 090	N/A	N/A	13,6	5,8

Band Detection	Automatically detected bands with sensitivity: Low
Lane Background	Lane background subtracted with disk size: 10
Lane Width	7.37 mm

Lane 7

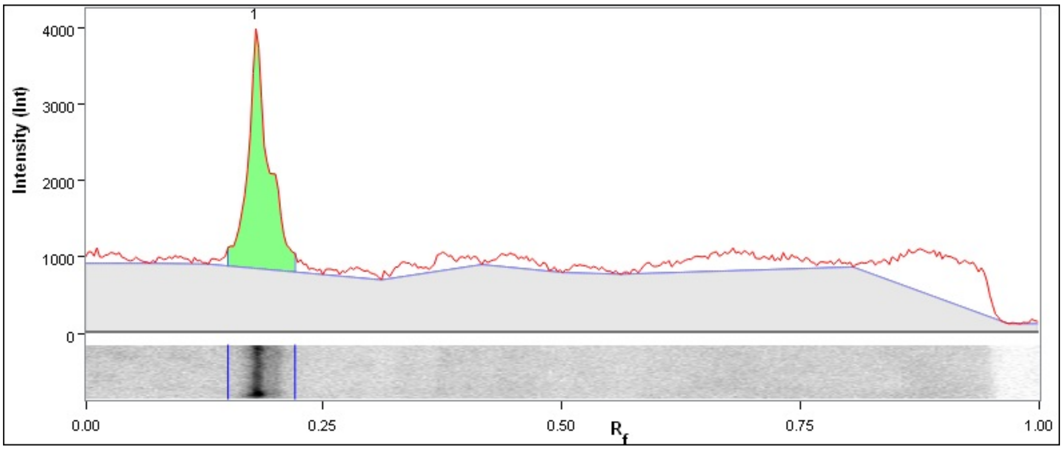

Band No.	Band Label	Mol. Wt. (KDa)	Relative Front	Adj. Volume (Int)	Volume (Int)	Abs. Quant.	Rel. Quant.	Band %	Lane %
1		N/A	0,181	1 610 448	2 819 520	N/A	N/A	100,0	36,6

Band Detection	Automatically detected bands with sensitivity: Low
Lane Background	Lane background subtracted with disk size: 10
Lane Width	7.86 mm

Volume Analysis

No.	Label	Type	Volume (Int)	Adj. Vol. (Int)	Mean Bkgd. (Int)	Abs. Quant.	Rel. Quant.	# of Pixels	Min. Value (Int)	Max. Value (Int)	Mean Value (Int)	Std. Dev.	Area (mm2)
1	U1	Unknown	8 514 096	1 057 852	2 564,9	N/A	N/A	2 907	728	9 528	2 928,8	1 369,9	78,0
2	U2	Unknown	6 956 488	1 133 186	2 003,2	N/A	N/A	2 907	592	8 760	2 393,0	1 235,6	78,0
3	U3	Unknown	5 019 812	294 721	1 625,4	N/A	N/A	2 907	312	4 292	1 726,8	507,9	78,0
4	U4	Unknown	4 755 252	647 925	1 412,9	N/A	N/A	2 907	356	4 484	1 635,8	737,1	78,0
5	U5	Unknown	5 148 812	1 342 016	1 309,5	N/A	N/A	2 907	332	6 352	1 771,2	1 013,4	78,0
6	U6	Unknown	5 075 228	1 058 653	1 381,7	N/A	N/A	2 907	348	6 068	1 745,9	904,2	78,0
7	U7	Unknown	4 718 920	1 022 009	1 271,7	N/A	N/A	2 907	208	7 248	1 623,3	980,6	78,0

Image Report: Histologia 2023-01-12 14hr 11min_Exposure_16.8sec pstat3 1b

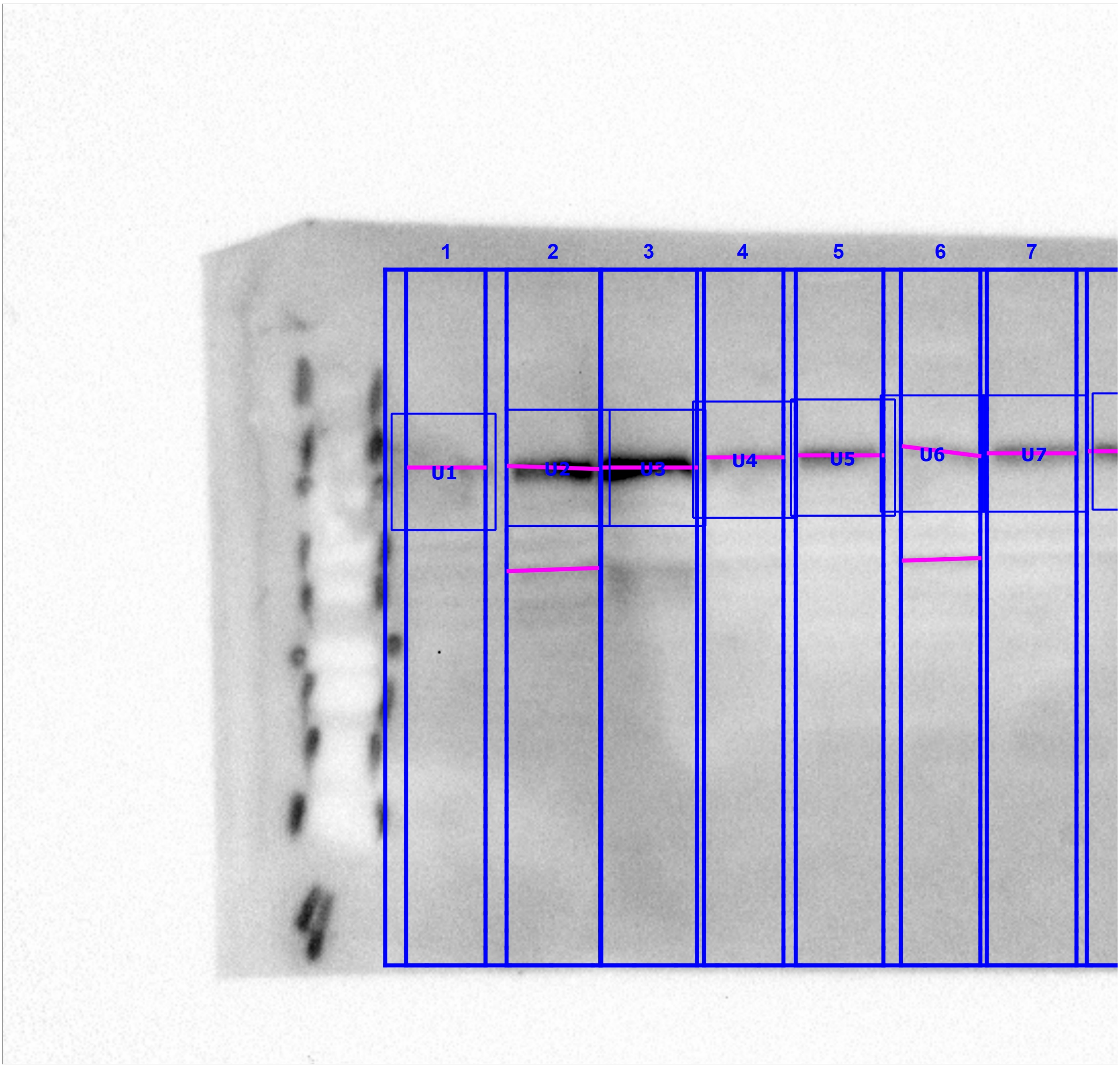

C:\Users\rusak\OneDrive\Dokumenty\Badania\CHI3L2 in BC\BC westerny ilościowo\pSTAT 3 BC 12.1.23\pSTAT3 12.1.23 BC 1 bis\Histologia 2023-01-12 14hr 11min_Exposure_16.8sec pstat3 1b.scn

Acquisition Information

Imager	ChemiDoc MP
Exposure Time (sec)	16.800 (Signal Accumulation)

Flat Field	Applied (Lens)
Serial Number	731BR01769
Software Version	5.0
Application	Chemi Hi Resolution
Excitation Source	No Illumination
Emission Filter	No Filter
Binning	2x2

Image Information

Acquisition Date	12/1/2023 2:12:00 PM
User Name	Histologia
Image Area (mm)	X: 114.0 Y: 85.2
Pixel Size (µm)	X: 163.8 Y: 163.8
Data Range (Int)	0 - 35116

Analysis Settings

Detection	Lane detection:<br>Manually created lanes<br><br>Band detection:<br>Automatically detected bands with sensitivity: Low<br>Manually adjusted bands<br><br>Lane Background Subtraction:<br>Lane background subtracted with disk size: 10<br><br>Lane width: Variable
Volume Analysis	Background subtraction method: Local<br>Quantity regression method: Linear

Lane Statistics

Lane No.	Adj. Total Band Vol. (Int)	Total Band Vol. (Int)	Adj. Total Lane Vol. (Int)	Total Lane Vol. (Int)	Bkgd. Vol. (Int)	Norm. Factor
1	1 607 034	6 207 396	5 911 620	46 998 276	41 086 656	N/A
2	5 423 492	12 401 784	11 123 904	68 766 090	57 642 186	N/A
3	7 886 036	12 368 661	12 064 806	71 924 382	59 859 576	N/A
4	1 562 691	3 268 590	5 300 490	42 426 813	37 126 323	N/A
5	2 404 646	4 506 572	5 509 031	45 422 104	39 913 073	N/A
6	1 323 231	3 379 740	4 474 392	41 263 443	36 789 051	N/A
7	1 927 772	4 199 096	5 070 164	47 967 128	42 896 964	N/A
8	2 341 155	4 978 416	5 186 700	56 335 620	51 148 920	N/A

Lane And Band Analysis

Lane 1

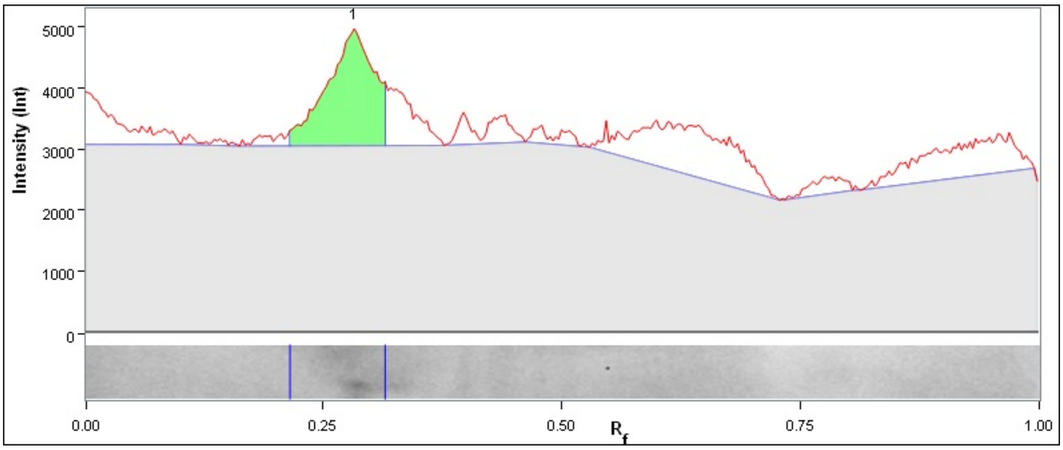

Band No.	Band Label	Mol. Wt. (KDa)	Relative Front	Adj. Volume (Int)	Volume (Int)	Abs. Quant.	Rel. Quant.	Band %	Lane %
1		N/A	0,284	1 607 034	6 207 396	N/A	N/A	100,0	27,2

Band Detection	Automatically detected bands with sensitivity: Low
Lane Background	Lane background subtracted with disk size: 10
Lane Width	6.39 mm

Lane 2

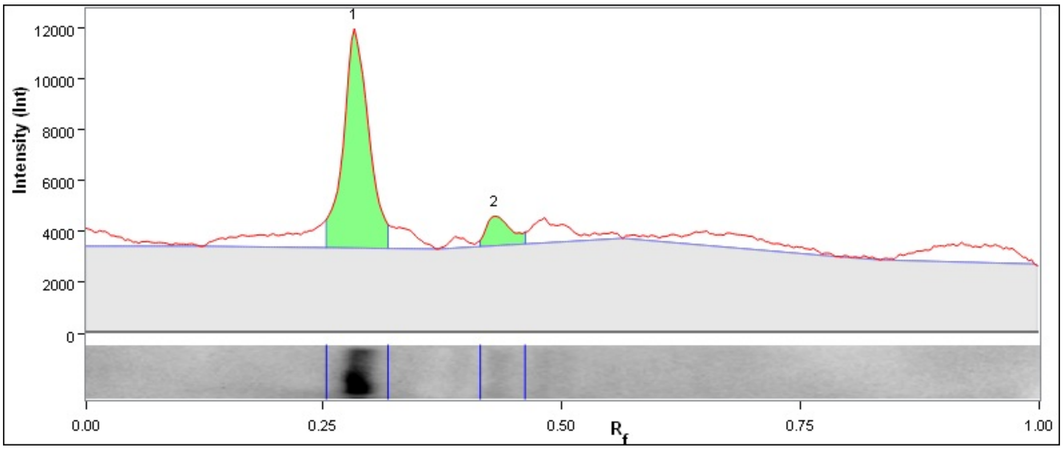

Band No.	Band Label	Mol. Wt. (KDa)	Relative Front	Adj. Volume (Int)	Volume (Int)	Abs. Quant.	Rel. Quant.	Band %	Lane %
1		N/A	0,284	4 780 688	8 737 608	N/A	N/A	88,1	43,0
2		N/A	0,431	642 804	3 664 176	N/A	N/A	11,9	5,8

Band Detection	Automatically detected bands with sensitivity: Low
Lane Background	Lane background subtracted with disk size: 10
Lane Width	7.53 mm

Lane 3

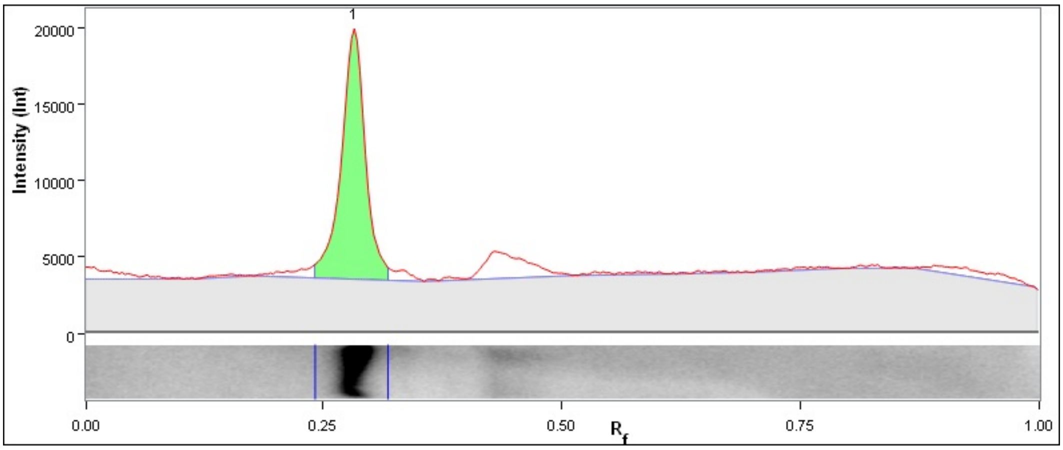

Band No.	Band Label	Mol. Wt. (KDa)	Relative Front	Adj. Volume (Int)	Volume (Int)	Abs. Quant.	Rel. Quant.	Band %	Lane %
1		N/A	0,284	7 886 036	12 368 661	N/A	N/A	100,0	65,4

Band Detection	Automatically detected bands with sensitivity: Low
Lane Background	Lane background subtracted with disk size: 10
Lane Width	7.70 mm

Lane 4

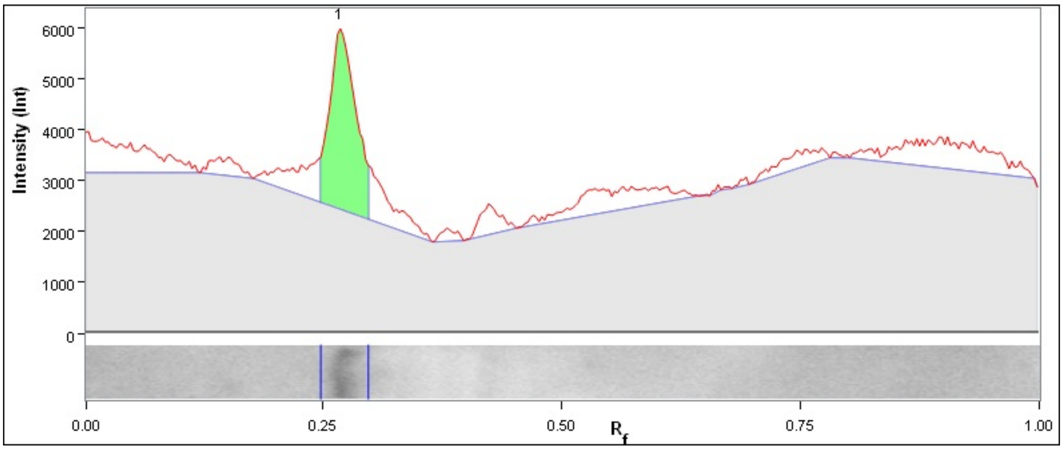

Band No.	Band Label	Mol. Wt. (KDa)	Relative Front	Adj. Volume (Int)	Volume (Int)	Abs. Quant.	Rel. Quant.	Band %	Lane %
1		N/A	0,270	1 562 691	3 268 590	N/A	N/A	100,0	29,5

Band Detection	Automatically detected bands with sensitivity: Low
Lane Background	Lane background subtracted with disk size: 10
Lane Width	6.39 mm

Lane 5

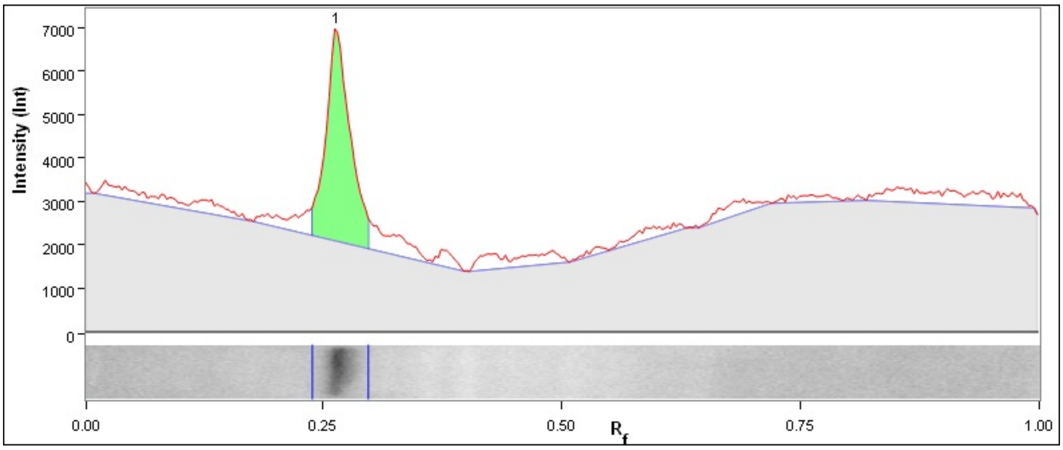

Band No.	Band Label	Mol. Wt. (KDa)	Relative Front	Adj. Volume (Int)	Volume (Int)	Abs. Quant.	Rel. Quant.	Band %	Lane %
1		N/A	0,267	2 404 646	4 506 572	N/A	N/A	100,0	43,6

Band Detection	Automatically detected bands with sensitivity: Low
Lane Background	Lane background subtracted with disk size: 10
Lane Width	7.04 mm

Lane 6

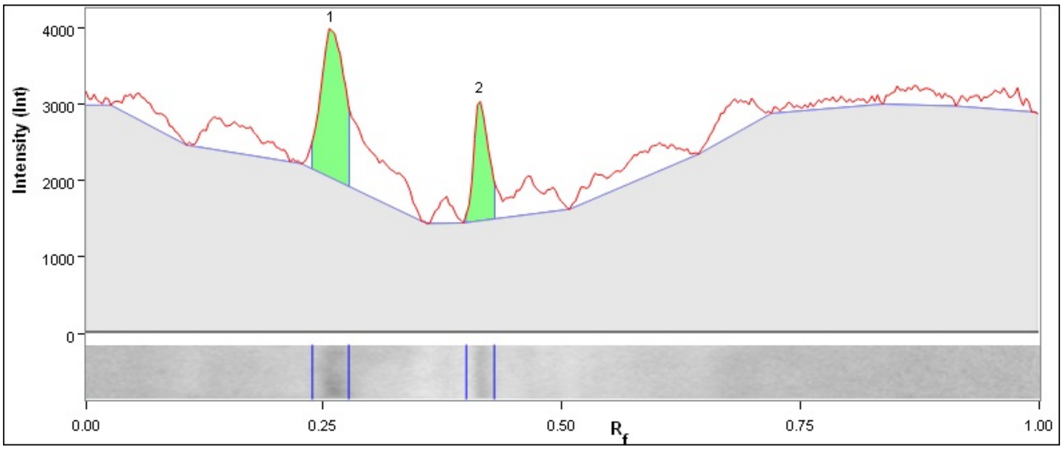

Band No.	Band Label	Mol. Wt. (KDa)	Relative Front	Adj. Volume (Int)	Volume (Int)	Abs. Quant.	Rel. Quant.	Band %	Lane %
1		N/A	0,261	874 731	2 187 861	N/A	N/A	66,1	19,5
2		N/A	0,416	448 500	1 191 879	N/A	N/A	33,9	10,0

Band Detection	Automatically detected bands with sensitivity: Low
Lane Background	Lane background subtracted with disk size: 10
Lane Width	6.39 mm

Lane 7

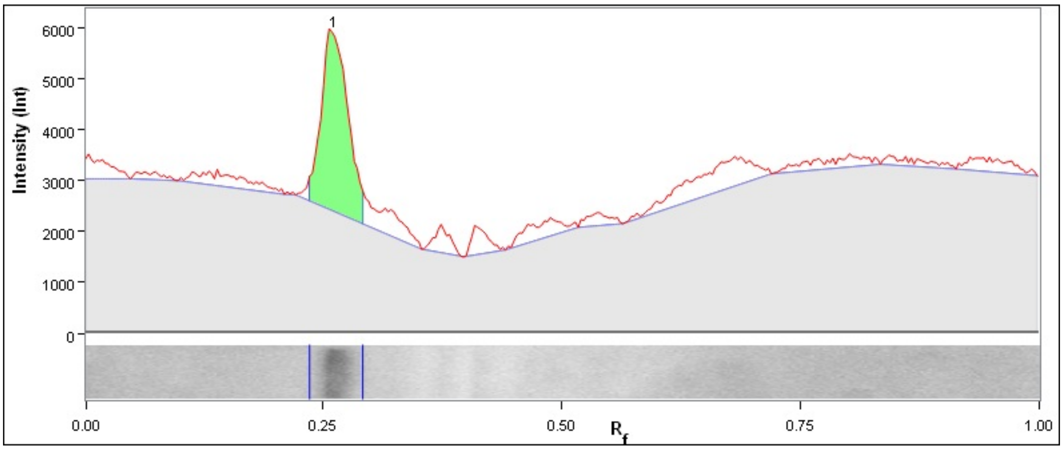

Band No.	Band Label	Mol. Wt. (KDa)	Relative Front	Adj. Volume (Int)	Volume (Int)	Abs. Quant.	Rel. Quant.	Band %	Lane %
1		N/A	0,264	1 927 772	4 199 096	N/A	N/A	100,0	38,0

Band Detection	Automatically detected bands with sensitivity: Low
Lane Background	Lane background subtracted with disk size: 10
Lane Width	7.21 mm

Lane 8

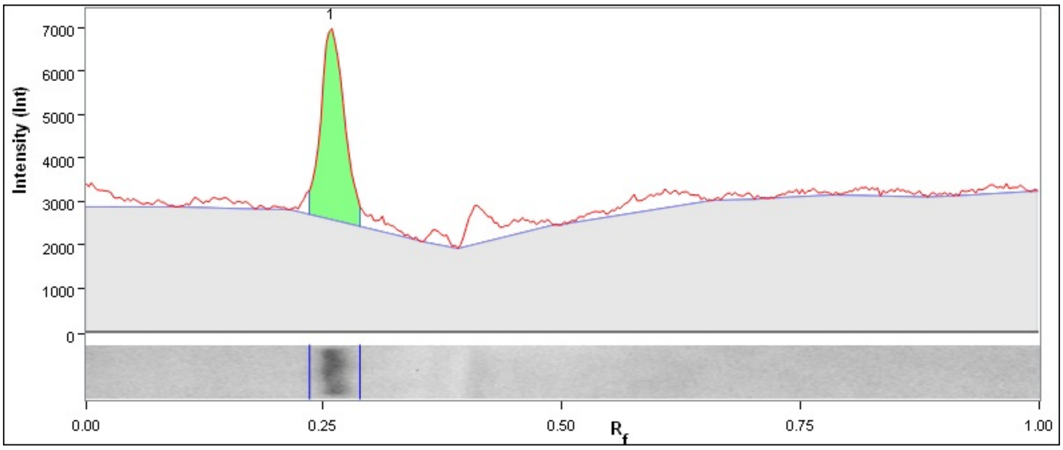

Band No.	Band Label	Mol. Wt. (KDa)	Relative Front	Adj. Volume (Int)	Volume (Int)	Abs. Quant.	Rel. Quant.	Band %	Lane %
1		N/A	0,261	2 341 155	4 978 416	N/A	N/A	100,0	45,1

Band Detection	Automatically detected bands with sensitivity: Low
Lane Background	Lane background subtracted with disk size: 10
Lane Width	8.35 mm

Volume Analysis

No.	Label	Type	Volume (Int)	Adj. Vol. (Int)	Mean Bkgd. (Int)	Abs. Quant.	Rel. Quant.	# of Pixels	Min. Value (Int)	Max. Value (Int)	Mean Value (Int)	Std. Dev.	Area (mm2)
1	U1	Unknown	12 623 276	500 716	4 170,1	N/A	N/A	2 907	2 616	7 588	4 342,4	748,1	78,0
2	U2	Unknown	17 972 832	1 668 262	5 608,7	N/A	N/A	2 907	3 004	32 916	6 182,6	3 489,2	78,0
3	U3	Unknown	18 569 412	5 651 021	4 443,9	N/A	N/A	2 907	1 780	35 116	6 387,8	5 338,0	78,0

4	U4	Unknown	10 028 064	530 895	3 267,0	N/A	N/A	2 907	1 364	7 224	3 449,6	1 023,7	78,0
5	U5	Unknown	10 190 040	1 865 449	2 863,6	N/A	N/A	2 907	1 484	10 652	3 505,3	1 477,7	78,0
6	U6	Unknown	8 987 332	563 216	2 897,9	N/A	N/A	2 907	1 224	6 096	3 091,6	701,8	78,0
7	U7	Unknown	10 066 384	1 510 872	2 943,1	N/A	N/A	2 907	1 288	8 204	3 462,8	1 208,5	78,0
8	U8	Unknown	10 450 932	1 988 126	2 911,2	N/A	N/A	2 907	1 576	9 892	3 595,1	1 512,2	78,0

Image Report: Histologia 2023-01-12 14hr 01min_Exposure_16.8sec pstat3 2a

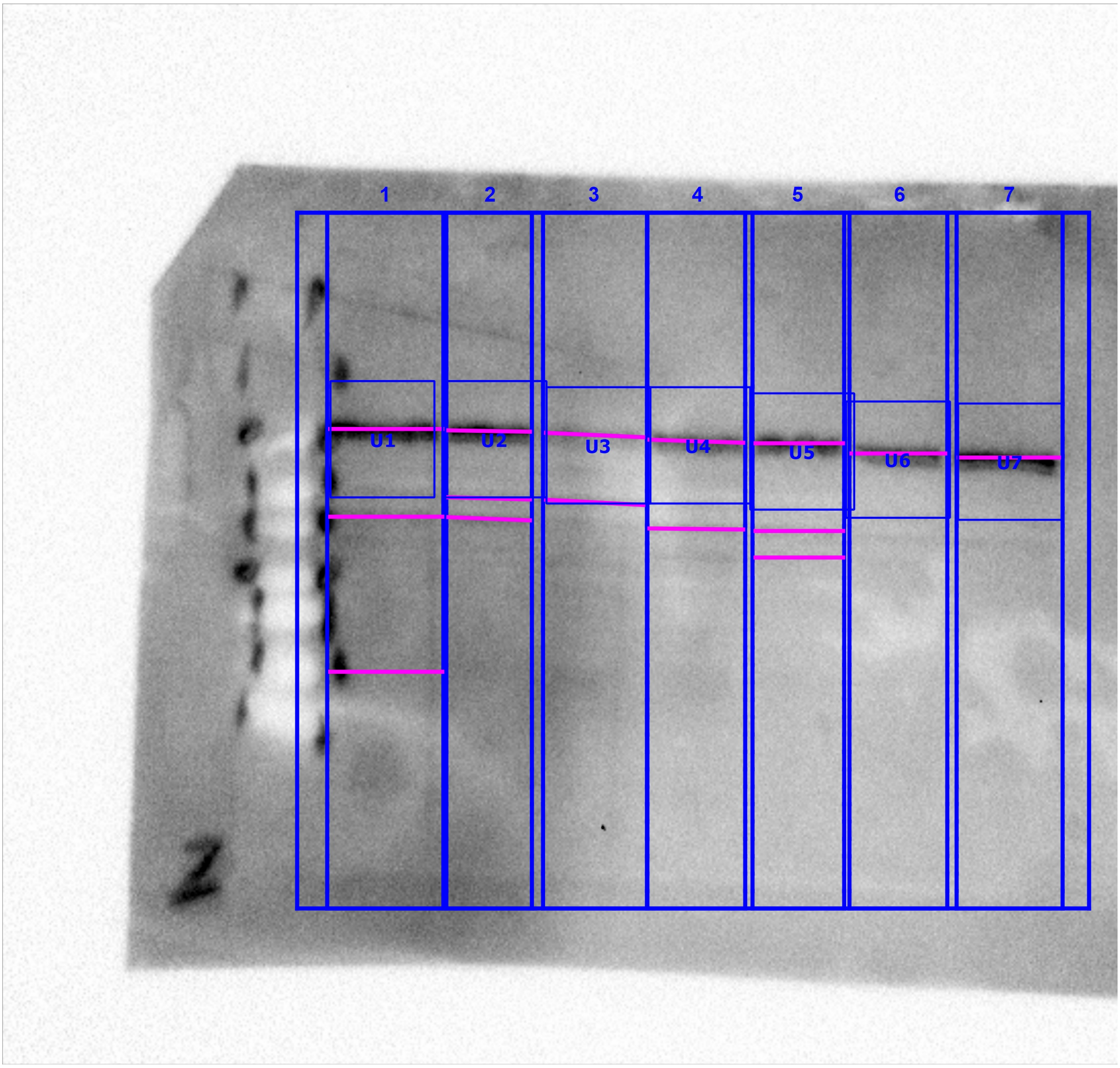

C:\Users\rusak\OneDrive\Dokumenty\Badania\CHI3L2 in BC\BC westerny ilościowo\pSTAT 3 BC 12.1.23\pSTAT3 BC 12.1.23 2\Histologia 2023-01-12 14hr 01min_Exposure_16.8sec pstat3 2a.scn

Acquisition Information

Imager	ChemiDoc MP
Exposure Time (sec)	16.800 (Signal Accumulation)
Flat Field	Applied (Lens)

Serial Number	731BR01769
Software Version	5.0
Application	Chemi Hi Resolution
Excitation Source	No Illumination
Emission Filter	No Filter
Binning	2x2

Image Information

Acquisition Date	12/1/2023 2:01:47 PM
User Name	Histologia
Image Area (mm)	X: 114.0 Y: 85.2
Pixel Size (μm)	X: 163.8 Y: 163.8
Data Range (Int)	0 - 18588

Analysis Settings

Detection	Lane detection:<br>Manually created lanes<br><br>Band detection:<br>Automatically detected bands with sensitivity: Low<br><br>Lane Background Subtraction:<br>Lane background subtracted with disk size: 10<br><br>Lane width: Variable
Volume Analysis	Background subtraction method: Local<br>Quantity regression method: Linear

Lane Statistics

Lane No.	Adj. Total Band Vol. (Int)	Total Band Vol. (Int)	Adj. Total Lane Vol. (Int)	Total Lane Vol. (Int)	Bkgd. Vol. (Int)	Norm. Factor
1	5 507 910	40 913 175	8 681 556	102 352 620	93 671 064	N/A
2	2 742 768	9 745 428	6 884 682	70 414 596	63 529 914	N/A
3	935 340	5 402 226	5 424 258	69 474 546	64 050 288	N/A
4	2 218 176	6 148 320	5 345 088	52 716 384	47 371 296	N/A
5	3 162 510	11 589 480	5 443 470	50 657 175	45 213 705	N/A
6	1 869 024	5 147 568	6 060 144	54 531 648	48 471 504	N/A
7	3 025 724	7 490 080	7 751 952	65 753 896	58 001 944	N/A

Lane And Band Analysis

Lane 1

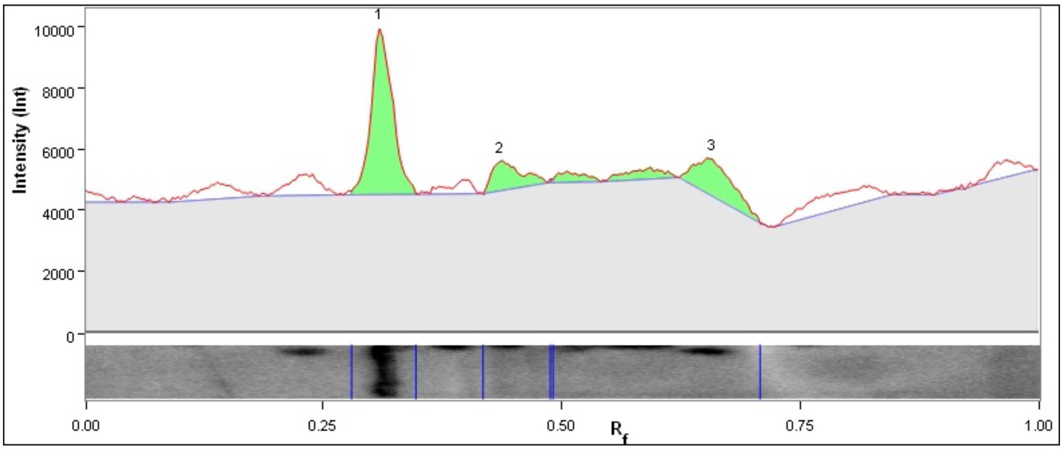

Band No.	Band Label	Mol. Wt. (KDa)	Relative Front	Adj. Volume (Int)	Volume (Int)	Abs. Quant.	Rel. Quant.	Band %	Lane %
1		N/A	0,311	2 978 934	9 590 535	N/A	N/A	54,1	34,3
2		N/A	0,437	726 750	7 941 867	N/A	N/A	13,2	8,4
3		N/A	0,660	1 802 226	23 380 773	N/A	N/A	32,7	20,8

Band Detection	Automatically detected bands with sensitivity: Low
Lane Background	Lane background subtracted with disk size: 10
Lane Width	9.34 mm

Lane 2

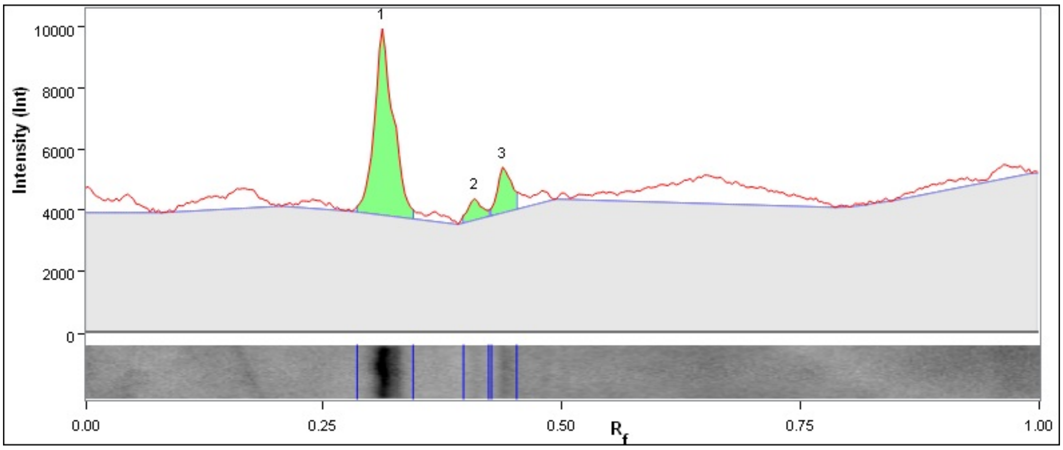

Band No.	Band Label	Mol. Wt. (KDa)	Relative Front	Adj. Volume (Int)	Volume (Int)	Abs. Quant.	Rel. Quant.	Band %	Lane %
1		N/A	0,314	2 161 068	5 757 948	N/A	N/A	78,8	31,4
2		N/A	0,411	185 976	1 835 862	N/A	N/A	6,8	2,7
3		N/A	0,440	395 724	2 151 618	N/A	N/A	14,4	5,7

Band Detection	Automatically detected bands with sensitivity: Low
Lane Background	Lane background subtracted with disk size: 10
Lane Width	6.88 mm

Lane 3

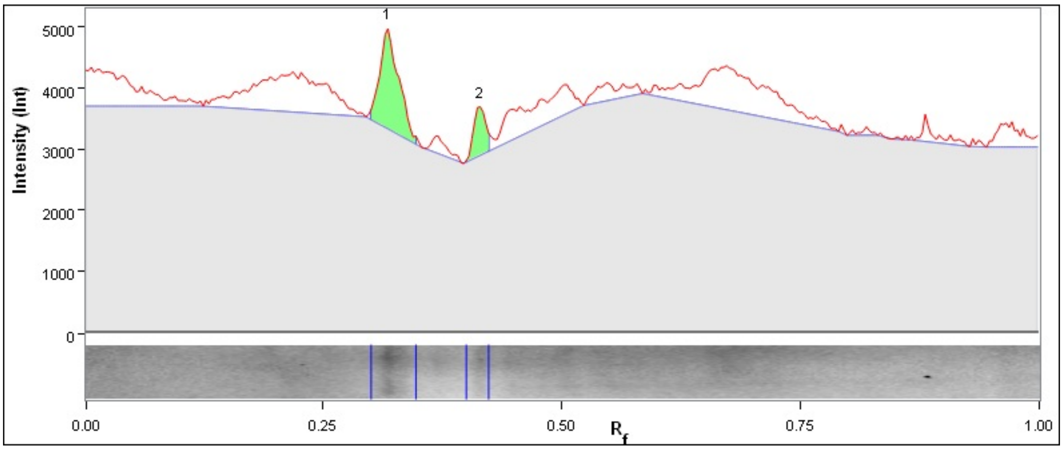

Band No.	Band Label	Mol. Wt. (KDa)	Relative Front	Adj. Volume (Int)	Volume (Int)	Abs. Quant.	Rel. Quant.	Band %	Lane %
1		N/A	0,320	710 226	3 761 250	N/A	N/A	75,9	13,1
2		N/A	0,416	225 114	1 640 976	N/A	N/A	24,1	4,2

Band Detection	Automatically detected bands with sensitivity: Low
Lane Background	Lane background subtracted with disk size: 10
Lane Width	8.35 mm

Lane 4

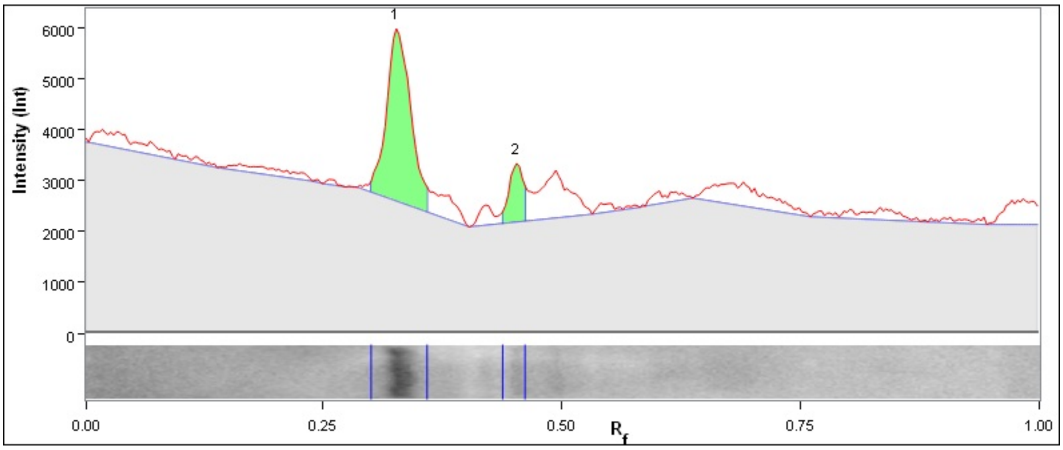

Band No.	Band Label	Mol. Wt. (KDa)	Relative Front	Adj. Volume (Int)	Volume (Int)	Abs. Quant.	Rel. Quant.	Band %	Lane %
1		N/A	0,328	1 847 184	4 733 280	N/A	N/A	83,3	34,6
2		N/A	0,455	370 992	1 415 040	N/A	N/A	16,7	6,9

Band Detection	Automatically detected bands with sensitivity: Low
Lane Background	Lane background subtracted with disk size: 10
Lane Width	7.86 mm

Lane 5

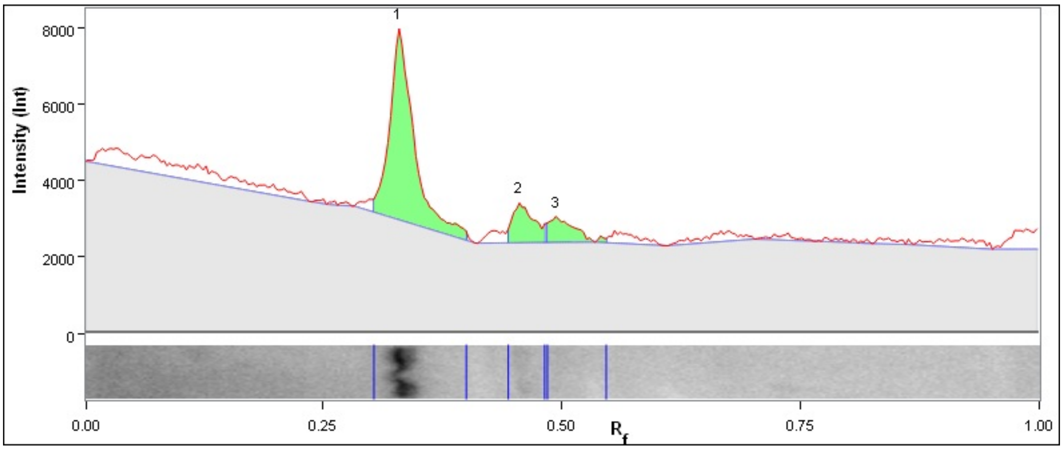

Band No.	Band Label	Mol. Wt. (KDa)	Relative Front	Adj. Volume (Int)	Volume (Int)	Abs. Quant.	Rel. Quant.	Band %	Lane %
1		N/A	0,331	2 370 645	6 816 060	N/A	N/A	75,0	43,6
2		N/A	0,457	445 185	1 989 720	N/A	N/A	14,1	8,2
3		N/A	0,496	346 680	2 783 700	N/A	N/A	11,0	6,4

Band Detection	Automatically detected bands with sensitivity: Low
Lane Background	Lane background subtracted with disk size: 10
Lane Width	7.37 mm

Lane 6

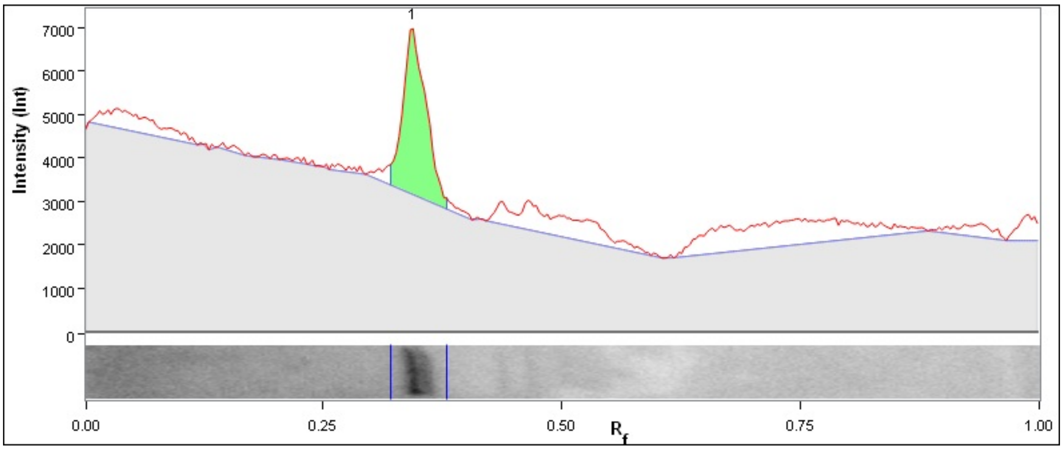

Band No.	Band Label	Mol. Wt. (KDa)	Relative Front	Adj. Volume (Int)	Volume (Int)	Abs. Quant.	Rel. Quant.	Band %	Lane %
1		N/A	0,346	1 869 024	5 147 568	N/A	N/A	100,0	30,8

Band Detection	Automatically detected bands with sensitivity: Low
Lane Background	Lane background subtracted with disk size: 10
Lane Width	7.86 mm

Lane 7

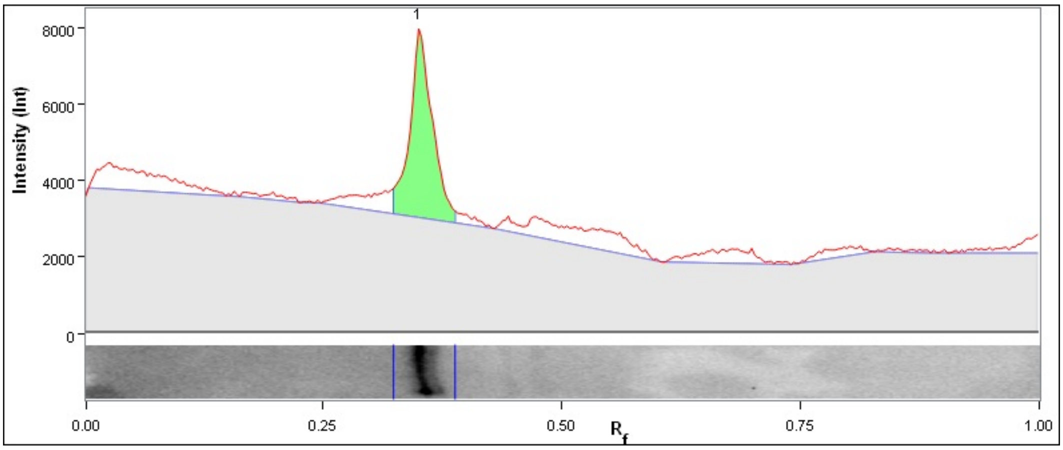

Band No.	Band Label	Mol. Wt. (KDa)	Relative Front	Adj. Volume (Int)	Volume (Int)	Abs. Quant.	Rel. Quant.	Band %	Lane %
1		N/A	0,352	3 025 724	7 490 080	N/A	N/A	100,0	39,0

Band Detection	Automatically detected bands with sensitivity: Low
Lane Background	Lane background subtracted with disk size: 10
Lane Width	8.52 mm

Volume Analysis

No.	Label	Type	Volume (Int)	Adj. Vol. (Int)	Mean Bkgd. (Int)	Abs. Quant.	Rel. Quant.	# of Pixels	Min. Value (Int)	Max. Value (Int)	Mean Value (Int)	Std. Dev.	Area (mm2)
1	U1	Unknown	17 054 448	-389 878	6 000,8	N/A	N/A	2 907	3 340	15 232	5 866,7	1 910,3	78,0
2	U2	Unknown	14 465 040	886 919	4 670,8	N/A	N/A	2 907	3 040	13 104	4 975,9	1 607,3	78,0
3	U3	Unknown	10 991 756	-314 096	3 889,2	N/A	N/A	2 907	1 252	7 460	3 781,1	957,9	78,0
4	U4	Unknown	10 484 816	1 341 772	3 145,2	N/A	N/A	2 907	912	7 968	3 606,7	1 230,0	78,0
5	U5	Unknown	11 317 608	1 471 705	3 387,0	N/A	N/A	2 907	1 656	10 444	3 893,2	1 490,3	78,0
6	U6	Unknown	11 595 080	866 294	3 690,7	N/A	N/A	2 907	1 896	9 788	3 988,7	1 326,8	78,0
7	U7	Unknown	14 455 064	2 060 515	4 263,7	N/A	N/A	2 907	2 352	13 592	4 972,5	1 758,2	78,0

Image Report: Histologia 2023-01-12 14hr 09min_Exposure_16.8sec pstat3 3a

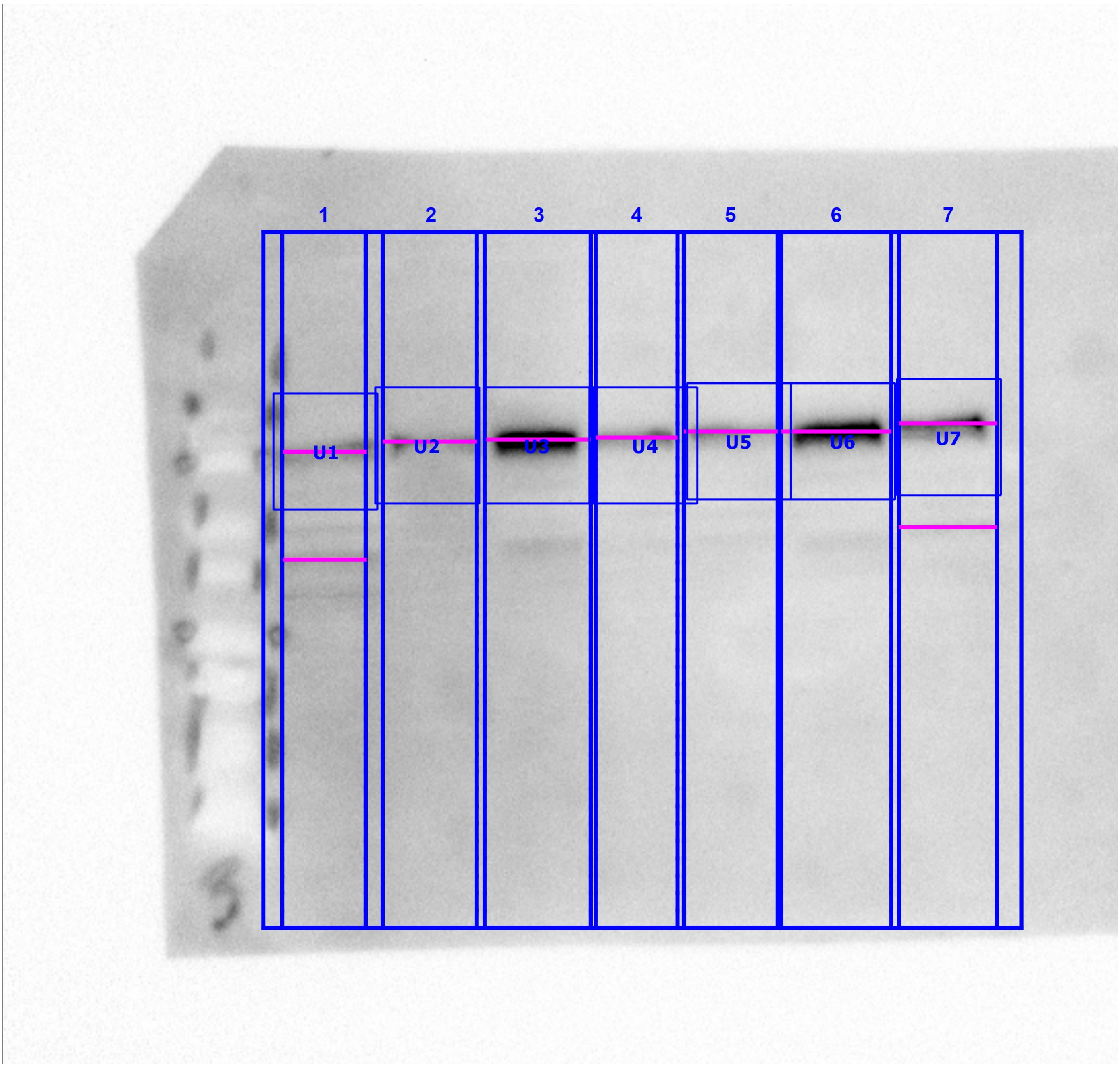

C:\Users\rusak\OneDrive\Dokumenty\Badania\CHI3L2 in BC\BC westerny ilościowo\pSTAT 3 BC 12.1.23\pSTAT3 BC 3 12.1.23\Histologia 2023-01-12 14hr 09min_Exposure_16.8sec pstat3 3a.scn

Acquisition Information

Imager	ChemiDoc MP
Exposure Time (sec)	16.800 (Signal Accumulation)
Flat Field	Applied (Lens)

Serial Number	731BR01769
Software Version	5.0
Application	Chemi Hi Resolution
Excitation Source	No Illumination
Emission Filter	No Filter
Binning	2x2

Image Information

Acquisition Date	12/1/2023 2:10:01 PM
User Name	Histologia
Image Area (mm)	X: 114.0 Y: 85.2
Pixel Size (µm)	X: 163.8 Y: 163.8
Data Range (Int)	0 - 28268

Analysis Settings

Detection	Lane detection:<br>Manually created lanes<br><br>Band detection:<br>Automatically detected bands with sensitivity: Low<br><br>Lane Background Subtraction:<br>Lane background subtracted with disk size: 10<br><br>Lane width: Variable
Volume Analysis	Background subtraction method: Local<br>Quantity regression method: Linear

Lane Statistics

Lane No.	Adj. Total Band Vol. (Int)	Total Band Vol. (Int)	Adj. Total Lane Vol. (Int)	Total Lane Vol. (Int)	Bkgd. Vol. (Int)	Norm. Factor
1	2 216 460	8 585 728	6 580 131	74 100 366	67 520 235	N/A
2	1 488 928	5 649 306	4 748 994	78 825 002	74 076 008	N/A
3	10 055 240	15 888 912	18 134 116	82 565 860	64 431 744	N/A
4	2 350 240	4 602 680	5 051 440	45 563 120	40 511 680	N/A
5	2 633 868	5 264 056	6 521 558	48 255 058	41 733 500	N/A
6	9 452 646	13 300 362	14 541 444	61 033 500	46 492 056	N/A
7	5 083 248	10 869 840	7 380 432	51 827 040	44 446 608	N/A

Lane And Band Analysis

Lane 1

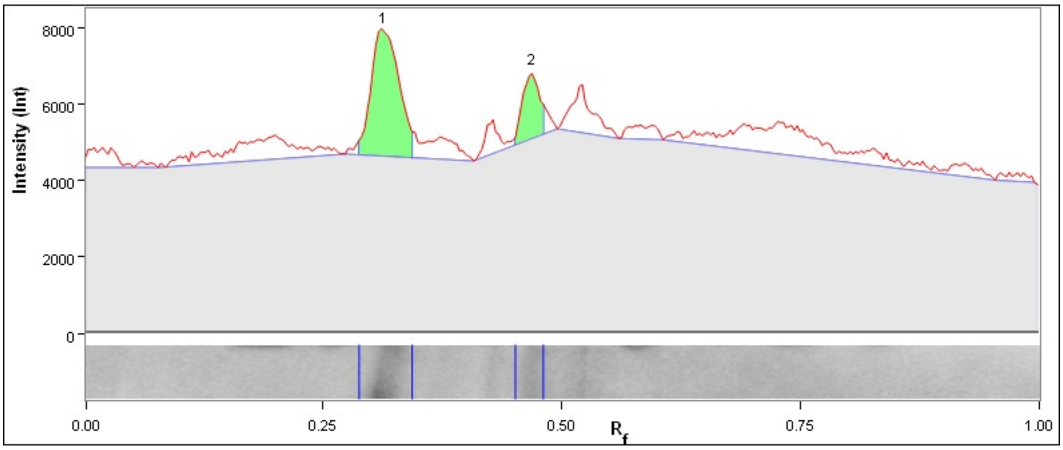

Band No.	Band Label	Mol. Wt. (KDa)	Relative Front	Adj. Volume (Int)	Volume (Int)	Abs. Quant.	Rel. Quant.	Band %	Lane %
1		N/A	0,316	1 684 403	5 659 599	N/A	N/A	76,0	25,6
2		N/A	0,471	532 057	2 926 129	N/A	N/A	24,0	8,1

Band Detection	Automatically detected bands with sensitivity: Low
Lane Background	Lane background subtracted with disk size: 10
Lane Width	6.72 mm

Lane 2

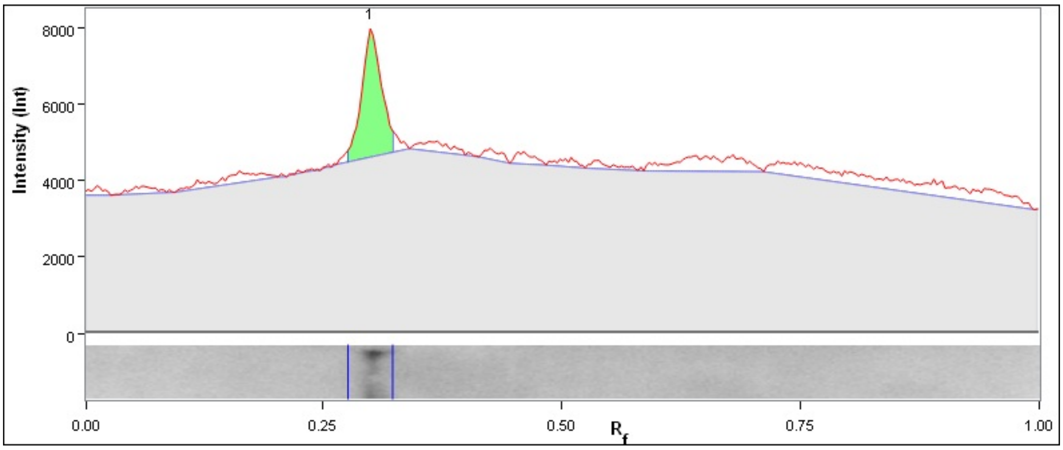

Band No.	Band Label	Mol. Wt. (KDa)	Relative Front	Adj. Volume (Int)	Volume (Int)	Abs. Quant.	Rel. Quant.	Band %	Lane %
1		N/A	0,301	1 488 928	5 649 306	N/A	N/A	100,0	31,4

Band Detection	Automatically detected bands with sensitivity: Low
Lane Background	Lane background subtracted with disk size: 10
Lane Width	7.53 mm

Lane 3

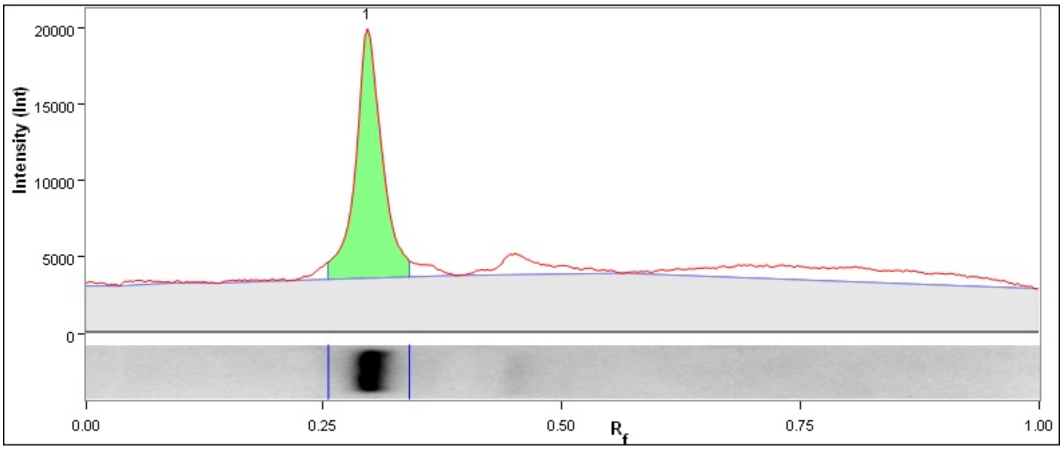

Band No.	Band Label	Mol. Wt. (KDa)	Relative Front	Adj. Volume (Int)	Volume (Int)	Abs. Quant.	Rel. Quant.	Band %	Lane %
1		N/A	0,298	10 055 240	15 888 912	N/A	N/A	100,0	55,4

Band Detection	Automatically detected bands with sensitivity: Low
Lane Background	Lane background subtracted with disk size: 10
Lane Width	8.52 mm

Lane 4

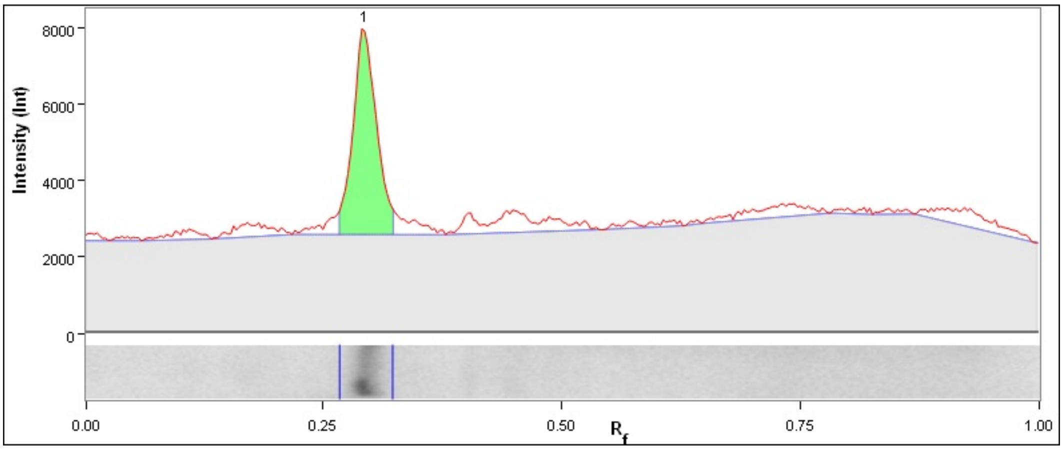

Band No.	Band Label	Mol. Wt. (KDa)	Relative Front	Adj. Volume (Int)	Volume (Int)	Abs. Quant.	Rel. Quant.	Band %	Lane %
1		N/A	0,295	2 350 240	4 602 680	N/A	N/A	100,0	46,5

Band Detection	Automatically detected bands with sensitivity: Low
Lane Background	Lane background subtracted with disk size: 10
Lane Width	6.55 mm

Lane 5

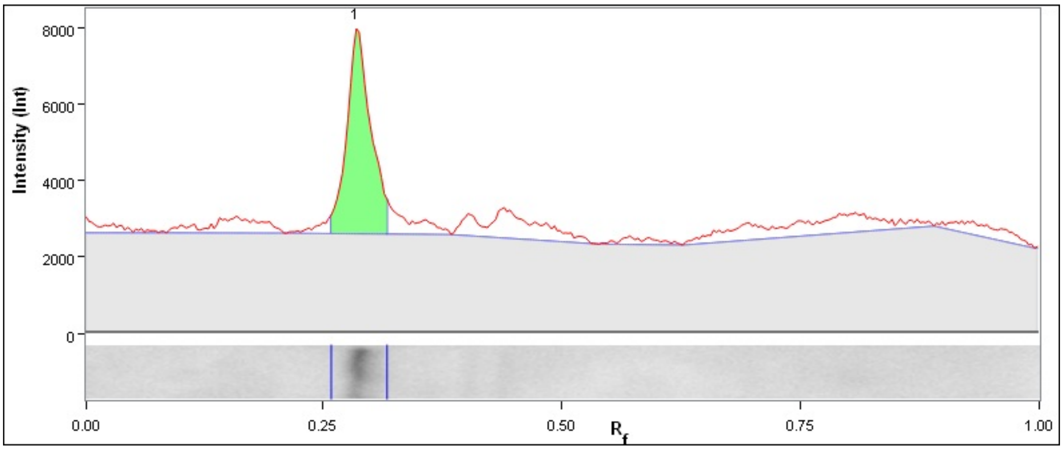

Band No.	Band Label	Mol. Wt. (KDa)	Relative Front	Adj. Volume (Int)	Volume (Int)	Abs. Quant.	Rel. Quant.	Band %	Lane %
1		N/A	0,287	2 633 868	5 264 056	N/A	N/A	100,0	40,4

Band Detection	Automatically detected bands with sensitivity: Low
Lane Background	Lane background subtracted with disk size: 10
Lane Width	7.53 mm

Lane 6

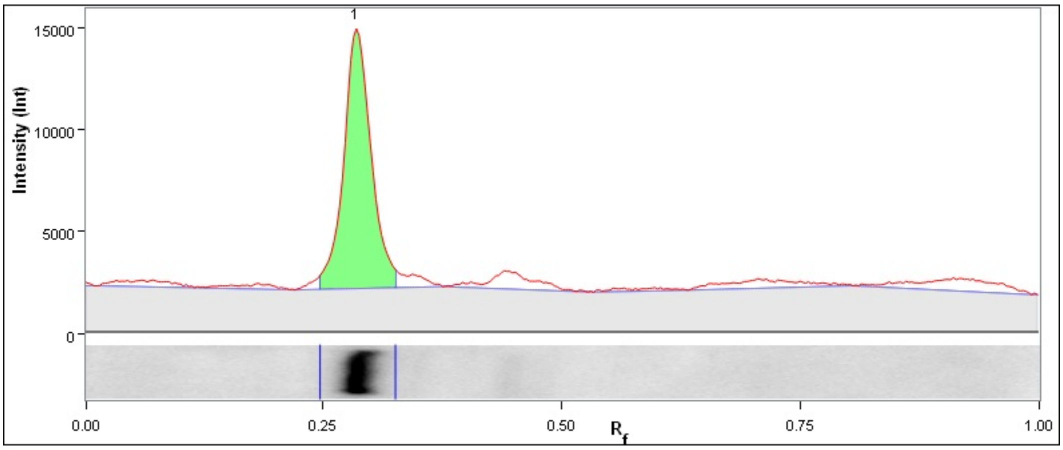

Band No.	Band Label	Mol. Wt. (KDa)	Relative Front	Adj. Volume (Int)	Volume (Int)	Abs. Quant.	Rel. Quant.	Band %	Lane %
1		N/A	0,287	9 452 646	13 300 362	N/A	N/A	100,0	65,0

Band Detection	Automatically detected bands with sensitivity: Low
Lane Background	Lane background subtracted with disk size: 10
Lane Width	8.84 mm

Lane 7

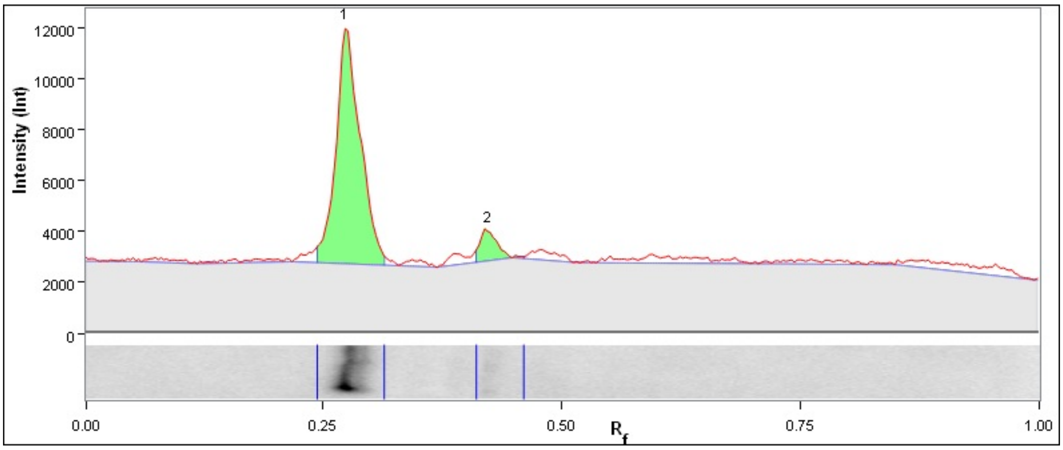

Band No.	Band Label	Mol. Wt. (KDa)	Relative Front	Adj. Volume (Int)	Volume (Int)	Abs. Quant.	Rel. Quant.	Band %	Lane %
1		N/A	0,275	4 633 344	7 909 776	N/A	N/A	91,1	62,8
2		N/A	0,424	449 904	2 960 064	N/A	N/A	8,9	6,1

Band Detection	Automatically detected bands with sensitivity: Low
Lane Background	Lane background subtracted with disk size: 10
Lane Width	7.86 mm

Volume Analysis

No.	Label	Type	Volume (Int)	Adj. Vol. (Int)	Mean Bkgd. (Int)	Abs. Quant.	Rel. Quant.	# of Pixels	Min. Value (Int)	Max. Value (Int)	Mean Value (Int)	Std. Dev.	Area (mm2)
1	U1	Unknown	17 171 564	-235 763	5 988,1	N/A	N/A	2 907	3 788	12 000	5 907,0	1 375,4	78,0
2	U2	Unknown	17 266 020	1 319 539	5 485,5	N/A	N/A	2 907	3 644	14 124	5 939,5	1 265,0	78,0
3	U3	Unknown	21 699 428	9 470 049	4 206,9	N/A	N/A	2 907	2 528	28 268	7 464,5	5 675,0	78,0
4	U4	Unknown	11 623 336	1 443 128	3 502,0	N/A	N/A	2 907	2 120	12 628	3 998,4	1 683,9	78,0
5	U5	Unknown	11 284 672	1 095 214	3 505,1	N/A	N/A	2 907	1 604	10 952	3 881,9	1 612,6	78,0
6	U6	Unknown	17 294 248	7 786 085	3 270,8	N/A	N/A	2 907	1 500	24 192	5 949,2	5 134,3	78,0
7	U7	Unknown	12 907 672	3 726 045	3 158,5	N/A	N/A	2 907	1 704	21 820	4 440,2	2 941,2	78,0

Image Report: Histologia 2023-01-12 13hr 58min_Exposure_16.8sec pstat3 1a

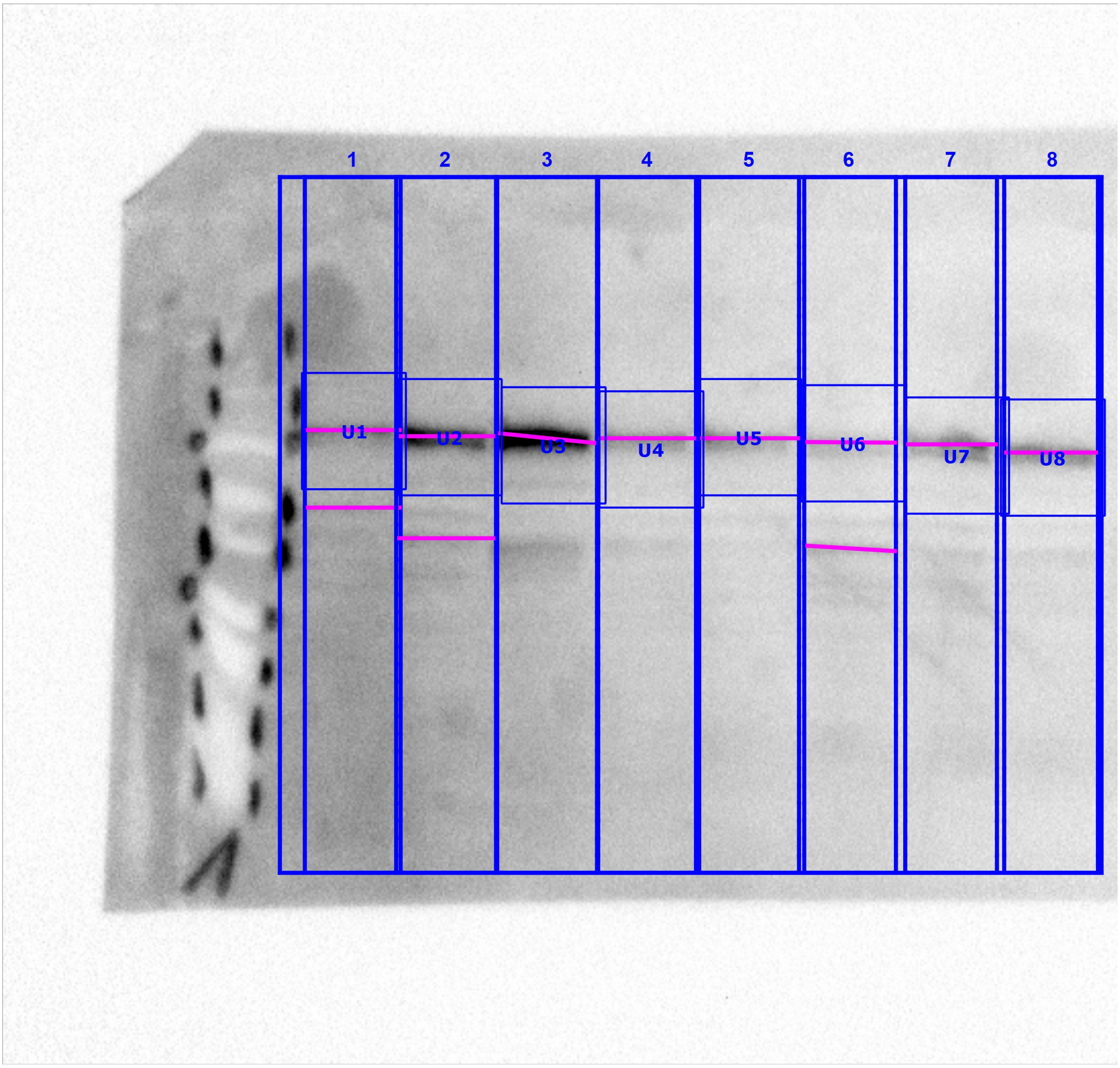

C:\Users\rusak\OneDrive\Dokumenty\Badania\CHI3L2 in BC\BC westerny ilościowo\pSTAT 3 BC 12.1.23\pSTAT3 BC 1 12.1.23\Histologia 2023-01-12 13hr 58min_Exposure_16.8sec pstat3 1a.scn

---

Acquisition Information

Imager	ChemiDoc MP
Exposure Time (sec)	16.800 (Signal Accumulation)
Flat Field	Applied (Lens)

Serial Number	731BR01769
Software Version	5.0
Application	Chemi Hi Resolution
Excitation Source	No Illumination
Emission Filter	No Filter
Binning	2x2

Image Information

Acquisition Date	12/1/2023 1:59:18 PM
User Name	Histologia
Image Area (mm)	X: 114.0 Y: 85.2
Pixel Size (µm)	X: 163.8 Y: 163.8
Data Range (Int)	0 - 19812

Analysis Settings

Detection	Lane detection:<br>Manually created lanes<br><br>Band detection:<br>Automatically detected bands with sensitivity: Low<br><br>Lane Background Subtraction:<br>Lane background subtracted with disk size: 10<br><br>Lane width: Variable
Volume Analysis	Background subtraction method: Local<br>Quantity regression method: Linear

Lane Statistics

Lane No.	Adj. Total Band Vol. (Int)	Total Band Vol. (Int)	Adj. Total Lane Vol. (Int)	Total Lane Vol. (Int)	Bkgd. Vol. (Int)	Norm. Factor
1	1 371 883	6 655 341	5 867 339	70 861 900	64 994 561	N/A
2	4 164 069	10 940 965	7 767 039	65 407 356	57 640 317	N/A
3	6 450 752	10 433 570	10 018 932	59 312 295	49 293 363	N/A
4	1 652 352	3 409 728	4 446 528	41 482 752	37 036 224	N/A
5	1 585 738	3 268 349	4 831 939	35 259 861	30 427 922	N/A
6	1 638 945	3 505 590	4 989 420	29 319 300	24 329 880	N/A
7	2 137 860	3 637 845	5 251 905	28 317 465	23 065 560	N/A
8	2 113 240	3 354 320	4 969 058	25 242 592	20 273 534	N/A

Lane And Band Analysis

Lane 1

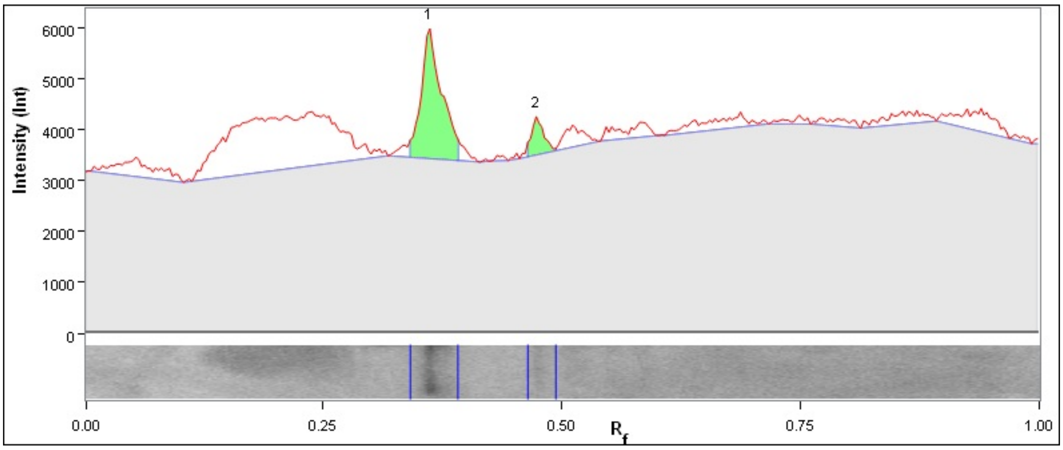

Band No.	Band Label	Mol. Wt. (KDa)	Relative Front	Adj. Volume (Int)	Volume (Int)	Abs. Quant.	Rel. Quant.	Band %	Lane %
1		N/A	0,364	1 165 694	4 408 412	N/A	N/A	85,0	19,9
2		N/A	0,475	206 189	2 246 929	N/A	N/A	15,0	3,5

Band Detection	Automatically detected bands with sensitivity: Low
Lane Background	Lane background subtracted with disk size: 10
Lane Width	7.70 mm

Lane 2

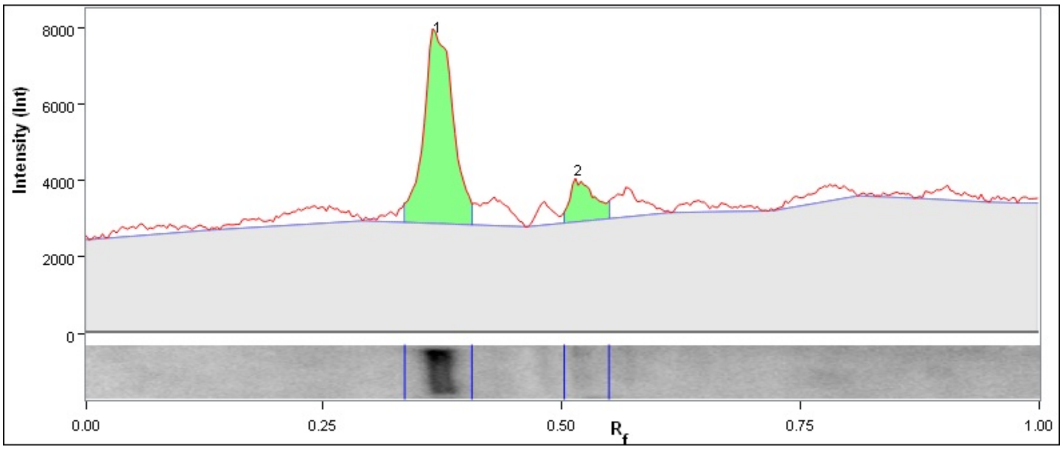

Band No.	Band Label	Mol. Wt. (KDa)	Relative Front	Adj. Volume (Int)	Volume (Int)	Abs. Quant.	Rel. Quant.	Band %	Lane %
1		N/A	0,372	3 508 547	7 501 557	N/A	N/A	84,3	45,2
2		N/A	0,519	655 522	3 439 408	N/A	N/A	15,7	8,4

Band Detection	Automatically detected bands with sensitivity: Low
Lane Background	Lane background subtracted with disk size: 10
Lane Width	8.03 mm

Lane 3

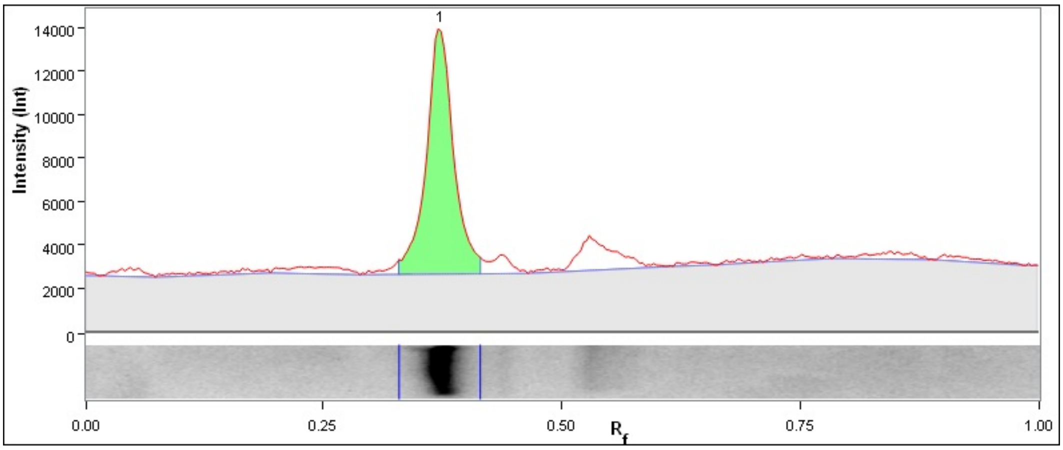

Band No.	Band Label	Mol. Wt. (KDa)	Relative Front	Adj. Volume (Int)	Volume (Int)	Abs. Quant.	Rel. Quant.	Band %	Lane %
1		N/A	0,375	6 450 752	10 433 570	N/A	N/A	100,0	64,4

Band Detection	Automatically detected bands with sensitivity: Low
Lane Background	Lane background subtracted with disk size: 10
Lane Width	8.03 mm

Lane 4

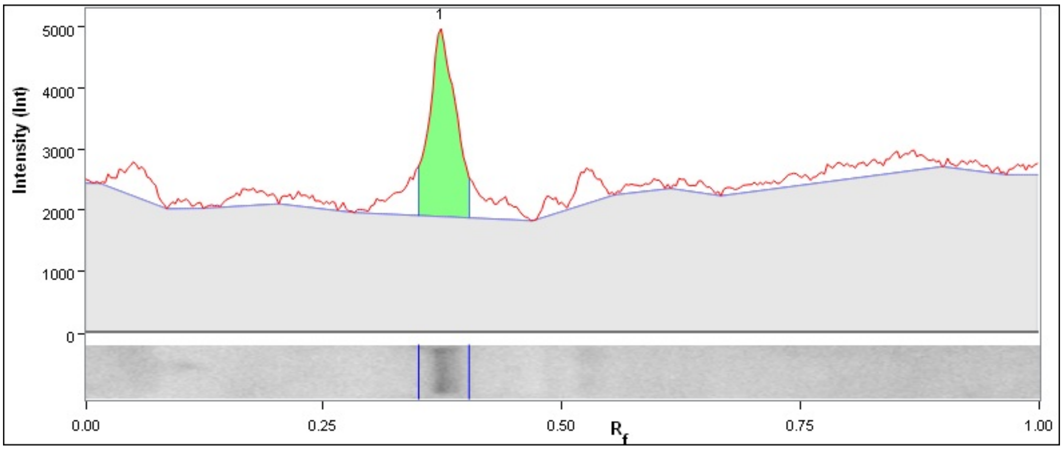

Band No.	Band Label	Mol. Wt. (KDa)	Relative Front	Adj. Volume (Int)	Volume (Int)	Abs. Quant.	Rel. Quant.	Band %	Lane %
1		N/A	0,375	1 652 352	3 409 728	N/A	N/A	100,0	37,2

Band Detection	Automatically detected bands with sensitivity: Low
Lane Background	Lane background subtracted with disk size: 10
Lane Width	7.86 mm

Lane 5

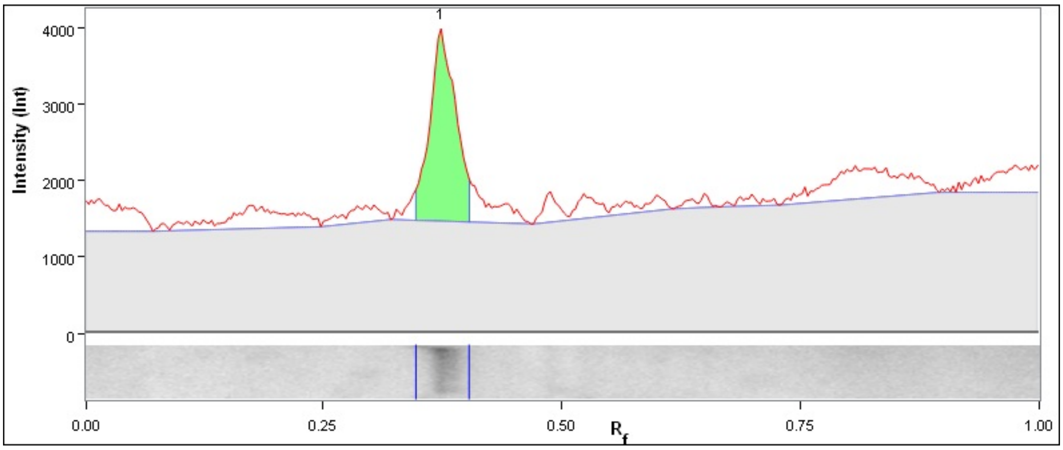

Band No.	Band Label	Mol. Wt. (KDa)	Relative Front	Adj. Volume (Int)	Volume (Int)	Abs. Quant.	Rel. Quant.	Band %	Lane %
1		N/A	0,375	1 585 738	3 268 349	N/A	N/A	100,0	32,8

Band Detection	Automatically detected bands with sensitivity: Low
Lane Background	Lane background subtracted with disk size: 10
Lane Width	8.03 mm

Lane 6

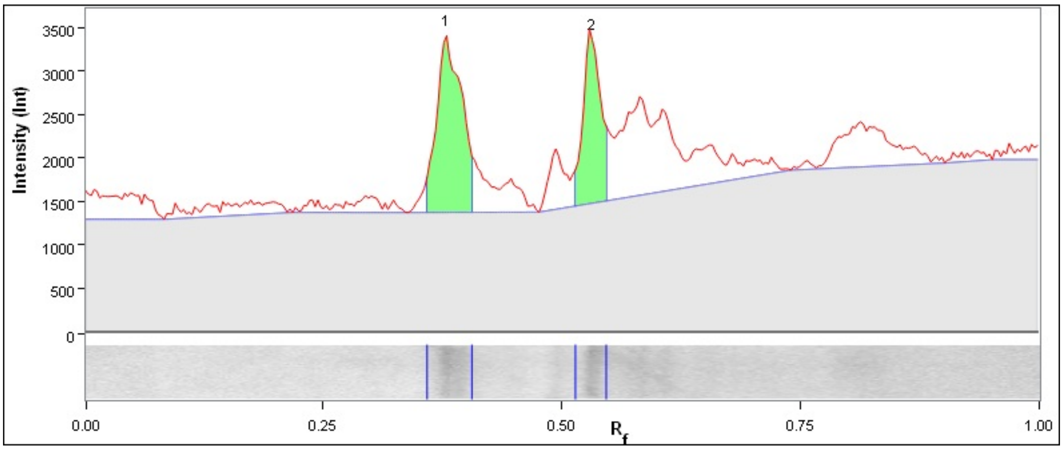

Band No.	Band Label	Mol. Wt. (KDa)	Relative Front	Adj. Volume (Int)	Volume (Int)	Abs. Quant.	Rel. Quant.	Band %	Lane %
1		N/A	0,381	975 960	2 037 420	N/A	N/A	59,5	19,6
2		N/A	0,534	662 985	1 468 170	N/A	N/A	40,5	13,3

Band Detection	Automatically detected bands with sensitivity: Low
Lane Background	Lane background subtracted with disk size: 10
Lane Width	7.37 mm

Lane 7

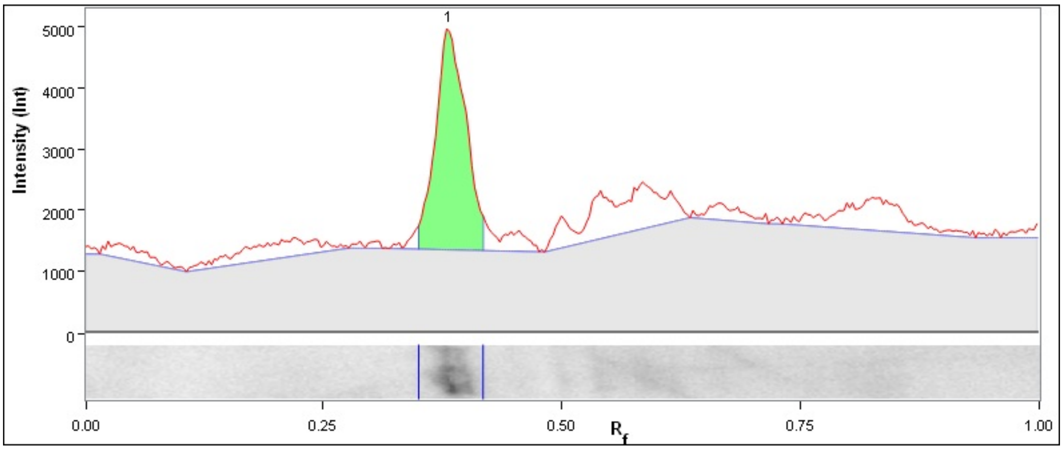

Band No.	Band Label	Mol. Wt. (KDa)	Relative Front	Adj. Volume (Int)	Volume (Int)	Abs. Quant.	Rel. Quant.	Band %	Lane %
1		N/A	0,384	2 137 860	3 637 845	N/A	N/A	100,0	40,7

Band Detection	Automatically detected bands with sensitivity: Low
Lane Background	Lane background subtracted with disk size: 10
Lane Width	7.37 mm

Lane 8

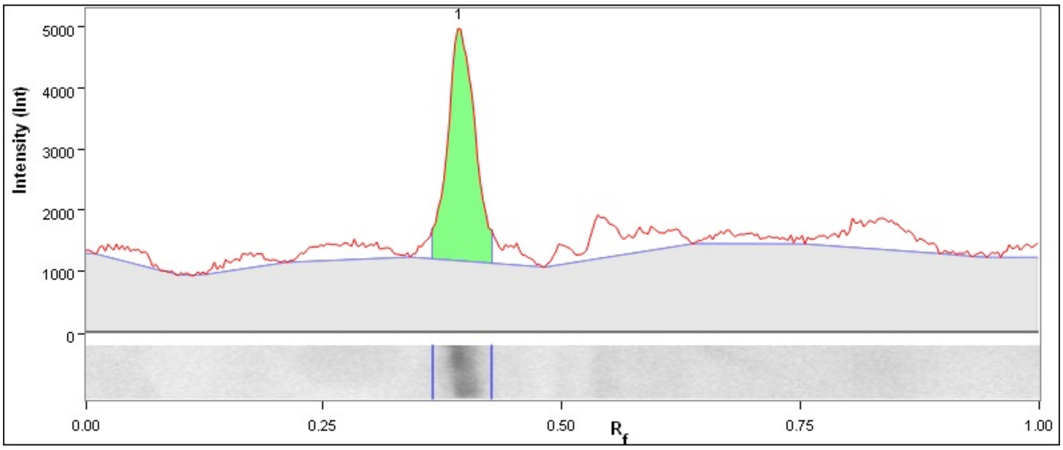

Band No.	Band Label	Mol. Wt. (KDa)	Relative Front	Adj. Volume (Int)	Volume (Int)	Abs. Quant.	Rel. Quant.	Band %	Lane %
1		N/A	0,396	2 113 240	3 354 320	N/A	N/A	100,0	42,5

Band Detection	Automatically detected bands with sensitivity: Low
Lane Background	Lane background subtracted with disk size: 10
Lane Width	7.53 mm

Volume Analysis

No.	Label	Type	Volume (Int)	Adj. Vol. (Int)	Mean Bkgd. (Int)	Abs. Quant.	Rel. Quant.	# of Pixels	Min. Value (Int)	Max. Value (Int)	Mean Value (Int)	Std. Dev.	Area (mm2)
1	U1	Unknown	12 850 956	-1 930 505	5 084,8	N/A	N/A	2 907	2 744	10 356	4 420,7	942,4	78,0
2	U2	Unknown	13 878 736	1 023 665	4 422,1	N/A	N/A	2 907	2 376	16 132	4 774,2	2 029,2	78,0
3	U3	Unknown	14 544 132	3 954 724	3 642,7	N/A	N/A	2 907	1 568	19 812	5 003,1	3 536,4	78,0

4	U4	Unknown	7 934 428	738 017	2 475,5	N/A	N/A	2 907	1 136	6 240	2 729,4	942,4	78,0
5	U5	Unknown	7 044 908	894 806	2 115,6	N/A	N/A	2 907	940	7 144	2 423,4	952,1	78,0
6	U6	Unknown	5 520 916	460 780	1 740,7	N/A	N/A	2 907	468	4 920	1 899,2	653,8	78,0
7	U7	Unknown	6 705 224	1 682 457	1 727,8	N/A	N/A	2 907	564	8 152	2 306,6	1 268,0	78,0
8	U8	Unknown	6 093 932	1 648 495	1 529,2	N/A	N/A	2 907	496	6 596	2 096,3	1 245,1	78,0

Image Report: Histologia 2023-01-19 14hr 17min_Exposure_16.8sec 1c pERK

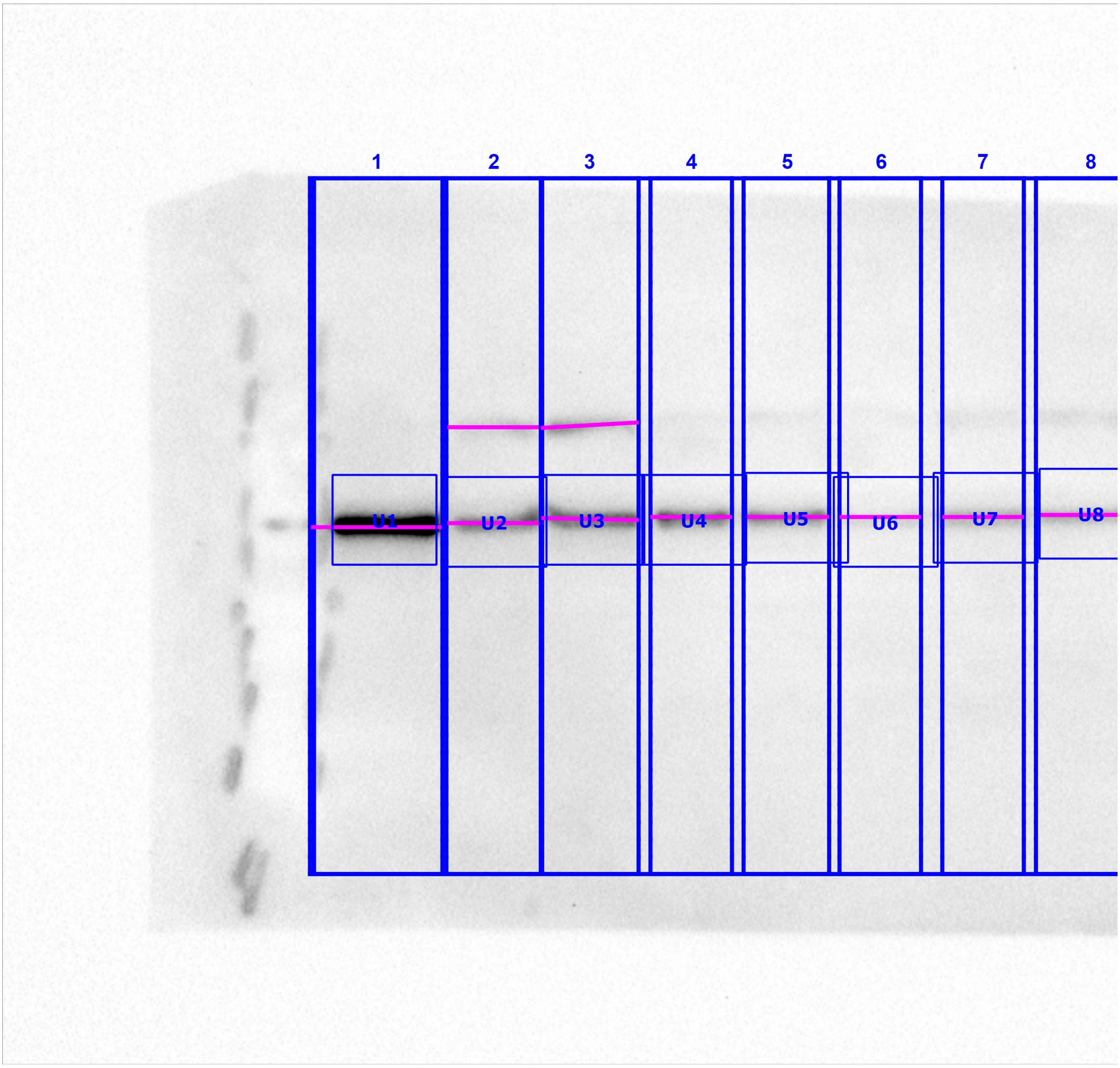

C:\Users\rusak\OneDrive\Dokumenty\Badania\CHI3L2 in BC\BC westerny ilościowo\pErK BC 19.1.23\1 bis\Histologia 2023-01-19 14hr 17min_Exposure_16.8sec 1c pERK.scn

Acquisition Information

Imager	ChemiDoc MP
Exposure Time (sec)	16.800 (Signal Accumulation)
Flat Field	Applied (Lens)

Serial Number	731BR01769
Software Version	5.0
Application	Chemi Hi Resolution
Excitation Source	No Illumination
Emission Filter	No Filter
Binning	2x2

Image Information

Acquisition Date	19/1/2023 2:18:34 PM
User Name	Histologia
Image Area (mm)	X: 114.0 Y: 85.2
Pixel Size (μm)	X: 163.8 Y: 163.8
Data Range (Int)	0 - 47668

Analysis Settings

Detection	Lane detection:<br>Manually created lanes<br><br>Band detection:<br>Automatically detected bands with sensitivity: Low<br><br>Lane Background Subtraction:<br>Lane background subtracted with disk size: 10<br><br>Lane width: Variable
Volume Analysis	Background subtraction method: Local<br>Quantity regression method: Linear

Lane Statistics

Lane No.	Adj. Total Band Vol. (Int)	Total Band Vol. (Int)	Adj. Total Lane Vol. (Int)	Total Lane Vol. (Int)	Bkgd. Vol. (Int)	Norm. Factor
1	15 577 705	18 671 315	21 677 240	54 238 210	32 560 970	N/A
2	4 299 090	8 983 486	7 189 167	32 219 346	25 030 179	N/A
3	5 483 424	7 883 760	8 215 488	31 724 880	23 509 392	N/A
4	2 968 520	3 820 160	6 198 240	20 704 040	14 505 800	N/A
5	2 522 310	3 130 848	5 783 148	19 024 026	13 240 878	N/A
6	630 480	943 600	4 234 640	16 403 600	12 168 960	N/A
7	1 627 880	2 262 320	4 895 840	18 967 880	14 072 040	N/A
8	1 790 360	2 531 705	5 944 290	26 088 755	20 144 465	N/A

Lane And Band Analysis

Lane 1

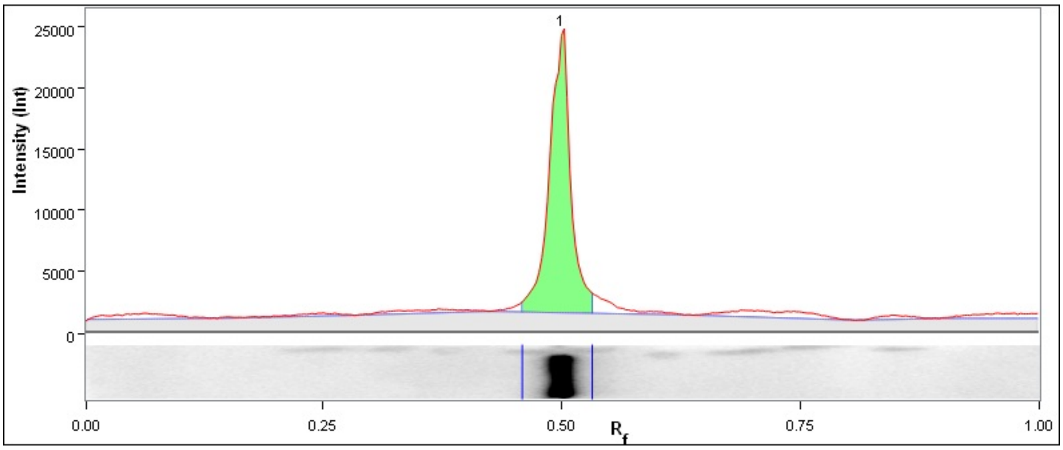

Band No.	Band Label	Mol. Wt. (KDa)	Relative Front	Adj. Volume (Int)	Volume (Int)	Abs. Quant.	Rel. Quant.	Band %	Lane %
1		N/A	0,501	15 577 705	18 671 315	N/A	N/A	100,0	71,9

Band Detection	Automatically detected bands with sensitivity: Low
Lane Background	Lane background subtracted with disk size: 10
Lane Width	10.65 mm

Lane 2

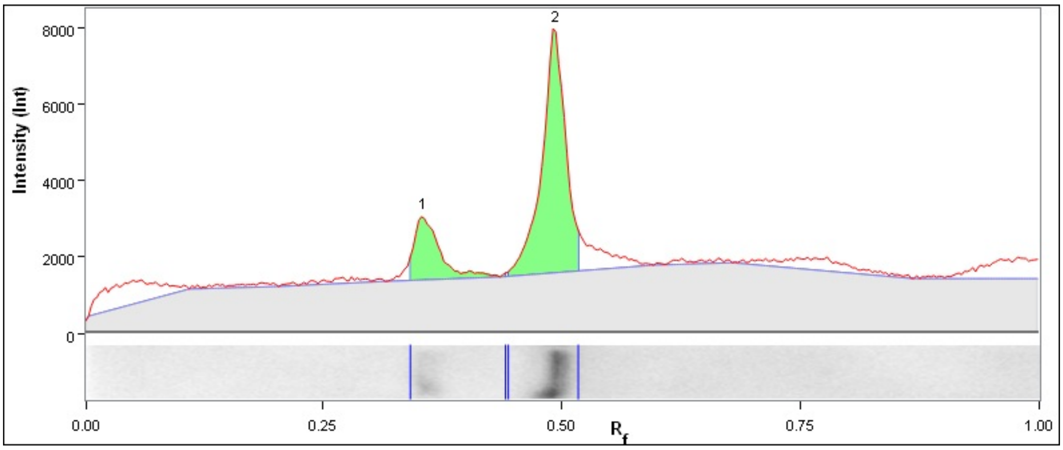

Band No.	Band Label	Mol. Wt. (KDa)	Relative Front	Adj. Volume (Int)	Volume (Int)	Abs. Quant.	Rel. Quant.	Band %	Lane %
1		N/A	0,358	950 011	3 535 481	N/A	N/A	22,1	13,2
2		N/A	0,496	3 349 079	5 448 005	N/A	N/A	77,9	46,6

Band Detection	Automatically detected bands with sensitivity: Low
Lane Background	Lane background subtracted with disk size: 10
Lane Width	7.70 mm

Lane 3

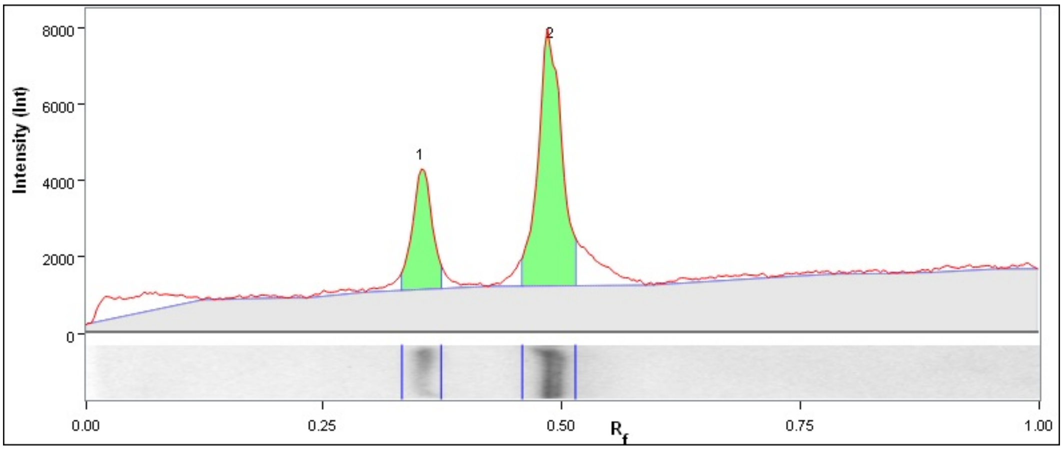

Band No.	Band Label	Mol. Wt. (KDa)	Relative Front	Adj. Volume (Int)	Volume (Int)	Abs. Quant.	Rel. Quant.	Band %	Lane %
1		N/A	0,355	1 548 384	2 529 360	N/A	N/A	28,2	18,8
2		N/A	0,490	3 935 040	5 354 400	N/A	N/A	71,8	47,9

Band Detection	Automatically detected bands with sensitivity: Low
Lane Background	Lane background subtracted with disk size: 10
Lane Width	7.86 mm

Lane 4

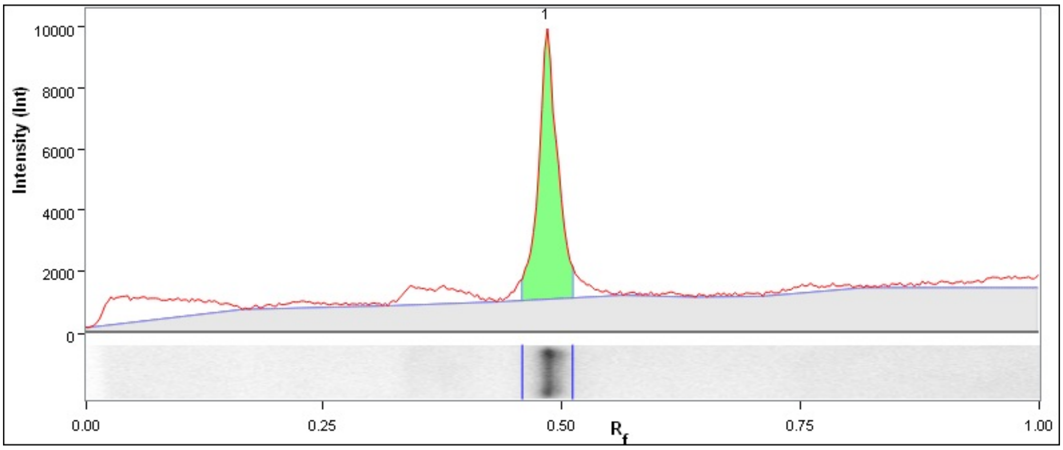

Band No.	Band Label	Mol. Wt. (KDa)	Relative Front	Adj. Volume (Int)	Volume (Int)	Abs. Quant.	Rel. Quant.	Band %	Lane %
1		N/A	0,487	2 968 520	3 820 160	N/A	N/A	100,0	47,9

Band Detection	Automatically detected bands with sensitivity: Low
Lane Background	Lane background subtracted with disk size: 10
Lane Width	6.55 mm

Lane 5

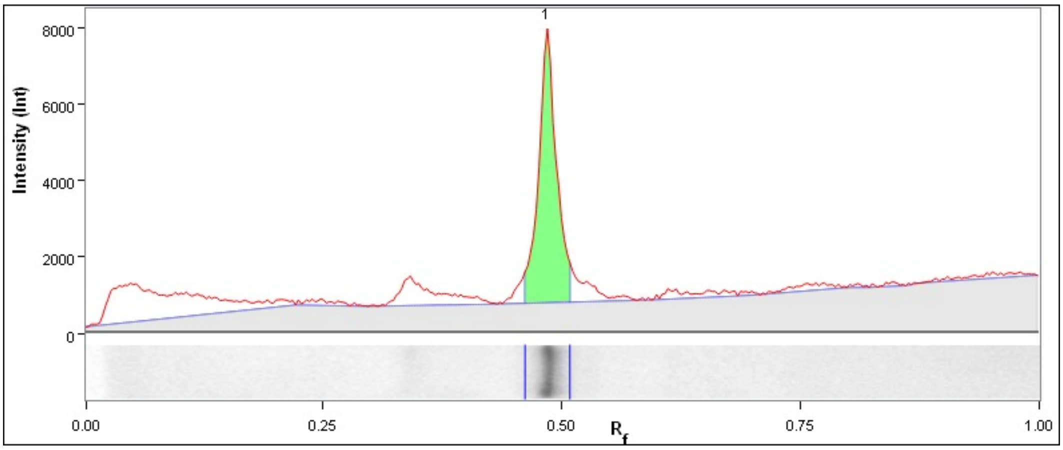

Band No.	Band Label	Mol. Wt. (KDa)	Relative Front	Adj. Volume (Int)	Volume (Int)	Abs. Quant.	Rel. Quant.	Band %	Lane %
1		N/A	0,487	2 522 310	3 130 848	N/A	N/A	100,0	43,6

Band Detection	Automatically detected bands with sensitivity: Low
Lane Background	Lane background subtracted with disk size: 10
Lane Width	6.88 mm

Lane 6

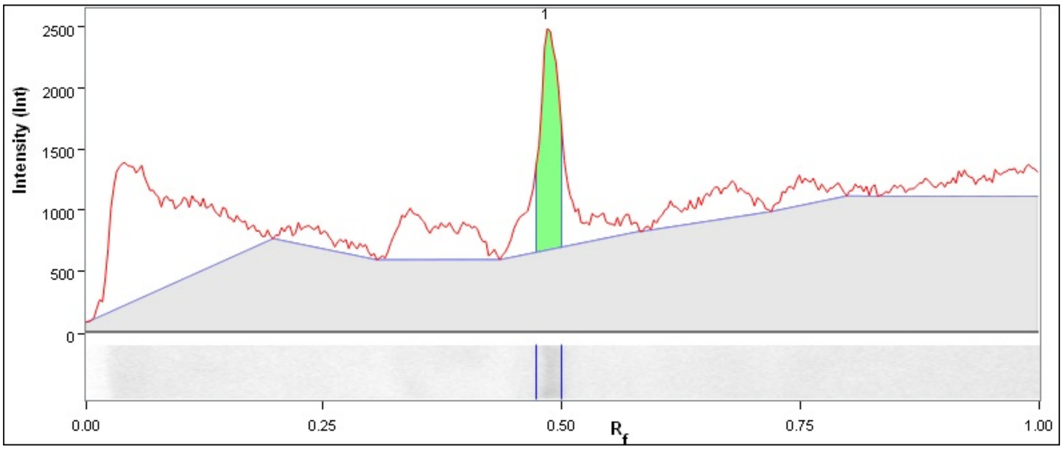

Band No.	Band Label	Mol. Wt. (KDa)	Relative Front	Adj. Volume (Int)	Volume (Int)	Abs. Quant.	Rel. Quant.	Band %	Lane %
1		N/A	0,487	630 480	943 600	N/A	N/A	100,0	14,9

Band Detection	Automatically detected bands with sensitivity: Low
Lane Background	Lane background subtracted with disk size: 10
Lane Width	6.55 mm

Lane 7

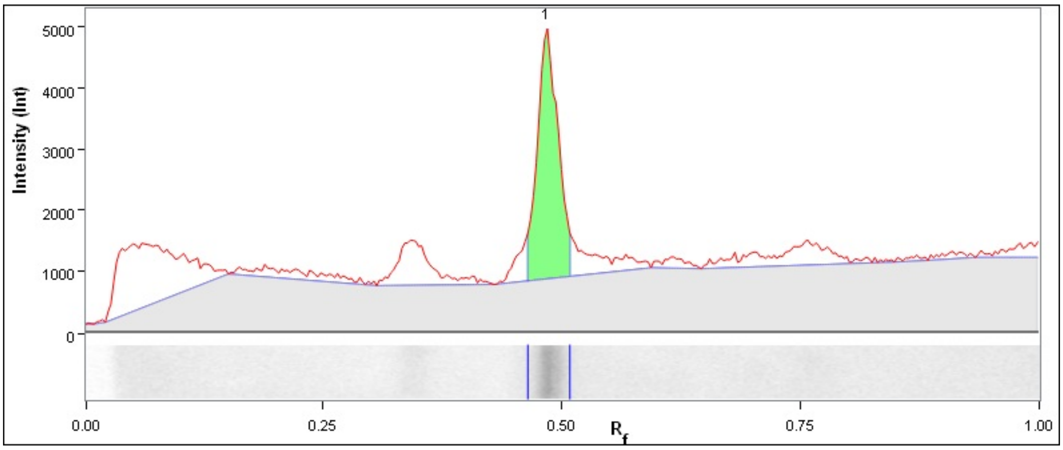

Band No.	Band Label	Mol. Wt. (KDa)	Relative Front	Adj. Volume (Int)	Volume (Int)	Abs. Quant.	Rel. Quant.	Band %	Lane %
1		N/A	0,487	1 627 880	2 262 320	N/A	N/A	100,0	33,3

Band Detection	Automatically detected bands with sensitivity: Low
Lane Background	Lane background subtracted with disk size: 10
Lane Width	6.55 mm

Lane 8

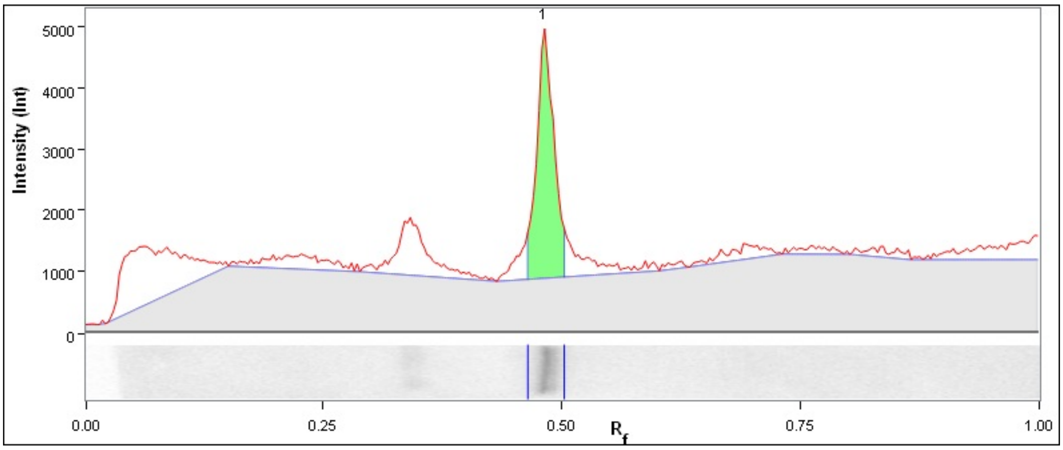

Band No.	Band Label	Mol. Wt. (KDa)	Relative Front	Adj. Volume (Int)	Volume (Int)	Abs. Quant.	Rel. Quant.	Band %	Lane %
1		N/A	0,484	1 790 360	2 531 705	N/A	N/A	100,0	30,1

Band Detection	Automatically detected bands with sensitivity: Low
Lane Background	Lane background subtracted with disk size: 10
Lane Width	9.01 mm

Volume Analysis

No.	Label	Type	Volume (Int)	Adj. Vol. (Int)	Mean Bkgd. (Int)	Abs. Quant.	Rel. Quant.	# of Pixels	Min. Value (Int)	Max. Value (Int)	Mean Value (Int)	Std. Dev.	Area (mm2)
1	U1	Unknown	19 290 736	10 909 928	3 734,8	N/A	N/A	2 244	1 156	47 668	8 596,6	9 741,6	60,2
2	U2	Unknown	7 923 184	1 556 239	2 837,3	N/A	N/A	2 244	1 016	12 064	3 530,8	2 189,1	60,2
3	U3	Unknown	8 098 032	3 165 396	2 198,1	N/A	N/A	2 244	820	11 260	3 608,7	2 473,7	60,2

4	U4	Unknown	6 177 048	2 209 887	1 767,9	N/A	N/A	2 244	280	13 964	2 752,7	2 423,7	60,2
5	U5	Unknown	4 970 552	2 145 749	1 258,8	N/A	N/A	2 244	120	10 344	2 215,0	2 035,4	60,2
6	U6	Unknown	3 069 312	306 185	1 231,3	N/A	N/A	2 244	128	4 336	1 367,8	641,4	60,2
7	U7	Unknown	4 563 096	1 474 103	1 376,6	N/A	N/A	2 244	264	6 468	2 033,5	1 274,8	60,2
8	U8	Unknown	4 302 988	1 438 719	1 276,4	N/A	N/A	2 244	280	7 192	1 917,6	1 335,8	60,2

Image Report: Histologia 2023-02-15 16hr 36min_Exposure_16.8sec 2a pERK

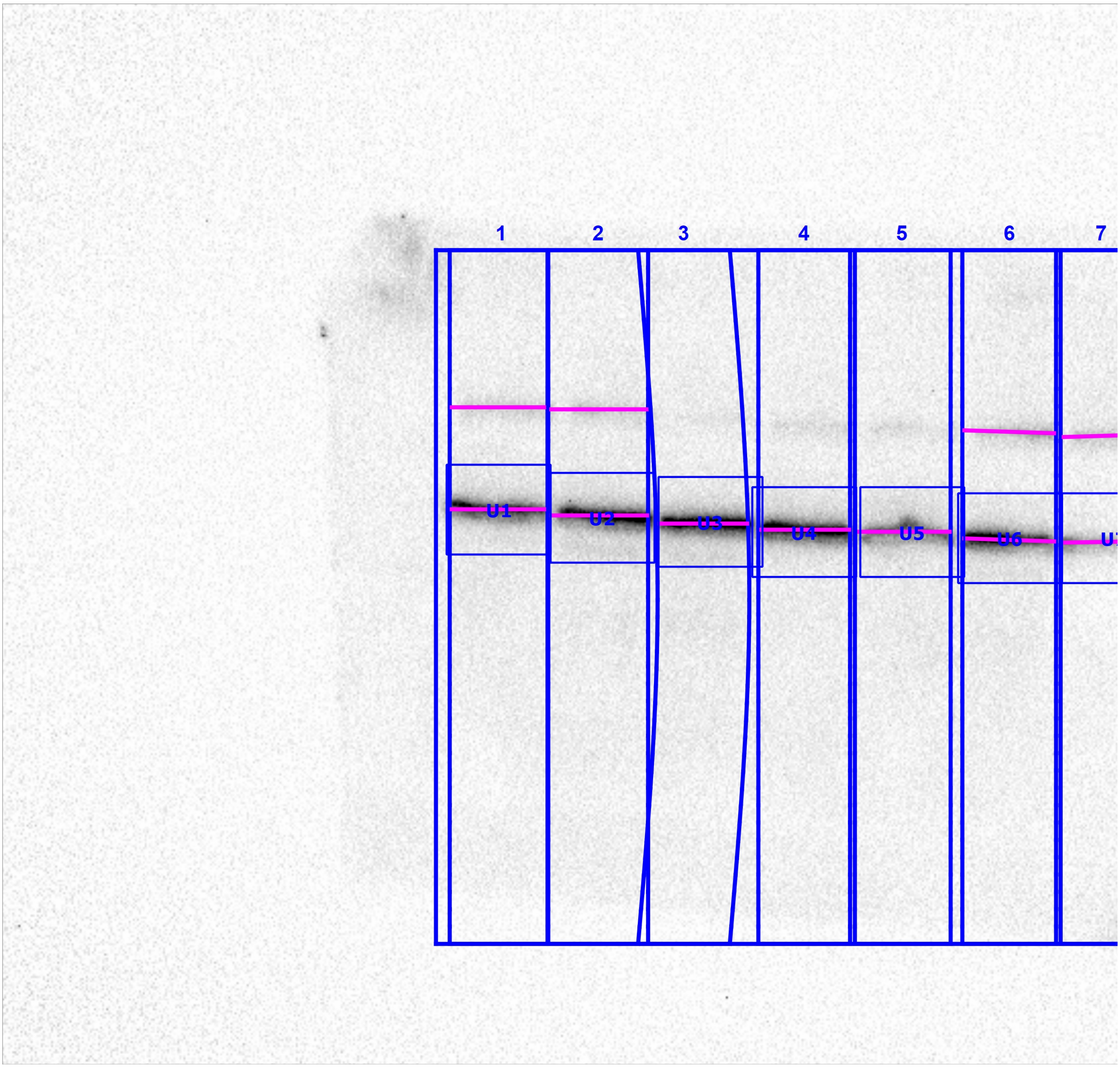

C:\Users\rusak\OneDrive\Dokumenty\Badania\CHI3L2 in BC\BC westerny ilościowo\pERK 2 BC 15.2.23\Histologia 2023-02-15 16hr 36min_Exposure_16.8sec 2a pERK.scn

Acquisition Information

Imager	ChemiDoc MP
Exposure Time (sec)	16.800 (Signal Accumulation)
Flat Field	Applied (Lens)

Serial Number	731BR01769
Software Version	5.0
Application	Chemi Hi Resolution
Excitation Source	No Illumination
Emission Filter	No Filter
Binning	2x2

Image Information

Acquisition Date	15/2/2023 4:37:27 PM
User Name	Histologia
Image Area (mm)	X: 114.0 Y: 85.2
Pixel Size (μm)	X: 163.8 Y: 163.8
Data Range (Int)	0 - 8684

Analysis Settings

Detection	Lane detection:<br>Manually created lanes<br><br>Band detection:<br>Automatically detected bands with sensitivity: Low<br><br>Lane Background Subtraction:<br>Lane background subtracted with disk size: 10<br><br>Lane width: Variable
Volume Analysis	Background subtraction method: Local<br>Quantity regression method: Linear

Lane Statistics

Lane No.	Adj. Total Band Vol. (Int)	Total Band Vol. (Int)	Adj. Total Lane Vol. (Int)	Total Lane Vol. (Int)	Bkgd. Vol. (Int)	Norm. Factor
1	2 237 424	2 858 352	3 600 480	7 750 944	4 150 464	N/A
2	2 652 174	3 300 444	3 969 784	8 743 266	4 773 482	N/A
3	2 510 370	2 962 305	3 893 175	8 178 570	4 285 395	N/A
4	2 424 870	2 830 365	3 513 690	7 706 835	4 193 145	N/A
5	1 477 304	1 833 470	2 818 825	6 975 975	4 157 150	N/A
6	2 480 688	3 032 458	3 648 352	7 808 224	4 159 872	N/A
7	1 019 137	1 308 351	1 958 939	4 879 369	2 920 430	N/A

Lane And Band Analysis

Lane 1

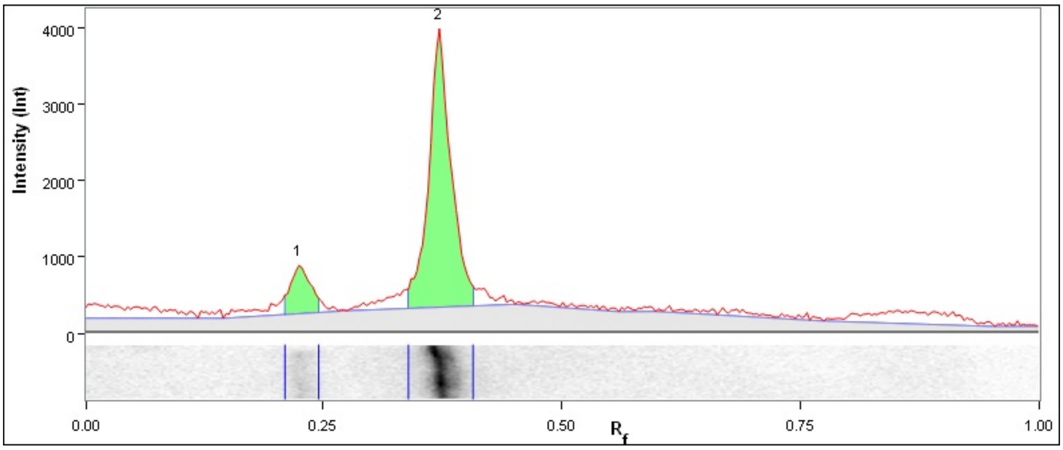

Band No.	Band Label	Mol. Wt. (KDa)	Relative Front	Adj. Volume (Int)	Volume (Int)	Abs. Quant.	Rel. Quant.	Band %	Lane %
1		N/A	0,226	303 216	481 296	N/A	N/A	13,6	8,4
2		N/A	0,374	1 934 208	2 377 056	N/A	N/A	86,4	53,7

Band Detection	Automatically detected bands with sensitivity: Low
Lane Background	Lane background subtracted with disk size: 10
Lane Width	7.86 mm

Lane 2

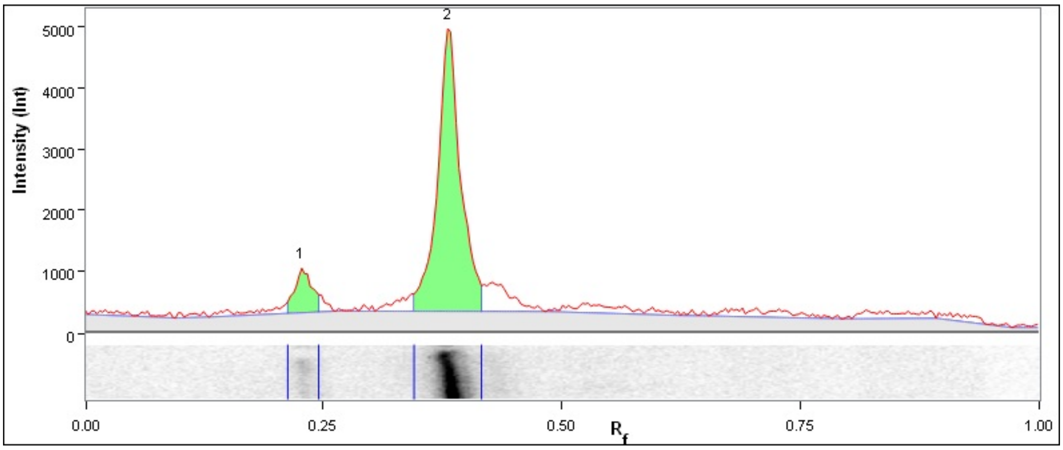

Band No.	Band Label	Mol. Wt. (KDa)	Relative Front	Adj. Volume (Int)	Volume (Int)	Abs. Quant.	Rel. Quant.	Band %	Lane %
1		N/A	0,229	280 133	480 739	N/A	N/A	10,6	7,1
2		N/A	0,382	2 372 041	2 819 705	N/A	N/A	89,4	59,8

Band Detection	Automatically detected bands with sensitivity: Low
Lane Background	Lane background subtracted with disk size: 10
Lane Width	8.03 mm

Lane 3

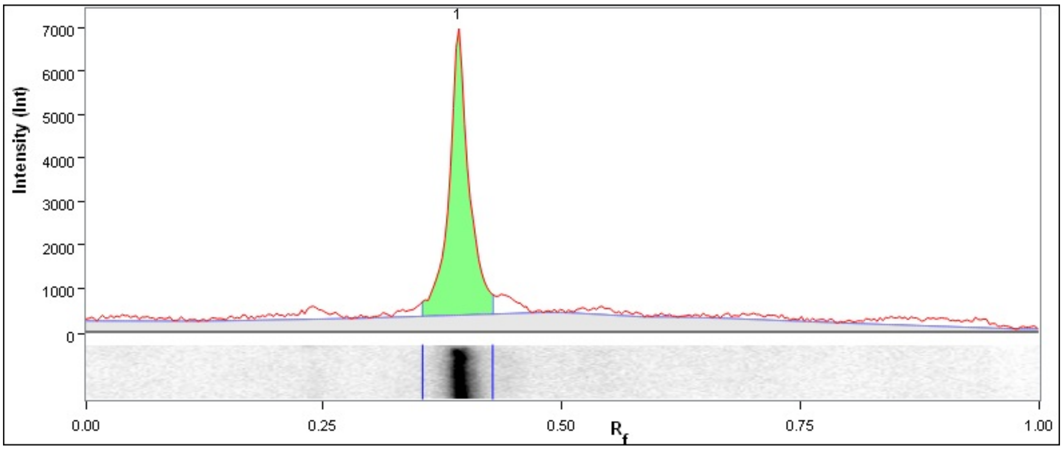

Band No.	Band Label	Mol. Wt. (KDa)	Relative Front	Adj. Volume (Int)	Volume (Int)	Abs. Quant.	Rel. Quant.	Band %	Lane %
1		N/A	0,394	2 510 370	2 962 305	N/A	N/A	100,0	64,5

Band Detection	Automatically detected bands with sensitivity: Low
Lane Background	Lane background subtracted with disk size: 10
Lane Width	7.37 mm

Lane 4

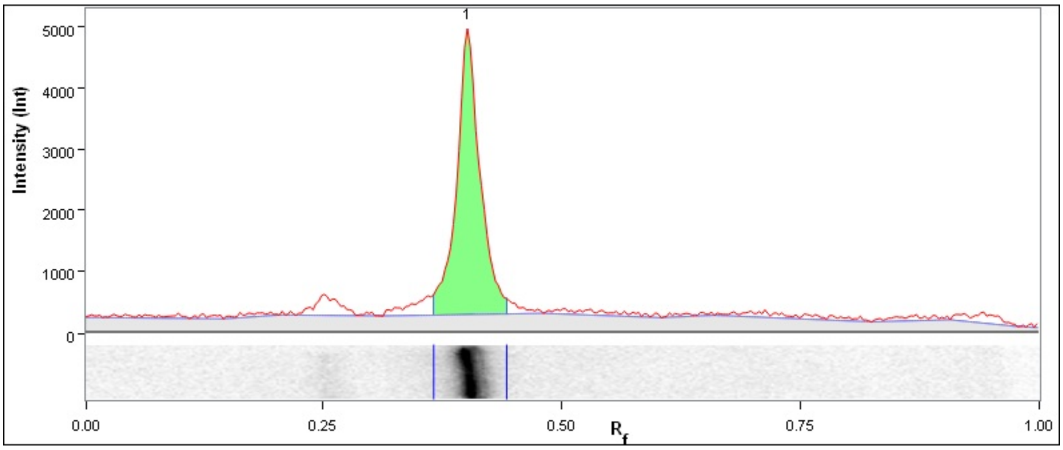

Band No.	Band Label	Mol. Wt. (KDa)	Relative Front	Adj. Volume (Int)	Volume (Int)	Abs. Quant.	Rel. Quant.	Band %	Lane %
1		N/A	0,403	2 424 870	2 830 365	N/A	N/A	100,0	69,0

Band Detection	Automatically detected bands with sensitivity: Low
Lane Background	Lane background subtracted with disk size: 10
Lane Width	7.37 mm

Lane 5

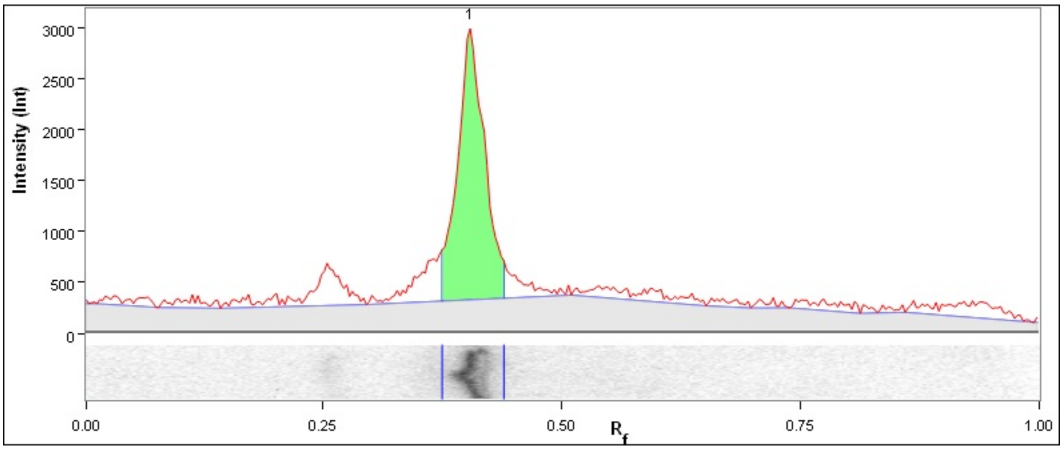

Band No.	Band Label	Mol. Wt. (KDa)	Relative Front	Adj. Volume (Int)	Volume (Int)	Abs. Quant.	Rel. Quant.	Band %	Lane %
1		N/A	0,406	1 477 304	1 833 470	N/A	N/A	100,0	52,4

Band Detection	Automatically detected bands with sensitivity: Low
Lane Background	Lane background subtracted with disk size: 10
Lane Width	7.70 mm

Lane 6

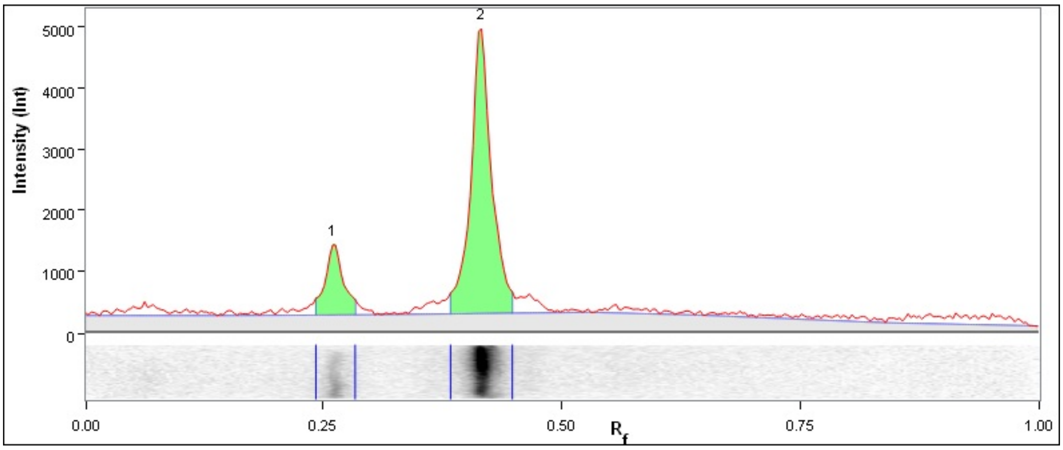

Band No.	Band Label	Mol. Wt. (KDa)	Relative Front	Adj. Volume (Int)	Volume (Int)	Abs. Quant.	Rel. Quant.	Band %	Lane %
1		N/A	0,262	440 910	648 094	N/A	N/A	17,8	12,1
2		N/A	0,418	2 039 778	2 384 364	N/A	N/A	82,2	55,9

Band Detection	Automatically detected bands with sensitivity: Low
Lane Background	Lane background subtracted with disk size: 10
Lane Width	7.53 mm

Lane 7

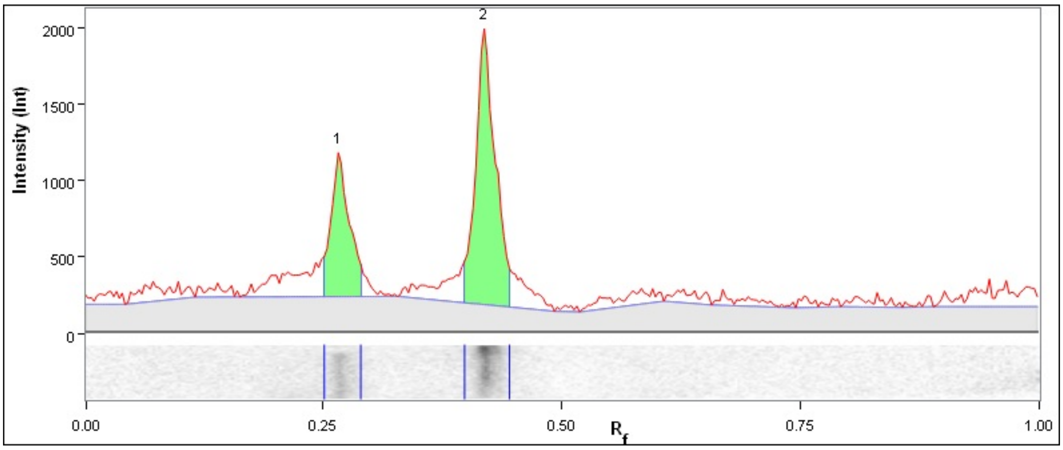

Band No.	Band Label	Mol. Wt. (KDa)	Relative Front	Adj. Volume (Int)	Volume (Int)	Abs. Quant.	Rel. Quant.	Band %	Lane %
1		N/A	0,268	334 273	484 251	N/A	N/A	32,8	17,1
2		N/A	0,421	684 864	824 100	N/A	N/A	67,2	35,0

Band Detection	Automatically detected bands with sensitivity: Low
Lane Background	Lane background subtracted with disk size: 10
Lane Width	6.72 mm

Volume Analysis

No.	Label	Type	Volume (Int)	Adj. Vol. (Int)	Mean Bkgd. (Int)	Abs. Quant.	Rel. Quant.	# of Pixels	Min. Value (Int)	Max. Value (Int)	Mean Value (Int)	Std. Dev.	Area (mm2)
1	U1	Unknown	3 051 676	1 607 234	643,7	N/A	N/A	2 244	0	6 364	1 359,9	1 262,4	60,2
2	U2	Unknown	3 628 264	2 017 859	717,6	N/A	N/A	2 244	0	7 976	1 616,9	1 553,4	60,2
3	U3	Unknown	3 849 008	1 967 472	838,5	N/A	N/A	2 244	0	8 684	1 715,2	1 764,0	60,2
4	U4	Unknown	3 526 936	1 885 531	731,5	N/A	N/A	2 244	0	7 316	1 571,7	1 575,2	60,2
5	U5	Unknown	2 574 848	909 245	742,2	N/A	N/A	2 244	0	4 796	1 147,4	893,4	60,2
6	U6	Unknown	3 091 784	1 752 972	596,6	N/A	N/A	2 244	0	8 008	1 377,8	1 461,0	60,2
7	U7	Unknown	1 319 508	494 780	367,5	N/A	N/A	2 244	0	4 008	588,0	592,2	60,2

Image Report: Histologia 2023-02-15 16hr 39min_Exposure_16.8sec 3a pERK

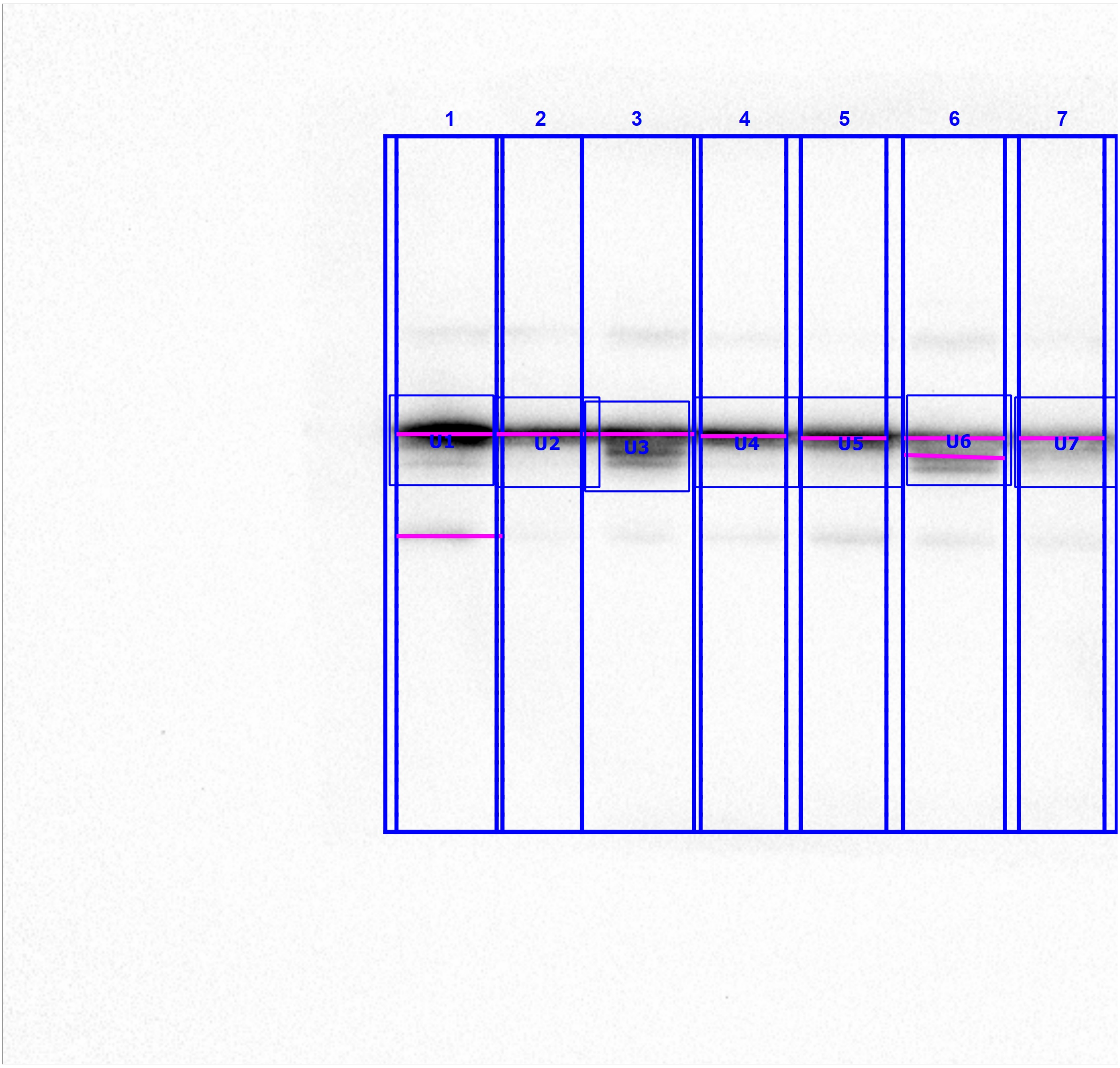

C:\Users\rusak\OneDrive\Dokumenty\Badania\CHI3L2 in BC\BC westerny ilościowo\pERk 3 BC 15.2.23\Histologia 2023-02-15 16hr 39min_Exposure_16.8sec 3a pERK.scn

Acquisition Information

Imager	ChemiDoc MP
Exposure Time (sec)	16.800 (Signal Accumulation)
Flat Field	Applied (Lens)

Serial Number	731BR01769
Software Version	5.0
Application	Chemi Hi Resolution
Excitation Source	No Illumination
Emission Filter	No Filter
Binning	2x2

Image Information

Acquisition Date	15/2/2023 4:39:29 PM
User Name	Histologia
Image Area (mm)	X: 114.0 Y: 85.2
Pixel Size (μm)	X: 163.8 Y: 163.8
Data Range (Int)	0 - 65535

Analysis Settings

Detection	Lane detection:<br>Manually created lanes<br><br>Band detection:<br>Automatically detected bands with sensitivity: Low<br><br>Lane Background Subtraction:<br>Lane background subtracted with disk size: 10<br><br>Lane width: Variable
Volume Analysis	Background subtraction method: Local<br>Quantity regression method: Linear

Lane Statistics

Lane No.	Adj. Total Band Vol. (Int)	Total Band Vol. (Int)	Adj. Total Lane Vol. (Int)	Total Lane Vol. (Int)	Bkgd. Vol. (Int)	Norm. Factor
1	13 203 528	14 445 912	14 698 424	20 166 848	5 468 424	N/A
2	5 479 614	5 833 926	7 385 364	10 417 092	3 031 728	N/A
3	10 872 510	11 390 390	13 115 190	16 384 280	3 269 090	N/A
4	5 772 942	5 991 804	7 641 732	9 816 744	2 175 012	N/A
5	6 184 668	6 464 094	8 125 698	10 501 008	2 375 310	N/A
6	6 950 350	7 436 500	9 061 800	12 210 750	3 148 950	N/A
7	4 034 940	4 399 668	5 760 468	8 942 598	3 182 130	N/A

Lane And Band Analysis

Lane 1

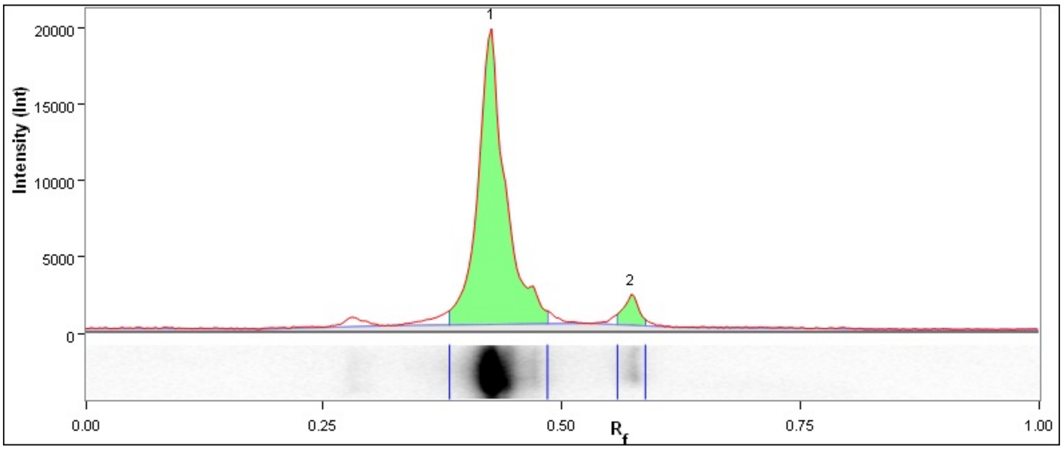

Band No.	Band Label	Mol. Wt. (KDa)	Relative Front	Adj. Volume (Int)	Volume (Int)	Abs. Quant.	Rel. Quant.	Band %	Lane %
1		N/A	0,428	12 484 212	13 460 928	N/A	N/A	94,6	84,9
2		N/A	0,575	719 316	984 984	N/A	N/A	5,4	4,9

Band Detection	Automatically detected bands with sensitivity: Low
Lane Background	Lane background subtracted with disk size: 10
Lane Width	8.52 mm

Lane 2

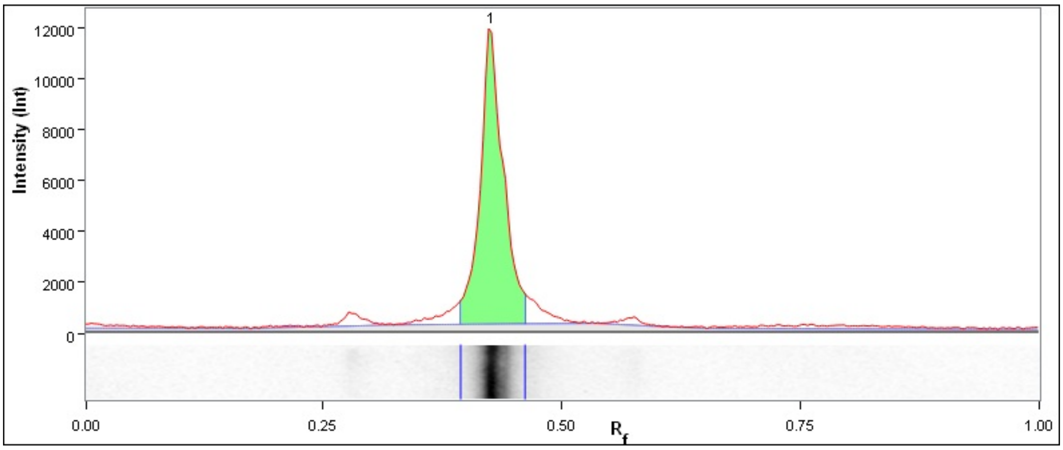

Band No.	Band Label	Mol. Wt. (KDa)	Relative Front	Adj. Volume (Int)	Volume (Int)	Abs. Quant.	Rel. Quant.	Band %	Lane %
1		N/A	0,428	5 479 614	5 833 926	N/A	N/A	100,0	74,2

Band Detection	Automatically detected bands with sensitivity: Low
Lane Background	Lane background subtracted with disk size: 10
Lane Width	6.88 mm

Lane 3

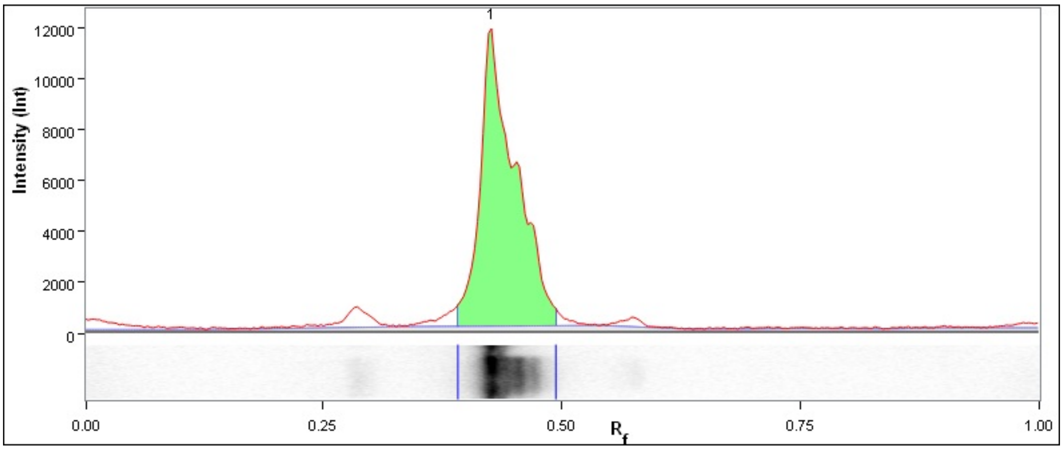

Band No.	Band Label	Mol. Wt. (KDa)	Relative Front	Adj. Volume (Int)	Volume (Int)	Abs. Quant.	Rel. Quant.	Band %	Lane %
1		N/A	0,428	10 872 510	11 390 390	N/A	N/A	100,0	82,9

Band Detection	Automatically detected bands with sensitivity: Low
Lane Background	Lane background subtracted with disk size: 10
Lane Width	9.01 mm

Lane 4

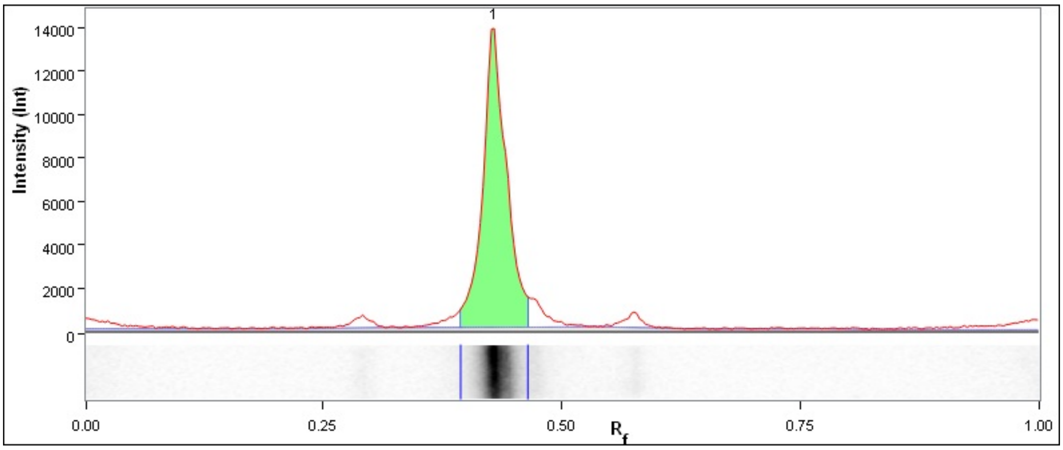

Band No.	Band Label	Mol. Wt. (KDa)	Relative Front	Adj. Volume (Int)	Volume (Int)	Abs. Quant.	Rel. Quant.	Band %	Lane %
1		N/A	0,431	5 772 942	5 991 804	N/A	N/A	100,0	75,5

Band Detection	Automatically detected bands with sensitivity: Low
Lane Background	Lane background subtracted with disk size: 10
Lane Width	6.88 mm

Lane 5

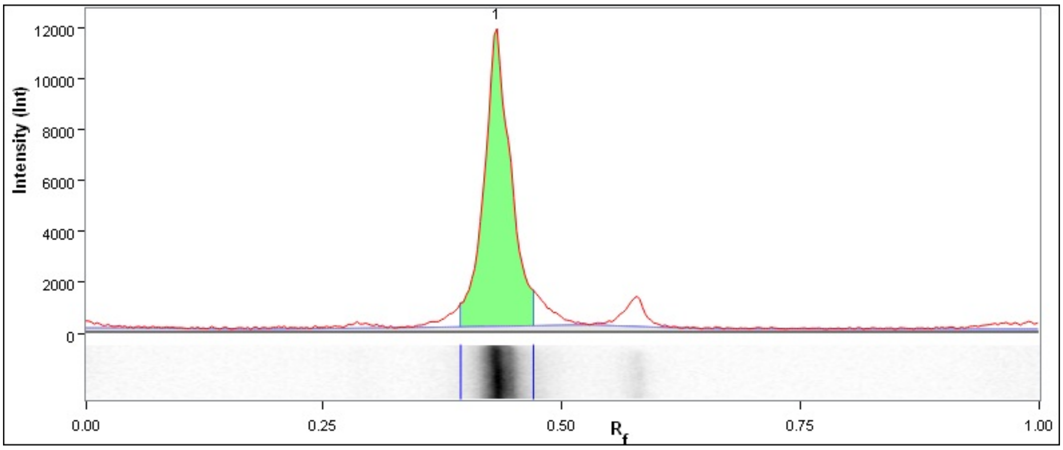

Band No.	Band Label	Mol. Wt. (KDa)	Relative Front	Adj. Volume (Int)	Volume (Int)	Abs. Quant.	Rel. Quant.	Band %	Lane %
1		N/A	0,434	6 184 668	6 464 094	N/A	N/A	100,0	76,1

Band Detection	Automatically detected bands with sensitivity: Low
Lane Background	Lane background subtracted with disk size: 10
Lane Width	6.88 mm

Lane 6

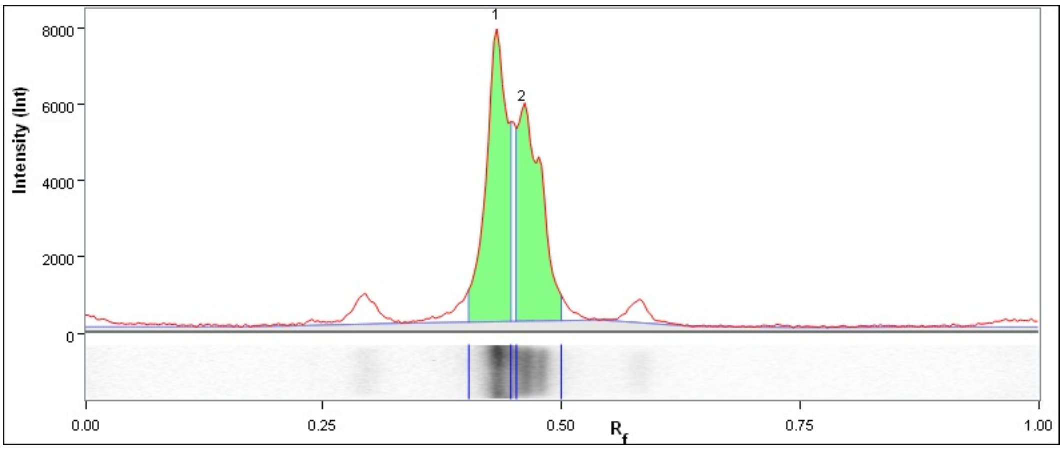

Band No.	Band Label	Mol. Wt. (KDa)	Relative Front	Adj. Volume (Int)	Volume (Int)	Abs. Quant.	Rel. Quant.	Band %	Lane %
1		N/A	0,434	3 731 650	3 957 800	N/A	N/A	53,7	41,2
2		N/A	0,460	3 218 700	3 478 700	N/A	N/A	46,3	35,5

Band Detection	Automatically detected bands with sensitivity: Low
Lane Background	Lane background subtracted with disk size: 10
Lane Width	8.19 mm

Lane 7

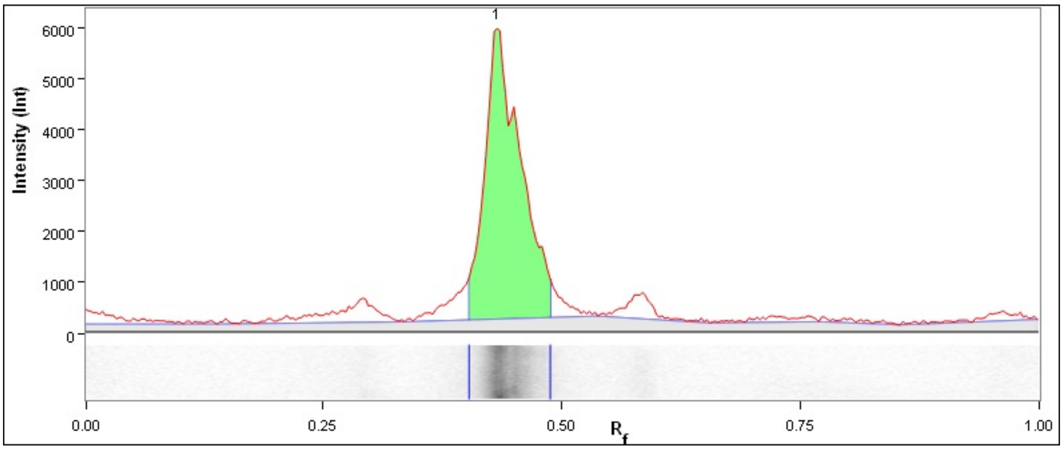

Band No.	Band Label	Mol. Wt. (KDa)	Relative Front	Adj. Volume (Int)	Volume (Int)	Abs. Quant.	Rel. Quant.	Band %	Lane %
1		N/A	0,434	4 034 940	4 399 668	N/A	N/A	100,0	70,0

Band Detection	Automatically detected bands with sensitivity: Low
Lane Background	Lane background subtracted with disk size: 10
Lane Width	6.88 mm

Volume Analysis

No.	Label	Type	Volume (Int)	Adj. Vol. (Int)	Mean Bkgd. (Int)	Abs. Quant.	Rel. Quant.	# of Pixels	Min. Value (Int)	Max. Value (Int)	Mean Value (Int)	Std. Dev.	Area (mm2)
1	U1	Unknown	13 521 596	9 715 263	1 696,2	N/A	N/A	2 244	204	28 404	6 025,7	6 389,5	60,2
2	U2	Unknown	7 998 088	3 449 384	2 027,1	N/A	N/A	2 244	0	15 444	3 564,2	3 775,3	60,2
3	U3	Unknown	11 121 344	6 798 937	1 926,2	N/A	N/A	2 244	0	17 100	4 956,0	4 186,4	60,2
4	U4	Unknown	7 699 604	4 360 301	1 488,1	N/A	N/A	2 244	0	16 260	3 431,2	3 864,7	60,2
5	U5	Unknown	8 412 364	5 143 249	1 456,8	N/A	N/A	2 244	0	15 728	3 748,8	3 765,1	60,2
6	U6	Unknown	8 058 520	4 801 389	1 451,5	N/A	N/A	2 244	0	11 036	3 591,1	2 685,3	60,2
7	U7	Unknown	5 664 636	3 277 807	1 063,6	N/A	N/A	2 244	0	9 092	2 524,3	1 932,3	60,2

Image Report: Histologia 2023-01-19 14hr 15min_Exposure_16.8sec 3c pERK

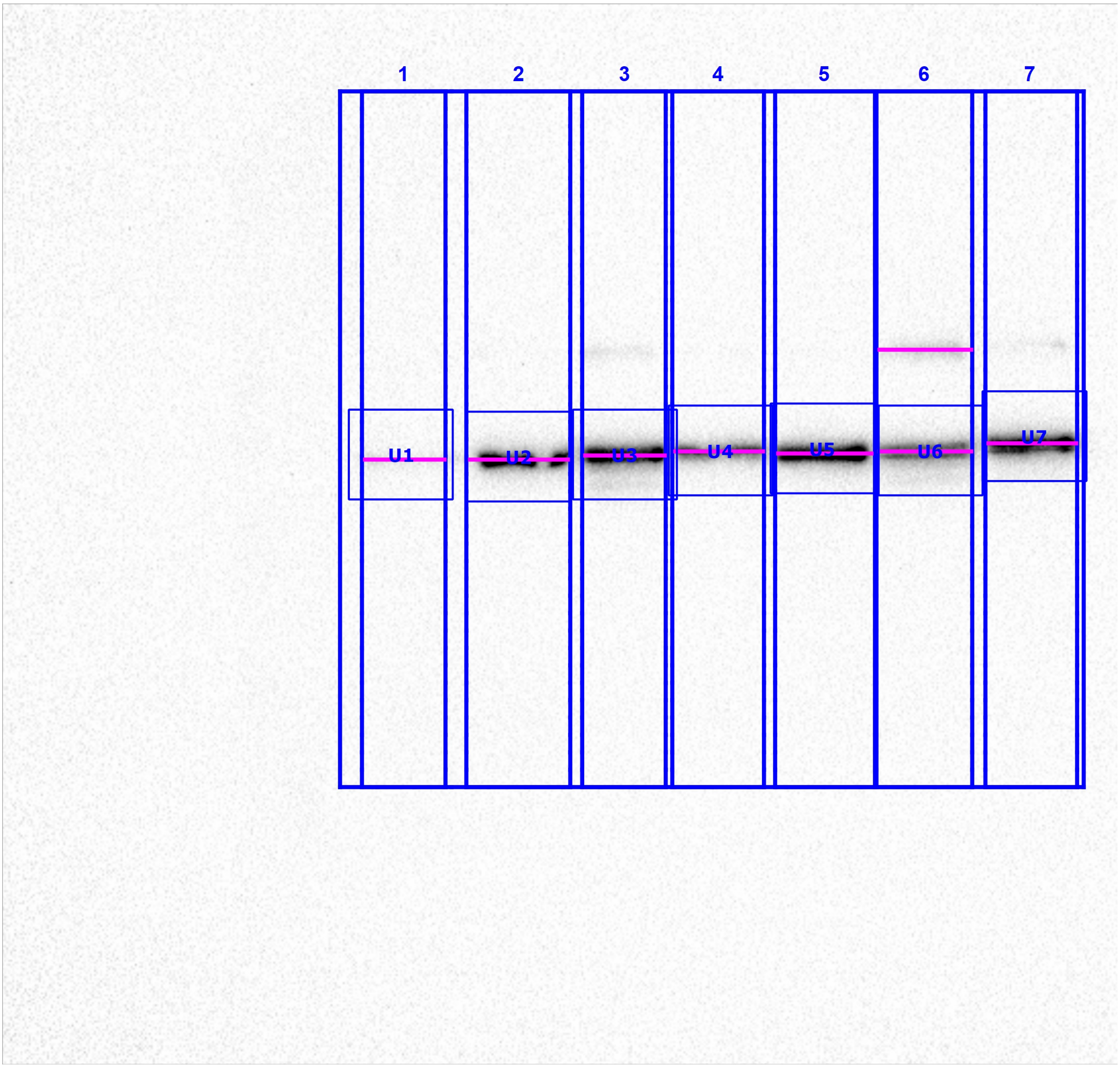

C:\Users\rusak\OneDrive\Dokumenty\Badania\CHI3L2 in BC\BC westerny ilościowo\pErK BC 19.1.23\3\Histologia 2023-01-19 14hr 15min_Exposure_16.8sec 3c pERK.scn

Acquisition Information

Imager	ChemiDoc MP
Exposure Time (sec)	16.800 (Signal Accumulation)
Flat Field	Applied (Lens)

Serial Number	731BR01769
Software Version	5.0
Application	Chemi Hi Resolution
Excitation Source	No Illumination
Emission Filter	No Filter
Binning	2x2

Image Information

Acquisition Date	19/1/2023 2:16:27 PM
User Name	Histologia
Image Area (mm)	X: 114.0 Y: 85.2
Pixel Size (μm)	X: 163.8 Y: 163.8
Data Range (Int)	0 - 12900

Analysis Settings

Detection	Lane detection:<br>Manually created lanes<br><br>Band detection:<br>Automatically detected bands with sensitivity: Low<br><br>Lane Background Subtraction:<br>Lane background subtracted with disk size: 10<br><br>Lane width: Variable
Volume Analysis	Background subtraction method: Local<br>Quantity regression method: Linear

Lane Statistics

Lane No.	Adj. Total Band Vol. (Int)	Total Band Vol. (Int)	Adj. Total Lane Vol. (Int)	Total Lane Vol. (Int)	Bkgd. Vol. (Int)	Norm. Factor
1	193 561	233 864	840 582	2 167 998	1 327 416	N/A
2	2 956 674	3 071 883	3 613 503	5 223 216	1 609 713	N/A
3	3 280 287	3 399 064	3 967 160	5 070 347	1 103 187	N/A
4	1 808 955	1 889 010	2 411 235	3 522 330	1 111 095	N/A
5	3 556 812	3 673 187	4 177 397	5 568 850	1 391 453	N/A
6	2 888 291	3 090 955	3 468 177	4 865 299	1 397 122	N/A
7	3 389 490	3 493 980	4 172 175	5 382 000	1 209 825	N/A

Lane And Band Analysis

Lane 1

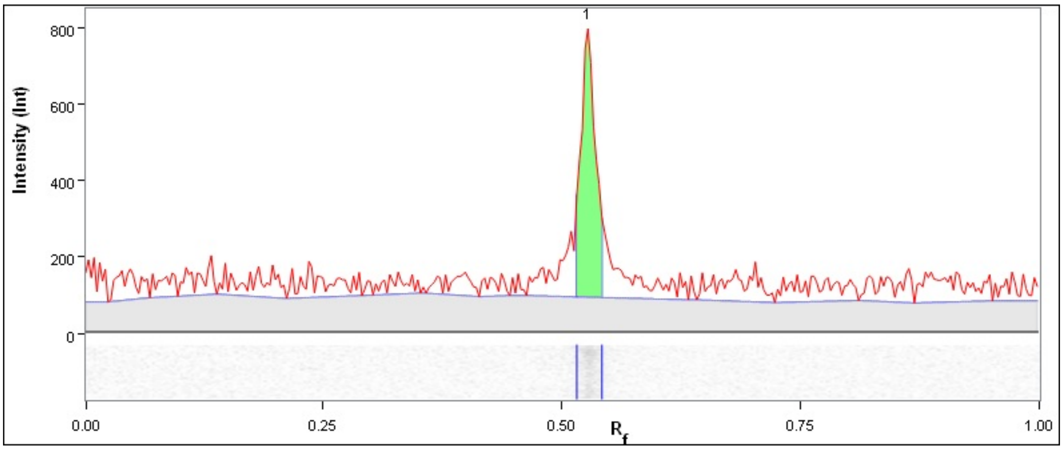

Band No.	Band Label	Mol. Wt. (KDa)	Relative Front	Adj. Volume (Int)	Volume (Int)	Abs. Quant.	Rel. Quant.	Band %	Lane %
1		N/A	0,529	193 561	233 864	N/A	N/A	100,0	23,0

Band Detection	Automatically detected bands with sensitivity: Low
Lane Background	Lane background subtracted with disk size: 10
Lane Width	6.72 mm

Lane 2

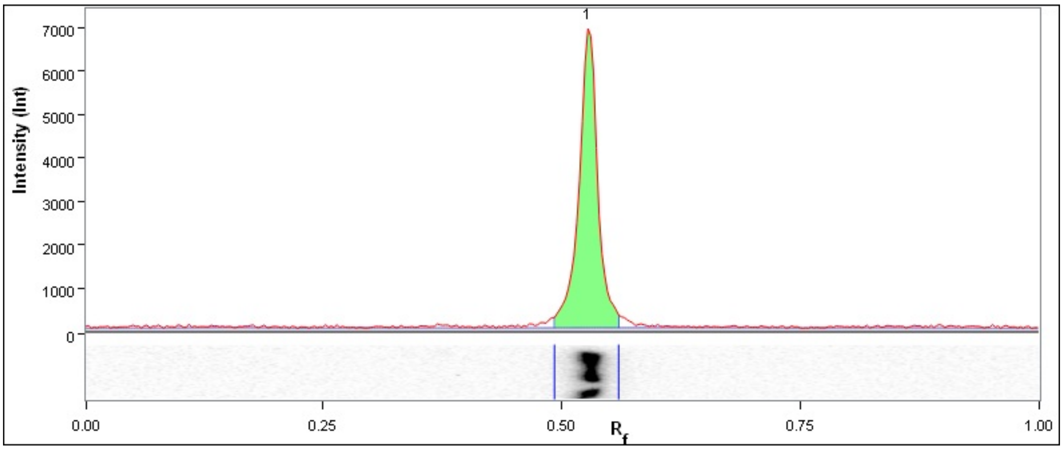

Band No.	Band Label	Mol. Wt. (KDa)	Relative Front	Adj. Volume (Int)	Volume (Int)	Abs. Quant.	Rel. Quant.	Band %	Lane %
1		N/A	0,529	2 956 674	3 071 883	N/A	N/A	100,0	81,8

Band Detection	Automatically detected bands with sensitivity: Low
Lane Background	Lane background subtracted with disk size: 10
Lane Width	8.35 mm

Lane 3

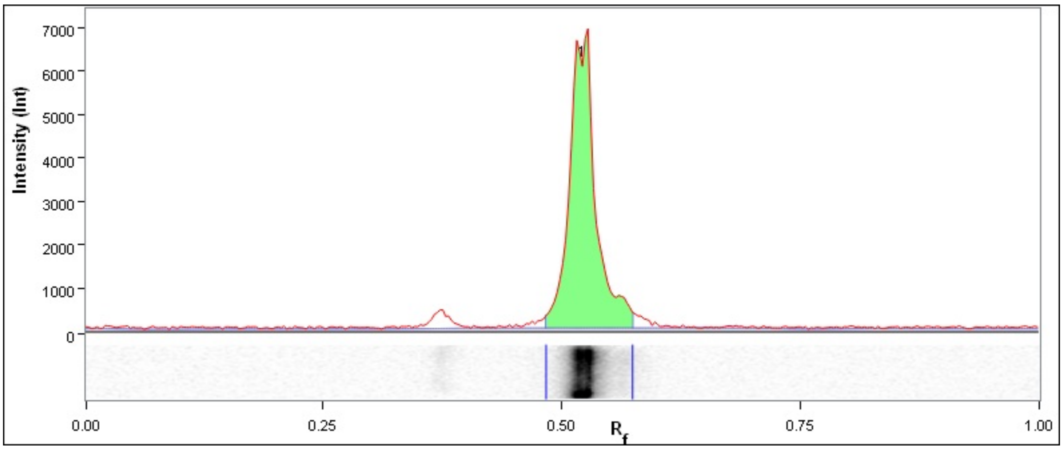

Band No.	Band Label	Mol. Wt. (KDa)	Relative Front	Adj. Volume (Int)	Volume (Int)	Abs. Quant.	Rel. Quant.	Band %	Lane %
1		N/A	0,523	3 280 287	3 399 064	N/A	N/A	100,0	82,7

Band Detection	Automatically detected bands with sensitivity: Low
Lane Background	Lane background subtracted with disk size: 10
Lane Width	6.72 mm

Lane 4

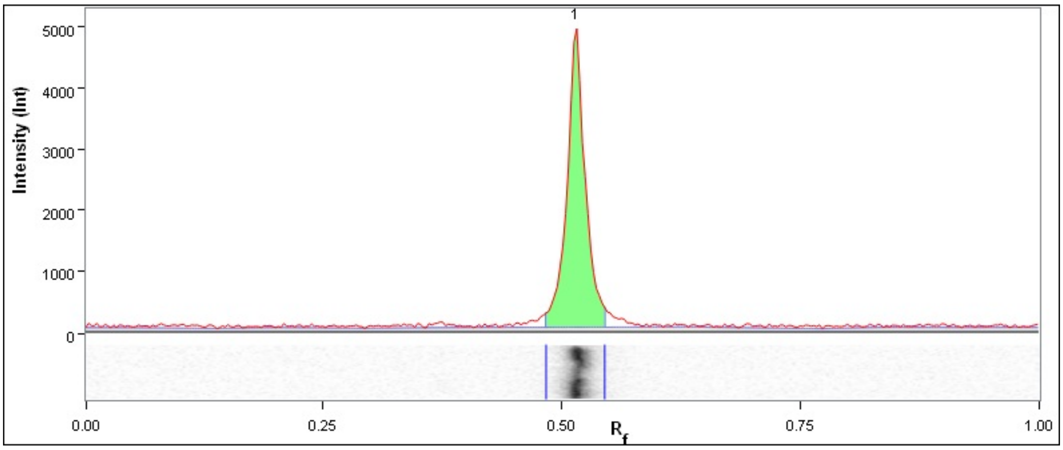

Band No.	Band Label	Mol. Wt. (KDa)	Relative Front	Adj. Volume (Int)	Volume (Int)	Abs. Quant.	Rel. Quant.	Band %	Lane %
1		N/A	0,518	1 808 955	1 889 010	N/A	N/A	100,0	75,0

Band Detection	Automatically detected bands with sensitivity: Low
Lane Background	Lane background subtracted with disk size: 10
Lane Width	7.37 mm

Lane 5

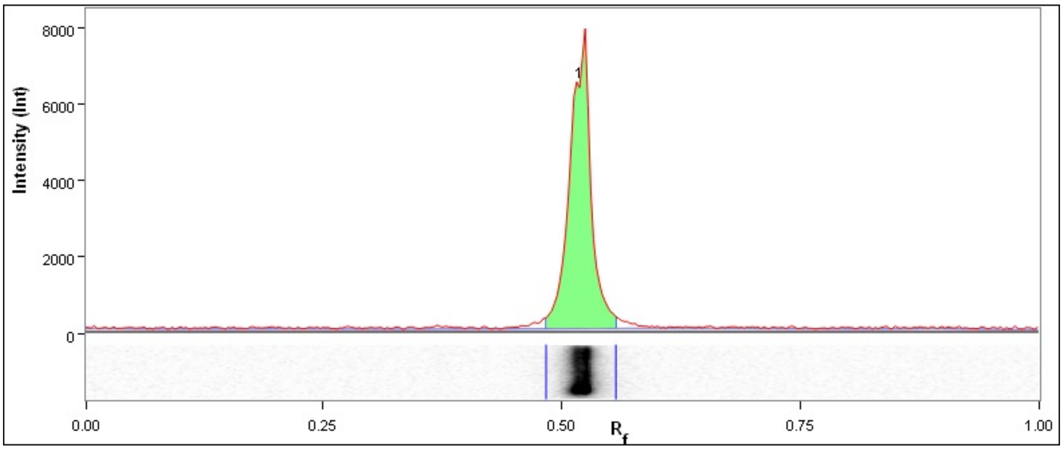

Band No.	Band Label	Mol. Wt. (KDa)	Relative Front	Adj. Volume (Int)	Volume (Int)	Abs. Quant.	Rel. Quant.	Band %	Lane %
1		N/A	0,520	3 556 812	3 673 187	N/A	N/A	100,0	85,1

Band Detection	Automatically detected bands with sensitivity: Low
Lane Background	Lane background subtracted with disk size: 10
Lane Width	8.03 mm

Lane 6

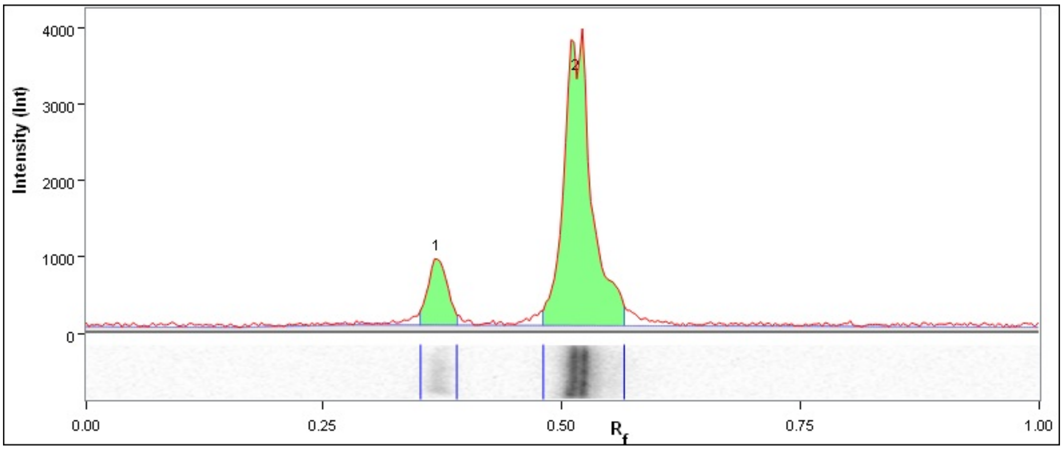

Band No.	Band Label	Mol. Wt. (KDa)	Relative Front	Adj. Volume (Int)	Volume (Int)	Abs. Quant.	Rel. Quant.	Band %	Lane %
1		N/A	0,371	402 790	470 235	N/A	N/A	13,9	11,6
2		N/A	0,518	2 485 501	2 620 720	N/A	N/A	86,1	71,7

Band Detection	Automatically detected bands with sensitivity: Low
Lane Background	Lane background subtracted with disk size: 10
Lane Width	7.70 mm

Lane 7

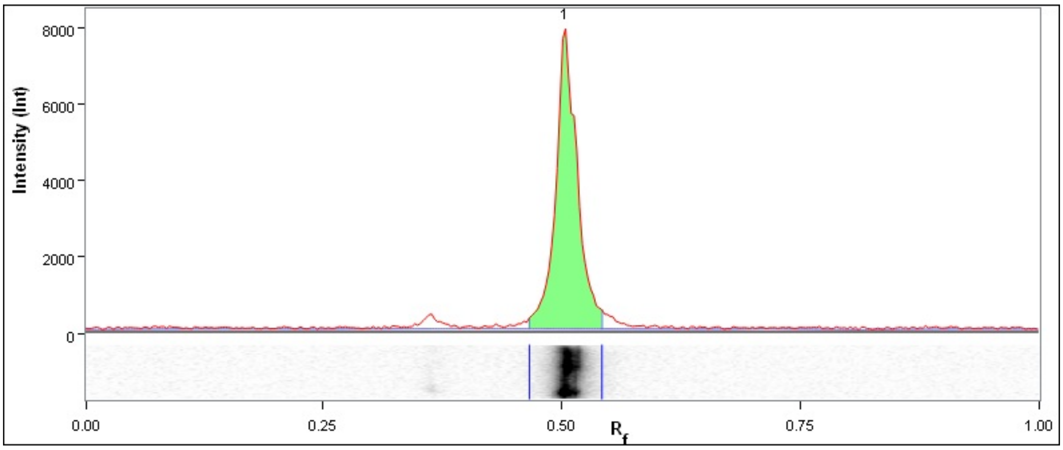

Band No.	Band Label	Mol. Wt. (KDa)	Relative Front	Adj. Volume (Int)	Volume (Int)	Abs. Quant.	Rel. Quant.	Band %	Lane %
1		N/A	0,506	3 389 490	3 493 980	N/A	N/A	100,0	81,2

Band Detection	Automatically detected bands with sensitivity: Low
Lane Background	Lane background subtracted with disk size: 10
Lane Width	7.37 mm

Volume Analysis

No.	Label	Type	Volume (Int)	Adj. Vol. (Int)	Mean Bkgd. (Int)	Abs. Quant.	Rel. Quant.	# of Pixels	Min. Value (Int)	Max. Value (Int)	Mean Value (Int)	Std. Dev.	Area (mm2)
1	U1	Unknown	576 060	218 917	159,2	N/A	N/A	2 244	0	1 672	256,7	269,2	60,2
2	U2	Unknown	3 329 184	2 661 073	297,7	N/A	N/A	2 244	0	12 900	1 483,6	2 407,9	60,2
3	U3	Unknown	3 867 540	2 724 581	509,3	N/A	N/A	2 244	0	11 112	1 723,5	2 179,7	60,2
4	U4	Unknown	2 263 760	1 316 191	422,3	N/A	N/A	2 244	0	7 100	1 008,8	1 405,5	60,2
5	U5	Unknown	3 937 260	3 050 718	395,1	N/A	N/A	2 244	0	12 732	1 754,6	2 485,0	60,2
6	U6	Unknown	2 897 656	1 858 476	463,1	N/A	N/A	2 244	0	5 848	1 291,3	1 427,8	60,2
7	U7	Unknown	3 857 212	3 110 214	332,9	N/A	N/A	2 244	0	12 224	1 718,9	2 362,1	60,2

Image Report: Histologia 2023-01-19 14hr 07min_Exposure_16.8sec 2cpERK

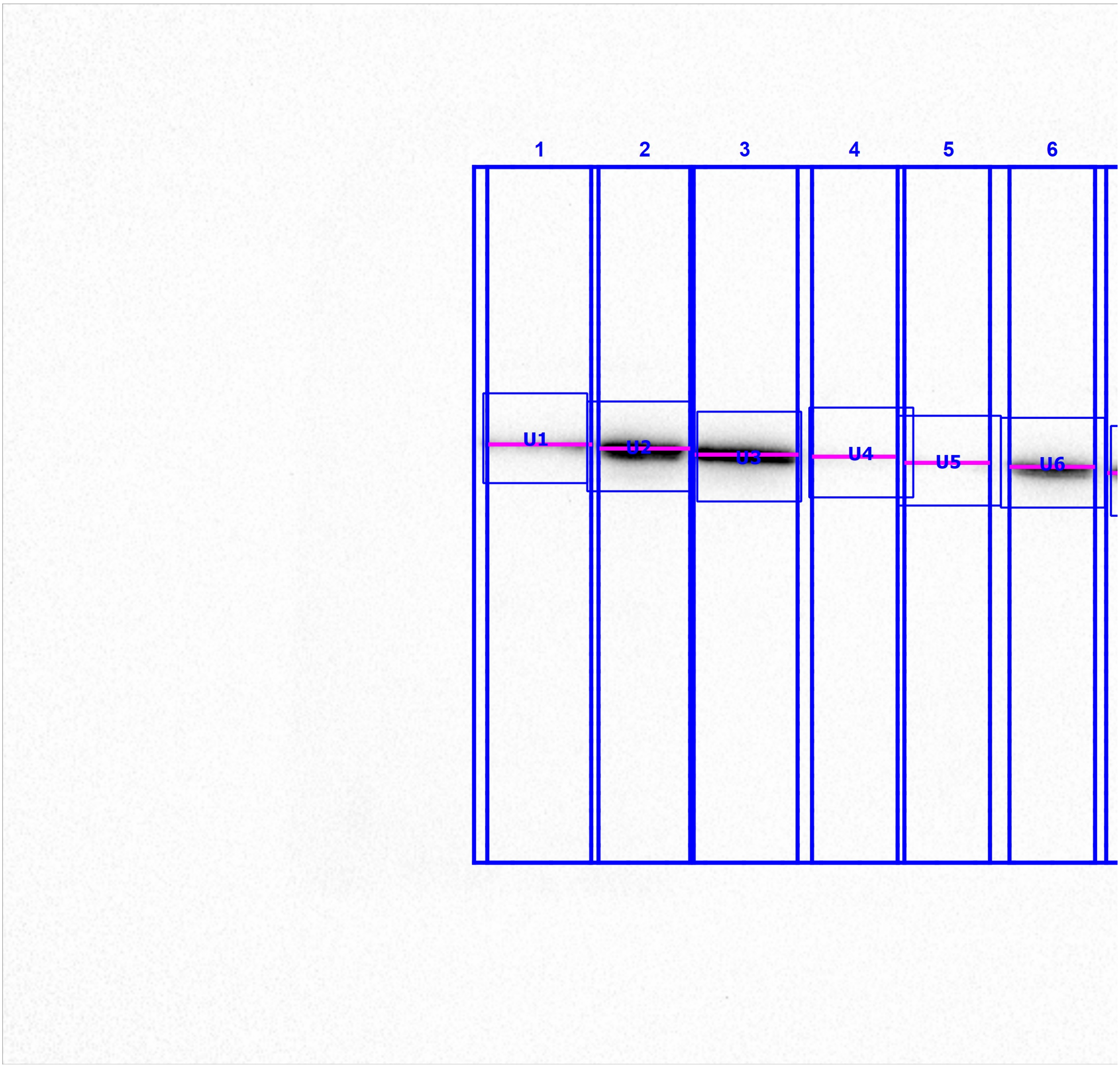

C:\Users\rusak\OneDrive\Dokumenty\Badania\CHI3L2 in BC\BC westerny ilościowo\pErK BC 19.1.23\2\Histologia 2023-01-19 14hr 07min_Exposure_16.8sec 2cpERK.scn

Acquisition Information

Imager	ChemiDoc MP
Exposure Time (sec)	16.800 (Signal Accumulation)
Flat Field	Applied (Lens)

Serial Number	731BR01769
Software Version	5.0
Application	Chemi Hi Resolution
Excitation Source	No Illumination
Emission Filter	No Filter
Binning	2x2

Image Information

Acquisition Date	19/1/2023 2:07:54 PM
User Name	Histologia
Image Area (mm)	X: 114.0 Y: 85.2
Pixel Size (µm)	X: 163.8 Y: 163.8
Data Range (Int)	0 - 25524

Analysis Settings

Detection	Lane detection:<br>Manually created lanes<br><br>Band detection:<br>Automatically detected bands with sensitivity: Low<br>Manually adjusted bands<br><br>Lane Background Subtraction:<br>Lane background subtracted with disk size: 10<br><br>Lane width: Variable
Volume Analysis	Background subtraction method: Local<br>Quantity regression method: Linear

Lane Statistics

Lane No.	Adj. Total Band Vol. (Int)	Total Band Vol. (Int)	Adj. Total Lane Vol. (Int)	Total Lane Vol. (Int)	Bkgd. Vol. (Int)	Norm. Factor
1	1 665 099	1 765 008	2 645 268	4 560 369	1 915 101	N/A
2	7 554 555	7 740 855	8 320 095	10 063 980	1 743 885	N/A
3	8 343 957	8 452 791	9 359 265	10 707 450	1 348 185	N/A
4	338 142	409 752	832 776	1 995 714	1 162 938	N/A
5	287 826	330 162	834 666	2 018 898	1 184 232	N/A
6	3 961 860	4 056 486	4 740 666	5 986 512	1 245 846	N/A
7	4 900 555	5 000 050	6 176 060	7 936 665	1 760 605	N/A

Lane And Band Analysis

Lane 1

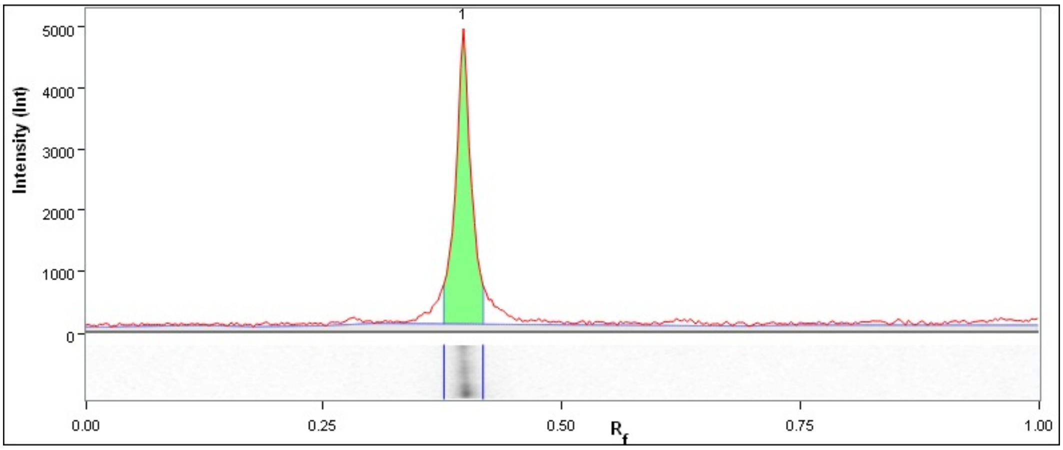

Band No.	Band Label	Mol. Wt. (KDa)	Relative Front	Adj. Volume (Int)	Volume (Int)	Abs. Quant.	Rel. Quant.	Band %	Lane %
1		N/A	0,399	1 665 099	1 765 008	N/A	N/A	100,0	62,9

Band Detection	Automatically detected bands with sensitivity: Low
Lane Background	Lane background subtracted with disk size: 10
Lane Width	8.35 mm

Lane 2

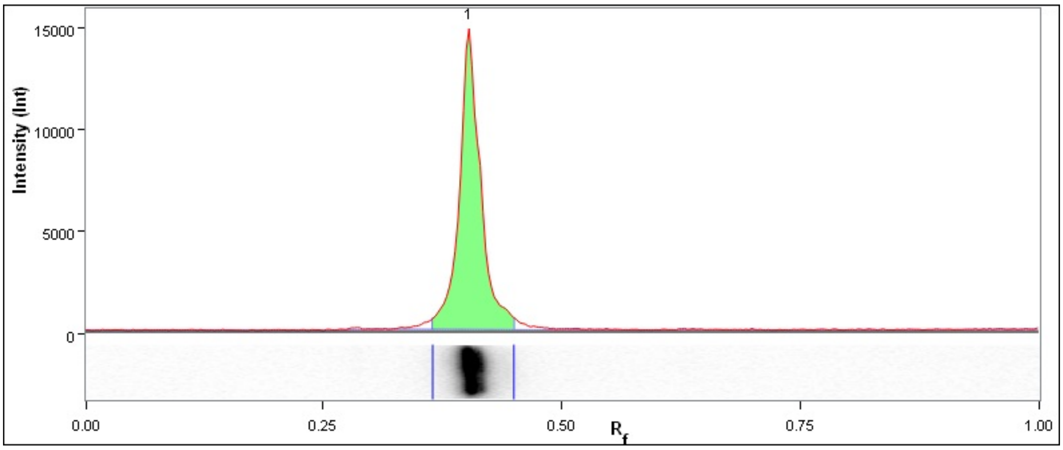

Band No.	Band Label	Mol. Wt. (KDa)	Relative Front	Adj. Volume (Int)	Volume (Int)	Abs. Quant.	Rel. Quant.	Band %	Lane %
1		N/A	0,405	7 554 555	7 740 855	N/A	N/A	100,0	90,8

Band Detection	Automatically detected bands with sensitivity: Low
Lane Background	Lane background subtracted with disk size: 10
Lane Width	7.37 mm

Lane 3

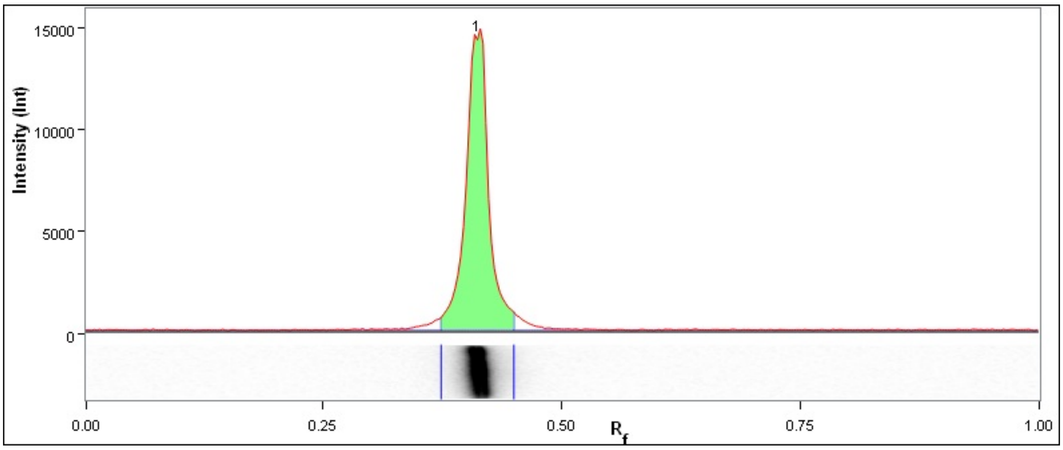

Band No.	Band Label	Mol. Wt. (KDa)	Relative Front	Adj. Volume (Int)	Volume (Int)	Abs. Quant.	Rel. Quant.	Band %	Lane %
1		N/A	0,413	8 343 957	8 452 791	N/A	N/A	100,0	89,2

Band Detection	Automatically detected bands with sensitivity: Low
Lane Background	Lane background subtracted with disk size: 10
Lane Width	8.35 mm

Lane 4

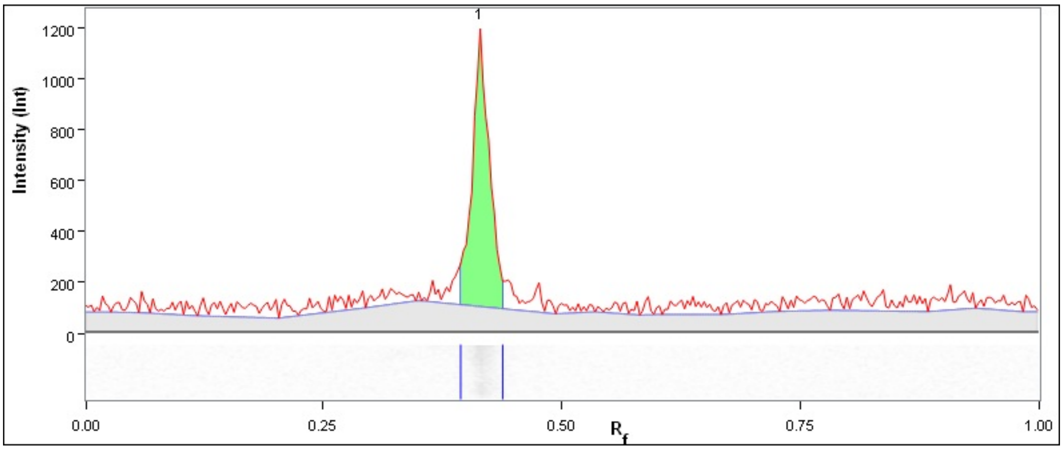

Band No.	Band Label	Mol. Wt. (KDa)	Relative Front	Adj. Volume (Int)	Volume (Int)	Abs. Quant.	Rel. Quant.	Band %	Lane %
1		N/A	0,416	338 142	409 752	N/A	N/A	100,0	40,6

Band Detection	Automatically detected bands with sensitivity: Low
Lane Background	Lane background subtracted with disk size: 10
Lane Width	6.88 mm

Lane 5

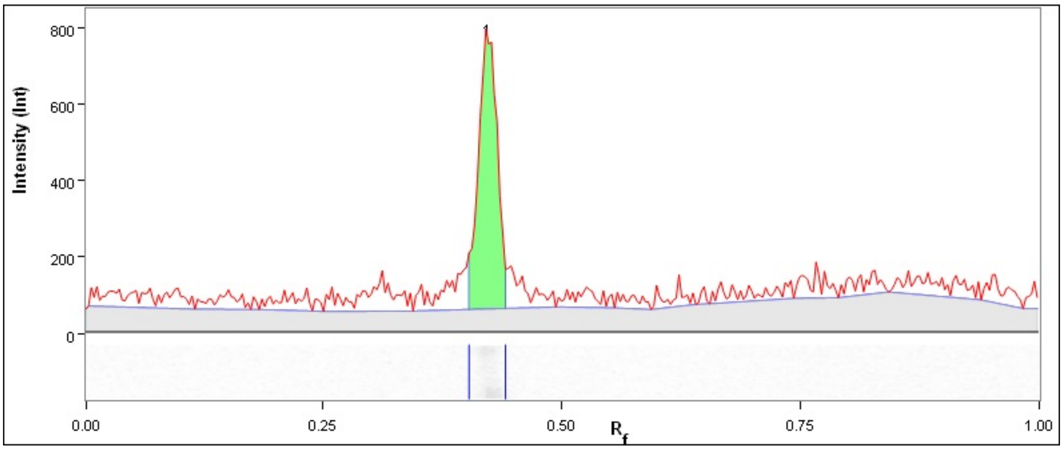

Band No.	Band Label	Mol. Wt. (KDa)	Relative Front	Adj. Volume (Int)	Volume (Int)	Abs. Quant.	Rel. Quant.	Band %	Lane %
1		N/A	0,425	287 826	330 162	N/A	N/A	100,0	34,5

Band Detection	Automatically detected bands with sensitivity: Low
Lane Background	Lane background subtracted with disk size: 10
Lane Width	6.88 mm

Lane 6

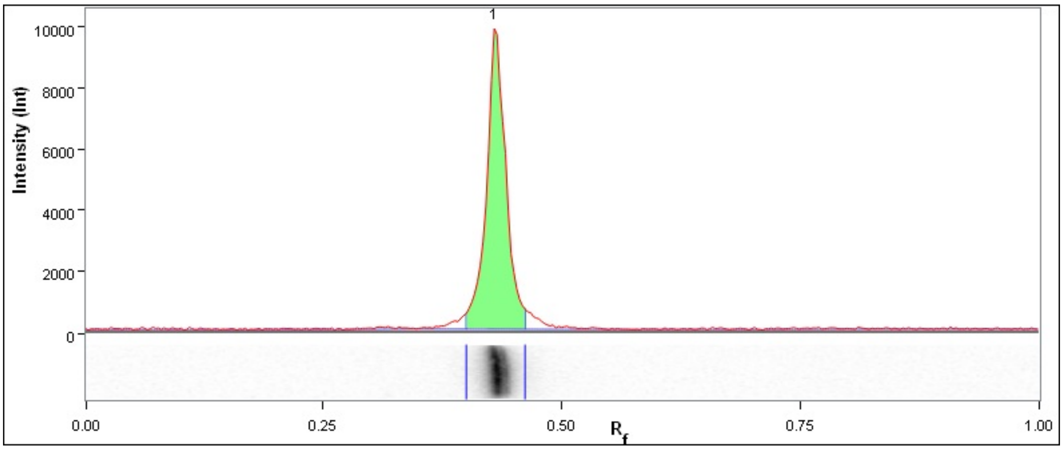

Band No.	Band Label	Mol. Wt. (KDa)	Relative Front	Adj. Volume (Int)	Volume (Int)	Abs. Quant.	Rel. Quant.	Band %	Lane %
1		N/A	0,431	3 961 860	4 056 486	N/A	N/A	100,0	83,6

Band Detection	Automatically detected bands with sensitivity: Low
Lane Background	Lane background subtracted with disk size: 10
Lane Width	6.88 mm

Lane 7

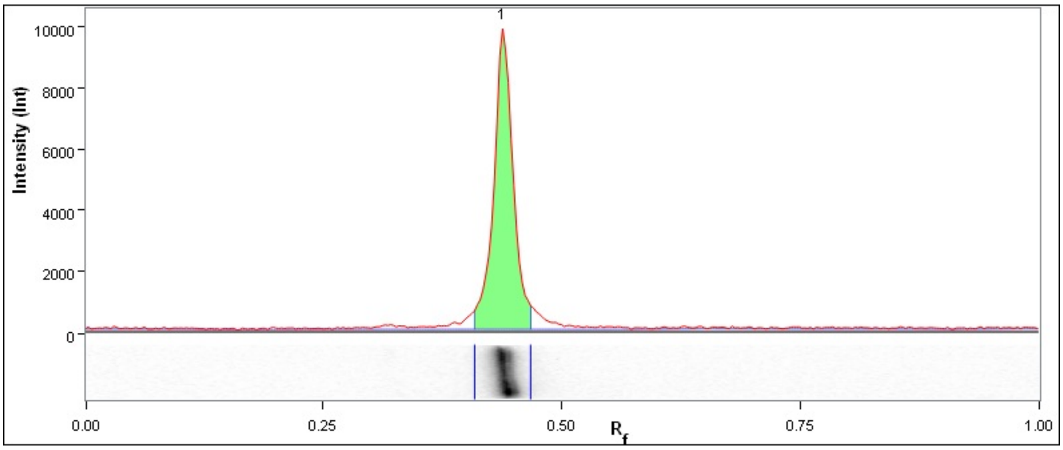

Band No.	Band Label	Mol. Wt. (KDa)	Relative Front	Adj. Volume (Int)	Volume (Int)	Abs. Quant.	Rel. Quant.	Band %	Lane %
1		N/A	0,440	4 900 555	5 000 050	N/A	N/A	100,0	79,3

Band Detection	Automatically detected bands with sensitivity: Low
Lane Background	Lane background subtracted with disk size: 10
Lane Width	9.01 mm

Volume Analysis

No.	Label	Type	Volume (Int)	Adj. Vol. (Int)	Mean Bkgd. (Int)	Abs. Quant.	Rel. Quant.	# of Pixels	Min. Value (Int)	Max. Value (Int)	Mean Value (Int)	Std. Dev.	Area (mm2)
1	U1	Unknown	2 240 832	1 126 605	496,5	N/A	N/A	2 244	0	8 364	998,6	1 334,1	60,2
2	U2	Unknown	8 367 848	6 456 330	851,8	N/A	N/A	2 244	0	25 524	3 729,0	5 334,3	60,2
3	U3	Unknown	8 778 904	6 586 169	977,2	N/A	N/A	2 244	0	22 192	3 912,2	5 481,4	60,2
4	U4	Unknown	702 308	20 178	304,0	N/A	N/A	2 244	0	2 024	313,0	353,8	60,2
5	U5	Unknown	613 372	191 593	188,0	N/A	N/A	2 244	0	1 988	273,3	327,2	60,2
6	U6	Unknown	4 690 704	3 858 851	370,7	N/A	N/A	2 244	0	15 212	2 090,3	3 174,3	60,2
7	U7	Unknown	5 341 440	3 797 105	688,2	N/A	N/A	2 244	0	16 348	2 380,3	3 402,9	60,2

Image Report: Histologia 2023-01-19 14hr 05min_Exposure_16.8sec 1b pERK

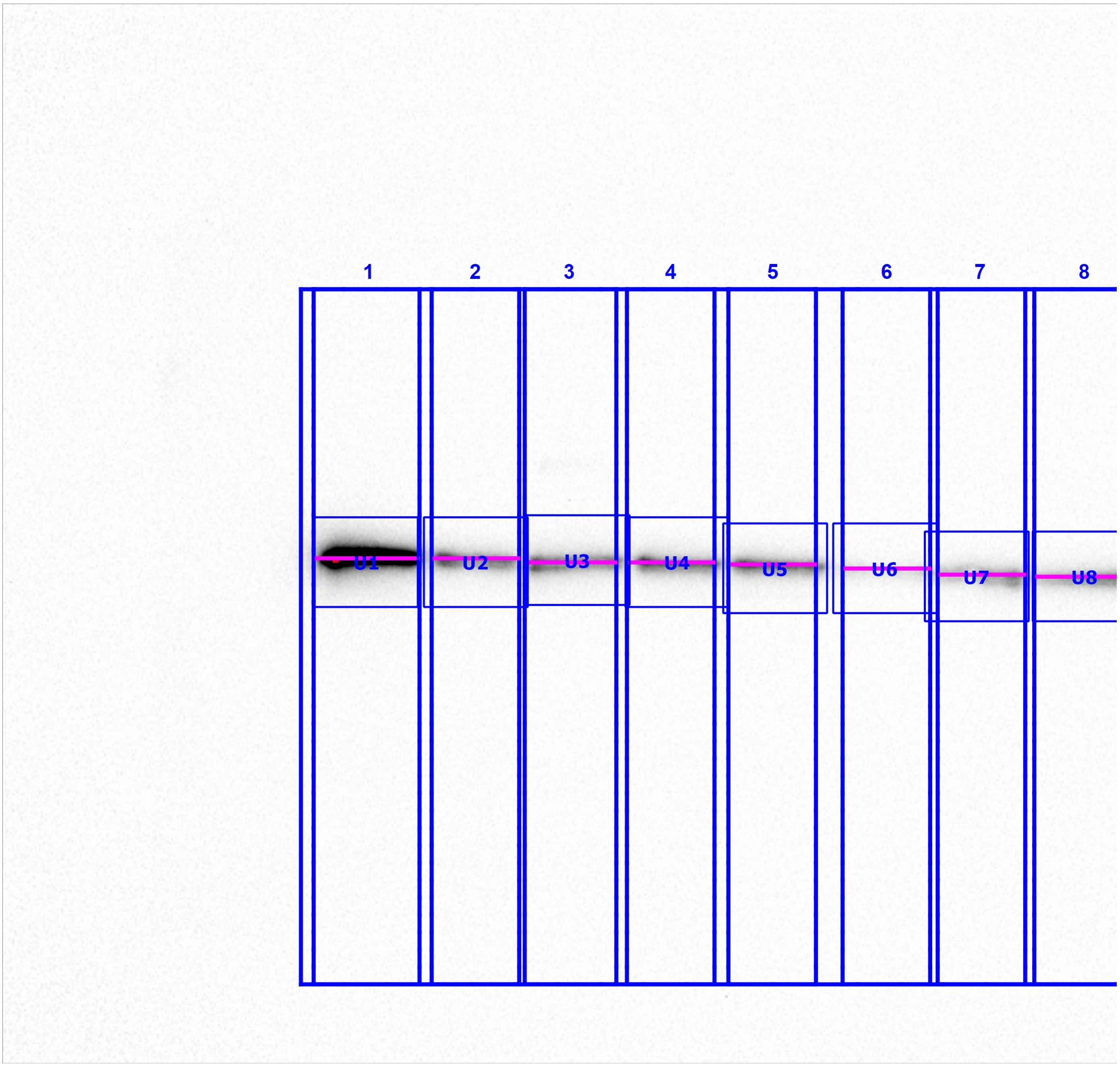

C:\Users\rusak\OneDrive\Dokumenty\Badania\CHI3L2 in BC\BC westerny ilościowo\pErK BC 19.1.23\1\Histologia 2023-01-19 14hr 05min_Exposure_16.8sec 1b pERK.scn

Acquisition Information

Imager	ChemiDoc MP
Exposure Time (sec)	16.800 (Signal Accumulation)
Flat Field	Applied (Lens)

Serial Number	731BR01769
Software Version	5.0
Application	Chemi Hi Resolution
Excitation Source	No Illumination
Emission Filter	No Filter
Binning	2x2

Image Information

Acquisition Date	19/1/2023 2:05:42 PM
User Name	Histologia
Image Area (mm)	X: 114.0 Y: 85.2
Pixel Size (μm)	X: 163.8 Y: 163.8
Data Range (Int)	0 - 65535

Analysis Settings

Detection	Lane detection:<br>Manually created lanes<br><br>Band detection:<br>Automatically detected bands with sensitivity: Low<br>Manually adjusted bands<br><br>Lane Background Subtraction:<br>Lane background subtracted with disk size: 10<br><br>Lane width: Variable
Volume Analysis	Background subtraction method: Local<br>Quantity regression method: Linear

Lane Statistics

Lane No.	Adj. Total Band Vol. (Int)	Total Band Vol. (Int)	Adj. Total Lane Vol. (Int)	Total Lane Vol. (Int)	Bkgd. Vol. (Int)	Norm. Factor
1	15 633 020	15 782 156	17 209 400	18 922 332	1 712 932	N/A
2	2 963 947	3 041 347	3 779 270	5 078 773	1 299 503	N/A
3	3 030 525	3 099 375	3 995 460	5 244 885	1 249 425	N/A
4	2 890 116	2 944 339	3 700 924	4 767 797	1 066 873	N/A
5	2 976 761	3 034 897	3 739 667	4 819 139	1 079 472	N/A
6	707 350	758 735	1 281 357	2 232 345	950 988	N/A
7	1 951 426	2 012 658	2 631 471	3 681 574	1 050 103	N/A
8	2 431 772	2 499 833	3 176 376	4 502 218	1 325 842	N/A

Lane And Band Analysis

Lane 1

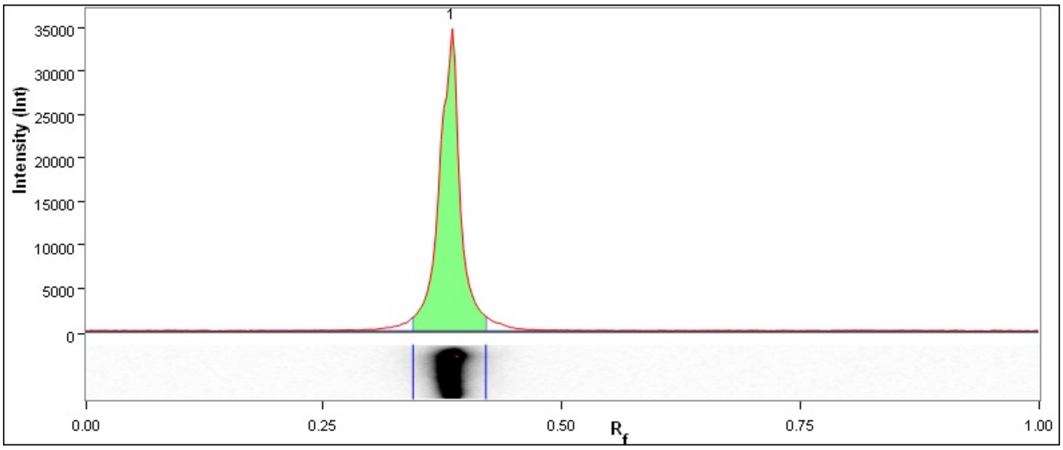

Band No.	Band Label	Mol. Wt. (KDa)	Relative Front	Adj. Volume (Int)	Volume (Int)	Abs. Quant.	Rel. Quant.	Band %	Lane %
1		N/A	0,387	15 633 020	15 782 156	N/A	N/A	100,0	90,8

Band Detection	Automatically detected bands with sensitivity: Low
Lane Background	Lane background subtracted with disk size: 10
Lane Width	8.52 mm

Lane 2

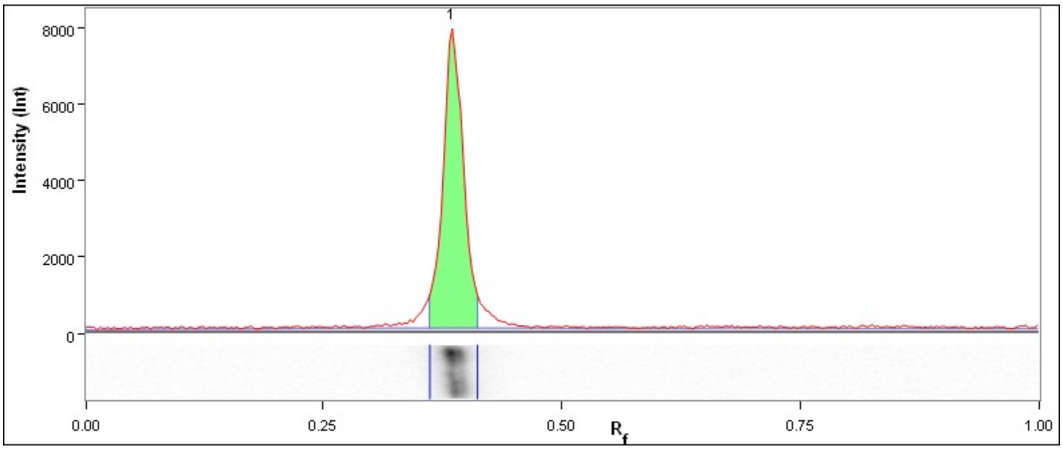

Band No.	Band Label	Mol. Wt. (KDa)	Relative Front	Adj. Volume (Int)	Volume (Int)	Abs. Quant.	Rel. Quant.	Band %	Lane %
1		N/A	0,387	2 963 947	3 041 347	N/A	N/A	100,0	78,4

Band Detection	Automatically detected bands with sensitivity: Low
Lane Background	Lane background subtracted with disk size: 10
Lane Width	7.04 mm

Lane 3

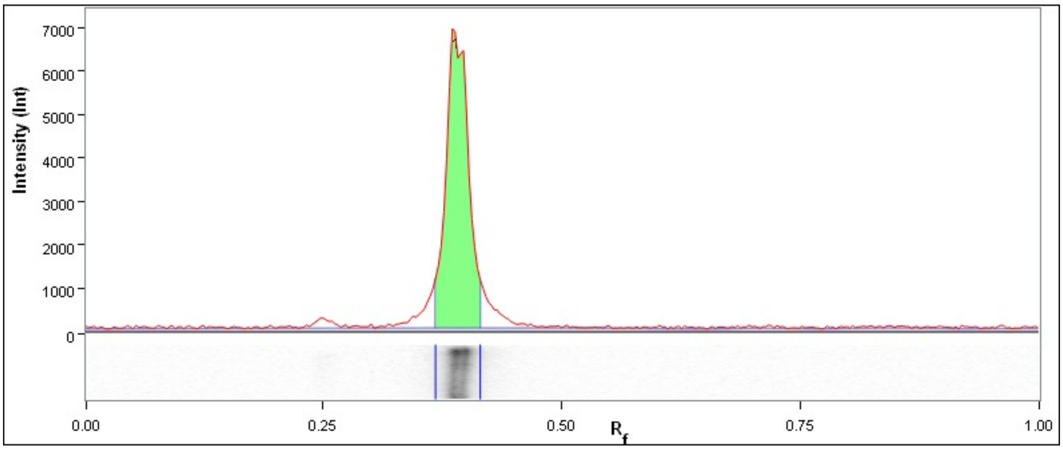

Band No.	Band Label	Mol. Wt. (KDa)	Relative Front	Adj. Volume (Int)	Volume (Int)	Abs. Quant.	Rel. Quant.	Band %	Lane %
1		N/A	0,393	3 030 525	3 099 375	N/A	N/A	100,0	75,8

Band Detection	Automatically detected bands with sensitivity: Low
Lane Background	Lane background subtracted with disk size: 10
Lane Width	7.37 mm

Lane 4

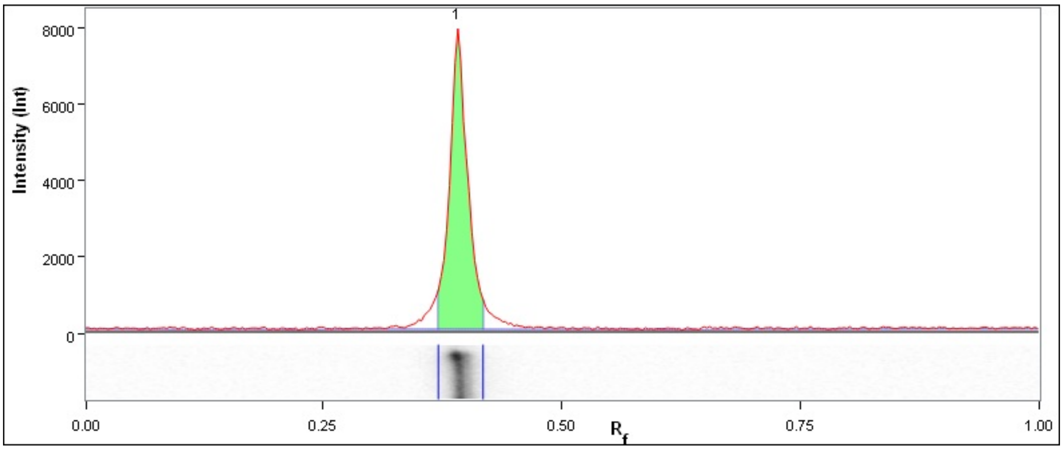

Band No.	Band Label	Mol. Wt. (KDa)	Relative Front	Adj. Volume (Int)	Volume (Int)	Abs. Quant.	Rel. Quant.	Band %	Lane %
1		N/A	0,393	2 890 116	2 944 339	N/A	N/A	100,0	78,1

Band Detection	Automatically detected bands with sensitivity: Low
Lane Background	Lane background subtracted with disk size: 10
Lane Width	7.04 mm

Lane 5

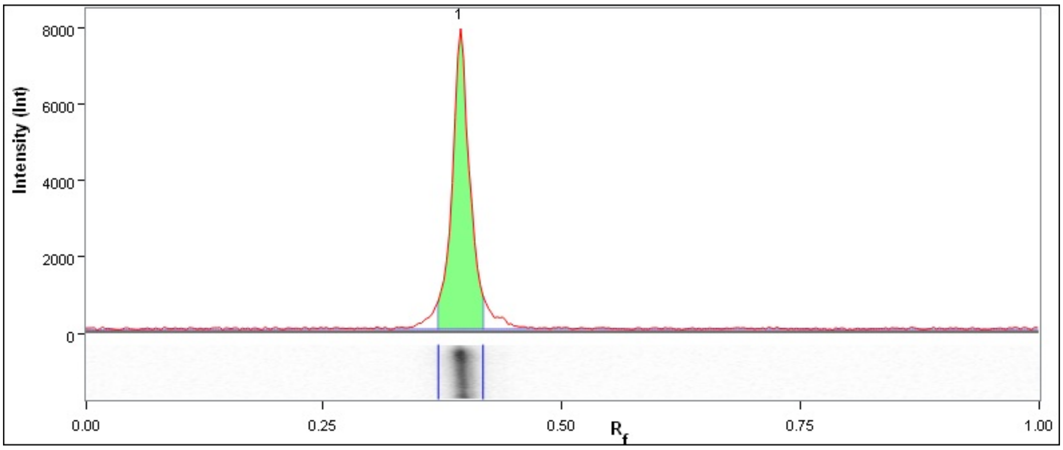

Band No.	Band Label	Mol. Wt. (KDa)	Relative Front	Adj. Volume (Int)	Volume (Int)	Abs. Quant.	Rel. Quant.	Band %	Lane %
1		N/A	0,396	2 976 761	3 034 897	N/A	N/A	100,0	79,6

Band Detection	Automatically detected bands with sensitivity: Low
Lane Background	Lane background subtracted with disk size: 10
Lane Width	7.04 mm

Lane 6

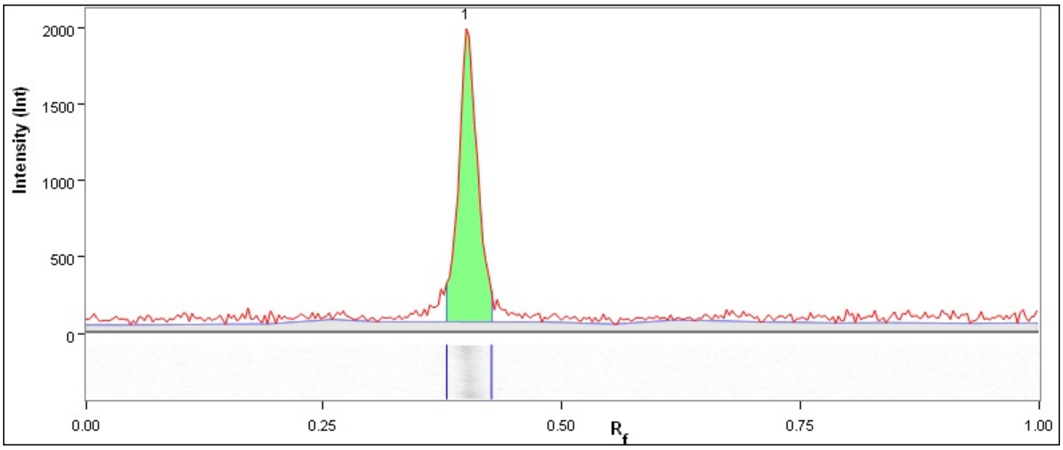

Band No.	Band Label	Mol. Wt. (KDa)	Relative Front	Adj. Volume (Int)	Volume (Int)	Abs. Quant.	Rel. Quant.	Band %	Lane %
1		N/A	0,402	707 350	758 735	N/A	N/A	100,0	55,2

Band Detection	Automatically detected bands with sensitivity: Low
Lane Background	Lane background subtracted with disk size: 10
Lane Width	7.04 mm

Lane 7

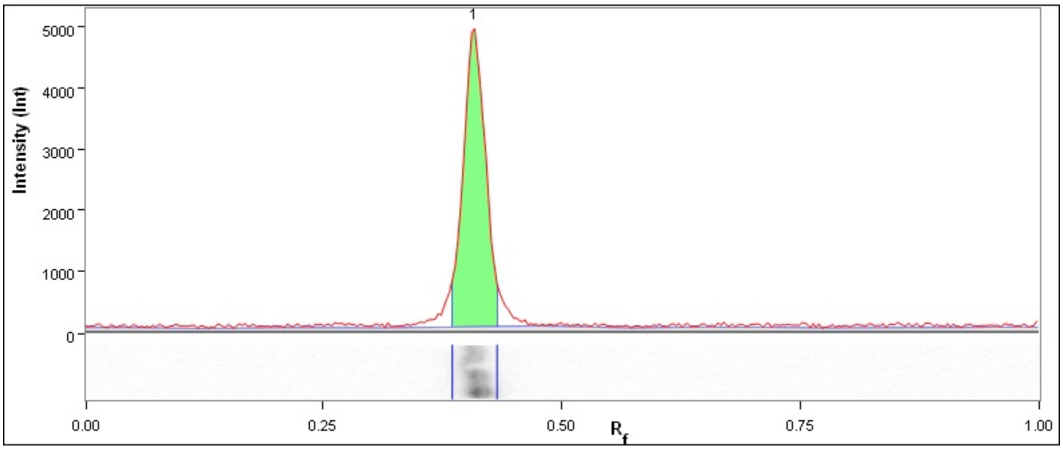

Band No.	Band Label	Mol. Wt. (KDa)	Relative Front	Adj. Volume (Int)	Volume (Int)	Abs. Quant.	Rel. Quant.	Band %	Lane %
1		N/A	0,411	1 951 426	2 012 658	N/A	N/A	100,0	74,2

Band Detection	Automatically detected bands with sensitivity: Low
Lane Background	Lane background subtracted with disk size: 10
Lane Width	7.04 mm

Lane 8

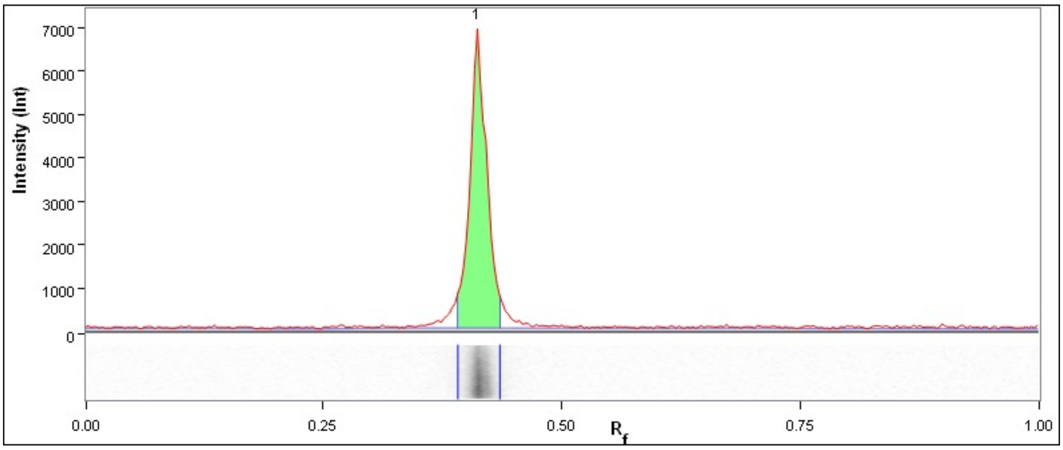

Band No.	Band Label	Mol. Wt. (KDa)	Relative Front	Adj. Volume (Int)	Volume (Int)	Abs. Quant.	Rel. Quant.	Band %	Lane %
1		N/A	0,413	2 431 772	2 499 833	N/A	N/A	100,0	76,6

Band Detection	Automatically detected bands with sensitivity: Low
Lane Background	Lane background subtracted with disk size: 10
Lane Width	8.03 mm

Volume Analysis

No.	Label	Type	Volume (Int)	Adj. Vol. (Int)	Mean Bkgd. (Int)	Abs. Quant.	Rel. Quant.	# of Pixels	Min. Value (Int)	Max. Value (Int)	Mean Value (Int)	Std. Dev.	Area (mm2)
1	U1	Unknown	16 442 829	13 284 295	1 407,5	N/A	N/A	2 244	0	65 535	7 327,5	10 685,2	60,2
2	U2	Unknown	3 870 804	1 803 502	921,3	N/A	N/A	2 244	0	13 848	1 725,0	2 373,1	60,2
3	U3	Unknown	3 971 928	2 876 578	488,1	N/A	N/A	2 244	0	11 808	1 770,0	2 295,0	60,2

4	U4	Unknown	3 800 912	2 604 235	533,3	N/A	N/A	2 244	0	13 504	1 693,8	2 475,9	60,2
5	U5	Unknown	3 862 424	2 922 304	418,9	N/A	N/A	2 244	0	12 472	1 721,2	2 509,4	60,2
6	U6	Unknown	1 132 576	391 547	330,2	N/A	N/A	2 244	0	3 716	504,7	645,9	60,2
7	U7	Unknown	2 583 836	1 736 853	377,4	N/A	N/A	2 244	0	8 944	1 151,4	1 602,0	60,2
8	U8	Unknown	2 963 936	2 135 553	369,2	N/A	N/A	2 244	0	9 616	1 320,8	1 920,4	60,2

Image Report: Histologia 2023-01-17 16hr 18min_Exposure_16.8sec 2b pERK

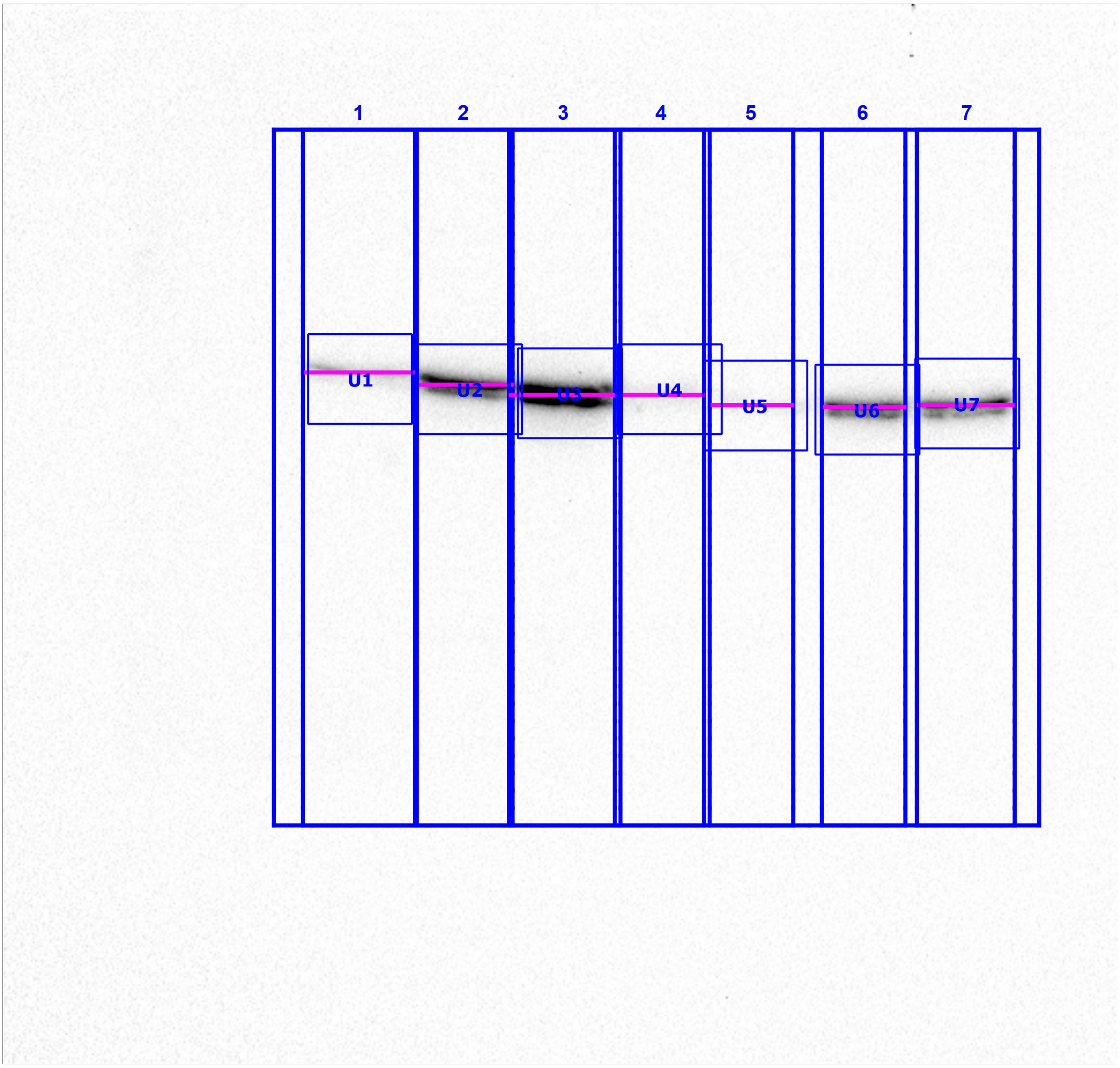

C:\Users\rusak\OneDrive\Dokumenty\Badania\CHI3L2 in BC\BC westerny ilościowo\pERK BC 17.1.23\2\Histologia 2023-01-17 16hr 18min_Exposure_16.8sec 2b pERK.scn

Acquisition Information

Imager	ChemiDoc MP
Exposure Time (sec)	16.800 (Signal Accumulation)
Flat Field	Applied (Lens)

Serial Number	731BR01769
Software Version	5.0
Application	Chemi Hi Resolution
Excitation Source	No Illumination
Emission Filter	No Filter
Binning	2x2

Image Information

Acquisition Date	17/1/2023 4:19:35 PM
User Name	Histologia
Image Area (mm)	X: 114.0 Y: 85.2
Pixel Size (μm)	X: 163.8 Y: 163.8
Data Range (Int)	0 - 21304

Analysis Settings

Detection	Lane detection:<br>Manually created lanes<br><br>Band detection:<br>Automatically detected bands with sensitivity: Low<br>Manually adjusted bands<br><br>Lane Background Subtraction:<br>Lane background subtracted with disk size: 10<br><br>Lane width: Variable
Volume Analysis	Background subtraction method: Local<br>Quantity regression method: Linear

Lane Statistics

Lane No.	Adj. Total Band Vol. (Int)	Total Band Vol. (Int)	Adj. Total Lane Vol. (Int)	Total Lane Vol. (Int)	Bkgd. Vol. (Int)	Norm. Factor
1	1 028 500	1 126 400	1 897 830	3 803 305	1 905 475	N/A
2	4 650 509	4 776 751	5 605 690	7 157 489	1 551 799	N/A
3	8 295 560	8 489 052	9 393 384	11 216 348	1 822 964	N/A
4	573 590	687 652	1 140 538	2 303 913	1 163 375	N/A
5	253 749	323 080	816 474	1 952 297	1 135 823	N/A
6	3 078 321	3 168 767	3 711 115	4 872 563	1 161 448	N/A
7	3 151 008	3 244 272	3 830 112	5 133 552	1 303 440	N/A

Lane And Band Analysis

Lane 1

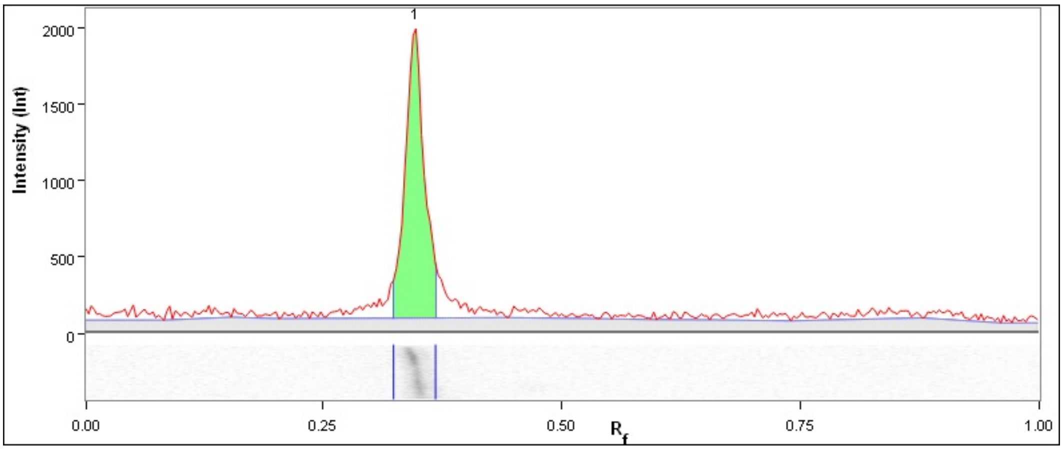

Band No.	Band Label	Mol. Wt. (KDa)	Relative Front	Adj. Volume (Int)	Volume (Int)	Abs. Quant.	Rel. Quant.	Band %	Lane %
1		N/A	0,349	1 028 500	1 126 400	N/A	N/A	100,0	54,2

Band Detection	Automatically detected bands with sensitivity: Low
Lane Background	Lane background subtracted with disk size: 10
Lane Width	9.01 mm

Lane 2

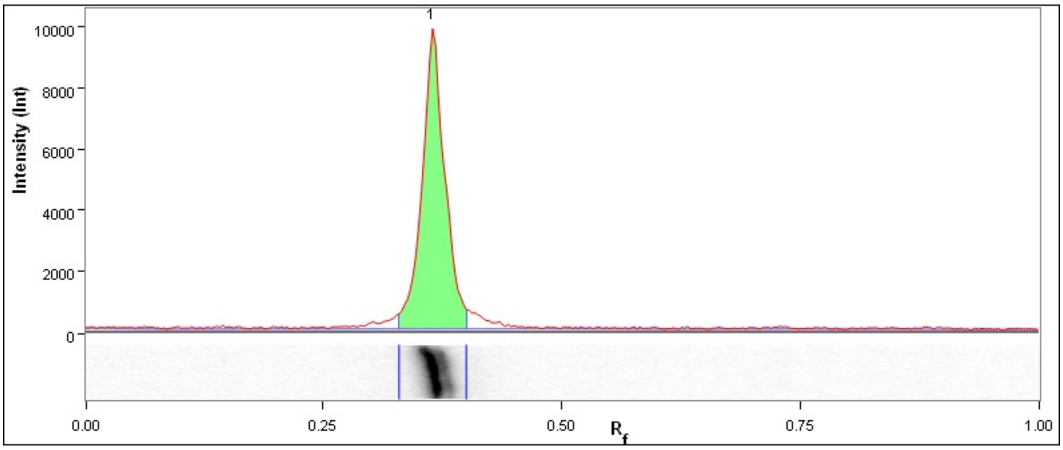

Band No.	Band Label	Mol. Wt. (KDa)	Relative Front	Adj. Volume (Int)	Volume (Int)	Abs. Quant.	Rel. Quant.	Band %	Lane %
1		N/A	0,367	4 650 509	4 776 751	N/A	N/A	100,0	83,0

Band Detection	Automatically detected bands with sensitivity: Low
Lane Background	Lane background subtracted with disk size: 10
Lane Width	7.70 mm

Lane 3

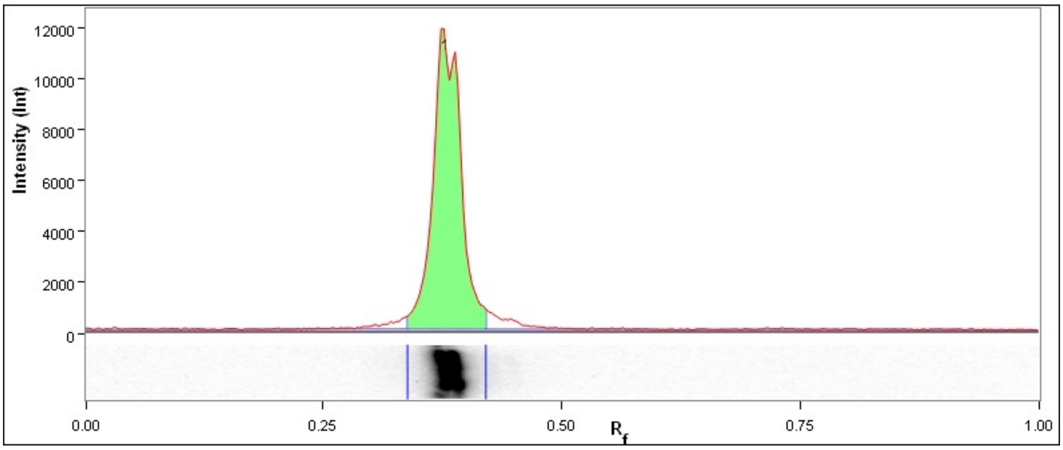

Band No.	Band Label	Mol. Wt. (KDa)	Relative Front	Adj. Volume (Int)	Volume (Int)	Abs. Quant.	Rel. Quant.	Band %	Lane %
1		N/A	0,381	8 295 560	8 489 052	N/A	N/A	100,0	88,3

Band Detection	Automatically detected bands with sensitivity: Low
Lane Background	Lane background subtracted with disk size: 10
Lane Width	8.52 mm

Lane 4

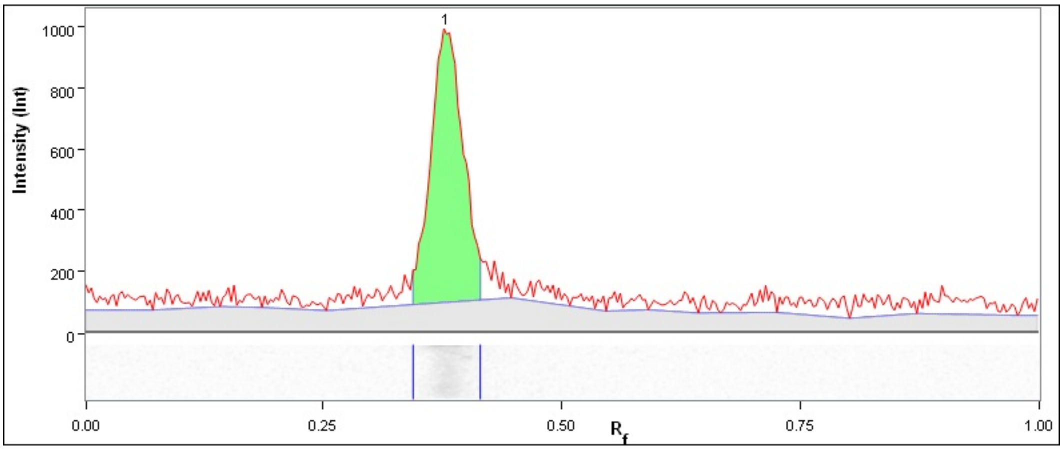

Band No.	Band Label	Mol. Wt. (KDa)	Relative Front	Adj. Volume (Int)	Volume (Int)	Abs. Quant.	Rel. Quant.	Band %	Lane %
1		N/A	0,381	573 590	687 652	N/A	N/A	100,0	50,3

Band Detection	Automatically detected bands with sensitivity: Low
Lane Background	Lane background subtracted with disk size: 10
Lane Width	6.72 mm

Lane 5

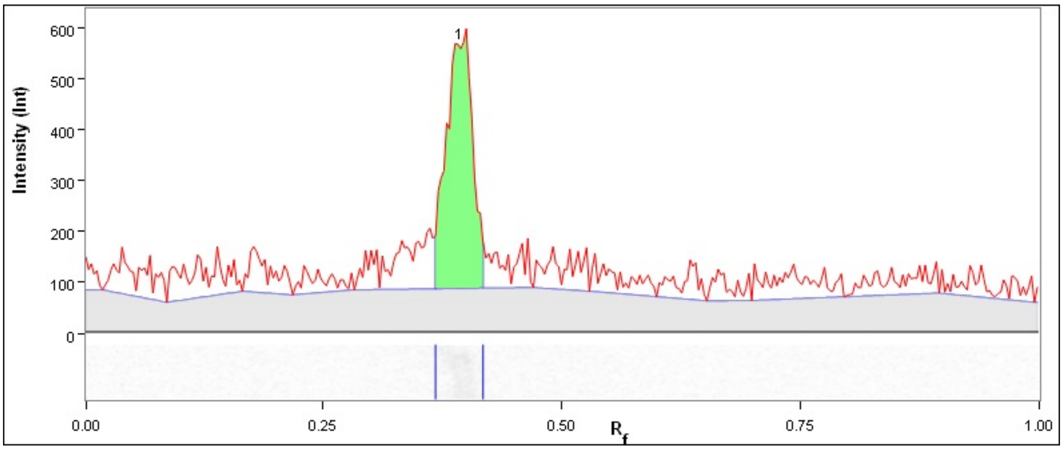

Band No.	Band Label	Mol. Wt. (KDa)	Relative Front	Adj. Volume (Int)	Volume (Int)	Abs. Quant.	Rel. Quant.	Band %	Lane %
1		N/A	0,396	253 749	323 080	N/A	N/A	100,0	31,1

Band Detection	Automatically detected bands with sensitivity: Low
Lane Background	Lane background subtracted with disk size: 10
Lane Width	6.72 mm

Lane 6

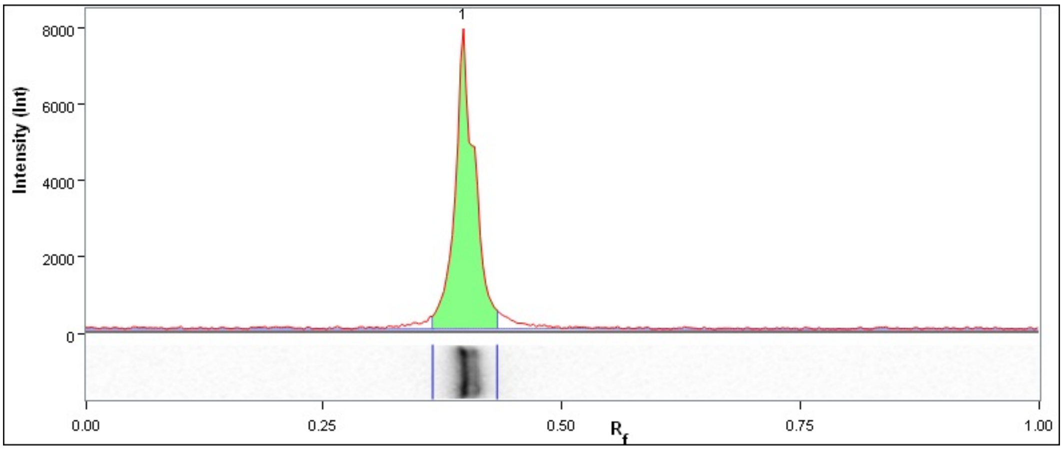

Band No.	Band Label	Mol. Wt. (KDa)	Relative Front	Adj. Volume (Int)	Volume (Int)	Abs. Quant.	Rel. Quant.	Band %	Lane %
1		N/A	0,399	3 078 321	3 168 767	N/A	N/A	100,0	82,9

Band Detection	Automatically detected bands with sensitivity: Low
Lane Background	Lane background subtracted with disk size: 10
Lane Width	6.72 mm

Lane 7

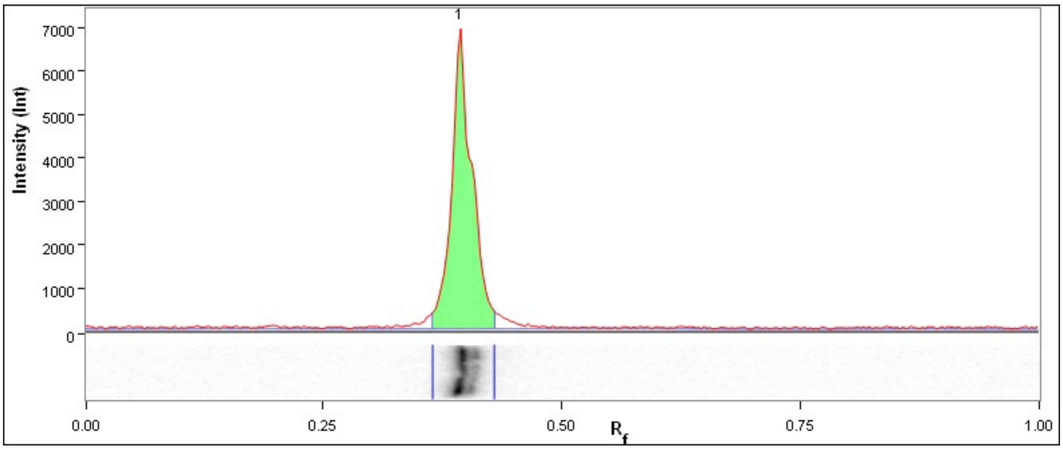

Band No.	Band Label	Mol. Wt. (KDa)	Relative Front	Adj. Volume (Int)	Volume (Int)	Abs. Quant.	Rel. Quant.	Band %	Lane %
1		N/A	0,396	3 151 008	3 244 272	N/A	N/A	100,0	82,3

Band Detection	Automatically detected bands with sensitivity: Low
Lane Background	Lane background subtracted with disk size: 10
Lane Width	7.86 mm

Volume Analysis

No.	Label	Type	Volume (Int)	Adj. Vol. (Int)	Mean Bkgd. (Int)	Abs. Quant.	Rel. Quant.	# of Pixels	Min. Value (Int)	Max. Value (Int)	Mean Value (Int)	Std. Dev.	Area (mm2)
1	U1	Unknown	1 418 084	552 039	385,9	N/A	N/A	2 244	0	4 580	631,9	777,1	60,2
2	U2	Unknown	5 637 296	2 694 186	1 311,5	N/A	N/A	2 244	0	15 520	2 512,2	3 234,3	60,2
3	U3	Unknown	8 770 772	6 792 582	881,5	N/A	N/A	2 244	0	21 304	3 908,5	4 980,2	60,2
4	U4	Unknown	981 592	-172 425	514,3	N/A	N/A	2 244	0	3 448	437,4	441,9	60,2
5	U5	Unknown	648 412	208 634	196,0	N/A	N/A	2 244	0	1 524	289,0	278,7	60,2
6	U6	Unknown	3 755 880	2 815 898	418,9	N/A	N/A	2 244	0	11 464	1 673,7	2 328,2	60,2
7	U7	Unknown	3 580 460	2 889 169	308,1	N/A	N/A	2 244	0	11 628	1 595,6	2 220,9	60,2

Image Report: Histologia 2023-01-17 16hr 21min_Exposure_16.8sec 3b pERK

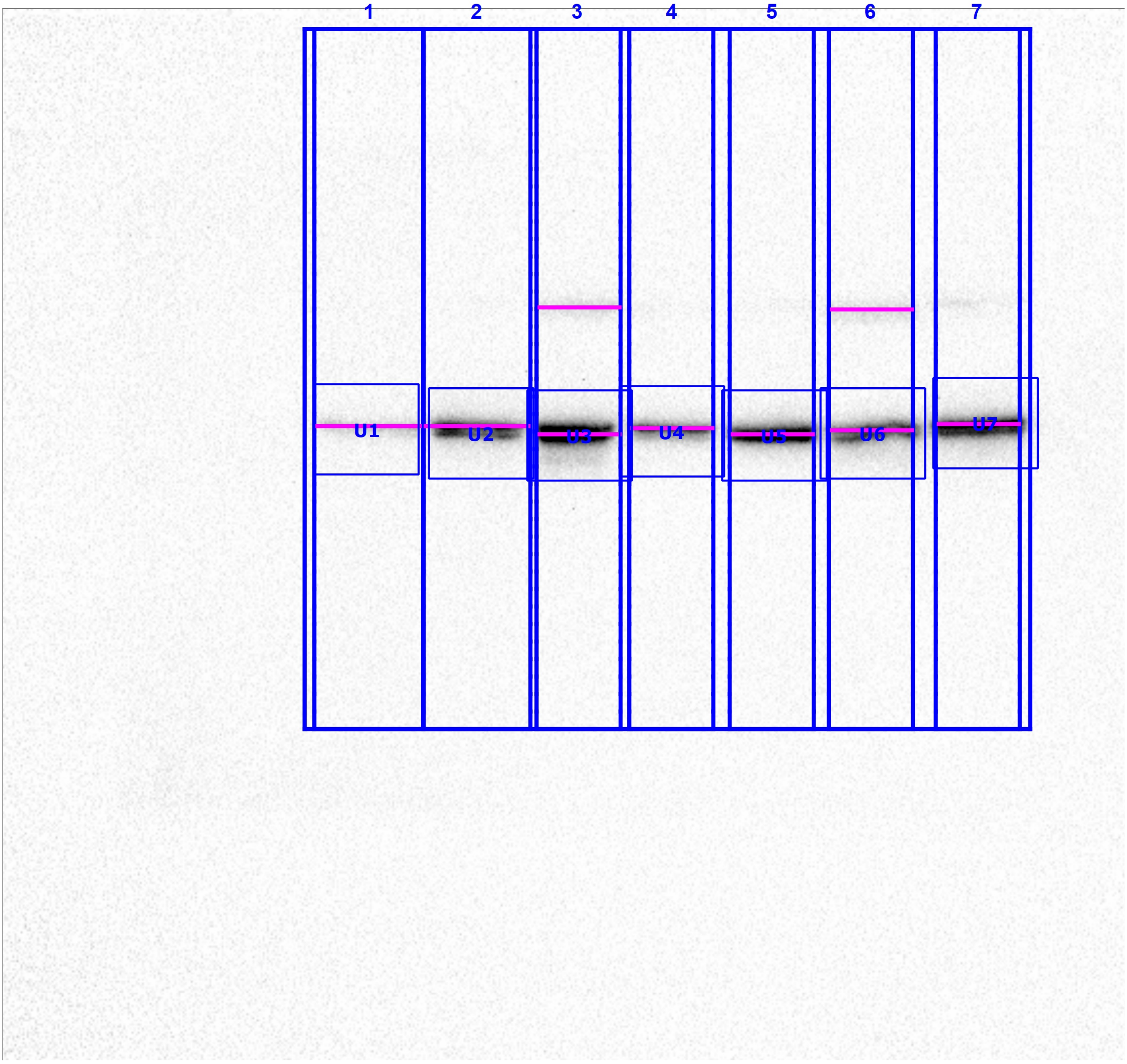

C:\Users\rusak\OneDrive\Dokumenty\Badania\CHI3L2 in BC\BC westerny ilościowo\pERK BC 17.1.23\3\Histologia 2023-01-17 16hr 21min_Exposure_16.8sec 3b pERK.scn

Acquisition Information

Imager	ChemiDoc MP
Exposure Time (sec)	16.800 (Signal Accumulation)
Flat Field	Applied (Lens)

Serial Number	731BR01769
Software Version	5.0
Application	Chemi Hi Resolution
Excitation Source	No Illumination
Emission Filter	No Filter
Binning	2x2

Image Information

Acquisition Date	17/1/2023 4:21:47 PM
User Name	Histologia
Image Area (mm)	X: 114.0 Y: 85.2
Pixel Size (μm)	X: 163.8 Y: 163.8
Data Range (Int)	0 - 11700

Analysis Settings

Detection	Lane detection:<br>Manually created lanes<br><br>Band detection:<br>Automatically detected bands with sensitivity: Low<br><br>Lane Background Subtraction:<br>Lane background subtracted with disk size: 10<br><br>Lane width: Variable
Volume Analysis	Background subtraction method: Local<br>Quantity regression method: Linear

Lane Statistics

Lane No.	Adj. Total Band Vol. (Int)	Total Band Vol. (Int)	Adj. Total Lane Vol. (Int)	Total Lane Vol. (Int)	Bkgd. Vol. (Int)	Norm. Factor
1	557 189	670 874	1 311 803	3 508 176	2 196 373	N/A
2	2 614 352	2 751 268	3 567 096	5 381 480	1 814 384	N/A
3	4 091 964	4 300 080	4 761 084	6 154 428	1 393 344	N/A
4	1 566 241	1 647 544	2 233 721	3 468 887	1 235 166	N/A
5	2 922 726	3 031 868	3 603 162	4 824 962	1 221 800	N/A
6	2 645 935	2 817 110	3 263 764	4 475 437	1 211 673	N/A
7	3 154 786	3 270 652	4 018 205	5 250 009	1 231 804	N/A

Lane And Band Analysis

Lane 1

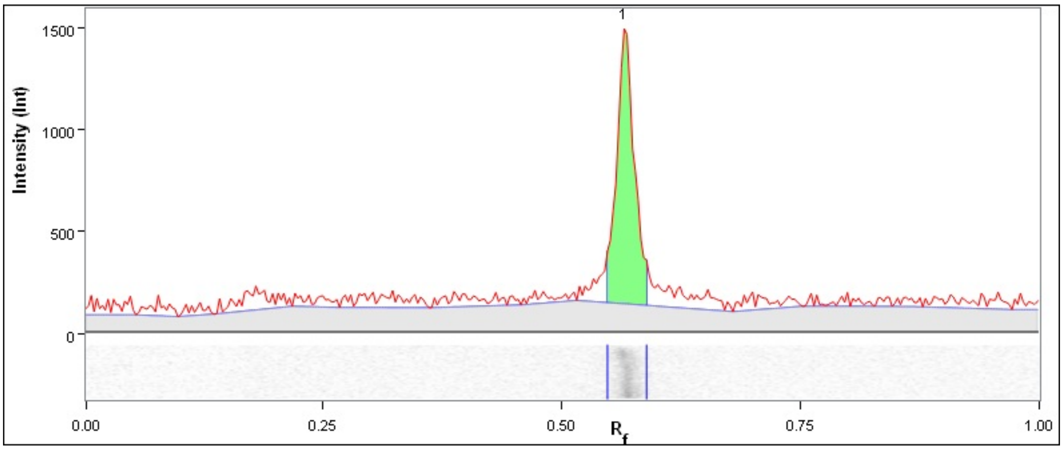

Band No.	Band Label	Mol. Wt. (KDa)	Relative Front	Adj. Volume (Int)	Volume (Int)	Abs. Quant.	Rel. Quant.	Band %	Lane %
1		N/A	0,567	557 189	670 874	N/A	N/A	100,0	42,5

Band Detection	Automatically detected bands with sensitivity: Low
Lane Background	Lane background subtracted with disk size: 10
Lane Width	8.68 mm

Lane 2

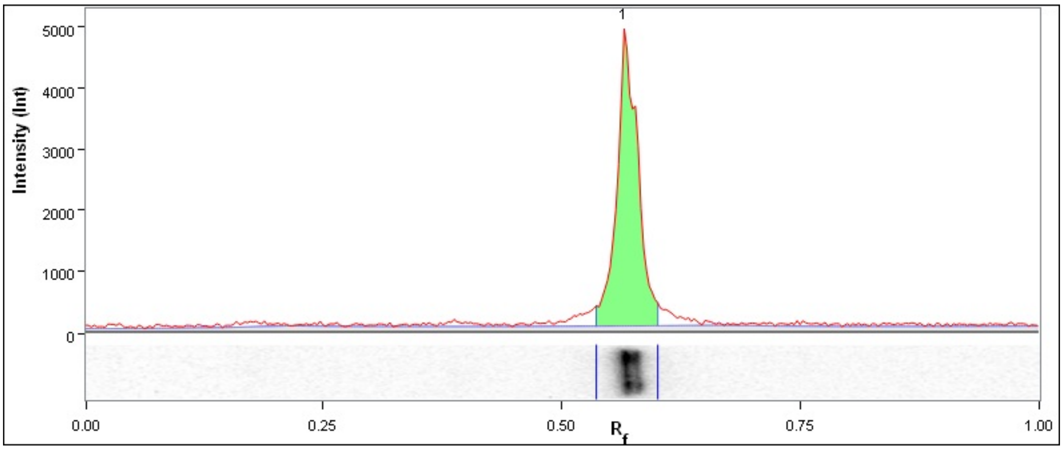

Band No.	Band Label	Mol. Wt. (KDa)	Relative Front	Adj. Volume (Int)	Volume (Int)	Abs. Quant.	Rel. Quant.	Band %	Lane %
1		N/A	0,567	2 614 352	2 751 268	N/A	N/A	100,0	73,3

Band Detection	Automatically detected bands with sensitivity: Low
Lane Background	Lane background subtracted with disk size: 10
Lane Width	8.52 mm

Lane 3

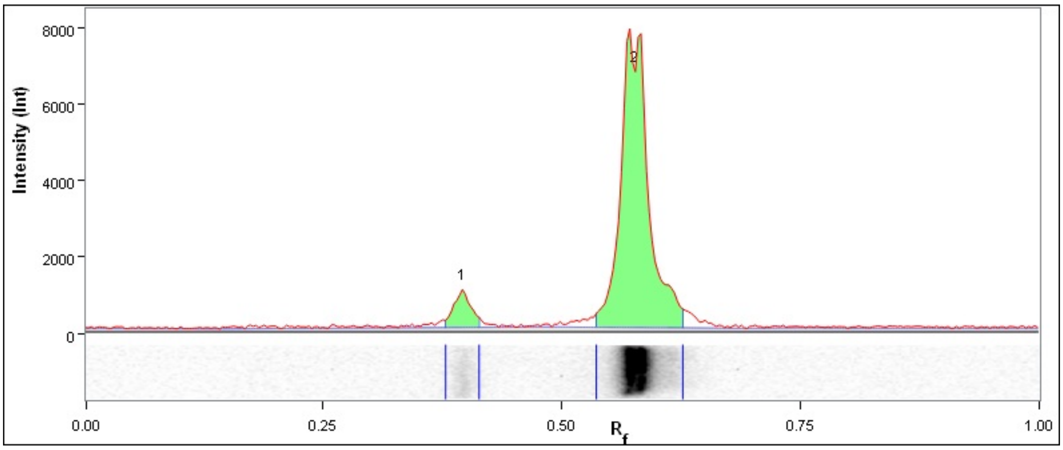

Band No.	Band Label	Mol. Wt. (KDa)	Relative Front	Adj. Volume (Int)	Volume (Int)	Abs. Quant.	Rel. Quant.	Band %	Lane %
1		N/A	0,398	297 701	358 873	N/A	N/A	7,3	6,3
2		N/A	0,579	3 794 263	3 941 207	N/A	N/A	92,7	79,7

Band Detection	Automatically detected bands with sensitivity: Low
Lane Background	Lane background subtracted with disk size: 10
Lane Width	6.72 mm

Lane 4

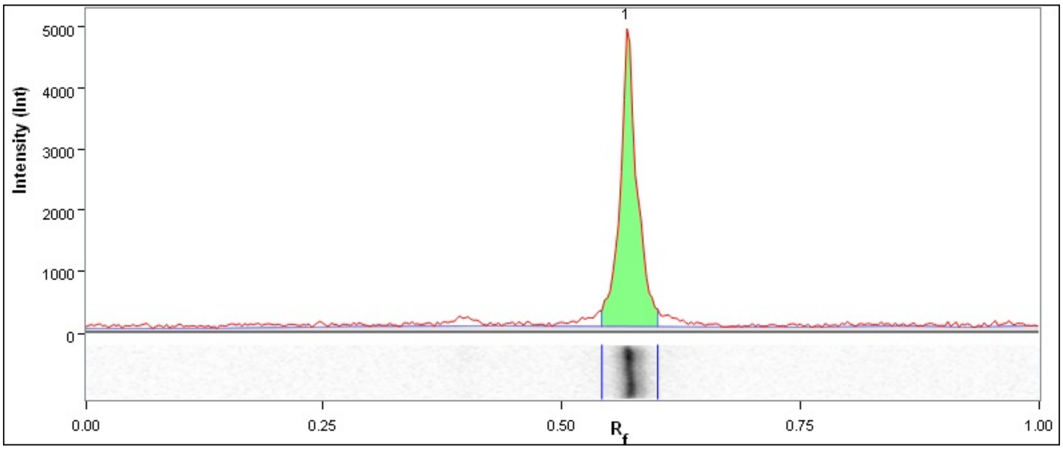

Band No.	Band Label	Mol. Wt. (KDa)	Relative Front	Adj. Volume (Int)	Volume (Int)	Abs. Quant.	Rel. Quant.	Band %	Lane %
1		N/A	0,570	1 566 241	1 647 544	N/A	N/A	100,0	70,1

Band Detection	Automatically detected bands with sensitivity: Low
Lane Background	Lane background subtracted with disk size: 10
Lane Width	6.72 mm

Lane 5

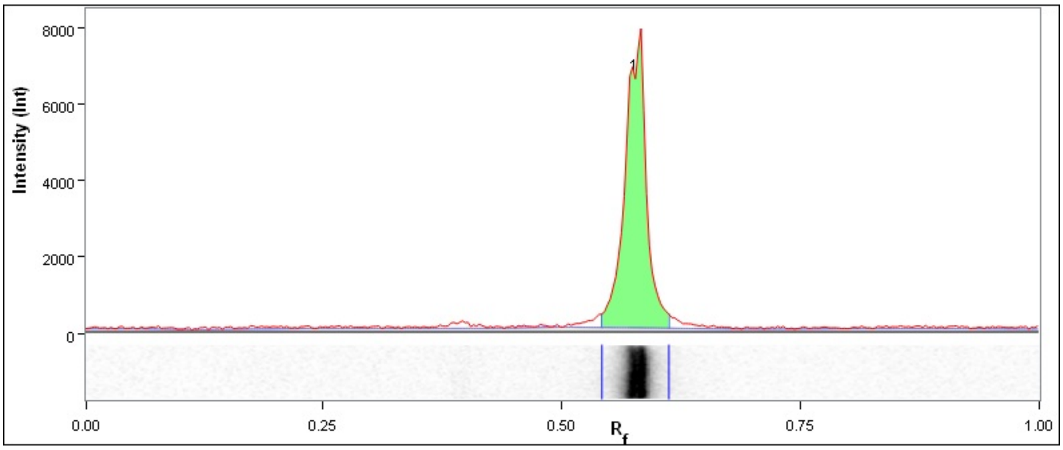

Band No.	Band Label	Mol. Wt. (KDa)	Relative Front	Adj. Volume (Int)	Volume (Int)	Abs. Quant.	Rel. Quant.	Band %	Lane %
1		N/A	0,579	2 922 726	3 031 868	N/A	N/A	100,0	81,1

Band Detection	Automatically detected bands with sensitivity: Low
Lane Background	Lane background subtracted with disk size: 10
Lane Width	6.72 mm

Lane 6

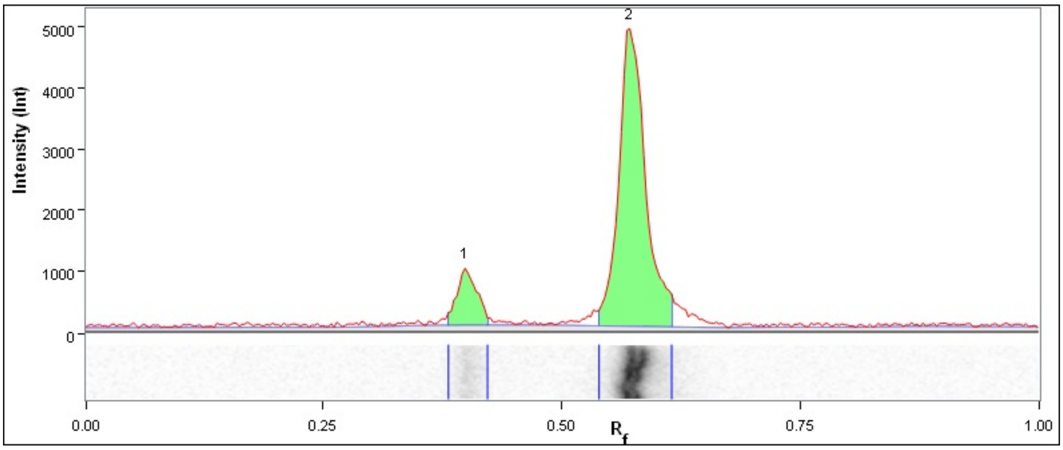

Band No.	Band Label	Mol. Wt. (KDa)	Relative Front	Adj. Volume (Int)	Volume (Int)	Abs. Quant.	Rel. Quant.	Band %	Lane %
1		N/A	0,401	332 797	401 554	N/A	N/A	12,6	10,2
2		N/A	0,573	2 313 138	2 415 556	N/A	N/A	87,4	70,9

Band Detection	Automatically detected bands with sensitivity: Low
Lane Background	Lane background subtracted with disk size: 10
Lane Width	6.72 mm

Lane 7

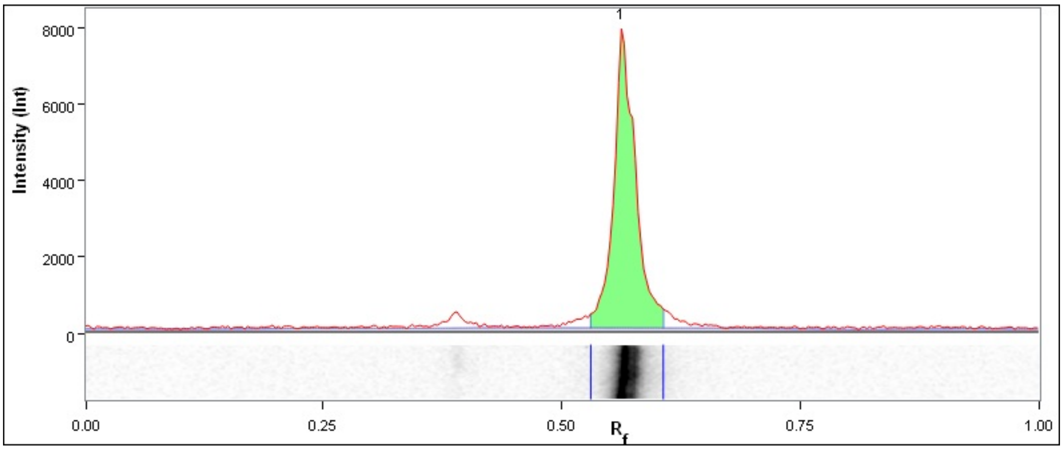

Band No.	Band Label	Mol. Wt. (KDa)	Relative Front	Adj. Volume (Int)	Volume (Int)	Abs. Quant.	Rel. Quant.	Band %	Lane %
1		N/A	0,564	3 154 786	3 270 652	N/A	N/A	100,0	78,5

Band Detection	Automatically detected bands with sensitivity: Low
Lane Background	Lane background subtracted with disk size: 10
Lane Width	6.72 mm

Volume Analysis

No.	Label	Type	Volume (Int)	Adj. Vol. (Int)	Mean Bkgd. (Int)	Abs. Quant.	Rel. Quant.	# of Pixels	Min. Value (Int)	Max. Value (Int)	Mean Value (Int)	Std. Dev.	Area (mm2)
1	U1	Unknown	979 664	426 668	246,4	N/A	N/A	2 244	0	2 656	436,6	464,5	60,2
2	U2	Unknown	3 115 488	2 073 763	464,2	N/A	N/A	2 244	0	9 676	1 388,4	1 791,6	60,2
3	U3	Unknown	4 393 496	3 344 276	467,6	N/A	N/A	2 244	0	11 700	1 957,9	2 563,4	60,2
4	U4	Unknown	2 066 840	1 330 021	328,4	N/A	N/A	2 244	0	7 500	921,1	1 304,8	60,2
5	U5	Unknown	3 576 648	2 791 942	349,7	N/A	N/A	2 244	0	10 116	1 593,9	2 252,3	60,2
6	U6	Unknown	3 016 968	2 071 851	421,2	N/A	N/A	2 244	0	6 620	1 344,5	1 570,5	60,2
7	U7	Unknown	3 876 192	2 887 675	440,5	N/A	N/A	2 244	0	10 032	1 727,4	2 270,1	60,2

Image Report: Histologia 2023-01-17 16hr 16min_Exposure_16.8sec 1a pERK

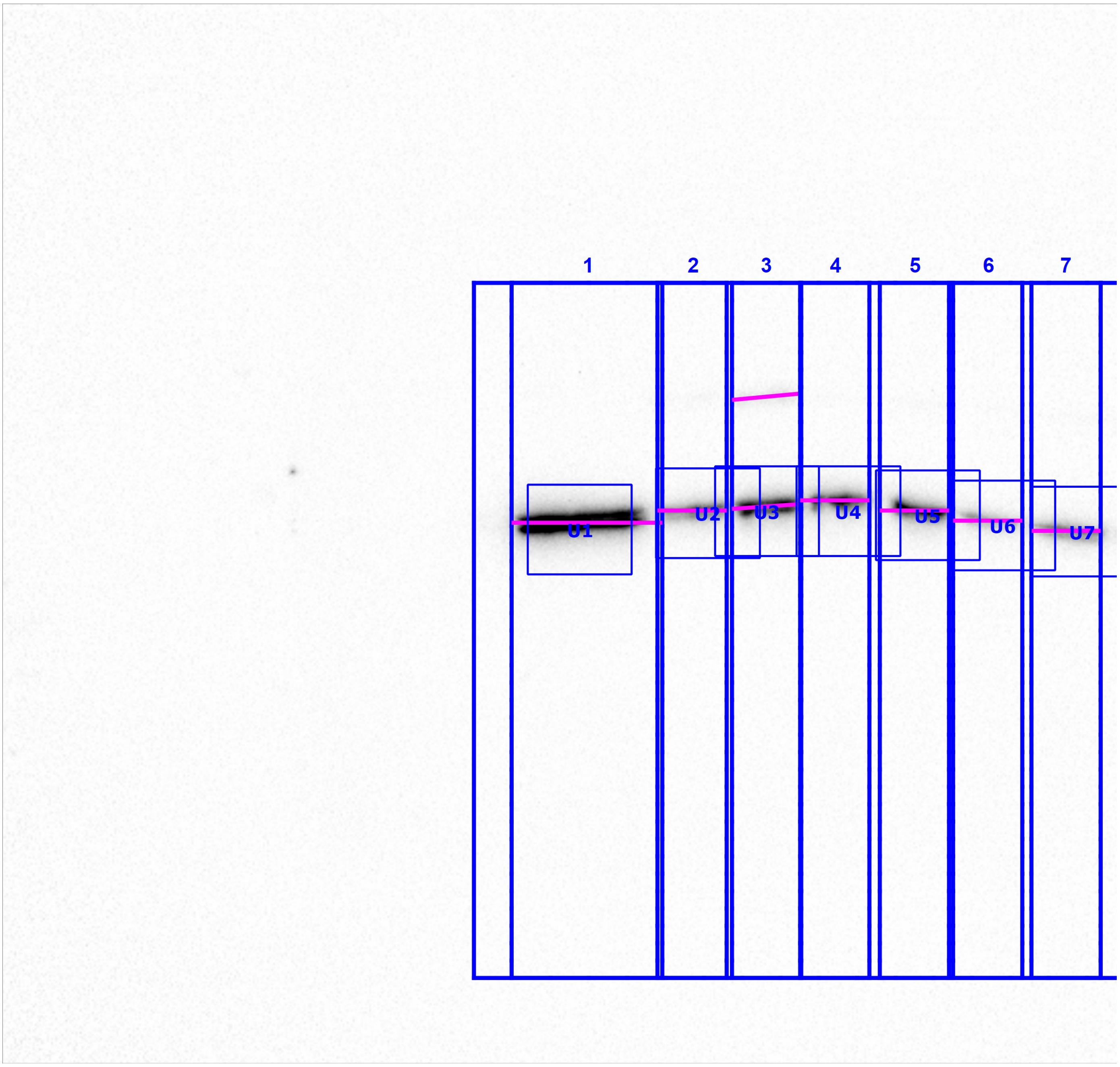

C:\Users\rusak\OneDrive\Dokumenty\Badania\CHI3L2 in BC\BC westerny ilościowo\pERK BC 17.1.23\1\Histologia 2023-01-17 16hr 16min_Exposure_16.8sec 1a pERK.scn

Acquisition Information

Imager	ChemiDoc MP
Exposure Time (sec)	16.800 (Signal Accumulation)
Flat Field	Applied (Lens)

Serial Number	731BR01769
Software Version	5.0
Application	Chemi Hi Resolution
Excitation Source	No Illumination
Emission Filter	No Filter
Binning	2x2

Image Information

Acquisition Date	17/1/2023 4:17:30 PM
User Name	Histologia
Image Area (mm)	X: 114.0 Y: 85.2
Pixel Size (μm)	X: 163.8 Y: 163.8
Data Range (Int)	0 - 29308

Analysis Settings

Detection	Lane detection:<br>Manually created lanes<br><br>Band detection:<br>Automatically detected bands with sensitivity: Low<br><br>Lane Background Subtraction:<br>Lane background subtracted with disk size: 10<br><br>Lane width: Variable
Volume Analysis	Background subtraction method: Local<br>Quantity regression method: Linear

Lane Statistics

Lane No.	Adj. Total Band Vol. (Int)	Total Band Vol. (Int)	Adj. Total Lane Vol. (Int)	Total Lane Vol. (Int)	Bkgd. Vol. (Int)	Norm. Factor
1	14 168 410	14 398 772	15 505 146	17 906 890	2 401 744	N/A
2	2 413 966	2 489 276	3 083 664	4 076 770	993 106	N/A
3	4 413 540	4 561 236	4 969 610	6 105 720	1 136 110	N/A
4	3 345 158	3 412 342	4 068 576	4 942 274	873 698	N/A
5	3 418 360	3 490 168	4 087 752	5 035 230	947 478	N/A
6	1 177 692	1 229 542	1 847 322	2 714 390	867 068	N/A
7	1 921 646	1 987 674	2 554 896	3 473 746	918 850	N/A
8	1 742 008	1 809 688	2 429 430	3 799 339	1 369 909	N/A

Lane And Band Analysis

Lane 1

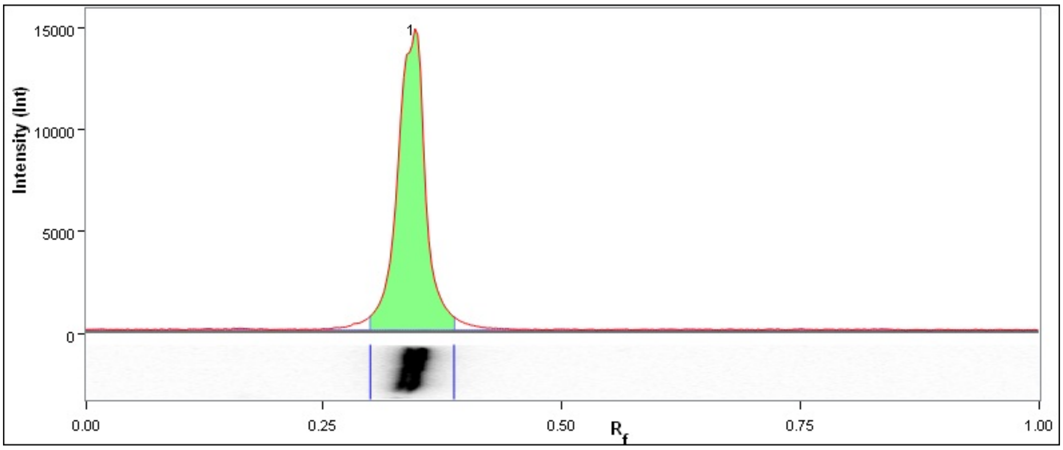

Band No.	Band Label	Mol. Wt. (KDa)	Relative Front	Adj. Volume (Int)	Volume (Int)	Abs. Quant.	Rel. Quant.	Band %	Lane %
1		N/A	0,345	14 168 410	14 398 772	N/A	N/A	100,0	91,4

Band Detection	Automatically detected bands with sensitivity: Low
Lane Background	Lane background subtracted with disk size: 10
Lane Width	12.12 mm

Lane 2

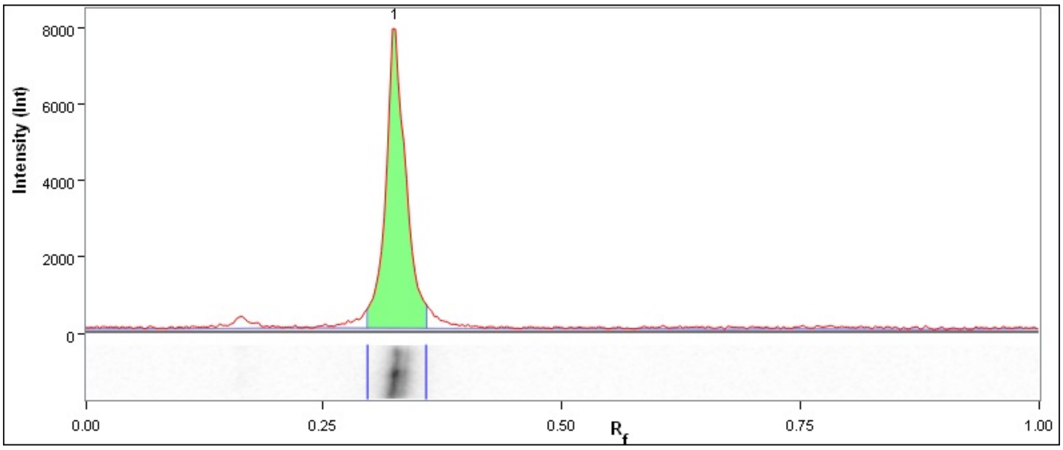

Band No.	Band Label	Mol. Wt. (KDa)	Relative Front	Adj. Volume (Int)	Volume (Int)	Abs. Quant.	Rel. Quant.	Band %	Lane %
1		N/A	0,327	2 413 966	2 489 276	N/A	N/A	100,0	78,3

Band Detection	Automatically detected bands with sensitivity: Low
Lane Background	Lane background subtracted with disk size: 10
Lane Width	5.57 mm

Lane 3

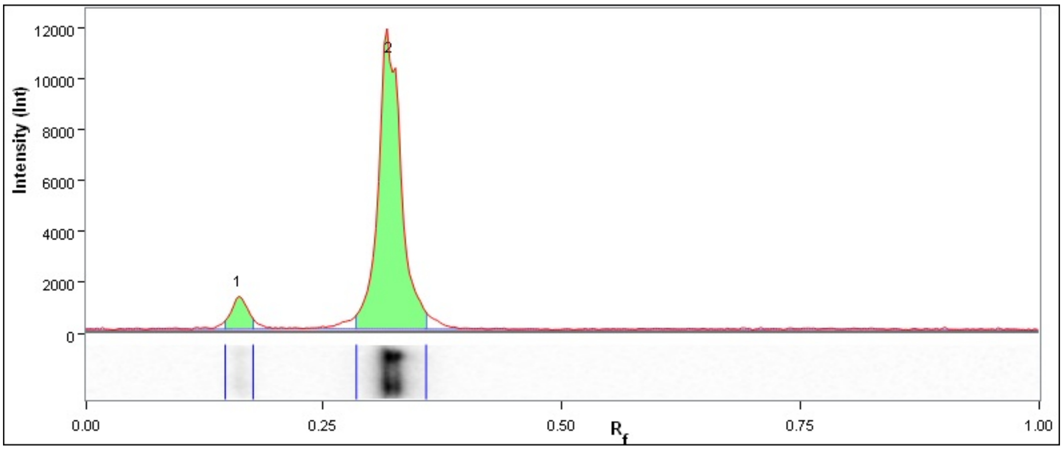

Band No.	Band Label	Mol. Wt. (KDa)	Relative Front	Adj. Volume (Int)	Volume (Int)	Abs. Quant.	Rel. Quant.	Band %	Lane %
1		N/A	0,164	320 892	363 698	N/A	N/A	7,3	6,5
2		N/A	0,322	4 092 648	4 197 538	N/A	N/A	92,7	82,4

Band Detection	Automatically detected bands with sensitivity: Low
Lane Background	Lane background subtracted with disk size: 10
Lane Width	5.57 mm

Lane 4

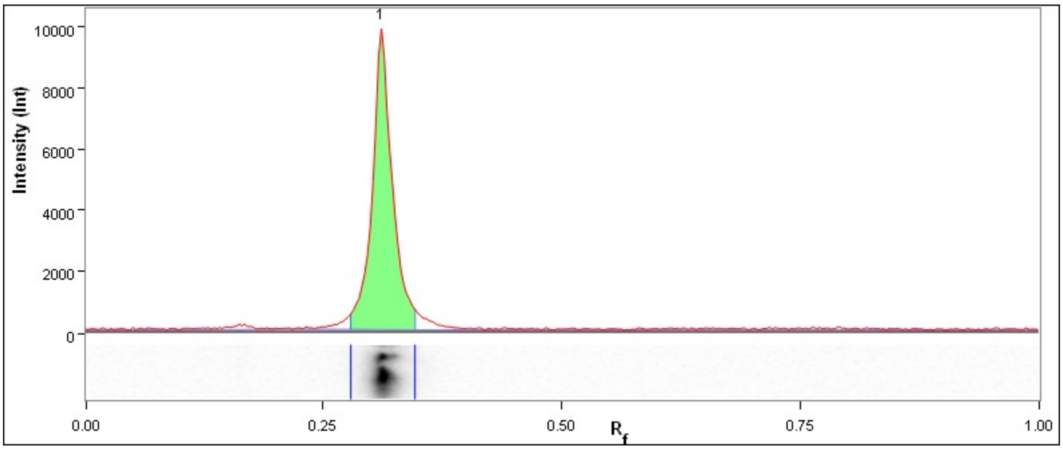

Band No.	Band Label	Mol. Wt. (KDa)	Relative Front	Adj. Volume (Int)	Volume (Int)	Abs. Quant.	Rel. Quant.	Band %	Lane %
1		N/A	0,313	3 345 158	3 412 342	N/A	N/A	100,0	82,2

Band Detection	Automatically detected bands with sensitivity: Low
Lane Background	Lane background subtracted with disk size: 10
Lane Width	5.57 mm

Lane 5

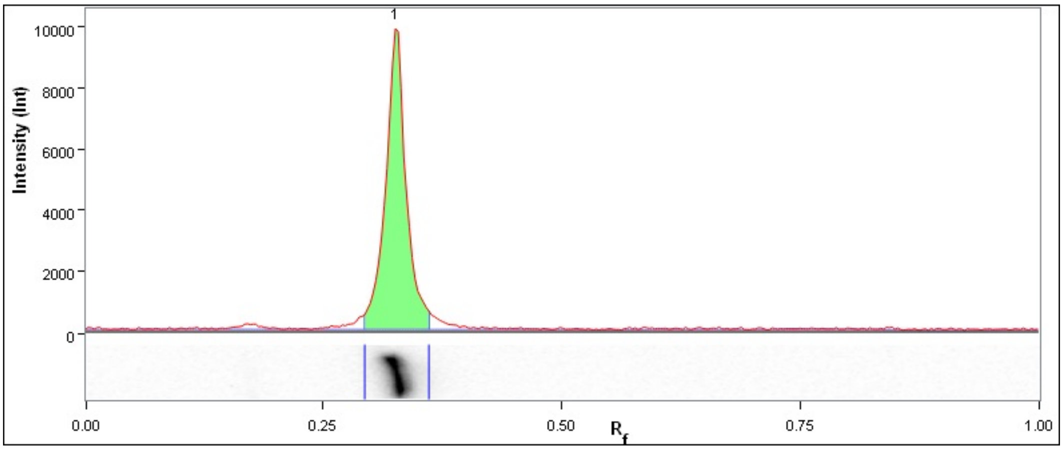

Band No.	Band Label	Mol. Wt. (KDa)	Relative Front	Adj. Volume (Int)	Volume (Int)	Abs. Quant.	Rel. Quant.	Band %	Lane %
1		N/A	0,327	3 418 360	3 490 168	N/A	N/A	100,0	83,6

Band Detection	Automatically detected bands with sensitivity: Low
Lane Background	Lane background subtracted with disk size: 10
Lane Width	5.57 mm

Lane 6

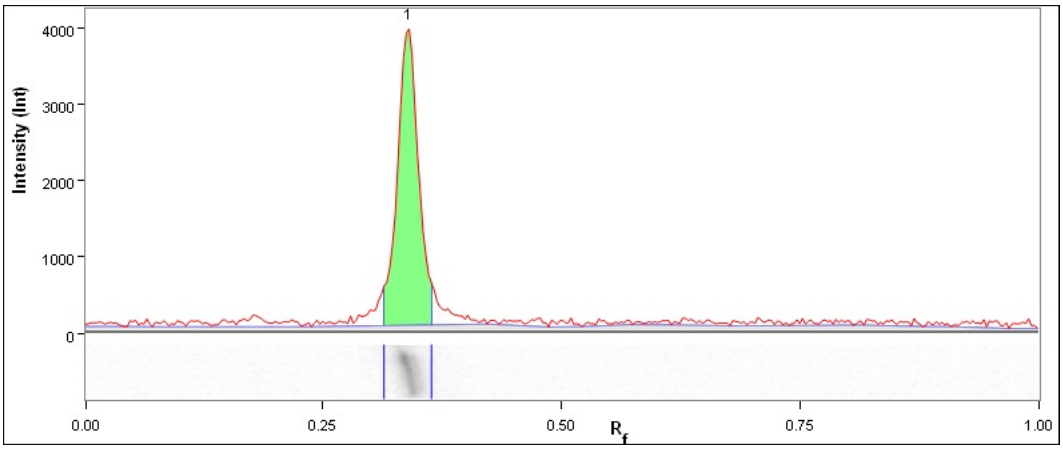

Band No.	Band Label	Mol. Wt. (KDa)	Relative Front	Adj. Volume (Int)	Volume (Int)	Abs. Quant.	Rel. Quant.	Band %	Lane %
1		N/A	0,342	1 177 692	1 229 542	N/A	N/A	100,0	63,8

Band Detection	Automatically detected bands with sensitivity: Low
Lane Background	Lane background subtracted with disk size: 10
Lane Width	5.57 mm

Lane 7

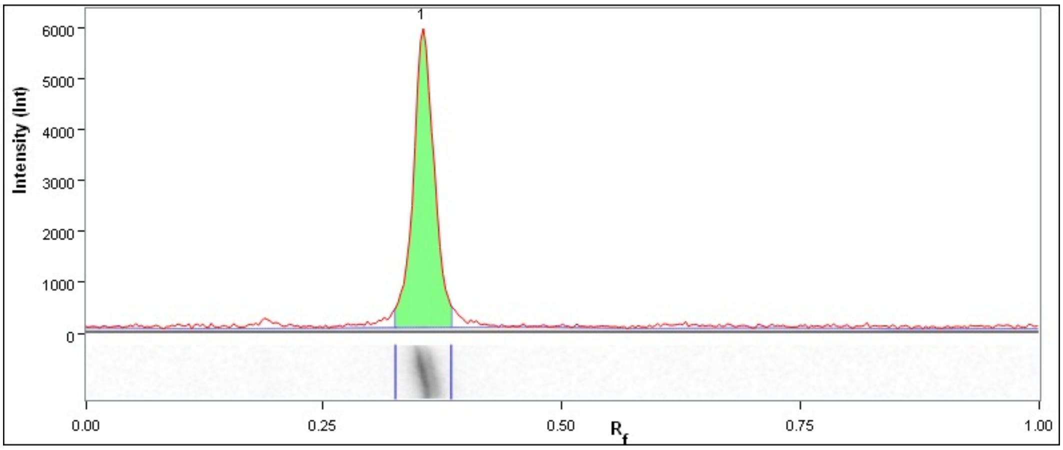

Band No.	Band Label	Mol. Wt. (KDa)	Relative Front	Adj. Volume (Int)	Volume (Int)	Abs. Quant.	Rel. Quant.	Band %	Lane %
1		N/A	0,357	1 921 646	1 987 674	N/A	N/A	100,0	75,2

Band Detection	Automatically detected bands with sensitivity: Low
Lane Background	Lane background subtracted with disk size: 10
Lane Width	5.57 mm

Lane 8

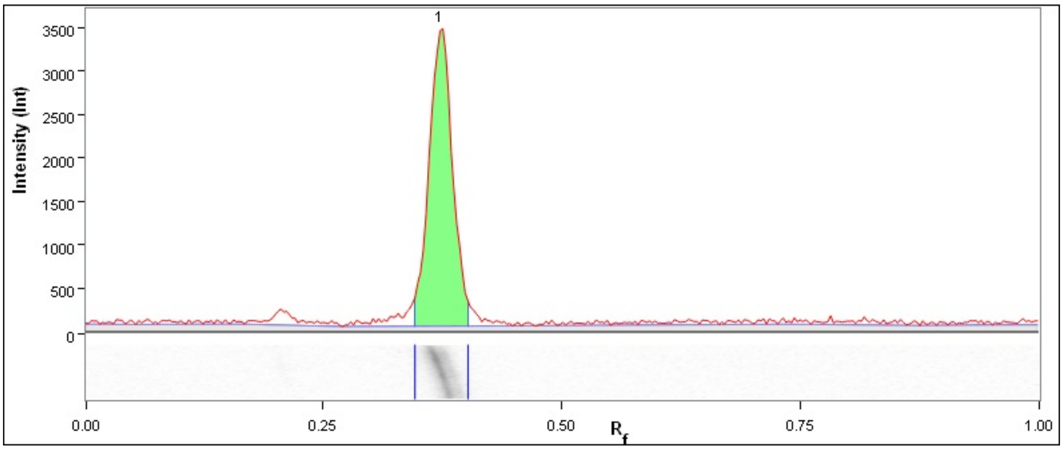

Band No.	Band Label	Mol. Wt. (KDa)	Relative Front	Adj. Volume (Int)	Volume (Int)	Abs. Quant.	Rel. Quant.	Band %	Lane %
1		N/A	0,374	1 742 008	1 809 688	N/A	N/A	100,0	71,7

Band Detection	Automatically detected bands with sensitivity: Low
Lane Background	Lane background subtracted with disk size: 10
Lane Width	7.70 mm

Volume Analysis

No.	Label	Type	Volume (Int)	Adj. Vol. (Int)	Mean Bkgd. (Int)	Abs. Quant.	Rel. Quant.	# of Pixels	Min. Value (Int)	Max. Value (Int)	Mean Value (Int)	Std. Dev.	Area (mm2)
1	U1	Unknown	12 354 792	7 048 819	2 364,5	N/A	N/A	2 244	0	29 308	5 505,7	7 018,2	60,2
2	U2	Unknown	4 694 872	2 495 336	980,2	N/A	N/A	2 244	0	18 668	2 092,2	3 051,6	60,2
3	U3	Unknown	5 845 432	3 202 833	1 177,6	N/A	N/A	2 244	0	18 668	2 604,9	3 665,4	60,2

4	U4	Unknown	4 712 180	1 454 771	1 451,6	N/A	N/A	2 244	0	20 276	2 099,9	3 235,9	60,2
5	U5	Unknown	4 457 640	3 276 047	526,6	N/A	N/A	2 244	0	20 276	1 986,5	3 399,9	60,2
6	U6	Unknown	2 227 356	675 850	691,4	N/A	N/A	2 244	0	8 680	992,6	1 385,9	60,2
7	U7	Unknown	2 718 272	1 643 697	478,9	N/A	N/A	2 244	0	9 784	1 211,4	1 828,3	60,2
8	U8	Unknown	2 120 328	1 482 569	284,2	N/A	N/A	2 244	0	7 336	944,9	1 396,2	60,2

Image Report: Histologia 2023-01-26 12hr 02min_Exposure_3.0sec 1 b

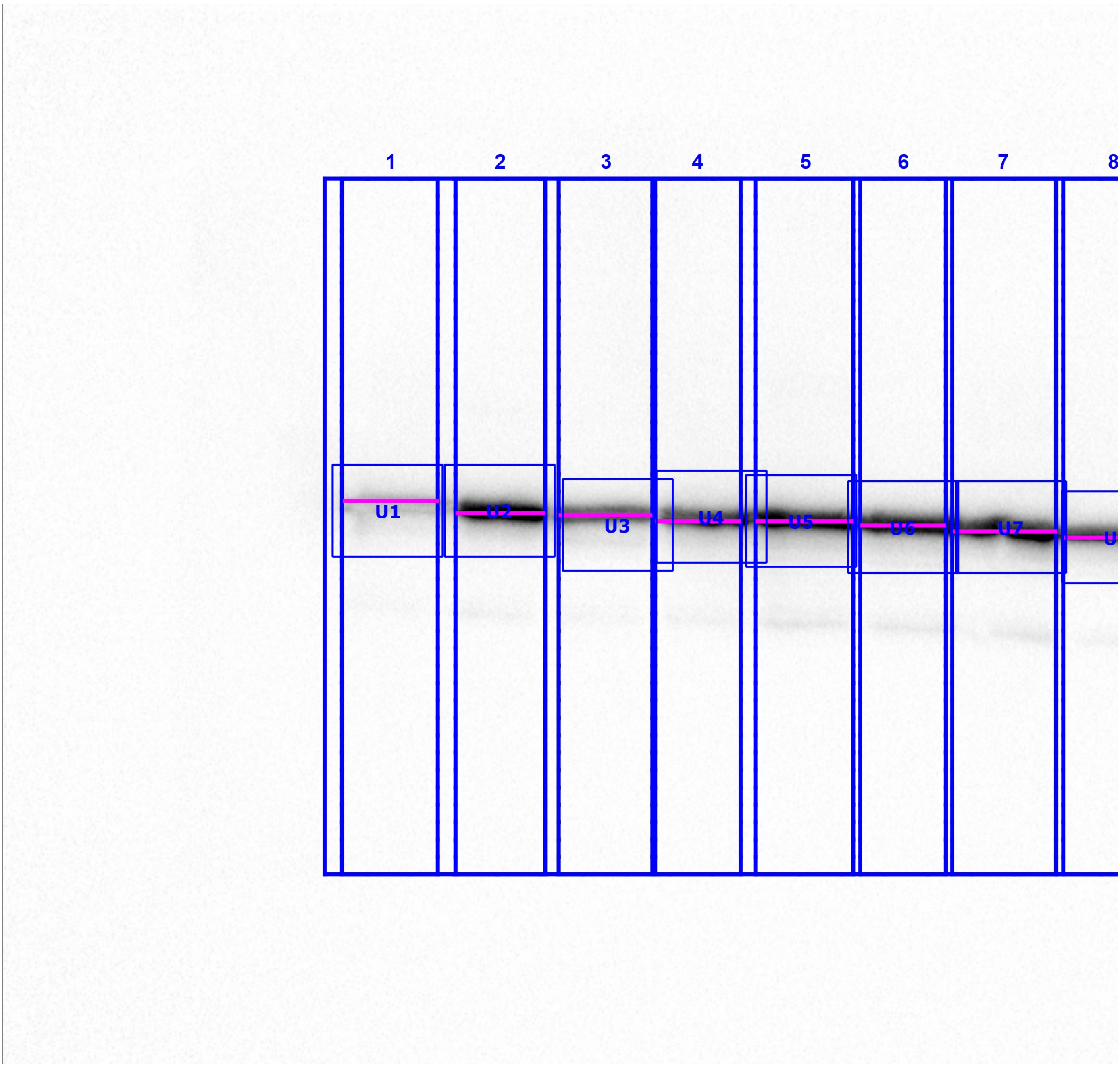

C:\Users\rusak\OneDrive\Dokumenty\Badania\CHI3L2 in BC\BC westerny ilościowo\ERK 26.1.23\1\Histologia 2023-01-26 12hr 02min_Exposure_3.0sec 1 b.scn

Acquisition Information

Imager	ChemiDoc MP
Exposure Time (sec)	3.000 (Signal Accumulation)
Flat Field	Applied (Lens)

Serial Number	731BR01769
Software Version	5.0
Application	Chemi Hi Resolution
Excitation Source	No Illumination
Emission Filter	No Filter
Binning	2x2

Image Information

Acquisition Date	26/1/2023 12:02:35 PM
User Name	Histologia
Image Area (mm)	X: 114.0 Y: 85.2
Pixel Size (µm)	X: 163.8 Y: 163.8
Data Range (Int)	0 - 32732

Analysis Settings

Detection	Lane detection:<br>Manually created lanes<br><br>Band detection:<br>Automatically detected bands with sensitivity: Low<br><br>Lane Background Subtraction:<br>Lane background subtracted with disk size: 10<br><br>Lane width: Variable
Volume Analysis	Background subtraction method: Local<br>Quantity regression method: Linear

Lane Statistics

Lane No.	Adj. Total Band Vol. (Int)	Total Band Vol. (Int)	Adj. Total Lane Vol. (Int)	Total Lane Vol. (Int)	Bkgd. Vol. (Int)	Norm. Factor
1	3 717 888	4 039 744	5 927 217	9 361 178	3 433 961	N/A
2	12 860 144	13 381 984	14 479 388	18 009 992	3 530 604	N/A
3	8 135 054	8 597 952	9 609 262	13 595 530	3 986 268	N/A
4	8 866 956	9 385 572	11 120 424	14 826 378	3 705 954	N/A
5	13 244 304	14 234 880	15 478 608	21 024 288	5 545 680	N/A
6	10 783 332	11 256 252	12 845 910	16 098 180	3 252 270	N/A
7	14 040 453	14 686 878	16 344 174	20 577 633	4 233 459	N/A
8	10 189 060	10 530 345	12 190 857	15 362 774	3 171 917	N/A

Lane And Band Analysis

Lane 1

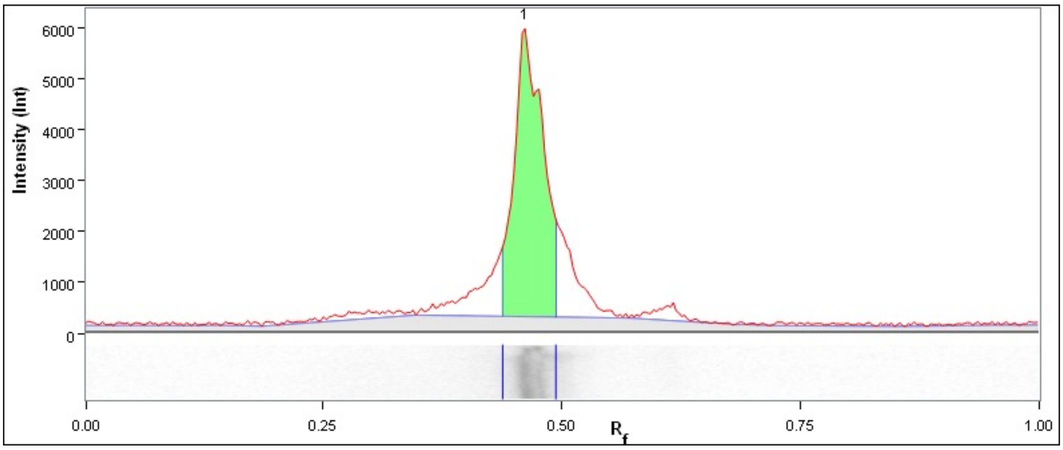

Band No.	Band Label	Mol. Wt. (KDa)	Relative Front	Adj. Volume (Int)	Volume (Int)	Abs. Quant.	Rel. Quant.	Band %	Lane %
1		N/A	0,463	3 717 888	4 039 744	N/A	N/A	100,0	62,7

Band Detection	Automatically detected bands with sensitivity: Low
Lane Background	Lane background subtracted with disk size: 10
Lane Width	7.70 mm

Lane 2

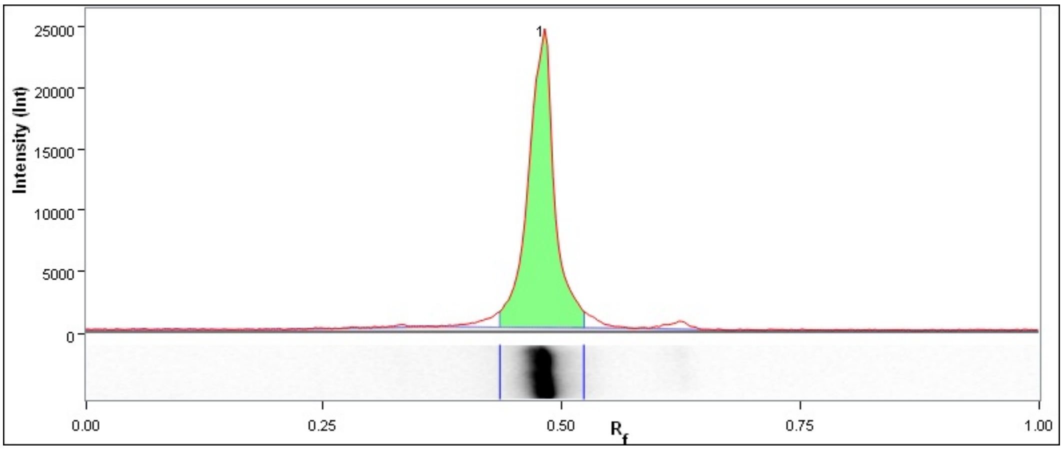

Band No.	Band Label	Mol. Wt. (KDa)	Relative Front	Adj. Volume (Int)	Volume (Int)	Abs. Quant.	Rel. Quant.	Band %	Lane %
1		N/A	0,481	12 860 144	13 381 984	N/A	N/A	100,0	88,8

Band Detection	Automatically detected bands with sensitivity: Low
Lane Background	Lane background subtracted with disk size: 10
Lane Width	7.21 mm

Lane 3

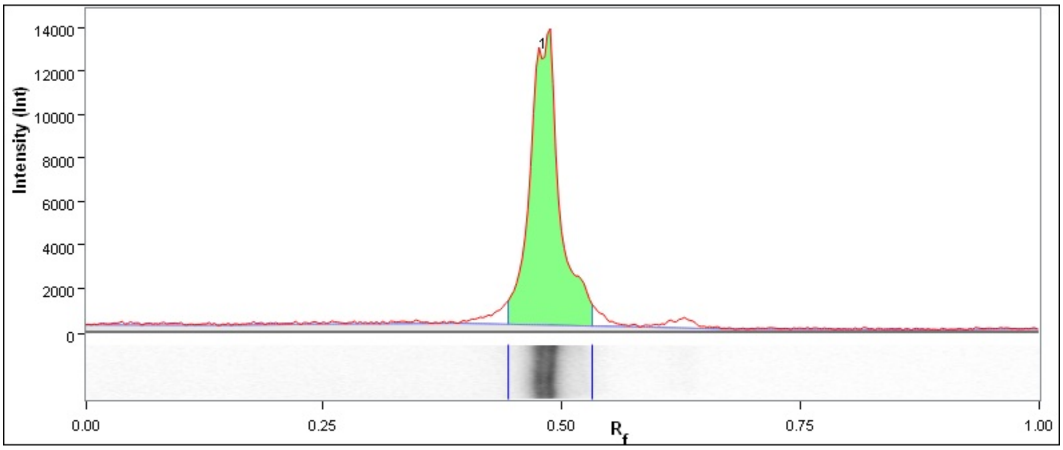

Band No.	Band Label	Mol. Wt. (KDa)	Relative Front	Adj. Volume (Int)	Volume (Int)	Abs. Quant.	Rel. Quant.	Band %	Lane %
1		N/A	0,484	8 135 054	8 597 952	N/A	N/A	100,0	84,7

Band Detection	Automatically detected bands with sensitivity: Low
Lane Background	Lane background subtracted with disk size: 10
Lane Width	7.53 mm

Lane 4

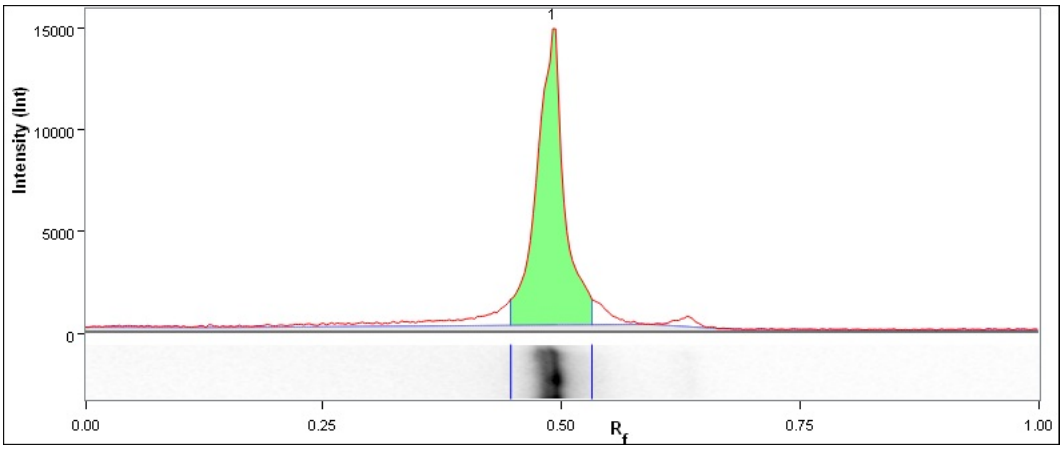

Band No.	Band Label	Mol. Wt. (KDa)	Relative Front	Adj. Volume (Int)	Volume (Int)	Abs. Quant.	Rel. Quant.	Band %	Lane %
1		N/A	0,493	8 866 956	9 385 572	N/A	N/A	100,0	79,7

Band Detection	Automatically detected bands with sensitivity: Low
Lane Background	Lane background subtracted with disk size: 10
Lane Width	6.88 mm

Lane 5

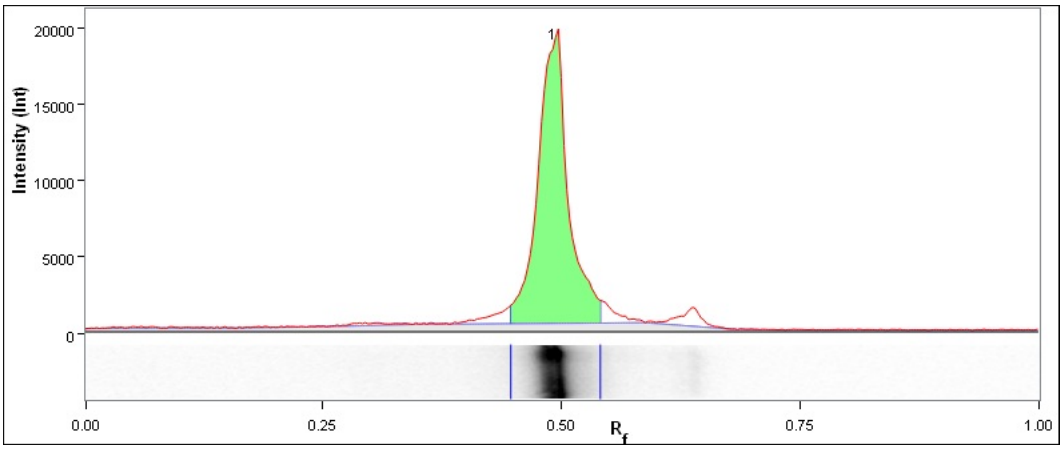

Band No.	Band Label	Mol. Wt. (KDa)	Relative Front	Adj. Volume (Int)	Volume (Int)	Abs. Quant.	Rel. Quant.	Band %	Lane %
1		N/A	0,493	13 244 304	14 234 880	N/A	N/A	100,0	85,6

Band Detection	Automatically detected bands with sensitivity: Low
Lane Background	Lane background subtracted with disk size: 10
Lane Width	7.86 mm

Lane 6

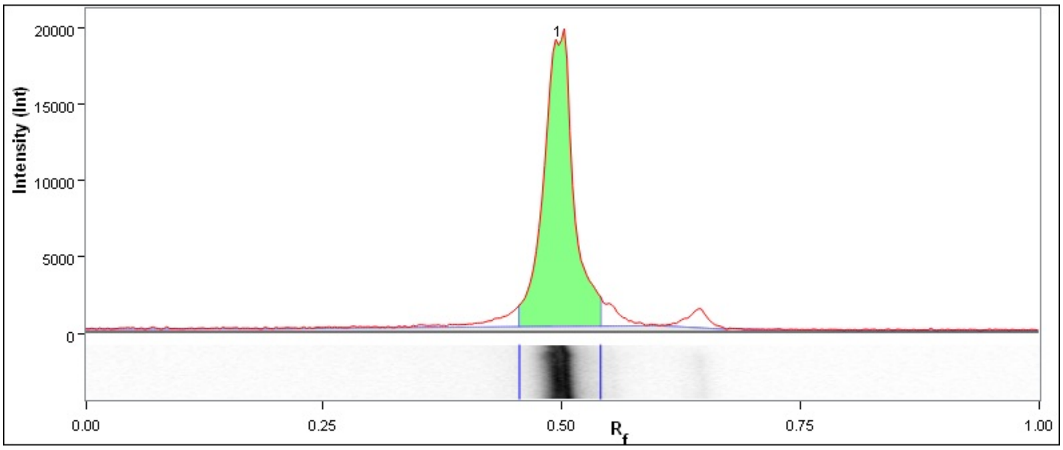

Band No.	Band Label	Mol. Wt. (KDa)	Relative Front	Adj. Volume (Int)	Volume (Int)	Abs. Quant.	Rel. Quant.	Band %	Lane %
1		N/A	0,499	10 783 332	11 256 252	N/A	N/A	100,0	83,9

Band Detection	Automatically detected bands with sensitivity: Low
Lane Background	Lane background subtracted with disk size: 10
Lane Width	6.88 mm

Lane 7

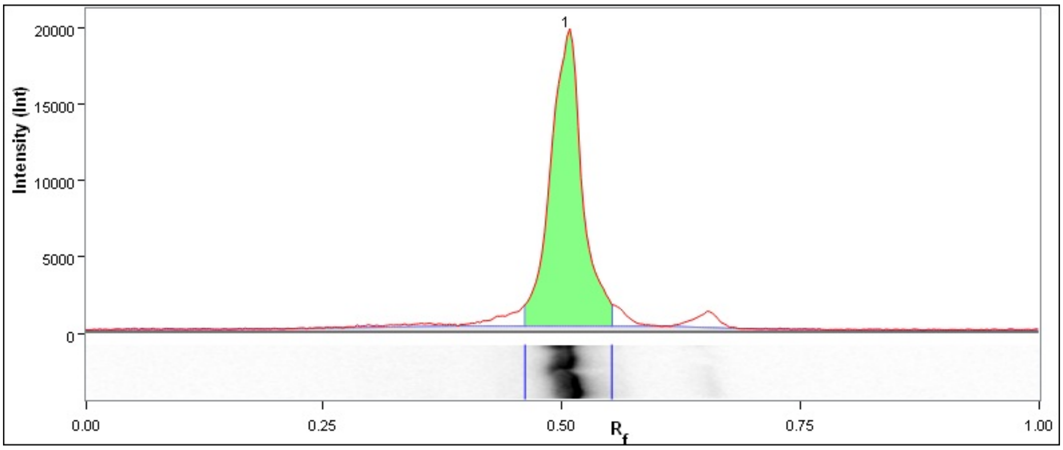

Band No.	Band Label	Mol. Wt. (KDa)	Relative Front	Adj. Volume (Int)	Volume (Int)	Abs. Quant.	Rel. Quant.	Band %	Lane %
1		N/A	0,507	14 040 453	14 686 878	N/A	N/A	100,0	85,9

Band Detection	Automatically detected bands with sensitivity: Low
Lane Background	Lane background subtracted with disk size: 10
Lane Width	8.35 mm

Lane 8

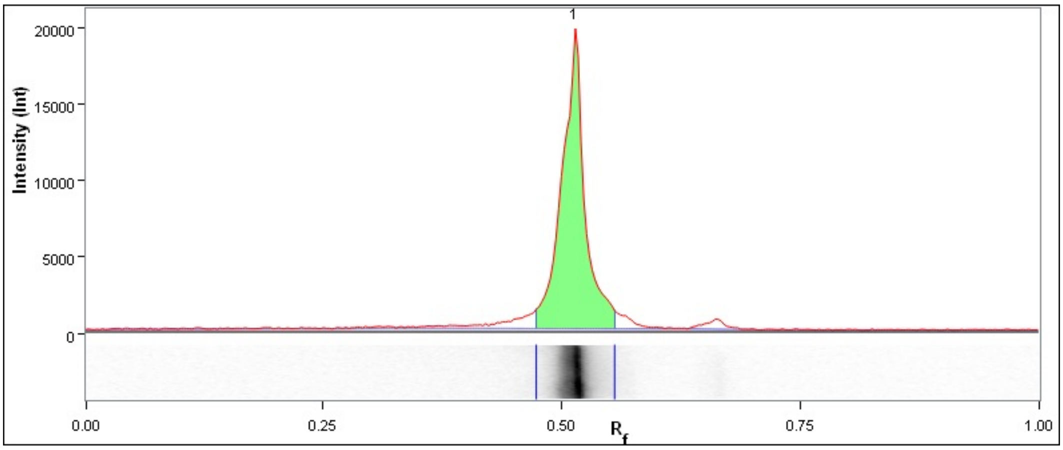

Band No.	Band Label	Mol. Wt. (KDa)	Relative Front	Adj. Volume (Int)	Volume (Int)	Abs. Quant.	Rel. Quant.	Band %	Lane %
1		N/A	0,516	10 189 060	10 530 345	N/A	N/A	100,0	83,6

Band Detection	Automatically detected bands with sensitivity: Low
Lane Background	Lane background subtracted with disk size: 10
Lane Width	8.03 mm

Volume Analysis

No.	Label	Type	Volume (Int)	Adj. Vol. (Int)	Mean Bkgd. (Int)	Abs. Quant.	Rel. Quant.	# of Pixels	Min. Value (Int)	Max. Value (Int)	Mean Value (Int)	Std. Dev.	Area (mm2)
1	U1	Unknown	6 128 292	3 181 255	1 212,8	N/A	N/A	2 430	0	8 584	2 521,9	1 916,0	65,2
2	U2	Unknown	15 566 088	10 971 078	1 891,0	N/A	N/A	2 430	0	32 732	6 405,8	7 707,5	65,2
3	U3	Unknown	10 423 404	3 989 919	2 647,5	N/A	N/A	2 430	0	18 488	4 289,5	4 463,9	65,2

4	U4	Unknown	13 786 312	6 626 666	2 946,4	N/A	N/A	2 430	8	27 320	5 673,4	5 796,0	65,2
5	U5	Unknown	16 700 004	8 669 744	3 304,6	N/A	N/A	2 430	400	28 052	6 872,4	6 785,7	65,2
6	U6	Unknown	15 661 852	7 456 151	3 376,8	N/A	N/A	2 430	276	25 808	6 445,2	6 625,1	65,2
7	U7	Unknown	16 096 108	8 343 253	3 190,5	N/A	N/A	2 430	308	27 400	6 623,9	6 764,3	65,2
8	U8	Unknown	12 174 172	7 731 651	1 828,2	N/A	N/A	2 430	0	25 436	5 009,9	5 722,0	65,2

Image Report: Histologia 2023-01-27 18hr 05min_Exposure_1.0sec 3c ERK

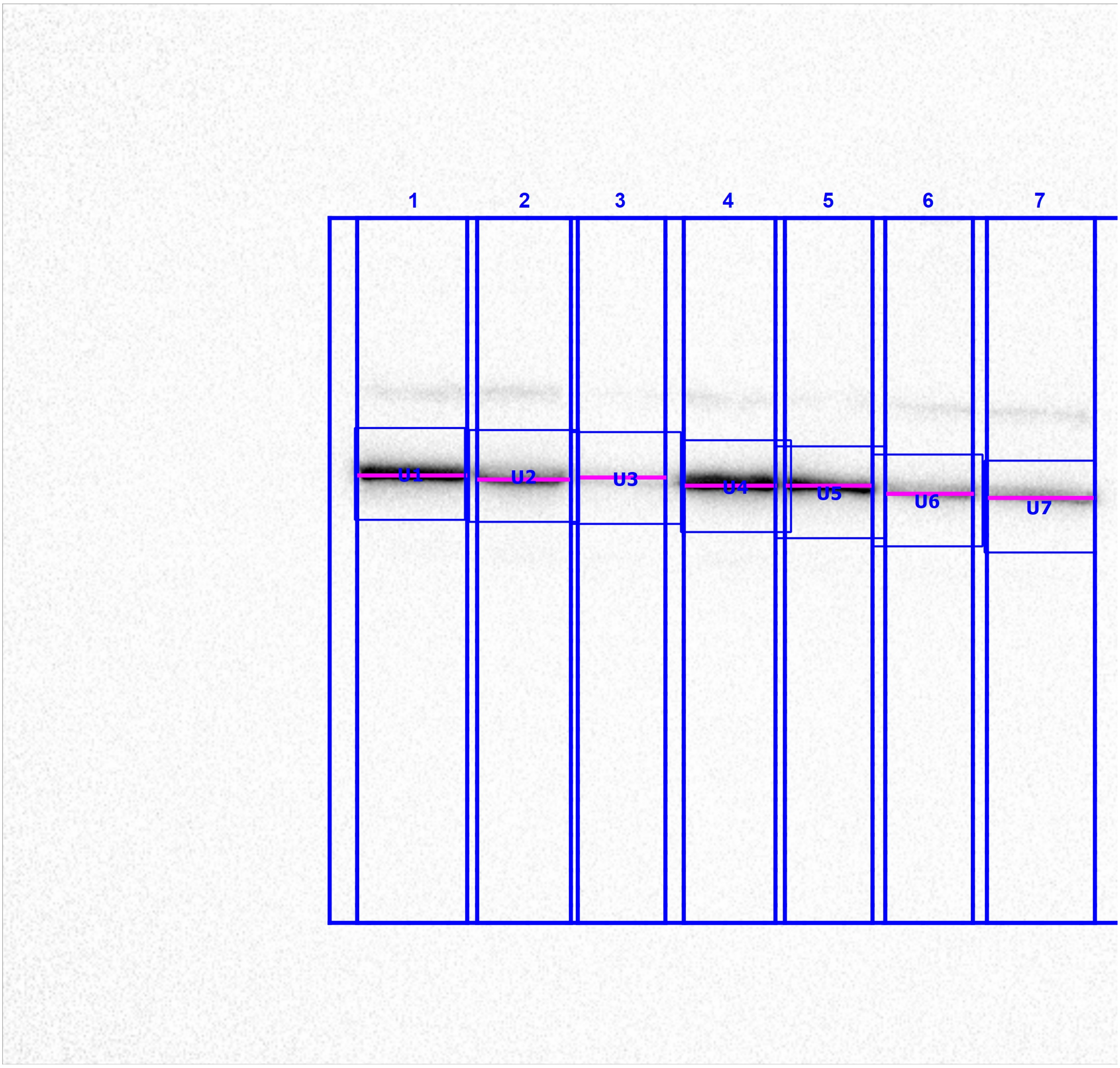

C:\Users\rusak\OneDrive\Dokumenty\Badania\CHI3L2 in BC\BC westerny ilościowo\ERK 27.1.23\3\Histologia 2023-01-27 18hr 05min_Exposure_1.0sec 3c ERK.scn

Acquisition Information

Imager	ChemiDoc MP
Exposure Time (sec)	3.000 (Signal Accumulation)
Flat Field	Applied (Lens)

Serial Number	731BR01769
Software Version	5.0
Application	Chemi Hi Resolution
Excitation Source	No Illumination
Emission Filter	No Filter
Binning	2x2

Image Information

Acquisition Date	27/1/2023 6:05:48 PM
User Name	Histologia
Image Area (mm)	X: 114.0 Y: 85.2
Pixel Size (µm)	X: 163.8 Y: 163.8
Data Range (Int)	0 - 17288

Analysis Settings

Detection	Lane detection:<br>Manually created lanes<br><br>Band detection:<br>Automatically detected bands with sensitivity: Low<br><br>Lane Background Subtraction:<br>Lane background subtracted with disk size: 10<br><br>Lane width: Variable
Volume Analysis	Background subtraction method: Local<br>Quantity regression method: Linear

Lane Statistics

Lane No.	Adj. Total Band Vol. (Int)	Total Band Vol. (Int)	Adj. Total Lane Vol. (Int)	Total Lane Vol. (Int)	Bkgd. Vol. (Int)	Norm. Factor
1	5 300 964	5 573 124	6 555 492	8 820 468	2 264 976	N/A
2	2 622 644	2 826 010	3 765 284	5 575 338	1 810 054	N/A
3	677 250	757 230	1 485 005	2 904 134	1 419 129	N/A
4	4 824 810	4 994 820	5 813 865	7 362 270	1 548 405	N/A
5	3 532 106	3 710 384	4 323 177	5 992 867	1 669 690	N/A
6	1 486 854	1 617 832	2 387 962	4 074 207	1 686 245	N/A
7	1 800 728	1 962 802	2 938 320	4 940 448	2 002 128	N/A

Lane And Band Analysis

Lane 1

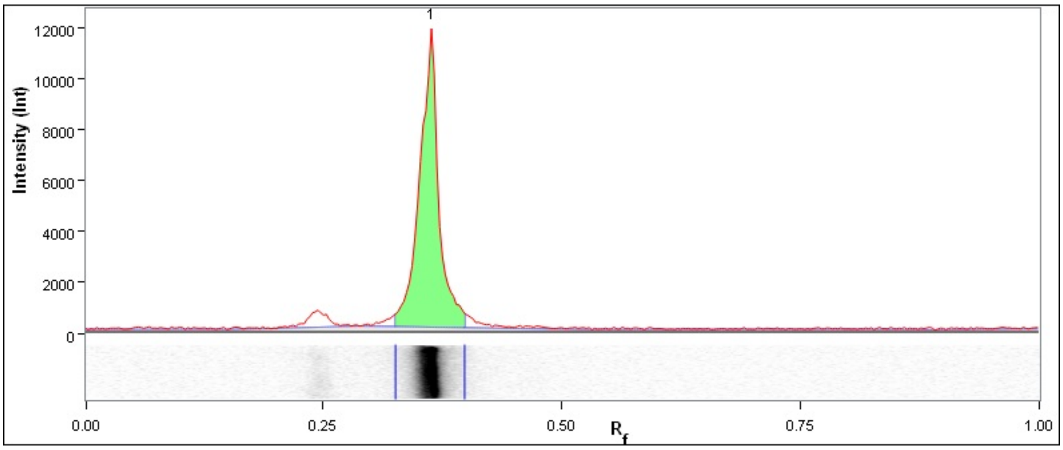

Band No.	Band Label	Mol. Wt. (KDa)	Relative Front	Adj. Volume (Int)	Volume (Int)	Abs. Quant.	Rel. Quant.	Band %	Lane %
1		N/A	0,365	5 300 964	5 573 124	N/A	N/A	100,0	80,9

Band Detection	Automatically detected bands with sensitivity: Low
Lane Background	Lane background subtracted with disk size: 10
Lane Width	8.84 mm

Lane 2

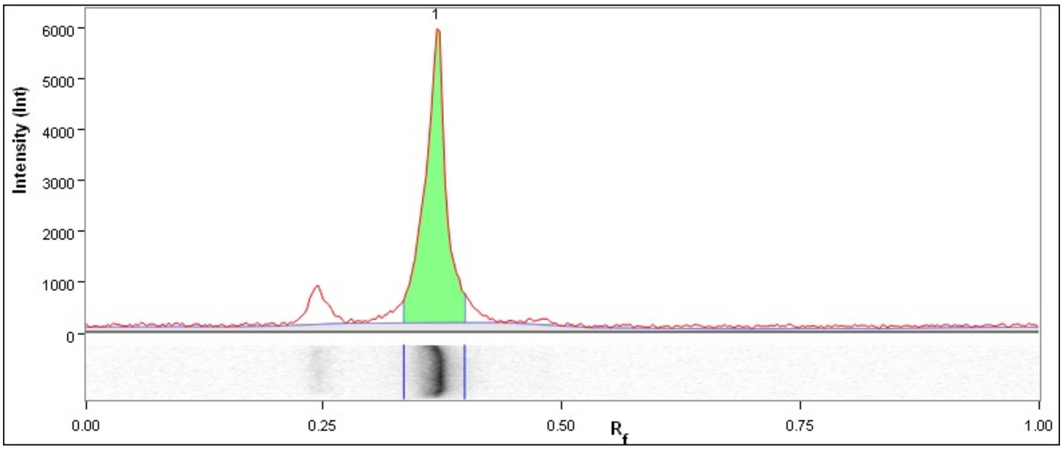

Band No.	Band Label	Mol. Wt. (KDa)	Relative Front	Adj. Volume (Int)	Volume (Int)	Abs. Quant.	Rel. Quant.	Band %	Lane %
1		N/A	0,371	2 622 644	2 826 010	N/A	N/A	100,0	69,7

Band Detection	Automatically detected bands with sensitivity: Low
Lane Background	Lane background subtracted with disk size: 10
Lane Width	7.53 mm

Lane 3

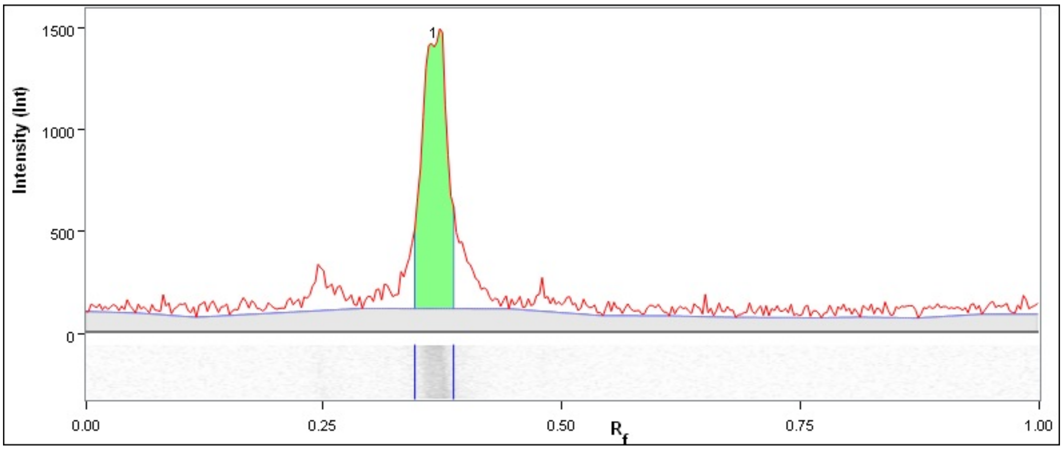

Band No.	Band Label	Mol. Wt. (KDa)	Relative Front	Adj. Volume (Int)	Volume (Int)	Abs. Quant.	Rel. Quant.	Band %	Lane %
1		N/A	0,368	677 250	757 230	N/A	N/A	100,0	45,6

Band Detection	Automatically detected bands with sensitivity: Low
Lane Background	Lane background subtracted with disk size: 10
Lane Width	7.04 mm

Lane 4

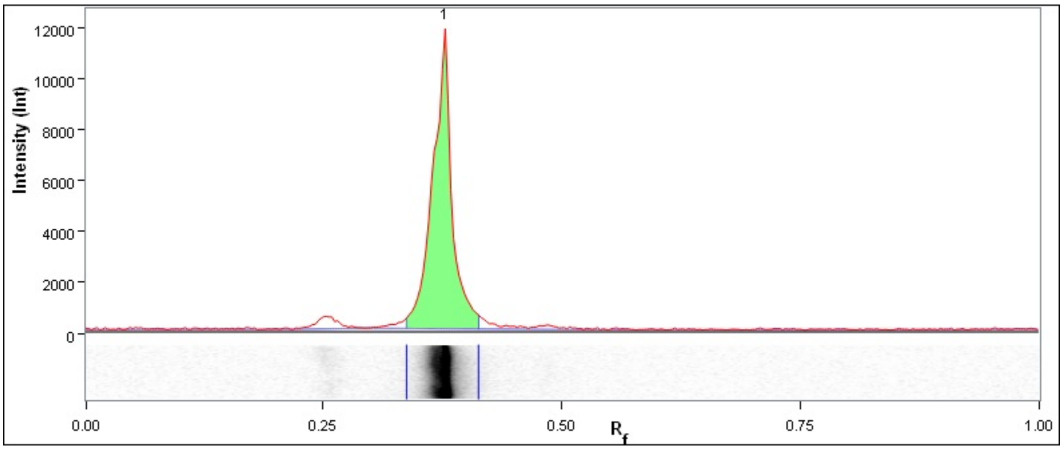

Band No.	Band Label	Mol. Wt. (KDa)	Relative Front	Adj. Volume (Int)	Volume (Int)	Abs. Quant.	Rel. Quant.	Band %	Lane %
1		N/A	0,380	4 824 810	4 994 820	N/A	N/A	100,0	83,0

Band Detection	Automatically detected bands with sensitivity: Low
Lane Background	Lane background subtracted with disk size: 10
Lane Width	7.37 mm

Lane 5

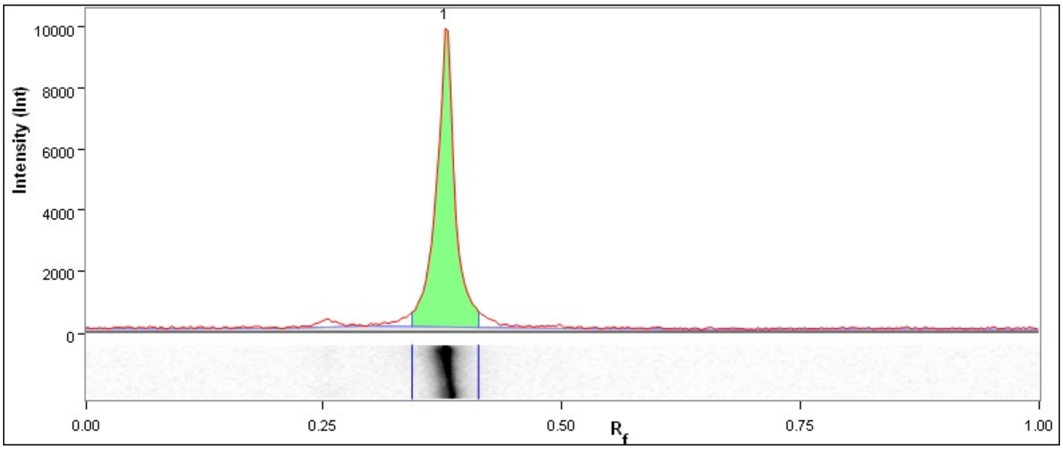

Band No.	Band Label	Mol. Wt. (KDa)	Relative Front	Adj. Volume (Int)	Volume (Int)	Abs. Quant.	Rel. Quant.	Band %	Lane %
1		N/A	0,380	3 532 106	3 710 384	N/A	N/A	100,0	81,7

Band Detection	Automatically detected bands with sensitivity: Low
Lane Background	Lane background subtracted with disk size: 10
Lane Width	7.04 mm

Lane 6

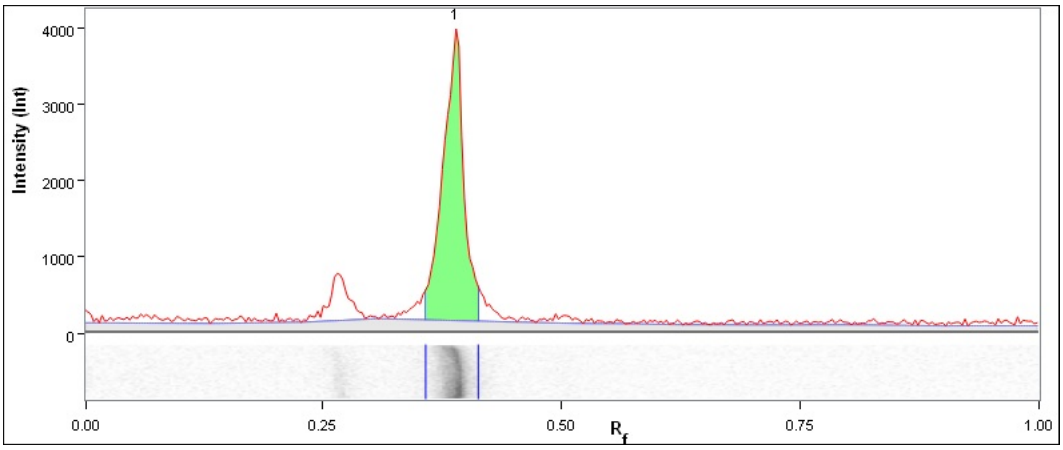

Band No.	Band Label	Mol. Wt. (KDa)	Relative Front	Adj. Volume (Int)	Volume (Int)	Abs. Quant.	Rel. Quant.	Band %	Lane %
1		N/A	0,391	1 486 854	1 617 832	N/A	N/A	100,0	62,3

Band Detection	Automatically detected bands with sensitivity: Low
Lane Background	Lane background subtracted with disk size: 10
Lane Width	7.04 mm

Lane 7

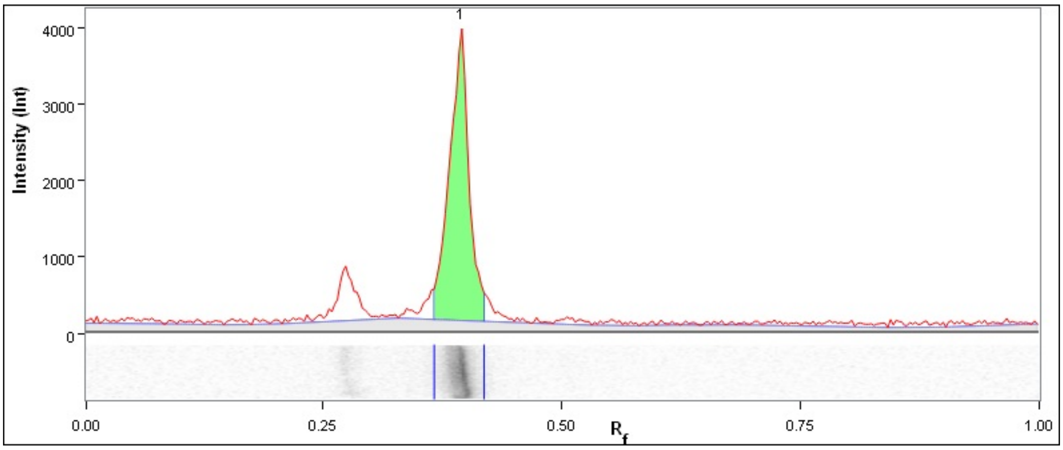

Band No.	Band Label	Mol. Wt. (KDa)	Relative Front	Adj. Volume (Int)	Volume (Int)	Abs. Quant.	Rel. Quant.	Band %	Lane %
1		N/A	0,397	1 800 728	1 962 802	N/A	N/A	100,0	61,3

Band Detection	Automatically detected bands with sensitivity: Low
Lane Background	Lane background subtracted with disk size: 10
Lane Width	8.68 mm

Volume Analysis

No.	Label	Type	Volume (Int)	Adj. Vol. (Int)	Mean Bkgd. (Int)	Abs. Quant.	Rel. Quant.	# of Pixels	Min. Value (Int)	Max. Value (Int)	Mean Value (Int)	Std. Dev.	Area (mm2)
1	U1	Unknown	5 978 024	4 267 304	704,0	N/A	N/A	2 430	0	16 288	2 460,1	3 295,8	65,2
2	U2	Unknown	3 573 652	2 177 388	574,6	N/A	N/A	2 430	0	8 668	1 470,6	1 731,3	65,2
3	U3	Unknown	1 534 156	225	631,2	N/A	N/A	2 430	0	6 024	631,3	643,5	65,2
4	U4	Unknown	6 067 216	3 628 603	1 003,5	N/A	N/A	2 430	0	17 288	2 496,8	3 325,1	65,2
5	U5	Unknown	4 671 736	2 593 869	855,1	N/A	N/A	2 430	0	15 644	1 922,5	2 693,4	65,2
6	U6	Unknown	2 340 004	941 912	575,3	N/A	N/A	2 430	0	6 780	963,0	1 142,1	65,2
7	U7	Unknown	2 373 072	1 357 717	417,8	N/A	N/A	2 430	0	6 212	976,6	1 204,2	65,2

Image Report: Histologia 2023-01-27 18hr 04min_Exposure_2.0sec 2c ERK

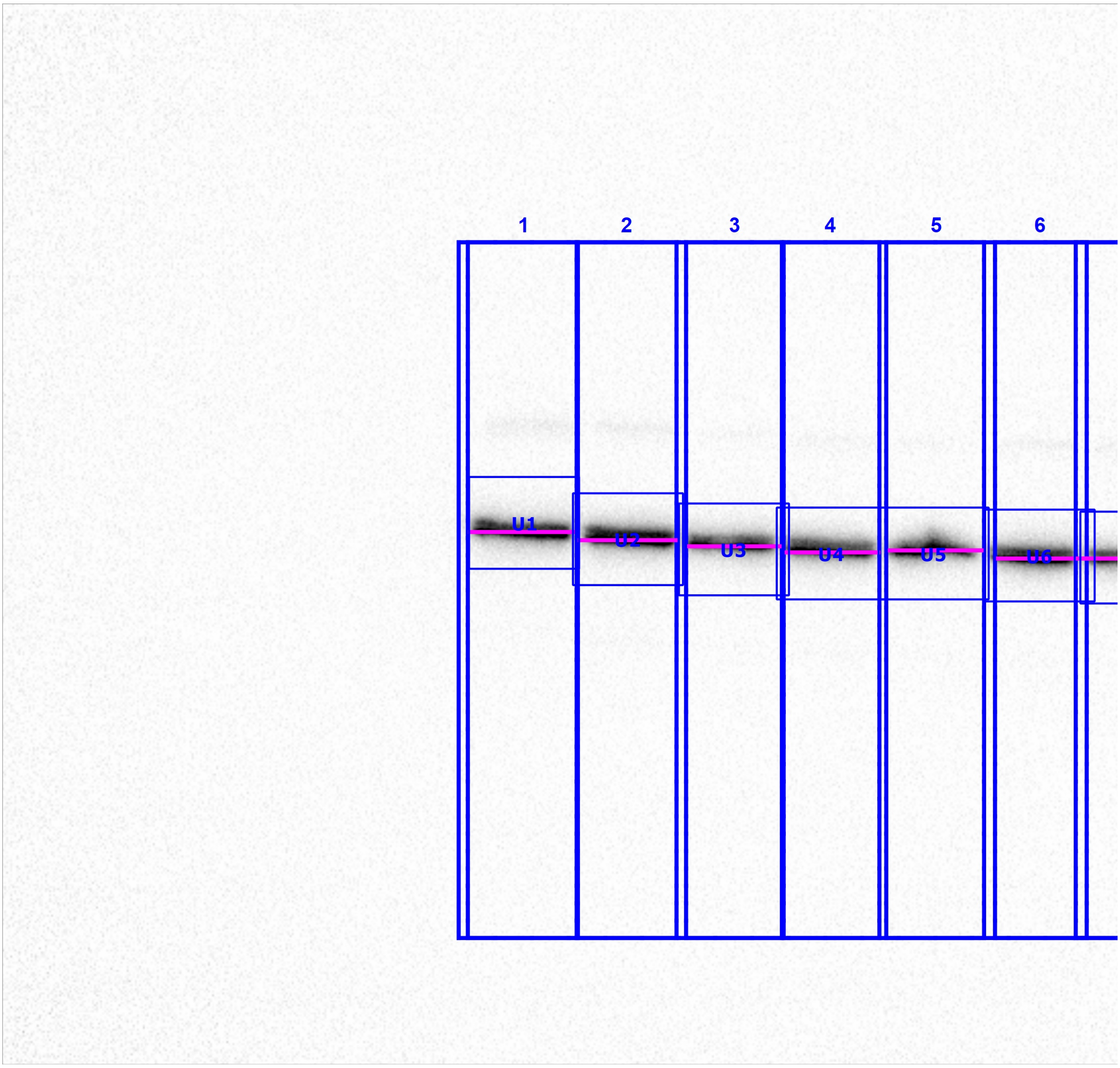

C:\Users\rusak\OneDrive\Dokumenty\Badania\CHI3L2 in BC\BC westerny ilościowo\ERK 27.1.23\2\Histologia 2023-01-27 18hr 04min_Exposure_2.0sec 2c ERK.scn

Acquisition Information

Imager	ChemiDoc MP
Exposure Time (sec)	2.000 (Signal Accumulation)
Flat Field	Applied (Lens)

Serial Number	731BR01769
Software Version	5.0
Application	Chemi Hi Resolution
Excitation Source	No Illumination
Emission Filter	No Filter
Binning	2x2

Image Information

Acquisition Date	27/1/2023 6:04:38 PM
User Name	Histologia
Image Area (mm)	X: 114.0 Y: 85.2
Pixel Size (μm)	X: 163.8 Y: 163.8
Data Range (Int)	0 - 13276

Analysis Settings

Detection	Lane detection:<br>Manually created lanes<br><br>Band detection:<br>Automatically detected bands with sensitivity: Low<br><br>Lane Background Subtraction:<br>Lane background subtracted with disk size: 10<br><br>Lane width: Variable
Volume Analysis	Background subtraction method: Local<br>Quantity regression method: Linear

Lane Statistics

Lane No.	Adj. Total Band Vol. (Int)	Total Band Vol. (Int)	Adj. Total Lane Vol. (Int)	Total Lane Vol. (Int)	Bkgd. Vol. (Int)	Norm. Factor
1	4 279 014	4 419 630	5 142 150	6 641 514	1 499 364	N/A
2	4 490 997	4 641 672	5 187 973	6 559 091	1 371 118	N/A
3	3 298 836	3 401 954	3 942 454	5 059 080	1 116 626	N/A
4	3 589 249	3 706 749	4 233 478	5 369 891	1 136 413	N/A
5	3 329 616	3 428 496	3 955 920	5 086 272	1 130 352	N/A
6	3 766 275	3 867 660	4 450 860	5 511 915	1 061 055	N/A
7	3 232 250	3 336 700	4 005 100	5 213 650	1 208 550	N/A

Lane And Band Analysis

Lane 1

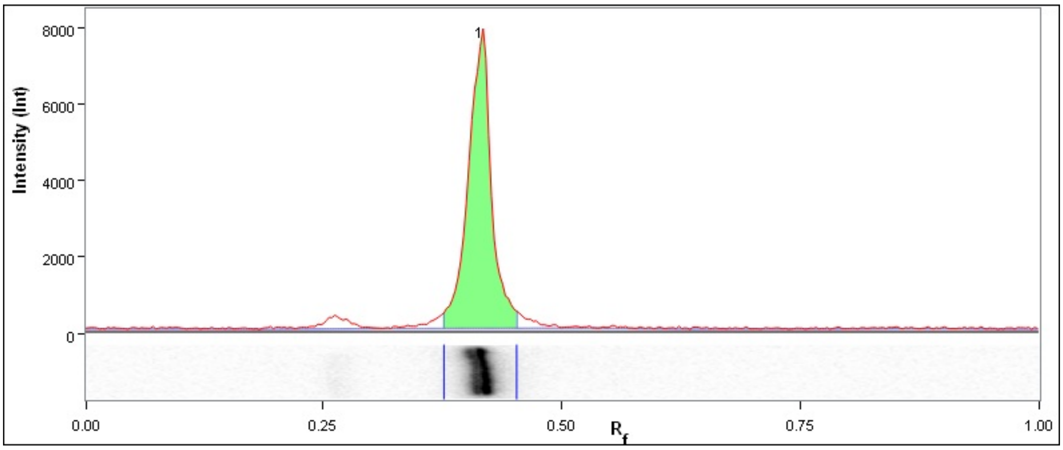

Band No.	Band Label	Mol. Wt. (KDa)	Relative Front	Adj. Volume (Int)	Volume (Int)	Abs. Quant.	Rel. Quant.	Band %	Lane %
1		N/A	0,416	4 279 014	4 419 630	N/A	N/A	100,0	83,2

Band Detection	Automatically detected bands with sensitivity: Low
Lane Background	Lane background subtracted with disk size: 10
Lane Width	8.84 mm

Lane 2

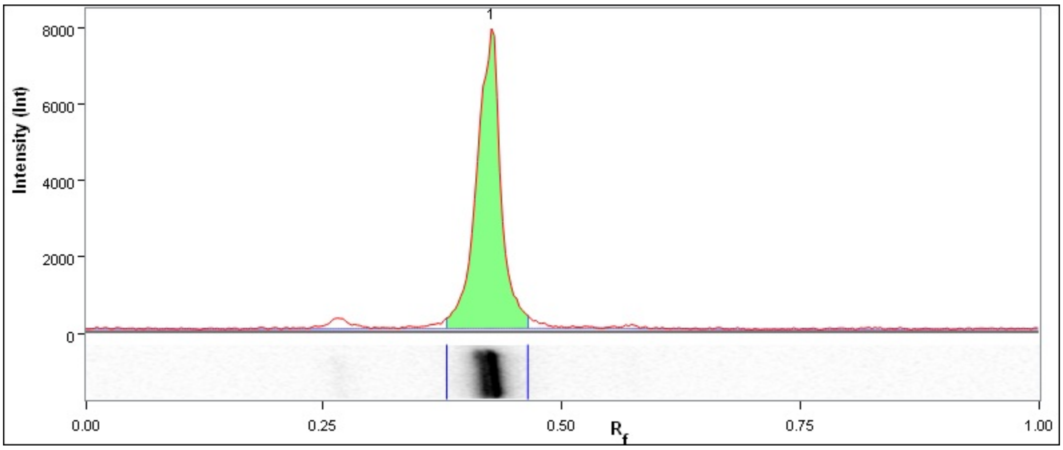

Band No.	Band Label	Mol. Wt. (KDa)	Relative Front	Adj. Volume (Int)	Volume (Int)	Abs. Quant.	Rel. Quant.	Band %	Lane %
1		N/A	0,428	4 490 997	4 641 672	N/A	N/A	100,0	86,6

Band Detection	Automatically detected bands with sensitivity: Low
Lane Background	Lane background subtracted with disk size: 10
Lane Width	8.03 mm

Lane 3

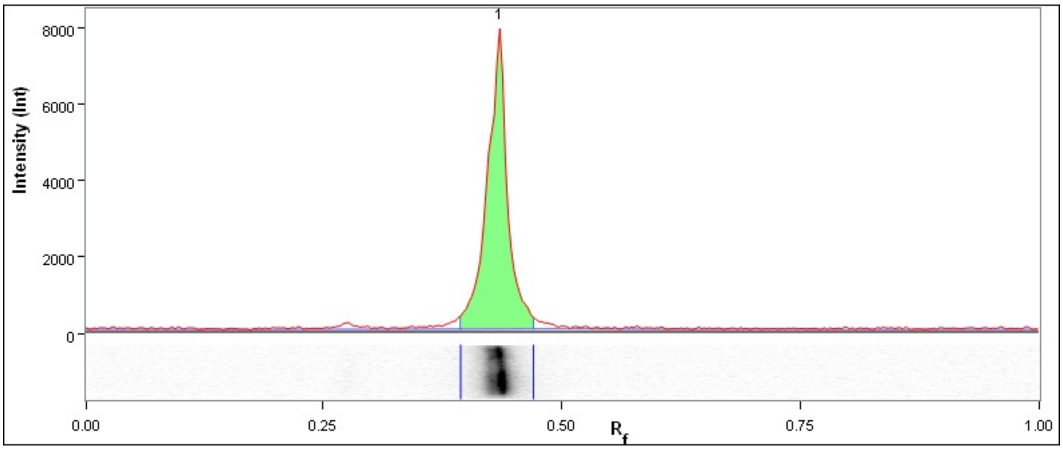

Band No.	Band Label	Mol. Wt. (KDa)	Relative Front	Adj. Volume (Int)	Volume (Int)	Abs. Quant.	Rel. Quant.	Band %	Lane %
1		N/A	0,437	3 298 836	3 401 954	N/A	N/A	100,0	83,7

Band Detection	Automatically detected bands with sensitivity: Low
Lane Background	Lane background subtracted with disk size: 10
Lane Width	7.70 mm

Lane 4

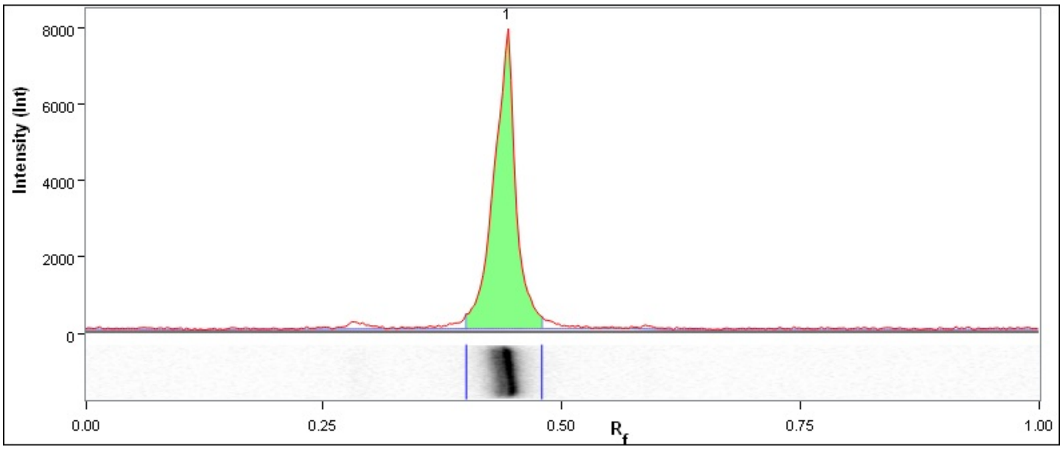

Band No.	Band Label	Mol. Wt. (KDa)	Relative Front	Adj. Volume (Int)	Volume (Int)	Abs. Quant.	Rel. Quant.	Band %	Lane %
1		N/A	0,446	3 589 249	3 706 749	N/A	N/A	100,0	84,8

Band Detection	Automatically detected bands with sensitivity: Low
Lane Background	Lane background subtracted with disk size: 10
Lane Width	7.70 mm

Lane 5

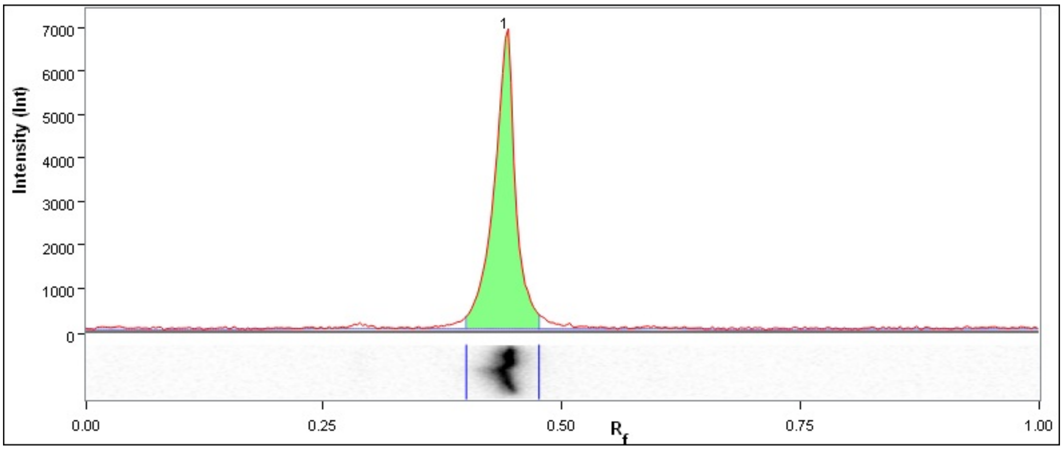

Band No.	Band Label	Mol. Wt. (KDa)	Relative Front	Adj. Volume (Int)	Volume (Int)	Abs. Quant.	Rel. Quant.	Band %	Lane %
1		N/A	0,443	3 329 616	3 428 496	N/A	N/A	100,0	84,2

Band Detection	Automatically detected bands with sensitivity: Low
Lane Background	Lane background subtracted with disk size: 10
Lane Width	7.86 mm

Lane 6

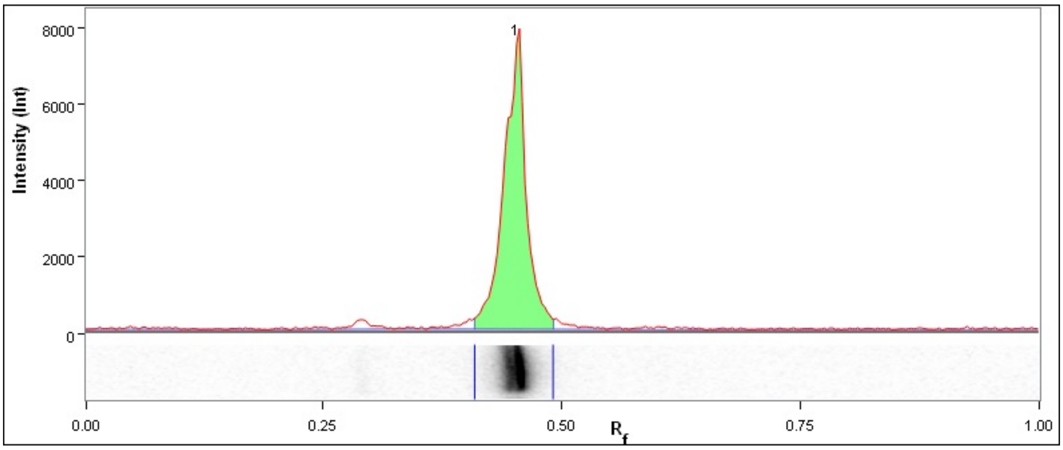

Band No.	Band Label	Mol. Wt. (KDa)	Relative Front	Adj. Volume (Int)	Volume (Int)	Abs. Quant.	Rel. Quant.	Band %	Lane %
1		N/A	0,455	3 766 275	3 867 660	N/A	N/A	100,0	84,6

Band Detection	Automatically detected bands with sensitivity: Low
Lane Background	Lane background subtracted with disk size: 10
Lane Width	7.37 mm

Lane 7

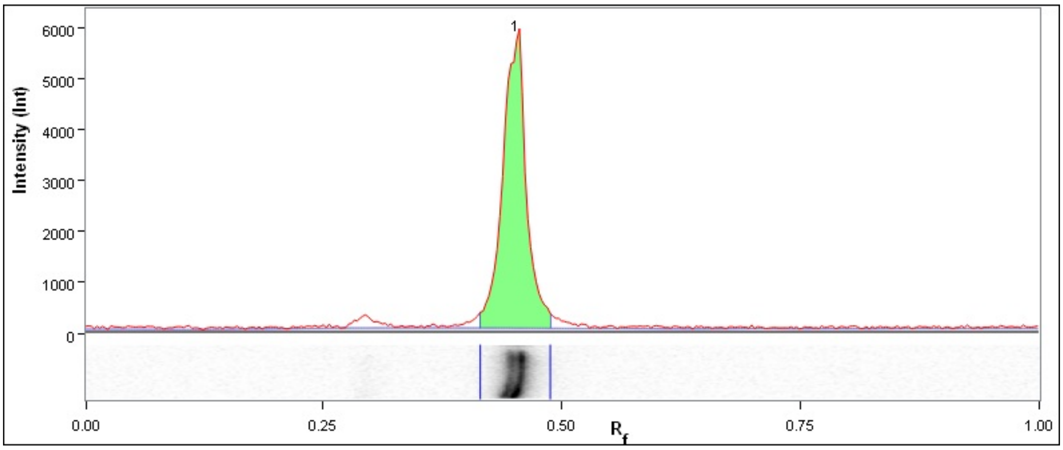

Band No.	Band Label	Mol. Wt. (KDa)	Relative Front	Adj. Volume (Int)	Volume (Int)	Abs. Quant.	Rel. Quant.	Band %	Lane %
1		N/A	0,455	3 232 250	3 336 700	N/A	N/A	100,0	80,7

Band Detection	Automatically detected bands with sensitivity: Low
Lane Background	Lane background subtracted with disk size: 10
Lane Width	8.19 mm

Volume Analysis

No.	Label	Type	Volume (Int)	Adj. Vol. (Int)	Mean Bkgd. (Int)	Abs. Quant.	Rel. Quant.	# of Pixels	Min. Value (Int)	Max. Value (Int)	Mean Value (Int)	Std. Dev.	Area (mm2)
1	U1	Unknown	4 710 024	3 700 924	415,3	N/A	N/A	2 430	0	11 940	1 938,3	2 620,6	65,2
2	U2	Unknown	5 032 520	3 731 628	535,3	N/A	N/A	2 430	0	12 756	2 071,0	2 848,4	65,2
3	U3	Unknown	3 890 908	2 400 813	613,2	N/A	N/A	2 430	0	12 024	1 601,2	2 251,2	65,2
4	U4	Unknown	4 109 864	2 959 584	473,4	N/A	N/A	2 430	0	11 440	1 691,3	2 348,1	65,2
5	U5	Unknown	3 799 088	2 731 283	439,4	N/A	N/A	2 430	0	12 548	1 563,4	2 318,6	65,2
6	U6	Unknown	4 500 180	3 195 246	537,0	N/A	N/A	2 430	0	13 276	1 851,9	2 590,7	65,2
7	U7	Unknown	3 543 228	2 868 073	277,8	N/A	N/A	2 430	0	10 640	1 458,1	2 128,6	65,2

Image Report: Histologia 2023-01-26 12hr 04min_Exposure_3.0sec 3b

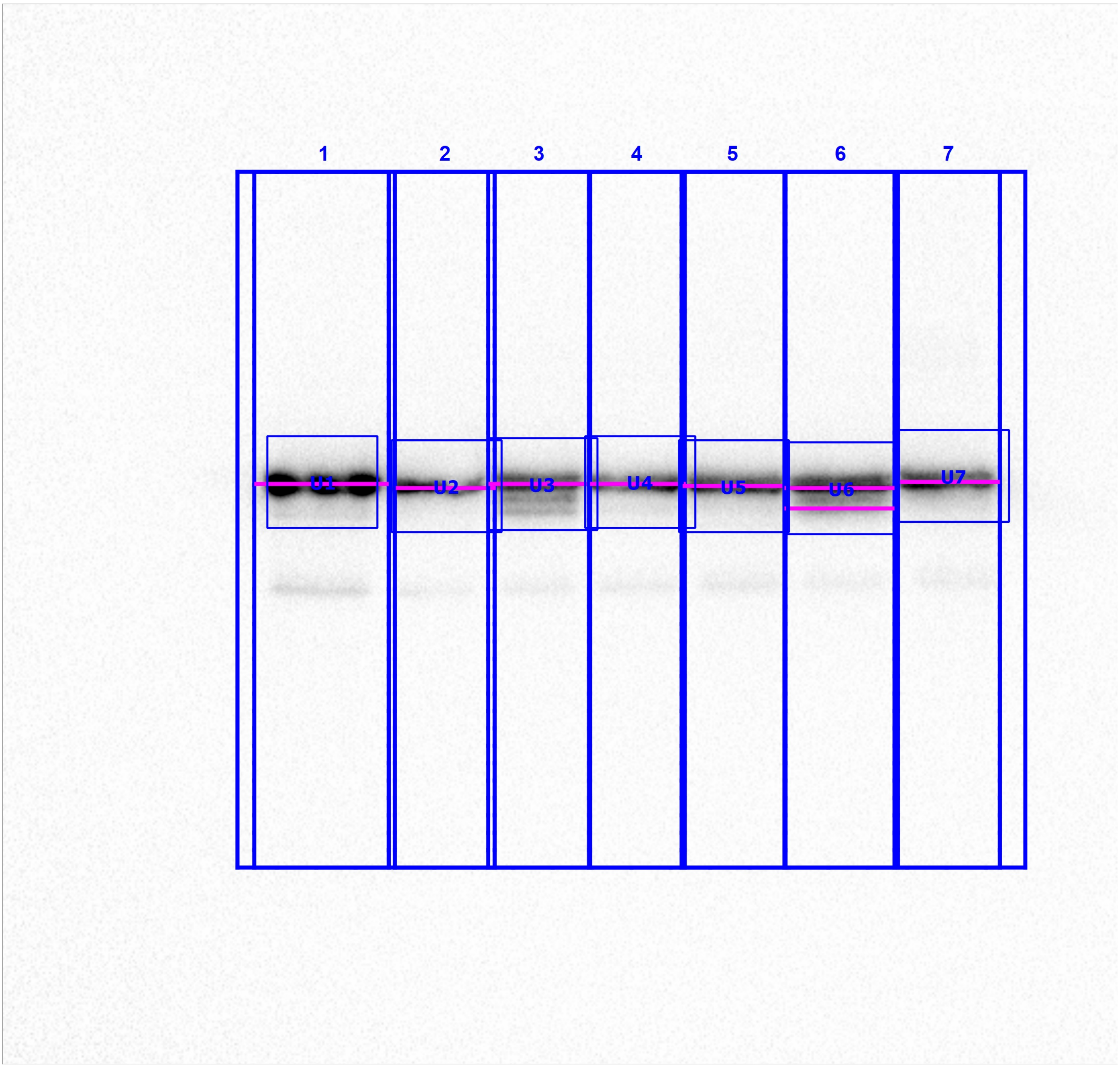

C:\Users\rusak\OneDrive\Dokumenty\Badania\CHI3L2 in BC\BC westerny ilościowo\ERK 26.1.23\3\Histologia 2023-01-26 12hr 04min_Exposure_3.0sec 3b.scn

Acquisition Information

Imager	ChemiDoc MP
Exposure Time (sec)	3.000 (Signal Accumulation)
Flat Field	Applied (Lens)
Serial Number	731BR01769

Software Version	5.0
Application	Chemi Hi Resolution
Excitation Source	No Illumination
Emission Filter	No Filter
Binning	2x2

Image Information

Acquisition Date	26/1/2023 12:05:08 PM
User Name	Histologia
Image Area (mm)	X: 114.0 Y: 85.2
Pixel Size (μm)	X: 163.8 Y: 163.8
Data Range (Int)	0 - 30348

Analysis Settings

Detection	Lane detection:<br>Manually created lanes<br><br>Band detection:<br>Automatically detected bands with sensitivity: Low<br><br>Lane Background Subtraction:<br>Lane background subtracted with disk size: 10<br><br>Lane width: Variable
Volume Analysis	Background subtraction method: Local<br>Quantity regression method: Linear

Lane Statistics

Lane No.	Adj. Total Band Vol. (Int)	Total Band Vol. (Int)	Adj. Total Lane Vol. (Int)	Total Lane Vol. (Int)	Bkgd. Vol. (Int)	Norm. Factor
1	14 013 978	14 695 098	16 186 632	20 655 492	4 468 860	N/A
2	5 165 923	5 459 286	6 741 861	9 638 496	2 896 635	N/A
3	7 610 850	8 044 350	9 071 000	11 876 700	2 805 700	N/A
4	5 335 722	5 664 487	6 707 558	9 330 393	2 622 835	N/A
5	6 597 972	7 018 977	8 243 589	11 388 351	3 144 762	N/A
6	9 606 762	10 085 256	11 050 938	14 093 190	3 042 252	N/A
7	6 698 700	7 100 900	8 411 050	11 570 500	3 159 450	N/A

Lane And Band Analysis

Lane 1

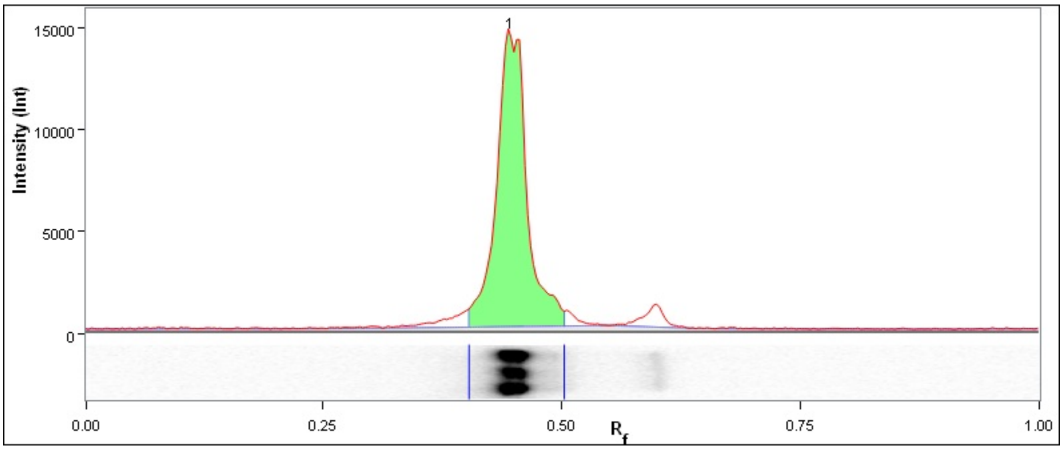

Band No.	Band Label	Mol. Wt. (KDa)	Relative Front	Adj. Volume (Int)	Volume (Int)	Abs. Quant.	Rel. Quant.	Band %	Lane %
1		N/A	0,449	14 013 978	14 695 098	N/A	N/A	100,0	86,6

Band Detection	Automatically detected bands with sensitivity: Low
Lane Background	Lane background subtracted with disk size: 10
Lane Width	10.81 mm

Lane 2

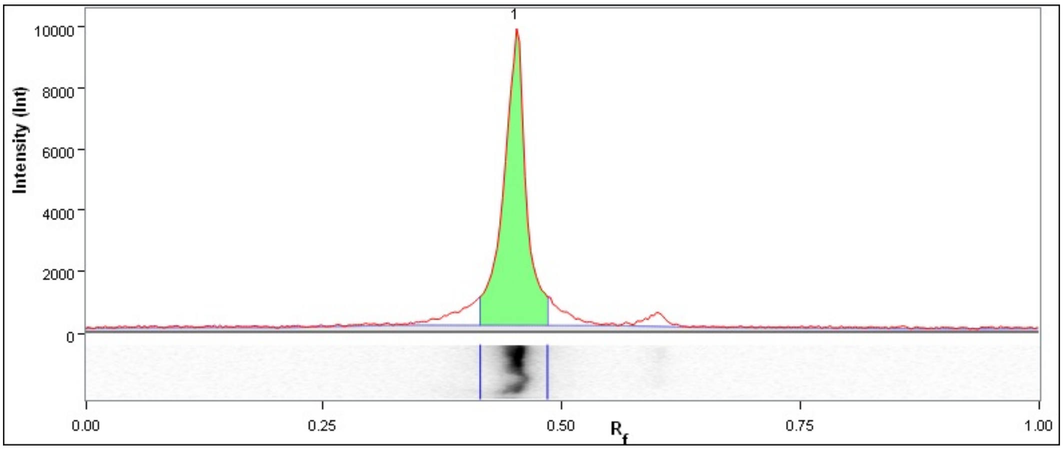

Band No.	Band Label	Mol. Wt. (KDa)	Relative Front	Adj. Volume (Int)	Volume (Int)	Abs. Quant.	Rel. Quant.	Band %	Lane %
1		N/A	0,455	5 165 923	5 459 286	N/A	N/A	100,0	76,6

Band Detection	Automatically detected bands with sensitivity: Low
Lane Background	Lane background subtracted with disk size: 10
Lane Width	8.03 mm

Lane 3

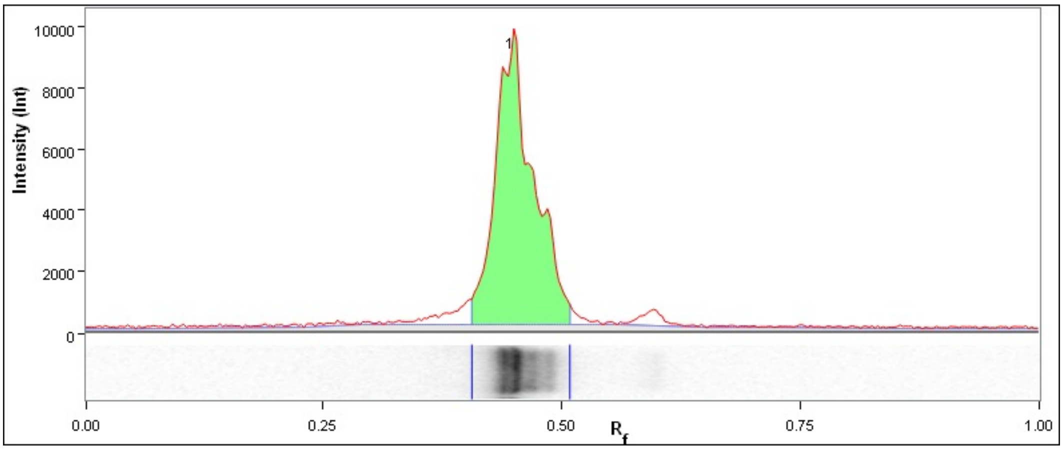

Band No.	Band Label	Mol. Wt. (KDa)	Relative Front	Adj. Volume (Int)	Volume (Int)	Abs. Quant.	Rel. Quant.	Band %	Lane %
1		N/A	0,449	7 610 850	8 044 350	N/A	N/A	100,0	83,9

Band Detection	Automatically detected bands with sensitivity: Low
Lane Background	Lane background subtracted with disk size: 10
Lane Width	8.19 mm

Lane 4

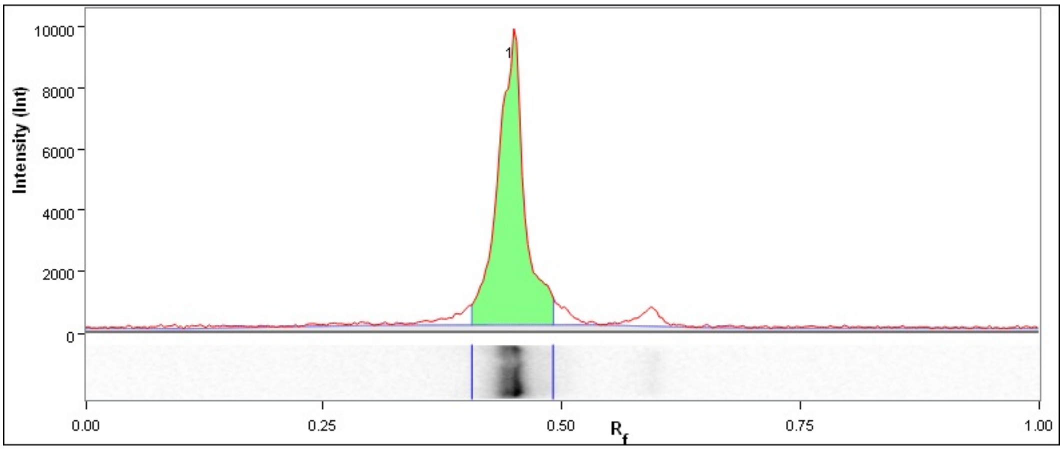

Band No.	Band Label	Mol. Wt. (KDa)	Relative Front	Adj. Volume (Int)	Volume (Int)	Abs. Quant.	Rel. Quant.	Band %	Lane %
1		N/A	0,449	5 335 722	5 664 487	N/A	N/A	100,0	79,5

Band Detection	Automatically detected bands with sensitivity: Low
Lane Background	Lane background subtracted with disk size: 10
Lane Width	7.70 mm

Lane 5

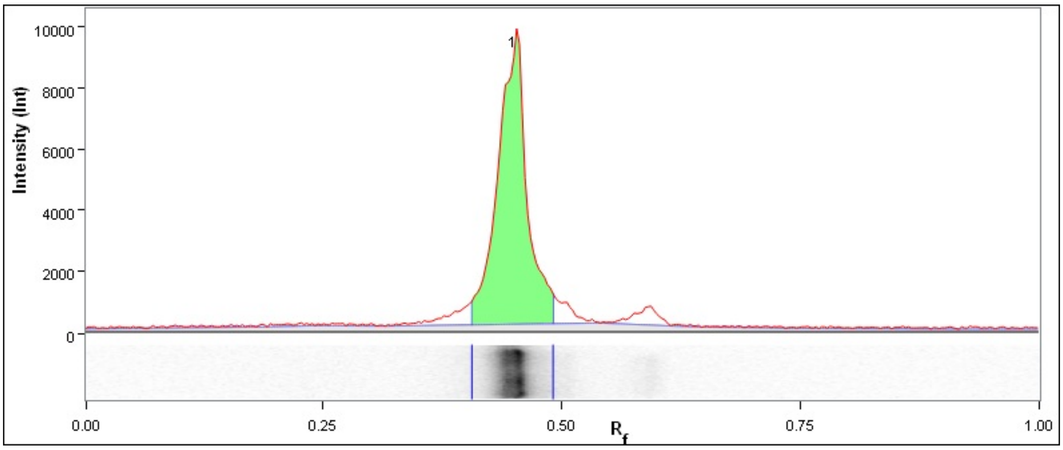

Band No.	Band Label	Mol. Wt. (KDa)	Relative Front	Adj. Volume (Int)	Volume (Int)	Abs. Quant.	Rel. Quant.	Band %	Lane %
1		N/A	0,452	6 597 972	7 018 977	N/A	N/A	100,0	80,0

Band Detection	Automatically detected bands with sensitivity: Low
Lane Background	Lane background subtracted with disk size: 10
Lane Width	8.35 mm

Lane 6

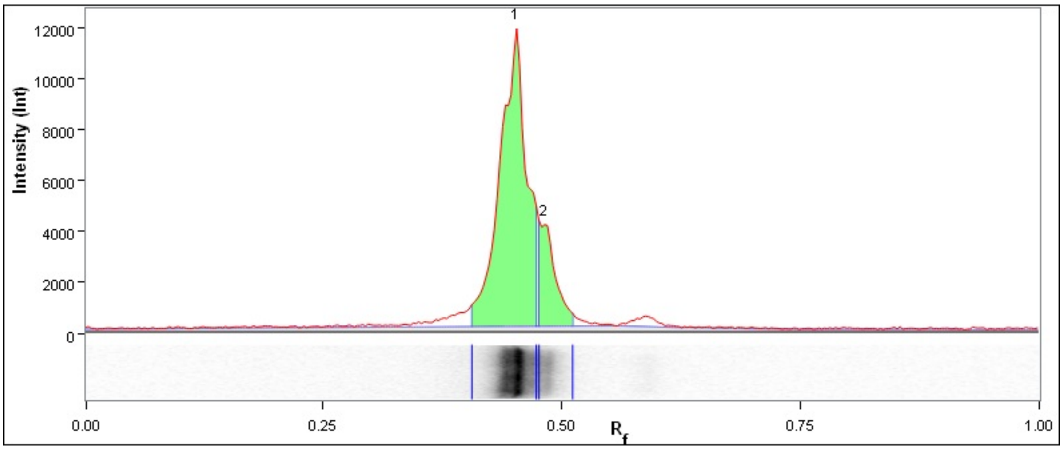

Band No.	Band Label	Mol. Wt. (KDa)	Relative Front	Adj. Volume (Int)	Volume (Int)	Abs. Quant.	Rel. Quant.	Band %	Lane %
1		N/A	0,455	7 855 542	8 159 832	N/A	N/A	81,8	71,1
2		N/A	0,484	1 751 220	1 925 424	N/A	N/A	18,2	15,8

Band Detection	Automatically detected bands with sensitivity: Low
Lane Background	Lane background subtracted with disk size: 10
Lane Width	8.84 mm

Lane 7

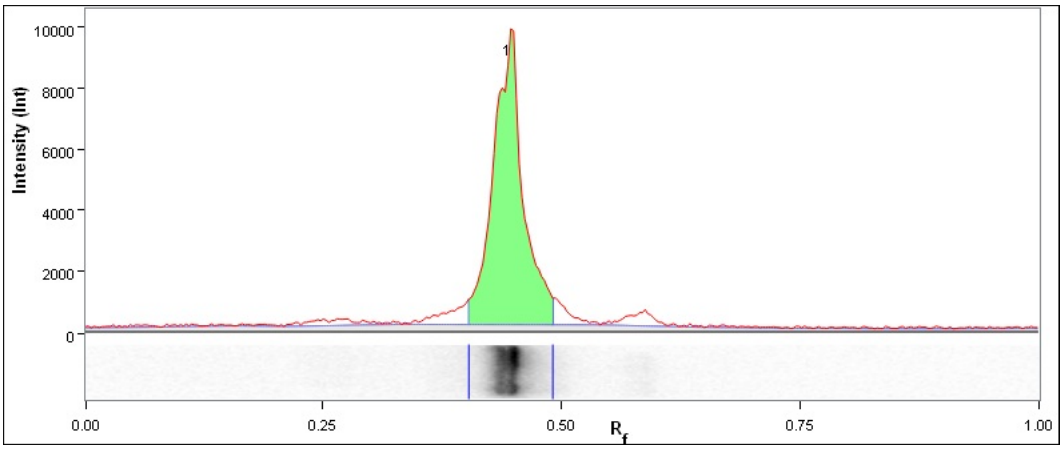

Band No.	Band Label	Mol. Wt. (KDa)	Relative Front	Adj. Volume (Int)	Volume (Int)	Abs. Quant.	Rel. Quant.	Band %	Lane %
1		N/A	0,446	6 698 700	7 100 900	N/A	N/A	100,0	79,6

Band Detection	Automatically detected bands with sensitivity: Low
Lane Background	Lane background subtracted with disk size: 10
Lane Width	8.19 mm

Volume Analysis

No.	Label	Type	Volume (Int)	Adj. Vol. (Int)	Mean Bkgd. (Int)	Abs. Quant.	Rel. Quant.	# of Pixels	Min. Value (Int)	Max. Value (Int)	Mean Value (Int)	Std. Dev.	Area (mm2)

1	U1	Unknown	14 167 400	9 340 698	1 986,3	N/A	N/A	2 430	0	30 348	5 830,2	6 730,9	65,2
2	U2	Unknown	7 029 692	2 557 963	1 840,2	N/A	N/A	2 430	0	17 984	2 892,9	3 296,4	65,2
3	U3	Unknown	8 884 092	5 606 142	1 349,0	N/A	N/A	2 430	0	12 784	3 656,0	3 098,8	65,2
4	U4	Unknown	7 001 628	3 311 493	1 518,6	N/A	N/A	2 430	0	15 204	2 881,3	3 002,5	65,2
5	U5	Unknown	8 027 108	4 131 505	1 603,1	N/A	N/A	2 430	0	14 280	3 303,3	3 193,7	65,2
6	U6	Unknown	10 427 272	7 020 075	1 402,1	N/A	N/A	2 430	0	15 892	4 291,1	3 775,3	65,2
7	U7	Unknown	7 812 684	5 135 450	1 101,7	N/A	N/A	2 430	0	15 640	3 215,1	3 280,2	65,2

Image Report: Histologia 2023-01-27 18hr 02min_Exposure_3.0sec 1c ERK

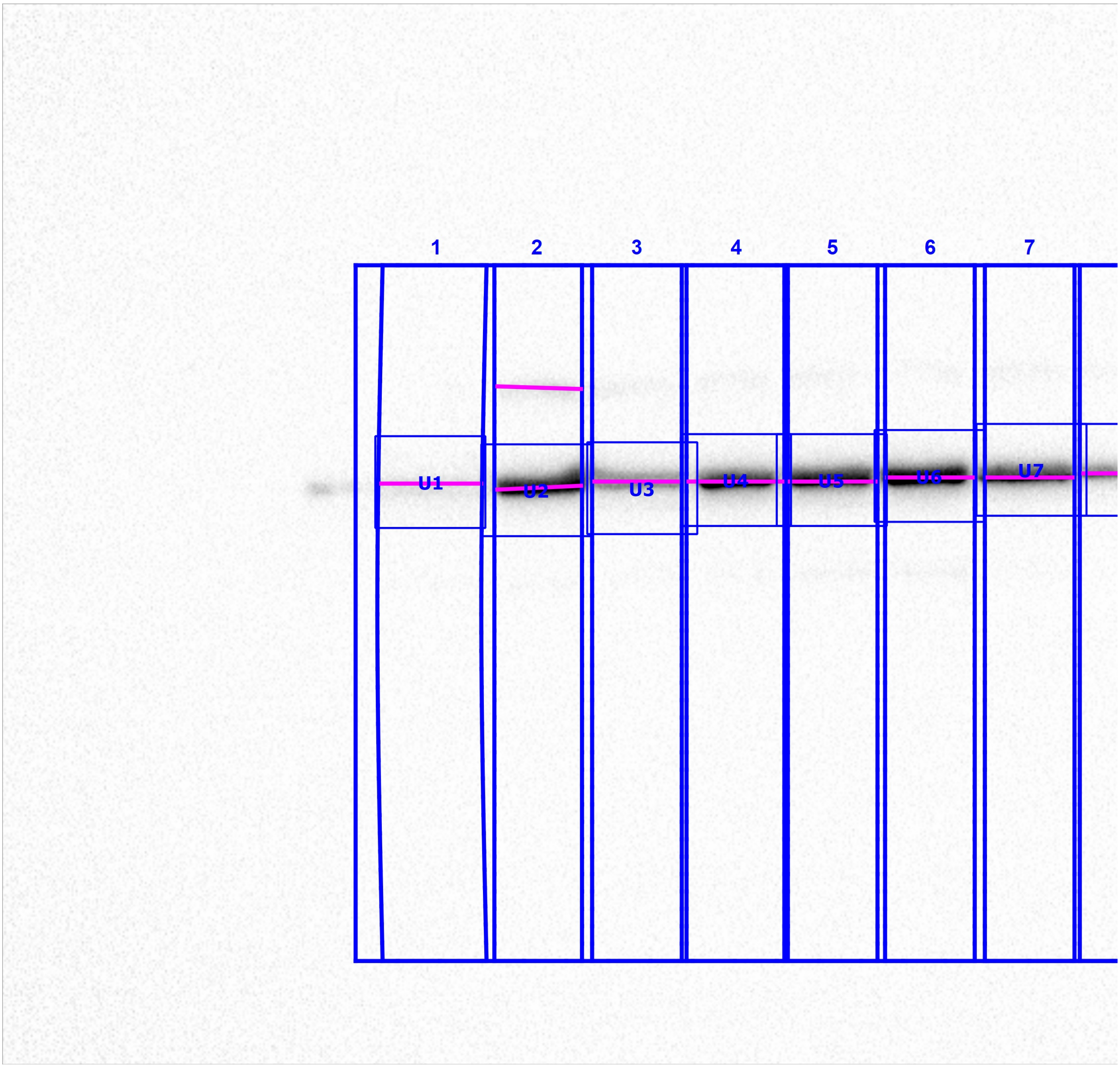

C:\Users\rusak\OneDrive\Dokumenty\Badania\CHI3L2 in BC\BC westerny ilościowo\ERK 27.1.23\1\Histologia 2023-01-27 18hr 02min_Exposure_3.0sec 1c ERK.scn

Acquisition Information

Imager	ChemiDoc MP
Exposure Time (sec)	3.000 (Signal Accumulation)
Flat Field	Applied (Lens)

Serial Number	731BR01769
Software Version	5.0
Application	Chemi Hi Resolution
Excitation Source	No Illumination
Emission Filter	No Filter
Binning	2x2

Image Information

Acquisition Date	27/1/2023 6:03:09 PM
User Name	Histologia
Image Area (mm)	X: 114.0 Y: 85.2
Pixel Size (µm)	X: 163.8 Y: 163.8
Data Range (Int)	0 - 16904

Analysis Settings

Detection	Lane detection:<br>Manually created lanes<br><br>Band detection:<br>Automatically detected bands with sensitivity: Low<br><br>Lane Background Subtraction:<br>Lane background subtracted with disk size: 10<br><br>Lane width: Variable
Volume Analysis	Background subtraction method: Local<br>Quantity regression method: Linear

Lane Statistics

Lane No.	Adj. Total Band Vol. (Int)	Total Band Vol. (Int)	Adj. Total Lane Vol. (Int)	Total Lane Vol. (Int)	Bkgd. Vol. (Int)	Norm. Factor
1	718 284	799 629	1 655 562	3 345 753	1 690 191	N/A
2	5 543 173	5 790 122	6 193 978	7 760 855	1 566 877	N/A
3	2 484 724	2 607 308	3 399 000	4 795 780	1 396 780	N/A
4	4 713 408	4 882 656	5 607 024	7 115 424	1 508 400	N/A
5	5 128 112	5 289 680	6 007 144	7 380 516	1 373 372	N/A
6	5 713 488	5 864 716	6 610 164	7 979 444	1 369 280	N/A
7	4 476 164	4 623 300	5 258 880	6 625 300	1 366 420	N/A
8	2 505 579	2 600 694	3 327 036	4 840 512	1 513 476	N/A

Lane And Band Analysis

Lane 1

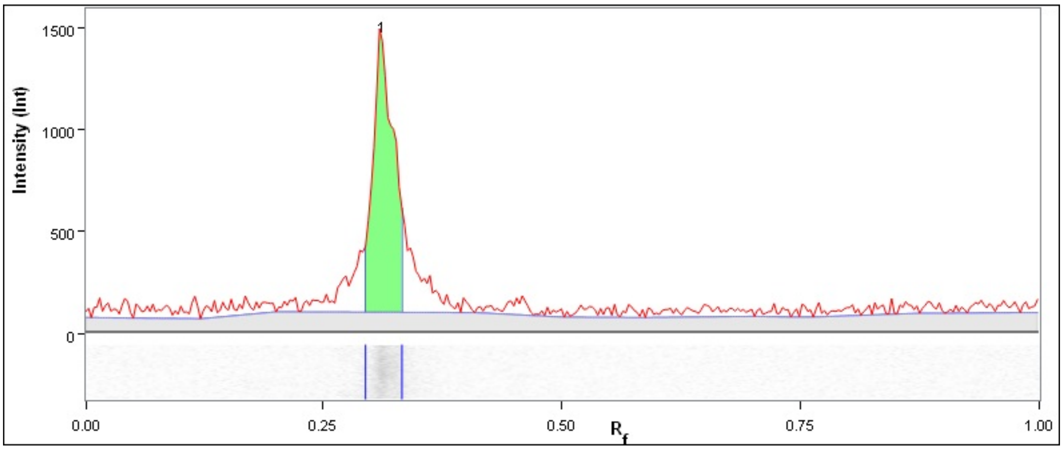

Band No.	Band Label	Mol. Wt. (KDa)	Relative Front	Adj. Volume (Int)	Volume (Int)	Abs. Quant.	Rel. Quant.	Band %	Lane %
1		N/A	0,314	718 284	799 629	N/A	N/A	100,0	43,4

Band Detection	Automatically detected bands with sensitivity: Low
Lane Background	Lane background subtracted with disk size: 10
Lane Width	8.35 mm

Lane 2

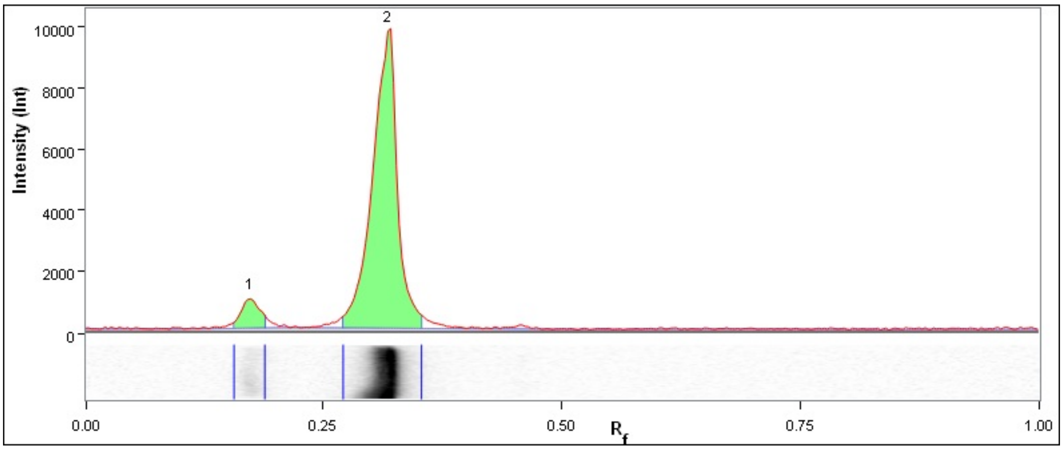

Band No.	Band Label	Mol. Wt. (KDa)	Relative Front	Adj. Volume (Int)	Volume (Int)	Abs. Quant.	Rel. Quant.	Band %	Lane %
1		N/A	0,176	384 506	456 832	N/A	N/A	6,9	6,2
2		N/A	0,320	5 158 667	5 333 290	N/A	N/A	93,1	83,3

Band Detection	Automatically detected bands with sensitivity: Low
Lane Background	Lane background subtracted with disk size: 10
Lane Width	7.04 mm

Lane 3

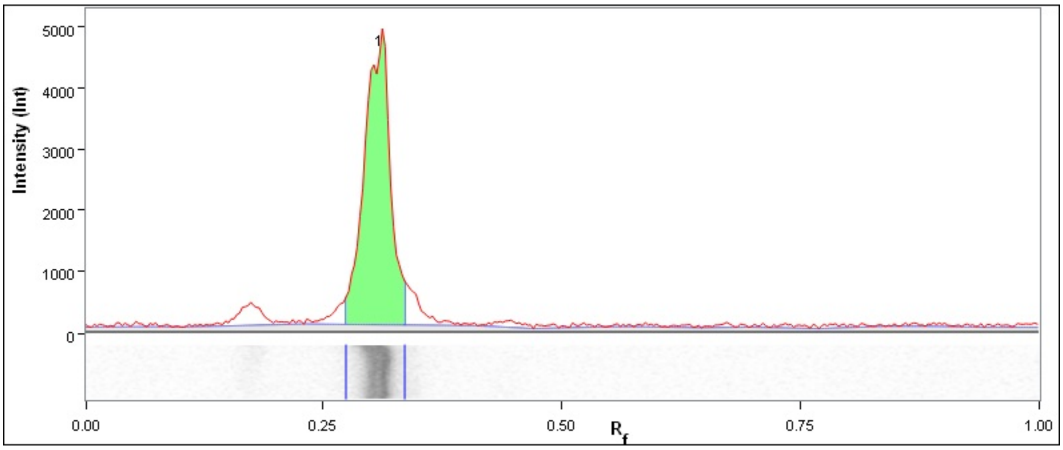

Band No.	Band Label	Mol. Wt. (KDa)	Relative Front	Adj. Volume (Int)	Volume (Int)	Abs. Quant.	Rel. Quant.	Band %	Lane %
1		N/A	0,311	2 484 724	2 607 308	N/A	N/A	100,0	73,1

Band Detection	Automatically detected bands with sensitivity: Low
Lane Background	Lane background subtracted with disk size: 10
Lane Width	7.21 mm

Lane 4

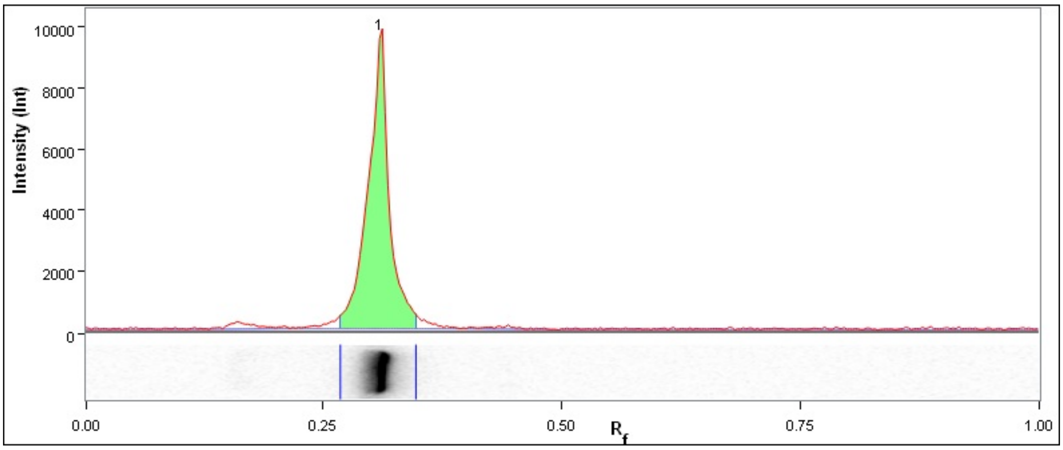

Band No.	Band Label	Mol. Wt. (KDa)	Relative Front	Adj. Volume (Int)	Volume (Int)	Abs. Quant.	Rel. Quant.	Band %	Lane %
1		N/A	0,311	4 713 408	4 882 656	N/A	N/A	100,0	84,1

Band Detection	Automatically detected bands with sensitivity: Low
Lane Background	Lane background subtracted with disk size: 10
Lane Width	7.86 mm

Lane 5

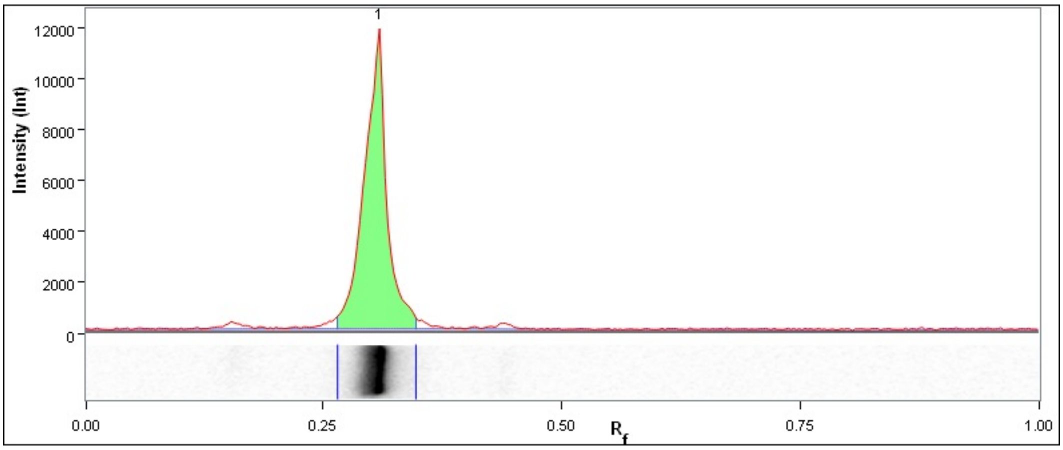

Band No.	Band Label	Mol. Wt. (KDa)	Relative Front	Adj. Volume (Int)	Volume (Int)	Abs. Quant.	Rel. Quant.	Band %	Lane %
1		N/A	0,311	5 128 112	5 289 680	N/A	N/A	100,0	85,4

Band Detection	Automatically detected bands with sensitivity: Low
Lane Background	Lane background subtracted with disk size: 10
Lane Width	7.21 mm

Lane 6

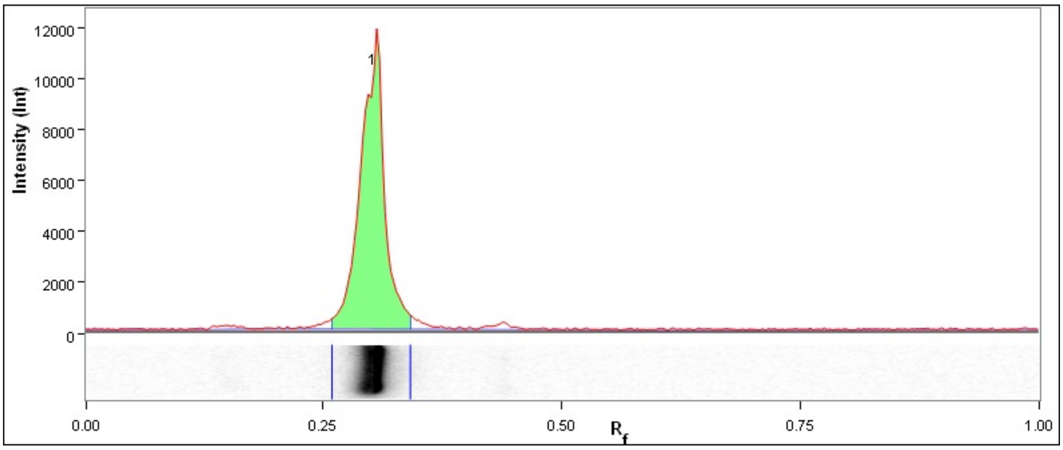

Band No.	Band Label	Mol. Wt. (KDa)	Relative Front	Adj. Volume (Int)	Volume (Int)	Abs. Quant.	Rel. Quant.	Band %	Lane %
1		N/A	0,305	5 713 488	5 864 716	N/A	N/A	100,0	86,4

Band Detection	Automatically detected bands with sensitivity: Low
Lane Background	Lane background subtracted with disk size: 10
Lane Width	7.21 mm

Lane 7

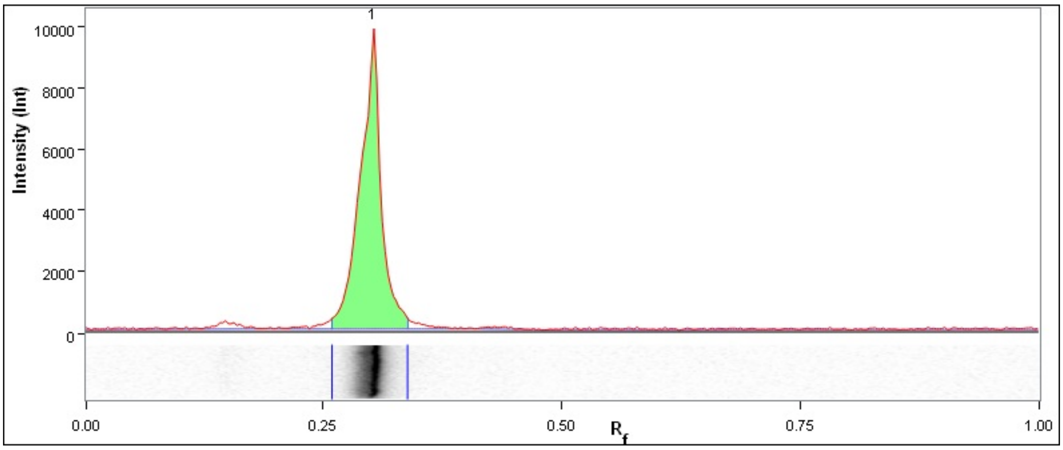

Band No.	Band Label	Mol. Wt. (KDa)	Relative Front	Adj. Volume (Int)	Volume (Int)	Abs. Quant.	Rel. Quant.	Band %	Lane %
1		N/A	0,305	4 476 164	4 623 300	N/A	N/A	100,0	85,1

Band Detection	Automatically detected bands with sensitivity: Low
Lane Background	Lane background subtracted with disk size: 10
Lane Width	7.21 mm

Lane 8

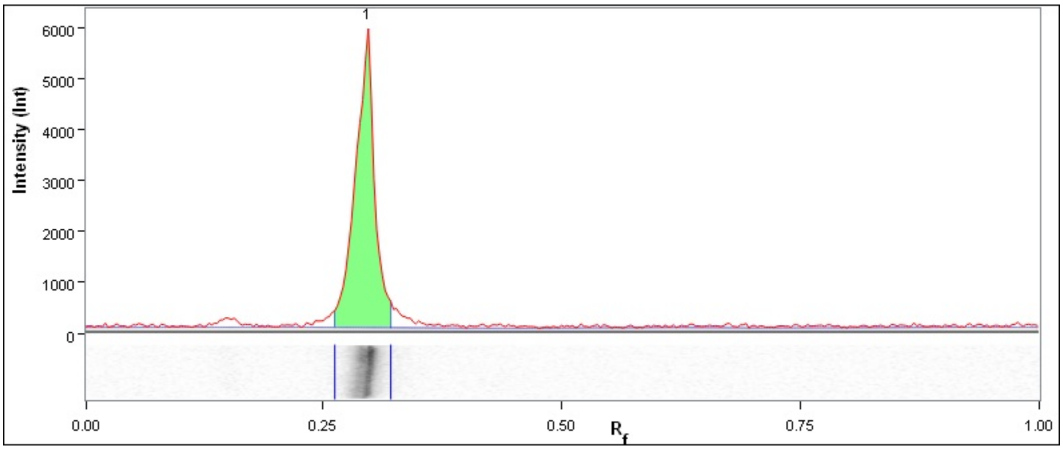

Band No.	Band Label	Mol. Wt. (KDa)	Relative Front	Adj. Volume (Int)	Volume (Int)	Abs. Quant.	Rel. Quant.	Band %	Lane %
1		N/A	0,299	2 505 579	2 600 694	N/A	N/A	100,0	75,3

Band Detection	Automatically detected bands with sensitivity: Low
Lane Background	Lane background subtracted with disk size: 10
Lane Width	8.35 mm

Volume Analysis

No.	Label	Type	Volume (Int)	Adj. Vol. (Int)	Mean Bkgd. (Int)	Abs. Quant.	Rel. Quant.	# of Pixels	Min. Value (Int)	Max. Value (Int)	Mean Value (Int)	Std. Dev.	Area (mm2)
1	U1	Unknown	1 355 256	686 982	275,0	N/A	N/A	2 430	0	2 676	557,7	512,7	65,2
2	U2	Unknown	6 230 140	4 678 790	638,4	N/A	N/A	2 430	0	14 604	2 563,8	3 461,1	65,2
3	U3	Unknown	3 500 740	1 364 553	879,1	N/A	N/A	2 430	0	6 772	1 440,6	1 607,4	65,2

4	U4	Unknown	5 553 604	3 155 074	987,0	N/A	N/A	2 430	0	16 848	2 285,4	3 249,7	65,2
5	U5	Unknown	6 250 780	3 612 329	1 085,8	N/A	N/A	2 430	0	15 528	2 572,3	3 389,6	65,2
6	U6	Unknown	6 875 676	4 620 155	928,2	N/A	N/A	2 430	0	16 904	2 829,5	3 782,2	65,2
7	U7	Unknown	5 470 448	3 763 914	702,3	N/A	N/A	2 430	0	13 376	2 251,2	3 033,0	65,2
8	U8	Unknown	2 941 328	2 075 863	356,2	N/A	N/A	2 430	0	8 984	1 210,4	1 694,3	65,2

Image Report: Histologia 2023-01-24 13hr 39min_Exposure_3.0sec 3a

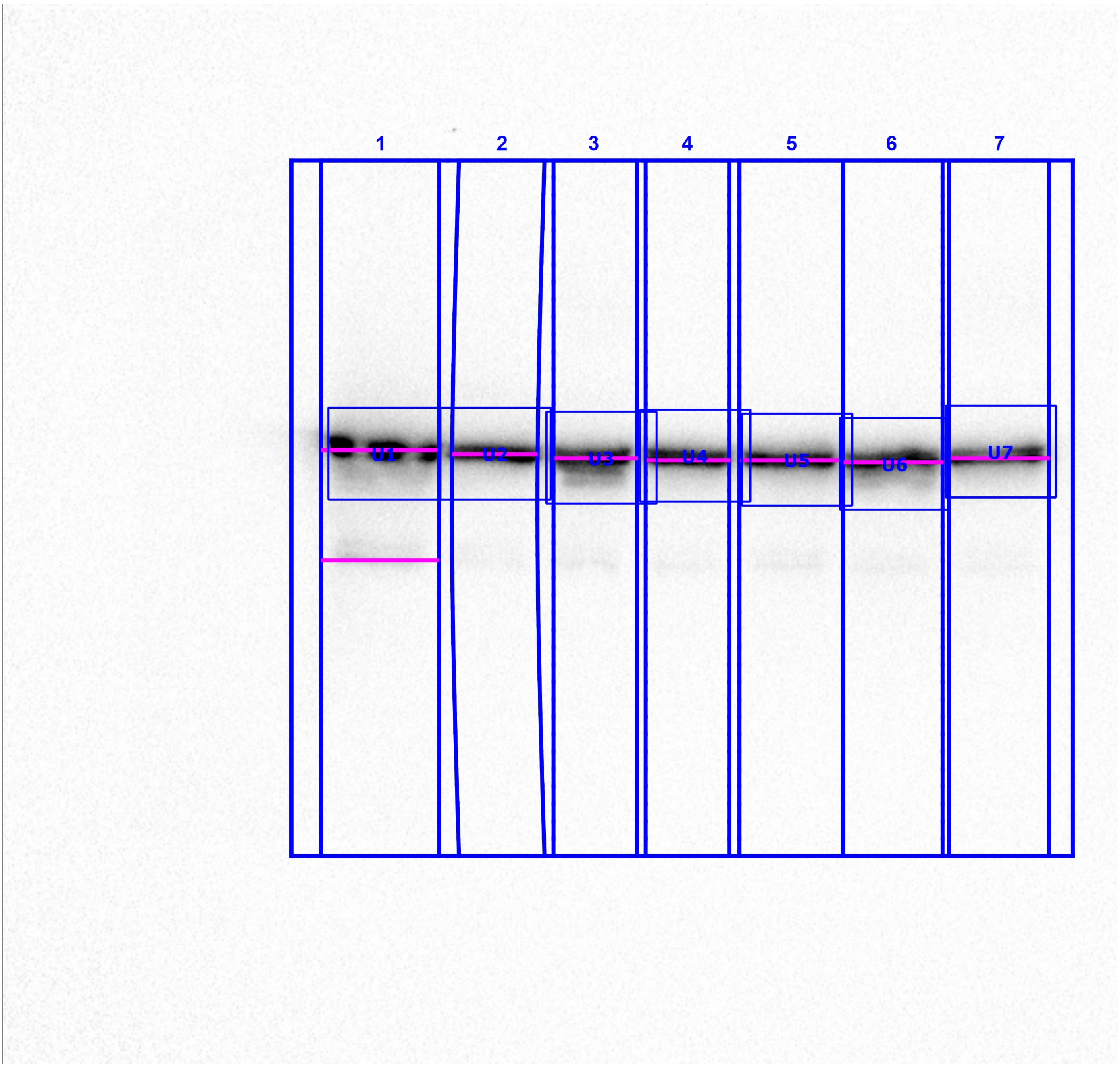

C:\Users\rusak\OneDrive\Dokumenty\Badania\CHI3L2 in BC\BC westerny ilościowo\ERK 24.1.23 BC\3\Histologia 2023-01-24 13hr 39min_Exposure_3.0sec 3a.scn

Acquisition Information

Imager	ChemiDoc MP
Exposure Time (sec)	3.000 (Signal Accumulation)
Flat Field	Applied (Lens)
Serial Number	731BR01769

Software Version	5.0
Application	Chemi Hi Resolution
Excitation Source	No Illumination
Emission Filter	No Filter
Binning	2x2

Image Information

Acquisition Date	24/1/2023 1:39:56 PM
User Name	Histologia
Image Area (mm)	X: 114.0 Y: 85.2
Pixel Size (µm)	X: 163.8 Y: 163.8
Data Range (Int)	0 - 30836

Analysis Settings

Detection	Lane detection:<br>Manually created lanes<br><br>Band detection:<br>Automatically detected bands with sensitivity: Low<br><br>Lane Background Subtraction:<br>Lane background subtracted with disk size: 10<br><br>Lane width: Variable
Volume Analysis	Background subtraction method: Local<br>Quantity regression method: Linear

Lane Statistics

Lane No.	Adj. Total Band Vol. (Int)	Total Band Vol. (Int)	Adj. Total Lane Vol. (Int)	Total Lane Vol. (Int)	Bkgd. Vol. (Int)	Norm. Factor
1	14 774 514	17 390 720	16 490 792	23 046 996	6 556 204	N/A
2	7 091 742	7 484 526	8 695 302	11 550 378	2 855 076	N/A
3	8 201 804	8 748 990	9 442 628	12 417 957	2 975 329	N/A
4	7 149 703	7 480 327	8 247 109	10 501 043	2 253 934	N/A
5	8 221 149	8 690 349	9 619 008	12 751 836	3 132 828	N/A
6	9 263 940	9 837 926	10 380 258	13 656 055	3 275 797	N/A
7	6 925 366	7 315 357	8 399 580	11 176 900	2 777 320	N/A

Lane And Band Analysis

Lane 1

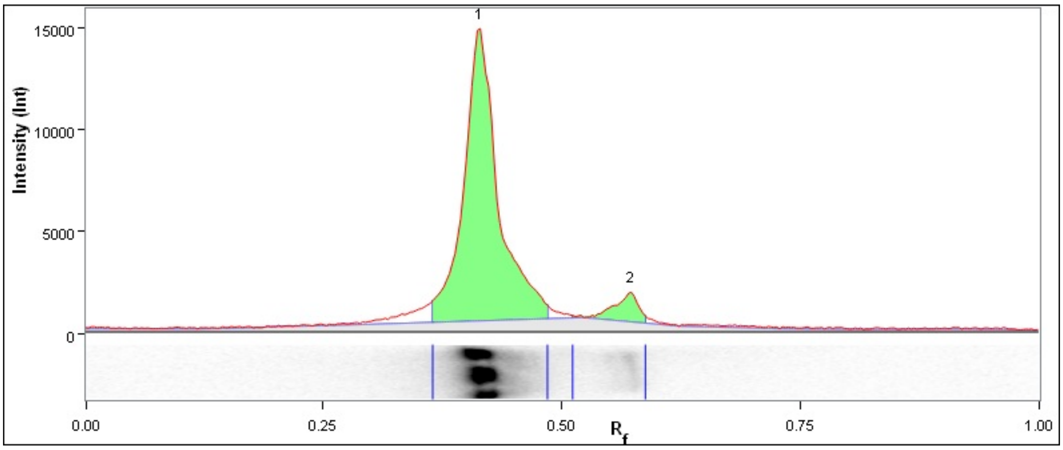

Band No.	Band Label	Mol. Wt. (KDa)	Relative Front	Adj. Volume (Int)	Volume (Int)	Abs. Quant.	Rel. Quant.	Band %	Lane %
1		N/A	0,416	13 797 214	15 360 430	N/A	N/A	93,4	83,7
2		N/A	0,575	977 300	2 030 290	N/A	N/A	6,6	5,9

Band Detection	Automatically detected bands with sensitivity: Low
Lane Background	Lane background subtracted with disk size: 10
Lane Width	9.50 mm

Lane 2

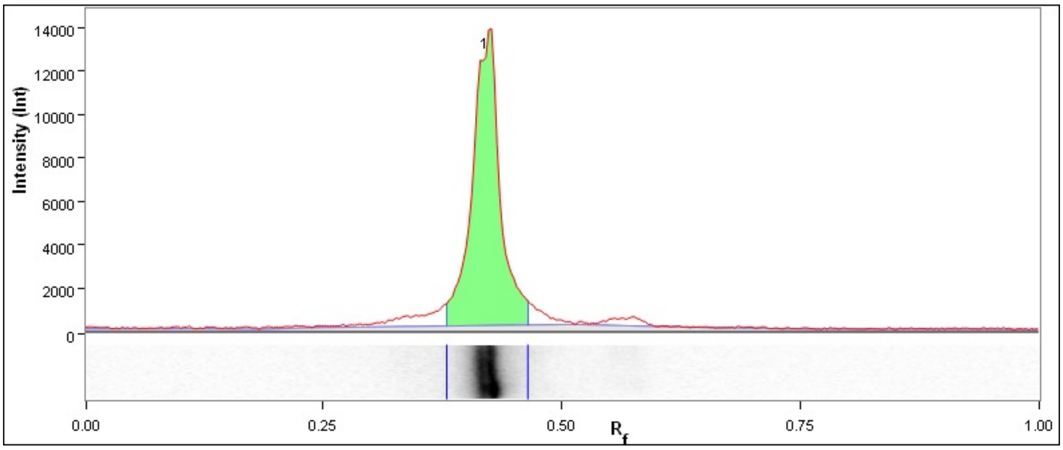

Band No.	Band Label	Mol. Wt. (KDa)	Relative Front	Adj. Volume (Int)	Volume (Int)	Abs. Quant.	Rel. Quant.	Band %	Lane %
1		N/A	0,422	7 091 742	7 484 526	N/A	N/A	100,0	81,6

Band Detection	Automatically detected bands with sensitivity: Low
Lane Background	Lane background subtracted with disk size: 10
Lane Width	6.88 mm

Lane 3

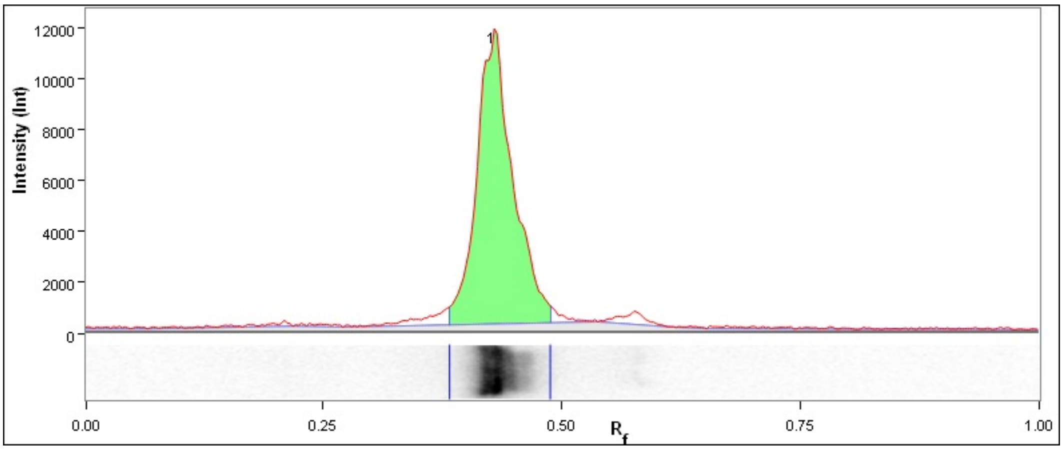

Band No.	Band Label	Mol. Wt. (KDa)	Relative Front	Adj. Volume (Int)	Volume (Int)	Abs. Quant.	Rel. Quant.	Band %	Lane %

1		N/A	0,428	8 201 804	8 748 990	N/A	N/A	100,0	86,9

Band Detection	Automatically detected bands with sensitivity: Low
Lane Background	Lane background subtracted with disk size: 10
Lane Width	6.72 mm

Lane 4

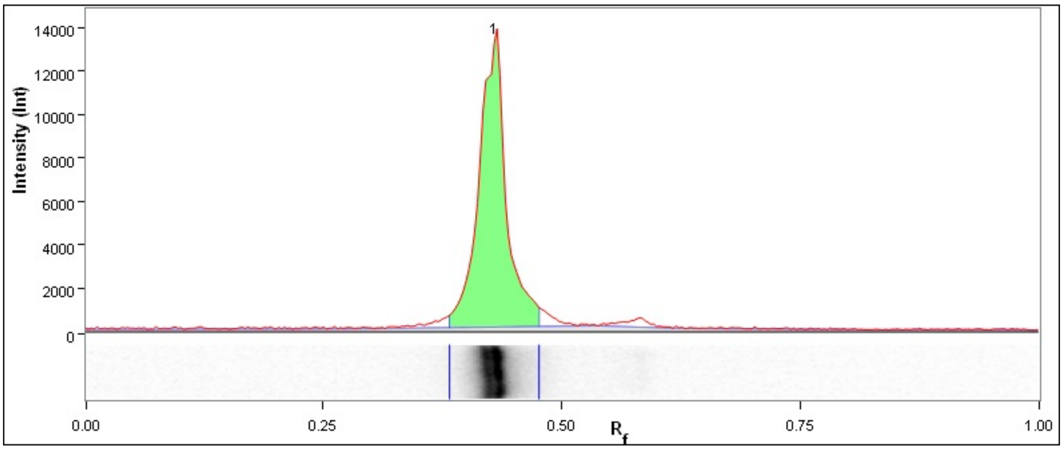

Band No.	Band Label	Mol. Wt. (KDa)	Relative Front	Adj. Volume (Int)	Volume (Int)	Abs. Quant.	Rel. Quant.	Band %	Lane %
1		N/A	0,431	7 149 703	7 480 327	N/A	N/A	100,0	86,7

Band Detection	Automatically detected bands with sensitivity: Low
Lane Background	Lane background subtracted with disk size: 10
Lane Width	6.72 mm

Lane 5

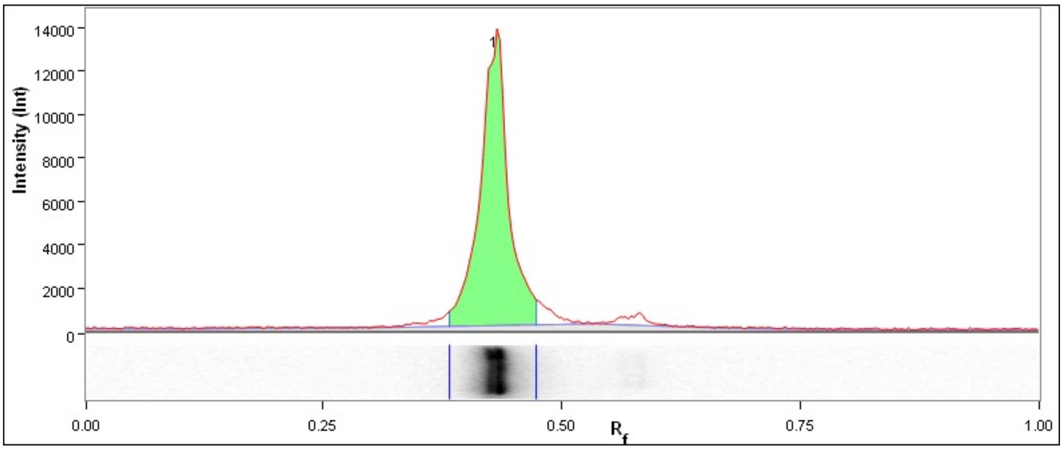

Band No.	Band Label	Mol. Wt. (KDa)	Relative Front	Adj. Volume (Int)	Volume (Int)	Abs. Quant.	Rel. Quant.	Band %	Lane %
1		N/A	0,431	8 221 149	8 690 349	N/A	N/A	100,0	85,5

Band Detection	Automatically detected bands with sensitivity: Low
Lane Background	Lane background subtracted with disk size: 10
Lane Width	8.35 mm

Lane 6

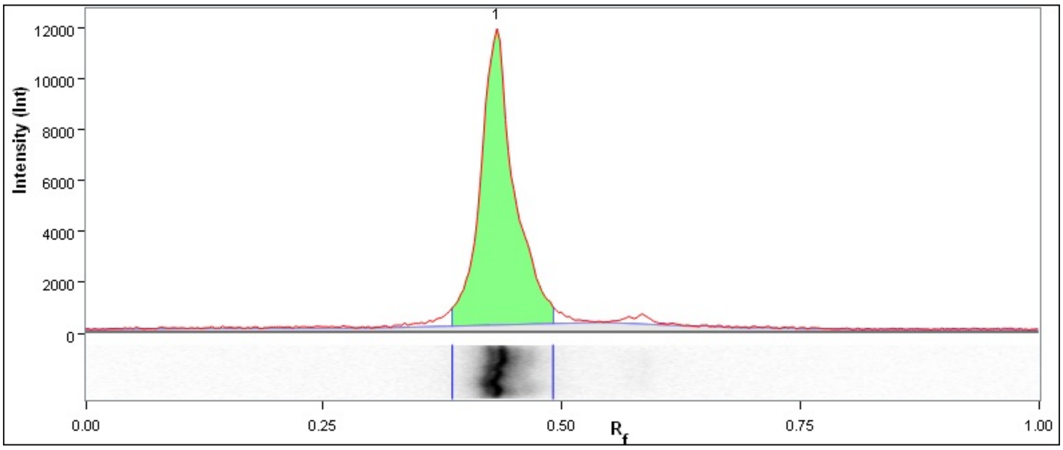

Band No.	Band Label	Mol. Wt. (KDa)	Relative Front	Adj. Volume (Int)	Volume (Int)	Abs. Quant.	Rel. Quant.	Band %	Lane %
1		N/A	0,434	9 263 940	9 837 926	N/A	N/A	100,0	89,2

Band Detection	Automatically detected bands with sensitivity: Low
Lane Background	Lane background subtracted with disk size: 10
Lane Width	8.03 mm

Lane 7

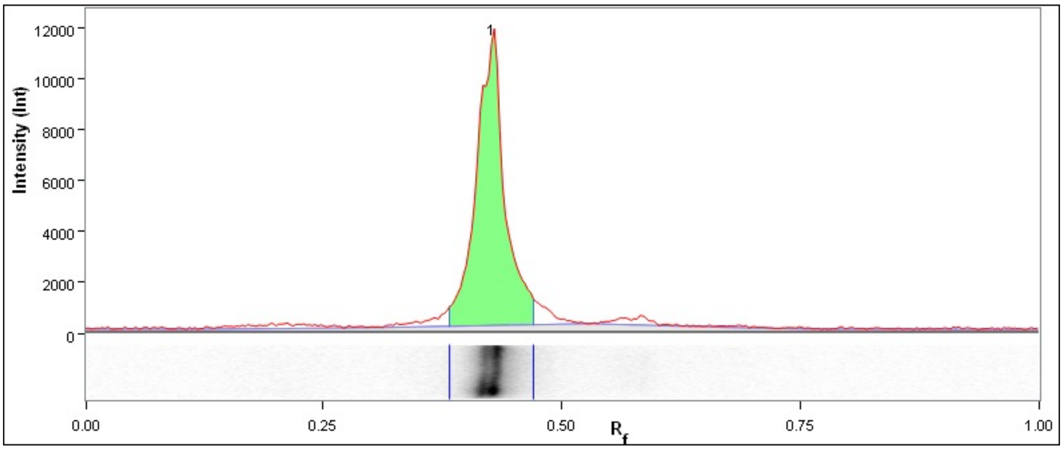

Band No.	Band Label	Mol. Wt. (KDa)	Relative Front	Adj. Volume (Int)	Volume (Int)	Abs. Quant.	Rel. Quant.	Band %	Lane %
1		N/A	0,428	6 925 366	7 315 357	N/A	N/A	100,0	82,4

Band Detection	Automatically detected bands with sensitivity: Low
Lane Background	Lane background subtracted with disk size: 10
Lane Width	8.03 mm

Volume Analysis

No.	Label	Type	Volume (Int)	Adj. Vol. (Int)	Mean Bkgd. (Int)	Abs. Quant.	Rel. Quant.	# of Pixels	Min. Value (Int)	Max. Value (Int)	Mean Value (Int)	Std. Dev.	Area (mm2)

1	U1	Unknown	15 150 660	9 646 734	2 265,0	N/A	N/A	2 430	556	30 836	6 234,8	5 875,6	65,2
2	U2	Unknown	9 622 076	5 020 233	1 893,8	N/A	N/A	2 430	0	18 564	3 959,7	4 196,1	65,2
3	U3	Unknown	10 708 936	6 150 256	1 876,0	N/A	N/A	2 430	0	17 700	4 407,0	4 252,6	65,2
4	U4	Unknown	9 248 560	5 455 402	1 561,0	N/A	N/A	2 430	0	18 756	3 806,0	4 345,3	65,2
5	U5	Unknown	9 922 728	5 479 870	1 828,3	N/A	N/A	2 430	0	18 124	4 083,4	4 214,3	65,2
6	U6	Unknown	10 721 060	6 739 806	1 638,4	N/A	N/A	2 430	0	17 356	4 412,0	4 137,4	65,2
7	U7	Unknown	8 120 716	5 649 574	1 016,9	N/A	N/A	2 430	0	17 796	3 341,9	3 600,6	65,2

Image Report: Histologia 2023-01-26 12hr 03min_Exposure_1.0sec 2b ERK

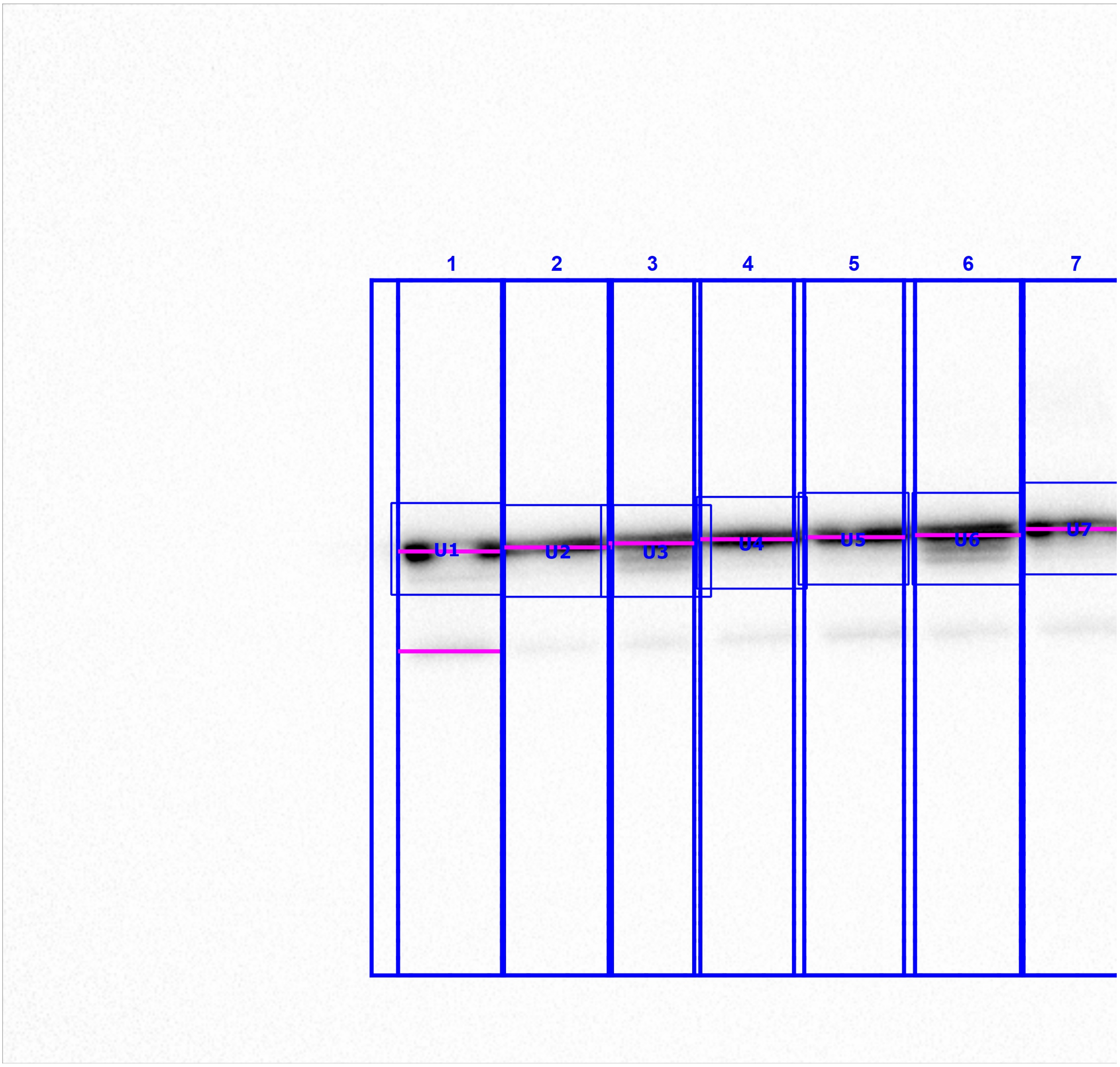

C:\Users\rusak\OneDrive\Dokumenty\Badania\CHI3L2 in BC\BC westerny ilościowo\ERK 26.1.23\2\Histologia 2023-01-26 12hr 03min_Exposure_1.0sec 2b ERK.scn

Acquisition Information

Imager	ChemiDoc MP
Exposure Time (sec)	3.000 (Signal Accumulation)
Flat Field	Applied (Lens)

Serial Number	731BR01769
Software Version	5.0
Application	Chemi Hi Resolution
Excitation Source	No Illumination
Emission Filter	No Filter
Binning	2x2

Image Information

Acquisition Date	26/1/2023 12:03:50 PM
User Name	Histologia
Image Area (mm)	X: 114.0 Y: 85.2
Pixel Size (µm)	X: 163.8 Y: 163.8
Data Range (Int)	0 - 33868

Analysis Settings

Detection	Lane detection:<br>Manually created lanes<br><br>Band detection:<br>Automatically detected bands with sensitivity: Low<br><br>Lane Background Subtraction:<br>Lane background subtracted with disk size: 10<br><br>Lane width: Variable
Volume Analysis	Background subtraction method: Local<br>Quantity regression method: Linear

Lane Statistics

Lane No.	Adj. Total Band Vol. (Int)	Total Band Vol. (Int)	Adj. Total Lane Vol. (Int)	Total Lane Vol. (Int)	Bkgd. Vol. (Int)	Norm. Factor
1	11 471 668	12 298 988	12 686 908	15 933 944	3 247 036	N/A
2	9 309 924	9 638 784	10 694 052	13 232 484	2 538 432	N/A
3	9 565 752	9 905 490	10 808 112	12 757 416	1 949 304	N/A
4	9 998 928	10 298 434	11 332 284	13 335 170	2 002 886	N/A
5	10 418 674	10 790 437	12 131 371	14 482 195	2 350 824	N/A
6	14 869 296	15 344 368	16 459 664	19 006 416	2 546 752	N/A
7	10 334 636	10 708 932	12 471 472	15 093 728	2 622 256	N/A

Lane And Band Analysis

Lane 1

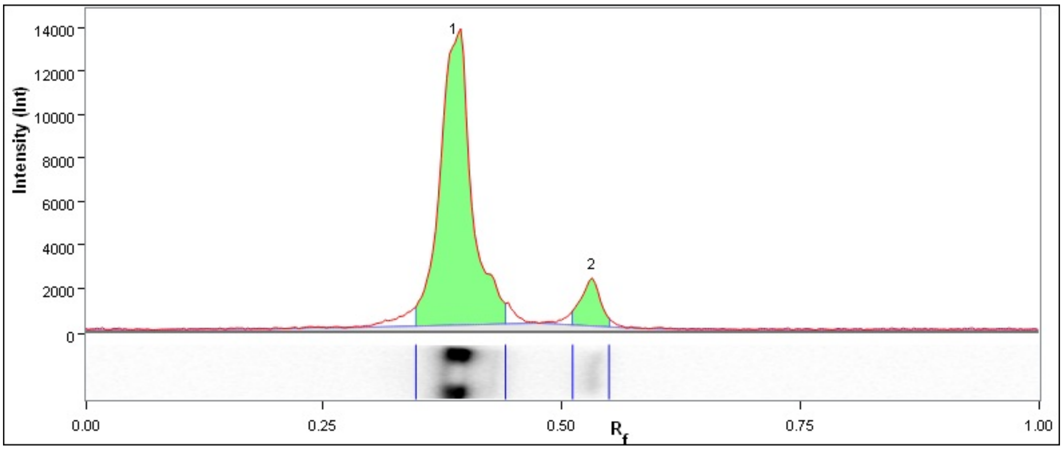

Band No.	Band Label	Mol. Wt. (KDa)	Relative Front	Adj. Volume (Int)	Volume (Int)	Abs. Quant.	Rel. Quant.	Band %	Lane %
1		N/A	0,390	10 433 072	11 037 832	N/A	N/A	90,9	82,2
2		N/A	0,534	1 038 596	1 261 156	N/A	N/A	9,1	8,2

Band Detection	Automatically detected bands with sensitivity: Low
Lane Background	Lane background subtracted with disk size: 10
Lane Width	8.52 mm

Lane 2

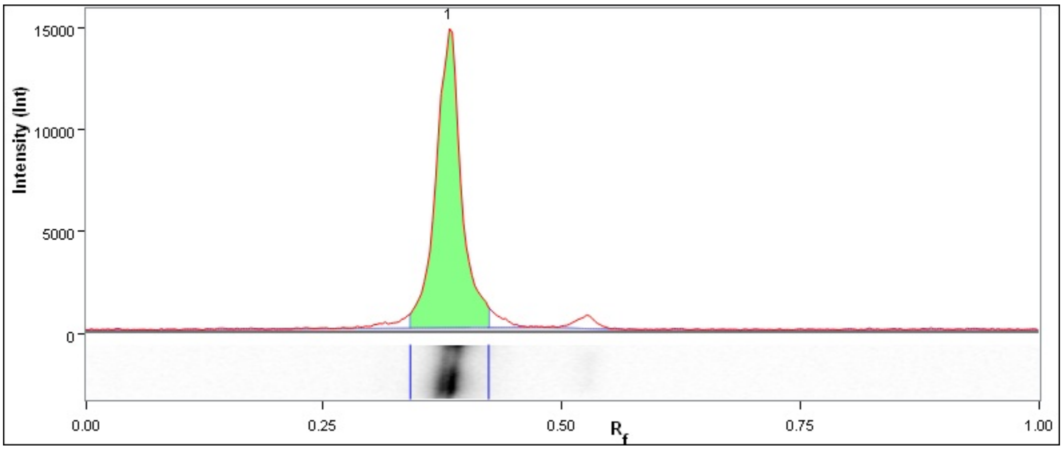

Band No.	Band Label	Mol. Wt. (KDa)	Relative Front	Adj. Volume (Int)	Volume (Int)	Abs. Quant.	Rel. Quant.	Band %	Lane %
1		N/A	0,384	9 309 924	9 638 784	N/A	N/A	100,0	87,1

Band Detection	Automatically detected bands with sensitivity: Low
Lane Background	Lane background subtracted with disk size: 10
Lane Width	8.84 mm

Lane 3

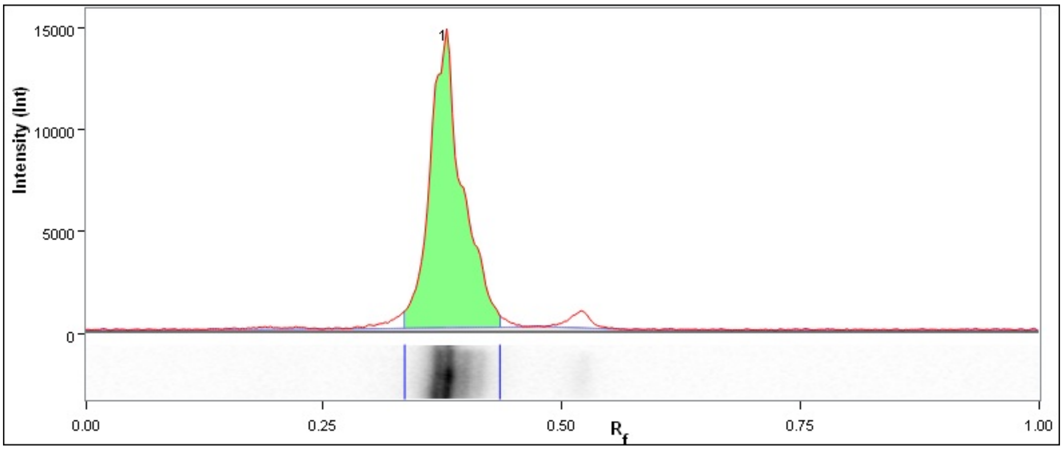

Band No.	Band Label	Mol. Wt. (KDa)	Relative Front	Adj. Volume (Int)	Volume (Int)	Abs. Quant.	Rel. Quant.	Band %	Lane %
1		N/A	0,378	9 565 752	9 905 490	N/A	N/A	100,0	88,5

Band Detection	Automatically detected bands with sensitivity: Low
Lane Background	Lane background subtracted with disk size: 10
Lane Width	6.88 mm

Lane 4

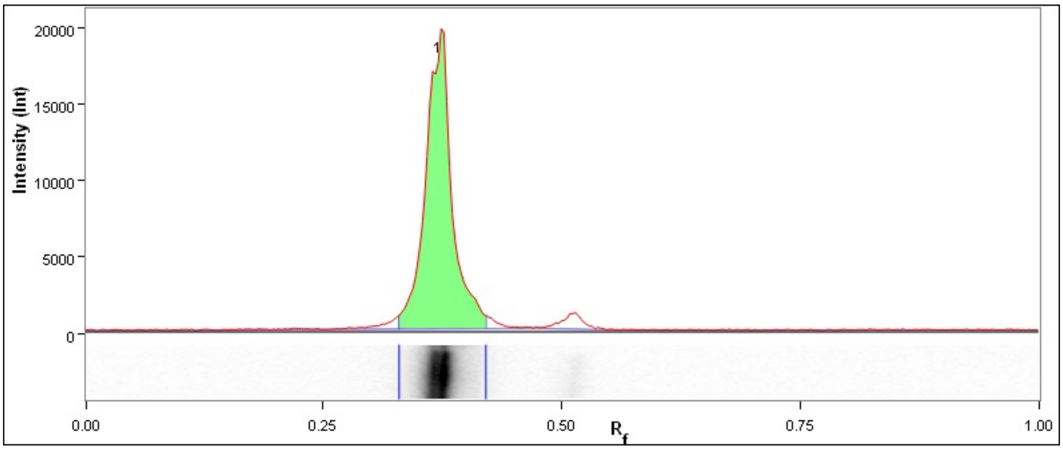

Band No.	Band Label	Mol. Wt. (KDa)	Relative Front	Adj. Volume (Int)	Volume (Int)	Abs. Quant.	Rel. Quant.	Band %	Lane %
1		N/A	0,372	9 998 928	10 298 434	N/A	N/A	100,0	88,2

Band Detection	Automatically detected bands with sensitivity: Low
Lane Background	Lane background subtracted with disk size: 10
Lane Width	7.53 mm

Lane 5

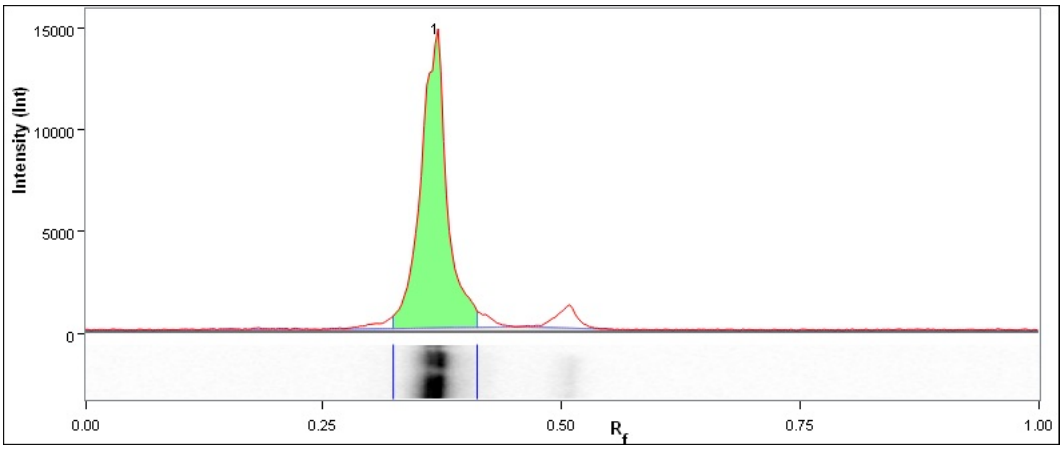

Band No.	Band Label	Mol. Wt. (KDa)	Relative Front	Adj. Volume (Int)	Volume (Int)	Abs. Quant.	Rel. Quant.	Band %	Lane %
1		N/A	0,370	10 418 674	10 790 437	N/A	N/A	100,0	85,9

Band Detection	Automatically detected bands with sensitivity: Low
Lane Background	Lane background subtracted with disk size: 10
Lane Width	8.03 mm

Lane 6

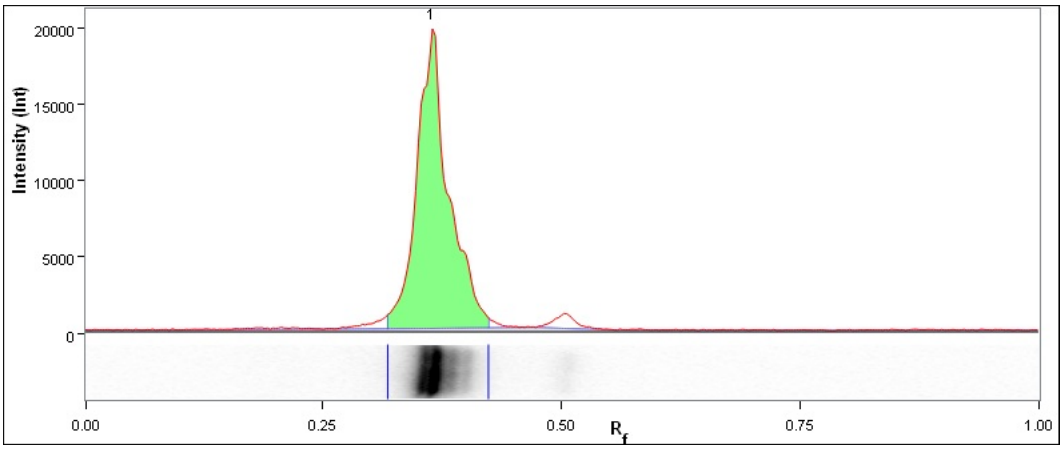

Band No.	Band Label	Mol. Wt. (KDa)	Relative Front	Adj. Volume (Int)	Volume (Int)	Abs. Quant.	Rel. Quant.	Band %	Lane %
1		N/A	0,367	14 869 296	15 344 368	N/A	N/A	100,0	90,3

Band Detection	Automatically detected bands with sensitivity: Low
Lane Background	Lane background subtracted with disk size: 10
Lane Width	8.52 mm

Lane 7

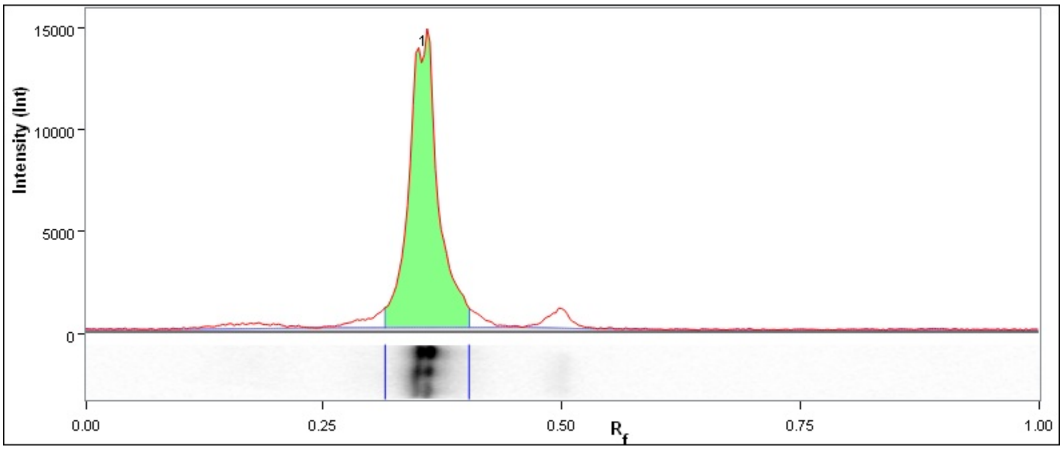

Band No.	Band Label	Mol. Wt. (KDa)	Relative Front	Adj. Volume (Int)	Volume (Int)	Abs. Quant.	Rel. Quant.	Band %	Lane %
1		N/A	0,358	10 334 636	10 708 932	N/A	N/A	100,0	82,9

Band Detection	Automatically detected bands with sensitivity: Low
Lane Background	Lane background subtracted with disk size: 10
Lane Width	8.52 mm

Volume Analysis

No.	Label	Type	Volume (Int)	Adj. Vol. (Int)	Mean Bkgd. (Int)	Abs. Quant.	Rel. Quant.	# of Pixels	Min. Value (Int)	Max. Value (Int)	Mean Value (Int)	Std. Dev.	Area (mm2)
1	U1	Unknown	11 513 596	7 299 880	1 734,0	N/A	N/A	2 430	0	33 868	4 738,1	5 916,6	65,2
2	U2	Unknown	10 166 384	4 947 899	2 147,5	N/A	N/A	2 430	0	23 716	4 183,7	5 093,4	65,2
3	U3	Unknown	12 379 220	7 059 685	2 189,1	N/A	N/A	2 430	0	21 448	5 094,3	5 090,0	65,2
4	U4	Unknown	12 203 348	7 329 394	2 005,7	N/A	N/A	2 430	0	24 980	5 022,0	5 978,8	65,2
5	U5	Unknown	12 324 188	6 921 889	2 223,2	N/A	N/A	2 430	0	26 232	5 071,7	6 016,4	65,2
6	U6	Unknown	15 996 628	11 115 600	2 008,7	N/A	N/A	2 430	0	28 172	6 583,0	6 666,6	65,2
7	U7	Unknown	11 407 928	8 042 161	1 385,1	N/A	N/A	2 430	0	31 356	4 694,6	5 534,1	65,2

Image Report: Histologia 2023-01-24 13hr 36min_Exposure_3.0sec 1a

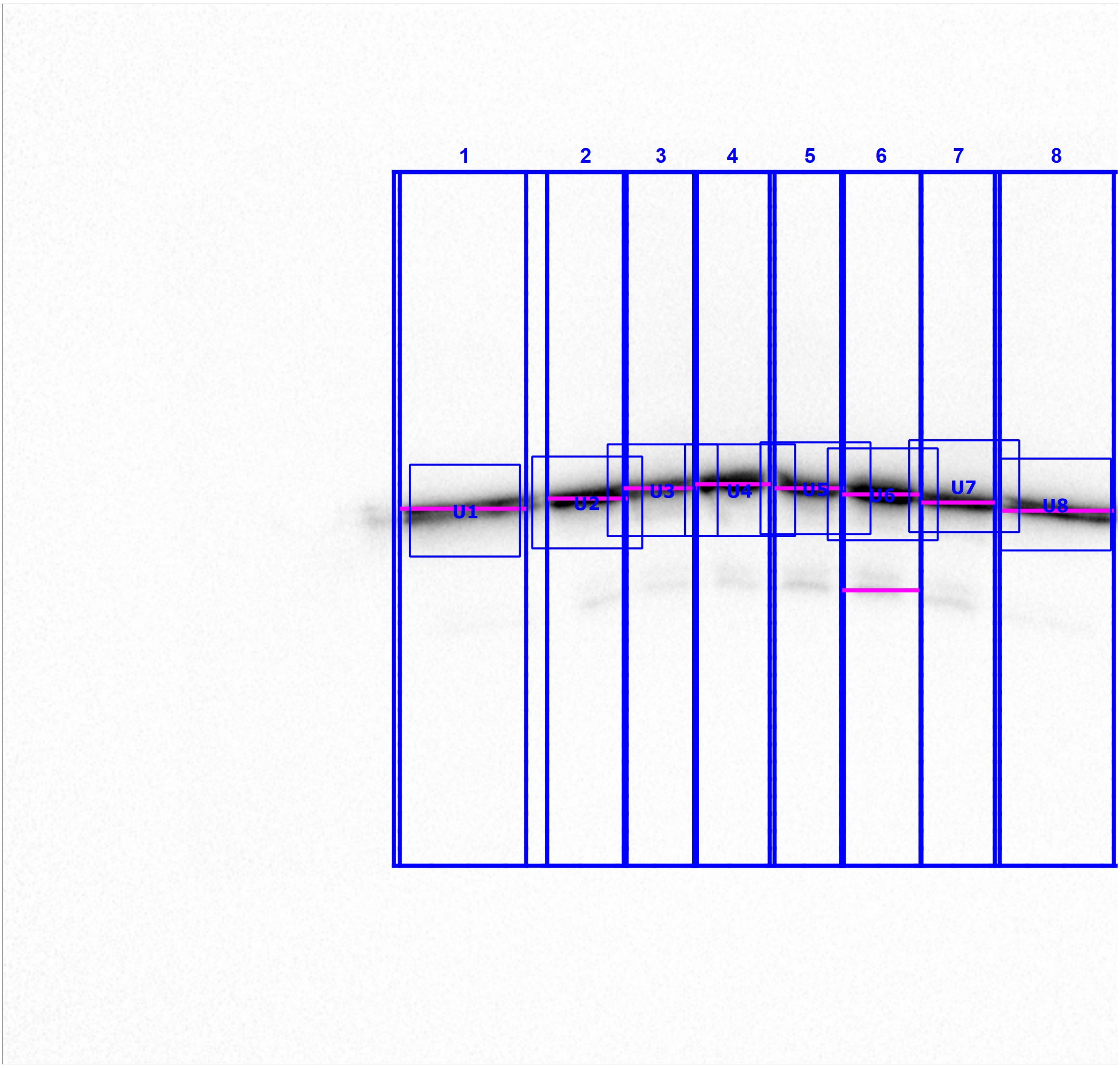

C:\Users\rusak\OneDrive\Dokumenty\Badania\CHI3L2 in BC\BC westerny ilościowo\ERK 24.1.23 BC\1\Histologia 2023-01-24 13hr 36min_Exposure_3.0sec 1a.scn

Acquisition Information

Imager	ChemiDoc MP
Exposure Time (sec)	3.000 (Signal Accumulation)
Flat Field	Applied (Lens)
Serial Number	731BR01769

Software Version	5.0
Application	Chemi Hi Resolution
Excitation Source	No Illumination
Emission Filter	No Filter
Binning	2x2

Image Information

Acquisition Date	24/1/2023 1:37:06 PM
User Name	Histologia
Image Area (mm)	X: 114.0 Y: 85.2
Pixel Size (µm)	X: 163.8 Y: 163.8
Data Range (Int)	0 - 31900

Analysis Settings

Detection	Lane detection:<br>Manually created lanes<br><br>Band detection:<br>Automatically detected bands with sensitivity: Low<br><br>Lane Background Subtraction:<br>Lane background subtracted with disk size: 10<br><br>Lane width: Variable
Volume Analysis	Background subtraction method: Local<br>Quantity regression method: Linear

Lane Statistics

Lane No.	Adj. Total Band Vol. (Int)	Total Band Vol. (Int)	Adj. Total Lane Vol. (Int)	Total Lane Vol. (Int)	Bkgd. Vol. (Int)	Norm. Factor
1	12 608 506	13 097 004	14 641 610	18 840 374	4 198 764	N/A
2	10 087 350	10 581 636	11 586 705	14 775 228	3 188 523	N/A
3	7 797 564	8 450 388	9 246 708	13 363 740	4 117 032	N/A
4	10 275 566	10 941 418	12 275 490	16 061 774	3 786 284	N/A
5	8 264 380	9 171 092	9 875 130	14 108 402	4 233 272	N/A
6	13 127 127	14 774 916	14 165 268	18 257 772	4 092 504	N/A
7	8 674 812	9 189 108	10 717 488	14 035 824	3 318 336	N/A
8	10 910 368	11 288 928	13 148 016	16 933 896	3 785 880	N/A

Lane And Band Analysis

Lane 1

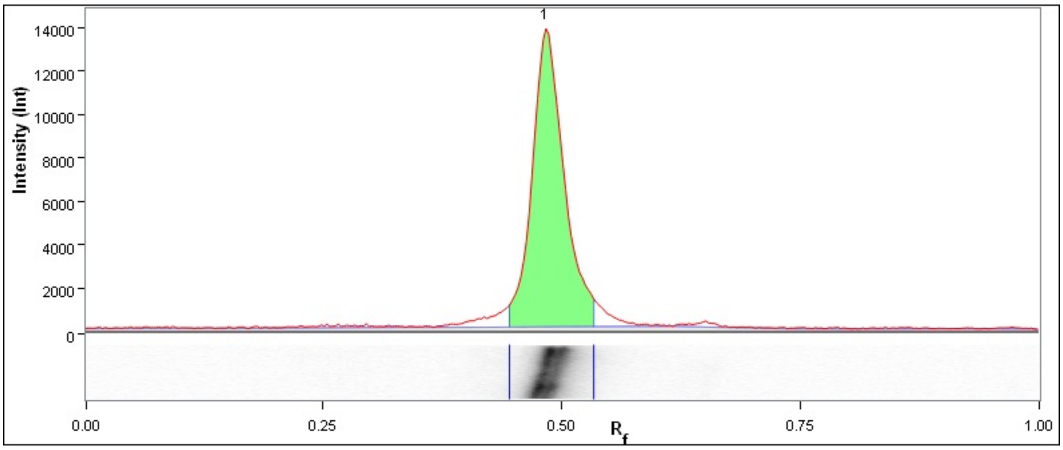

Band No.	Band Label	Mol. Wt. (KDa)	Relative Front	Adj. Volume (Int)	Volume (Int)	Abs. Quant.	Rel. Quant.	Band %	Lane %
1		N/A	0,485	12 608 506	13 097 004	N/A	N/A	100,0	86,1

Band Detection	Automatically detected bands with sensitivity: Low
Lane Background	Lane background subtracted with disk size: 10
Lane Width	10.16 mm

Lane 2

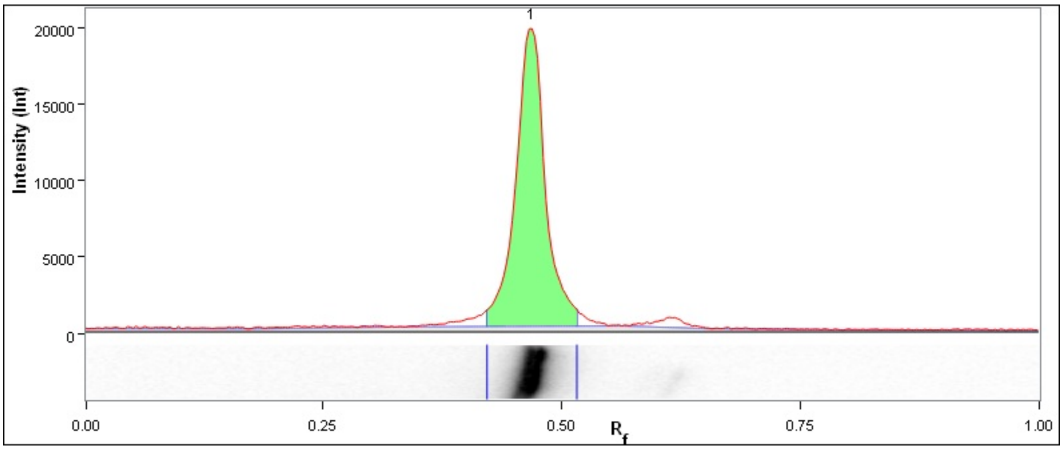

Band No.	Band Label	Mol. Wt. (KDa)	Relative Front	Adj. Volume (Int)	Volume (Int)	Abs. Quant.	Rel. Quant.	Band %	Lane %
1		N/A	0,471	10 087 350	10 581 636	N/A	N/A	100,0	87,1

Band Detection	Automatically detected bands with sensitivity: Low
Lane Background	Lane background subtracted with disk size: 10
Lane Width	6.39 mm

Lane 3

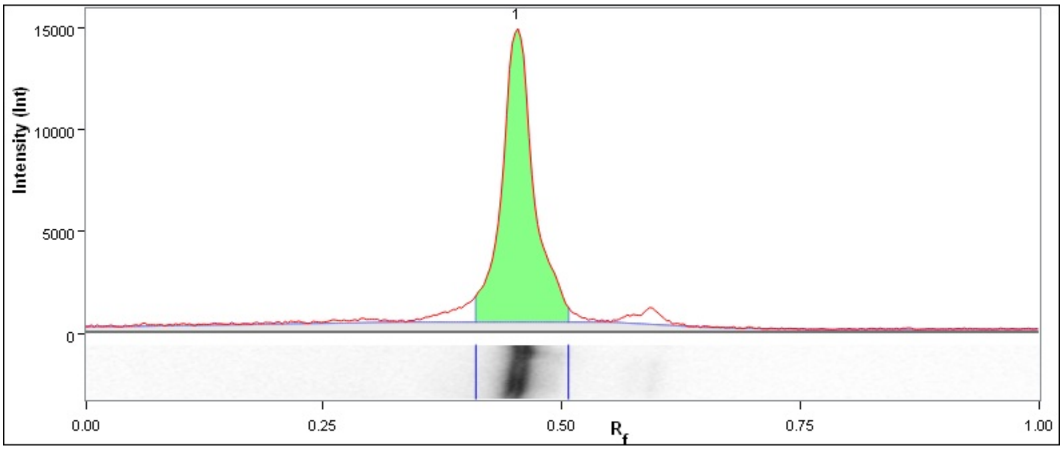

Band No.	Band Label	Mol. Wt. (KDa)	Relative Front	Adj. Volume (Int)	Volume (Int)	Abs. Quant.	Rel. Quant.	Band %	Lane %
1		N/A	0,456	7 797 564	8 450 388	N/A	N/A	100,0	84,3

Band Detection	Automatically detected bands with sensitivity: Low
Lane Background	Lane background subtracted with disk size: 10
Lane Width	5.90 mm

Lane 4

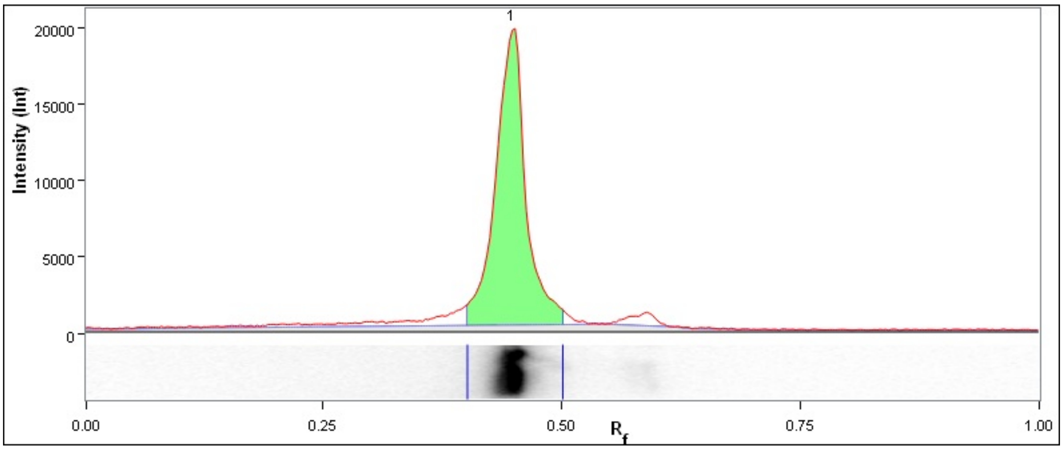

Band No.	Band Label	Mol. Wt. (KDa)	Relative Front	Adj. Volume (Int)	Volume (Int)	Abs. Quant.	Rel. Quant.	Band %	Lane %
1		N/A	0,450	10 275 566	10 941 418	N/A	N/A	100,0	83,7

Band Detection	Automatically detected bands with sensitivity: Low
Lane Background	Lane background subtracted with disk size: 10
Lane Width	6.06 mm

Lane 5

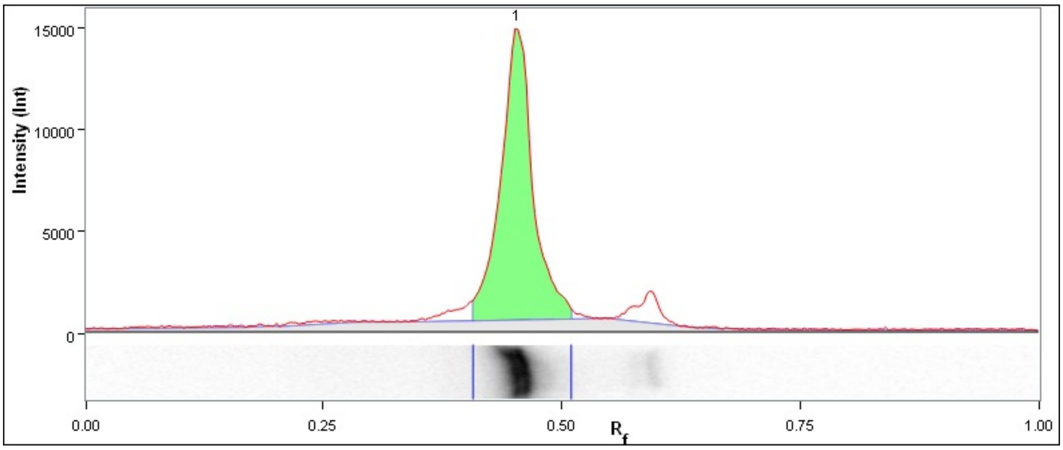

Band No.	Band Label	Mol. Wt. (KDa)	Relative Front	Adj. Volume (Int)	Volume (Int)	Abs. Quant.	Rel. Quant.	Band %	Lane %
1		N/A	0,456	8 264 380	9 171 092	N/A	N/A	100,0	83,7

Band Detection	Automatically detected bands with sensitivity: Low
Lane Background	Lane background subtracted with disk size: 10
Lane Width	5.57 mm

Lane 6

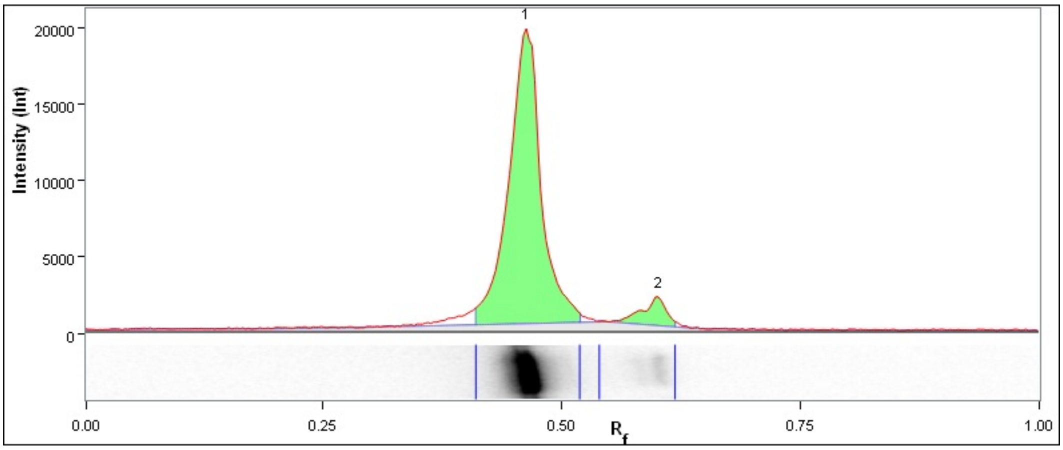

Band No.	Band Label	Mol. Wt. (KDa)	Relative Front	Adj. Volume (Int)	Volume (Int)	Abs. Quant.	Rel. Quant.	Band %	Lane %
1		N/A	0,465	12 233 793	13 205 868	N/A	N/A	93,2	86,4
2		N/A	0,603	893 334	1 569 048	N/A	N/A	6,8	6,3

Band Detection	Automatically detected bands with sensitivity: Low
Lane Background	Lane background subtracted with disk size: 10
Lane Width	6.39 mm

Lane 7

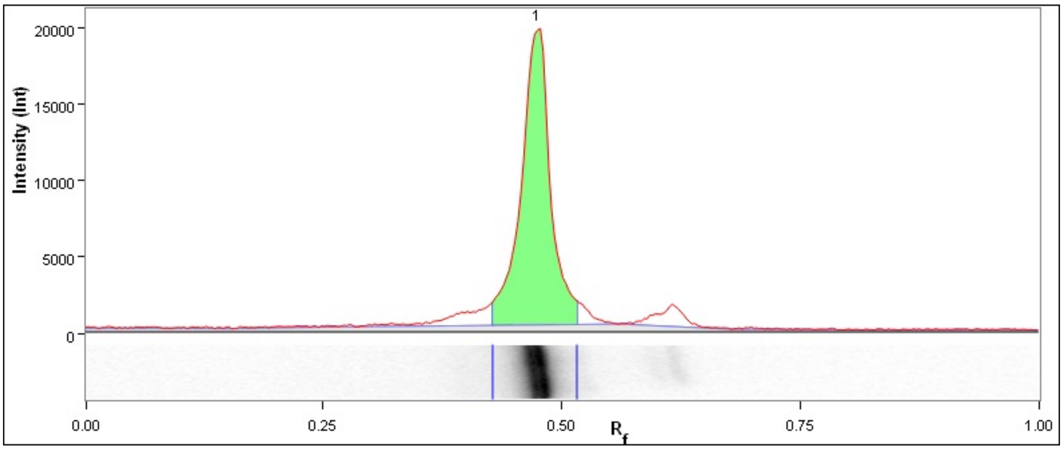

Band No.	Band Label	Mol. Wt. (KDa)	Relative Front	Adj. Volume (Int)	Volume (Int)	Abs. Quant.	Rel. Quant.	Band %	Lane %
1		N/A	0,476	8 674 812	9 189 108	N/A	N/A	100,0	80,9

Band Detection	Automatically detected bands with sensitivity: Low
Lane Background	Lane background subtracted with disk size: 10
Lane Width	5.90 mm

Lane 8

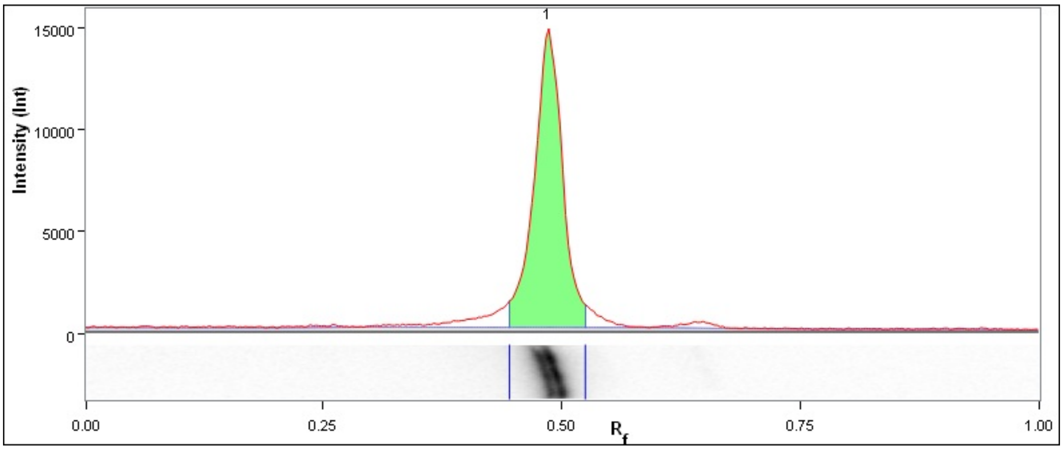

Band No.	Band Label	Mol. Wt. (KDa)	Relative Front	Adj. Volume (Int)	Volume (Int)	Abs. Quant.	Rel. Quant.	Band %	Lane %
1		N/A	0,488	10 910 368	11 288 928	N/A	N/A	100,0	83,0

Band Detection	Automatically detected bands with sensitivity: Low
Lane Background	Lane background subtracted with disk size: 10
Lane Width	9.17 mm

Volume Analysis

No.	Label	Type	Volume (Int)	Adj. Vol. (Int)	Mean Bkgd. (Int)	Abs. Quant.	Rel. Quant.	# of Pixels	Min. Value (Int)	Max. Value (Int)	Mean Value (Int)	Std. Dev.	Area (mm2)
1	U1	Unknown	12 477 660	6 082 189	2 631,9	N/A	N/A	2 430	0	20 092	5 134,8	5 285,4	65,2
2	U2	Unknown	14 173 812	7 905 134	2 579,7	N/A	N/A	2 430	0	27 280	5 832,8	6 545,6	65,2
3	U3	Unknown	14 293 308	5 360 724	3 676,0	N/A	N/A	2 430	92	28 836	5 882,0	5 916,9	65,2

4	U4	Unknown	15 555 536	7 221 983	3 429,4	N/A	N/A	2 430	0	28 836	6 401,5	6 756,2	65,2
5	U5	Unknown	15 141 056	5 375 487	4 018,8	N/A	N/A	2 430	0	28 700	6 230,9	6 178,4	65,2
6	U6	Unknown	17 525 120	9 143 400	3 449,3	N/A	N/A	2 430	368	31 900	7 212,0	7 488,5	65,2
7	U7	Unknown	13 682 548	4 927 138	3 603,0	N/A	N/A	2 430	140	24 696	5 630,7	5 896,2	65,2
8	U8	Unknown	11 981 928	7 120 966	2 000,4	N/A	N/A	2 430	0	23 276	4 930,8	5 652,7	65,2

Image Report: Histologia 2023-01-24 13hr 38min_Exposure_3.0sec 2a

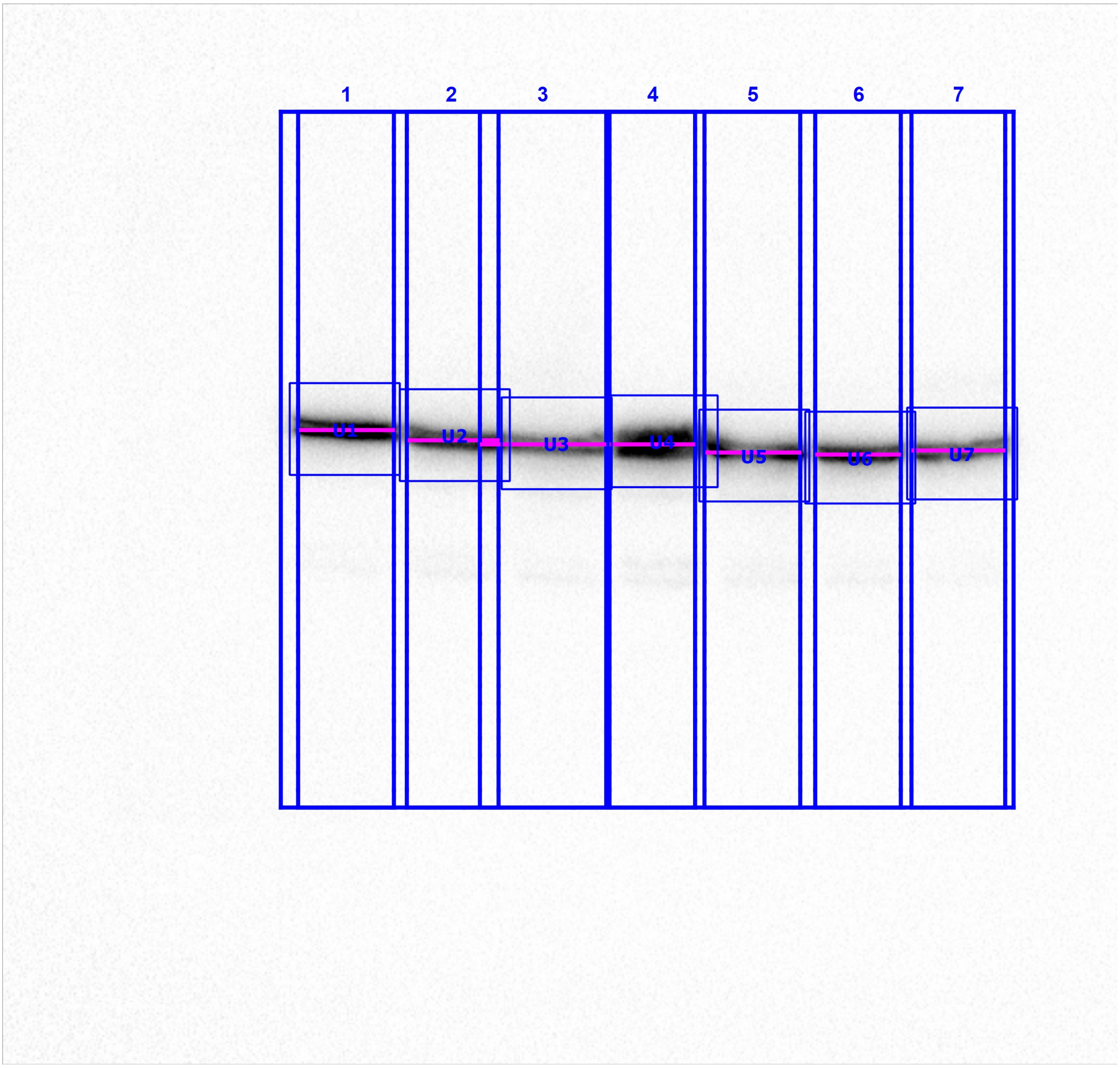

C:\Users\rusak\OneDrive\Dokumenty\Badania\CHI3L2 in BC\BC westerny ilościowo\ERK 24.1.23 BC\2\Histologia 2023-01-24 13hr 38min_Exposure_3.0sec 2a.scn

Acquisition Information

Imager	ChemiDoc MP
Exposure Time (sec)	3.000 (Signal Accumulation)
Flat Field	Applied (Lens)
Serial Number	731BR01769

Software Version	5.0
Application	Chemi Hi Resolution
Excitation Source	No Illumination
Emission Filter	No Filter
Binning	2x2

Image Information

Acquisition Date	24/1/2023 1:38:38 PM
User Name	Histologia
Image Area (mm)	X: 114.0 Y: 85.2
Pixel Size (µm)	X: 163.8 Y: 163.8
Data Range (Int)	0 - 23772

Analysis Settings

Detection	Lane detection:<br>Manually created lanes<br><br>Band detection:<br>Automatically detected bands with sensitivity: Low<br><br>Lane Background Subtraction:<br>Lane background subtracted with disk size: 10<br><br>Lane width: Variable
Volume Analysis	Background subtraction method: Local<br>Quantity regression method: Linear

Lane Statistics

Lane No.	Adj. Total Band Vol. (Int)	Total Band Vol. (Int)	Adj. Total Lane Vol. (Int)	Total Lane Vol. (Int)	Bkgd. Vol. (Int)	Norm. Factor
1	9 925 460	10 281 720	11 530 980	14 448 787	2 917 807	N/A
2	7 885 440	8 234 640	9 623 070	12 356 415	2 733 345	N/A
3	9 282 888	9 741 378	12 073 198	15 788 672	3 715 474	N/A
4	12 288 276	12 619 950	14 113 638	16 157 148	2 043 510	N/A
5	8 712 296	9 085 664	10 621 389	13 207 564	2 586 175	N/A
6	7 312 032	7 560 840	9 014 082	10 939 656	1 925 574	N/A
7	6 370 586	6 629 428	7 946 960	10 162 366	2 215 406	N/A

Lane And Band Analysis

Lane 1

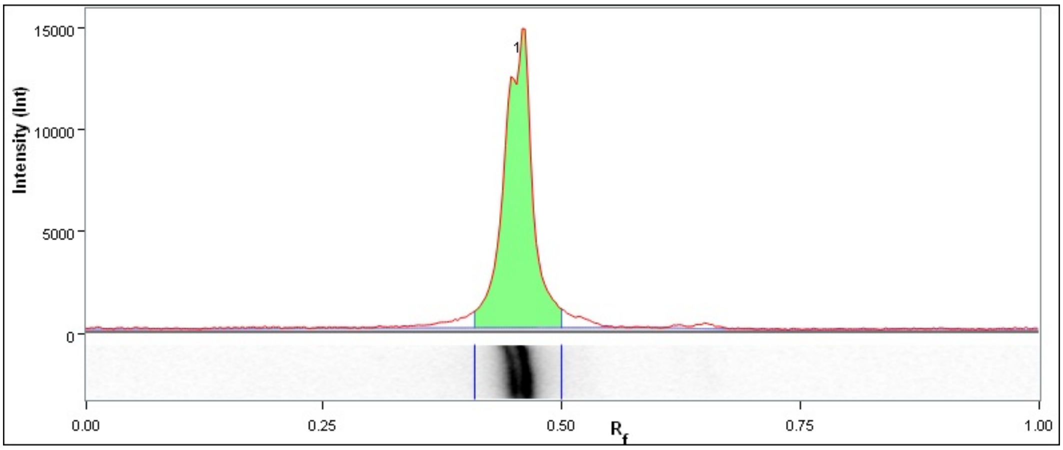

Band No.	Band Label	Mol. Wt. (KDa)	Relative Front	Adj. Volume (Int)	Volume (Int)	Abs. Quant.	Rel. Quant.	Band %	Lane %
1		N/A	0,457	9 925 460	10 281 720	N/A	N/A	100,0	86,1

Band Detection	Automatically detected bands with sensitivity: Low
Lane Background	Lane background subtracted with disk size: 10
Lane Width	7.70 mm

Lane 2

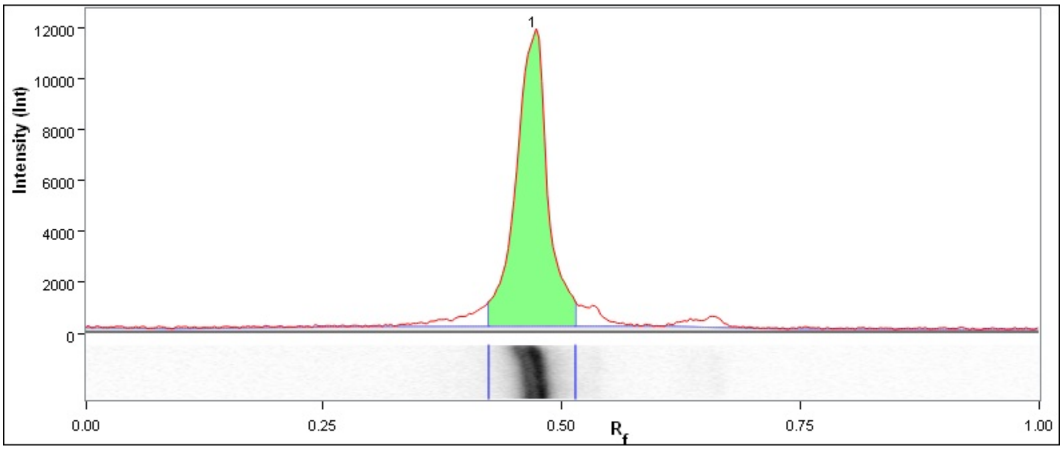

Band No.	Band Label	Mol. Wt. (KDa)	Relative Front	Adj. Volume (Int)	Volume (Int)	Abs. Quant.	Rel. Quant.	Band %	Lane %
1		N/A	0,472	7 885 440	8 234 640	N/A	N/A	100,0	81,9

Band Detection	Automatically detected bands with sensitivity: Low
Lane Background	Lane background subtracted with disk size: 10
Lane Width	7.37 mm

Lane 3

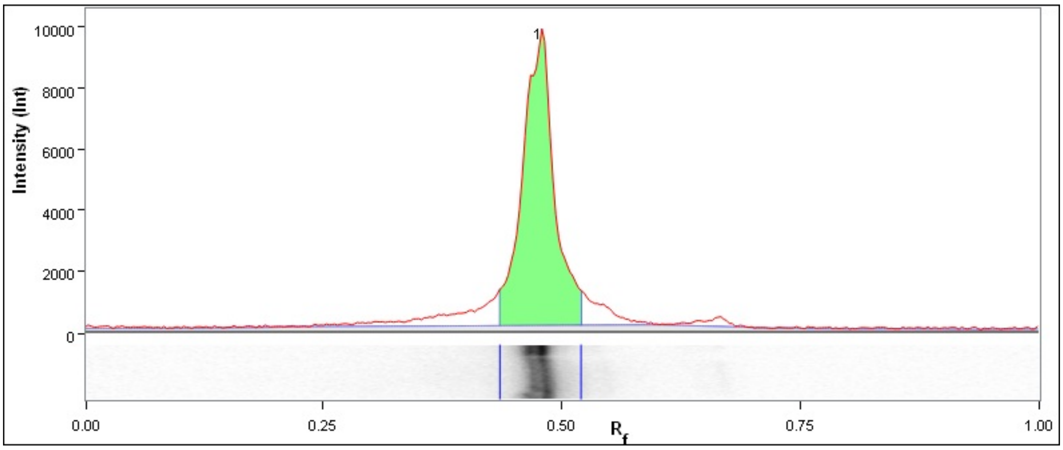

Band No.	Band Label	Mol. Wt. (KDa)	Relative Front	Adj. Volume (Int)	Volume (Int)	Abs. Quant.	Rel. Quant.	Band %	Lane %
1		N/A	0,478	9 282 888	9 741 378	N/A	N/A	100,0	76,9

Band Detection	Automatically detected bands with sensitivity: Low
Lane Background	Lane background subtracted with disk size: 10
Lane Width	10.16 mm

Lane 4

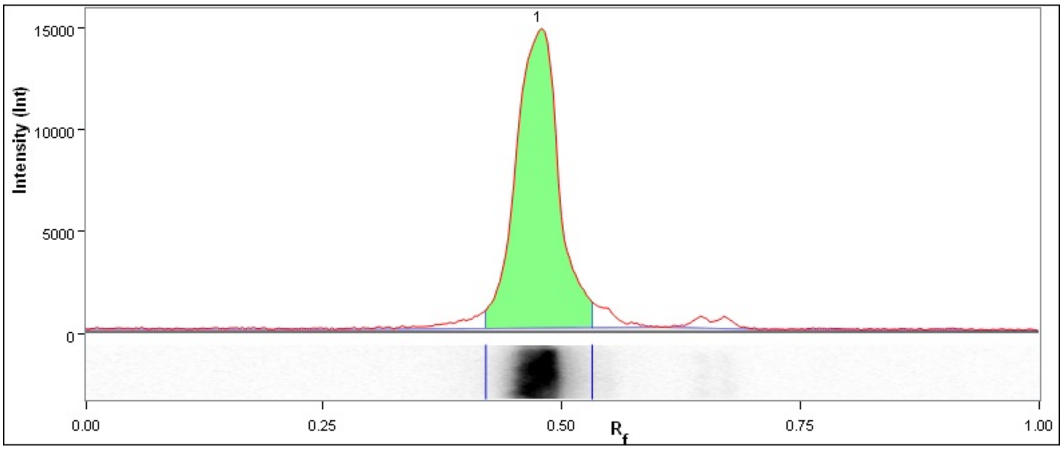

Band No.	Band Label	Mol. Wt. (KDa)	Relative Front	Adj. Volume (Int)	Volume (Int)	Abs. Quant.	Rel. Quant.	Band %	Lane %
1		N/A	0,478	12 288 276	12 619 950	N/A	N/A	100,0	87,1

Band Detection	Automatically detected bands with sensitivity: Low
Lane Background	Lane background subtracted with disk size: 10
Lane Width	6.88 mm

Lane 5

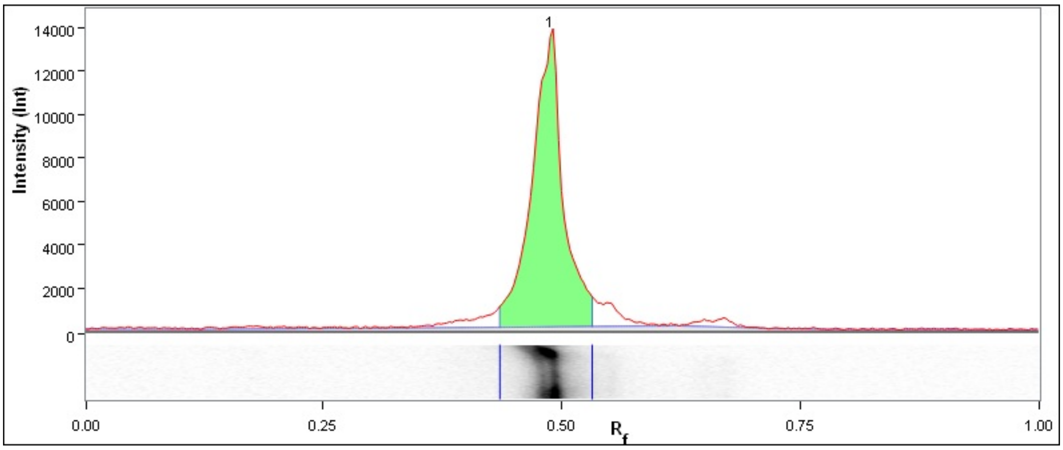

Band No.	Band Label	Mol. Wt. (KDa)	Relative Front	Adj. Volume (Int)	Volume (Int)	Abs. Quant.	Rel. Quant.	Band %	Lane %
1		N/A	0,490	8 712 296	9 085 664	N/A	N/A	100,0	82,0

Band Detection	Automatically detected bands with sensitivity: Low
Lane Background	Lane background subtracted with disk size: 10
Lane Width	7.70 mm

Lane 6

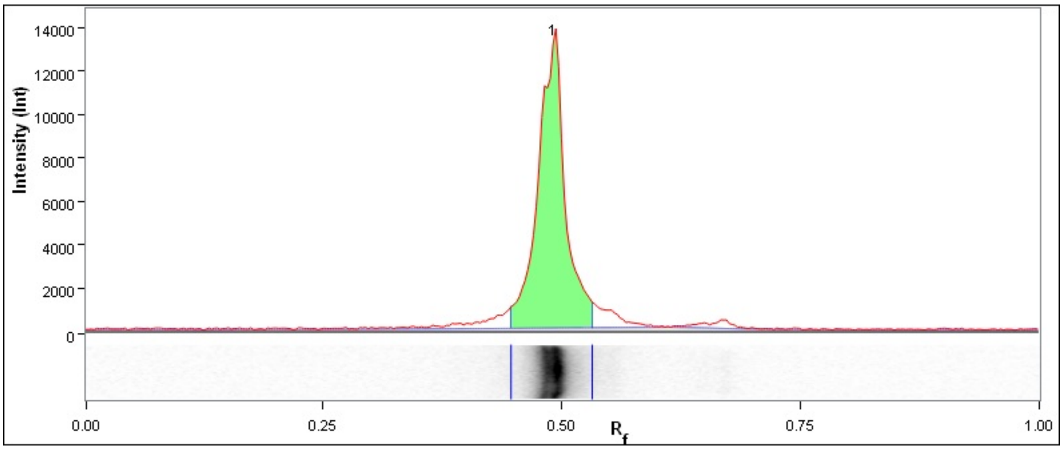

Band No.	Band Label	Mol. Wt. (KDa)	Relative Front	Adj. Volume (Int)	Volume (Int)	Abs. Quant.	Rel. Quant.	Band %	Lane %
1		N/A	0,493	7 312 032	7 560 840	N/A	N/A	100,0	81,1

Band Detection	Automatically detected bands with sensitivity: Low
Lane Background	Lane background subtracted with disk size: 10
Lane Width	6.88 mm

Lane 7

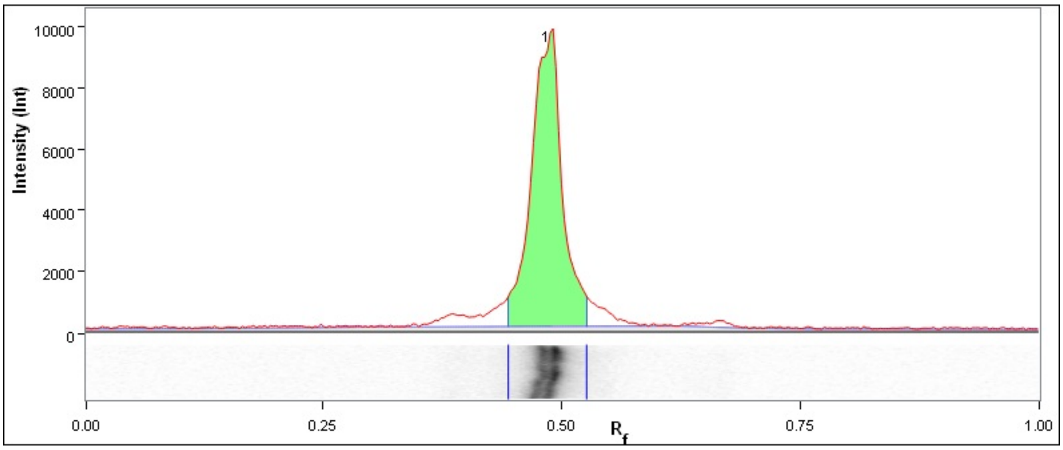

Band No.	Band Label	Mol. Wt. (KDa)	Relative Front	Adj. Volume (Int)	Volume (Int)	Abs. Quant.	Rel. Quant.	Band %	Lane %
1		N/A	0,487	6 370 586	6 629 428	N/A	N/A	100,0	80,2

Band Detection	Automatically detected bands with sensitivity: Low
Lane Background	Lane background subtracted with disk size: 10
Lane Width	7.53 mm

Volume Analysis

No.	Label	Type	Volume (Int)	Adj. Vol. (Int)	Mean Bkgd. (Int)	Abs. Quant.	Rel. Quant.	# of Pixels	Min. Value (Int)	Max. Value (Int)	Mean Value (Int)	Std. Dev.	Area (mm2)
1	U1	Unknown	11 839 820	8 465 103	1 388,8	N/A	N/A	2 430	0	21 688	4 872,4	5 476,5	65,2

2	U2	Unknown	10 212 072	5 489 307	1 943,5	N/A	N/A	2 430	0	16 224	4 202,5	4 238,0	65,2
3	U3	Unknown	9 118 244	3 631 785	2 257,8	N/A	N/A	2 430	108	14 696	3 752,4	3 281,7	65,2
4	U4	Unknown	15 737 216	9 135 459	2 716,8	N/A	N/A	2 430	0	23 772	6 476,2	5 806,3	65,2
5	U5	Unknown	11 032 112	5 445 037	2 299,2	N/A	N/A	2 430	56	23 772	4 540,0	4 445,8	65,2
6	U6	Unknown	9 622 920	4 806 997	1 981,9	N/A	N/A	2 430	0	19 260	3 960,0	4 241,4	65,2
7	U7	Unknown	7 914 584	5 681 582	918,9	N/A	N/A	2 430	0	14 928	3 257,0	3 406,0	65,2

G1
G2
G3

1 żel 1 powt YKL39 UMED 2018-02-13 11hr 00min_Exposure_60.0sec

No.	Label	Volume (Int)	Adj. Vol. (I	Mean Bkg	Abs. Quan	Rel. Quant	# of Pixels	Min. Value	Max. Value	Mean Valu	Std. Dev.	Area (mm2
1	U1	7117380,00	5499270	749,125	N/A	N/A	2160	0	18 792,000(	3 295,0833	3 588,5394	41,95454
2	U2	11621504,00	8714279	1 345,9375	N/A	N/A	2160	0	19 276,000(	5 380,3259	4 893,8441	41,95454
3	U3	6160516,00	4447906	792,875	N/A	N/A	2160	0	11 200,000(	2 852,0907	1 982,4505	41,95454
4	U4	756348,00	136203	287,1042	N/A	N/A	2160	0	2 212,0000(	350,1611	348,1578	41,95454
5	U5	10484952,00	8792907	783,3542	N/A	N/A	2160	0	18 576,000(	4 854,1444	4 501,6403	41,95454
6	U6	2358272,00	958952	647,8333	N/A	N/A	2160	0	3 296,0000(	1 091,7925	589,9416	41,95454
7	U7	4284864,00	2318319	910,4375	N/A	N/A	2160	0	7 616,0000(	1 983,7333	1 285,2939	41,95454
8	U8	17656584,00	13993809	1 695,7291	N/A	N/A	2160	0	31 556,000(	8 174,3444	7 453,4712	41,95454

1 żel 2 powt YKL-39

No.	Label	Volume (Int)	Adj. Vol. (I	Mean Bkg	Abs. Quan	Rel. Quant	# of Pixels	Min. Value	Max. Value	Mean Valu	Std. Dev.	Area (mm2
1	U1	2 654 244,000(	1819224	386,5833	N/A	N/A	2160	0	7 628,0000(	1 228,8166	1 121,9099	47,30528
2	U2	6 239 580,000(	4654320	733,9167	N/A	N/A	2160	0	12 864,000(	2 888,6944	2 650,2353	47,30528
3	U3	3 737 504,000(	2500499	572,6875	N/A	N/A	2160	0	5 820,0000(	1 730,3259	1 129,9091	47,30528
4	U4	663 332,00000	-67828	338,5	N/A	N/A	2160	0	1 696,0000(	307,0981	316,1672	47,30528
5	U5	4 888 684,000(	3976309	422,3958	N/A	N/A	2160	0	9 092,0000(	2 263,2796	2 069,3441	47,30528
6	U6	1 389 776,000(	483431	419,6042	N/A	N/A	2160	0	1 924,0000(	643,4148	412,7609	47,30528
7	U7	2 445 720,000(	1334175	514,6042	N/A	N/A	2160	0	3 944,0000(	1 132,2777	767,9922	47,30528
8	U8	7 747 948,000(	6280723	679,2708	N/A	N/A	2160	0	19 652,000(	3 587,0129	3 889,5426	47,30528

1 żel 3 powt YKL39 UMED 2018-01-24 11hr 40min_Exposure_60.0sec

No.	Label	Volume (Int)	Adj. Vol. (I	Mean Bkg	Abs. Quan	Rel. Quant	# of Pixels	Min. Value	Max. Value	Mean Valu	Std. Dev.	Area (mm2
1	U1	15 059 776,00(	11674471	1 567,2708	N/A	N/A	2160	0	43 780,000(	6 972,1185	7 528,4948	46,3912
2	U2	19 532 856,00(	14940741	2 125,9791	N/A	N/A	2160	140	37 304,000(	9 042,9888	8 480,6932	46,3912
3	U3	17 882 756,00(	13368761	2 089,8125	N/A	N/A	2160	120	23 668,000(	8 279,0537	5 547,3136	46,3912
4	U4	2 131 088,000(	485888	761,6667	N/A	N/A	2160	0	5 240,0000(	986,6148	775,2589	46,3912
5	U5	18 758 648,00(	15533633	1 493,0625	N/A	N/A	2160	0	32 348,000(	8 684,5592	8 053,6277	46,3912
6	U6	5 611 360,000(	2512120	1 434,8333	N/A	N/A	2160	0	9 692,0000(	2 597,8518	1 777,4232	46,3912
7	U7	9 889 544,000(	5365739	2 094,3541	N/A	N/A	2160	0	15 972,000(	4 578,4925	2 945,2640	46,3912
8	U8	28 275 136,00(	23097751	2 396,9375	N/A	N/A	2160	0	52 820,000(	13 090,340	12 381,869	46,3912

1 żel 1 powt tub UMED 2018-02-14 10hr 53min_Exposure_16.8sec

No.	Label	Volume (In	Adj. Vol. (I	Mean Bkgd	Abs. Quan	Rel. Quant	# of Pixels	Min. Value	Max. Value	Mean Valu	Std. Dev.	Area (mm2
1	U1	6 412 800,	4697951	567,6429	N/A	N/A	3021	0	13 768,00	2 122,740	2 792,256	58,67808
2	U2	9 737 924,	7275323	815,1607	N/A	N/A	3021	0	15 444,00	3 223,4107	3 539,639	58,67808
3	U3	9 947 944,	7362292	855,8929	N/A	N/A	3021	0	14 112,00	3 292,930	3 497,202	58,67808
4	U4	6 741 460,	4815465	637,5357	N/A	N/A	3021	0	12 436,00	2 231,532	2 955,913	58,67808
5	U5	13 185 08	10254872	969,9464	N/A	N/A	3021	0	19 784,00	4 364,475	5 058,015	58,67808
6	U6	15 887 16	11697033	1 387,000	N/A	N/A	3021	0	21 256,00	5 258,907	5 223,102	58,67808
7	U7	14 483 91	10436315	1 339,821	N/A	N/A	3021	0	20 248,00	4 794,4111	5 206,002	58,67808
8	U8	16 883 06	12662669	1 397,017	N/A	N/A	3021	0	26 024,00	5 588,5667	5 975,963	58,67808

1 żel 2 powt tub UMED 2018-02-21 10hr 54min_Exposure_16.8sec

No.	Label	Volume (In	Adj. Vol. (I	Mean Bkgd	Abs. Quan	Rel. Quant	# of Pixels	Min. Value	Max. Value	Mean Valu	Std. Dev.	Area (mm2
1	U1	7 336 200,	4203585	1 036,946	N/A	N/A	3021	0	18 048,00	2 428,4011	3 260,4601	66,16169
2	U2	19 774 28	15038539	1 567,6071	N/A	N/A	3021	0	41 216,00	6 545,607	8 340,8111	66,16169
3	U3	14 582 61	11654670	969,1964	N/A	N/A	3021	0	21 848,00	4 827,081	5 821,7131	66,16169
4	U4	11 593 19	8635205	979,1429	N/A	N/A	3021	0	31 424,00	3 837,535	6 081,473	66,16169
5	U5	20 025 58	14690386	1 766,0357	N/A	N/A	3021	0	39 784,00	6 628,7917	8 282,968	66,16169
6	U6	19 812 10	13316796	2 150,053	N/A	N/A	3021	0	36 400,00	6 558,129	7 368,461	66,16169
7	U7	16 161 81	10355076	1 922,125	N/A	N/A	3021	0	29 476,00	5 349,823	6 130,166	66,16169
8	U8	21 916 60	16726364	1 718,053	N/A	N/A	3021	0	40 996,00	7 254,751	8 877,6261	66,16169

1 żel 3 powt tub UMED 2018-01-26 11hr 11min_Exposure_16.8sec

No.	Label	Volume (In	Adj. Vol. (I	Mean Bkgd	Abs. Quan	Rel. Quant	# of Pixels	Min. Value	Max. Value	Mean Valu	Std. Dev.	Area (mm2
1	U1	12 122 63	8420504	1 225,464	N/A	N/A	3021	0	21 836,00	4 012,787	5 013,797	66,16169
2	U2	20 399 60	13797752	2 185,321	N/A	N/A	3021	316	24 324,00	6 752,6011	5 661,569	66,16169
3	U3	15 698 92	10918839	1 582,2857	N/A	N/A	3021	0	17 156,00	5 196,598	4 508,827	66,16169
4	U4	8 169 820,	5187068	987,3393	N/A	N/A	3021	0	14 092,00	2 704,342	3 238,137	66,16169
5	U5	13 050 41	8911426	1 370,071	N/A	N/A	3021	0	18 156,00	4 319,898	4 324,725	66,16169
6	U6	14 590 82	8347222	2 066,7321	N/A	N/A	3021	192	15 952,00	4 829,798	3 830,513	66,16169
7	U7	18 480 56	11949274	2 161,964	N/A	N/A	3021	112	21 108,00	6 117,3677	5 267,598	66,16169
8	U8	17 594 08	12535585	1 674,446	N/A	N/A	3021	0	21 648,00	5 823,928	5 279,9821	66,16169

2 żel 1 powt YKL39 UMED 2018-02-13 11hr 08min_Exposure_60.0sec

No.	Label	Volume (In	Adj. Vol. (I	Mean Bkgd	Abs. Quan	Rel. Quant	# of Pixels	Min. Value	Max. Value	Mean Valu	Std. Dev.	Area (mm2
1	U1	7 762 124,	5709158	718,8257	N/A	N/A	2856	0	16 020,000	2 717,8305	3 155,0418	55,47322
2	U2	5 608 264,	3938211	584,7523	N/A	N/A	2856	0	9 224,0000	1 963,6778	1 852,0520	55,47322
3	U3	2 766 124,	1247204	531,8349	N/A	N/A	2856	0	6 796,0000	968,5308	604,7804	55,47322
4	U4	2 401 768,	1038225	477,4312	N/A	N/A	2856	0	2 528,0000	840,9552	461,7564	55,47322
5	U5	4 674 784,	3182590	522,4771	N/A	N/A	2856	0	9 116,0000	1 636,8291	1 337,4277	55,47322
6	U6	9 349 960,	6693670	930,0734	N/A	N/A	2856	0	22 700,000	3 273,7955	3 797,6115	55,47322
7	U7	13 867 160	9274817	1 607,9633	N/A	N/A	2856	0	24 020,000	4 855,4481	4 418,9764	55,47322

2 żel 2 powt YKL39 UMED 2018-02-20 10hr 58min_Exposure_60.0sec

No.	Label	Volume (In	Adj. Vol. (I	Mean Bkgd	Abs. Quan	Rel. Quant	# of Pixels	Min. Value	Max. Value	Mean Valu	Std. Dev.	Area (mm2
1	U1	6 289 876,	4557568	606,5505	N/A	N/A	2856	0	15 828,000	2 202,3375	2 753,4512	62,54809
2	U2	3 967 212,	2757264	423,6514	N/A	N/A	2856	0	9 492,0000	1 389,0798	1 463,0279	62,54809
3	U3	2 012 872,	1003735	353,3395	N/A	N/A	2856	0	2 564,0000	704,7871	472,728	62,54809
4	U4	1 555 100,	732048	288,1835	N/A	N/A	2856	0	2 096,0000	544,5028	420,9342	62,54809
5	U5	3 435 752,	2426300	353,4495	N/A	N/A	2856	0	4 784,0000	1 202,9943	1 007,7897	62,54809
6	U6	6 138 204,	4827536	458,9174	N/A	N/A	2856	0	12 048,000	2 149,2310	2 597,1631	62,54809
7	U7	5 470 280,	4302779	408,789	N/A	N/A	2856	0	9 960,0000	1 915,3641	2 022,0537	62,54809

2 żel 3 powt YKL39 UMED 2018-01-24 11hr 50min_Exposure_60.0sec

No.	Label	Volume (In	Adj. Vol. (I	Mean Bkgd	Abs. Quan	Rel. Quant	# of Pixels	Min. Value	Max. Value	Mean Valu	Std. Dev.	Area (mm2
1	U1	18 005 184	12768171	1 833,6880	N/A	N/A	2856	0	29 124,000	6 304,3361	6 321,2426	55,47322
2	U2	12 832 784	9512959	1 162,4036	N/A	N/A	2856	0	20 908,000	4 493,2717	3 948,9957	55,47322
3	U3	4 589 828,	1958011	921,5046	N/A	N/A	2856	0	8 476,0000	1 607,0826	746,9869	55,47322
4	U4	5 238 512,	2637089	910,8624	N/A	N/A	2856	0	4 432,0000	1 834,2128	857,975	55,47322
5	U5	11 444 108	6987752	1 560,3486	N/A	N/A	2856	0	14 984,000	4 007,0406	3 023,6136	55,47322
6	U6	17 756 144	12884070	1 705,9082	N/A	N/A	2856	0	26 760,000	6 217,1372	6 373,2896	55,47322
7	U7	15 453 284	10452402	1 751,0091	N/A	N/A	2856	0	25 216,000	5 410,8137	4 837,9975	55,47322

2 żel 1 powt tub UMED 2018-02-14 11hr 01min_Exposure_16.8sec

No.	Label	Volume (In	Adj. Vol. (I	Mean Bkgr	Abs. Quan	Rel. Quant	# of Pixels	Min. Value	Max. Value	Mean Valu	Std. Dev.	Area (mm2
1	U1	20 514 688	15951792	1 721,8476	N/A	N/A	2650	88	47 568,000	7 741,3916	8 731,4196	51,472
2	U2	12 310 576	8657665	1 378,4571	N/A	N/A	2650	0	23 840,000	4 645,5003	4 905,5084	51,472
3	U3	15 494 120	11418723	1 537,8857	N/A	N/A	2650	104	22 616,000	5 846,8377	5 456,4840	51,472
4	U4	18 004 248	13206486	1 810,4761	N/A	N/A	2650	400	25 764,000	6 794,0558	6 270,0064	51,472
5	U5	15 728 460	11345814	1 653,8285	N/A	N/A	2650	0	22 880,000	5 935,2679	6 054,6163	51,472
6	U6	13 646 120	10127879	1 327,6380	N/A	N/A	2650	0	28 808,000	5 149,4792	5 878,5851	51,472
7	U7	16 464 948	12268307	1 583,6380	N/A	N/A	2650	0	27 372,000	6 213,1879	6 352,8818	51,472

2 żel 2 powt tub

No.	Label	Volume (In	Adj. Vol. (I	Mean Bkgr	Abs. Quan	Rel. Quant	# of Pixels	Min. Value	Max. Value	Mean Valu	Std. Dev.	Area (mm2
1	U1	16 975 180	12441459	1 710,8380	N/A	N/A	2650	0	39 376,000	6 405,7283	7 369,4584	58,03657
2	U2	10 762 144	6388130	1 650,5714	N/A	N/A	2650	0	19 068,000	4 061,1864	4 354,4185	58,03657
3	U3	14 013 004	7805240	2 342,5523	N/A	N/A	2650	56	20 972,000	5 287,9260	5 231,6381	58,03657
4	U4	14 446 304	11152127	1 243,0857	N/A	N/A	2650	68	25 404,000	5 451,4354	5 655,2141	58,03657
5	U5	9 551 188,	7415238	806,019	N/A	N/A	2650	0	14 752,000	3 604,2218	3 916,0374	58,03657
6	U6	12 825 252	10036342	1 052,4190	N/A	N/A	2650	0	25 920,000	4 839,7177	5 750,1499	58,03657
7	U7	14 815 224	12084210	1 030,5714	N/A	N/A	2650	0	31 772,000	5 590,6505	6 435,2289	58,03657

2 żel 3 powt tub UMED 2018-01-26 11hr 19min_Exposure_16.8sec

No.	Label	Volume (In	Adj. Vol. (I	Mean Bkgr	Abs. Quan	Rel. Quant	# of Pixels	Min. Value	Max. Value	Mean Valu	Std. Dev.	Area (mm2
1	U1	24 537 896	16311236	3 104,4000	N/A	N/A	2650	636	36 384,000	9 259,5833	7 829,9501	51,472
2	U2	20 461 212	11925587	3 220,9904	N/A	N/A	2650	1 056,0000	28 568,000	7 721,2120	6 103,5903	51,472
3	U3	26 205 560	14503968	4 415,6952	N/A	N/A	2650	1 384,0000	28 200,000	9 888,8905	7 832,3173	51,472
4	U4	26 838 144	15154471	4 408,9333	N/A	N/A	2650	1 004,0000	28 972,000	10 127,601	6 844,5898	51,472
5	U5	20 666 096	12707465	3 003,2571	N/A	N/A	2650	600	24 400,000	7 798,5267	6 283,4541	51,472
6	U6	21 706 200	14317849	2 788,0571	N/A	N/A	2650	544	36 568,000	8 191,0188	8 190,4102	51,472
7	U7	33 580 124	24174340	3 549,3523	N/A	N/A	2650	944	51 760,000	12 671,744	12 775,995	51,472

3 żel 1 powt YKL39 UMED 2018-03-06 11hr 04min_Exposure_60.0sec

No.	Label	Volume (In	Adj. Vol. (I	Mean Bkgr	Abs. Quan	Rel. Quant	# of Pixels	Min. Value	Max. Value	Mean Valu	Std. Dev.	Area (mm2
1	U1	3 544 984,	2038312	627,78	N/A	N/A	2400	0	5 660,0000	1 477,0766	922,2807	46,61615
2	U2	7 145 564,	5390684	731,2	N/A	N/A	2400	0	13 672,000	2 977,3183	2 690,7798	46,61615
3	U3	7 988 104,	6376120	671,66	N/A	N/A	2400	0	17 288,000	3 328,3766	3 506,8365	46,61615
4	U4	1 778 856,	1062360	298,54	N/A	N/A	2400	0	3 620,0000	741,19	645,8735	46,61615
5	U5	3 296 888,	2194040	459,52	N/A	N/A	2400	0	5 872,0000	1 373,7033	1 160,4969	46,61615
6	U6	2 382 332,	1655324	302,92	N/A	N/A	2400	0	4 620,0000	992,6383	859,5959	46,61615
7	U7	1 016 152,	312952	293	N/A	N/A	2400	0	2 316,0000	423,3967	370,0445	46,61615

3 żel 2 powt YKL39 UMED 2018-02-28 11hr 01min_Exposure_60.0sec

No.	Label	Volume (In	Adj. Vol. (I	Mean Bkgr	Abs. Quan	Rel. Quant	# of Pixels	Min. Value	Max. Value	Mean Valu	Std. Dev.	Area (mm2
1	U1	5 977 176,	3923592	855,66	N/A	N/A	2400	0	9 416,0000	2 490,4900	1 709,1494	52,56142
2	U2	6 020 140,	4318924	708,84	N/A	N/A	2400	0	11 560,000	2 508,3916	2 358,0519	52,56142
3	U3	6 625 432,	5175832	604	N/A	N/A	2400	0	13 260,000	2 760,5966	2 902,1411	52,56142
4	U4	3 414 764,	2363756	437,92	N/A	N/A	2400	0	7 476,0000	1 422,8183	1 406,3820	52,56142
5	U5	3 038 504,	1899752	474,48	N/A	N/A	2400	0	7 108,0000	1 266,0433	1 112,8064	52,56142
6	U6	4 810 780,	3458380	563,5	N/A	N/A	2400	0	7 436,0000	2 004,4916	1 494,4143	52,56142
7	U7	1 652 068,	567028	452,1	N/A	N/A	2400	0	2 508,0000	688,3617	461,7687	52,56142

3 żel 3 powt YKL39 UMED 2018-02-28 11hr 09min_Exposure_60.0sec

No.	Label	Volume (In	Adj. Vol. (I	Mean Bkgr	Abs. Quan	Rel. Quant	# of Pixels	Min. Value	Max. Value	Mean Valu	Std. Dev.	Area (mm2
1	U1	7 352 684,	4490300	1 192,6600	N/A	N/A	2400	0	9 724,0000	3 063,6183	1 826,4232	46,61615
2	U2	8 306 684,	6132380	905,96	N/A	N/A	2400	0	13 708,000	3 461,1183	2 969,2930	46,61615
3	U3	10 018 196	7928036	870,9	N/A	N/A	2400	0	18 560,000	4 174,2483	4 118,0837	46,61615
4	U4	4 179 164,	2782508	581,94	N/A	N/A	2400	0	6 772,0000	1 741,3183	1 455,3745	46,61615
5	U5	3 635 424,	2376048	524,74	N/A	N/A	2400	0	7 052,0000	1 514,7600	1 355,9466	46,61615
6	U6	7 083 280,	5556160	636,3	N/A	N/A	2400	0	10 576,000	2 951,3666	2 416,2412	46,61615
7	U7	2 914 192,	1498864	589,72	N/A	N/A	2400	0	3 364,0000	1 214,2466	722,5347	46,61615

3 żel 1 powt tub UMED 2018-03-07 11hr 10min_Exposure_16.8sec

No.	Label	Volume (In	Adj. Vol. (I	Mean Bkgd	Abs. Quan	Rel. Quant	# of Pixels	Min. Value	Max. Value	Mean Valu	Std. Dev.	Area (mm2
1	U1	11 696 704	8244248	1 123,1150	N/A	N/A	3074	0	17 356,000	3 805,0435	4 174,2221	59,70752
2	U2	7 152 748,	5265530	613,9292	N/A	N/A	3074	0	14 612,000	2 326,8536	3 030,1852	59,70752
3	U3	10 491 068	8278604	719,7345	N/A	N/A	3074	0	16 916,000	3 412,8392	3 739,4686	59,70752
4	U4	10 373 072	8516267	604,0354	N/A	N/A	3074	0	18 152,000	3 374,4541	4 192,9318	59,70752
5	U5	7 579 696,	5534099	665,4513	N/A	N/A	3074	0	13 236,000	2 465,7436	2 635,1106	59,70752
6	U6	8 572 368,	6603539	640,4779	N/A	N/A	3074	0	13 872,000	2 788,6688	3 009,1954	59,70752
7	U7	5 858 952,	4138110	559,8053	N/A	N/A	3074	0	12 796,000	1 905,9700	2 304,4586	59,70752

3 zel 2 powt tub UMED 2018-03-01 10hr 48min_Exposure_16.8sec

No.	Label	Volume (In	Adj. Vol. (I	Mean Bkgd	Abs. Quan	Rel. Quant	# of Pixels	Min. Value	Max. Value	Mean Valu	Std. Dev.	Area (mm2
1	U1	16 815 112	13682951	1 018,9203	N/A	N/A	3074	0	39 152,000	5 470,1080	7 217,4417	66,02156
2	U2	8 656 812,	6770355	613,6814	N/A	N/A	3074	0	16 248,000	2 816,1392	3 875,4727	66,02156
3	U3	11 855 336	9985854	608,1593	N/A	N/A	3074	0	22 620,000	3 856,6480	4 898,1949	66,02156
4	U4	9 426 984,	7412507	655,3274	N/A	N/A	3074	0	24 540,000	3 066,6831	4 217,7114	66,02156
5	U5	9 370 860,	7349800	657,469	N/A	N/A	3074	0	17 440,000	3 048,4255	3 641,7403	66,02156
6	U6	11 280 604	9275649	652,2301	N/A	N/A	3074	0	20 516,000	3 669,6824	4 337,2510	66,02156
7	U7	8 645 472,	7025882	526,8673	N/A	N/A	3074	0	22 912,000	2 812,4502	3 958,9111	66,02156

3 żel 3 powt tub UMED 2018-03-01 10hr 55min_Exposure_16.8sec

No.	Label	Volume (In	Adj. Vol. (I	Mean Bkgd	Abs. Quan	Rel. Quant	# of Pixels	Min. Value	Max. Value	Mean Valu	Std. Dev.	Area (mm2
1	U1	20 994 736	15755334	1 704,4247	N/A	N/A	3074	0	33 812,000	6 829,7774	8 382,8915	59,70752
2	U2	12 322 092	9784056	825,646	N/A	N/A	3074	0	21 256,000	4 008,4879	5 297,9288	59,70752
3	U3	10 216 060	8361867	603,1858	N/A	N/A	3074	0	14 388,000	3 323,3767	3 633,8904	59,70752
4	U4	8 344 444,	6676269	542,6726	N/A	N/A	3074	0	12 096,000	2 714,5230	3 089,8847	59,70752
5	U5	6 744 448,	5373553	445,9646	N/A	N/A	3074	0	11 408,000	2 194,0299	2 706,7482	59,70752
6	U6	7 912 080,	6326005	515,9646	N/A	N/A	3074	0	10 676,000	2 573,8711	2 791,0924	59,70752
7	U7	7 207 224,	5256023	634,7434	N/A	N/A	3074	0	11 468,000	2 344,5751	2 928,1239	59,70752

CHI3L2protein normalisation to b-tubulin

membrane 1		membrane 2		membrane 3	
Adj.Prot/Adj Ref		Adj.Prot/Adj Ref.		Adj.Prot/Adj. Ref	
1,170568	G1	0,357901	G2	0,24724	G2
1,197786	G1	0,454882	G2	1,023769	G2
0,604147	G1	0,109224	G2	0,770193	G2
0,028284	G2	0,078615	G3	0,124745	G2
0,857437	G2	0,280508	G3	0,396458	G3
0,081982	G3	0,660915	G3	0,250672	G3
0,22214	G3	0,755998	G3	0,075627	G3
1,105123	G3				
0,432779	G1	0,366321	G2	0,28675	G2
0,309493	G1	0,431623	G2	0,637917	G2
0,214549	G1	0,128598	G3	0,518316	G2
-0,00785	G2	0,065642	G3	0,318888	G2
0,270674	G2	0,327205	G3	0,258477	G3
0,036302	G3	0,481006	G3	0,372845	G3
0,128843	G3	0,356066	G3	0,080706	G3
0,375498	G3				
1,386434	G1	0,782784	G2	0,285002	G2
1,082839	G1	0,797693	G2	0,626773	G2
1,224376	G1	0,134998	G3	0,948118	G2
0,093673	G2	0,174014	G3	0,416776	G2
1,743114	G2	0,549894	G3	0,442174	G3
0,300953	G3	0,899861	G3	0,878305	G3
0,449043	G3	0,432376	G3	0,285171	G3
1,842575	G3				

G1
G2
G3

CHI3L2/b-tub level	G1	G2	G3
	1,170568	0,028284	0,081982
	1,197786	0,857437	0,22214
	0,604147	0,007855	1,105123
	0,432779	0,270674	0,036302
	0,309493	0,093673	0,128843
	0,214549	1,743114	0,375498
	1,386434	0,357901	0,300953
	1,082839	0,454882	0,449043
	1,224376	0,109224	1,842575
		0,366321	0,078615
		0,431623	0,280508
		0,128598	0,660915
		0,782784	0,755998
		0,797693	0,065642
		0,134998	0,327205
		0,24724	0,481006
		1,023769	0,356066
		0,770193	0,174014
		0,124745	0,549894
		0,28675	0,899861
		0,637917	0,432376
		0,518316	0,396458
		0,318888	0,250672
		0,285002	0,075627
		0,626773	0,258477
		0,948118	0,372845
		0,416776	0,080706
			0,442174
			0,878305
			0,285171

	9	27	30
	G1	G2	G3
mean	0,846997	0,472946	0,4215
SD	0,452249	0,386935	5,241643
SEM	0,05025	0,014331	0,174721

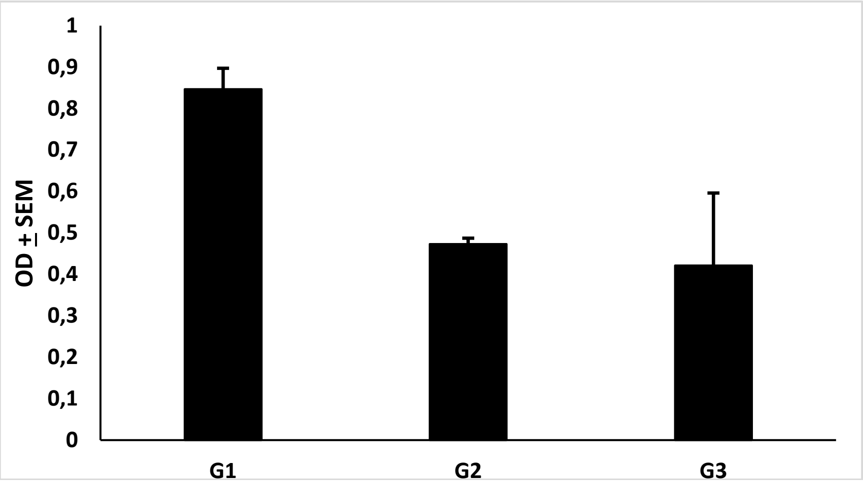

G1
G2
G3

Histologia 2023-01-24 13hr 36min_Exposure_3.0sec 1a

No.	Label	Type	Volume (In	Adj. Vol. (Ir	Mean Bkgd	Abs. Quant	Rel. Quant.	# of Pixels	Min. Value	Max. Value	Mean Valu	Std. Dev.	Area (mm2
1	U1	Unknown	12 477 660	6 082 188,	2 631,8811	N/A	N/A	2430	0	20 092,000	5 134,8395	5 285,3681	65,19247
2	U2	Unknown	14 173 812	7 905 133,	2 579,7029	N/A	N/A	2430	0	27 280,000	5 832,8444	6 545,6250	65,19247
3	U3	Unknown	14 293 308	5 360 724,	3 675,9603	N/A	N/A	2430	92	28 836,000	5 882,0197	5 916,8655	65,19247
4	U4	Unknown	15 555 536	7 221 983,	3 429,4455	N/A	N/A	2430	0	28 836,000	6 401,4551	6 756,2027	65,19247
5	U5	Unknown	15 141 056	5 375 487,	4 018,7524	N/A	N/A	2430	0	28 700,000	6 230,8872	6 178,3935	65,19247
6	U6	Unknown	17 525 120	9 143 400,	3 449,2673	N/A	N/A	2430	368	31 900,000	7 211,9835	7 488,4577	65,19247
7	U7	Unknown	13 682 548	4 927 137,	3 603,0495	N/A	N/A	2430	140	24 696,000	5 630,6781	5 896,1791	65,19247
8	U8	Unknown	11 981 928	7 120 965,	2 000,3960	N/A	N/A	2430	0	23 276,000	4 930,8345	5 652,6977	65,19247

Histologia 2023-01-26 12hr 02min_Exposure_3.0sec 1 b

No.	Label	Type	Volume (In	Adj. Vol. (Ir	Mean Bkgd	Abs. Quant	Rel. Quant.	# of Pixels	Min. Value	Max. Value	Mean Valu	Std. Dev.	Area (mm2
1	U1	Unknown	6 128 292,	3 181 255,	1 212,7722	N/A	N/A	2430	0	8 584,0000	2 521,9308	1 916,0225	65,19247
2	U2	Unknown	15 566 088	10 971 078	1 890,9504	N/A	N/A	2430	0	32 732,000	6 405,7975	7 707,4596	65,19247
3	U3	Unknown	10 423 404	3 989 918,	2 647,5247	N/A	N/A	2430	0	18 488,000	4 289,4666	4 463,9320	65,19247
4	U4	Unknown	13 786 312	6 626 665,	2 946,3564	N/A	N/A	2430	8	27 320,000	5 673,3794	5 795,9761	65,19247
5	U5	Unknown	16 700 004	8 669 744,	3 304,6336	N/A	N/A	2430	400	28 052,000	6 872,4296	6 785,6824	65,19247
6	U6	Unknown	15 661 852	7 456 151,	3 376,8316	N/A	N/A	2430	276	25 808,000	6 445,2065	6 625,0577	65,19247
7	U7	Unknown	16 096 108	8 343 253,	3 190,4752	N/A	N/A	2430	308	27 400,000	6 623,9127	6 764,2768	65,19247
8	U8	Unknown	12 174 172	7 731 650,	1 828,1980	N/A	N/A	2430	0	25 436,000	5 009,9473	5 722,0135	65,19247

Histologia 2023-01-27 18hr 02min_Exposure_3.0sec 1c ERK

No.	Label	Type	Volume (In	Adj. Vol. (Ir	Mean Bkgd	Abs. Quant	Rel. Quant.	# of Pixels	Min. Value	Max. Value	Mean Valu	Std. Dev.	Area (mm2
1	U1	Unknown	1 355 256,	686 981,94	275,0099	N/A	N/A	2430	0	2 676,0000	557,7185	512,7065	65,19247
2	U2	Unknown	6 230 140,	4 678 789,	638,4158	N/A	N/A	2430	0	14 604,000	2 563,8436	3 461,0790	65,19247
3	U3	Unknown	3 500 740,	1 364 553,	879,0891	N/A	N/A	2430	0	6 772,0000	1 440,6337	1 607,4318	65,19247
4	U4	Unknown	5 553 604,	3 155 073,	987,0495	N/A	N/A	2430	0	16 848,000	2 285,4337	3 249,6505	65,19247
5	U5	Unknown	6 250 780,	3 612 329,	1 085,7821	N/A	N/A	2430	0	15 528,000	2 572,3374	3 389,5649	65,19247
6	U6	Unknown	6 875 676,	4 620 154,	928,198	N/A	N/A	2430	0	16 904,000	2 829,4962	3 782,1963	65,19247
7	U7	Unknown	5 470 448,	3 763 914,	702,2772	N/A	N/A	2430	0	13 376,000	2 251,2131	3 033,0015	65,19247
8	U8	Unknown	2 941 328,	2 075 863,	356,1584	N/A	N/A	2430	0	8 984,0000	1 210,4230	1 694,2795	65,19247

Histologia 2023-01-24 13hr 38min_Exposure_3.0sec 2a

No.	Label	Type	Volume (In	Adj. Vol. (Ir	Mean Bkgd	Abs. Quant	Rel. Quant.	# of Pixels	Min. Value	Max. Value	Mean Valu	Std. Dev.	Area (mm2
1	U1	Unknown	11 897 488	8 072 908,[illegible]	1 573,9009	N/A	N/A	2430	0	21 688,000	4 896,0855	5 480,7422	65,19247
2	U2	Unknown	10 226 032	5 538 249,[illegible]	1 929,1287	N/A	N/A	2430	0	16 224,000	4 208,2436	4 242,0508	65,19247
3	U3	Unknown	9 103 428,[illegible]	3 627 651,[illegible]	2 253,4059	N/A	N/A	2430	108	14 696,000	3 746,2666	3 286,7910	65,19247
4	U4	Unknown	15 630 820	8 897 217,[illegible]	2 771,0297	N/A	N/A	2430	0	22 804,000	6 432,4362	5 691,4044	65,19247
5	U5	Unknown	10 232 276	3 510 607,[illegible]	2 766,1188	N/A	N/A	2430	0	23 772,000	4 210,8131	4 308,8218	65,19247
6	U6	Unknown	9 553 264,[illegible]	4 654 239,[illegible]	2 016,0594	N/A	N/A	2430	0	19 260,000	3 931,3843	4 261,9624	65,19247
7	U7	Unknown	7 761 864,[illegible]	5 529 439,[illegible]	918,6931	N/A	N/A	2430	0	14 928,000	3 194,1827	3 425,8771	65,19247

Histologia 2023-01-26 12hr 03min_Exposure_1.0sec 2b ERK

No.	Label	Type	Volume (In	Adj. Vol. (Ir	Mean Bkgd	Abs. Quant	Rel. Quant.	# of Pixels	Min. Value	Max. Value	Mean Valu	Std. Dev.	Area (mm2
1	U1	Unknown	11 513 596	7 299 879,[illegible]	1 734,0396	N/A	N/A	2430	0	33 868,000	4 738,1053	5 916,6073	65,19247
2	U2	Unknown	10 166 384	4 947 898,[illegible]	2 147,5247	N/A	N/A	2430	0	23 716,000	4 183,6971	5 093,3890	65,19247
3	U3	Unknown	12 379 220	7 059 685,[illegible]	2 189,1089	N/A	N/A	2430	0	21 448,000	5 094,3292	5 090,0104	65,19247
4	U4	Unknown	12 203 348	7 329 393,[illegible]	2 005,7425	N/A	N/A	2430	0	24 980,000	5 021,9539	5 978,7666	65,19247
5	U5	Unknown	12 324 188	6 921 888,[illegible]	2 223,1683	N/A	N/A	2430	0	26 232,000	5 071,6823	6 016,3782	65,19247
6	U6	Unknown	15 996 628	11 115 600	2 008,6534	N/A	N/A	2430	0	28 172,000	6 582,9744	6 666,6374	65,19247
7	U7	Unknown	11 407 928	8 042 161,[illegible]	1 385,0891	N/A	N/A	2430	0	31 356,000	4 694,6205	5 534,1343	65,19247

Histologia 2023-01-27 18hr 04min_Exposure_2.0sec 2c ERK

No.	Label	Type	Volume (In	Adj. Vol. (Ir	Mean Bkgd	Abs. Quant	Rel. Quant.	# of Pixels	Min. Value	Max. Value	Mean Valu	Std. Dev.	Area (mm2
1	U1	Unknown	4 710 024,[illegible]	3 700 924,[illegible]	415,2673	N/A	N/A	2430	0	11 940,000	1 938,2814	2 620,5978	65,19247
2	U2	Unknown	5 032 520,[illegible]	3 731 627,[illegible]	535,3465	N/A	N/A	2430	0	12 756,000	2 070,9958	2 848,3998	65,19247
3	U3	Unknown	3 890 908,[illegible]	2 400 812,[illegible]	613,2079	N/A	N/A	2430	0	12 024,000	1 601,1967	2 251,2065	65,19247
4	U4	Unknown	4 109 864,[illegible]	2 959 583,[illegible]	473,3663	N/A	N/A	2430	0	11 440,000	1 691,3020	2 348,1045	65,19247
5	U5	Unknown	3 799 088,[illegible]	2 731 283,[illegible]	439,4257	N/A	N/A	2430	0	12 548,000	1 563,4107	2 318,6485	65,19247
6	U6	Unknown	4 500 180,[illegible]	3 195 245,[illegible]	537,0099	N/A	N/A	2430	0	13 276,000	1 851,9259	2 590,6801	65,19247
7	U7	Unknown	3 543 228,[illegible]	2 868 072,[illegible]	277,8416	N/A	N/A	2430	0	10 640,000	1 458,1185	2 128,5795	65,19247

Histologia 2023-01-24 13hr 39min_Exposure_3.0sec 3a

No.	Label	Type	Volume (In	Adj. Vol. (Ir	Mean Bkgd	Abs. Quant	Rel. Quant.	# of Pixels	Min. Value	Max. Value	Mean Valu	Std. Dev.	Area (mm2
1	U1	Unknown	15 150 660	9 646 734,(	2 264,9900	N/A	N/A	2430	556	30 836,000	6 234,8395	5 875,6326	65,19247
2	U2	Unknown	9 622 076,(	5 020 233,4	1 893,7623	N/A	N/A	2430	0	18 564,000	3 959,7020	4 196,1421	65,19247
3	U3	Unknown	10 708 936	6 150 256,(	1 876,0000	N/A	N/A	2430	0	17 700,000	4 406,9695	4 252,5565	65,19247
4	U4	Unknown	9 248 560,(	5 455 402,1	1 560,9702	N/A	N/A	2430	0	18 756,000	3 805,9917	4 345,3327	65,19247
5	U5	Unknown	9 922 728,(	5 479 869,9	1 828,3366	N/A	N/A	2430	0	18 124,000	4 083,4271	4 214,2883	65,19247
6	U6	Unknown	10 721 060	6 739 805,7	1 638,3762	N/A	N/A	2430	0	17 356,000	4 411,9588	4 137,3849	65,19247
7	U7	Unknown	8 120 716,(	5 649 574,4	1 016,9306	N/A	N/A	2430	0	17 796,000	3 341,8584	3 600,6285	65,19247

Histologia 2023-01-26 12hr 04min_Exposure_3.0sec 3b

No.	Label	Type	Volume (In	Adj. Vol. (Ir	Mean Bkgd	Abs. Quant	Rel. Quant.	# of Pixels	Min. Value	Max. Value	Mean Valu	Std. Dev.	Area (mm2
1	U1	Unknown	14 167 400	9 340 698,2	1 986,2970	N/A	N/A	2430	0	30 348,000	5 830,2057	6 730,8803	65,19247
2	U2	Unknown	7 029 692,(	2 557 962,6	1 840,2178	N/A	N/A	2430	0	17 984,000	2 892,8773	3 296,4384	65,19247
3	U3	Unknown	8 884 092,(	5 606 142,2	1 348,9504	N/A	N/A	2430	0	12 784,000	3 656,0049	3 098,7989	65,19247
4	U4	Unknown	7 001 628,(	3 311 492,5	1 518,5742	N/A	N/A	2430	0	15 204,000	2 881,3283	3 002,5389	65,19247
5	U5	Unknown	8 027 108,(	4 131 505,2	1 603,1287	N/A	N/A	2430	0	14 280,000	3 303,3366	3 193,6660	65,19247
6	U6	Unknown	10 427 272	7 020 075,1	1 402,1386	N/A	N/A	2430	0	15 892,000	4 291,0584	3 775,3061	65,19247
7	U7	Unknown	7 812 684,(	5 135 449,5	1 101,7425	N/A	N/A	2430	0	15 640,000	3 215,0962	3 280,2063	65,19247

Histologia 2023-01-27 18hr 05min_Exposure_1.0sec 3c ERK

No.	Label	Type	Volume (In	Adj. Vol. (Ir	Mean Bkgd	Abs. Quant	Rel. Quant.	# of Pixels	Min. Value	Max. Value	Mean Valu	Std. Dev.	Area (mm2
1	U1	Unknown	5 978 024,(	4 267 304,(	704	N/A	N/A	2430	0	16 288,000	2 460,0921	3 295,7825	65,19247
2	U2	Unknown	3 573 652,(	2 177 388,4	574,5941	N/A	N/A	2430	0	8 668,0000	1 470,6386	1 731,3241	65,19247
3	U3	Unknown	1 534 156,(	224,5149	631,2475	N/A	N/A	2430	0	6 024,0000	631,3399	643,4798	65,19247
4	U4	Unknown	6 067 216,(	3 628 602,7	1 003,5445	N/A	N/A	2430	0	17 288,000	2 496,7967	3 325,1100	65,19247
5	U5	Unknown	4 671 736,(	2 593 869,4	855,0891	N/A	N/A	2430	0	15 644,000	1 922,5251	2 693,3521	65,19247
6	U6	Unknown	2 340 004,(	941 911,92	575,3465	N/A	N/A	2430	0	6 780,0000	962,9646	1 142,0537	65,19247
7	U7	Unknown	2 373 072,(	1 357 716,9	417,8416	N/A	N/A	2430	0	6 212,0000	976,5728	1 204,1818	65,19247

Histologia 2023-01-17 16hr 16min_Exposure_16.8sec 1a pERK

No.	Label	Type	Volume (In	Adj. Vol. (Int)	Mean Bkgd	Abs. Quant	Rel. Quant.	# of Pixels	Min. Value	Max. Value	Mean Valu	Std. Dev.	Area (mm2
1	U1	Unknown	12 354 792	7 048 819,298	2 364,5154	N/A	N/A	2244	0	29 308,000	5 505,7005	7 018,2142	60,20243
2	U2	Unknown	4 694 872,(	2 495 335,587	980,1856	N/A	N/A	2244	0	18 668,000	2 092,1889	3 051,5838	60,20243
3	U3	Unknown	5 845 432,(	3 202 832,824	1 177,6288	N/A	N/A	2244	0	18 668,000	2 604,9162	3 665,4015	60,20243
4	U4	Unknown	4 712 180,(	1 454 771,092	1 451,6082	N/A	N/A	2244	0	20 276,000	2 099,9019	3 235,9006	60,20243
5	U5	Unknown	4 457 640,(	3 276 046,762	526,5567	N/A	N/A	2244	0	20 276,000	1 986,4705	3 399,9278	60,20243
6	U6	Unknown	2 227 356,(	675 849,77319	691,4021	N/A	N/A	2244	0	8 680,0000	992,5829	1 385,9417	60,20243
7	U7	Unknown	2 718 272,(	1 643 696,742	478,866	N/A	N/A	2244	0	9 784,0000	1 211,3511	1 828,3459	60,20243
8	U8	Unknown	2 120 328,(	1 482 569,319	284,2062	N/A	N/A	2244	0	7 336,0000	944,8877	1 396,2260	60,20243

Histologia 2023-01-19 14hr 05min_Exposure_16.8sec 1b pERK

No.	Label	Type	Volume (In	Adj. Vol. (Int)	Mean Bkgd	Abs. Quant	Rel. Quant.	# of Pixels	Min. Value	Max. Value	Mean Valu	Std. Dev.	Area (mm2
1	U1	Unknown	16 442 829	13 284 294,89	1 407,5463	N/A	N/A	2244	0	65 535,000	7 327,4639	10 685,173	60,20243
2	U2	Unknown	3 870 804,(	1 803 501,649	921,2577	N/A	N/A	2244	0	13 848,000	1 724,9572	2 373,0875	60,20243
3	U3	Unknown	3 971 928,(	2 876 578,391	488,1237	N/A	N/A	2244	0	11 808,000	1 770,0213	2 295,0061	60,20243
4	U4	Unknown	3 800 912,(	2 604 235,381	533,2784	N/A	N/A	2244	0	13 504,000	1 693,8110	2 475,8889	60,20243
5	U5	Unknown	3 862 424,(	2 922 303,670	418,9485	N/A	N/A	2244	0	12 472,000	1 721,2228	2 509,3968	60,20243
6	U6	Unknown	1 132 576,(	391 547,05154	330,2268	N/A	N/A	2244	0	3 716,0000	504,713	645,8611	60,20243
7	U7	Unknown	2 583 836,(	1 736 853,237	377,4433	N/A	N/A	2244	0	8 944,0000	1 151,4420	1 601,9564	60,20243
8	U8	Unknown	2 963 936,(	2 135 552,989	369,1546	N/A	N/A	2244	0	9 616,0000	1 320,8270	1 920,3569	60,20243

Histologia 2023-01-19 14hr 17min_Exposure_16.8sec 1c pERK

No.	Label	Type	Volume (In	Adj. Vol. (Int)	Mean Bkgd	Abs. Quant	Rel. Quant.	# of Pixels	Min. Value	Max. Value	Mean Valu	Std. Dev.	Area (mm2
1	U1	Unknown	19 290 736	10 909 928,08	3 734,7628	N/A	N/A	2244	1 156,0000	47 668,000	8 596,5846	9 741,5991	60,20243
2	U2	Unknown	7 923 184,(	1 556 238,845	2 837,3195	N/A	N/A	2244	1 016,0000	12 064,000	3 530,8306	2 189,1061	60,20243
3	U3	Unknown	8 098 032,(	3 165 396,123	2 198,1443	N/A	N/A	2244	820	11 260,000	3 608,7486	2 473,7310	60,20243
4	U4	Unknown	6 177 048,(	2 209 887,340	1 767,8969	N/A	N/A	2244	280	13 964,000	2 752,6951	2 423,7014	60,20243
5	U5	Unknown	4 970 552,(	2 145 749,278	1 258,8247	N/A	N/A	2244	120	10 344,000	2 215,0409	2 035,3661	60,20243
6	U6	Unknown	3 069 312,(	306 184,57732	1 231,3402	N/A	N/A	2244	128	4 336,0000	1 367,7860	641,4129	60,20243
7	U7	Unknown	4 563 096,(	1 474 102,762	1 376,5567	N/A	N/A	2244	264	6 468,0000	2 033,4652	1 274,7738	60,20243
8	U8	Unknown	4 302 988,(	1 438 718,639	1 276,4123	N/A	N/A	2244	280	7 192,0000	1 917,5525	1 335,8483	60,20243

Histologia 2023-02-15 16hr 36min_Exposure_16.8sec 2a pERK

No.	Label	Type	Volume (In	Adj. Vol. (Int)	Mean Bkgd	Abs. Quant	Rel. Quant.	# of Pixels	Min. Value	Max. Value	Mean Valu	Std. Dev.	Area (mm2
1	U1	Unknown	3 051 676,(	1 607 234,020(	643,6907	N/A	N/A	2244	0	6 364,0000	1 359,9269	1 262,3875	60,20243
2	U2	Unknown	3 628 264,(	2 017 858,556	717,6495	N/A	N/A	2244	0	7 976,0000	1 616,8734	1 553,4116	60,20243
3	U3	Unknown	3 849 008,(	1 967 471,835(	838,4742	N/A	N/A	2244	0	8 684,0000	1 715,2442	1 764,0415	60,20243
4	U4	Unknown	3 526 936,(	1 885 530,969(	731,4639	N/A	N/A	2244	0	7 316,0000	1 571,7183	1 575,1738	60,20243
5	U5	Unknown	2 574 848,(	909 244,78350	742,2474	N/A	N/A	2244	0	4 796,0000	1 147,4367	893,4206	60,20243
6	U6	Unknown	3 091 784,(	1 752 971,958	596,6186	N/A	N/A	2244	0	8 008,0000	1 377,8003	1 461,0415	60,20243
7	U7	Unknown	1 319 508,(	494 780,16494	367,5258	N/A	N/A	2244	0	4 008,0000	588,016	592,1737	60,20243

Histologia 2023-01-17 16hr 18min_Exposure_16.8sec 2b pERK

No.	Label	Type	Volume (In	Adj. Vol. (Int)	Mean Bkgd	Abs. Quant	Rel. Quant.	# of Pixels	Min. Value	Max. Value	Mean Valu	Std. Dev.	Area (mm2
1	U1	Unknown	1 418 084,(	552 038,80412	385,9381	N/A	N/A	2244	0	4 580,0000	631,9447	777,1277	60,20243
2	U2	Unknown	5 637 296,(	2 694 185,896	1 311,5463	N/A	N/A	2244	0	15 520,000	2 512,1639	3 234,2979	60,20243
3	U3	Unknown	8 770 772,(	6 792 581,896	881,5464	N/A	N/A	2244	0	21 304,000	3 908,5436	4 980,1523	60,20243
4	U4	Unknown	981 592,00	-172 425,4845	514,268	N/A	N/A	2244	0	3 448,0000	437,4296	441,8745	60,20243
5	U5	Unknown	648 412,00	208 634,26804	195,9794	N/A	N/A	2244	0	1 524,0000	288,9537	278,7265	60,20243
6	U6	Unknown	3 755 880,(	2 815 898,474	418,8866	N/A	N/A	2244	0	11 464,000	1 673,7433	2 328,2275	60,20243
7	U7	Unknown	3 580 460,(	2 889 169,195	308,0619	N/A	N/A	2244	0	11 628,000	1 595,5704	2 220,9455	60,20243

Histologia 2023-01-19 14hr 07min_Exposure_16.8sec 2cpERK

No.	Label	Type	Volume (In	Adj. Vol. (Int)	Mean Bkgd	Abs. Quant	Rel. Quant.	# of Pixels	Min. Value	Max. Value	Mean Valu	Std. Dev.	Area (mm2
1	U1	Unknown	2 240 832,(	1 126 605,030	496,5361	N/A	N/A	2244	0	8 364,0000	998,5882	1 334,1038	60,20243
2	U2	Unknown	8 367 848,(	6 456 330,144	851,8351	N/A	N/A	2244	0	25 524,000	3 728,9875	5 334,3464	60,20243
3	U3	Unknown	8 778 904,(	6 586 168,989	977,1546	N/A	N/A	2244	0	22 192,000	3 912,1675	5 481,4381	60,20243
4	U4	Unknown	702 308,00	20 178,268041	303,9794	N/A	N/A	2244	0	2 024,0000	312,9715	353,8441	60,20243
5	U5	Unknown	613 372,00	191 592,53608	187,9588	N/A	N/A	2244	0	1 988,0000	273,3387	327,205	60,20243
6	U6	Unknown	4 690 704,(	3 858 850,886	370,701	N/A	N/A	2244	0	15 212,000	2 090,3315	3 174,3166	60,20243
7	U7	Unknown	5 341 440,(	3 797 105,319	688,2062	N/A	N/A	2244	0	16 348,000	2 380,3208	3 402,9315	60,20243

Histologia 2023-02-15 16hr 39min_Exposure_16.8sec 3a pERK

No.	Label	Type	Volume (In	Adj. Vol. (Int)	Mean Bkgd	Abs. Quant	Rel. Quant.	# of Pixels	Min. Value	Max. Value	Mean Valu	Std. Dev.	Area (mm2
1	U1	Unknown	13 521 596	9 715 263,051	1 696,2268	N/A	N/A	2244	204	28 404,000	6 025,6666	6 389,5215	60,20243
2	U2	Unknown	7 998 088,(	3 449 384,3298	2 027,0515	N/A	N/A	2244	0	15 444,000	3 564,2103	3 775,3329	60,20243
3	U3	Unknown	11 121 344	6 798 937,319	1 926,2061	N/A	N/A	2244	0	17 100,000	4 956,0356	4 186,4261	60,20243
4	U4	Unknown	7 699 604,(	4 360 300,659	1 488,1030	N/A	N/A	2244	0	16 260,000	3 431,1960	3 864,6792	60,20243
5	U5	Unknown	8 412 364,(	5 143 249,278	1 456,8247	N/A	N/A	2244	0	15 728,000	3 748,8253	3 765,0836	60,20243
6	U6	Unknown	8 058 520,(	4 801 388,701(	1 451,4845	N/A	N/A	2244	0	11 036,000	3 591,1408	2 685,3389	60,20243
7	U7	Unknown	5 664 636,(	3 277 806,556	1 063,6494	N/A	N/A	2244	0	9 092,0000	2 524,3475	1 932,3087	60,20243

Histologia 2023-01-17 16hr 21min_Exposure_16.8sec 3b pERK

No.	Label	Type	Volume (In	Adj. Vol. (Int)	Mean Bkgd	Abs. Quant	Rel. Quant.	# of Pixels	Min. Value	Max. Value	Mean Valu	Std. Dev.	Area (mm2
1	U1	Unknown	979 664,00	426 668,37113	246,433	N/A	N/A	2244	0	2 656,0000	436,5704	464,5158	60,20243
2	U2	Unknown	3 115 488,(	2 073 763,051	464,2268	N/A	N/A	2244	0	9 676,0000	1 388,3636	1 791,5954	60,20243
3	U3	Unknown	4 393 496,(	3 344 275,628	467,567	N/A	N/A	2244	0	11 700,000	1 957,8859	2 563,4105	60,20243
4	U4	Unknown	2 066 840,(	1 330 021,443	328,3505	N/A	N/A	2244	0	7 500,0000	921,0517	1 304,8278	60,20243
5	U5	Unknown	3 576 648,(	2 791 942,020	349,6907	N/A	N/A	2244	0	10 116,000	1 593,8716	2 252,2551	60,20243
6	U6	Unknown	3 016 968,(	2 071 850,721	421,1753	N/A	N/A	2244	0	6 620,0000	1 344,4598	1 570,5431	60,20243
7	U7	Unknown	3 876 192,(	2 887 675,298	440,5155	N/A	N/A	2244	0	10 032,000	1 727,3582	2 270,0761	60,20243

Histologia 2023-01-19 14hr 15min_Exposure_16.8sec 3c pERK

No.	Label	Type	Volume (In	Adj. Vol. (Int)	Mean Bkgd	Abs. Quant	Rel. Quant.	# of Pixels	Min. Value	Max. Value	Mean Valu	Std. Dev.	Area (mm2
1	U1	Unknown	576 060,00	218 916,98969	159,1546	N/A	N/A	2244	0	1 672,0000	256,7112	269,2324	60,20243
2	U2	Unknown	3 329 184,(	2 661 073,484	297,732	N/A	N/A	2244	0	12 900,000	1 483,5935	2 407,9073	60,20243
3	U3	Unknown	3 867 540,(	2 724 580,577	509,3402	N/A	N/A	2244	0	11 112,000	1 723,5026	2 179,7414	60,20243
4	U4	Unknown	2 263 760,(	1 316 190,515	422,268	N/A	N/A	2244	0	7 100,0000	1 008,8057	1 405,4684	60,20243
5	U5	Unknown	3 937 260,(	3 050 718,061	395,0722	N/A	N/A	2244	0	12 732,000	1 754,5721	2 485,0466	60,20243
6	U6	Unknown	2 897 656,(	1 858 475,793	463,0928	N/A	N/A	2244	0	5 848,0000	1 291,2905	1 427,7719	60,20243
7	U7	Unknown	3 857 212,(	3 110 214,474	332,8866	N/A	N/A	2244	0	12 224,000	1 718,9001	2 362,1257	60,20243

pERK1/2 protein normalisation to ERK1/2

membrane 1	membrane 2	membrane 3
Adj.Prot/Adj Ref	Adj.Prot/Adj Ref.	Adj.Prot/Adj. Ref
1,158928	0,19909	1,007104
0,31566	0,36435	0,687096
0,597463	0,542354	1,105472
0,201437	0,211924	0,799263
0,609442	0,258999	0,938571
0,073917	0,37664	0,712393
0,333601	0,089481	0,580186
0,208198		
4,175803	0,075623	0,045678
0,164387	0,544511	0,810709
0,720962	0,962165	0,596538
0,392993	0,023525	0,401638
0,337069	0,030141	0,675769
0,052513	0,253329	0,295132
0,208175	0,359253	0,562302
0,276209		
15,88095	0,304412	0,051301
0,332616	1,730164	1,22214
2,31973	2,743308	1,213541
0,700423	0,006818	0,362727
0,594007	0,070147	1,176126
0,066271	1,207685	1,973089
0,391641	1,323922	2,290768
0,69307		

G1
G2
G3

G1	G2	G3
1,158928	0,201437	0,073917
0,31566	0,609442	0,333601
0,597463	0,392993	0,208198
4,175803	0,337069	0,052513
0,164387	0,700423	0,208175
0,720962	0,594007	0,276209
1,588095	0,19909	0,066271
0,332616	0,36435	0,391641
2,31973	0,542354	0,69307
	0,075623	0,211924
	0,544511	0,258999
	0,962165	0,37664
	0,304412	0,089481
	1,730164	0,023525
	2,743308	0,030141
	1,007104	0,253329
	0,687096	0,359253
	1,105472	0,006818
	0,799263	0,070147
	0,045678	1,207685
	0,810709	1,323922
	0,596538	0,938571
	0,401638	0,712393
	0,051301	0,580186
	1,22214	0,675769
	1,213541	0,295132
	0,362727	0,562302
		1,176126
		1,973089
		2,290768

pERK/ERK

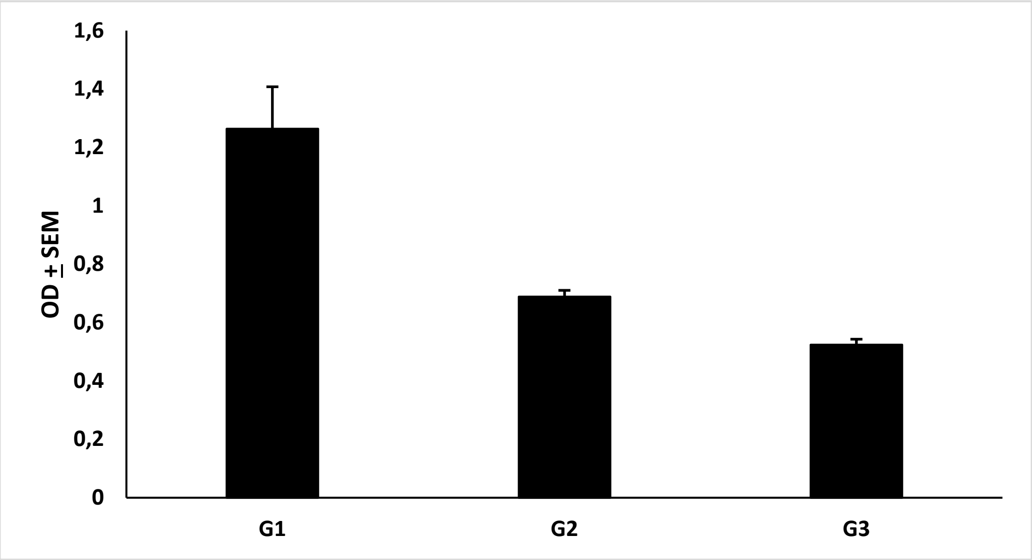

	G1	G2	G3
	9	27	30
śr	1,263738	0,689058	0,523993
SD	1,295395	0,576279	0,571305
SEM	0,143933	0,021344	0,019044

G1
G2
G3

Histologia 2021-09-21 13hr 43min_Exposure_120.0sec stat3 1a

No.	Label	Type	Volume (In	Adj. Vol. (Ir	Mean Bkgd	Abs. Quant	Rel. Quant.	# of Pixels	Min. Value	Max. Value	Mean Valu	Std. Dev.	Area (mm2
1	U1	Unknown	5 820 340,(	3 905 185,(	610,8947	N/A	N/A	3135	0	13 916,000	1 856,5677	2 629,4118	81,18117
2	U2	Unknown	13 997 372	10 213 427	1 207,0000	N/A	N/A	3135	0	35 484,000	4 464,8714	6 928,1412	81,18117
3	U3	Unknown	7 395 468,(	4 596 188,(	892,9123	N/A	N/A	3135	0	17 344,000	2 359,0009	3 489,6103	81,18117
4	U4	Unknown	7 298 624,(	5 119 909,(	694,9649	N/A	N/A	3135	0	18 296,000	2 328,1097	3 507,8370	81,18117
5	U5	Unknown	4 851 548,(	2 468 453,(	760,1579	N/A	N/A	3135	0	13 748,000	1 547,5432	2 244,5285	81,18117
6	U6	Unknown	6 228 112,(	4 673 152,(	496	N/A	N/A	3135	0	15 040,000	1 986,6385	3 035,1453	81,18117
7	U7	Unknown	10 803 460	8 984 940,(	580,0702	N/A	N/A	3135	0	33 636,000	3 446,0797	5 876,8998	81,18117
8	U8	Unknown	5 035 376,(	3 681 276,(	431,9298	N/A	N/A	3135	0	12 632,000	1 606,1805	2 463,0308	81,18117

Histologia 2023-01-24 13hr 48min_Exposure_60.0sec stat3 1b

No.	Label	Type	Volume (In	Adj. Vol. (Ir	Mean Bkgd	Abs. Quant	Rel. Quant.	# of Pixels	Min. Value	Max. Value	Mean Valu	Std. Dev.	Area (mm2
1	U1	Unknown	2 501 796,(	213 978,00	756,5536	N/A	N/A	3024	0	4 688,0000	827,3135	731,6829	81,12841
2	U2	Unknown	10 228 640	6 859 040,(	1 114,2857	N/A	N/A	3024	0	25 624,000	3 382,4867	4 840,7539	81,12841
3	U3	Unknown	5 142 084,(	2 330 304,(	929,8214	N/A	N/A	3024	0	13 468,000	1 700,4246	2 226,8483	81,12841
4	U4	Unknown	4 486 192,(	2 421 610,(	682,7321	N/A	N/A	3024	0	8 780,0000	1 483,5291	1 897,7753	81,12841
5	U5	Unknown	3 945 024,(	1 374 948,(	849,8929	N/A	N/A	3024	0	9 048,0000	1 304,5714	1 568,5939	81,12841
6	U6	Unknown	5 576 808,(	2 885 124,(	890,1071	N/A	N/A	3024	0	12 696,000	1 844,1825	2 409,3053	81,12841
7	U7	Unknown	8 748 568,(	5 764 204,(	986,8929	N/A	N/A	3024	0	22 696,000	2 893,0449	4 041,4198	81,12841
8	U8	Unknown	4 802 484,(	3 068 328,(	573,4643	N/A	N/A	3024	0	9 484,0000	1 588,1230	2 171,2901	81,12841

Histologia 2021-09-21 13hr 43min_Exposure_120.0sec stat3 1c

No.	Label	Type	Volume (In	Adj. Vol. (Ir	Mean Bkgd	Abs. Quant	Rel. Quant.	# of Pixels	Min. Value	Max. Value	Mean Valu	Std. Dev.	Area (mm2
1	U1	Unknown	5 693 560,(	3 953 635,(	555	N/A	N/A	3135	0	13 916,000	1 816,1275	2 640,0599	81,18117
2	U2	Unknown	13 952 352	10 769 007	1 015,4210	N/A	N/A	3135	0	35 484,000	4 450,5110	6 925,0118	81,18117
3	U3	Unknown	7 424 660,(	4 800 335,(	837,1053	N/A	N/A	3135	0	17 344,000	2 368,3126	3 483,0634	81,18117
4	U4	Unknown	7 336 276,(	5 099 866,(	713,3684	N/A	N/A	3135	0	18 296,000	2 340,1199	3 502,1335	81,18117
5	U5	Unknown	4 978 480,(	1 950 510,(	965,8596	N/A	N/A	3135	0	13 748,000	1 588,0318	2 288,1893	81,18117
6	U6	Unknown	6 486 636,(	3 228 051,(	1 039,4210	N/A	N/A	3135	0	15 040,000	2 069,1023	3 087,2161	81,18117
7	U7	Unknown	10 797 944	8 926 844,(	596,8421	N/A	N/A	3135	0	33 636,000	3 444,3202	5 877,4463	81,18117
8	U8	Unknown	4 986 284,(	3 695 764,(	411,6491	N/A	N/A	3135	0	12 632,000	1 590,5212	2 467,9782	81,18117

Histologia 2021-09-22 15hr 42min_Exposure_120.0sec stat3 2a

No.	Label	Type	Volume (In	Adj. Vol. (Ir	Mean Bkgd	Abs. Quant	Rel. Quant.	# of Pixels	Min. Value	Max. Value	Mean Valu	Std. Dev.	Area (mm2
1	U1	Unknown	12 442 128	10 577 408	594,807	N/A	N/A	3135	0	32 696,000	3 968,7808	6 475,0971	81,18117
2	U2	Unknown	11 584 444	9 439 389,(	684,2281	N/A	N/A	3135	0	33 004,000	3 695,1974	6 178,2600	81,18117
3	U3	Unknown	6 112 812,(	3 089 682,(	964,3158	N/A	N/A	3135	0	48 088,000	1 949,8602	2 848,0435	81,18117
4	U4	Unknown	10 935 832	8 087 492,(	908,5614	N/A	N/A	3135	0	23 312,000	3 488,3036	4 664,7364	81,18117
5	U5	Unknown	10 367 180	5 191 680,(	1 650,8771	N/A	N/A	3135	0	16 312,000	3 306,9154	3 151,7693	81,18117
6	U6	Unknown	16 447 416	12 743 771	1 181,3859	N/A	N/A	3135	0	37 648,000	5 246,3846	7 663,2505	81,18117
7	U7	Unknown	14 189 332	12 101 972	665,8246	N/A	N/A	3135	0	49 880,000	4 526,1027	7 804,0487	81,18117

Histologia 2023-01-24 13hr 50min_Exposure_60.0sec stat3 2b

No.	Label	Type	Volume (In	Adj. Vol. (Ir	Mean Bkgd	Abs. Quant	Rel. Quant.	# of Pixels	Min. Value	Max. Value	Mean Valu	Std. Dev.	Area (mm2
1	U1	Unknown	10 349 624	7 453 334,(	957,7679	N/A	N/A	3024	0	24 676,000	3 422,4947	5 147,5633	81,12841
2	U2	Unknown	6 383 576,(	2 608 166,(	1 248,4821	N/A	N/A	3024	0	17 364,000	2 110,9708	2 946,8354	81,12841
3	U3	Unknown	5 854 352,(	2 906 762,(	974,7321	N/A	N/A	3024	0	11 660,000	1 935,9629	2 573,6857	81,12841
4	U4	Unknown	6 540 888,(	4 206 090,(	772,0893	N/A	N/A	3024	0	13 532,000	2 162,9920	3 209,6965	81,12841
5	U5	Unknown	3 989 016,(	1 914 174,(	686,125	N/A	N/A	3024	0	7 972,0000	1 319,1190	1 646,0751	81,12841
6	U6	Unknown	6 782 516,(	4 799 852,(	655,6429	N/A	N/A	3024	0	12 484,000	2 242,8955	3 105,5659	81,12841
7	U7	Unknown	5 246 536,(	3 682 750,(	517,125	N/A	N/A	3024	0	11 764,000	1 734,9656	2 381,6959	81,12841

Histologia 2021-09-22 15hr 42min_Exposure_120.0sec stat3 2c

No.	Label	Type	Volume (In	Adj. Vol. (Ir	Mean Bkgd	Abs. Quant	Rel. Quant.	# of Pixels	Min. Value	Max. Value	Mean Valu	Std. Dev.	Area (mm2
1	U1	Unknown	12 322 724	10 314 729	640,5088	N/A	N/A	3135	0	32 696,000	3 930,6934	6 491,2130	81,18117
2	U2	Unknown	11 564 196	8 952 631,(	833,0351	N/A	N/A	3135	0	33 004,000	3 688,7387	6 181,1094	81,18117
3	U3	Unknown	6 174 348,(	2 206 593,(	1 265,6315	N/A	N/A	3135	0	48 088,000	1 969,4889	2 885,9043	81,18117
4	U4	Unknown	10 952 192	8 102 917,(	908,8596	N/A	N/A	3135	0	23 312,000	3 493,5221	4 661,5181	81,18117
5	U5	Unknown	10 595 464	3 766 554,(	2 178,2807	N/A	N/A	3135	0	17 160,000	3 379,7333	3 218,8639	81,18117
6	U6	Unknown	15 906 448	11 377 913	1 444,5087	N/A	N/A	3135	0	37 648,000	5 073,8271	7 748,5034	81,18117
7	U7	Unknown	13 904 320	10 968 035	936,614	N/A	N/A	3135	0	49 880,000	4 435,1897	7 830,4141	81,18117

Histologia 2021-09-17 13hr 50min_Exposure_120.0sec stat3 3a

No.	Label	Type	Volume (In	Adj. Vol. (Ir	Mean Bkgd	Abs. Quant	Rel. Quant.	# of Pixels	Min. Value	Max. Value	Mean Valu	Std. Dev.	Area (mm2
1	U1	Unknown	9 193 504,(	7 114 669,(	663,1053	N/A	N/A	3135	0	19 488,000	2 932,5371	4 054,7947	81,80697
2	U2	Unknown	4 315 720,(	2 088 055,(	710,5789	N/A	N/A	3135	0	11 240,000	1 376,6251	1 926,3564	81,80697
3	U3	Unknown	3 597 964,(	1 890 874,(	544,5263	N/A	N/A	3135	0	7 684,0000	1 147,6759	1 474,4786	81,80697
4	U4	Unknown	7 611 464,(	5 920 434,(	539,4035	N/A	N/A	3135	0	19 384,000	2 427,8992	4 054,2896	81,80697
5	U5	Unknown	3 835 032,(	830 217,00	958,4737	N/A	N/A	3135	0	7 632,0000	1 223,2956	1 457,1633	81,80697
6	U6	Unknown	7 048 300,(	4 544 480,(	798,6667	N/A	N/A	3135	0	14 476,000	2 248,2615	3 180,0779	81,80697
7	U7	Unknown	12 006 700	9 556 175,(	781,6667	N/A	N/A	3135	0	39 316,000	3 829,8883	6 461,2782	81,80697

Histologia 2023-01-24 13hr 52min_Exposure_60.0sec stat3 3b

No.	Label	Type	Volume (In	Adj. Vol. (Ir	Mean Bkgd	Abs. Quant	Rel. Quant.	# of Pixels	Min. Value	Max. Value	Mean Valu	Std. Dev.	Area (mm2
1	U1	Unknown	9 713 544,(	6 314 406,(	1 124,0535	N/A	N/A	3024	0	21 204,000	3 212,1507	3 780,5370	81,12841
2	U2	Unknown	5 531 904,(	1 937 070,(	1 188,7678	N/A	N/A	3024	0	14 388,000	1 829,3333	1 697,7751	81,12841
3	U3	Unknown	6 082 872,(	2 402 718,(	1 216,9821	N/A	N/A	3024	0	9 316,0000	2 011,5317	1 892,5348	81,12841
4	U4	Unknown	7 593 396,(	4 069 140,(	1 165,4285	N/A	N/A	3024	0	18 592,000	2 511,0436	2 963,9247	81,12841
5	U5	Unknown	6 677 200,(	2 294 506,(	1 449,3035	N/A	N/A	3024	0	9 860,0000	2 208,0687	1 896,0455	81,12841
6	U6	Unknown	8 257 128,(	4 711 704,(	1 172,4285	N/A	N/A	3024	0	12 596,000	2 730,5317	2 900,5499	81,12841
7	U7	Unknown	6 241 668,(	4 145 226,(	693,2679	N/A	N/A	3024	0	14 004,000	2 064,0436	2 582,9773	81,12841

Histologia 2021-09-17 13hr 50min_Exposure_120.0sec stat3 3c

No.	Label	Type	Volume (In	Adj. Vol. (Ir	Mean Bkgd	Abs. Quant	Rel. Quant.	# of Pixels	Min. Value	Max. Value	Mean Valu	Std. Dev.	Area (mm2
1	U1	Unknown	9 147 896,(	7 260 901,(	601,9123	N/A	N/A	3135	0	19 488,000	2 917,9891	4 062,2622	81,80697
2	U2	Unknown	4 280 224,(	2 268 269,(	641,7719	N/A	N/A	3135	0	11 240,000	1 365,3027	1 931,5170	81,80697
3	U3	Unknown	3 594 660,(	1 919 800,(	534,2456	N/A	N/A	3135	0	7 684,0000	1 146,6220	1 474,9831	81,80697
4	U4	Unknown	7 635 004,(	5 922 194,(	546,3509	N/A	N/A	3135	0	19 384,000	2 435,4079	4 050,8222	81,80697
5	U5	Unknown	3 733 100,(	1 630 725,(	670,614	N/A	N/A	3135	0	7 632,0000	1 190,7814	1 440,6460	81,80697
6	U6	Unknown	7 250 808,(	3 813 088,(	1 096,5614	N/A	N/A	3135	0	14 476,000	2 312,8574	3 171,3226	81,80697
7	U7	Unknown	12 055 200	9 389 680,(	850,2456	N/A	N/A	3135	0	39 316,000	3 845,3588	6 454,1423	81,80697

G1
G2
G3

Histologia 2023-01-12 13hr 58min_Exposure_16.8sec pstat3 1a

No.	Label	Type	Volume (In	Adj. Vol. (Ir	Mean Bkgd	Abs. Quant	Rel. Quant.	# of Pixels	Min. Value	Max. Value	Mean Valu	Std. Dev.	Area (mm2
1	U1	Unknown	12 850 956	-1 930 504,	5 084,7818	N/A	N/A	2907	2 744,0000	10 356,000	4 420,6934	942,4353	77,98951
2	U2	Unknown	13 878 736	1 023 664,	4 422,1090	N/A	N/A	2907	2 376,0000	16 132,000	4 774,2469	2 029,1927	77,98951
3	U3	Unknown	14 544 132	3 954 723,	3 642,7272	N/A	N/A	2907	1 568,0000	19 812,000	5 003,1413	3 536,4141	77,98951
4	U4	Unknown	7 934 428,	738 017,36	2 475,5454	N/A	N/A	2907	1 136,0000	6 240,0000	2 729,4213	942,3644	77,98951
5	U5	Unknown	7 044 908,	894 805,94	2 115,6181	N/A	N/A	2907	940	7 144,0000	2 423,4289	952,1264	77,98951
6	U6	Unknown	5 520 916,	460 780,38	1 740,6727	N/A	N/A	2907	468	4 920,0000	1 899,1799	653,7821	77,98951
7	U7	Unknown	6 705 224,	1 682 456,	1 727,8181	N/A	N/A	2907	564	8 152,0000	2 306,5786	1 267,9675	77,98951
8	U8	Unknown	6 093 932,	1 648 494,	1 529,2181	N/A	N/A	2907	496	6 596,0000	2 096,2958	1 245,1209	77,98951

Histologia 2023-01-12 14hr 11min_Exposure_16.8sec pstat3 1b

No.	Label	Type	Volume (In	Adj. Vol. (Ir	Mean Bkgd	Abs. Quant	Rel. Quant.	# of Pixels	Min. Value	Max. Value	Mean Valu	Std. Dev.	Area (mm2
1	U1	Unknown	12 623 276	500 716,01	4 170,1272	N/A	N/A	2907	2 616,0000	7 588,0000	4 342,3722	748,1004	77,98951
2	U2	Unknown	17 972 832	1 668 261,	5 608,7272	N/A	N/A	2907	3 004,0000	32 916,000	6 182,6047	3 489,2333	77,98951
3	U3	Unknown	18 569 412	5 651 021,	4 443,8909	N/A	N/A	2907	1 780,0000	35 116,000	6 387,8266	5 338,0326	77,98951
4	U4	Unknown	10 028 064	530 895,00	3 267,0000	N/A	N/A	2907	1 364,0000	7 224,0000	3 449,6264	1 023,7457	77,98951
5	U5	Unknown	10 190 040	1 865 449,	2 863,6363	N/A	N/A	2907	1 484,0000	10 652,000	3 505,3457	1 477,7237	77,98951
6	U6	Unknown	8 987 332,	563 215,98	2 897,8727	N/A	N/A	2907	1 224,0000	6 096,0000	3 091,6174	701,8411	77,98951
7	U7	Unknown	10 066 384	1 510 871,	2 943,0727	N/A	N/A	2907	1 288,0000	8 204,0000	3 462,8083	1 208,5086	77,98951
8	U8	Unknown	10 450 932	1 988 126,	2 911,1818	N/A	N/A	2907	1 576,0000	9 892,0000	3 595,0918	1 512,1606	77,98951

Histologia 2023-01-13 14hr 38min_Exposure_16.8sec pstat3 1c

No.	Label	Type	Volume (In	Adj. Vol. (Ir	Mean Bkgd	Abs. Quant	Rel. Quant.	# of Pixels	Min. Value	Max. Value	Mean Valu	Std. Dev.	Area (mm2
1	U1	Unknown	6 291 952,	304 377,67	2 059,7090	N/A	N/A	2907	864	5 656,0000	2 164,4141	668,1362	77,98951
2	U2	Unknown	7 841 404,	1 419 471,	2 209,1272	N/A	N/A	2907	804	13 608,000	2 697,4213	1 851,0634	77,98951
3	U3	Unknown	9 890 016,	4 386 483,	1 893,2000	N/A	N/A	2907	544	19 128,000	3 402,1382	3 526,9471	77,98951
4	U4	Unknown	5 889 496,	-85 604,65	2 055,4181	N/A	N/A	2907	472	15 584,000	2 025,9704	1 541,2086	77,98951
5	U5	Unknown	5 767 260,	1 182 868,	1 577,0181	N/A	N/A	2907	420	9 040,0000	1 983,9215	1 333,4101	77,98951
6	U6	Unknown	5 818 732,	687 665,58	1 765,0727	N/A	N/A	2907	376	7 796,0000	2 001,6277	1 167,5787	77,98951
7	U7	Unknown	6 718 740,	1 719 439,	1 719,7454	N/A	N/A	2907	496	9 904,0000	2 311,2280	1 567,2442	77,98951
8	U8	Unknown	6 770 580,	2 266 157,	1 549,5090	N/A	N/A	2907	392	9 564,0000	2 329,0608	1 699,0131	77,98951

Histologia 2023-01-12 14hr 01min_Exposure_16.8sec pstat3 2a

No.	Label	Type	Volume (In	Adj. Vol. (Ir	Mean Bkgd	Abs. Quant	Rel. Quant.	# of Pixels	Min. Value	Max. Value	Mean Valu	Std. Dev.	Area (mm2
1	U1	Unknown	17 054 448	-389 877,6(	6 000,8000	N/A	N/A	2907	3 340,0000	15 232,000	5 866,6831	1 910,2725	77,98951
2	U2	Unknown	14 465 040	886 918,69	4 670,8363	N/A	N/A	2907	3 040,0000	13 104,000	4 975,9339	1 607,2665	77,98951
3	U3	Unknown	10 991 756	-314 095,5	3 889,1818	N/A	N/A	2907	1 252,0000	7 460,0000	3 781,1338	957,892	77,98951
4	U4	Unknown	10 484 816	1 341 772,4	3 145,1818	N/A	N/A	2907	912	7 968,0000	3 606,7478	1 230,0396	77,98951
5	U5	Unknown	11 317 608	1 471 704,1	3 386,9636	N/A	N/A	2907	1 656,0000	10 444,000	3 893,2260	1 490,3315	77,98951
6	U6	Unknown	11 595 080	866 294,38	3 690,6727	N/A	N/A	2907	1 896,0000	9 788,0000	3 988,6756	1 326,7637	77,98951
7	U7	Unknown	14 455 064	2 060 514,5	4 263,6909	N/A	N/A	2907	2 352,0000	13 592,000	4 972,5022	1 758,1865	77,98951

Histologia 2023-01-13 14hr 41min_Exposure_16.8sec pstat3 2b

No.	Label	Type	Volume (In	Adj. Vol. (Ir	Mean Bkgd	Abs. Quant	Rel. Quant.	# of Pixels	Min. Value	Max. Value	Mean Valu	Std. Dev.	Area (mm2
1	U1	Unknown	8 514 096,(	1 057 852,4	2 564,9272	N/A	N/A	2907	728	9 528,0000	2 928,8255	1 369,8528	77,98951
2	U2	Unknown	6 956 488,(	1 133 185,(	2 003,2000	N/A	N/A	2907	592	8 760,0000	2 393,0127	1 235,5693	77,98951
3	U3	Unknown	5 019 812,(	294 721,34	1 625,4181	N/A	N/A	2907	312	4 292,0000	1 726,8015	507,8512	77,98951
4	U4	Unknown	4 755 252,(	647 925,27	1 412,9090	N/A	N/A	2907	356	4 484,0000	1 635,7936	737,0681	77,98951
5	U5	Unknown	5 148 812,(	1 342 016,1	1 309,5272	N/A	N/A	2907	332	6 352,0000	1 771,1771	1 013,3791	77,98951
6	U6	Unknown	5 075 228,(	1 058 652,5	1 381,6909	N/A	N/A	2907	348	6 068,0000	1 745,8644	904,171	77,98951
7	U7	Unknown	4 718 920,(	1 022 008,8	1 271,7272	N/A	N/A	2907	208	7 248,0000	1 623,2954	980,6456	77,98951

Histologia 2023-01-19 14hr 27min_Exposure_16.8sec pstat3 2c

No.	Label	Type	Volume (In	Adj. Vol. (Ir	Mean Bkgd	Abs. Quant	Rel. Quant.	# of Pixels	Min. Value	Max. Value	Mean Valu	Std. Dev.	Area (mm2
1	U1	Unknown	15 081 088	3 012 968,1	4 151,4000	N/A	N/A	2907	2 308,0000	14 268,000	5 187,8527	2 456,6554	77,98951
2	U2	Unknown	13 151 992	2 790 651,1	3 564,2727	N/A	N/A	2907	2 372,0000	15 340,000	4 524,2490	2 035,7153	77,98951
3	U3	Unknown	10 090 628	760 585,07	3 209,5090	N/A	N/A	2907	2 068,0000	6 804,0000	3 471,1482	786,5251	77,98951
4	U4	Unknown	9 384 580,(	1 410 256,1	2 743,1454	N/A	N/A	2907	1 460,0000	7 268,0000	3 228,2696	1 059,7297	77,98951
5	U5	Unknown	9 815 372,(	2 078 999,3	2 661,2909	N/A	N/A	2907	1 188,0000	10 312,000	3 376,4609	1 566,4162	77,98951
6	U6	Unknown	9 326 856,(	1 388 050,4	2 730,9272	N/A	N/A	2907	1 296,0000	9 256,0000	3 208,4127	1 344,0103	77,98951
7	U7	Unknown	9 320 988,(	3 796 630,9	1 900,3636	N/A	N/A	2907	0	10 684,000	3 206,3942	1 841,7495	77,98951

Histologia 2023-01-12 14hr 09min_Exposure_16.8sec pstat3 3a

No.	Label	Type	Volume (In	Adj. Vol. (Ir	Mean Bkgd	Abs. Quant	Rel. Quant.	# of Pixels	Min. Value	Max. Value	Mean Valu	Std. Dev.	Area (mm2
1	U1	Unknown	17 171 564	-235 763,4	5 988,0727	N/A	N/A	2907	3 788,0000	12 000,000	5 906,9707	1 375,4372	77,98951
2	U2	Unknown	17 266 020	1 319 539,	5 485,5454	N/A	N/A	2907	3 644,0000	14 124,000	5 939,4633	1 265,0463	77,98951
3	U3	Unknown	21 699 428	9 470 048,	4 206,8727	N/A	N/A	2907	2 528,0000	28 268,000	7 464,5435	5 674,9581	77,98951
4	U4	Unknown	11 623 336	1 443 127,	3 501,9636	N/A	N/A	2907	2 120,0000	12 628,000	3 998,3955	1 683,8609	77,98951
5	U5	Unknown	11 284 672	1 095 214,	3 505,1454	N/A	N/A	2907	1 604,0000	10 952,000	3 881,8961	1 612,5684	77,98951
6	U6	Unknown	17 294 248	7 786 085,	3 270,7818	N/A	N/A	2907	1 500,0000	24 192,000	5 949,1737	5 134,2985	77,98951
7	U7	Unknown	12 907 672	3 726 044,	3 158,4545	N/A	N/A	2907	1 704,0000	21 820,000	4 440,2036	2 941,1957	77,98951

Histologia 2023-01-13 14hr 43min_Exposure_16.8sec pstat3 3b

No.	Label	Type	Volume (In	Adj. Vol. (Ir	Mean Bkgd	Abs. Quant	Rel. Quant.	# of Pixels	Min. Value	Max. Value	Mean Valu	Std. Dev.	Area (mm2
1	U1	Unknown	9 189 964,	548 932,92	2 972,4909	N/A	N/A	2907	1 532,0000	7 496,0000	3 161,3223	938,9509	77,98951
2	U2	Unknown	9 570 436,	1 339 926,	2 831,2727	N/A	N/A	2907	1 472,0000	8 572,0000	3 292,2036	1 208,5970	77,98951
3	U3	Unknown	13 281 660	5 296 078,	2 747,0181	N/A	N/A	2907	1 464,0000	17 592,000	4 568,8544	3 449,1467	77,98951
4	U4	Unknown	7 795 720,	1 068 340,	2 314,2000	N/A	N/A	2907	692	9 208,0000	2 681,7062	1 373,9140	77,98951
5	U5	Unknown	7 406 872,	743 605,16	2 292,1454	N/A	N/A	2907	676	10 504,000	2 547,9435	1 439,5664	77,98951
6	U6	Unknown	10 896 880	3 831 918,	2 430,3272	N/A	N/A	2907	812	15 152,000	3 748,4967	3 109,6552	77,98951
7	U7	Unknown	7 491 528,	1 916 007,	1 917,9636	N/A	N/A	2907	484	10 092,000	2 577,0650	1 656,1189	77,98951

Histologia 2023-01-19 14hr 30min_Exposure_16.8sec pstat3 3c

No.	Label	Type	Volume (In	Adj. Vol. (Ir	Mean Bkgd	Abs. Quant	Rel. Quant.	# of Pixels	Min. Value	Max. Value	Mean Valu	Std. Dev.	Area (mm2
1	U1	Unknown	12 052 068	2 831 539,	3 171,8363	N/A	N/A	2907	1 756,0000	11 636,000	4 145,8782	1 792,7897	77,98951
2	U2	Unknown	12 200 720	2 880 508,	3 206,1272	N/A	N/A	2907	1 864,0000	16 692,000	4 197,0141	2 422,0287	77,98951
3	U3	Unknown	16 903 128	7 935 244,	3 084,9272	N/A	N/A	2907	1 576,0000	24 868,000	5 814,6295	5 017,2736	77,98951
4	U4	Unknown	8 813 764,	1 698 062,	2 447,7818	N/A	N/A	2907	676	9 452,0000	3 031,9105	1 721,5155	77,98951
5	U5	Unknown	8 443 084,	1 474 370,	2 397,2181	N/A	N/A	2907	1 164,0000	8 720,0000	2 904,3976	1 464,9280	77,98951
6	U6	Unknown	15 150 544	7 795 041,	2 530,2727	N/A	N/A	2907	1 308,0000	20 088,000	5 211,7454	4 420,6519	77,98951
7	U7	Unknown	10 889 312	3 061 500,	2 692,7454	N/A	N/A	2907	1 444,0000	12 620,000	3 745,8933	2 127,9376	77,98951

pSTAT-3 protein normalisation to STAT-3

membrane 1	membrane 2	membrane 3
Adj.Prot/Adj Ref	Adj.Prot/Adj Ref.	Adj.Prot/Adj. Ref
0,126647002	0,284849	0,397986
0,154913245	0,295639	1,379517
1,177213908	0,246169	4,196601
0,104099794	0,174375	0,286814
0,956390427	0,400448	1,775886
0,174475553	0,10892	1,715277
0,169250362	0,31372	0,320369
0,537947351		
1,422471809	0,14193	0,075601
0,206948934	0,434476	0,590726
1,882365391	0,101392	2,758661
-0,035350306	0,154045	0,180396
0,860300277	0,701094	0,455997
0,238348709	0,220559	1,004938
0,298296168	0,277512	0,204055
0,738564154		
-0,494343993	-0,0378	-0,03734
0,100227365	0,099068	0,681204
0,860435608	-0,14234	3,94139
0,144146578	0,165591	0,354652
0,362496651	0,39073	0,47732
0,098601625	0,076138	1,652499
0,187252953	0,187865	0,898876
0,447805257		

G1
G2
G3

pSTAT-3/STAT-3 level

G1	G2	G3
0,126647	0,1041	0,174476
0,154913	0,95639	0,16925
1,177214	0,03535	0,537947
1,422472	0,8603	0,238349
0,206949	0,144147	0,298296
1,882365	0,362497	0,738564
0,494344	0,284849	0,098602
0,100227	0,295639	0,187253
0,860436	0,246169	0,447805
	0,14193	0,174375
	0,434476	0,400448
	0,101392	0,10892
	0,037798	0,31372
	0,099068	0,154045
	0,142344	0,701094
	0,397986	0,220559
	1,379517	0,277512
	4,196601	0,165591
	0,286814	0,39073
	0,075601	0,076138
	0,590726	0,187865
	2,758661	1,775886
	0,180396	1,715277
	0,037337	0,320369
	0,681204	0,455997
	3,94139	1,004938
	0,354652	0,204055
		0,47732
		1,652499
		0,898876

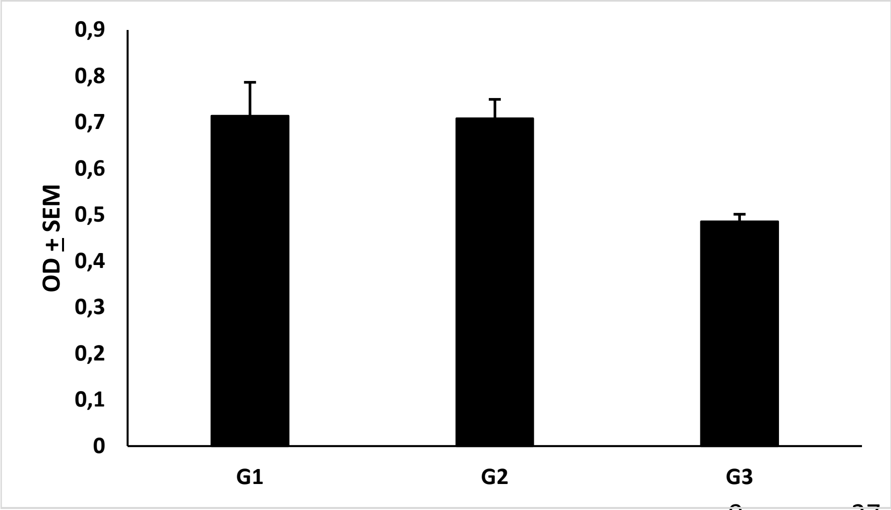

	9	27	30
mean	0,713952	0,70842	0,485559
SD	0,655996	1,120218	0,477053
SEM	0,072888	0,04149	0,015902

pSTAT-3/STAT-3
